# Supplementary material for: Ni(II)-catalyzed asymmetric alkenylations of ketimines
Source: Nat Commun. 2018 Jun 8;9:2258. doi: 10.1038/s41467-018-04645-3 (PMC5993804; doi:10.1038/s41467-018-04645-3)
Supplement: Supplementary file 1 — Supplementary Information [file 41467_2018_4645_MOESM1_ESM.pdf]

Supplementary Information for:

"Ni(II)-Catalyzed Asymmetric Alkenylations of Ketimines"

Mao Quan et al.

Supplementary Methods

All air and moisture sensitive manipulations were carried out with standard Schlenk techniques nitrogen atmosphere. Column chromatography was performed using 100-200 mesh silica gels. All the reagents were purchased from Adamas-Beta Ltd., Energy Chemical Inc. or *J&K* Scientific Inc. and used without further purification unless otherwise specified. The NMR spectra were recorded on a Varian MERCURY plus-400 (400 MHz, ^{1}H; 100 MHz, ^{13}C) or Varian MERCURY plus-500 (500 MHz, ^{1}H; 125 MHz, ^{13}C) spectrometer with chemical shifts reported in ppm relative to the residual deuterated solvents. Mass spectrometry analysis was carried out using an electrospray spectrometer Waters Micromass Q-TOF Premier Mass Spectrometer. Melting points were measured with SGW X-4 micro melting point apparatus. Optical rotations were measured on a Rudolph Research Analytical Autopol VI automatic polarimeter using a 50 mm path-length cell at 589 nm. Chiral analyses were performed on a Shimadzu LC-2010 HPLC system, using an Enantiocol Chiral AD, AY, OD or OX-3 column (Guangzhou Research & Creativity Biotechnology Co., Ltd.) or Daicel Chiral AD-H, IE-H, OD-H, AS-H columns with *n*-hexane / *i*-propyl alcohol as an eluent.

Supplementary Note 1
Preparation of substrates

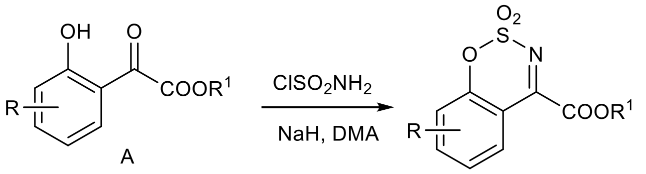

Supplementary Figure 1: Synthesis of six-membered ester imines

To a solution of **A** (2.78 mmol) in 5.0 mL of DMA was quickly transferred solid H_2NSO_2Cl (1.12 g, 9.71 mmol, 3.5 equiv) and stirred for 1 h. NaH (60% in mineral oil, 388 mg, 9.71 mmol, 3.5 equiv) was added over 3 potions in 2 h and stirred for another 2 h at room temperature. After stirring at 50 ^{o}C for 12 h, the reaction was quenched by the addition of 5 mL of H_2O and transferred to a separatory funnel with 20 mL of Et_2O. The organic layer was separated, and the aqueous layer was extracted with 2 x15 mL of Et_2O. The combined organic layers were dried over Na_2SO_4 and concentrated under reduced pressure. Purification by chromatography on silica gel (EtOAc/petroleum ether = 1:4) afforded the desired product.

Methyl 6,7-dimethylbenzo[*e*][1,2,3]oxathiazine-4-carboxylate 2,2-dioxide (1d)

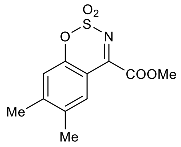

Light yellow solid, 441 mg, yield: 59%, Mp: 119-120 °C. ^{1}H NMR (400 MHz, $CDCl_3$): δ 7.68 (s, 1H), 7.09 (s, 1H), 4.04 (s, 3H), 2.38 (s, 3H), 2.30 (s, 3H); ^{13}C NMR (100 MHz, $CDCl_3$): δ 165.1, 161.7, 153.3, 150.5, 135.8, 130.2, 119.8, 111.5, 54.3, 21.2, 19.5. FMS (ESI) calcd for $C_{11}H_{12}NO_5S$ $(M+H)^+$ 270.043170, found 270.043070.

Methyl 7-fluorobenzo[*e*][1,2,3]oxathiazine-4-carboxylate 2,2-dioxide (1f)

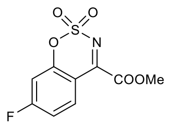

Light yellow solid, 439 mg, yield: 61%, Mp: 92-93 °C. ^{1}H NMR (400 MHz, $CDCl_3$): δ 8.14 (dd, *J* = 8.8, 5.6 Hz, 1H), 7.17-7.10 (m, 1H), 7.06 (dd, *J* = 8.0, 2.4 Hz, 1H), 4.05 (s, 3H); ^{13}C NMR (100 MHz, $CDCl_3$): δ 169.3, 166.6, 162.6 (d, *J* = 259.6 Hz), 156.9 (d, *J* = 13.4 Hz), 133.4 (d, *J* = 11.4 Hz), 114.8 (d, *J* = 22.5 Hz), 110.8 (d, *J* = 3.1 Hz), 107.5 (d, *J* = 26.1 Hz), 54.5. HRMS (ESI) calcd for $C_9H_7FNO_5S$ $(M+H)^+$ 260.0029, found 260.0031.

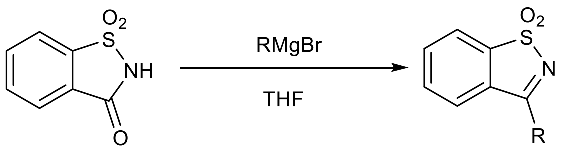

Supplementary Figure 2: Synthesis of alkyl substituted five-membered ketimines

Saccharin (1.83 g, 10.0 mmol, 1.0 equiv) and anhydrous THF (20 mL) were added to a flame-dried flask and cooled to 0 °C. Grignard reagent RMgBr (3.0 equiv, 30 mmol, in 30 mL THF, prepared from RBr and magnesium powder) was added dropwise to the mixture over 20 min. The reaction mixture was stirred at room temperature for 24 h, then acidified with 1 N HCl to pH 2 and diluted with water (30 mL). The mixture was extracted with EtOAc (3 × 50 mL). The combined organic layers were dried over Na_2SO_4, filtered, and concentrated under reduced pressure. Purification by column chromatography on silica gel (petroleum ether/EtOAc = 6/1) gave the desired product.

3-Propylbenzo[*d*]isothiazole 1,1-dioxide (1k)

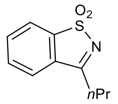

White solid, 1.67 g, yield: 80%, Mp: 118-119 °C. ^{1}H NMR (400 MHz, $CDCl_3$): δ 7.89-7.79 (m, 1H), 7.75-7.67 (m, 3H), 2.93 (t, *J* = 7.2 Hz, 2H), 1.93-1.83 (m, 2H), 1.06 (t, *J* = 7.6 Hz, 3H); ^{13}C NMR (100 MHz, $CDCl_3$): δ 176.7, 139.7, 134.3, 133.8, 131.5, 124.4, 122.5, 33.2, 19.1, 14.0. HRMS (ESI) calcd for $C_{10}H_{12}NO_2S$ $(M+H)^+$ 210.0589, found 210.0588.

3-Isopentylbenzo[*d*]isothiazole 1,1-dioxide (1p)

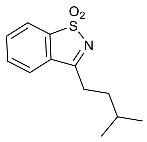

White solid, 1.47 g, yield: 62%, Mp: 61-62 °C. ^{1}H NMR (400 MHz, $CDCl_3$): δ 7.89-7.79 (m, 1H), 7.76-7.66 (m, 3H), 2.99-2.90 (m, 2H), 1.78-1.67 (m, 3H), 0.96 (d, *J* = 6.4 Hz, 6H); ^{13}C NMR (100 MHz, $CDCl_3$): δ 177.1, 139.8, 134.3, 133.8, 131.4, 124.3, 122.5, 34.4, 29.4, 28.0, 22.5. FMS (ESI) calcd for $C_{12}H_{16}NO_2S$ $(M+H)^+$ 238.089646, found 238.089626.

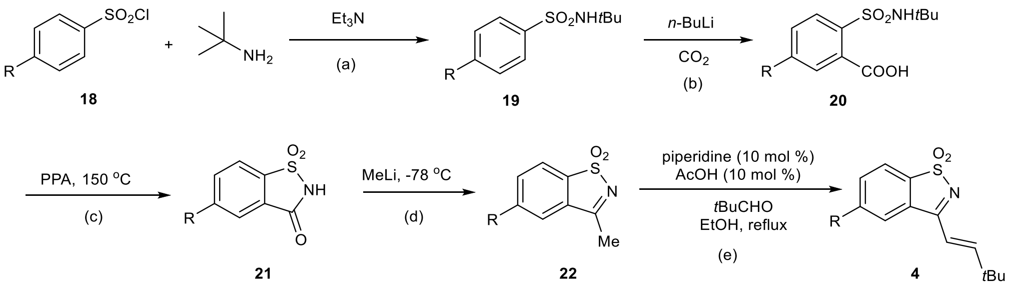

Supplementary Figure 3: Synthesis of alkenyl substituted five-membered ketimines(1)

A modified procedure was followed according to the literature.[1] (a) To a solution of *tert*-butylamine (11.7 g, 0.16 mol) and triethylamine (20.2 g, 0.2 mol) in dichloromethane was added arylsulfonyl chloride **18** (0.1 mol) dropwise in an ice bath. The mixture was stirred at room temperature overnight and was then washed with saturated sodium carbonate and brine. The organic layer was separated, and the aqueous layer was extracted with dichloromethane (3 ×100 mL). The combined organic phases were dried over anhydrous Na_2SO_4. The solvent was evaporated in vacuo to give the aryl sulfonamide **19** as a white solid without further purification. (b) *n*-BuLi (40 mmol) was added dropwise over a period of 20 minute to a cold (0 °C), stirred solution of the aryl sulfonamide **19** (20 mmol) in anhydrous tetrahydrofuran (50 ml) under a dry nitrogen atmosphere. After 10 min at 0 °C, the reaction was stirred for 2 h at room temperature. Then the solution was poured into a 200 mL beaker which contained several bars of dry ice and left overnight. 50 mL of water and

12 N HCl (4 mL) were added to the solution. The solvent was evaporated in vacuo and the aqueous layer was extracted with dichloromethane (3 × 100 mL). The combined organic phases were dried over anhydrous Na_2SO_4. The solvent was evaporated in vacuo to give the aryl sulfonamide **20** as a yellow solid without further purification. (c) A yellow suspension of the above products **20** in polyphosphoric acid (100 mL) was heated (150 °C) for 15 min while mixing manually with a spatula. The thick syrup was poured (hot) in a thin stream onto an excess of crushed ice which was vigorously stirred. Filtration of the solid and a thorough wash with water gave the product **21** as a gray solid without further purification. (d) To a 250 mL Schlenck flask equipped with a condenser, septum and magnetic stirring bar was added a solution of saccharin **21** (11 mmol) in THF (120 mL). The flask was cooled to –78 °C and methyl lithium (22 mmol) was carefully added by syringe. The reaction was stirred at –78 °C overnight. H_2O (100 mL) and NH_4Cl (2 g) was then added, and the reaction mixture was warmed to room temperature. Removal of the solvent in vacuo, filtration of the solid and a thorough wash with water gave the product **22** as a white solid without further purification. (e) Piperidine (2 drops) and acetic acid (2 drops) were added to a solution of cyclic *N*-sulfonylimines **22** (2 mmol) and *t*-BuCHO (4.4 mmol) in EtOH and the mixture was heated at reflux overnight. The mixture was cooled to 0 °C and the solvent was evaporated. Then H_2O (20 ml) and EtOAc (40 ml) was added and extracted. The solvent was evaporated and purified by column chromatography (PE:EA = 7:1) to afford the desired products.

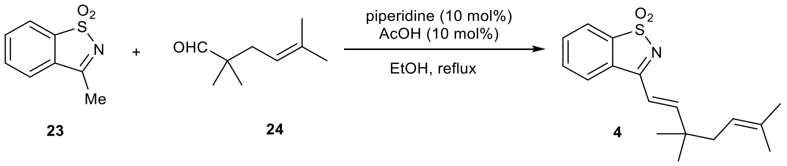

Supplementary Figure 4: Synthesis of alkenyl substituted five-membered ketimines(2)

Piperidine (5 drops) and acetic acid (5 drops) were added to a solution of cyclic *N*-sulfonylimines **23** (8 mmol) and aldehyde **24** (17.6 mmol) in EtOH and the mixture was heated at reflux overnight. The mixture was cooled to 0 °C and the solvent was evaporated. Then H_2O (40 ml) and EtOAc (80 ml) was added and extracted. The solvent was evaporated and purified by column chromatography (PE:EA = 7:1) to afford the desired product.

(*E*)-3-(3,3-Dimethylbut-1-en-1-yl)-5-methylbenzo[*d*]isothiazole 1,1-dioxide (4b)

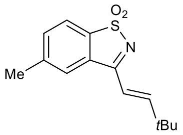

White solid, 373 mg, yield: 71%. Mp: 206-207 °C. ^{1}H NMR (400 MHz, $CDCl_3$): δ 7.79 (d, *J* = 7.6 Hz, 1H), 7.61 (d, *J* = 15.6 Hz, 1H), 7.59-7.50 (m, 2H), 6.59 (d, *J* = 15.6 Hz, 1H), 2.54 (s, 3H), 1.22 (s, 9H); ^{13}C NMR (100 MHz, $CDCl_3$): δ 167.9, 163.3, 145.1, 138.0, 134.3, 132.2, 124.7, 122.6, 113.0, 35.5, 28.8, 22.0. HRMS (ESI) calcd for $C_{14}H_{18}NO_2S$ $(M+H)^+$ 264.1058, found 264.1078.

(*E*)-3-(3,3-Dimethylbut-1-en-1-yl)-5-ethylbenzo[*d*]isothiazole 1,1-dioxide (4c)

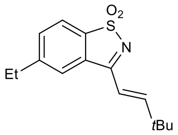

White solid, 438 mg, yield: 79%. Mp: 191-192 °C. ^{1}H NMR (400 MHz, $CDCl_3$): δ 7.83 (d, *J* = 8.4 Hz, 1H), 7.62 (d, *J* = 15.6 Hz, 1H), 7.59-7.52 (m, 2H), 6.59 (d, *J* = 15.6 Hz, 1H), 2.83 (q, *J* = 7.6 Hz, 2H), 1.32 (t, *J* = 7.6 Hz, 3H), 1.22 (s, 9H); ^{13}C NMR (100 MHz, $CDCl_3$): δ 167.7, 163.1, 151.1, 138.0, 133.0, 132.1, 123.3, 122.6, 112.6, 35.2, 29.1, 28.6, 15.5. HRMS (ESI) calcd for $C_{15}H_{20}NO_2S$ $(M+H)^+$ 278.1215, found 278.1235.

(*E*)-3-(3,3-Dimethylbut-1-en-1-yl)-5-phenylbenzo[*d*]isothiazole 1,1-dioxide (4d)

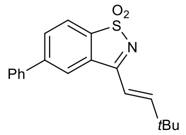

White solid, 436 mg, yield: 67%. Mp: 187-188 °C. ^1H NMR (400 MHz, $CDCl_3$): δ 8.00-7.89 (m, 3H), 7.67 (d, *J* = 15.6 Hz, 1H), 7.65-7.61 (m, 2H), 7.58-7.46 (m, 3H), 6.68 (d, *J* = 15.2 Hz, 1H), 1.24 (s, 9H); ^{13}C NMR (100 MHz, $CDCl_3$): δ 167.7, 163.8, 147.6, 139.1, 138.8, 132.7, 132.5, 129.6, 129.4, 127.7, 123.2, 122.9, 112.9, 35.5, 28.8. HRMS (ESI) calcd for $C_{19}H_{20}NO_2S$ $(M+H)^+$ 326.1215, found 326.1234.

(*E*)-3-(3,3,6-Trimethylhepta-1,5-dien-1-yl)benzo[*d*]isothiazole 1,1-dioxide (4e)

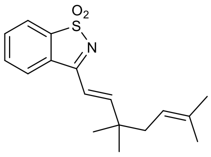

White solid, 2.06 g, yield: 85%. Mp: 100-101 °C. ^1H NMR (400 MHz, $CDCl_3$): δ 7.96-7.88 (m, 1H), 7.81-7.68 (m, 3H), 7.60 (d, *J* = 15.6 Hz, 1H), 6.57 (d, *J* = 15.6 Hz, 1H), 5.14-5.06 (m, 1H), 2.15 (d, *J* = 7.6 Hz, 2H), 1.71 (s, 3H), 1.61 (s, 3H), 1.17 (s, 6H); ^{13}C NMR (100 MHz, $CDCl_3$): δ 167.7, 163.0, 140.7, 134.9, 133.9, 133.8, 131.7, 124.3, 122.8, 119.7, 114.0, 40.5, 39.4, 26.3, 26.2, 18.2. HRMS (ESI) calcd for $C_{17}H_{22}NO_2S$ $(M+H)^+$ 304.1371, found 304.1378.

Other substrates were synthesized according to the literature.[1-12]

Preparation of chiral BOX ligands

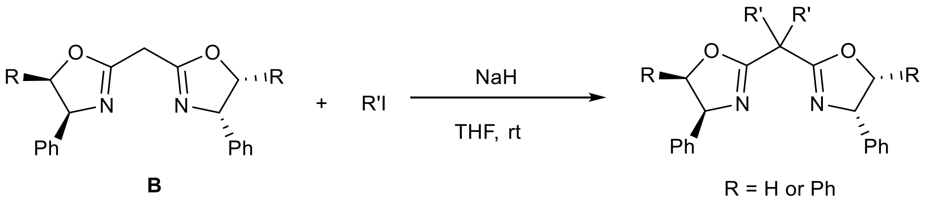

Supplementary Figure 5: Synthesis of bisoxazoline ligands

A modified procedure of Tang's literature[13] to synthesise chiral ligands was used as follows: NaH [60 % dispersion in mineral oil] (245 mg, 9.63 mmol) was added to a solution of B (1.75 mmol) in anhydrous THF (25 mL) at room temperature under N_2 atmosphere, and the resulting mixture was stirred for 30 min. R'I (5.25 mmol) was then added dropwise to the mixture, and the reaction was stirred at room temperature for 12 h. The mixture was quenched with water, extracted with EtOAc, and the organic layer was dried over anhydrous Na_2SO_4, and concentrated under reduced pressure. The product was purified by column chromatography using *n*-hexane/ethyl acetate (6:1) as eluent to give the desired products.

(4*S*,4'*S*)-2,2'-(Pentane-3,3-diyl)bis(4-phenyl-4,5-dihydrooxazole) (L1a)

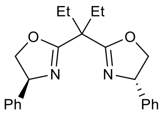

Colorless oil, 424 mg, yield: 67%. ^1H NMR (400 MHz, $CDCl_3$): δ 7.36-7.23 (m, 10H), 5.25 (dd, *J* = 10.0, 8.0 Hz, 2H), 4.66 (dd, *J* = 10.0, 8.4 Hz, 2H), 4.12 (t, *J* =8.0 Hz, 2H), 2.22-2.09 (m, 4H), 0.96 (t, *J* = 7.2 Hz, 6H); ^{13}C NMR (100 MHz, $CDCl_3$): δ 169.1, 142.6, 128.9, 127.7, 127.0, 75.2, 69.8, 47.2, 25.8, 8.8. FMS (ESI) calcd for $C_{23}H_{27}N_2O_2$ $(M+H)^+$ 363.206606, found 363.206705.

(4*S*,4'*S*)-2,2'-(Heptane-4,4-diyl)bis(4-phenyl-4,5-dihydrooxazole) (L1c)

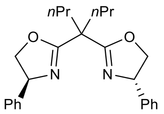

Colorless oil, 423 mg, yield: 62%. ^1H NMR (400 MHz, $CDCl_3$): δ 7.37-7.24 (m, 10H), 5.24 (dd, *J* = 10.0, 8.0 Hz, 2H), 4.66 (dd, *J* = 10.0, 8.4 Hz, 2H), 4.12 (t, *J* = 8.0 Hz, 2H), 2.17-2.03 (m, 4H), 1.40-1.33 (m, 4H), 0.98 (t, *J* = 7.2 Hz, 6H); ^{13}C NMR (100 MHz, $CDCl_3$): δ 169.4, 142.6, 128.9, 127.8, 127.0,

75.2, 69.8, 46.5, 35.3, 17.7, 14.7. HRMS (ESI) calcd for $C_{25}H_{31}N_2O_2$ $(M+H)^+$ 391.2386, found 391.2388.

(4*S*,4'*S*,5*R*,5'*R*)-2,2'-(Pentane-3,3-diyl)bis(4,5-diphenyl-4,5-dihydrooxazole) (L13)

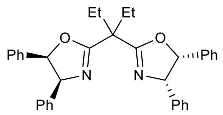

White solid, 603 mg, yield: 67%, Mp: 86-87 °C. ^{1}H NMR (400 MHz, $CDCl_3$): δ 7.10-6.85 (m, 20H), 5.96 (d, *J* = 10.0 Hz, 2H), 5.61 (d, *J* = 10.4 Hz, 2H), 2.55-2.43 (m, 2H), 2.39-2.28 (m, 2H) 1.16 (t, *J* = 7.6 Hz, 6H); ^{13}C NMR (100 MHz, $CDCl_3$): δ 169.2, 137.7, 136.3, 128.1, 127.8, 127.8, 127.6, 127.1, 126.9, 86.3, 74.0, 47.8, 26.1, 9.0. HRMS (ESI) calcd for $C_{35}H_{35}N_2O_2$ $(M+H)^+$ 515.2699, found 515.2698.

(4*S*,4'*S*,5*R*,5'*R*)-2,2'-(Heptane-4,4-diyl)bis(4,5-diphenyl-4,5-dihydrooxazole) (L2)

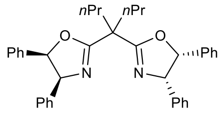

White solid, 550 mg, yield: 58%, Mp: 181-182 °C. ^{1}H NMR (400 MHz, $CDCl_3$): δ 7.10-6.85 (m, 20H), 5.95 (d, *J* = 10.0 Hz, 2H), 5.59 (d, *J* = 10.0 Hz, 2H), 2.48-2.37 (m, 2H), 2.32-2.22 (m, 2H), 1.65-1.50 (m, 4H), 1.09 (t, *J* = 7.2 Hz, 6H); ^{13}C NMR (100 MHz, $CDCl_3$): δ 169.4, 137.7, 136.3, 128.1, 127.8, 127.8, 127.6, 127.2, 126.9, 86.4, 73.9, 47.1, 35.6, 17.9, 14.8. FMS (ESI) calcd for $C_{37}H_{39}N_2O_2$ $(M+H)^+$ 543.300525, found 543.300605.

Condition screening for asymmetric alkenylation of ketimines

Supplementary Table 1.[a,b,c]

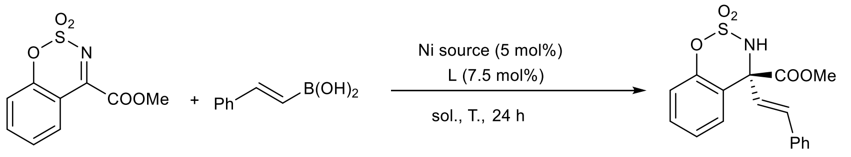

1a **2a** **3aa**

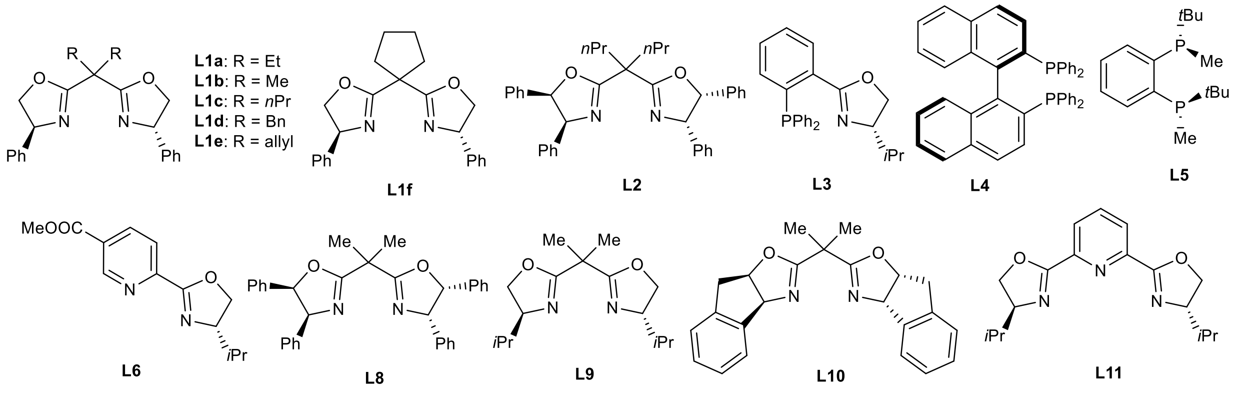

Entry	Ni source	Ligand	Solvent	T/°C	Yield [%]	ee [%]
1	$Ni(ClO_4)_2{\cdot}6H_2O$	**L1a**	DCE	80	trace	---
2	$Ni(ClO_4)_2{\cdot}6H_2O$	**L1a**	Toluene	80	48	72
3	$Ni(ClO_4)_2{\cdot}6H_2O$	**L1a**	*i*PrOH	80	32	93
4	$Ni(ClO_4)_2{\cdot}6H_2O$	**L1a**	dioxane	80	54	96
5	$Ni(ClO_4)_2{\cdot}6H_2O$	**L1a**	MeCN	80	trace	---
**6**	**$Ni(ClO_4)_2{\cdot}6H_2O$**	**L1a**	**TFE**	**reflux**	**99**	**78**
7	$Ni(ClO_4)_2{\cdot}6H_2O$	**L1a**	TFE	60	99	73

8	$Ni(ClO_4)_2\cdot 6H_2O$	**L1a**	TFE	50	99	64
9	$Ni(ClO_4)_2\cdot 6H_2O$	**L1a**	TFE	40	99	60
10[d]	$Ni(ClO_4)_2\cdot 6H_2O$	**L1a**	TFE	100	87	78
11[d]	$Ni(ClO_4)_2\cdot 6H_2O$	**L1a**	TFE	120	90	83
12	$Ni(ClO_4)_2\cdot 6H_2O$	**L1b**	TFE	reflux	99	30
13	$Ni(ClO_4)_2\cdot 6H_2O$	**L1c**	TFE	reflux	99	90
14	$Ni(ClO_4)_2\cdot 6H_2O$	**L1d**	TFE	reflux	91	78
15	$Ni(ClO_4)_2\cdot 6H_2O$	**L1e**	TFE	reflux	96	70
16	$Ni(ClO_4)_2\cdot 6H_2O$	**L1f**	TFE	reflux	96	14
**17**	**$Ni(ClO_4)_2\cdot 6H_2O$**	**L2**	**TFE**	**reflux**	**99**	**92**
18	$Ni(ClO_4)_2\cdot 6H_2O$	**L3**	TFE	reflux	87	90
19	$Ni(ClO_4)_2\cdot 6H_2O$	**L4**	TFE	reflux	trace	---
20	$Ni(ClO_4)_2\cdot 6H_2O$	**L5**	TFE	reflux	trace	---
21	$Ni(ClO_4)_2\cdot 6H_2O$	**L6**	TFE	reflux	trace	---
22	$Ni(ClO_4)_2\cdot 6H_2O$	**L8**	TFE	reflux	99	57
23	$Ni(ClO_4)_2\cdot 6H_2O$	**L9**	TFE	reflux	50	29
24	$Ni(ClO_4)_2\cdot 6H_2O$	**L10**	TFE	reflux	38	61
25	$Ni(ClO_4)_2\cdot 6H_2O$	**L11**	TFE	reflux	NR	---
26	$NiCl_2\cdot 6H_2O$	**L2**	TFE	reflux	97	86
27	$NiSO_4\cdot 6H_2O$	**L2**	TFE	reflux	86	80
28	$Ni(OAc)_2\cdot 4H_2O$	**L2**	TFE	reflux	91	81
29	$NiBr_2$	**L2**	TFE	reflux	80	86
30	$NiCl_2\cdot DME$	**L2**	TFE	reflux	94	89
31	$Ni(acac)_2$	**L2**	TFE	reflux	83	80
**32**	**$Ni(OTf)_2$**	**L2**	**TFE**	**reflux**	**99**	**94**
33	**---**	**L2**	TFE	reflux	NR	---
34	$Ni(ClO_4)_2\cdot 6H_2O$	---	TFE	reflux	NR	---

[a] Reactions were carried out on a 0.20 mmol scale (**1a**) using *trans*-PhCH=CHB$(OH)_2$ (**2a**) (0.30 mmol), 5 mol% nickel salt, 7.5 mol% ligand in unpurified solvent (2.0 mL) in a test tube for 24 h which was opened to air. [b] Yield of isolated product. [c] Ees were determined by HPLC using a chiral column. [d] A sealed tube was used instead of a test tube. TFE = trifluoroethanol, DME = 1,2-dimethoxyethane, DCE = 1,2-dichloroethane, NR = no reaction.

Ni(II)/DiPh-BOX-catalyzed asymmetric alkenylation of ketimines

A test tube (100 mL, 25 * 250 mm) was charged with $Ni(OTf)_2$ (3.6 mg, 0.010 mmol, 0.050 equiv), **L2** (8.1 mg, 0.015 mmol, 0.075 equiv) and unpurified TFE (1.0 mL). The solution was stirred at reflux for 5 min, then substrate (0.20 mmol, 1.0 equiv) and alkenylboronic acid (0.30 mmol, 1.5 equiv) were added into the tube. The wall of the tube was rinsed with an additional portion of TFE (1.0 mL). After stirring at reflux for 24 h in air, the reaction mixture was cooled to room

temperature and the solvent was removed by rotary evaporation. The residue was purified by preparative TLC on silica gel (petroleum ether/EtOAc = 5/1) to give the product.
(Racemic ligand ***rac*-L2** was used for racemic products and the procedure is the same as above.)

(*R*,*E*)-Methyl 4-styryl-3,4-dihydrobenzo[*e*][1,2,3]oxathiazine-4-carboxylate 2,2-dioxide (3aa).

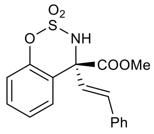

White solid, 68.6 mg, yield: 99%, Mp: 169-170 °C. ^{1}H NMR (400 MHz, $CDCl_3$) δ 7.75 (dd, *J* = 8.0, 1.6 Hz, 1H), 7.46-7.40 (m, 3H), 7.38-7.28 (m, 4H), 7.11 (dd, *J* = 8.0, 1.2 Hz, 1H), 6.85 (d, *J* = 15.6 Hz, 1H), 6.53 (d, *J* = 16.0 Hz, 1H), 6.34 (brs, 1H), 3.94 (s, 3H). ^{13}C NMR (100 MHz, $CDCl_3$) δ 169.9, 150.3, 135.3, 133.8, 130.7, 129.1, 128.7, 128.6, 127.1, 126.0, 125.6, 119.7, 119.4, 68.5, 54.7. HPLC [Daicel Chiralpak AD-H, hexane/*i*-PrOH = 90/10, 210 nm, 1.0 mL/min. t_{R1} = 21.2 min (major), t_{R2} = 25.3 min (minor)]; ee = 94%, $[\alpha]^{25}_D$ = +3.9 (c = 0.83, $CHCl_3$); HRMS (ESI) calcd for $C_{17}H_{16}NO_5S$ $(M+H)^+$ 346.0749, found 346.0743.

(*R*,*E*)-Methyl 8-methyl-4-styryl-3,4-dihydrobenzo[*e*][1,2,3]oxathiazine-4-carboxylate 2,2-dioxide (**3ba**).

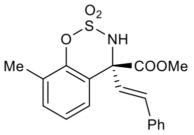

White ointment, 62.5 mg, yield: 87%. ^{1}H NMR (400 MHz, $CDCl_3$) δ 7.52 (dd, *J* = 8.0, 1.2 Hz, 1H), 7.42-7.37 (m, 2H), 7.35-7.26 (m, 3H), 7.26-7.23 (m, 1H), 7.16 (t, *J* = 7.6 Hz, 1H), 6.80 (d, *J* = 16.0 Hz, 1H), 6.50 (d, *J* = 16.0 Hz, 1H), 6.27 (brs, 1H), 3.90 (s, 3H), 2.30 (s, 3H). ^{13}C NMR (100 MHz, $CDCl_3$) δ 170.1, 148.8, 135.4, 133.6, 132.1, 129.1, 128.6, 128.6, 127.1, 126.4, 126.2, 124.9, 119.2, 68.6, 54.7, 15.7. HPLC [Daicel Chiralpak AD-H, hexane/*i*-PrOH = 98/2, 210 nm, 0.5 mL/min. t_{R1} = 89.6 min (major), t_{R2} = 94.2 min (minor)]; ee = 98%, $[\alpha]^{25}_D$ = +2.1 (c = 0.29, $CHCl_3$); HRMS (ESI) calcd for $C_{18}H_{17}NNaO_5S$ $(M+Na)^+$ 382.0725, found 382.0723.

(*R*,*E*)-Methyl 7-methyl-4-styryl-3,4-dihydrobenzo[*e*][1,2,3]oxathiazine-4-carboxylate 2,2-dioxide (3ca).

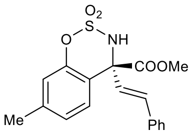

White ointment, 64.6 mg, yield: 90%. ^{1}H NMR (400 MHz, $CDCl_3$) δ 7.57 (d, *J* = 8.0 Hz, 1H), 7.42-7.36 (m, 2H), 7.35-7.26 (m, 3H), 7.11-7.05 (m, 1H), 6.91-6.87 (m, 1H), 6.80 (d, *J* = 15.6 Hz, 1H), 6.48 (d, *J* = 15.6 Hz, 1H), 6.24 (brs, 1H), 3.89 (s, 3H), 2.37 (s, 3H). ^{13}C NMR (100 MHz, $CDCl_3$) δ 170.3, 141.7, 135.7, 133.8, 128.9, 128.9, 128.8, 127.8, 127.3, 126.8, 126.4, 120.1, 116.5, 68.5, 54.8, 21.2. HPLC [Daicel Chiralpak AD-H, hexane/*i*-PrOH = 95/5, 210 nm, 1.0 mL/min. t_{R1} = 56.5 min (major), t_{R2} = 68.7 min (minor)]; ee > 99%, $[\alpha]^{25}_D$ = +2.9 (c = 0.28, $CHCl_3$); HRMS (ESI) calcd for $C_{18}H_{18}NO_5S$ $(M+H)^+$ 360.0905, found 360.0894.

(*R*,*E*)-Methyl 6,7-dimethyl-4-styryl-3,4-dihydrobenzo[*e*][1,2,3]oxathiazine-4-carboxylate 2,2-dioxide (**3da**).

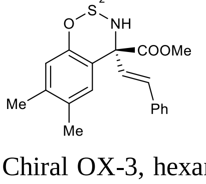

White ointment, 60.4 mg, yield: 81%. ^{1}H NMR (400 MHz, $CDCl_3$) δ 7.44-7.39 (m, 3H), 7.36-7.27 (m, 3H), 6.86 (s, 1H), 6.82 (d, *J* = 16.0 Hz, 1H), 6.50 (d, *J* = 16.0 Hz, 1H), 6.24 (brs, 1H), 3.90 (s, 3H), 2.28 (s, 3H), 2.26 (s, 3H). ^{13}C NMR (100 MHz, $CDCl_3$) δ 170.3, 148.3, 140.3, 135.7, 134.6, 133.7, 129.5, 128.9, 128.7, 127.3, 126.4, 120.4, 116.5, 68.5, 54.8, 19.8, 19.7. HPLC [Enantiocol Chiral OX-3, hexane/*i*-PrOH = 90/10, 210 nm, 1.0 mL/min. t_{R1} = 35.8 min (minor), t_{R2} = 47.7 min (major)]; ee = 96%, $[\alpha]^{25}_D$ = +29.7 (c = 0.87, $CHCl_3$); HRMS (ESI) calcd for $C_{19}H_{20}NO_5S$ $(M+H)^+$ 374.1062, found 374.1068.

(*R*,*E*)-Methyl 7-chloro-4-styryl-3,4-dihydrobenzo[*e*][1,2,3]oxathiazine-4-carboxylate 2,2-dioxide (3ea).

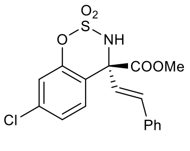

White ointment, 70.5 mg, yield: 93%. ^{1}H NMR (400 MHz, $CDCl_3$) δ 7.65 (d, *J* = 8.4 Hz, 1H), 7.43-7.37 (m, 2H), 7.36-7.24 (m, 4H), 7.10 (d, *J* = 2.4 Hz, 1H), 6.79 (d, *J* = 15.6 Hz, 1H), 6.45 (d, *J* = 15.6 Hz, 1H), 3.91 (s, 3H). ^{13}C NMR (100 MHz, $CDCl_3$) δ 169.8, 150.9, 136.4, 135.3, 134.4, 130.5,

129.0, 128.9, 127.3, 126.2, 125.8, 120.1, 118.2, 68.5, 55.0. HPLC [Enantiocol Chiral OX-3, hexane/*i*-PrOH = 95/5, 210 nm, 0.5 mL/min. t_{R1} = 60.7 min (minor), t_{R2} = 76.6 min (major)]; ee = 79%, $[\alpha]^{25}_{D}$ = +14.1 (c = 0.85, $CHCl_3$); HRMS (ESI) calcd for $C_{17}H_{15}ClNO_5S$ $(M+H)^+$ 380.0359, found 380.0358.

(*R*,*E*)-Methyl 7-fluoro-4-styryl-3,4-dihydrobenzo[*e*][1,2,3]oxathiazine-4-carboxylate 2,2-dioxide (**3fa**).

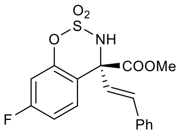

White ointment, 69.7 mg, yield: 96%. ^1H NMR (400 MHz, $CDCl_3$) δ 7.70 (dd, *J* = 9.2, 2.0 Hz, 1H), 7.43-7.37 (m, 2H), 7.37-7.24 (m, 3H), 7.05-6.98 (m, 1H), 6.83 (dd, *J* = 8.4, 2.4 Hz, 1H), 6.79 (d, *J* = 16.0 Hz, 1H), 6.45 (d, *J* = 16.0 Hz, 1H), 3.91 (s, 3H). ^{13}C NMR (100 MHz, $CDCl_3$) δ 169.7, 163.0 (d, *J* = 253.5 Hz), 151.2 (d, *J* = 11.9 Hz), 135.1, 134.0, 130.7 (d, *J* = 9.5 Hz), 128.8, 128.7, 127.1, 125.7, 115.4 (d, *J* = 3.9 Hz), 113.2 (d, *J* = 21.7 Hz), 107.3 (d, *J* = 25.6 Hz), 68.1, 54.8. HPLC [Enantiocol Chiral OX-3, hexane/*i*-PrOH = 90/10, 210 nm, 1.0 mL/min. t_{R1} = 17.3 min (minor), t_{R2} = 21.5 min (major)]; ee = 98%, $[\alpha]^{25}_{D}$ = +3.1 (c = 0.13, $CHCl_3$); HRMS (ESI) calcd for $C_{17}H_{15}FNO_5S$ $(M+H)^+$ 364.0655, found 364.0655.

(*R*,*E*)-Ethyl 4-styryl-3,4-dihydrobenzo[*e*][1,2,3]oxathiazine-4-carboxylate 2,2-dioxide (**3ga**).

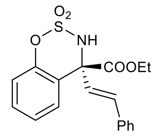

Colorless oil, 69.6 mg, yield: 97%. ^1H NMR (400 MHz, $CDCl_3$) δ 7.73 (dd, *J* = 8.0, 1.6 Hz, 1H), 7.43-7.37 (m, 3H), 7.35-7.27 (m, 4H), 7.09 (dd, *J* = 8.0, 1.2 Hz, 1H), 6.82 (d, *J* = 15.6 Hz, 1H), 6.50 (d, *J* = 15.6 Hz, 1H), 6.31 (brs, 1H), 4.36 (q, *J* = 7.2 Hz, 2H), 1.34 (t, *J* = 7.2 Hz, 3H). ^{13}C NMR (100 MHz, $CDCl_3$) δ 169.3, 150.4, 135.5, 133.6, 130.7, 129.0, 128.7, 128.6, 127.1, 126.1, 125.5, 119.7, 119.5, 68.4, 64.3, 14.0. HPLC [Daicel Chiralpak AD-H, hexane/*i*-PrOH = 90/10, 210 nm, 1.0 mL/min. t_{R1} = 18.0 min (major), t_{R2} = 20.1 min (minor)]; ee = 85%, $[\alpha]^{25}_{D}$ = +11.2 (c = 0.25, $CHCl_3$); HRMS (ESI) calcd for $C_{18}H_{18}NO_5S$ $(M+H)^+$ 360.0906, found 360.0898.

(*R*,*E*)-Propyl 4-styryl-3,4-dihydrobenzo[*e*][1,2,3]oxathiazine-4-carboxylate 2,2-dioxide (3ha).

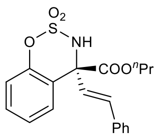

Colorless oil, 72.4 mg, yield: 97%. ^1H NMR (400 MHz, $CDCl_3$) δ 7.76 (dd, *J* = 8.0, 1.6 Hz, 1H), 7.45-7.37 (m, 3H), 7.37-7.26 (m, 4H), 7.09 (dd, *J* = 8.0, 1.2 Hz, 1H), 6.85 (d, *J* = 15.6 Hz, 1H), 6.53 (d, *J* = 16.0 Hz, 1H), 6.37 (brs, 1H), 4.32-4.22 (m, 2H), 1.80-1.69 (m, 2H), 0.96 (t, *J* = 7.6 Hz, 3H). ^{13}C NMR (100 MHz, $CDCl_3$) δ 169.7, 150.6, 135.7, 133.9, 130.9, 129.3, 128.9, 128.8, 127.3, 126.4, 125.7, 119.9, 119.8, 70.3, 68.7, 22.0, 10.5. HPLC [Daicel Chiralpak AD-H, hexane/*i*-PrOH = 90/10, 210 nm, 1.0 mL/min. t_{R1} = 16.6 min (major), t_{R2} = 18.3 min (minor)]; ee = 83%, $[\alpha]^{25}_{D}$ = +20.1 (c = 0.28, $CHCl_3$); HRMS (ESI) calcd for $C_{19}H_{20}NO_5S$ $(M+H)^+$ 374.1062, found 374.1065.

(*R*,*E*)-3-Methyl-3-styryl-2,3-dihydrobenzo[*d*]isothiazole 1,1-dioxide (3ia).

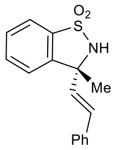

White ointment, 55.9 mg, yield: 98%. ^1H NMR (400 MHz, $CDCl_3$) δ 7.77 (d, *J* = 7.6 Hz, 1H), 7.63 (t, *J* = 8.0 Hz, 1H), 7.52 (t, *J* = 7.6 Hz, 1H), 7.42-7.33 (m, 3H), 7.33-7.23 (m, 3H), 6.75 (d, *J* = 16.0 Hz, 1H), 6.35 (d, *J* = 16.0 Hz, 1H), 5.04 (brs, 1H), 1.85 (s, 3H). ^{13}C NMR (100 MHz, $CDCl_3$) δ 144.0, 135.9, 134.9, 133.7, 131.4, 130.8, 129.7, 128.9, 128.6, 127.0, 124.2, 121.6, 64.0, 27.7. HPLC [Daicel Chiralpak IE-H, hexane/*i*-PrOH = 90/10, 210 nm, 1.5 mL/min. t_{R1} = 48.1 min (major), t_{R2} = 65.2 min (minor)]; ee = 99%, $[\alpha]^{25}_{D}$ = -9.8 (c = 0.61, $CHCl_3$); HRMS (ESI) calcd for $C_{16}H_{16}NO_2S$ $(M+H)^+$ 286.0902, found 286.0887.

(*R*,*E*)-3-Ethyl-3-styryl-2,3-dihydrobenzo[*d*]isothiazole 1,1-dioxide (**3ja**).

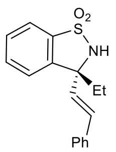

White ointment, 59.6 mg, yield: 99%. ^1H NMR (400 MHz, $CDCl_3$) δ 7.77 (d, *J* = 8.0 Hz, 1H), 7.64 (t, *J* = 7.6 Hz, 1H), 7.53 (t, *J* = 8.0 Hz, 1H), 7.39 (d, *J* = 7.6 Hz, 1H), 7.38-7.33 (m, 2H), 7.32-7.23 (m, 3H), 6.76 (d,

J = 16.0 Hz, 1H), 6.35 (d, *J* = 15.6 Hz, 1H), 4.89 (brs, 1H), 2.14 (q, *J* = 7.6 Hz, 2H), 0.93 (t, *J* = 7.6 Hz, 3H). ^{13}C NMR (100 MHz, $CDCl_3$) δ 142.5, 136.0, 135.1, 133.7, 130.9, 130.8, 129.7, 128.9, 128.4, 127.0, 124.1, 121.7, 67.8, 33.4, 8.4. HPLC [Daicel Chiralpak IE-H, hexane/*i*-PrOH = 90/10, 210 nm, 1.5 mL/min. t_{R1} = 45.0 min (minor), t_{R2} = 48.3 min (major)]; ee = 96%, $[\alpha]^{25}_{D}$ = -20.3 (c = 1.23, $CHCl_3$); HRMS (ESI) calcd for $C_{17}H_{18}NO_2S$ $(M+H)^+$ 300.1058, found 300.1060.

(*R*,*E*)-3-Propyl-3-styryl-2,3-dihydrobenzo[*d*]isothiazole 1,1-dioxide (3ka).

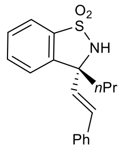

White ointment, 58.8 mg, yield: 94%. ^{1}H NMR (400 MHz, $CDCl_3$) δ 7.76 (d, *J* = 8.0 Hz, 1H), 7.63 (t, *J* = 8.0 Hz, 1H), 7.51 (t, *J* = 8.0 Hz, 1H), 7.41 (d, *J* = 8.0 Hz, 1H), 7.38-7.33 (m, 2H), 7.32-7.20 (m, 3H), 6.76 (d, *J* = 15.6 Hz, 1H), 6.35 (d, *J* = 15.6 Hz, 1H), 5.10 (brs, 1H), 2.12-2.01 (m, 2H), 1.57-1.50 (m, 1H), 1.20-1.11 (m, 1H), 0.92 (t, *J* = 7.6 Hz, 3H). ^{13}C NMR (100 MHz, $CDCl_3$) δ 142.8, 136.1, 134.9, 133.7, 131.1, 130.6, 129.6, 128.9, 128.4, 127.0, 124.1, 121.7, 67.4, 42.8, 17.5, 14.2. HPLC [Daicel Chiralpak IE-H, hexane/*i*-PrOH = 90/10, 210 nm, 1.0 mL/min. t_{R1} = 49.1 min (minor), t_{R2} = 51.8 min (major)]; ee = 97%, $[\alpha]^{25}_{D}$ = -29.2 (c = 0.91, $CHCl_3$); HRMS (ESI) calcd for $C_{18}H_{20}NO_2S$ $(M+H)^+$ 314.1215, found 314.1214.

(*R*,*E*)-3-Butyl-3-styryl-2,3-dihydrobenzo[*d*]isothiazole 1,1-dioxide (**3la**).

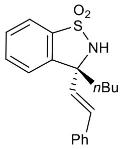

White ointment, 62.1 mg, yield: 95%. ^{1}H NMR (400 MHz, $CDCl_3$) δ 7.76 (d, *J* = 7.6 Hz, 1H), 7.63 (t, *J* = 8.0 Hz, 1H), 7.51 (t, *J* = 8.0 Hz, 1H), 7.44-7.32 (m, 3H), 7.32-7.20 (m, 3H), 6.77 (d, *J* = 16.0 Hz, 1H), 6.36 (d, *J* = 16.0 Hz, 1H), 5.13 (brs, 1H), 2.15-1.99 (m, 2H), 1.53-1.44 (m, 1H), 1.36-1.27 (m, 2H), 1.16-1.06 (m, 1H), 0.88 (t, *J* = 7.6 Hz, 3H). ^{13}C NMR (100 MHz, $CDCl_3$) δ 142.8, 136.1, 135.0, 133.6, 131.1, 130.6, 129.6, 128.8, 128.4, 127.0, 124.1, 121.7, 67.4, 40.4, 26.1, 22.9, 14.1. HPLC [Daicel Chiralpak IE-H, hexane/*i*-PrOH = 95/5, 210 nm, 0.4 mL/min. t_{R1} = 203.1 min (minor), t_{R2} = 209.9 min (major)]; ee = 98%, $[\alpha]^{25}_{D}$ = -30.3 (c = 1.18, $CHCl_3$); HRMS (ESI) calcd for $C_{19}H_{22}NO_2S$ $(M+H)^+$ 328.1371, found 328.1378.

(*R*,*E*)-3-Pentyl-3-styryl-2,3-dihydrobenzo[*d*]isothiazole 1,1-dioxide (3ma).

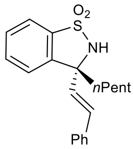

White ointment, 52.4 mg, yield: 97%. ^{1}H NMR (400 MHz, $CDCl_3$) δ 7.80 (d, *J* = 8.0 Hz, 1H), 7.67 (t, *J* = 8.0 Hz, 1H), 7.56 (t, *J* = 7.6 Hz, 1H), 7.43 (d, *J* = 8.0 Hz, 1H), 7.41-7.36 (m, 2H), 7.36-7.27 (m, 3H), 6.78 (d, *J* = 15.6 Hz, 1H), 6.37 (d, *J* = 16.0 Hz, 1H), 4.87 (brs, 1H), 2.14-2.06 (m, 2H), 1.56-1.48 (m, 1H), 1.34-1.28 (m, 4H), 1.20-1.11 (m, 1H), 0.88 (t, *J* = 7.2 Hz, 3H). ^{13}C NMR (100 MHz, $CDCl_3$) δ 142.8, 136.1, 134.9, 133.6, 131.0, 130.7, 129.6, 128.8, 128.4, 127.0, 124.0, 121.7, 67.4, 40.6, 31.9, 23.7, 22.6, 14.2. HPLC [Daicel Chiralpak IE-H, hexane/*i*-PrOH = 95/5, 210 nm, 0.4 mL/min. t_{R1} = 181.2 min (minor), t_{R2} = 188.0 min (major)]; ee = 96%, $[\alpha]^{25}_{D}$ = -27.5 (c = 1.09, $CHCl_3$); HRMS (ESI) calcd for $C_{20}H_{24}NO_2S$ $(M+H)^+$ 342.1528, found 342.1523.

(*R*,*E*)-3-(3-(Benzyloxy)propyl)-3-styryl-2,3-dihydrobenzo[*d*]isothiazole 1,1-dioxide (**3na**).

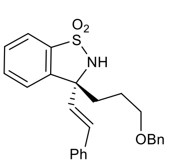

White ointment, 72.9 mg, yield: 87%. ^{1}H NMR (400 MHz, $CDCl_3$) δ 7.77 (d, *J* = 8.0 Hz, 1H), 7.62 (td, *J* = 7.6, 1.2 Hz, 1H), 7.52 (t, *J* = 7.6 Hz, 1H), 7.40 (d, *J* = 7.6 Hz, 1H), 7.37-7.33 (m, 5H), 7.33-7.17 (m, 5H), 6.80 (d, *J* = 16.0 Hz, 1H), 6.31 (d, *J* = 16.0 Hz, 1H), 5.86 (brs, 1H), 4.55 (d, *J* = 12.0 Hz, 1H), 4.50 (d, *J* = 12.0 Hz, 1H), 3.50-3.43 (m, 2H), 2.29-2.19 (m, 2H), 1.76-1.68 (m, 1H), 1.61-1.55 (m, 1H). ^{13}C NMR (100 MHz, $CDCl_3$) δ 142.8, 137.7, 135.9, 135.2, 133.3, 130.7, 130.5, 129.4, 128.6, 128.5, 128.1, 128.0, 127.9, 126.8, 123.7, 121.6, 73.2, 69.4, 66.8, 37.3, 24.1. HPLC [Enantiocol Chiral AD, hexane/*i*-PrOH = 90/10, 210 nm, 1.5 mL/min. t_{R1} = 44.3 min (major), t_{R2} = 60.5 min (minor)]; ee = 84%, $[\alpha]^{25}_{D}$ = -17.8 (c = 0.27, $CHCl_3$); HRMS (ESI) calcd for $C_{25}H_{26}NO_3S$ $(M+H)^+$ 420.1633, found 420.1643.

(*R*,*E*)-3-(3-Phenylpropyl)-3-styryl-2,3-dihydrobenzo[*d*]isothiazole 1,1-dioxide (3oa).

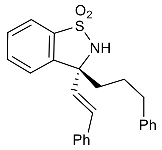

White ointment, 73.1 mg, yield: 94%. ^{1}H NMR (400 MHz, $CDCl_3$) δ 7.77 (d, *J* = 8.0 Hz, 1H), 7.62 (t, *J* = 8.0 Hz, 1H), 7.53 (t, *J* = 8.0 Hz, 1H), 7.36-7.22 (m, 8H), 7.22-7.16 (m, 1H), 7.12 (d, *J* = 8.0 Hz, 2H), 6.68 (d, *J* = 15.6 Hz, 1H), 6.29 (d, *J* = 15.6 Hz, 1H), 4.75 (brs, 1H), 2.63 (t, *J* = 7.2 Hz, 2H), 2.14-2.06 (m, 2H), 1.92-1.78 (m, 1H), 1.54-1.40 (m, 1H). ^{13}C NMR (100 MHz, $CDCl_3$) δ 142.5, 141.5, 135.9, 135.0, 133.7, 130.9, 130.8, 129.7, 128.9, 128.7, 128.5, 127.0, 126.3, 124.0, 121.8, 67.2, 39.7, 35.6, 25.6. HPLC [Enantiocol Chiral AD, hexane/*i*-PrOH = 80/20, 210 nm, 1.0 mL/min. t_{R1} = 19.8 min (major), t_{R2} = 21.7 min (minor)]; ee = 97%, $[\alpha]^{25}_D$ = -24.0 (c = 0.64, $CHCl_3$); HRMS (ESI) calcd for $C_{24}H_{24}NO_2S$ $(M+H)^+$ 390.1528, found 390.1520.

(*R*,*E*)-3-Isopentyl-3-styryl-2,3-dihydrobenzo[*d*]isothiazole 1,1-dioxide (**3pa**).

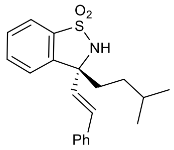

White ointment, 66.8 mg, yield: 98%. ^{1}H NMR (400 MHz, $CDCl_3$) δ 7.77 (d, *J* = 8.0 Hz, 1H), 7.65 (t, *J* = 7.6 Hz, 1H), 7.53 (t, *J* = 7.6 Hz, 1H), 7.41 (d, *J* = 8.0 Hz, 1H), 7.38-7.33 (m, 2H), 7.33-7.21 (m, 3H), 6.75 (d, *J* = 16.0 Hz, 1H), 6.34 (d, *J* = 16.0 Hz, 1H), 4.81 (brs, 1H), 2.12-2.02 (m, 2H), 1.60-1.49 (m, 1H), 1.42-1.33 (m, 1H), 1.06-0.96 (m, 1H), 0.88 (t, *J* = 6.8 Hz, 6H). ^{13}C NMR (100 MHz, $CDCl_3$) δ 142.8, 136.0, 135.0, 133.7, 131.0, 130.7, 129.6, 128.9, 128.4, 127.0, 124.0, 121.8, 67.3, 38.5, 32.7, 28.3, 22.7. HPLC [Enantiocol Chiral AD, hexane/*i*-PrOH = 90/10, 210 nm, 1.0 mL/min. t_{R1} = 42.9 min (minor), t_{R2} = 57.1 min (major)]; ee = 97%, $[\alpha]^{25}_D$ = -19.8 (c = 1.04, $CHCl_3$); HRMS (ESI) calcd for $C_{20}H_{24}NO_2S$ $(M+H)^+$ 342.1528, found 342.1523.

(*R*,*E*)-3-Isopropyl-3-styryl-2,3-dihydrobenzo[*d*]isothiazole 1,1-dioxide (3qa).

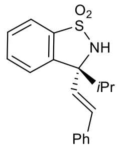

White ointment, 55.1 mg, yield: 86%. ^{1}H NMR (400 MHz, $CDCl_3$) δ 7.76 (d, *J* = 8.0 Hz, 1H), 7.64 (t, *J* = 7.6 Hz, 1H), 7.52 (t, *J* = 7.6 Hz, 1H), 7.41 (d, *J* = 8.0 Hz, 1H), 7.38-7.32 (m, 2H), 7.33-7.26 (m, 2H), 7.25-7.22 (m, 1H), 6.81 (d, *J* = 15.6 Hz, 1H), 6.37 (d, *J* = 15.6 Hz, 1H), 4.65 (brs, 1H), 2.49-2.38 (m, 1H), 1.09 (d, *J* = 7.2 Hz, 3H), 0.79 (d, *J* = 6.8 Hz, 3H). ^{13}C NMR (100 MHz, $CDCl_3$) δ 142.5, 136.1, 135.2, 133.7, 131.1, 130.7, 129.6, 128.8, 128.3, 126.9, 123.7, 121.9, 70.9, 37.1, 17.7, 16.8. HPLC [Enantiocol Chiral AD, hexane/*i*-PrOH = 90/10, 210 nm, 1.0 mL/min. t_{R1} = 43.6 min (major), t_{R2} = 74.1 min (minor)]; ee = 88%, $[\alpha]^{25}_D$ = -73.1 (c = 0.50, $CHCl_3$); HRMS (ESI) calcd for $C_{18}H_{20}NO_2S$ $(M+H)^+$ 314.1215, found 314.1217.

(*R*,*E*)-3-Cyclopropyl-3-styryl-2,3-dihydrobenzo[*d*]isothiazole 1,1-dioxide (**3ra**).

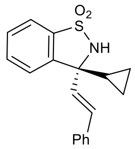

White solid, 55.4 mg, yield: 89%, Mp: 53-54°C. ^{1}H NMR (400 MHz, $CDCl_3$) δ 7.77 (d, *J* = 8.0 Hz, 1H), 7.66 (t, *J* = 8.0 Hz, 1H), 7.55 (t, *J* = 8.0 Hz, 1H), 7.49 (d, *J* = 8.0 Hz, 1H),7.39-7.34 (m, 2H), 7.34-7.22 (m, 3H), 6.91 (d, *J* = 15.6 Hz, 1H), 6.25 (d, *J* = 16.0 Hz, 1H), 4.65 (brs, 1H), 1.49-1.41 (m, 1H), 0.78-0.71 (m, 1H), 0.69-0.62 (m, 1H), 0.59-0.54 (m, 1H), 0.53-0.46 (m, 1H). ^{13}C NMR (100 MHz, $CDCl_3$) δ 143.2, 135.9, 133.7, 132.5, 129.8, 129.2, 128.9, 128.6, 127.8, 127.1, 124.7, 121.6, 67.0, 20.3, 2.6, 0.7. HPLC [Enantiocol Chiral AD, hexane/*i*-PrOH = 90/10, 210 nm, 1.5 mL/min. t_{R1} = 22.3 min (major), t_{R2} = 40.8 min (minor)]; ee = 90%, $[\alpha]^{25}_D$ = +4.4 (c = 0.91, $CHCl_3$); HRMS (ESI) calcd for $C_{18}H_{18}NO_2S$ $(M+H)^+$ 312.1058, found 312.1062.

(*R*,*E*)-3-Cyclohexyl-3-styryl-2,3-dihydrobenzo[*d*]isothiazole 1,1-dioxide (3sa).

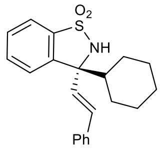

White ointment, 59.3 mg, yield: 84%. ^{1}H NMR (400 MHz, $CDCl_3$) δ 7.75 (d, *J* = 8.0 Hz, 1H), 7.64 (t, *J* = 7.6 Hz, 1H), 7.51 (t, *J* = 7.6 Hz, 1H), 7.41 (d, *J* = 8.0 Hz, 1H), 7.38-7.26 (m, 4H), 7.25-7.19 (m, 1H), 6.80 (d, *J* = 16.0 Hz, 1H), 6.35 (d, *J* = 16.0 Hz, 1H), 4.76 (brs, 1H), 2.08-1.96 (m, 2H), 1.88-1.80 (m, 1H), 1.76-1.60 (m, 3H), 1.89-0.98 (m, 4H), 0.92-0.84 (m, 1H). ^{13}C NMR (100 MHz, $CDCl_3$) δ 142.2,

136.1, 134.4, 133.7, 131.0, 130.6, 129.5, 128.8, 128.2, 126.9, 123.7, 121.9, 70.7, 47.1, 27.9, 26.7, 26.6, 26.2, 26.2. HPLC [Enantiocol Chiral AD, hexane/*i*-PrOH = 90/10, 210 nm, 1.5 mL/min. t_{R1} = 26.5 min (major), t_{R2} = 42.8 min (minor)]; ee = 88%, $[\alpha]^{25}_{D}$ = -35.4 (c = 0.80, $CHCl_3$); HRMS (ESI) calcd for $C_{21}H_{24}NO_2S$ $(M+H)^+$ 354.1528, found 354.1526.

(*S*,*E*)-2-Methyl-4-phenyl-4-styryl-1,2,5-thiadiazolidin-3-one 1,1-dioxide (3ta).

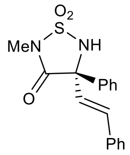

White ointment, 44.6 mg, yield: 68%. ^1H NMR (400 MHz, $CDCl_3$) δ 7.65 (d, *J* = 8.0 Hz, 1H), 7.47-7.36 (m, 3H), 7.35-7.29 (m, 3H), 7.29-7.23 (m, 2H), 7.09 (d, *J* = 1.6 Hz, 1H), 6.79 (d, *J* = 16.0 Hz, 1H), 6.45 (d, *J* = 16.0 Hz, 1H), 3.91 (s, 3H). ^{13}C NMR (100 MHz, $CDCl_3$) δ 168.3, 137.3, 135.3, 134.4, 129.8, 129.5, 129.1, 129.0, 127.3, 127.1, 125.1, 73.8, 26.4. HPLC [Enantiocol Chiral AD, hexane/*i*-PrOH = 90/10, 210 nm, 1.0 mL/min. t_{R1} = 13.1 min (minor), t_{R2} = 18.4 min (major)]; ee = 86%, $[\alpha]^{25}_{D}$ = +1.2 (c = 0.68, $CHCl_3$); HRMS (ESI) calcd for $C_{17}H_{17}N_2O_3S$ $(M+H)^+$ 329.0960, found 329.0961.

(*R*,*E*)-3-Methyl-3-(4-methylstyryl)-2,3-dihydrobenzo[*d*]isothiazole 1,1-dioxide (**3ib**).

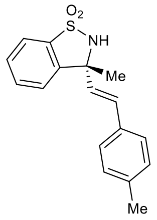

White ointment, 58.6 mg, yield: 98%. ^1H NMR (400 MHz, $CDCl_3$) δ 7.77 (d, *J* = 8.0 Hz, 1H), 7.63 (t, *J* = 7.6 Hz, 1H), 7.53 (t, *J* = 7.6 Hz, 1H), 7.38 (d, *J* = 7.6 Hz, 1H), 7.29-7.22 (m, 2H), 7.11 (d, *J* = 8.0 Hz, 2H), 6.70 (d, *J* = 15.6 Hz, 1H), 6.29 (d, *J* = 16.0 Hz, 1H), 4.84 (brs, 1H), 2.33 (s, 3H), 1.84 (s, 3H). ^{13}C NMR (100 MHz, $CDCl_3$) δ 144.1, 138.6, 135.0, 133.7, 133.0, 130.8, 130.3, 129.6, 127.8, 126.9, 124.2, 121.6, 64.0, 27.8, 21.4. HPLC [Enantiocol Chiral AD, hexane/*i*-PrOH = 90/10, 210 nm, 1.5 mL/min. t_{R1} = 23.3 min (major), t_{R2} = 49.1 min (minor)]; ee = 97%, $[\alpha]^{25}_{D}$ = -23.6 (c = 0.88, $CHCl_3$); HRMS (ESI) calcd for $C_{17}H_{18}NO_2S$ $(M+H)^+$ 300.1058, found 300.1068.

(*R*,*E*)-3-(4-Chlorostyryl)-3-methyl-2,3-dihydrobenzo[*d*]isothiazole 1,1-dioxide (**3ic**).

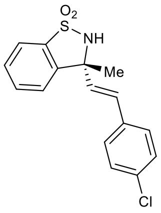

White solid, 61.2 mg, yield: 96%, Mp: 46-47 °C. ^1H NMR (400 MHz, $CDCl_3$) δ 7.77 (d, *J* = 7.6 Hz, 1H), 7.64 (t, *J* = 7.6 Hz, 1H), 7.53 (t, *J* = 7.6 Hz, 1H), 7.39 (d, *J* = 7.6 Hz, 1H), 7.34-7.19 (m, 4H), 6.69 (d, *J* = 16.0 Hz, 1H), 6.32 (d, *J* = 16.0 Hz, 1H), 5.07 (brs, 1H), 1.84 (s, 3H). ^{13}C NMR (100 MHz, $CDCl_3$) δ 143.7, 134.9, 134.4, 134.2, 133.8, 132.0, 129.7, 129.4, 129.0, 128.3, 124.1, 121.6, 63.9, 27.7. HPLC [Enantiocol Chiral AD, hexane/*i*-PrOH = 90/10, 210 nm, 1.5 mL/min. t_{R1} = 34.1 min (major), t_{R2} = 54.9 min (minor)]; ee = 99%, $[\alpha]^{25}_{D}$ = -22.1 (c = 0.73, $CHCl_3$); HRMS (ESI) calcd for $C_{16}H_{15}ClNO_2S$ $(M+H)^+$ 320.0512, found 320.0505.

(*R*,*E*)-3-(4-Fluorostyryl)-3-methyl-2,3-dihydrobenzo[*d*]isothiazole 1,1-dioxide (**3id**).

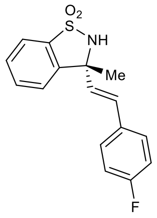

White ointment, 60.0 mg, yield: 99%. ^1H NMR (400 MHz, $CDCl_3$) δ 7.77 (d, *J* = 8.0 Hz, 1H), 7.63 (td, *J* = 7.6, 0.8 Hz, 1H), 7.53 (td, *J* = 7.6, 0.8 Hz, 1H), 7.39 (d, *J* = 7.6 Hz, 1H), 7.36-7.30 (m, 2H), 7.03-6.95 (m, 2H), 6.70 (d, *J* = 16.0 Hz, 1H), 6.26 (d, *J* = 16.0 Hz, 1H), 1.84 (s, 3H). ^{13}C NMR (100 MHz, $CDCl_3$) δ 162.9 (d, *J* = 249.0 Hz), 143.9, 135.0, 133.7, 132.0 (d, *J* = 2.3 Hz), 131.2, 129.7, 129.6, 128.6 (d, *J* = 8.1 Hz), 124.1, 121.6, 115.8 (d, *J* = 21.8 Hz), 63.9, 27.7. HPLC [Enantiocol Chiral AD, hexane/*i*-PrOH = 90/10, 210 nm, 1.5 mL/min. t_{R1} = 29.4 min (major), t_{R2} = 51.7 min (minor)]; ee > 99%, $[\alpha]^{25}_{D}$ = -12.5 (c = 0.77, $CHCl_3$); HRMS (ESI) calcd for $C_{16}H_{15}FNO_2S$ $(M+H)^+$ 304.0808, found 304.0811.

(*R*,*E*)-3-(3-Fluorostyryl)-3-methyl-2,3-dihydrobenzo[*d*]isothiazole 1,1-dioxide (**3ie**).

White ointment, 60.1 mg, yield: 99%. ^1H NMR (400 MHz, $CDCl_3$) δ 7.77 (d, *J* = 7.6 Hz, 1H), 7.64 (t, *J* = 7.6 Hz, 1H),

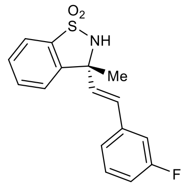

7.54 (t, *J* = 7.6 Hz, 1H), 7.40 (d, *J* = 7.6 Hz, 1H), 7.29-7.21 (m, 1H), 7.12 (d, *J* = 7.6 Hz, 1H), 7.06 (d, *J* = 10.0 Hz, 1H), 6.94 (td, *J* = 8.4, 2.4 Hz, 1H), 6.72 (d, *J* = 16.0 Hz, 1H), 6.35 (d, *J* = 16.0 Hz, 1H), 5.05 (brs, 1H), 1.85 (s, 3H). ^{13}C NMR (100 MHz, $CDCl_3$) δ 163.0 (d, *J* = 246.8 Hz), 143.4, 138.0 (d, *J* = 7.8 Hz), 134.7, 133.6, 132.5, 130.2 (d, *J* = 8.4 Hz), 129.6, 129.4 (d, *J* = 2.4 Hz), 123.8, 122.7 (d, *J* = 2.8 Hz), 121.5, 115.2 (d, *J* = 21.4 Hz), 113.3 (d, *J* = 22.0 Hz), 63.6, 27.5. HPLC [Enantiocol Chiral AD, hexane/*i*-PrOH = 90/10, 210 nm, 1.5 mL/min. t_{R1} = 26.3 min (minor), t_{R2} = 28.0 min (major)]; ee > 99%, $[\alpha]^{25}_{D}$ = -11.5 (c = 0.63, $CHCl_3$); HRMS (ESI) calcd for $C_{16}H_{15}FNO_2S$ $(M+H)^+$ 304.0808, found 304.0805.

(*R*,*E*)-3-Methyl-3-(4-(trifluoromethyl)styryl)-2,3-dihydrobenzo[*d*]isothiazole 1,1-dioxide (**3if**).

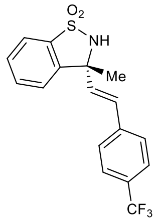

White ointment, 69.2 mg, yield: 98%. ^{1}H NMR (400 MHz, $CDCl_3$) δ 7.78 (d, *J* = 8.0 Hz, 1H), 7.65 (td, *J* = 7.6, 1.2 Hz, 1H), 7.59-7.49 (m, 3H), 7.47-7.39 (m, 3H), 6.79 (d, *J* = 16.0 Hz, 1H), 6.44 (d, *J* = 15.6 Hz, 1H), 5.11 (brs, 1H), 1.86 (s, 3H). ^{13}C NMR (100 MHz, $CDCl_3$) δ 143.2, 139.2, 134.7, 133.7, 133.6, 130.1 (q, *J* = 32.3 Hz), 129.6, 129.1, 127.0, 125.6 (q, *J* = 3.7 Hz), 124.0 (q, *J* = 273.0 Hz), 123.8, 121.5, 63.6, 27.5. HPLC [Enantiocol Chiral AD, hexane/*i*-PrOH = 90/10, 210 nm, 1.5 mL/min. t_{R1} = 34.9 min (major), t_{R2} = 50.2 min (minor)]; ee > 99%, $[\alpha]^{25}_{D}$ = -19.9 (c = 0.87, $CHCl_3$); HRMS (ESI) calcd for $C_{17}H_{15}F_3NO_2S$ $(M+H)^+$ 354.0776, found 354.0764.

(*R*,*E*)-3-Methyl-3-(pent-1-en-1-yl)-2,3-dihydrobenzo[*d*]isothiazole 1,1-dioxide (**3ig**).

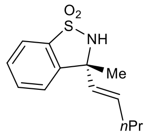

White ointment, 48.2 mg, yield: 96%. ^{1}H NMR (400 MHz, $CDCl_3$) δ 7.74 (dt, *J* = 8.0, 0.8 Hz, 1H), 7.61 (td, *J* = 7.6, 1.2 Hz, 1H), 7.51 (td, *J* = 7.6, 0.8 Hz, 1H), 7.31 (dt, *J* = 8.0, 0.8 Hz, 1H), 5.82 (dt, *J* = 15.6, 6.8 Hz, 1H), 5.65 (dt, *J* = 15.2, 1.2 Hz, 1H), 4.63 (brs, 1H), 2.03 (qt, *J* = 7.2, 1.2 Hz, 2H), 1.72 (s, 3H), 1.45-1.35 (m, 2H), 0.88 (t, *J* = 7.2 Hz, 3H). ^{13}C NMR (100 MHz, $CDCl_3$) δ 144.7, 135.1, 133.5, 132.7, 132.5, 129.4, 124.2, 121.4, 63.9, 34.3, 27.8, 22.3, 13.8. HPLC [Enantiocol Chiral OX-3, hexane/*i*-PrOH = 90/10, 210 nm, 1.0 mL/min. t_{R1} = 33.3 min (minor), t_{R2} = 37.2 min (major)]; ee = 98%, $[\alpha]^{25}_{D}$ = -7.6 (c = 0.97, $CHCl_3$); HRMS (ESI) calcd for $C_{13}H_{18}NO_2S$ $(M+H)^+$ 252.1058, found 252.1069.

(*R*,*E*)-3-(Hex-1-en-1-yl)-3-methyl-2,3-dihydrobenzo[*d*]isothiazole 1,1-dioxide (**3ih**).

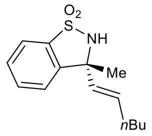

White ointment, 51.4 mg, yield: 97%. ^{1}H NMR (400 MHz, $CDCl_3$) δ 7.72 (d, *J* = 7.6 Hz, 1H), 7.60 (t, *J* = 7.6 Hz, 1H), 7.49 (t, *J* = 7.6 Hz, 1H), 7.30 (d, *J* = 7.6 Hz, 1H), 5.82 (dt, *J* = 15.2, 6.8 Hz, 1H), 5.64 (d, *J* = 15.6 Hz, 1H), 4.79 (brs, 1H), 2.04 (q, *J* = 6.8 Hz, 2H), 1.70 (s, 3H), 1.36-1.24 (m, 4H), 0.87 (t, *J* = 6.8 Hz, 3H). ^{13}C NMR (100 MHz, $CDCl_3$) δ 144.7, 135.1, 133.5, 132.8, 132.2, 129.4, 124.2, 121.4, 63.9, 32.0, 31.3, 27.8, 22.4, 14.1. HPLC [Enantiocol Chiral AD, hexane/*i*-PrOH = 90/10, 210 nm, 1.5 mL/min. t_{R1} = 8.4 min (major), t_{R2} = 9.8 min (minor)]; ee = 98%, $[\alpha]^{25}_{D}$ = -6.6 (c = 0.66, $CHCl_3$); HRMS (ESI) calcd for $C_{14}H_{20}NO_2S$ $(M+H)^+$ 266.1215, found 266.1223.

(*R*,*E*)-3-Methyl-3-(4-methylpent-1-en-1-yl)-2,3-dihydrobenzo[*d*]isothiazole 1,1-dioxide (**3ii**).

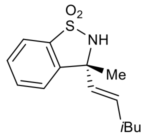

White solid, 49.3 mg, yield: 93%, Mp: 86-87 °C. ^{1}H NMR (400 MHz, $CDCl_3$) δ 7.74 (d, *J* = 7.6 Hz, 1H), 7.61 (td, *J* = 7.6, 0.8 Hz, 1H), 7.50 (t, *J* = 7.6 Hz, 1H), 7.31 (d, *J* = 8.0 Hz, 1H), 5.80 (dt, *J* = 15.2, 7.2 Hz, 1H), 5.63 (d, *J* = 15.6 Hz, 1H), 4.72 (brs, 1H), 2.00-1.85 (m, 2H), 1.72 (s, 3H), 1.67-1.59 (m, 1H), 0.86 (d, *J* = 6.8 Hz, 6H). ^{13}C NMR (100 MHz, $CDCl_3$) δ 144.7, 133.5, 133.4, 131.6, 129.4, 127.8, 124.1, 121.4, 63.9, 41.5, 28.4, 27.8, 22.5, 22.4. HPLC [Enantiocol Chiral AD, hexane/*i*-PrOH = 90/10, 210 nm, 1.5 mL/min. t_{R1} = 8.7

min (major), t_{R2} = 12.0 min (minor)]; ee = 98%, $[\alpha]^{25}_{D}$ = -9.4 (c = 0.53, $CHCl_3$); HRMS (ESI) calcd for $C_{14}H_{20}NO_2S$ $(M+H)^+$ 266.1215, found 266.1216.

(*R*,*E*)-3-(2-Cyclohexylvinyl)-3-methyl-2,3-dihydrobenzo[*d*]isothiazole 1,1-dioxide (**3ij**).

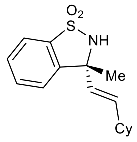

White solid, 53.0 mg, yield: 91%, Mp: 73-74 °C. ^{1}H NMR (400 MHz, $CDCl_3$) δ7.74 (dt, *J* = 7.6, 0.8 Hz, 1H), 7.61 (td, *J*= 7.2, 1,2 Hz, 1H), 7.51 (td, *J* = 7.2, 1.2 Hz, 1H), 7.29 (dt, *J* = 8.0, 0.8 Hz, 1H), 5.78 (dd, *J* = 15.6, 6.8 Hz, 1H), 5.59 (dd, *J* = 15.6, 1.2 Hz, 1H), 4.55 (brs, 1H), 2.02-1.94 (m, 1H), 1.74-1.63 (m, 5H), 1.71 (s, 3H), 1.23-1.00 (m 5H). ^{13}C NMR (100 MHz, $CDCl_3$) δ 144.6, 138.3, 134.9, 133.3, 129.7, 129.2, 123.9, 121.2,63.7, 40.3, 32.6, 27.6, 26.0, 25.9. HPLC [Enantiocol Chiral AD, hexane/*i*-PrOH = 90/10, 210 nm, 1.5 mL/min. t_{R1} = 10.2 min (major), t_{R2} = 16.1 min (minor)]; ee = 95%, $[\alpha]^{25}_{D}$ = -8.8 (c = 0.84, $CHCl_3$); HRMS (ESI) calcd for $C_{16}H_{22}NO_2S$ $(M+H)^+$ 292.1371, found 292.1367.

(*R*)-3-(Cyclopent-1-en-1-yl)-3-methyl-2,3-dihydrobenzo[*d*]isothiazole 1,1-dioxide (**3ik**).

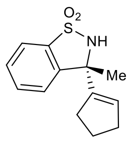

White solid, 46.8 mg, yield: 94%, Mp: 190-191 °C. ^{1}H NMR (400 MHz, $CDCl_3$) δ 7.79 (d, *J* = 8.0 Hz, 1H), 7.63 (td, *J* = 7.6, 0.8 Hz, 1H), 7.55 (t, *J* = 7.6 Hz, 1H), 7.32 (d, *J* = 8.0 Hz, 1H), 5.85 (t, *J* = 1.6 Hz, 1H), 4.60 (brs, 1H), 2.46-2.37 (m, 3H), 2.07-1.98 (m, 1H), 1.94-1.85 (m, 2H), 1.82 (s, 3H). ^{13}C NMR (100 MHz, $CDCl_3$) δ 145.3, 143.5, 134.9, 133.2, 129.3, 128.9, 123.8, 121.3, 63.6, 32.6, 31.4, 27.6, 23.5. HPLC [Daicel Chiralpak IE-H, hexane/*i*-PrOH = 90/10, 210 nm, 1.5 mL/min. t_{R1} = 28.3 min (minor), t_{R2} = 32.2 min (major)]; ee = 99%, $[\alpha]^{25}_{D}$ = -17.3 (c = 0.43, $CHCl_3$); HRMS (ESI) calcd for $C_{13}H_{16}NO_2S$ $(M+H)^+$ 250.0902, found 250.0900.

(*R*)-3-(Cyclohex-1-en-1-yl)-3-methyl-2,3-dihydrobenzo[*d*]isothiazole 1,1-dioxide (**3il**).

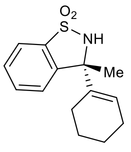

White ointment, 46.3 mg, yield: 88%. ^{1}H NMR (400 MHz, $CDCl_3$) δ 7.75 (d, *J* = 7.6 Hz, 1H), 7.60 (td, *J* = 8.0, 1.2 Hz, 1H), 7.51 (td, *J* = 8.0, 1.2 Hz, 1H), 7.24 (t, *J* = 1.2 Hz, 1H), 5.95 (t, *J* = 4.0 Hz, 1H), 2.15-2.03 (m, 4H), 1.74 (s, 3H), 1.56-1.50 (m 4H). ^{13}C NMR (100 MHz, $CDCl_3$) δ 144.1, 138.2, 135.3, 133.4, 129.4, 125.8, 124.0, 121.5, 67.0, 27.4, 25.6, 24.4, 22.9, 22.1. HPLC [Daicel Chiralpak IE-H, hexane/*i*-PrOH = 90/10, 210 nm, 0.5 mL/min. t_{R1} = 96.5 min (minor), t_{R2} = 98.4 min (major)]; ee > 99%, $[\alpha]^{25}_{D}$ = -12.5 (c = 0.85, $CHCl_3$); HRMS (ESI) calcd for $C_{14}H_{18}NO_2S$ $(M+H)^+$ 264.1058, found 264.1057.

(*R*,*E*)-Methyl 4-(4-methylstyryl)-3,4-dihydrobenzo[*e*][1,2,3]oxathiazine-4-carboxylate 2,2-dioxide (**3ab**).

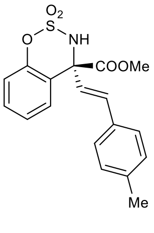

White ointment, 63.9 mg, yield: 89%. ^{1}H NMR (400 MHz, $CDCl_3$) δ 7.70 (dd, *J* = 8.0, 1.6 Hz, 1H), 7.43-7.36 (m, 1H), 7.32-7.26 (m, 3H), 7.14 (s, 1H), 7.12 (s, 1H), 7.08 (dd, *J* = 8.4, 1.2 Hz, 1H), 6.77 (d, *J* = 15.6 Hz, 1H), 6.44 (d, *J* = 16.0 Hz, 1H), 6.25 (brs, 1H), 3.90 (s, 3H), 2.34 (s, 3H). ^{13}C NMR (100 MHz, $CDCl_3$) δ 170.0, 150.3, 138.7, 133.7, 132.5, 130.7, 129.4, 129.2, 127.0, 125.6, 125.0, 119.7, 119.6, 68.5, 54.6, 21.3. HPLC [Daicel Chiralpak AD-H, hexane/*i*-PrOH = 90/10, 210 nm, 1.0 mL/min. t_{R1} = 27.2 min (major), t_{R2} = 31.4 min (minor)]; ee = 93%, $[\alpha]^{25}_{D}$ = +11.5 (c = 0.71, $CHCl_3$); HRMS (ESI) calcd for $C_{18}H_{18}NO_5S$ $(M+H)^+$ 360.0906, found 360.0910.

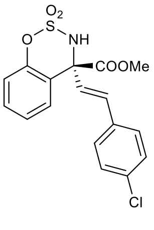

(*R*,*E*)-Methyl 4-(4-chlorostyryl)-3,4-dihydrobenzo[*e*][1,2,3]oxathiazine-4-carboxylate 2,2-dioxide (**3ac**).

White ointment, 71.3 mg, yield: 94%. ^1H NMR (400 MHz, $CDCl_3$) δ 7.70 (dd, *J* = 8.0, 1.6 Hz, 1H), 7.44-7.38 (m, 1H), 7.35-7.27 (m, 5H), 7.09 (dd, *J* = 8.4, 1.2 Hz, 1H), 6.78 (d, *J* = 15.6 Hz, 1H), 6.47 (d, *J* = 15.6 Hz, 1H), 6.30 (brs, 1H), 3.91 (s, 3H). ^{13}C NMR (100 MHz, $CDCl_3$) δ 169.7, 150.4, 134.4, 133.9, 132.5, 130.8, 128.8, 128.8, 128.3, 126.5, 125.7, 119.9, 119.2, 68.4, 54.8. HPLC [Daicel Chiralpak AD-H, hexane/*i*-PrOH = 90/10, 210 nm, 1.0 mL/min. t_{R1} = 29.5 min (major), t_{R2} = 38.9 min (minor)]; ee = 99%, $[\alpha]^{25}_D$ = +13.2 (c = 0.67, $CHCl_3$); HRMS (ESI) calcd for $C_{17}H_{15}ClNO_5S$ $(M+H)^+$ 380.0359, found 380.0360.

(*R*,*E*)-Methyl 4-(4-fluorostyryl)-3,4-dihydrobenzo[*e*][1,2,3]oxathiazine-4-carboxylate 2,2-dioxide (**3ad**).

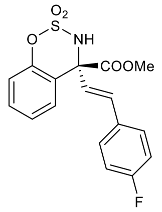

White ointment, 72.5 mg, yield: 99%. ^1H NMR (400 MHz, $CDCl_3$) δ 7.70 (d, *J* = 8.0, 1.6 Hz, 1H), 7.43-7.35 (m, 3H), 7.31-7.27 (m, 1H), 7.09 (dd, *J* = 8.0, 1.6 Hz, 1H), 7.04-6.97 (m, 2H), 6.78 (d, *J* = 15.6 Hz, 1H), 6.41 (d, *J* = 15.6 Hz, 1H), 6.30 (brs, 1H), 3.91 (s, 3H). ^{13}C NMR (100 MHz, $CDCl_3$) δ 169.8, 162.9 (d, *J* = 249.5 Hz), 150.3, 132.6, 131.5 (d, *J* = 3.3 Hz), 130.8, 128.8 (d, *J* = 7.9 Hz), 128.7, 128.7, 125.7, 119.8, 119.3, 115.6 (d, *J* = 21.8 Hz), 68.4, 54.8. HPLC [Daicel Chiralpak AD-H, hexane/*i*-PrOH = 90/10, 210 nm, 1.0 mL/min. t_{R1} = 25.6 min (major), t_{R2} = 34.2 min (minor)]; ee = 96%, $[\alpha]^{25}_D$ = +18.0 (c = 0.67, $CHCl_3$); FMS (ESI) calcd for $C_{17}H_{18}FN_2O_5S$ $(M+NH_4)^+$ 381.091440, found 381.091497.

Condition screening for asymmetric alkenylation/ring-expansion of ketimines

Supplementary Table 2.[a]

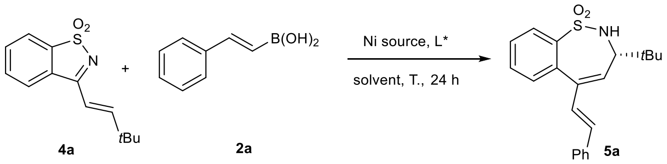

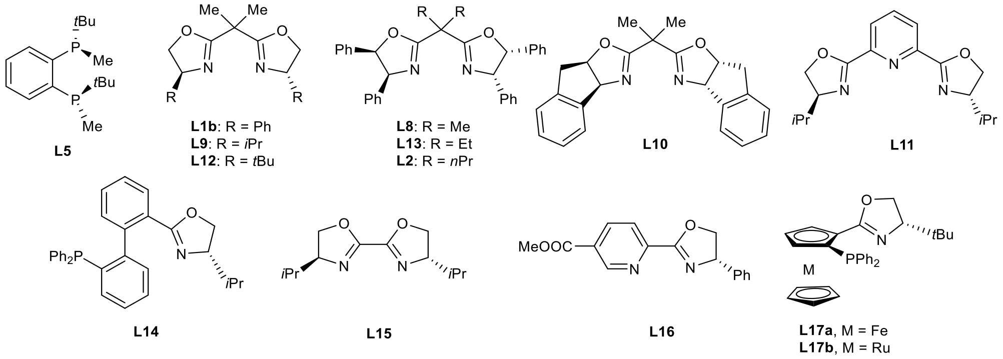

Entry	Ni source	Ligand	Solvent	T/°C	Yield (%)[b]	ee (%)[c]
1	$Ni(OTf)_2$	**L1b**	TFE	reflux	84	80
2	$Ni(OTf)_2$	**L2**	TFE	reflux	90	88
3	$Ni(OTf)_2$	**L5**	TFE	reflux	trace	---
4	$Ni(OTf)_2$	**L8**	TFE	reflux	91	85
5	$Ni(OTf)_2$	**L9**	TFE	reflux	51	77
6	$Ni(OTf)_2$	**L10**	TFE	reflux	63	78
7	$Ni(OTf)_2$	**L11**	TFE	reflux	NR	---

8	$Ni(OTf)_2$	**L12**	TFE	reflux	trace	---
9	$Ni(OTf)_2$	**L13**	TFE	reflux	89	86
10	$Ni(OTf)_2$	**L14**	TFE	reflux	trace	---
11	$Ni(OTf)_2$	**L15**	TFE	reflux	trace	---
12	$Ni(OTf)_2$	**L16**	TFE	reflux	trace	---
13	$Ni(OTf)_2$	**L17a**	TFE	reflux	NR	---
14	$Ni(OTf)_2$	**L17b**	TFE	reflux	NR	---
15	$Ni(OTf)_2$	**L2**	MeOH	reflux	trace	---
16	$Ni(OTf)_2$	**L2**	EtOH	reflux	trace	---
17	$Ni(OTf)_2$	**L2**	MeCN	reflux	NR	---
18	$Ni(OTf)_2$	**L2**	DMF	reflux	NR	---
19	$Ni(OTf)_2$	**L2**	DCE	reflux	NR	---
20	$Ni(OTf)_2$	**L2**	toluene	reflux	NR	---
21	$NiCl_2·6H_2O$	**L2**	TFE	reflux	67	77
22	$NiBr_2$	**L2**	TFE	reflux	75	48
23	$Ni(ClO_4)_2·6H_2O$	**L2**	TFE	reflux	86	83
24	$Ni(OTf)_2$	**L2**	TFE	60	90	92
25	$Ni(OTf)_2$	**L2**	TFE	50	89	97
26	---	**L2**	TFE	50	NR	---
27	$Ni(OTf)_2$	---	TFE	reflux	NR	---

[a] Reactions were carried out on a 0.20 mmol scale (**1a**) using **2a** (0.30 mmol), 5 mol % nickel salt, 7.5 mol % ligand in unpurified solvent (2.0 mL) in a test tube for 24 h which was opened to air. [b] Yield of isolated product. [c] Determined by HPLC using a chiral Enantiocol AD column. TFE = trifluoroethanol, DCE = 1,2-dichloroethane, DMF = N,N-Dimethylformamide NR = no reaction.

Ni(II)/DiPh-BOX-catalyzed asymmetric alkenylation/ring-expansion of ketimines

A test tube (100 mL, 25 * 250 mm) was charged with $Ni(OTf)_2$ (3.6 mg, 0.010 mmol, 0.050 equiv), **L2** (8.1 mg, 0.015 mmol, 0.075 equiv) and unpurified TFE (1.0 mL). The solution was stirred at 50 ºC for 20 min, then substrate (0.20 mmol, 1.0 equiv) and alkenylboronic acid (0.30 mmol, 1.5 equiv) were added into the tube. The wall of the tube was rinsed with an additional portion of TFE (1.0 mL). After stirring at 50 ºC for 24 h or 36 h in air, the reaction mixture was cooled to room temperature and the solvent was removed by rotary evaporation. The residue was purified by preparative TLC on silica gel (petroleum ether/EtOAc = 5/1) to give the product.
(Racemic ligand ***rac*-L2** was used for racemic products and the procedure is the same as above.)

(*S*,*E*)-3-(*tert*-Butyl)-5-styryl-2,3-dihydrobenzo[*f*][1,2]thiazepine 1,1-dioxide (5a).

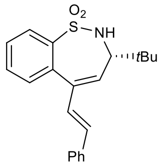

White solid, 62.8 mg, yield: 89%, Mp: 109-110 ºC. ^{1}H NMR (400 MHz, $CDCl_3$) δ 8.11-8.00 (m, 1H), 7.75-7.65 (m, 1H), 7.62-7.51 (m, 2H), 7.44-7.37 (m, 2H), 7.35-7.23 (m, 3H), 7.06 (d, *J* = 16.0 Hz, 1H), 6.50 (d, *J* = 16.4 Hz, 1H), 6.08 (d, *J* = 7.2 Hz, 1H), 5.05 (d, *J* = 2.4 Hz, 1H), 3.29 (dd, *J* = 7.2, 2.8 Hz, 1H), 1.01 (s, 9H). ^{13}C NMR (100 MHz, $CDCl_3$) δ 144.6, 139.3, 136.9, 136.0, 133.0, 132.5, 130.5, 129.3, 129.0, 128.8, 128.3, 127.3, 127.0, 126.6, 61.9, 33.9, 26.2. HPLC [Enantiocol Chiral AD,

hexane/*i*-PrOH = 90/10, 210 nm, 1.5 mL/min. t_{R1} = 7.8 min (major), t_{R2} = 17.7 min (minor)]; ee = 97%, $[\alpha]^{25}_{D}$ = +21.5 (c = 0.30, $CHCl_3$); HRMS (ESI) calcd for $C_{21}H_{24}NO_2S$ $(M+H)^+$ 354.1528, found 354.1521.

(*S*,*E*)-3-(*tert*-Butyl)-5-(4-methylstyryl)-2,3-dihydrobenzo[*f*][1,2]thiazepine 1,1-dioxide (5b).

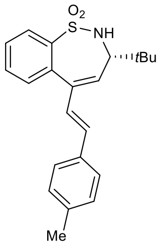

White ointment, 66.1 mg, yield: 90%. ^{1}H NMR (400 MHz, $CDCl_3$) δ 8.07 (dd, *J* = 8.0, 1.6 Hz, 1H), 7.72-7.62 (m, 1H), 7.62-7.52 (m, 2H), 7.29 (d, *J* = 8.0 Hz, 2H), 7.12 (d, *J* = 8.4 Hz, 2H), 7.00 (d, *J* = 16.4 Hz, 1H), 6.46 (d, *J* = 16.4 Hz, 1H), 6.04 (d, *J* = 6.8 Hz, 1H), 4.98 (d, *J* = 2.4 Hz, 1H), 3.28 (dd, *J* = 6.8, 2.8 Hz, 1H), 2.34 (s, 3H), 1.00 (s, 9H). ^{13}C NMR (100 MHz, $CDCl_3$) δ 144.5, 139.0, 138.0, 135.9, 133.9, 132.8, 132.2, 130.3, 129.3, 128.7, 128.0, 126.7, 126.4, 126.3, 61.7, 33.6, 25.9, 21.3. HPLC [Enantiocol Chiral AD, hexane/*i*-PrOH = 90/10, 210 nm, 1.0 mL/min. t_{R1} = 11.5 min (major), t_{R2} = 27.9 min (minor)]; ee = 82%, $[\alpha]^{25}_{D}$ = +33.6 (c = 1.6, $CHCl_3$); HRMS (ESI) calcd for $C_{22}H_{26}NO_2S$ $(M+H)^+$ 368.1684, found 368.1696.

(*S*,*E*)-3-(*tert*-Butyl)-5-(4-ethylstyryl)-2,3-dihydrobenzo[*f*][1,2]thiazepine 1,1-dioxide (5c).

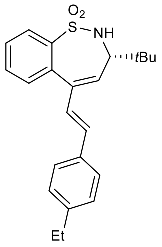

White ointment, 70.9 mg, yield: 93%. ^{1}H NMR (400 MHz, $CDCl_3$) δ 8.11-8.04 (m, 1H), 7.70-7.64 (m, 1H), 7.60-7.52 (m, 2H), 7.32 (d, *J* = 8.0 Hz, 2H), 7.15 (d, *J* = 8.0 Hz, 2H), 7.01 (d, *J* = 16.4 Hz, 1H), 6.47 (d, *J* = 16.4 Hz, 1H), 6.04 (d, *J* = 6.8 Hz, 1H), 4.98 (d, *J* = 2.0 Hz, 1H), 3.28 (dd, *J* = 7.2, 2.8 Hz, 1H), 2.64 (q, *J* = 7.6 Hz, 2H), 1.23 (t, *J* = 7.6 Hz, 3H), 1.00 (s, 9H). ^{13}C NMR (100 MHz, $CDCl_3$) δ 144.5, 144.4, 139.1, 135.9, 134.1, 132.8, 132.1, 130.3, 128.6, 128.1, 128.1, 126.8, 126.4, 126.3, 61.7, 33.6, 28.6, 25.9, 15.4. HPLC [Enantiocol Chiral AD, hexane/*i*-PrOH = 90/10, 210 nm, 1.0 mL/min. t_{R1} = 11.5 min (major), t_{R2} = 23.3 min (minor)]; ee = 84%, $[\alpha]^{25}_{D}$ = +44.6 (c = 1.3, $CHCl_3$); HRMS (ESI) calcd for $C_{23}H_{28}NO_2S$ $(M+H)^+$ 382.1841, found 382.1831.

(*S*,*E*)-3-(*tert*-Butyl)-5-(4-propylstyryl)-2,3-dihydrobenzo[*f*][1,2]thiazepine 1,1-dioxide (5d).

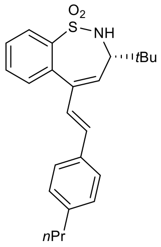

White ointment, 71.1 mg, yield: 90%. ^{1}H NMR (400 MHz, $CDCl_3$) δ 8.10-8.04 (m, 1H), 7.70-7.63 (m, 1H), 7.59-7.53 (m, 2H), 7.31 (d, *J* = 8.4 Hz, 2H), 7.13 (d, *J* = 8.0 Hz, 2H), 7.00 (d, *J* = 16.4 Hz, 1H), 6.47 (d, *J* = 16.0 Hz, 1H), 6.04 (d, *J* = 7.2 Hz, 1H), 4.98 (d, *J* = 2.8 Hz, 1H), 3.28 (dd, *J* = 7.2, 2.8 Hz, 1H), 2.57 (t, *J* = 8.0 Hz, 2H), 1.69-1.58 (m, 2H), 1.00 (s, 9H), 0.94 (t, *J* = 7.2 Hz, 3H). ^{13}C NMR (100 MHz, $CDCl_3$) δ 144.5, 142.9, 139.1, 135.9, 134.1, 132.8, 132.1, 130.3, 128.7, 128.6, 128.1, 126.7, 126.4, 126.3, 61.7, 37.8, 33.6, 25.9, 24.4, 13.8. HPLC [Enantiocol Chiral AD, hexane/*i*-PrOH = 90/10, 210 nm, 1.0 mL/min. t_{R1} = 10.8 min (major), t_{R2} = 22.2 min (minor)]; ee = 87%, $[\alpha]^{25}_{D}$ = +47.7 (c = 0.9, $CHCl_3$); HRMS (ESI) calcd for $C_{24}H_{30}NO_2S$ $(M+H)^+$ 396.1997, found 396.1986.

(*S*,*E*)-3-(*tert*-Butyl)-5-(4-fluorostyryl)-2,3-dihydrobenzo[*f*][1,2]thiazepine 1,1-dioxide (5e).

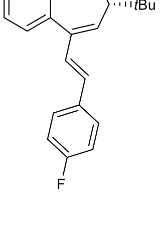

White ointment, 65.2 mg, yield: 88%. ^{1}H NMR (400 MHz, $CDCl_3$) δ 8.06 (d, *J* = 8.0 Hz, 1H), 7.66 (td, *J* = 7.6, 1.2 Hz, 1H), 7.60-7.52 (m, 2H), 7.39-7.31 (m, 2H), 7.02-6.92 (m, 3H), 6.44 (d, *J* = 16.0 Hz, 1H), 6.05 (d, *J* = 7.2 Hz, 1H), 4.97 (d, *J* = 2.4 Hz, 1H), 3.27 (dd, *J* = 7.2, 2.8 Hz, 1H), 0.99 (s, 9H). ^{13}C NMR (100 MHz, $CDCl_3$) δ 162.5 (d, J_{C-F} = 248.9 Hz), 144.3, 139.1, 135.7, 132.8 (d, J_{C-F} = 3.4 Hz), 132.2, 131.5, 130.2, 128.9 (d, J_{C-F} = 2.4 Hz), 128.8, 128.3 (d, J_{C-F} = 8.1 Hz), 127.1, 126.4, 115.6 (d, J_{C-F} = 21.8 Hz), 61.6, 33.6, 25.9. HPLC [Enantiocol Chiral AD, hexane/*i*-PrOH = 90/10, 210 nm, 1.5 mL/min. t_{R1} = 9.7 min (major), t_{R2} = 22.3 min (minor)]; ee > 99%, $[\alpha]^{25}_{D}$ = +50.2 (c = 0.41, $CHCl_3$); HRMS (ESI)

calcd for $C_{21}H_{23}FNO_2S$ $(M+H)^+$ 372.1434, found 372.1435.

(*S*,*E*)-3-(*tert*-Butyl)-5-(3-fluorostyryl)-2,3-dihydrobenzo[*f*][1,2]thiazepine 1,1-dioxide (5f).

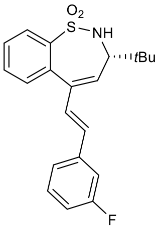

White solid, 60.8 mg, yield: 82%, Mp: 152-153 °C. ^{1}H NMR (400 MHz, $CDCl_3$) δ 8.05 (d, *J* = 7.6 Hz, 1H), 7.67 (td, *J* = 7.6, 0.8 Hz, 1H), 7.59-7.50 (m, 2H), 7.30-7.21 (m, 1H), 7.16-7.06 (m, 2H), 7.03 (d, *J* = 16.0 Hz, 1H), 6.92 (td, *J* = 8.4, 2.4 Hz, 1H), 6.44 (d, *J* = 16.0 Hz, 1H), 6.09 (d, *J* = 6.8 Hz, 1H), 5.04 (d, *J* = 2.8 Hz, 1H), 3.28 (dd, *J* = 7.2, 2.8 Hz, 1H), 0.99 (s, 9H). ^{13}C NMR (100 MHz, $CDCl_3$) δ 163.3 (d, $J_{C\text{-}F}$ = 246.1 Hz), 144.2, 139.3, 139.2 (d, $J_{C\text{-}F}$ = 7.8 Hz), 135.7, 132.5, 131.6, 130.6, 130.4, 130.2 (d, $J_{C\text{-}F}$ = 8.5 Hz), 129.0, 128.3, 126.6, 122.9, 115.0 (d, $J_{C\text{-}F}$ = 21.6 Hz), 113.2 (d, $J_{C\text{-}F}$ = 21.9 Hz), 61.8, 33.9, 26.1. HPLC [Enantiocol Chiral AD, hexane/*i*-PrOH = 90/10, 210 nm, 1.5 mL/min. t_{R1} = 7.7 min (major), t_{R2} = 13.6 min (minor)]; ee = 99%, $[\alpha]^{25}_D$ = +98.6 (c = 0.90, $CHCl_3$); HRMS (ESI) calcd for $C_{21}H_{23}FNO_2S$ $(M+H)^+$ 372.1434, found 372.1436.

(*S*,*E*)-3-(*tert*-Butyl)-5-(4-chlorostyryl)-2,3-dihydrobenzo[*f*][1,2]thiazepine 1,1-dioxide (5g).

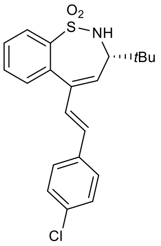

White ointment, 66.6 mg, yield: 86%. ^{1}H NMR (400 MHz, $CDCl_3$) δ 8.05 (dd, *J* = 8.0, 1.2 Hz, 1H), 7.67 (td, *J* = 7.6, 1.6 Hz, 1H), 7.58-7.51 (m, 2H), 7.34-7.24 (m, 4H), 7.00 (d, *J* = 16.0 Hz, 1H), 6.42 (d, *J* = 16.0 Hz, 1H), 6.07 (d, *J* = 6.8 Hz, 1H), 5.01 (d, *J* = 2.4 Hz, 1H), 3.27 (dd, *J* = 7.2, 2.8 Hz, 1H), 0.99 (s, 9H). ^{13}C NMR (100 MHz, $CDCl_3$) δ 144.2, 139.1, 135.5, 135.2, 133.6, 132.2, 131.3, 130.2, 129.7, 128.8, 128.8, 127.9, 127.7, 126.4, 61.6, 33.6, 25.9. HPLC [Enantiocol Chiral AD, hexane/*i*-PrOH = 90/10, 210 nm, 1.5 mL/min. t_{R1} = 10.1 min (major), t_{R2} = 23.0 min (minor)]; ee = 99%, $[\alpha]^{25}_D$ = +79.1 (c = 0.57, $CHCl_3$); HRMS (ESI) calcd for $C_{21}H_{23}ClNO_2S$ $(M+H)^+$ 388.1138, found 388.1139.

(*S*,*E*)-3-(*tert*-Butyl)-5-(3-chlorostyryl)-2,3-dihydrobenzo[*f*][1,2]thiazepine 1,1-dioxide (5h).

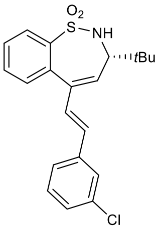

White ointment, 61.9 mg, yield: 80%. ^{1}H NMR (400 MHz, $CDCl_3$) δ 8.08 (dd, *J* = 7.6, 1.2 Hz, 1H), 7.72-7.65 (m, 1H), 7.61-7.53 (m, 2H), 7.41-7.37 (m, 1H), 7.26-7.20 (m, 3H), 7.05 (d, *J* = 16.0 Hz, 1H), 6.42 (d, *J* = 16.0 Hz, 1H), 6.10 (d, *J* = 6.8 Hz, 1H), 4.99 (d, *J* = 2.4 Hz, 1H), 3.29 (dd, *J* = 7.2, 2.8 Hz, 1H), 1.00 (s, 9H). ^{13}C NMR (100 MHz, $CDCl_3$) δ 144.1, 139.1, 138.5, 135.4, 134.6, 132.2, 131.2, 130.4, 130.2, 129.8, 128.8, 128.1, 127.9, 126.6, 126.4, 124.9, 61.6, 33.6, 25.9. HPLC [Enantiocol Chiral AD, hexane/*i*-PrOH = 90/10, 210 nm, 1.0 mL/min. t_{R1} = 16.3 min (major), t_{R2} = 21.6 min (minor)]; ee = 97%, $[\alpha]^{25}_D$ = +35.8 (c = 0.3, $CHCl_3$); HRMS (ESI) calcd for $C_{21}H_{22}ClNO_2SNa$ $(M+Na)^+$ 410.0957, found 410.0948.

(*S*,*E*)-5-(4-Bromostyryl)-3-(*tert*-butyl)-2,3-dihydrobenzo[*f*][1,2]thiazepine 1,1-dioxide (5i).

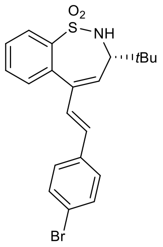

White ointment, 71.5 mg, yield: 83%. ^{1}H NMR (400 MHz, $CDCl_3$) δ 8.05 (d, *J* = 8.0 Hz, 1H), 7.70-7.64 (m, 1H), 7.59-7.51 (m, 2H), 7.42 (d, *J* = 8.4 Hz, 2H), 7.24 (d, *J* = 8.4 Hz, 2H), 7.02 (d, *J* = 16.4 Hz, 1H), 6.40 (d, *J* = 16.4 Hz, 1H), 6.07 (d, *J* = 7.2 Hz, 1H), 5.01 (brs, 1H), 3.27 (d, *J* = 7.2 Hz, 1H), 0.99 (s, 9H). ^{13}C NMR (100 MHz, $CDCl_3$) δ 144.1, 139.0, 135.6, 135.4, 132.2, 131.7, 131.3, 130.2, 129.7, 128.8, 128.2, 127.7, 126.4, 121.8, 61.6, 33.6, 25.9. HPLC [Enantiocol Chiral AD, hexane/*i*-PrOH = 90/10, 210 nm, 1.0 mL/min. t_{R1} = 17.6 min (major), t_{R2} = 42.9 min (minor)]; ee = 95%, $[\alpha]^{25}_D$ = +22.9 (c = 0.27, $CHCl_3$); HRMS (ESI) calcd for $C_{21}H_{23}BrNO_2S$ $(M+H)^+$ 432.0633, found 432.0631.

(*S*,*E*)-3-(*tert*-Butyl)-5-(4-(trifluoromethyl)styryl)-2,3-dihydrobenzo[*f*][1,2]thiazepine 1,1-dioxide (5j).

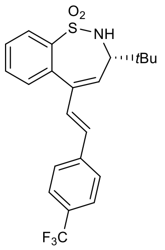

White ointment, 67.4 mg, yield: 80%. ^{1}H NMR (400 MHz, $CDCl_3$) δ 8.06 (d, *J* = 8.0 Hz, 1H), 7.68 (t, *J* = 7.6 Hz, 1H), 7.61-7.51 (m, 4H), 7.50-7.44 (m, 2H), 7.11 (d, *J* = 16.0 Hz, 1H), 6.49 (d, *J* = 16.0 Hz, 1H), 6.13 (d, *J* = 7.2 Hz, 1H), 5.03 (d, *J* = 2.0 Hz, 1H), 3.29 (dd, *J* = 7.2, 1.6 Hz, 1H), 1.00 (s, 9H). ^{13}C NMR (100 MHz, $CDCl_3$) δ 144.2, 140.4, 139.4, 135.5, 132.5, 131.7, 131.3, 130.3, 130.0 (q, $J_{C\text{-}F}$ = 32.7 Hz), 129.1, 129.0, 128.4, 127.0, 126.7, 125.7 (q, $J_{C\text{-}F}$ = 3.4 Hz), 124.3 (q, $J_{C\text{-}F}$ = 272.0 Hz), 61.9, 33.9, 26.1. HPLC [Enantiocol Chiral AD, hexane/*i*-PrOH = 90/10, 210 nm, 1.0 mL/min. t_{R1} = 15.9 min (major), t_{R2} = 34.9 min (minor)]; ee = 98%, $[\alpha]^{25}_{D}$ = +104.5 (c = 0.15, $CHCl_3$); HRMS (ESI) calcd for $C_{22}H_{23}F_3NO_2S$ $(M+H)^+$ 422.1402, found 422.1407.

(*S*,*E*)-3-(2,5-Dimethylhex-4-en-2-yl)-5-styryl-2,3-dihydrobenzo[*f*][1,2]thiazepine 1,1-dioxide (5k).

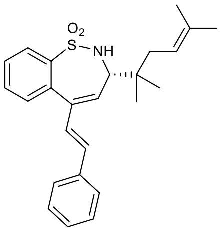

White ointment, 75.7 mg, yield: 93%. ^{1}H NMR (400 MHz, $CDCl_3$) δ 8.08-8.02 (m, 1H), 7.70-7.63 (m, 1H), 7.58-7.52 (m, 2H), 7.41-7.36 (m, 2H), 7.33-7.27 (m, 2H), 7.26-7.21 (m, 1H), 7.04 (d, *J* = 16.4 Hz, 1H), 6.49 (d, *J* = 16.0 Hz, 1H), 6.10 (d, *J* = 6.8 Hz, 1H), 5.10-5.04 (m, 1H), 5.00 (d, *J* = 2.4 Hz, 1H), 3.34 (dd, *J* = 7.2, 2.8 Hz, 1H), 1.99 (d, *J* = 7.6 Hz, 2H), 1.65 (s, 3H), 1.58 (s, 3H), 0.99 (s, 3H), 0.97 (s, 3H). ^{13}C NMR (100 MHz, $CDCl_3$) δ 144.4, 139.2, 136.9, 136.0, 134.9, 132.9, 132.4, 130.4, 129.3, 128.9, 128.8, 128.3, 127.2, 127.0, 126.5, 119.5, 60.8, 37.8, 37.4, 26.3, 23.6, 23.3, 18.2. HPLC [Enantiocol Chiral AD, hexane/*i*-PrOH = 90/10, 210 nm, 1.0 mL/min. t_{R1} = 9.2 min (major), t_{R2} = 19.5 min (minor)]; ee = 94%, $[\alpha]^{25}_{D}$ = +23.5 (c = 0.31, $CHCl_3$); HRMS (ESI) calcd for $C_{25}H_{30}NO_2S$ $(M+H)^+$ 408.1997, found 408.2007.

(*S*,*E*)-3-(2,5-Dimethylhex-4-en-2-yl)-5-(4-ethylstyryl)-2,3-dihydrobenzo[*f*][1,2]thiazepine 1,1-dioxide (5l).

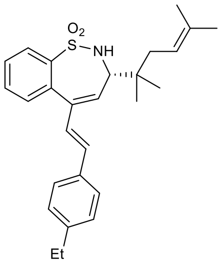

White ointment, 75.7 mg, yield: 87%. ^{1}H NMR (400 MHz, $CDCl_3$) δ 8.05 (d, *J* = 7.6 Hz, 1H), 7.70-7.61 (m, 1H), 7.59-7.50 (m, 2H), 7.31 (d, *J* = 8.0 Hz, 2H), 7.13 (d, *J* = 8.0 Hz, 2H), 6.99 (d, *J* = 16.0 Hz, 1H), 6.46 (d, *J* = 16.4 Hz, 1H), 6.07 (d, *J* = 7.2 Hz, 1H), 5.06 (t, *J* = 8.0 Hz, 1H), 4.99 (d, *J* = 2.4 Hz, 1H), 3.33 (dd, *J* = 7.2, 2.8 Hz, 1H), 2.63 (q, *J* = 7.6 Hz, 2H), 1.98 (d, *J* = 7.6 Hz, 2H), 1.65 (s, 3H), 1.58 (s, 3H), 1.22 (t, *J* = 7.6 Hz, 3H), 0.98 (s, 3H), 0.97 (s, 3H). ^{13}C NMR (100 MHz, $CDCl_3$) δ 144.6, 144.6, 139.2, 136.2, 134.9, 134.3, 132.9, 132.3, 130.4, 128.8, 128.3, 127.0, 126.6, 126.5, 119.5, 60.8, 37.9, 37.4, 28.9, 26.3, 23.6, 23.3, 18.2, 15.7. HPLC [Enantiocol Chiral AD, hexane/*i*-PrOH = 90/10, 210 nm, 1.0 mL/min. t_{R1} = 9.1 min (major), t_{R2} = 18.4 min (minor)]; ee = 81%, $[\alpha]^{25}_{D}$ = +18.8 (c = 0.9, $CHCl_3$); HRMS (ESI) calcd for $C_{27}H_{34}NO_2S$ $(M+H)^+$ 436.2310, found 436.2319.

(*S*,*E*)-3-(2,5-Dimethylhex-4-en-2-yl)-5-(4-propylstyryl)-2,3-dihydrobenzo[*f*][1,2]thiazepine 1,1-dioxide (5m).

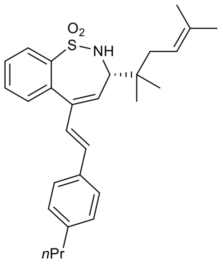

White ointment, 81.7 mg, yield: 91%. ^{1}H NMR (400 MHz, $CDCl_3$) δ 8.08-8.01 (m, 1H), 7.69-7.62 (m, 1H), 7.58-7.50 (m, 2H), 7.30 (d, *J* = 8.0 Hz, 2H), 7.11 (d, *J* = 8.0 Hz, 2H), 6.99 (d, *J* = 16.0 Hz, 1H), 6.46 (d, *J* = 16.0 Hz, 1H), 6.06 (d, *J* = 7.2 Hz, 1H), 5.06 (t, *J* = 8.0 Hz, 1H), 4.98 (d, *J* = 2.0 Hz, 1H), 3.33 (dd, *J* = 7.2, 2.4 Hz, 1H), 2.56 (t, *J* = 7.6 Hz, 2H), 1.98 (d, *J* = 7.6 Hz, 2H), 1.65 (s, 3H), 1.58 (s, 3H), 1.68-1.60 (m, 2H), 0.98 (s, 3H), 0.97 (s, 3H) ,0.92 (t, *J* = 7.6 Hz, 3H). ^{13}C NMR (100 MHz, $CDCl_3$) δ 144.6, 143.1, 139.2, 136.2, 134.9, 134.3, 132.9, 132.3, 130.4, 128.9, 128.8, 128.3, 126.9, 126.6, 126.5, 119.5, 60.8, 38.0, 37.9, 37.4, 26.3, 24.7, 23.6, 23.3, 18.2, 14.0. HPLC [Enantiocol Chiral AD, hexane/*i*-PrOH = 90/10, 210 nm, 1.0 mL/min. t_{R1} = 8.8 min (major), t_{R2} = 17.8 min (minor)]; ee = 92%, $[\alpha]^{25}_{D}$ = +21.3 (c = 1.0, $CHCl_3$); HRMS (ESI) calcd for $C_{28}H_{36}NO_2S$ $(M+H)^+$ 450.2467, found 450.2473.

(*S*,*E*)-5-(3-Chlorostyryl)-3-(2,5-dimethylhex-4-en-2-yl)-2,3-dihydrobenzo[*f*][1,2]thiazepine 1,1-dioxide (5n).

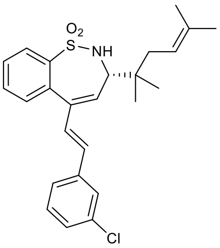

White ointment, 74.1 mg, yield: 84%. ^{1}H NMR (400 MHz, $CDCl_3$) δ 8.05 (d, *J* = 8.0 Hz, 1H), 7.70-7.64 (m, 1H), 7.59-7.54 (m, 1H), 7.52 (d, *J* = 7.6 Hz, 1H), 7.25-7.18 (m, 3H), 7.04 (d, *J* = 16.0 Hz, 1H), 6.41 (d, *J* = 16.0 Hz, 1H), 6.12 (d, *J* = 7.2 Hz, 1H), 5.09-5.04 (m, 1H), 5.02 (brs, 1H), 3.33 (d, *J* = 7.2 Hz, 1H), 1.98 (d, *J* = 8.0 Hz, 2H), 1.65 (s, 3H), 1.58 (s, 3H), 0.98 (s, 3H), 0.96 (s, 3H). ^{13}C NMR (100 MHz, $CDCl_3$) δ 144.1, 139.2, 138.8, 135.7, 135.0, 134.8, 132.4, 131.4, 130.6, 130.3, 130.0, 129.0, 128.3, 128.1, 126.8, 126.6, 125.2, 119.4, 60.7, 37.8, 37.3, 26.3, 23.6, 23.2, 18.2. HPLC [Enantiocol Chiral AD, hexane/*i*-PrOH = 90/10, 210 nm, 1.0 mL/min. t_{R1} = 10.9 min (minor), t_{R2} = 14.3 min (major)]; ee > 99%, $[\alpha]^{25}_{D}$ = +27.4 (c = 0.68, $CHCl_3$); HRMS (ESI) calcd for $C_{25}H_{29}ClNO_2S$ $(M+H)^+$ 442.1608, found 442.1620.

(*R*,*E*)-3-Isopropyl-5-styryl-2,3-dihydrobenzo[*f*][1,2]thiazepine 1,1-dioxide (5o).

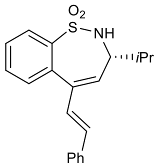

White ointment, 63.1 mg, yield: 93%. ^{1}H NMR (400 MHz, $CDCl_3$) δ 8.13-8.06 (m, 1H), 7.72-7.66 (m, 1H), 7.62-7.55 (m, 2H), 7.45-7.39 (m, 2H), 7.36-7.30 (m, 2H), 7.28-7.22 (m, 1H), 7.06 (d, *J* = 16.0 Hz, 1H), 6.53 (d, *J* = 16.0 Hz, 1H), 6.02 (d, *J* = 6.8 Hz, 1H), 4.96 (d, *J* = 2.4 Hz, 1H), 3.39 (td, *J* = 6.4, 2.8 Hz, 1H), 1.97-1.86 (m, 1H), 1.03 (d, *J* = 6.8 Hz, 3H), 1.00 (d, *J* = 6.8 Hz, 3H). ^{13}C NMR (100 MHz, $CDCl_3$) δ 144.6, 139.0, 136.6, 135.8, 132.9, 132.2, 130.3, 128.8, 128.7, 128.6, 128.1, 127.9, 126.7, 126.4, 58.3, 32.1, 18.5, 18.4. HPLC [Enantiocol Chiral OD, hexane/*i*-PrOH = 95/5, 210 nm, 0.5 mL/min. t_{R1} = 54.3 min (major), t_{R2} = 62.0 min (minor)]; ee = 94%, $[\alpha]^{25}_{D}$ = +69.9 (c = 0.25, $CHCl_3$); HRMS (ESI) calcd for $C_{20}H_{22}NO_2S$ $(M+H)^+$ 340.1371, found 340.1375.

(*R*,*E*)-3-Cyclohexyl-5-styryl-2,3-dihydrobenzo[*f*][1,2]thiazepine 1,1-dioxide (5p).

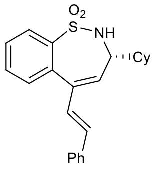

White ointment, 63.7 mg, yield: 84%. ^{1}H NMR (400 MHz, $CDCl_3$) δ 8.08 (d, *J* = 8.4 Hz, 1H), 7.68 (t, *J* = 7.6 Hz, 1H), 7.62-7.53 (m, 2H), 7.41 (d, *J* = 8.0 Hz, 2H), 7.38-7.24 (m, 3H), 7.05 (d, *J* = 16.4 Hz, 1H), 6.53 (d, *J* = 16.4 Hz, 1H), 6.04 (d, *J* = 6.8 Hz, 1H), 4.97 (brs, 1H), 3.35 (td, *J* = 6.8, 2.4 Hz, 1H), 1.89-1.65 (m, 6H), 1.25-0.90 (m, 5H). ^{13}C NMR (100 MHz, $CDCl_3$) δ 144.4, 139.0, 136.7, 135.9, 132.8, 132.2, 130.2, 128.8, 128.7, 128.6, 128.5, 128.1, 126.7, 126.4, 57.6, 41.8, 29.1, 28.7, 26.2, 25.9, 25.8. HPLC [Enantiocol Chiral AD, hexane/*i*-PrOH = 90/10, 210 nm, 1.5 mL/min. t_{R1} = 16.1 min (major), t_{R2} = 23.1 min (minor)]; ee = 91%, $[\alpha]^{25}_{D}$ = +62.3 (c = 0.25, $CHCl_3$); HRMS (ESI) calcd for $C_{23}H_{26}NO_2S$ $(M+H)^+$ 380.1684, found 380.1680.

(*S*,*E*)-3-(*tert*-Butyl)-7-methyl-5-styryl-2,3-dihydrobenzo[*f*][1,2]thiazepine 1,1-dioxide (5q).

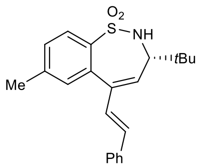

White ointment, 66.8 mg, yield: 91%. ^{1}H NMR (400 MHz, $CDCl_3$) δ 7.95 (d, *J* = 8.4 Hz, 1H), 7.44-7.38 (m, 2H), 7.38-7.30 (m, 4H), 7.29-7.25 (m, 1H), 7.04 (d, *J* = 16.0 Hz, 1H), 6.51 (d, *J* = 16.4 Hz, 1H), 6.06 (d, *J* = 7.2 Hz, 1H), 4.95 (d, *J* = 2.8 Hz, 1H), 3.27 (dd, *J* = 7.2, 2.8 Hz, 1H), 2.49 (s, 3H), 1.00 (s, 9H). ^{13}C NMR (100 MHz, $CDCl_3$) δ 144.4, 142.9, 136.7, 136.3, 135.7, 132.7, 130.7, 129.3, 129.1, 128.6, 128.0, 126.9, 126.8, 126.4, 61.7, 33.6, 25.9, 21.6. HPLC [Enantiocol Chiral AD, hexane/*i*-PrOH = 90/10, 210 nm, 1.0 mL/min. t_{R1} = 13.0 min (major), t_{R2} = 21.5 min (minor)]; ee > 99%, $[\alpha]^{25}_{D}$ = +81.7 (c = 1.7, $CHCl_3$); HRMS (ESI) calcd for $C_{22}H_{25}NO_2SNa$ $(M+Na)^+$ 390.1504, found 390.1504.

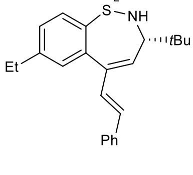

(*S*,*E*)-3-(*tert*-Butyl)-7-ethyl-5-styryl-2,3-dihydrobenzo[*f*][1,2]thiazepine 1,1-dioxide (5r).

White ointment, 66.3 mg, yield: 87%. ^{1}H NMR (400 MHz, $CDCl_3$) δ 7.98 (d, *J* = 8.4 Hz, 1H), 7.43-7.36 (m, 4H), 7.35-7.30 (m, 2H), 7.27-7.22 (m, 1H), 7.04 (d, *J* = 16.0 Hz, 1H), 6.51 (d, *J* = 16.0 Hz, 1H), 6.07 (d, *J* = 7.2 Hz, 1H), 4.96 (d, *J* = 2.8 Hz, 1H), 3.30 (dd, *J* = 7.2, 2.8 Hz, 1H),

2.78 (q, *J* = 7.6 Hz, 2H), 1.31 (t, *J* = 7.6 Hz, 3H), 1.01(s, 9H). ^{13}C NMR (100 MHz, $CDCl_3$) δ 149.3, 144.7, 137.0, 136.7, 136.0, 132.9, 130.0, 129.4, 128.9, 128.4, 128.3, 127.2, 127.0, 126.7, 61.8, 33.9, 29.1, 26.2, 15.5. HPLC [Enantiocol Chiral AD, hexane/*i*-PrOH = 90/10, 210 nm, 1.0 mL/min. t_{R1} = 12.0 min (major), t_{R2} = 17.9 min (minor)]; ee > 99%, $[\alpha]^{25}_D$ = +30.2 (c = 0.23, $CHCl_3$); HRMS (ESI) calcd for $C_{23}H_{28}NO_2S$ $(M+H)^+$ 382.1841, found 382.1840.

(*S*,*E*)-3-(*tert*-Butyl)-7-phenyl-5-styryl-2,3-dihydrobenzo[*f*][1,2]thiazepine 1,1-dioxide (5s).

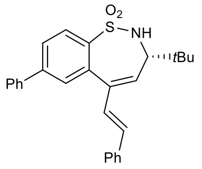

White solid, 80.6 mg, yield: 94%, Mp: 197-198 ºC. ^{1}H NMR (400 MHz, $CDCl_3$) δ 8.12 (d, *J* = 9.2 Hz, 1H), 7.80-7.73 (m, 2H), 7.67-7.60 (m, 2H), 7.54-7.38 (m, 5H), 7.34-7.27 (m, 2H), 7.26-7.22 (m, 1H), 7.08 (d, *J* = 16.0 Hz, 1H), 6.58 (d, *J* = 16.4 Hz, 1H), 6.10 (d, *J* = 6.8 Hz, 1H), 4.99 (d, *J* = 2.4 Hz, 1H), 3.56 (dd, *J* = 7.2, 2.8 Hz, 1H), 1.01(s, 9H). ^{13}C NMR (100 MHz, $CDCl_3$) δ 145.1, 144.4, 139.0, 136.2, 132.8, 129.1, 129.1, 128.8, 128.6, 128.6, 128.1, 127.3, 127.1, 126.9, 126.8, 61.4, 33.7, 25.9. HPLC [Enantiocol Chiral AD, hexane/*i*-PrOH = 90/10, 210 nm, 1.0 mL/min. t_{R1} = 10.1 min (major), t_{R2} = 17.1 min (minor)]; ee = 96%, $[\alpha]^{25}_D$ = +49.2 (c = 0.28, $CHCl_3$); HRMS (ESI) calcd for $C_{27}H_{28}NO_2S$ $(M+H)^+$ 430.1841, found 430.1838.

(*S*,*E*)-3-Propyl-5-styryl-2,3-dihydrobenzo[*f*][1,2]thiazepine 1,1-dioxide (6a).

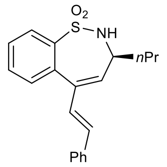

White ointment, 57.0 mg, yield: 84%. ^{1}H NMR (400 MHz, $CDCl_3$) δ 8.06 (d, *J* = 7.6 Hz, 1H), 7.69-7.63 (m, 1H), 7.58-7.52 (m, 2H), 7.38 (d, *J* = 7.6 Hz, 2H), 7.31 (t, *J* = 8.0 Hz, 2H), 7.27-7.20 (m, 1H), 7.01 (d, *J* = 16.0 Hz, 1H), 6.52 (d, *J* = 16.4 Hz, 1H), 5.94 (d, *J* = 6.8 Hz, 1H), 4.96 (d, *J* = 2.4 Hz, 1H), 3.63-3.54 (m, 1H), 1.75-1.65 (m, 2H), 1.40-1.35 (m, 2H), 0.92 (t, *J* = 7.6 Hz, 3H). ^{13}C NMR (100 MHz, $CDCl_3$) δ 144.3, 138.9, 136.6, 135.9, 133.1, 132.2, 130.3, 129.6, 128.7, 128.6, 128.1, 127.6, 126.8, 126.5, 52.9, 37.2, 19.0, 13.8. HPLC [Enantiocol Chiral AD, hexane/*i*-PrOH = 90/10, 210 nm, 1.0 mL/min. t_{R1} = 19.6 min (minor), t_{R2} = 40.1 min (major)]; ee = 88%, $[\alpha]^{25}_D$ = -81.1 (c = 0.79, $CHCl_3$); HRMS (ESI) calcd for $C_{20}H_{22}NO_2S$ $(M+H)^+$ 340.1371, found 340.1380.

(*S*,*E*)-3-Butyl-5-styryl-2,3-dihydrobenzo[*f*][1,2]thiazepine 1,1-dioxide (6b).

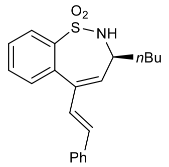

White ointment, 61.4 mg, yield: 87%. ^{1}H NMR (400 MHz, $CDCl_3$) δ 8.13-8.03 (m, 1H), 7.72-7.63 (m, 1H), 7.61-7.53 (m, 2H), 7.43-7.37 (m, 2H), 7.35-7.29 (m, 2H), 7.27-7.22 (m, 1H), 7.03 (d, *J* = 16.4 Hz, 1H), 6.53 (d, *J* = 16.4 Hz, 1H), 5.96 (d, *J* = 6.8 Hz, 1H), 4.97 (brs, 1H), 3.63-3.51 (m, 1H), 1.80-1.63 (m, 2H), 1.35-1.30 (m, 4H), 0.90 (t, *J* = 6.8 Hz, 3H). ^{13}C NMR (100 MHz, $CDCl_3$) δ 144.3, 138.8, 136.6, 135.9, 133.0, 132.2, 130.3, 129.7, 128.7, 128.6, 128.6, 128.1, 126.8, 126.5, 53.1, 34.8, 27.9, 22.5, 13.9. HPLC [Enantiocol Chiral OD, hexane/*i*-PrOH = 90/10, 210 nm, 1.0 mL/min. t_{R1} = 13.4 min (major), t_{R2} = 17.1 min (minor)]; ee = 89%, $[\alpha]^{25}_D$ = -69.3 (c = 0.29, $CHCl_3$); HRMS (ESI) calcd for $C_{21}H_{24}NO_2S$ $(M+H)^+$ 354.1528, found 354.1522.

(*S*,*E*)-3-Cyclohexyl-5-styryl-2,3-dihydrobenzo[*f*][1,2]thiazepine 1,1-dioxide (6c).

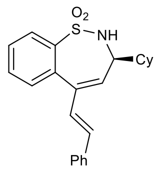

White ointment, 60.6 mg, yield: 80%. HPLC [Enantiocol Chiral AD, hexane/*i*-PrOH = 90/10, 210 nm, 1.5 mL/min. t_{R1} = 16.4 min (minor), t_{R2} = 23.6 min (major)]; ee = 83%, $[\alpha]^{25}_D$ = -40.6 (c = 0.91, $CHCl_3$);
Other data see **5p**.

(*S*,*E*)-5-(3-Methylstyryl)-3-propyl-2,3-dihydrobenzo[*f*][1,2]thiazepine 1,1-dioxide (6d).

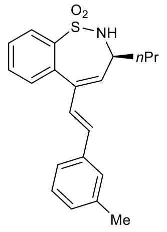

White ointment, 55.8 mg, yield: 79%. ^{1}H NMR (400 MHz, $CDCl_3$) δ 8.06 (dd, *J* = 8.0, 1.6 Hz, 1H), 7.69-7.62 (m, 1H), 7.58-7.52 (m, 2H), 7.23-7.16 (m, 3H), 7.09-7.04 (m, 1H), 7.00 (d, *J* = 16.4 Hz, 1H), 6.49 (d, *J* = 16.4 Hz, 1H), 5.93 (d, *J* = 6.8 Hz, 1H), 4.95 (brs, 1H), 3.61-3.52 (m, 1H), 2.33 (s, 3H), 1.73-1.60 (m, 2H), 1.40-1.30 (m, 2H), 0.91 (t, *J* = 7.6 Hz, 3H). ^{13}C NMR (100 MHz, $CDCl_3$) δ 144.3, 138.8, 138.2, 136.6, 136.0, 133.2, 132.2, 130.3, 129.4, 128.9, 128.7, 128.5, 128.4, 127.5, 126.4, 123.9, 52.9, 37.2, 21.4, 19.0, 13.8. HPLC [Enantiocol Chiral OD, hexane/*i*-PrOH = 90/10, 210 nm, 1.0 mL/min. t_{R1} = 13.5 min (major), t_{R2} = 16.8 min (minor)]; ee = 87%, $[\alpha]^{25}_{D}$ = -57.3 (c = 0.3, $CHCl_3$); HRMS (ESI) calcd for $C_{21}H_{24}NO_2S$ $(M+H)^+$ 354.1528, found 354.1515.

(*S*,*E*)-3-Butyl-5-(3-methylstyryl)-2,3-dihydrobenzo[*f*][1,2]thiazepine 1,1-dioxide (6e).

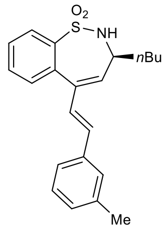

White ointment, 61.7 mg, yield: 84%. ^{1}H NMR (400 MHz, $CDCl_3$) δ 8.07 (dd, *J* = 8.4, 1.6 Hz, 1H), 7.70-7.63 (m, 1H), 7.59-7.52 (m, 2H), 7.23-7.17 (m, 3H), 7.09-7.05 (m, 1H), 7.01 (d, *J* = 16.4 Hz, 1H), 6.50 (d, *J* = 16.0 Hz, 1H), 5.94 (d, *J* = 6.8 Hz, 1H), 4.96 (d, *J* = 1.6 Hz, 1H), 3.61-3.53 (m, 1H), 2.34 (s, 3H), 1.75-1.62 (m, 2H), 1.34-1.30 (m, 4H), 0.90 (t, *J* = 6.8 Hz, 3H). ^{13}C NMR (100 MHz, $CDCl_3$) δ 144.3, 138.8, 138.2, 136.6, 136.0, 133.2, 132.2, 130.3, 129.4, 128.9, 128.7, 128.5, 128.4, 127.5, 126.4, 123.9, 53.1, 34.9, 27.9, 22.5, 21.4, 13.9. HPLC [Enantiocol Chiral OD, hexane/*i*-PrOH = 90/10, 210 nm, 1.0 mL/min. t_{R1} = 10.5 min (major), t_{R2} = 13.3 min (minor)]; ee = 89%, $[\alpha]^{25}_{D}$ = -64.7 (c = 0.25, $CHCl_3$); HRMS (ESI) calcd for $C_{22}H_{25}NO_2S$ $(M+H)^+$ 368.1684, found 368.1680.

(*S*,*E*)-3-Isobutyl-5-(3-methylstyryl)-2,3-dihydrobenzo[*f*][1,2]thiazepine 1,1-dioxide (6f).

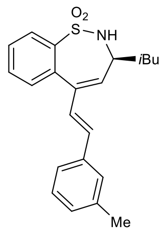

White ointment, 54.3 mg, yield: 74%. ^{1}H NMR (400 MHz, $CDCl_3$) δ 8.08 (dd, *J* = 8.0, 1.2 Hz, 1H), 7.71-7.64 (m, 1H), 7.61-7.52 (m, 2H), 7.23-7.18 (m, 3H), 7.09-7.05 (m, 1H), 7.01 (d, *J* = 16.4 Hz, 1H), 6.50 (d, *J* = 16.4 Hz, 1H), 5.92 (d, *J* = 6.8 Hz, 1H), 4.90 (brs, 1H), 3.66-3.60 (m, 1H), 2.34 (s, 3H), 2.04-1.99 (m, 1H), 1.55-1.48 (m, 2H), 0.89 (d, *J* = 6.0 Hz, 3H), 0.85 (d, *J* = 6.4 Hz, 3H). ^{13}C NMR (100 MHz, $CDCl_3$) δ 144.5, 139.0, 138.4, 136.7, 136.1, 133.4, 132.5, 130.5, 129.8, 129.1, 128.9, 128.7, 128.7, 127.7, 126.7, 124.1, 51.5, 44.3, 25.0, 22.7, 21.6. HPLC [Enantiocol Chiral OD, hexane/*i*-PrOH = 90/10, 210 nm, 1.0 mL/min. t_{R1} = 9.4 min (major), t_{R2} = 11.8 min (minor)]; ee = 88%, $[\alpha]^{25}_{D}$ = -50.4 (c = 0.37, $CHCl_3$); HRMS (ESI) calcd for $C_{22}H_{25}NO_2S$ $(M+H)^+$ 368.1684, found 368.1691.

(*S*,*E*)-5-(3,4-Dimethylstyryl)-3-propyl-2,3-dihydrobenzo[*f*][1,2]thiazepine 1,1-dioxide (6g).

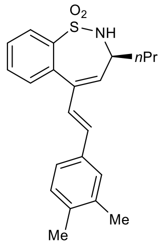

White ointment, 62.4 mg, yield: 85%. ^{1}H NMR (400 MHz, $CDCl_3$) δ 8.10-8.03 (m, 1H), 7.70-7.62 (m, 1H), 7.59-7.50 (m, 2H), 7.20-7.06 (m, 3H), 6.97 (d, *J* = 16.0 Hz, 1H), 6.48 (d, *J* = 16.0 Hz, 1H), 5.92 (d, *J* = 6.8 Hz, 1H), 3.61-3.54 (m, 1H), 2.25 (s, 3H), 2.25 (s, 3H), 1.74-1.64 (m, 2H), 1.39-1.31 (m, 2H), 0.93 (t, *J* = 7.2 Hz, 3H). ^{13}C NMR (100 MHz, $CDCl_3$) δ 144.5, 138.8, 136.8, 136.7, 136.1, 134.3, 133.2, 132.2, 130.3, 129.9, 128.8, 128.6, 128.1, 127.5, 126.4, 124.2, 52.9, 37.3, 19.8, 19.6, 19.0, 13.8. HPLC [Enantiocol Chiral OD, hexane/*i*-PrOH = 90/10, 210 nm, 1.0 mL/min. t_{R1} = 13.0 min (major), t_{R2} = 16.3 min (minor)]; ee = 82%, $[\alpha]^{25}_{D}$ = -25.4 (c = 0.17, $CHCl_3$); HRMS (ESI) calcd for $C_{22}H_{26}NO_2S$ $(M+H)^+$ 368.1684, found 368.1701.

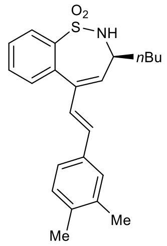

(*S*,*E*)-3-Butyl-5-(3,4-dimethylstyryl)-2,3-dihydrobenzo[*f*][1,2]thiazepine 1,1-dioxide (6h).

White ointment, 61.7 mg, yield: 81%. ^{1}H NMR (400 MHz, $CDCl_3$) δ 8.10-8.02 (m, 1H), 7.69-7.62 (m, 1H), 7.59-7.50 (m, 2H), 7.17 (s, 1H), 7.14 (d, *J* = 8.0 Hz, 1H), 7.08 (d, *J* = 8.0 Hz, 1H), 6.97 (d, *J* =

16.4 Hz, 1H), 6.48 (d, *J* = 16.0 Hz, 1H), 5.92 (d, *J* = 7.2 Hz, 1H), 4.93 (brs, 1H), 3.60-3.52 (m, 1H), 2.25 (s, 3H), 2.25 (s, 3H), 2.15-1.95 (m, 2H), 1.78-1.66 (m, 2H), 1.36-1.30 (m, 2H), 0.90 (t, *J* = 6.8 Hz, 3H). ^{13}C NMR (100 MHz, $CDCl_3$) δ 144.7, 139.0, 138.3, 137.0, 136.3, 134.5, 133.4, 132.4, 130.5, 130.1, 129.1, 128.8, 128.3, 127.7, 126.6, 124.4, 53.3, 35.1, 28.1, 22.7, 20.0, 19.8, 14.1. HPLC [Enantiocol Chiral OD, hexane/*i*-PrOH = 90/10, 210 nm, 1.0 mL/min. t_{R1} = 10.0 min (major), t_{R2} = 12.8 min (minor)]; ee = 87%, $[\alpha]^{25}_D$ = -35.0 (c = 0.88, $CHCl_3$); HRMS (ESI) calcd for $C_{23}H_{28}NO_2S$ $(M+H)^+$ 382.1841, found 382.1833.

(*S*,*E*)-5-(4-Methylstyryl)-3-propyl-2,3-dihydrobenzo[*f*][1,2]thiazepine 1,1-dioxide (6i).

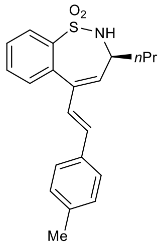

White ointment, 51.5 mg, yield: 73%. ^{1}H NMR (400 MHz, $CDCl_3$) δ 8.05 (d, *J* = 8.0 Hz, 1H), 7.69-7.61 (m, 1H), 7.58-7.51 (m, 2H), 7.27 (d, *J* = 8.0 Hz, 2H), 7.11 (d, *J* = 7.6 Hz, 2H), 6.96 (d, *J* = 16.4 Hz, 1H), 6.49 (d, *J* = 16.4 Hz, 1H), 5.91 (d, *J* = 6.8 Hz, 1H), 4.96 (d, *J* = 1.6 Hz, 1H), 3.61-3.52 (m, 1H), 2.33 (s, 3H), 1.77-1.63 (m, 2H), 1.40-1.30 (m, 2H), 0.91 (t, *J* = 7.6 Hz, 3H). ^{13}C NMR (100 MHz, $CDCl_3$) δ 144.4, 138.8, 138.1, 136.0, 133.8, 133.0, 132.2, 130.3, 129.3, 129.1, 128.6, 127.6, 126.7, 126.4, 52.9, 37.2, 21.3, 19.0, 13.8. HPLC [Enantiocol Chiral OD, hexane/*i*-PrOH = 90/10, 210 nm, 1.0 mL/min. t_{R1} = 13.0 min (major), t_{R2} = 16.4 min (minor)]; ee = 82%, $[\alpha]^{25}_D$ = -28.1 (c = 0.37, $CHCl_3$); HRMS (ESI) calcd for $C_{21}H_{24}NO_2S$ $(M+H)^+$ 354.1528, found 354.1527.

(*S*,*E*)-5-(3-Chlorostyryl)-3-propyl-2,3-dihydrobenzo[*f*][1,2]thiazepine 1,1-dioxide (6j).

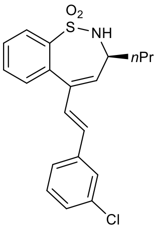

White ointment, 55.9 mg, yield: 75%. ^{1}H NMR (400 MHz, $CDCl_3$) δ 8.08 (dd, *J* = 8.0, 1.2 Hz, 1H), 7.72-7.64 (m, 1H), 7.61-7.52 (m, 2H), 7.41-7.36 (m, 1H), 7.27-7.18 (m, 3H), 7.02 (d, *J* = 16.4 Hz, 1H), 6.46 (d, *J* = 16.4 Hz, 1H), 5.98 (d, *J* = 6.8 Hz, 1H), 4.96 (brs, 1H), 3.62-3.55 (m, 1H), 1.75-1.58 (m, 2H), 1.40-1.30 (m, 2H), 0.93 (t, *J* = 7.2 Hz, 3H). ^{13}C NMR (100 MHz, $CDCl_3$) δ 144.0, 138.9, 138.5, 135.6, 134.6, 132.3, 131.5, 130.6, 130.1, 130.0, 129.8, 128.8, 128.8, 126.6, 126.5, 124.9, 52.8, 37.2, 19.0, 13.8. HPLC [Enantiocol Chiral OD, hexane/*i*-PrOH = 90/10, 210 nm, 1.0 mL/min. t_{R1} = 15.8 min (major), t_{R2} = 19.8 min (minor)]; ee = 93%, $[\alpha]^{25}_D$ = -37.0 (c = 0.47, $CHCl_3$); HRMS (ESI) calcd for $C_{20}H_{20}ClNO_2SNa$ $(M+Na)^+$ 396.0801, found 396.0788.

(*S*,*E*)-3-Propyl-5-(2-(thiophen-2-yl)vinyl)-2,3-dihydrobenzo[*f*][1,2]thiazepine 1,1-dioxide (6k).

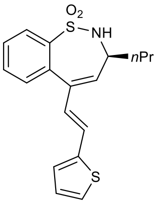

White ointment, 39.3 mg, yield: 57%. ^{1}H NMR (400 MHz, $CDCl_3$) δ 8.06 (dd, *J* = 8.0, 1.2 Hz, 1H), 7.69-7.63 (m, 1H), 7.59-7.52 (m, 2H), 7.19 (d, *J* = 4.8 Hz, 1H), 7.00-6.90 (m, 2H), 6.83 (d, *J* = 16.0 Hz, 1H), 6.62 (d, *J* = 16.0 Hz, 1H), 5.90 (d, *J* = 6.8 Hz, 1H), 4.92 (brs, 1H), 3.58-3.52 (m, 1H), 1.75-1.60 (m, 2H), 1.40-1.30 (m, 2H), 0.91 (t, *J* = 7.6 Hz, 3H). ^{13}C NMR (100 MHz, $CDCl_3$) δ 144.0, 142.1, 138.9, 135.7, 132.3, 130.2, 129.3, 128.8, 128.2, 127.6, 127.1, 126.5, 126.1, 125.2, 52.9, 37.2, 19.0, 13.8. HPLC [Enantiocol Chiral AD, hexane/*i*-PrOH = 90/10, 210 nm, 1.0 mL/min. t_{R1} = 21.1 min (minor), t_{R2} = 39.7 min (major)]; ee = 61%, $[\alpha]^{25}_D$ = -35.8 (c = 0.35, $CHCl_3$); HRMS (ESI) calcd for $C_{18}H_{20}NO_2S_2$ $(M+H)^+$ 346.0935, found 346.0941.

(*S*,*E*)-5-(3,3-Dimethylbut-1-en-1-yl)-3-propyl-2,3-dihydrobenzo[*f*][1,2]thiazepine 1,1-dioxide (6l).

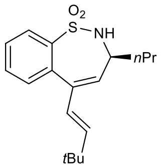

White solid, 45.9 mg, yield: 72%, Mp: 137-138 °C. ^{1}H NMR (400 MHz, $CDCl_3$) δ 8.00 (d, *J* = 7.6 Hz, 1H), 7.60 (t, *J* = 7.6 Hz, 1H), 7.48 (t, *J* = 7.6 Hz, 1H), 7.43 (d, *J* = 7.6 Hz, 1H), 6.18 (d, *J* = 16.0 Hz, 1H), 5.74 (d, *J* = 4.8 Hz, 1H), 5.71 (d, *J* = 16.0 Hz, 1H), 4.97 (brs, 1H), 3.56-3.47 (m, 1H), 1.65-1.50 (m, 2H), 1.36-1.25 (m, 2H), 1.04 (s, 9H), 0.89 (t, *J* = 7.6 Hz, 3H). ^{13}C NMR (100 MHz, $CDCl_3$) δ 146.5,

144.6, 138.9, 136.9, 132.2, 130.3, 128.5, 127.5, 126.5, 125.3, 52.9, 37.5, 33.7, 29.6, 19.2, 14.0. HPLC [Enantiocol Chiral AD, hexane/*i*-PrOH = 90/10, 210 nm, 1.5 mL/min. t_{R1} = 5.1 min (minor), t_{R2} = 8.4 min (major)]; ee = 92%, $[\alpha]^{25}_D$ = +81.2 (c = 0.56, $CHCl_3$); HRMS (ESI) calcd for $C_{18}H_{26}NO_2S$ $(M+H)^+$ 320.1684, found 320.1689.

(*S,E*)-3-Butyl-5-(3,3-dimethylbut-1-en-1-yl)-2,3-dihydrobenzo[*f*][1,2]thiazepine 1,1-dioxide (6m).

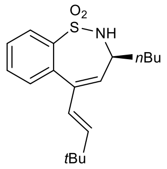

White ointment, 53.9 mg, yield: 81%. ^{1}H NMR (400 MHz, $CDCl_3$) δ 8.06-7.96 (m, 1H), 7.64-7.58 (m, 1H), 7.52-7.47 (m, 1H), 7.43 (dd, *J* = 7.6, 0.8 Hz, 1H), 6.19 (d, *J* = 16.0 Hz, 1H), 5.74 (d, *J* = 6.8 Hz, 1H), 5.71 (d, *J* = 15.6 Hz, 1H), 4.90 (d, *J* = 2.4 Hz, 1H), 3.53-3.45 (m, 1H), 1.72-1.64 (m, 1H), 1.64-1.52 (m, 2H), 1.31-1.24 (m, 3H), 1.04 (s, 9H), 0.87 (t, *J* = 6.8 Hz, 3H). ^{13}C NMR (100 MHz, $CDCl_3$) δ 146.3, 144.4, 138.7, 136.6, 132.0, 130.1, 128.3, 127.2, 126.3, 125.0, 53.0, 34.9, 33.5, 29.4, 27.9, 22.5, 13.9. HPLC [Enantiocol Chiral AD, hexane/*i*-PrOH = 90/10, 210 nm, 1.0 mL/min. t_{R1} = 7.7 min (minor), t_{R2} = 12.4 min (major)]; ee = 95%, $[\alpha]^{25}_D$ = -97.5 (c = 0.21, $CHCl_3$); HRMS (ESI) calcd for $C_{19}H_{28}NO_2S$ $(M+H)^+$ 334.1841, found 334.1858.

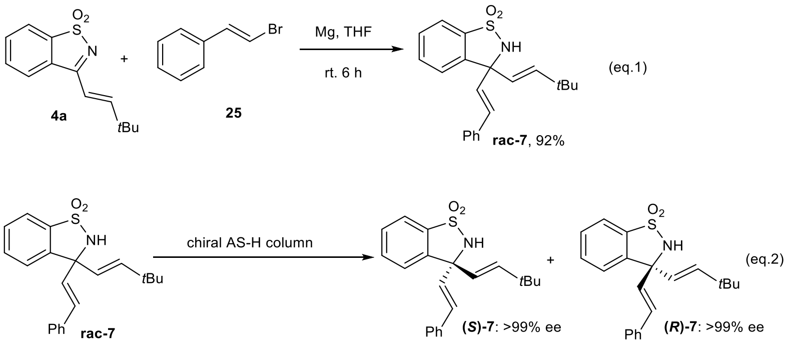

Supplementary Figure 6: Preparation of compound **7**

To a solution of Mg chips (48 mg, 2 mmol) in freshly distilled THF (4 mL) at room temperature was added (*E*)-(2-bromovinyl)benzene **25** (273 mg, 191 μl 1.5 mmol) dropwise for 10 min. After stirring at room temperature for 2 h, the solution changed to brown and most of the Mg chips disappeared. To another dried 25 ml flask was added **4a** (249 mg, 1 mmol) under N_2 and 4 ml THF. Then the above Grignard reagent was added to the flask dropwise at 0 °C over 10 min. The mixture was stirred at room temperature for 6 h. Then the reaction was quenched carefully with saturated NH_4Cl (10 mL) and extracted with EtOAc two times (20 ml each time). The organic solution was concentrated in vacuo. Purification of the residue by column chromatography (petroleum ether/EtOAc = 4/1) gave the product **rac-7** (325 mg, 92%) as a colorless oil. ^{1}H NMR (400 MHz $CDCl_3$) δ 7.78 (d, *J* = 8.0 Hz, 1H), 7.69-7.62 (m, 1H), 7.58-7.51 (m, 1H), 7.42-7.36 (m, 3H), 7.34-7.29 (m, 2H), 7.28-7.23 (m, 1H), 6.76 (d, *J* = 16.0 Hz, 1H), 6.36 (d, *J* = 16.0 Hz, 1H), 5.95 (d, *J* = 16.0 Hz, 1H), 5.65 (d, *J* = 15.6 Hz, 1H), 4.80 (brs, 1H), 1.04 (s, 9H); ^{13}C NMR (100 MHz $CDCl_3$) δ 145.9, 142.8, 136.0, 135.0, 133.6, 132.0, 129.8, 129.7, 128.9, 128.5, 127.1, 125.8, 125.1, 121.7, 67.5, 33.5, 29.6. HRMS (ESI) calcd for $C_{21}H_{24}NO_2S$ $(M+H)^+$ 354.1528, found 354.1540. Separation of **rac-7** using a Daicel Chiral AS-H column: hexane/*i*-PrOH = 90/10, 210 nm, 1.0 mL/min. t_{R1} = 13.0 min, t_{R2} = 21.5 min.

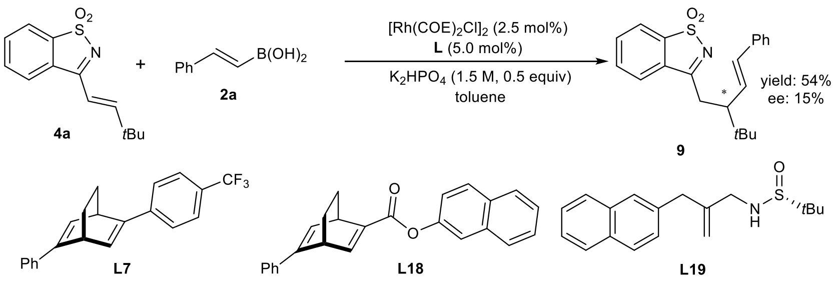

Supplementary Figure 7: Rh-catalyzed asymmetric 1,4-addition of alkenylboronic acid using the same substrate

A similar procedure was used as Xu's report.[14] Under N_2 atmosphere, a solution of substrate α,β-unsaturated ketimine **4a** (0.20 mmol), $[Rh(COE)_2Cl]_2$ (2.5 mol %, 3.6 mg, 0.01 mmol of Rh), ligand **L** (5.0 mol %, 0.01 mmol), and alkenylboronic acid **2a** (0.30 mmol) in 1.0 mL of toluene was stirred at room temperature for 30 min. To this mixture was added aqueous K_2HPO_4 (66 µL, 1.5 M, 0.10 mmol) and then the resulting mixture was stirred at room temperature for 12 hours. The solvent was removed under reduced pressure and the residue was purified by column chromatography (petroleum ether/THF = 4/1) to give the product **9** as a wax.

L7: (38.1 mg, 54%) ^{1}H NMR (500 MHz $CDCl_3$) δ 7.89-7.84 (m, 1H), 7.72-7.66 (m, 3H), 7.27-7.21 (m, 4H), 7.20-7.15 (m, 1H), 6.32 (d, *J* = 16.0 Hz, 1H), 6.03 (dd, *J* = 16.0, 9.5 Hz, 1H), 3.21 (dd, *J* = 14.5, 3.5 Hz, 1H), 3.00 (dd, *J* = 14.5, 10.5 Hz, 1H), 2.76 (dt, *J* = 10.0, 3.5 Hz, 1H), 1.09 (s, 9H); ^{13}C NMR (100 MHz $CDCl_3$) δ 176.3, 140.0, 137.0, 133.6, 133.4, 133.3, 131.7, 129.0, 128.4, 127.3, 126.2, 124.0, 122.5, 51.8, 34.0, 31.7, 27.7. HPLC [Enantiocol Chiral IC-3, hexane/*i*-PrOH = 90/10, 210 nm, 1.0 mL/min. t_{R1} = 51.0 min (major), t_{R2} = 58.7 min (minor)]; ee = 15%, $[\alpha]^{25}_D$ = -10.4 (c = 0.25, $CHCl_3$); HRMS (ESI) calcd for $C_{21}H_{24}NO_2S$ $(M+H)^+$ 354.1528, found 354.1528.
L18: (19.8 mg, 28%), **L19**: <5%.

Supplementary Note 2

Preliminary DFT computational study of racemization processes

All computations were carried out using the WB97XD method, as implemented in the Gaussian 09 software package.[15] All atoms were modeled at the 6-31G(d,p) level of theory. Geometry optimizations were performed with the account of the solvent effects (SMD, 2,2,2-Trifluoroethanol) without applying any geometry Constraints (C1 symmetry).

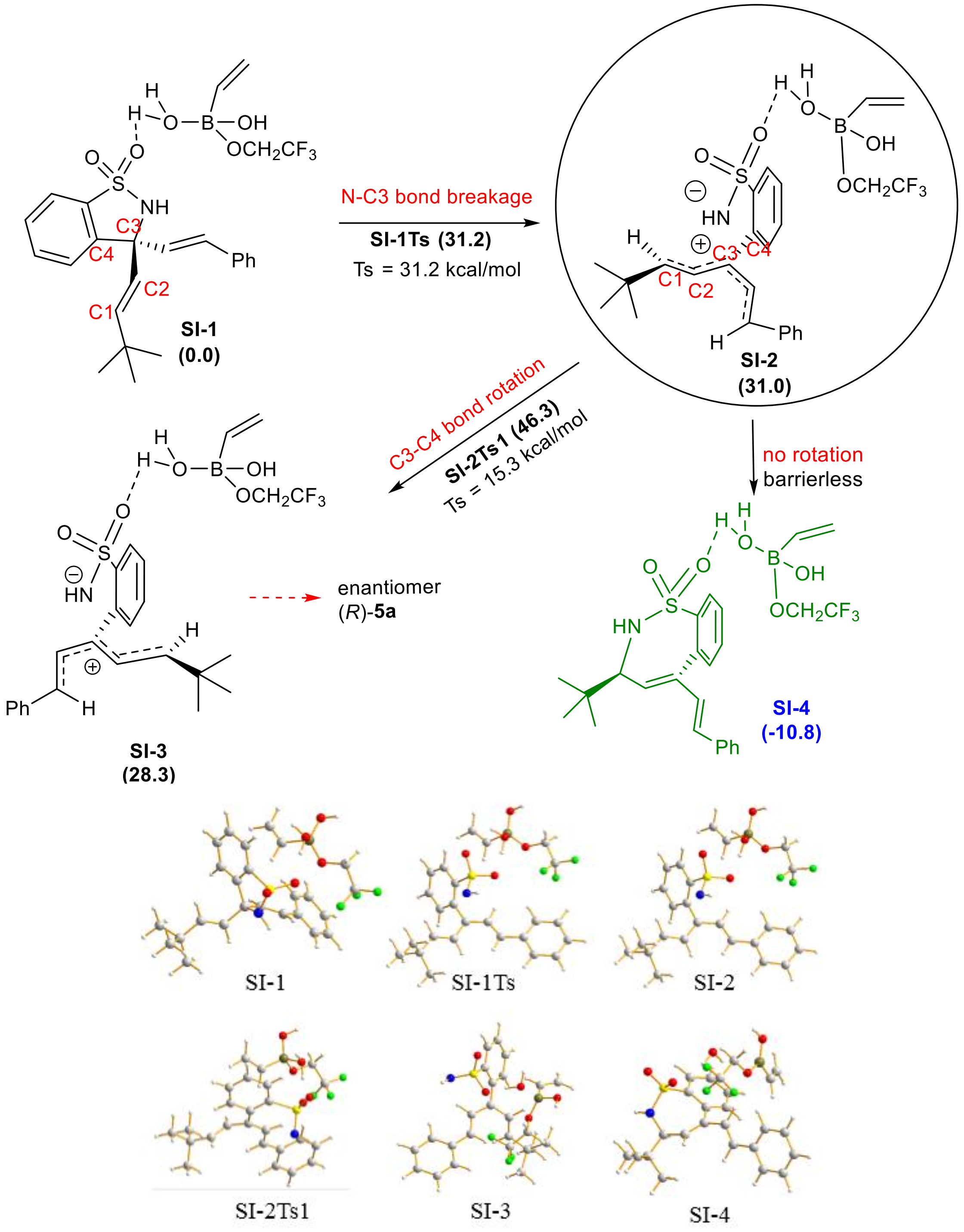

Supplementary Figure 8: Comparation of C3-C4 bond rotation and no rotation process. The energies are Gibbs free energy.

Control experiments indicate that the expansion process could be catalyzed by Lewis acid or Brønsted acid. During our calculation process, we found that alkenylboronic acid did not coordinate with the substrate while the alkenylboronic acid/TFE complex could activate the five-membered ring compound via hydrogen bonding. Vinylboronic acid was used instead of styrylboronic acid to simplify the calculation. After the formation of the carbocation intermediate **SI-2**, racemic product may be formed via the rotation of the C1-C2 bond, C2-C3 bond or C3-C4 bond. However, C1-C2 bond and C2-C3 bond are part of the conjugated allyl cation fragment (not single bonds). Therofore the rotation of these two bonds becomes much more difficult. C3-C4 bond rotation could lead to the formation of intermediate **SI-3** through an energy barrier of 15.3 kcal/mol (compared with **SI-2**, not **SI-1**). However, if there is no bond rotation, intermediate **SI-4** could be formed directly via bond formation at C1 and the nitrogen anion, with no energy barrier. The energy difference between no rotation and C3-C4 bond rotation is 15.3 kcal/mol. Based on the Arrhenius formula, the ratio of reaction rates is $2.2*10^{10}$, which means that C3-C4 bond rotation does not occur.

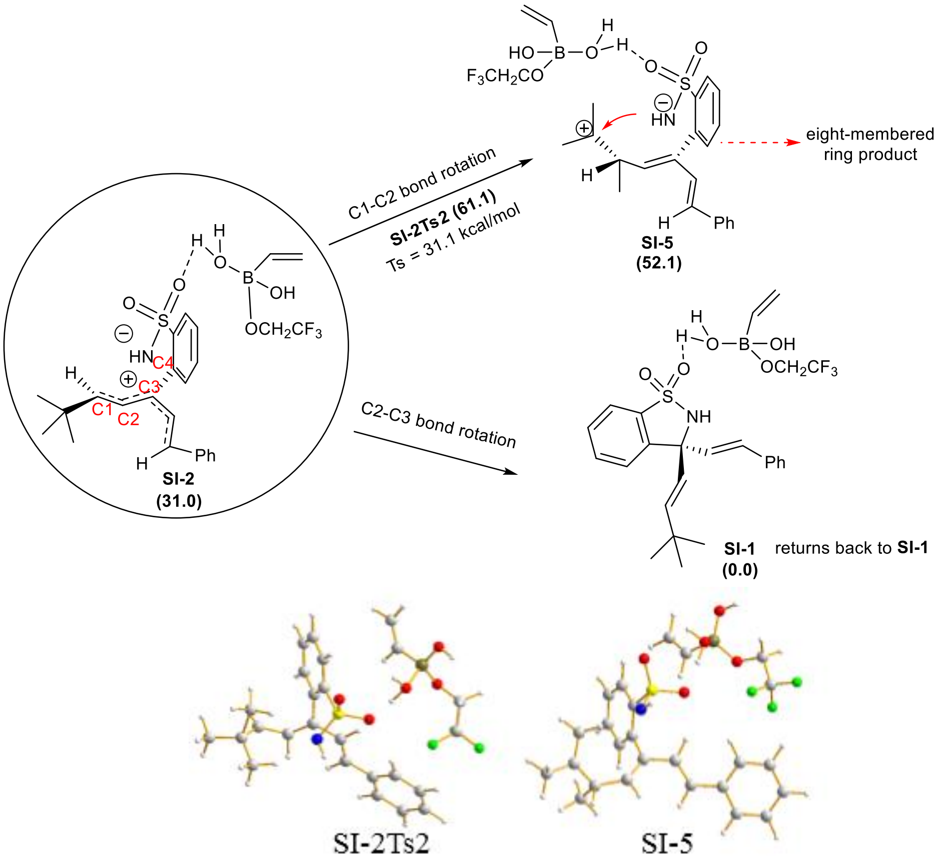

Supplementary Figure 9: Rotation process of C1-C2 bond and C2-C3 bond. The energies are Gibbs free energy.

Althouth the rotation of C1-C2 bond and C2-C3 bond is difficult, we calculated these two processes as well. The results show that C1-C2 bond rotation leads to a methyl migration intermediate **SI-5** through an energy barrier of 31.1 kcal/mol and an eight-membered ring product could be formed. However, the eight-membered ring product was not observed throughout the experiments, indicating that C1-C2 bond rotation is not possible. During the scanning calculation process, C2-C3 bond rotation led to the formation of intermediate **SI-1**, the energy of which is 31.0 kcal/mol lower than **SI-2**. The above results indicate that the ring-expansion step is a stereospecific process.

Supplementary Table 3.

Compound	ZPVE Corrected Energy, a.u.	Free Energy (323 K), a.u.
SI-1	-2123.782599	-2123.851717
SI-1Ts	-2123.731536	-2123.802051
SI-2	-2123.693720	-2123.802253
SI-2TS1	-2123.707092	-2123.777934
SI-3	-2123.736197	-2123.806587
SI-4	-2123.797199	-2123.868942
SI-2TS2	-2123.681426	-2123.754408
SI-5	-2123.699616	-2123.768628

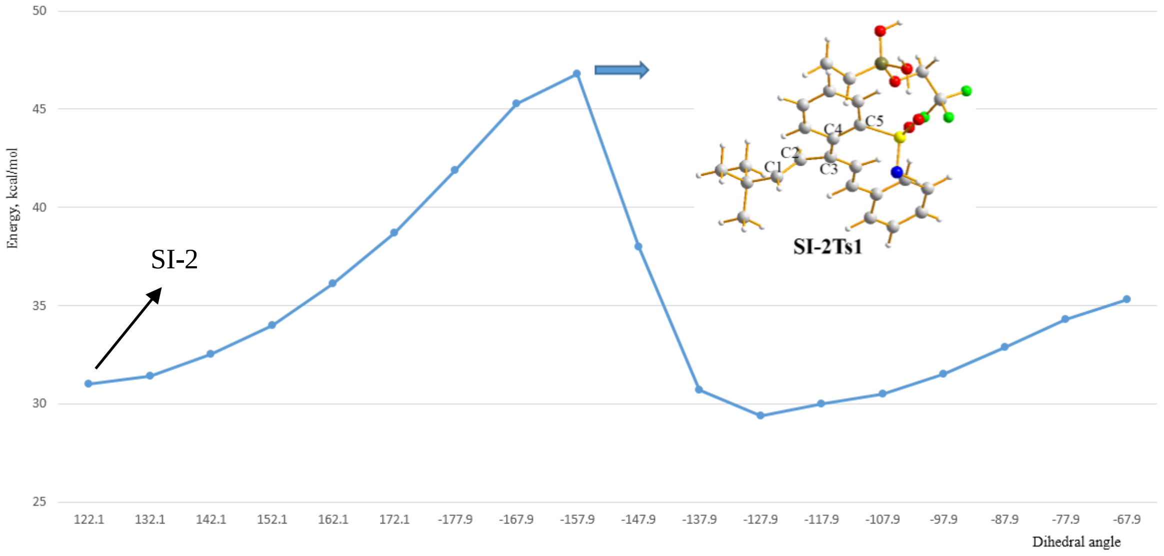

Supplementary Figure 10: Energy scanning of C3-C4 bond rotation. The x axis is the dihedral angle of C5-C4-C3-C2.

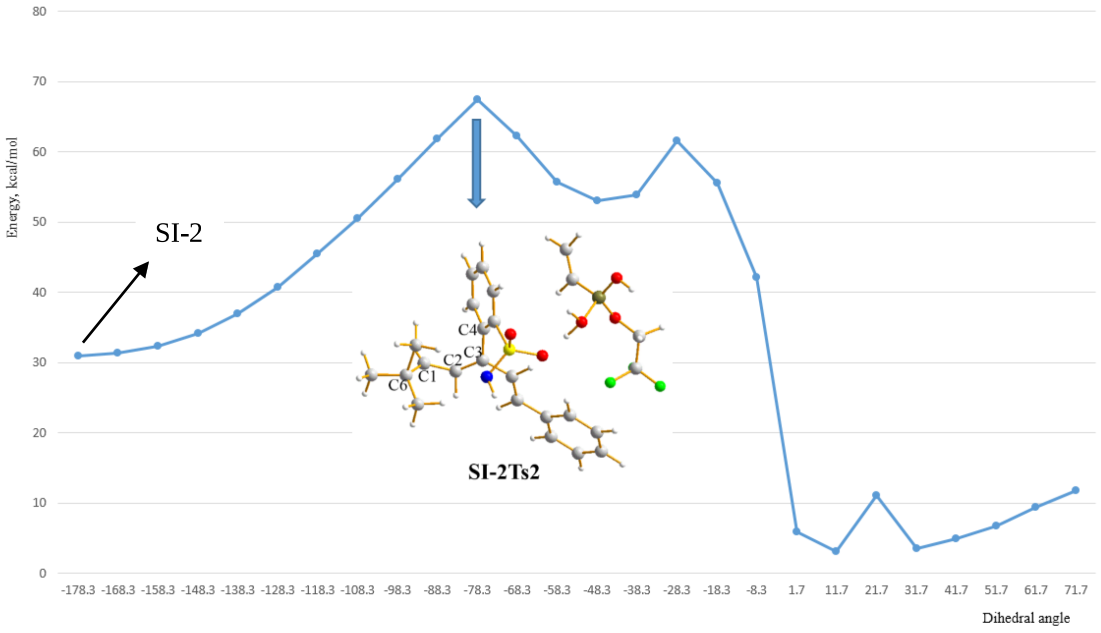

Supplementary Figure 11: Energy scanning of C1-C2 bond rotation. The x axis is the dihedral angle of C3-C2-C1-C6.

Supplementary Note 3
Transformations of alkenylation products

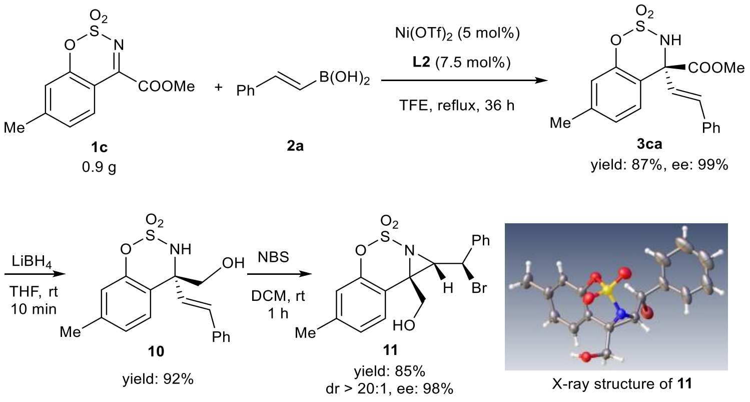

Supplementary Figure 12: Gram scale reaction and transformations of **3ca**

A test tube (100 mL, 25 * 250 mm) was charged with Ni(OTf)$_2$ (63.0 mg, 0.176 mmol, 0.050 equiv), **L2** (142.9 mg, 0.265 mmol, 0.075 equiv) and unpurified TFE (17.6 mL). The solution was stirred at reflux for 20 min, then substrate (900 mg, 3.53 mmol, 1.0 equiv) and alkenylboronic acid (784 mg, 5.29 mmol, 1.5 equiv) were added into the tube. The wall of the tube was rinsed with an additional portion of TFE (17.6 mL). After stirring at reflux for 36 h in air, the reaction mixture was cooled to room temperature and the solvent was removed by rotary evaporation. The residue was purified by column chromatography on silica gel (petroleum ether/EtOAc = 5/1) to give the product **3ca** (1.10 g, 87%).

To a flask (25 ml) was added **3ca** (71.8 mg, 0.20 mmol) and THF (2 ml) under N_2. Then $LiBH_4$ (17.4 mg, 0.80 mmol, 4.0 equiv) was added under N_2. After stirring at room temperature for 10 min, the mixture was quenched with water, extracted with EtOAc, and the organic layer was dried over anhydrous Na_2SO_4 and concentrated under reduced pressure. The product was purified by column chromatography using *n*-hexane/ethyl acetate (2:1) as eluent to give the desired product **10** (60.9 mg, 92%) as an ointment. ^{1}H NMR (400 MHz $CDCl_3$) δ 7.40-7.22 (m, 5H), 7.18 (d, *J* = 8.0 Hz, 1H), 7.09 (d, *J* = 8.0 Hz, 1H), 6.93 (s, 1H), 6.61 (d, *J* = 16.4 Hz, 1H), 6.38 (d, *J* = 16.0 Hz, 1H), 4.14 (d, *J* = 12.0 Hz, 1H), 3.83 (d, *J* = 12.0 Hz, 1H), 2.38 (s, 3H); ^{13}C NMR (100 MHz $CDCl_3$) δ 151.8, 141.2, 135.8, 134.1, 128.9, 128.7, 128.0, 127.2, 127.1, 126.8, 120.2, 118.6, 68.3, 67.6, 21.2. HRMS (ESI) calcd for $C_{17}H_{18}NO_4S$ $(M+H)^+$ 332.0957, found 332.0948.

To a flask (25 ml) was added **10** (49.7 mg, 0.15 mmol) and DCM (2 ml) under N_2. Then NBS (32.0 mg, 0.18 mmol, 1.2 equiv) was added under N_2. After stirring at room temperature for 1 h, the mixture was concentrated under reduced pressure. The product was purified by column chromatography using *n*-hexane/ethyl acetate (5:1) as eluent to give the desired product **11** (52.1 mg, 85%) as a white solid. Mp = 132-133 °C; ^{1}H NMR (400 MHz $CDCl_3$) δ 7.58-7.53 (m, 2H), 7.52 (d, *J* = 8.0 Hz, 1H), 7.45-7.35 (m, 3H), 7.24 (d, *J* = 8.0 Hz, 1H), 7.07 (s, 1H), 5.09 (d, *J* = 11.6 Hz, 1H), 4.28 (d, *J* = 12.4 Hz, 1H), 4.22-4.13 (m, 2H), 2.47 (s, 3H); ^{13}C NMR (100 MHz $CDCl_3$) δ 149.9, 142.3, 137.5, 129.6, 128.8, 128.5, 127.9, 127.9, 120.0, 113.4, 62.7, 56.2, 52.3, 45.7, 21.2. HPLC [Enantiocol Chiral AD, hexane/*i*-PrOH = 90/10, 210 nm, 1.0 mL/min. t_{R1} = 14.5 min (major), t_{R2} = 15.8 min (minor)]; ee = 98%, $[\alpha]^{25}_D$ = -16.7 (c = 0.66, $CHCl_3$); HRMS (ESI) calcd for $C_{17}H_{16}BrNNaO_4S$ $(M+Na)^+$ 431.9881, found 431.9893.[16]

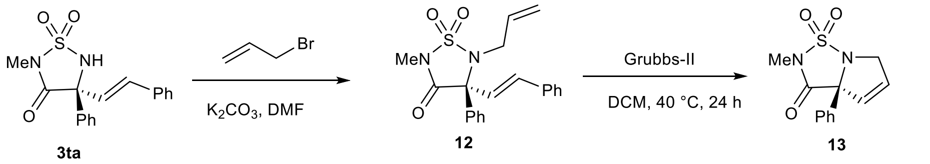

Supplementary Figure 13: Transformations of **3ta** and **3aa**

To a mixture of **3ta** (210 mg, 0.64 mmol) and K_2CO_3 (106 mg, 0.77 mmol) in DMF (7 mL) was added allyl bromide (155 mg, 1.28 mmol). The mixture was stirred at room temperature overnight and diluted with water and the mixture was extracted with ethyl acetate (3 x 20 mL). The combined organic phase was washed with brine and dried over Na_2SO_4. After filtration, concentration of the solvent *in vacuo* afforded a residue, which was purified by flash chromatography on silica gel, eluting with ethyl acetate/petroleum ether 5:1 (v/v), to afford the product **12** (180 mg, 78% yield). as a white solid. Mp = 140-141 °C; ^{1}H NMR (400 MHz, $CDCl_3$) δ 7.53-7.43 (m, 7H), 7.43-7.36 (m, 3H), 6.93 (d, *J* = 16.0 Hz, 1H), 6.59 (d, *J* = 16.0 Hz, 1H), 6.05-5.92 (m, 1H), 5.34-5.23 (m, 2H), 3.68 (ddd, *J* = 36.4, 14.8, 6.4 Hz, 1H), 3.27 (s, 3H); ^{13}C NMR (100 MHz, $CDCl_3$) δ 166.1, 135.5, 135.1, 135.0, 131.7, 129.6, 129.1, 128.9, 128.8, 128.0, 127.1, 123.8, 120.3, 76.3, 46.2, 25.4. HPLC [Enantiopak Chiralpak AD, hexane/*i*-PrOH = 90/10, 210 nm, 0.8 mL/min. t_{R1} = 8.3 min (minor), t_{R2} = 14.2 min (major)]; ee = 86%, $[\alpha]^{25}_D$ = +25.6 (c = 0.6, $CHCl_3$); HRMS (ESI) calcd for $C_{20}H_{21}N_2O_3S$ $(M+H)^+$ 369.1273, found 369.1275.

Then, the solution of compound **12** (147.2 mg, 0.4 mmol) and Grubbs-II Catalyst (14.0 mg, 0.02 mmol) in DCM (4 mL) was stirred at 40 ^{o}C for 24 hours. After completion, concentration of the solvent *in vacuo* afforded a residue, which was purified by flash chromatography on silica gel, eluting with ethyl acetate/petroleum ether 10:1 (v/v), to afford the product **13** (107 mg, 92% yield) as a white solid. Mp = 111-112 °C; ^{1}H NMR (400 MHz, $CDCl_3$) δ 7.73-7.67 (m, 2H), 7.47-7.37 (m, 3H), 6.20 (dt, *J* = 6.0, 2.4 Hz, 1H), 6.01 (dt, *J* = 6.0, 2.0 Hz, 1H), 4.91 (dt, *J* = 16.0, 2.4 Hz, 1H), 4.36 (ddd, *J* = 16.0, 2.4, 2.0 Hz, 1H), 3.15 (s, 3H); ^{13}C NMR (100 MHz, $CDCl_3$) δ 167.6, 136.7, 128.9, 128.5, 127.2, 125.7, 85.9, 58.1, 26.1. HPLC [Enantiopak Chiralpak AD, hexane/*i*-PrOH = 90/10, 210 nm, 0.8 mL/min. t_{R1} = 8.5 min (minor), t_{R2} = 9.7 min (major)]; ee = 86%, $[\alpha]^{25}_D$ = -55.1 (c = 0.6, $CHCl_3$); HRMS (ESI) calcd for $C_{12}H_{13}N_2O_3S$ $(M+H)^+$ 265.0647, found 265.0647.[17]

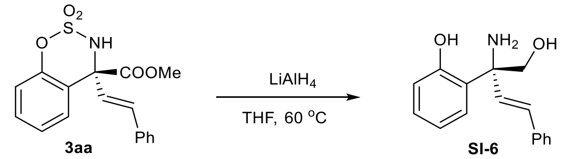

Supplementary Figure 14: Transformations of **3aa**

The reaction was carried out using a modified procedure reported by Lam.[18] To a solution of the alkenylation product **3aa** (137 mg, 0.397 mmol) in THF (2 mL) at room temperature was added $LiAlH_4$ (1.0 M in THF, 1.59 mL, 1.59 mmol) dropwise over 2 min. The mixture was heated at 60 °C overnight, cooled naturally to room temperature, and then to 0 °C with an ice bath. The reaction was quenched carefully with EtOAc (5 mL) followed by EtOH (5 mL). The solution was concentrated in vacuo. Purification of the residue by column chromatography (2:1 petroleum ether:EtOAc →1:1 petroleum ether:EtOAc) gave the product **SI-6** (89.9 mg, 89%) as a white ointment. ^{1}H NMR (400 MHz $CDCl_3$) δ

7.44-7.38 (m, 2H), 7.36-7.30 (m, 2H), 7.29-7.24 (m, 1H), 7.22-7.16 (m, 1H), 7.11 (dd, J = 8.0, 1.6 Hz, 1H), 6.88 (dd, J = 8.0, 1.2 Hz, 1H), 6.80 (td, J = 7.6, 1.2 Hz, 1H), 6.58 (d, J = 16.4 Hz, 1H), 6.43 (d, J = 16.4 Hz, 1H), 4.10 (d, J = 11.2 Hz, 1H), 3.79 (d, J = 11.2 Hz, 1H); ^{13}C NMR (100 MHz $CDCl_3$) δ 158.9, 136.4, 131.8, 130.5, 129.8, 128.9, 128.3, 127.5, 126.8, 124.9, 119.2, 118.4, 68.0, 62.3. HPLC [Enantiocol Chiral AY, hexane/*i*-PrOH = 90/10, 210 nm, 0.5 mL/min. t_{R1} = 30.7 min (minor), t_{R2} = 32.6 min (major)]; ee = 97%, $[\alpha]^{25}_D$ = -23.5 (c = 0.44, $CHCl_3$); HRMS (ESI) calcd for $C_{16}H_{18}O_2$ $(M+H)^+$ 256.1338, found 256.1333.

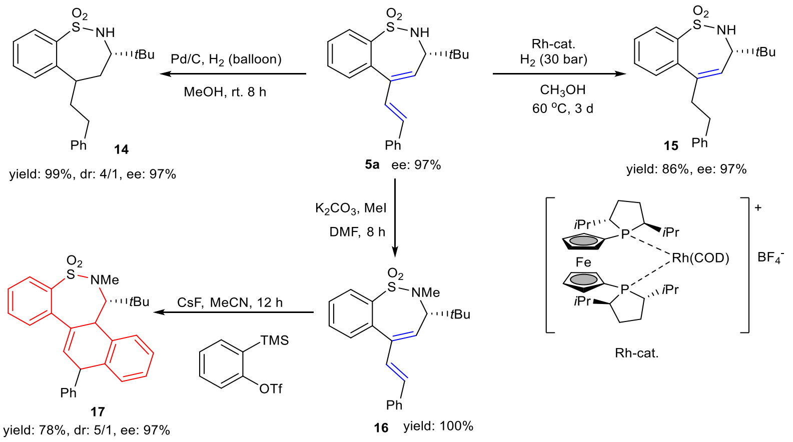

Supplementary Figure 15: Transformations of **5a**

Eq. 1: To a 10 ml flask was added **5a** (35.7 mg, 0.1 mmol), MeOH (2 ml) and Pd/C (20 mg). The flask was equipped with a H_2 balloon. After stirring at room temperature for 8 h, Pd/C was filtered and MeOH was evaporated to obtain the desired product **14** (35.2 mg, 99%) as a colorless oil. ^{1}H NMR (400 MHz $CDCl_3$) δ 8.04-7.95 (m, 1H), 7.54-7.44 (m, 1H), 7.39 (d, J = 7.6 Hz, 1H), 7.36-7.28 (m, 3H), 7.23-7.20 (m, 2H), 7.19-7.15 (m, 1H), 4.00 (d, J = 10.0 Hz, 0.8H), 3.90 (d, J = 10.0 Hz, 0.2H), 3.84-3.70 (m, 1H), 3.66-3.56 (m, 1H), 2.85-2.65 (m, 2H), 2.35-2.25 (m, 1H), 2.15 (d, J = 14.4 Hz, 1H), 2.03-1.94 (m, 1H), 1.35-1.29 (m, 1H), 1.00 (s, 9H); ^{13}C NMR (100 MHz $CDCl_3$) δ 138.6, 137.2, 137.1, 137.0, 128.1, 124.0, 123.9, 123.8, 123.8, 123.0, 121.8, 121.7, 121.6, 121.5, 61.3, 35.0, 32.3, 29.8, 29.8, 29.8, 29.7, 29.5, 22.2. HPLC {Enantiocol Chiral AD, hexane/*i*-PrOH = 92/8, 254 nm, 0.8 mL/min. major [t_{R1} = 10.8 min (major), t_{R2} = 18.1 min (minor), ee = 97%] minor [t_{R1} = 23.3 min (major), t_{R2} = 26.0 min (minor), ee = 98%]}, $[\alpha]^{25}_D$ = 13.0 (c = 0.83, $CHCl_3$); HRMS (ESI) calcd for $C_{21}H_{28}NO_2S$ $(M+H)^+$ 358.1841, found 358.1858.

Eq. 2: In a nitrogen-filled glovebox, a hydrogenation tube was charged with a stirring bar, **5a** (21.3 mg, 0.06 mmol), [Rh(*i*Pr-Ferphos)(cod)]BF_4 (0.92 mg, 1.2 μmol). MeOH (2 mL) was then injected into the hydrogenation tube by a syringe. The hydrogenation tube was then put into an autoclave. The system was evacuated and filled with hydrogen 3 times. The autoclave was then charged with hydrogen to 30 bar hydrogen pressure, and the reaction mixture was stirred at 60 °C for 3 days before releasing the hydrogen. After the reaction finished, the solvent was evaporated and the residue was purified by preparative TLC on silica gel (petroleum ether/EtOAc = 5/1) to give the product **15** (18.4 mg, 86%) as a colorless oil. ^{1}H NMR (400 MHz $CDCl_3$) δ 7.99 (d, J = 7.6 Hz, 1H), 7.58 (t, J = 7.6 Hz, 1H), 7.44 (t, J = 8.4 Hz, 2H), 7.25-7.13 (m, 5H), 5.83 (d, J = 6.4 Hz, 1H), 4.95 (brs, 1H), 3.19 (d, J = 6.0 Hz, 1H), 2.90-2.65 (m, 4H), 0.92 (s, 9H); ^{13}C NMR (100 MHz $CDCl_3$) δ 145.0, 141.7, 139.0, 138.3, 132.5, 128.3, 128.2, 128.1, 127.5, 126.1, 125.9, 124.4, 60.8, 39.3, 34.4, 33.5, 25.8. HPLC [Enantiocol Chiral AD, hexane/*i*-PrOH = 90/10, 210 nm, 1.0 mL/min. t_{R1} = 10.5 min (major), t_{R2} = 31.6 min (minor)]; ee = 97%, $[\alpha]^{25}_D$ = -33.9 (c = 0.33, $CHCl_3$); HRMS (ESI) calcd for $C_{21}H_{25}NO_2SNa$ $(M+Na)^+$ 378.1504, found 378.1520.

Eq. 3: Synthesis of **16**: To a 10 ml flask was added **5a** (53.0 mg, 0.15 mmol), DMF (2 ml) and K_2CO_3 (41.4 mg, 0.3 mmol) and stirred for 15 min. Then MeI (10.5 μl, 0.165 mmol) was added to the flask with a syringe. The solution was stirred at room temperature for 8 h. Then the reaction was quenched with H_2O (15 mL) and extracted with EtOAc two times (20 ml each time). The organic solution was concentrated in vacuo. Purification of the residue by column chromatography (petroleum ether/EtOAc = 4/1) gave the product **16** (55.0 mg, 100%) as a white solid. Mp.: 154-155 ^oC. ^1H NMR (400 MHz $CDCl_3$) δ 8.01 (dd, *J* = 7.6, 1.6 Hz, 1H), 7.57 (td, *J* = 7.6, 1.2 Hz, 1H), 7.52 (dd, *J* = 7.6, 1.6 Hz, 1H), 7.45-7.38 (m, 3H), 7.34-7.29 (m, 2H), 7.25-7.21 (m, 1H), 6.92 (d, *J* = 16.0 Hz, 1H), 6.49 (d, *J* = 16.0 Hz, 1H), 6.33 (d, *J* = 3.2 Hz, 1H), 3.49 (d, *J* = 3.2 Hz, 1H), 2.74 (s, 3H), 1.12 (s, 9H); ^{13}C NMR (100 MHz $CDCl_3$) δ 139.7, 139.1, 137.1, 134.0, 132.4, 132.2, 131.5, 131.3, 131.2, 128.9, 128.9, 128.0, 127.7, 126.8, 70.6, 41.5, 37.4, 27.3. HRMS (ESI) calcd for $C_{22}H_{26}NO_2S$ $(M+H)^+$ 368.1684, found 368.1677.

Synthesis of **17**: To a 10 ml flask was added **16** (36.7 mg, 0.10 mmol), CsF (91.2 mg, 6.0 equiv) and freshly distilled MeCN (2 ml) and stirred for 5 min. Then 2-(trimethylsilyl)phenyl trifluoromethanesulfonate (72.7 μl, 3.0 equiv) was added to the flask with a syringe. The solution was stirred at room temperature for 12 h. Then the reaction was quenched with H_2O (5 mL) and extracted with EtOAc two times (10 ml each time). The organic solution was concentrated in vacuo. Purification of the residue by preparative TLC on silica gel (petroleum ether/EtOAc = 7/1) to give the product (34.6 mg, 78%) as a white ointment. ^1H NMR (400 MHz $CDCl_3$) δ 7.96 (d, *J* = 7.6, 1.2 Hz, 1H), 7.52-7.41 (m, 5H), 7.41-7.32 (m, 4H), 7.32-7.24 (m, 3H), 6.92 (d, *J* = 6.8 Hz, 1H), 5.08 (d, *J* = 6.8 Hz, 1H), 4.82 (d, *J* = 11.2 Hz, 1H), 2.63 (d, *J* = 11.6 Hz, 1H), 2.43 (s, 3H), 0.77 (s, 9H); ^{13}C NMR (100 MHz $CDCl_3$) δ 143.8, 142.1, 139.6, 138.6, 137.9, 137.8, 133.5, 132.0, 130.9, 130.6, 129.6, 128.4, 128.3, 127.8, 127.5, 126.9, 126.7, 126.3, 77.7, 47.6, 46.4, 42.4, 37.5, 29.0. HPLC [Enantiocol Chiral AD, hexane/*i*-PrOH = 90/10, 210 nm, 1.0 mL/min. t_{R1} = 22.5 min (minor), t_{R2} = 128.5 min (major)]; ee = 97%, $[\alpha]^{25}_D$ = 43.9 (c = 0.25, $CHCl_3$); HRMS (ESI) calcd for $C_{28}H_{29}NO_2SNa$ $(M+Na)^+$ 466.1817, found 466.1821.

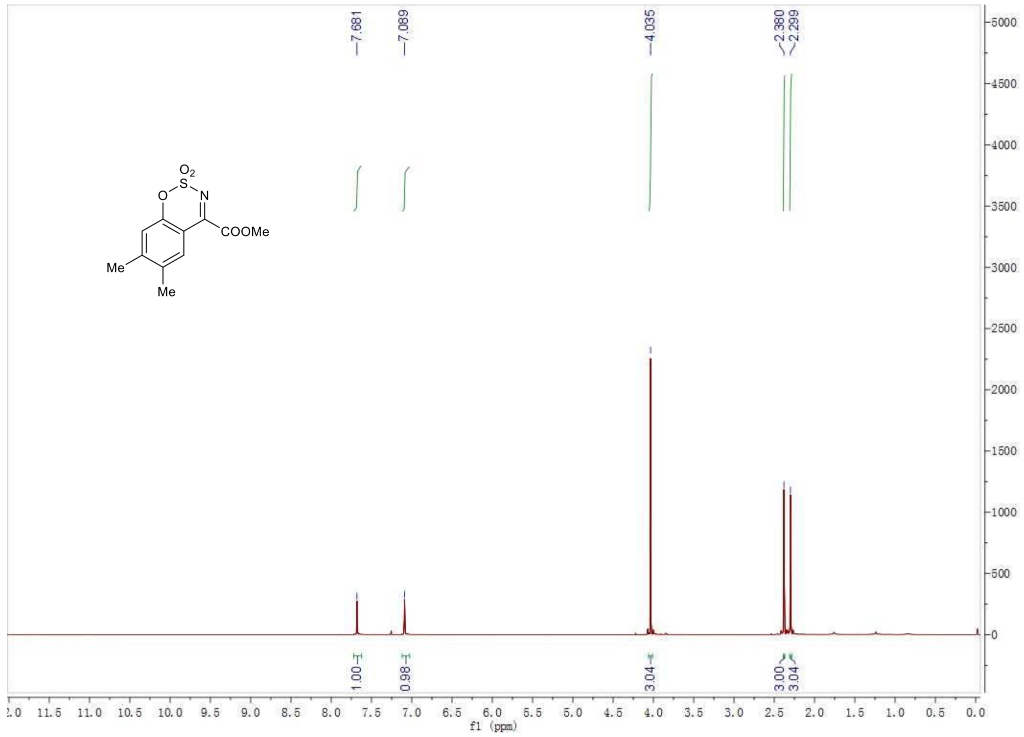

Supplementary Figure 16: ^{1}H NMR spectrum of compound **1d** in $CDCl_3$

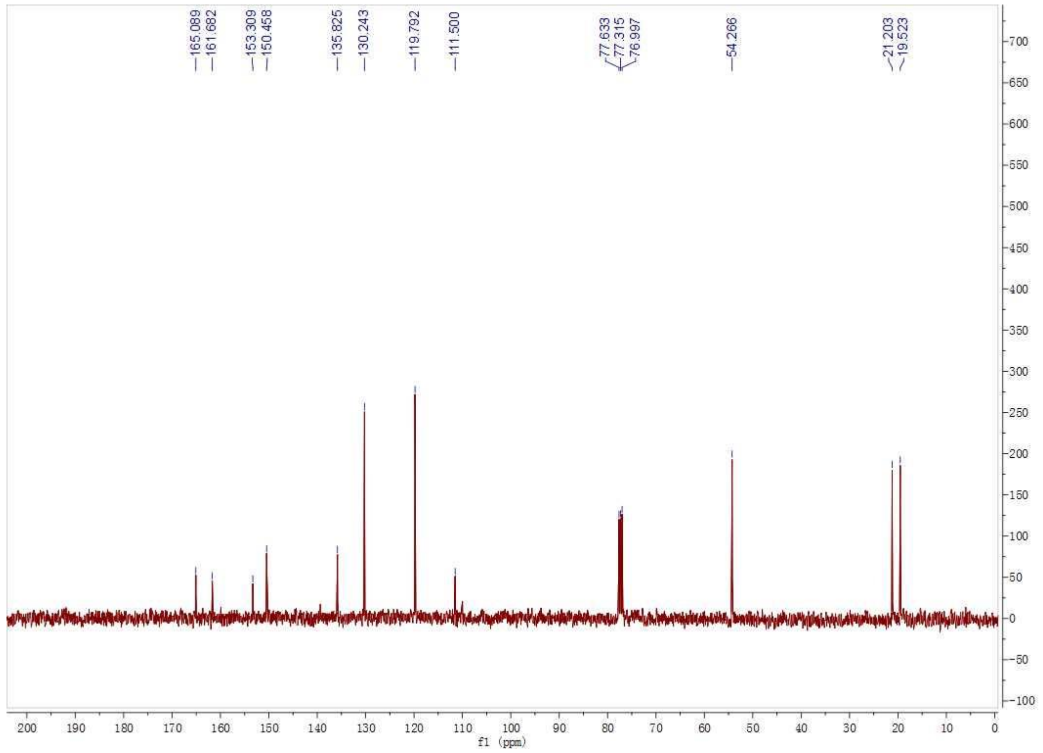

Supplementary Figure 17: ^{13}C NMR spectrum of compound **1d** in $CDCl_3$

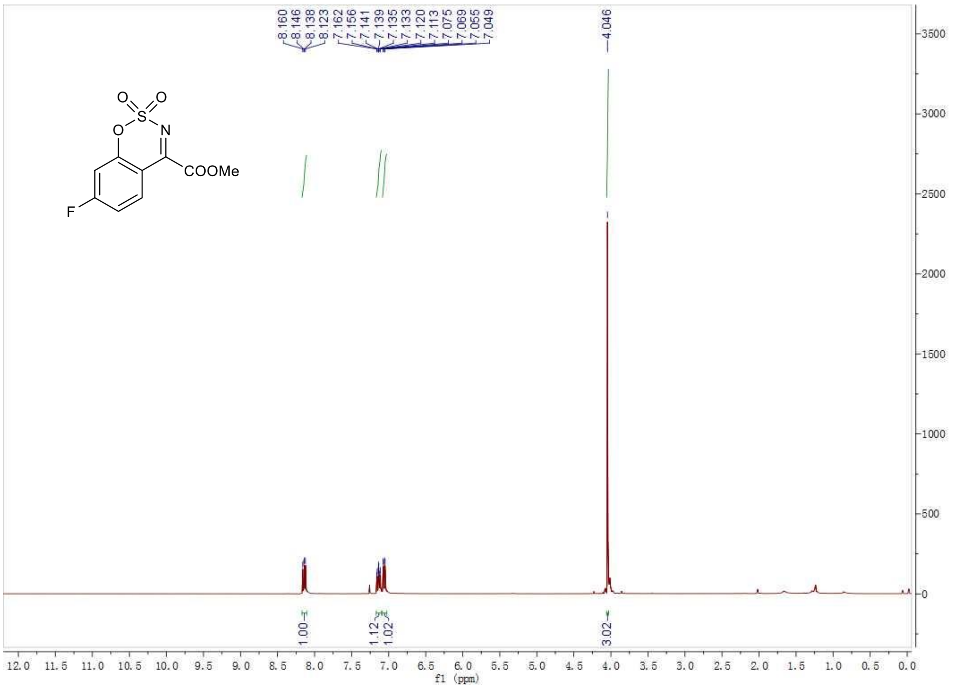

Supplementary Figure 18: ^{1}H NMR spectrum of compound **1f** in $CDCl_3$

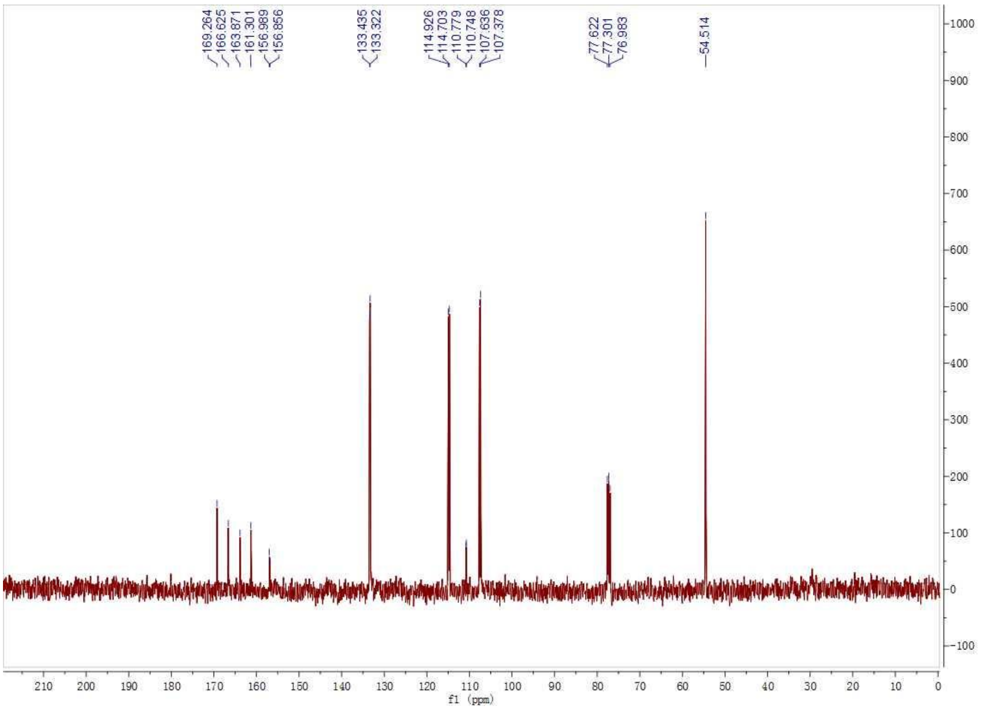

Supplementary Figure 19: ^{13}C NMR spectrum of compound **1f** in $CDCl_3$

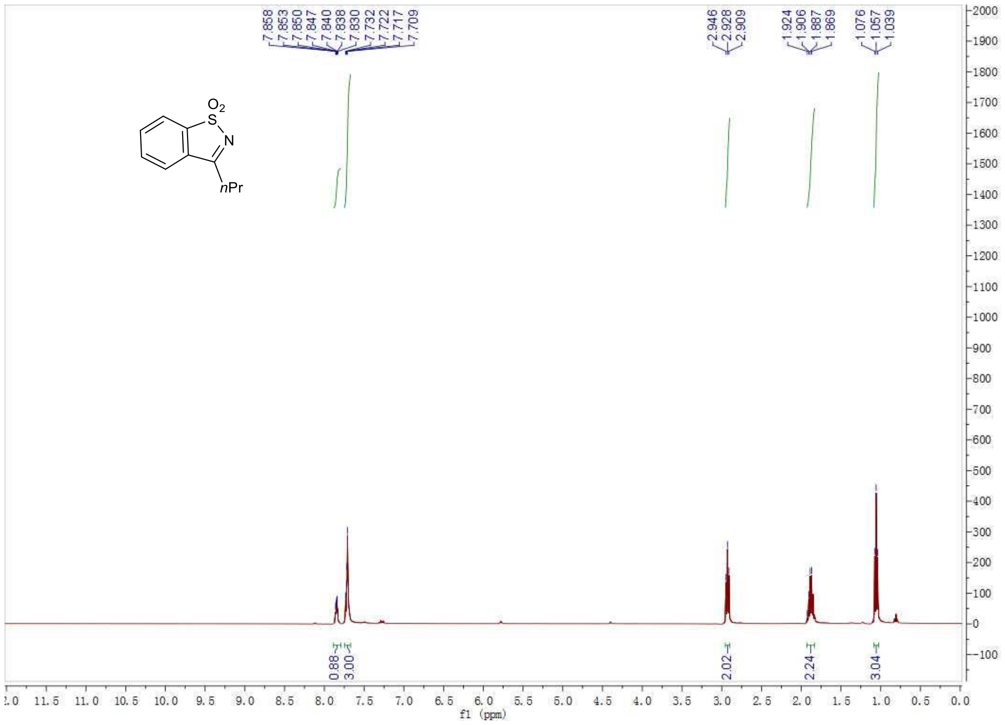

Supplementary Figure 20: ^{1}H NMR spectrum of compound **1k** in $CDCl_3$

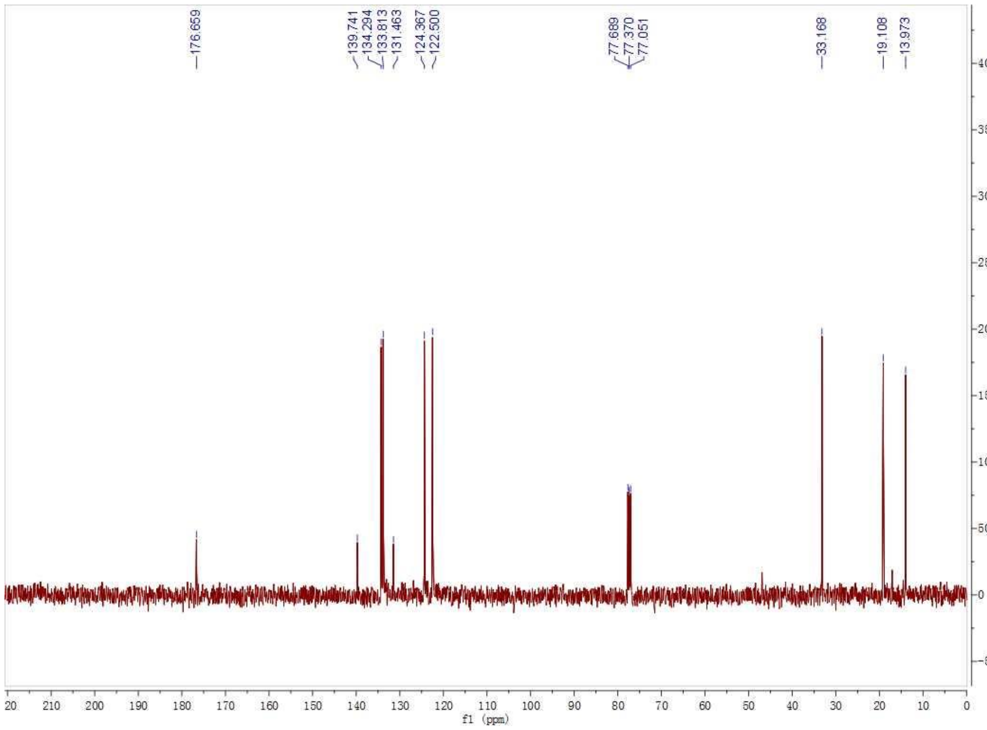

Supplementary Figure 21: ^{13}C NMR spectrum of compound **1k** in $CDCl_3$

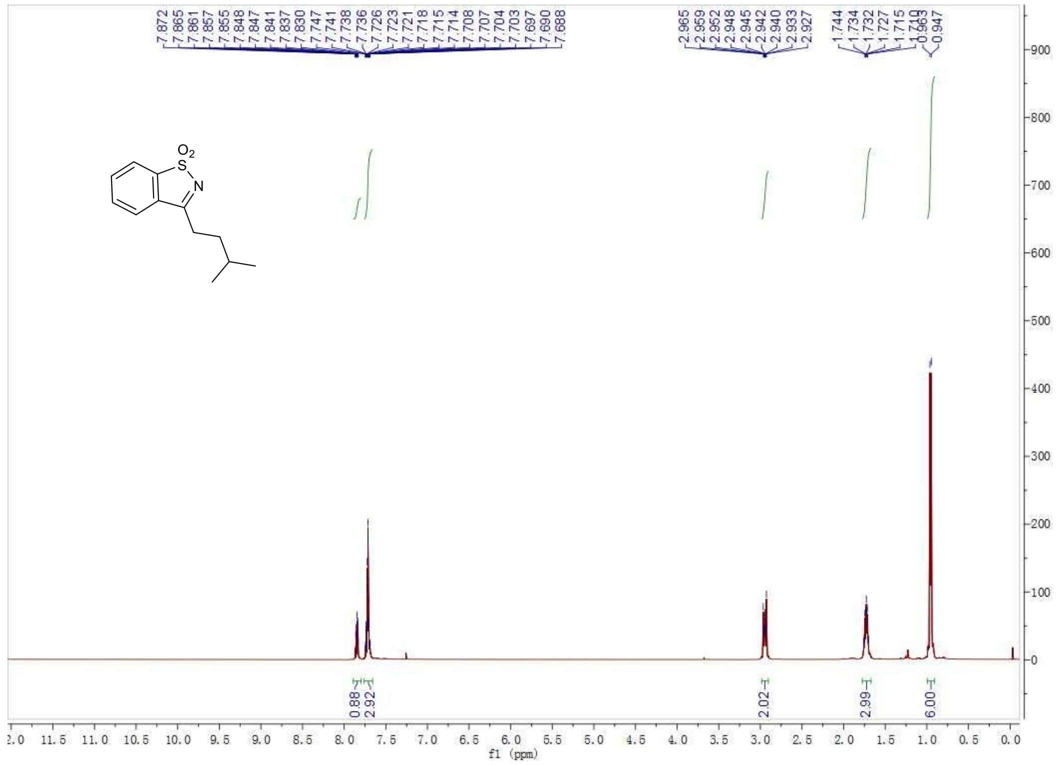

Supplementary Figure 22: ^{1}H NMR spectrum of compound **1p** in $CDCl_3$

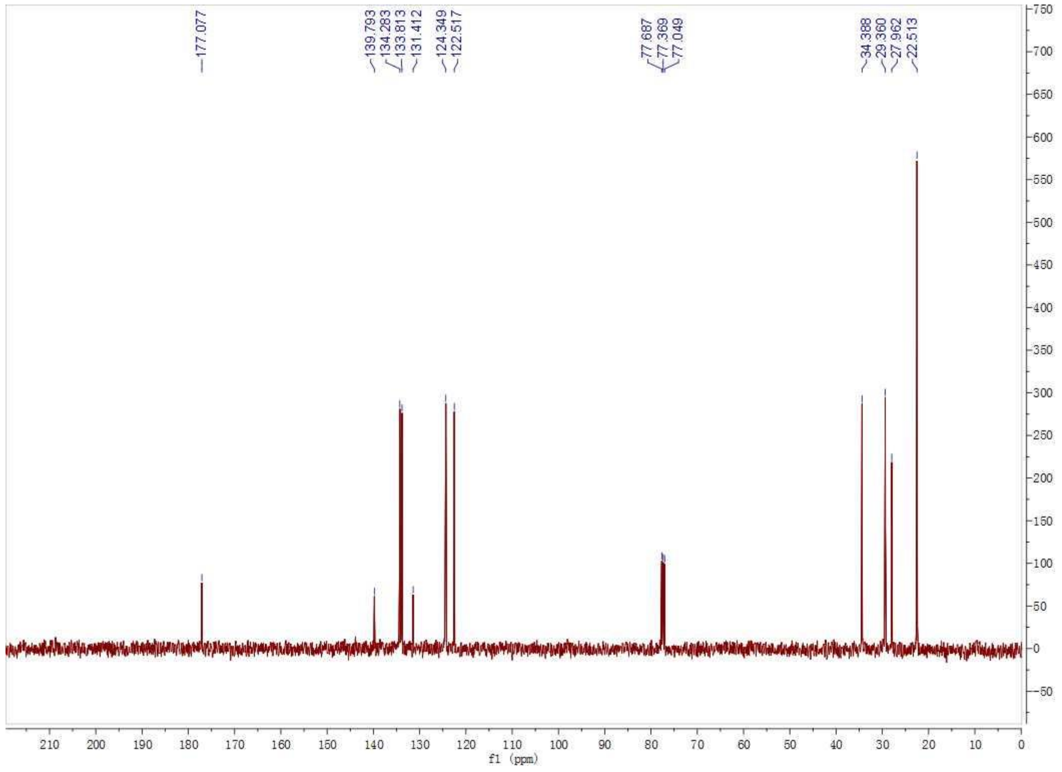

Supplementary Figure 23: ^{13}C NMR spectrum of compound **1p** in $CDCl_3$

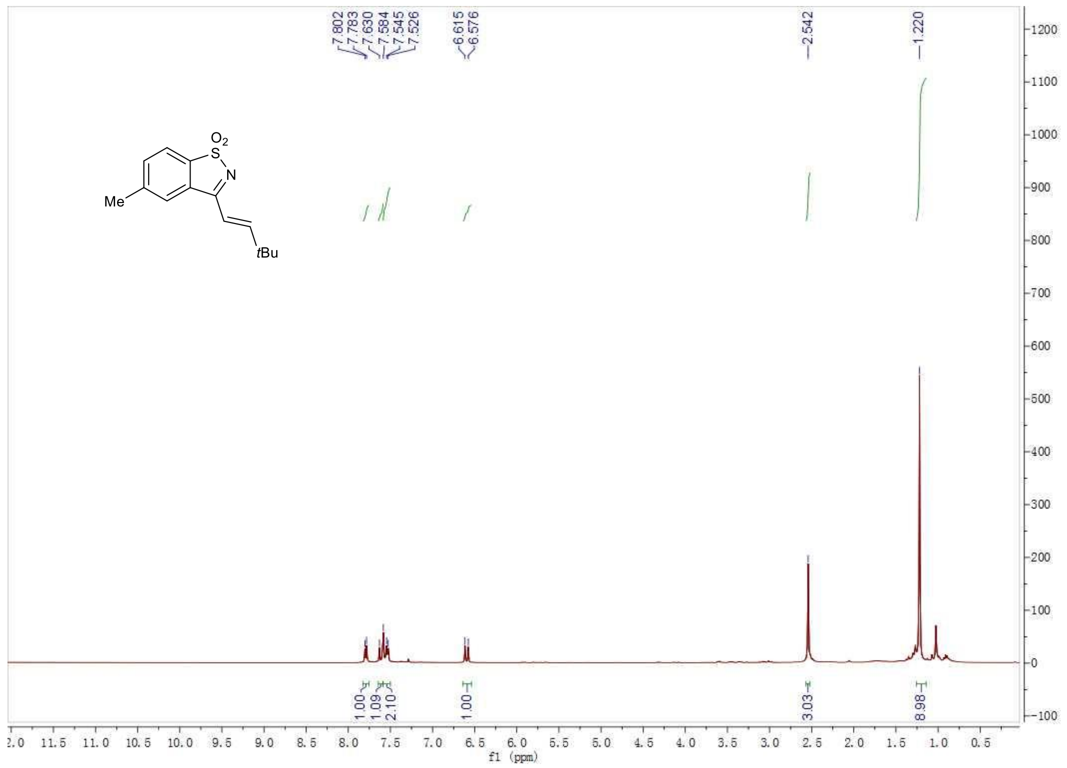

Supplementary Figure 24: ^{1}H NMR spectrum of compound **4b** in $CDCl_3$

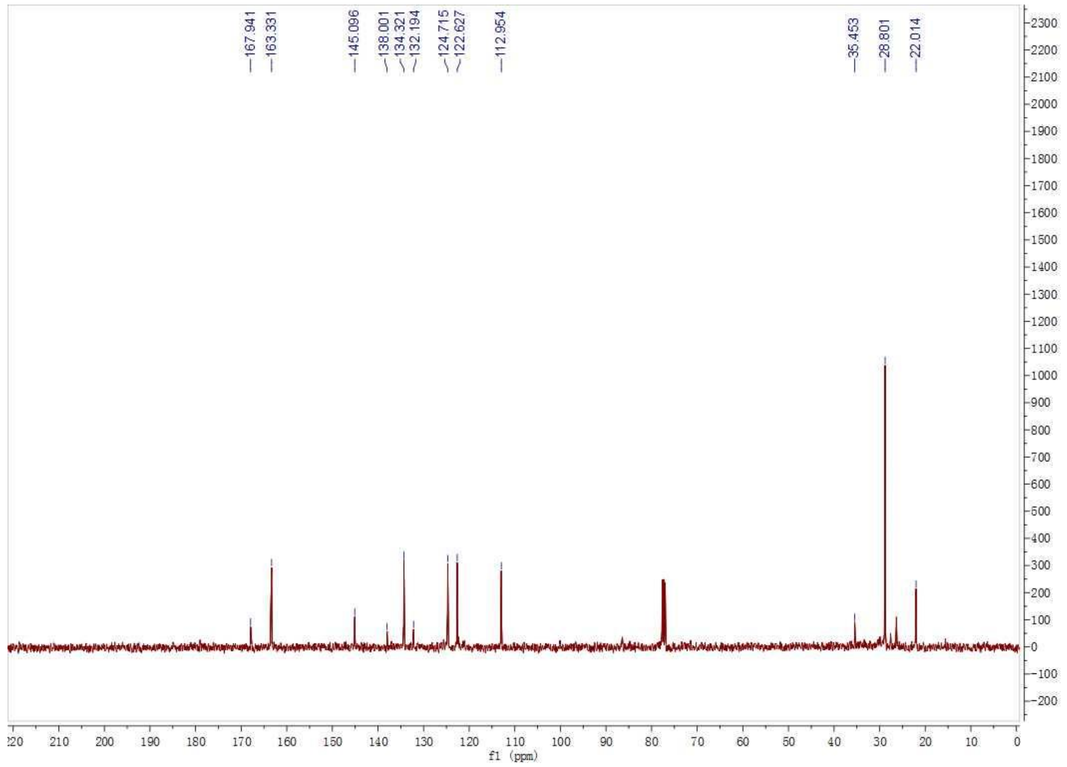

Supplementary Figure 25: ^{13}C NMR spectrum of compound **4b** in $CDCl_3$

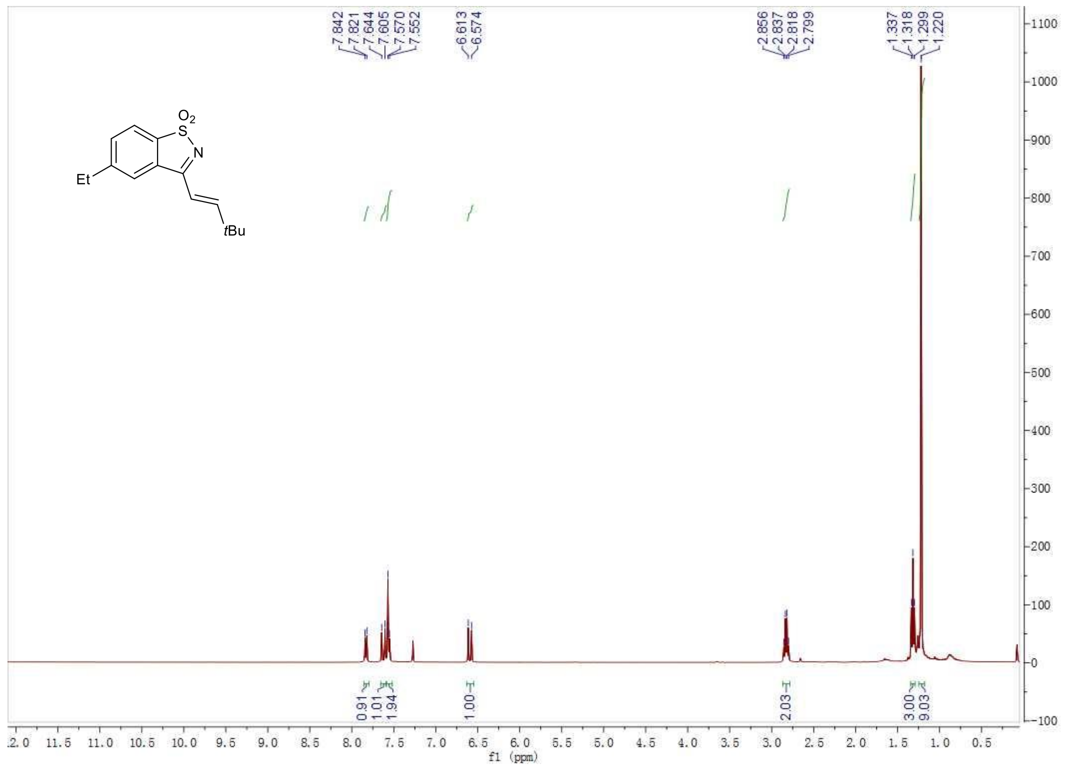

Supplementary Figure 26: ^{1}H NMR spectrum of compound **4c** in $CDCl_3$

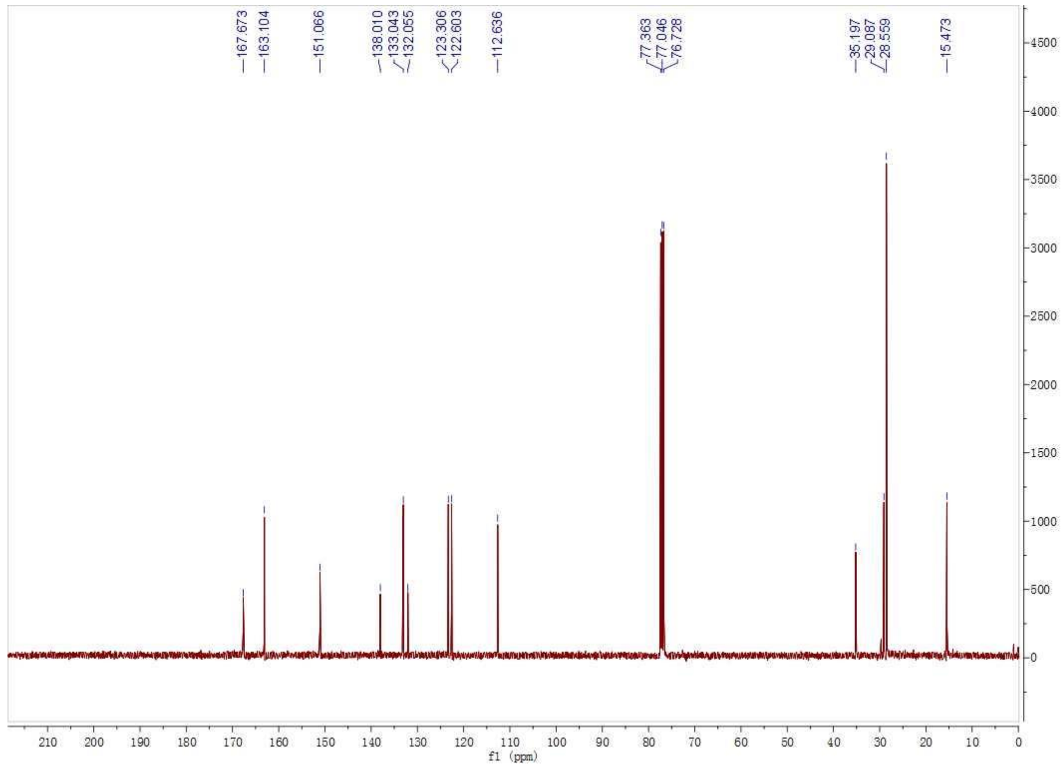

Supplementary Figure 27: ^{13}C NMR spectrum of compound **4c** in $CDCl_3$

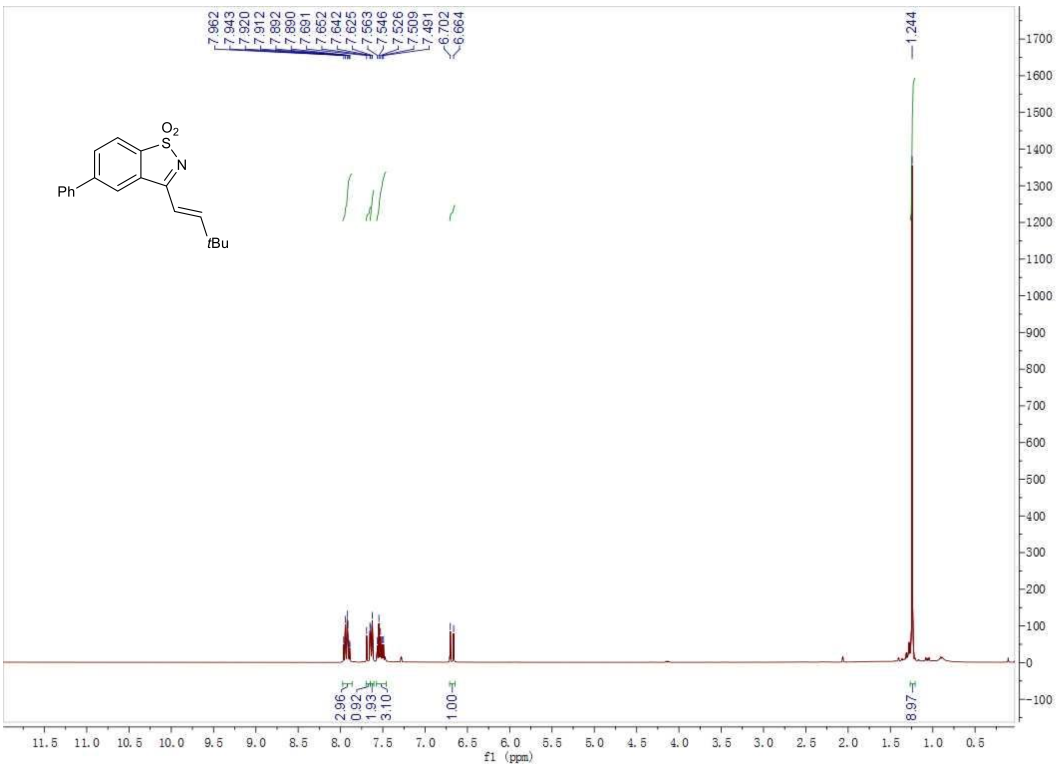

Supplementary Figure 28: ^{1}H NMR spectrum of compound **4d** in $CDCl_3$

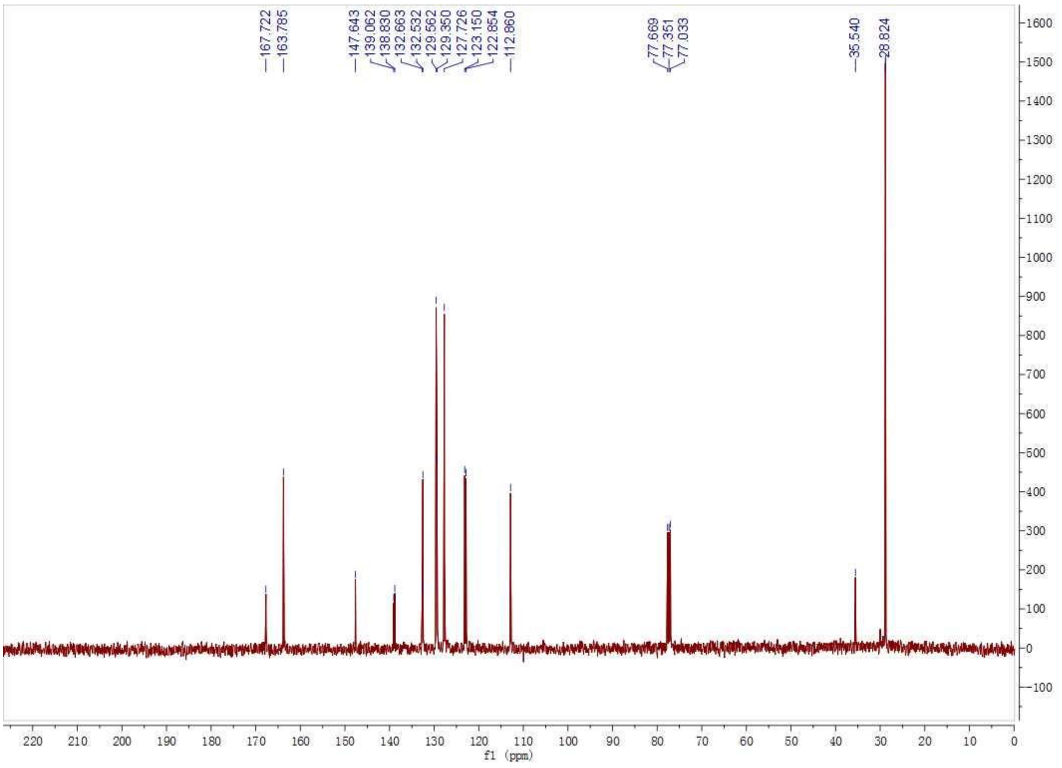

Supplementary Figure 29: ^{13}C NMR spectrum of compound **4d** in $CDCl_3$

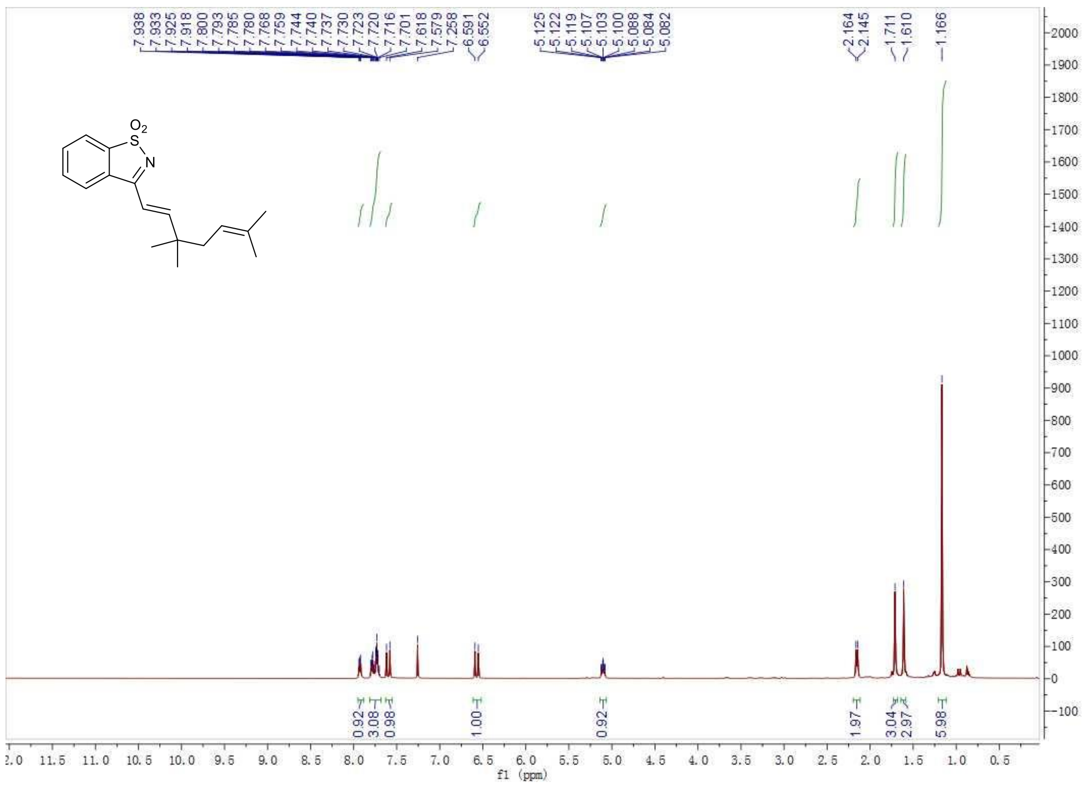

Supplementary Figure 30: ^{1}H NMR spectrum of compound **4e** in $CDCl_3$

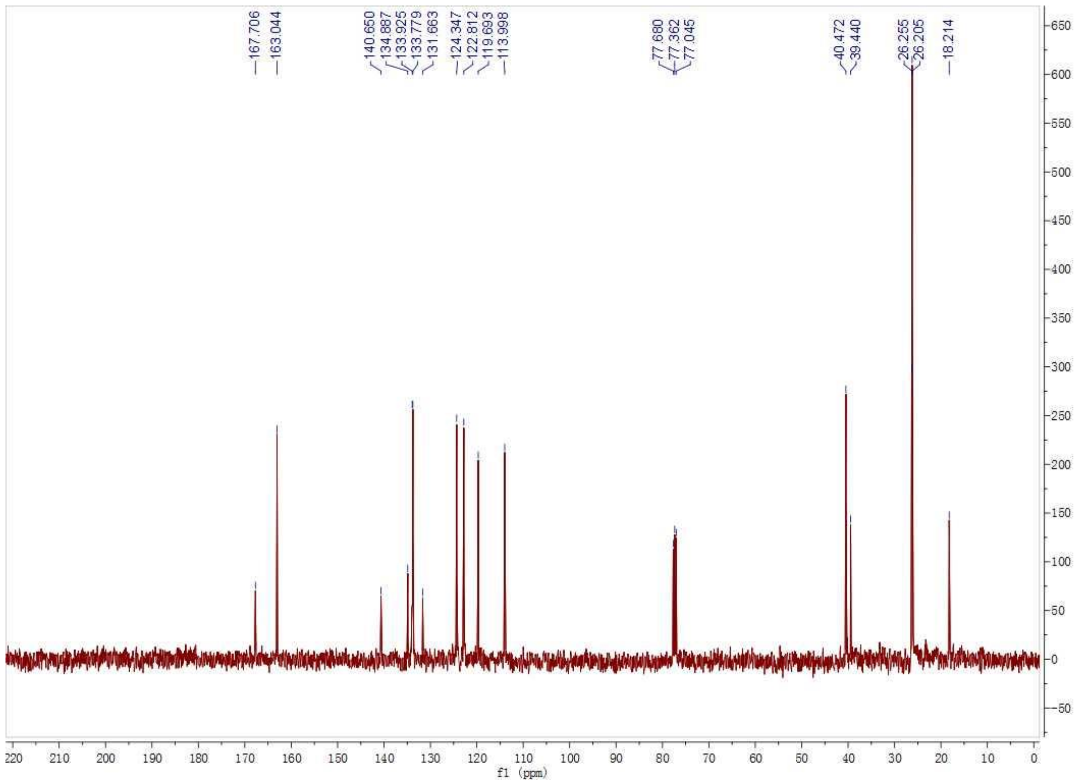

Supplementary Figure 31: ^{13}C NMR spectrum of compound **4e** in $CDCl_3$

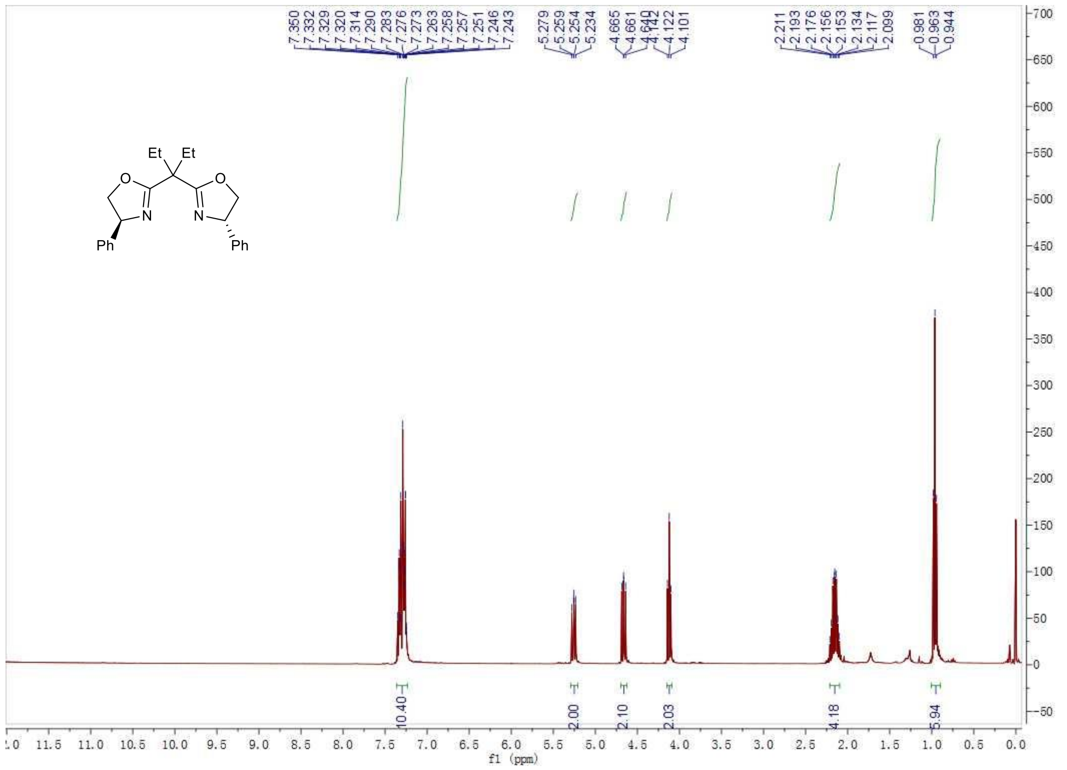

Supplementary Figure 32: ^{1}H NMR spectrum of compound **L1a** in $CDCl_3$

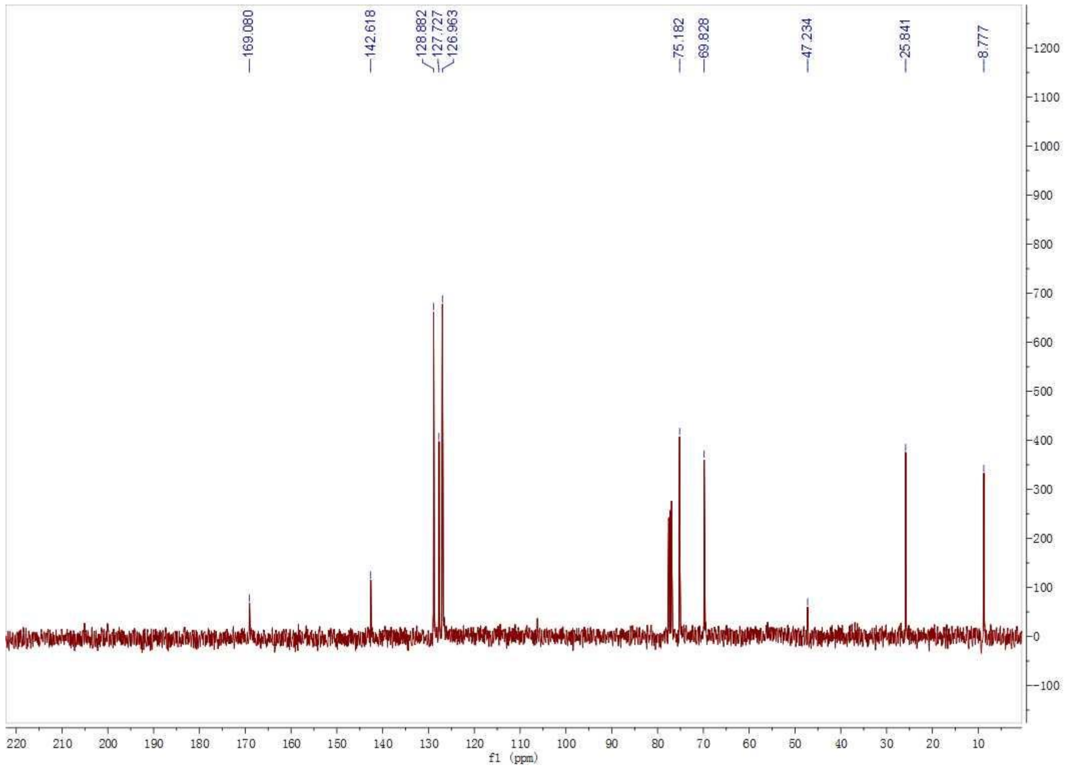

Supplementary Figure 33: ^{13}C NMR spectrum of compound **L1a** in $CDCl_3$

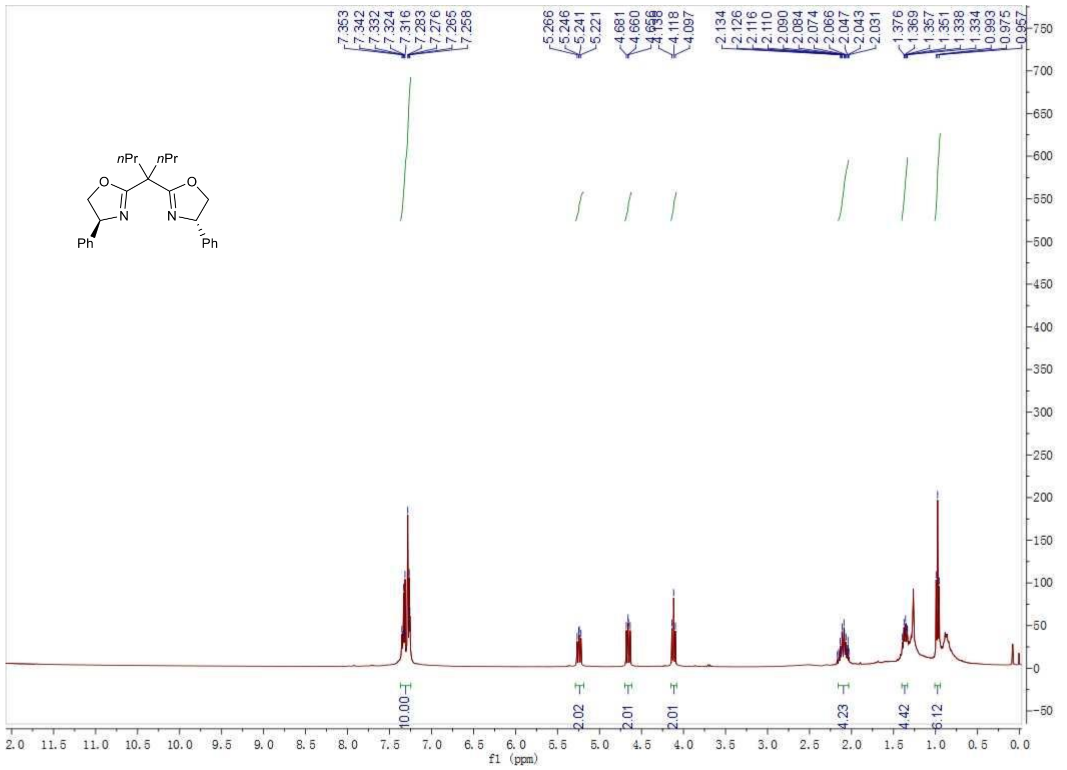

Supplementary Figure 34: ^{1}H NMR spectrum of compound **L1c** in $CDCl_3$

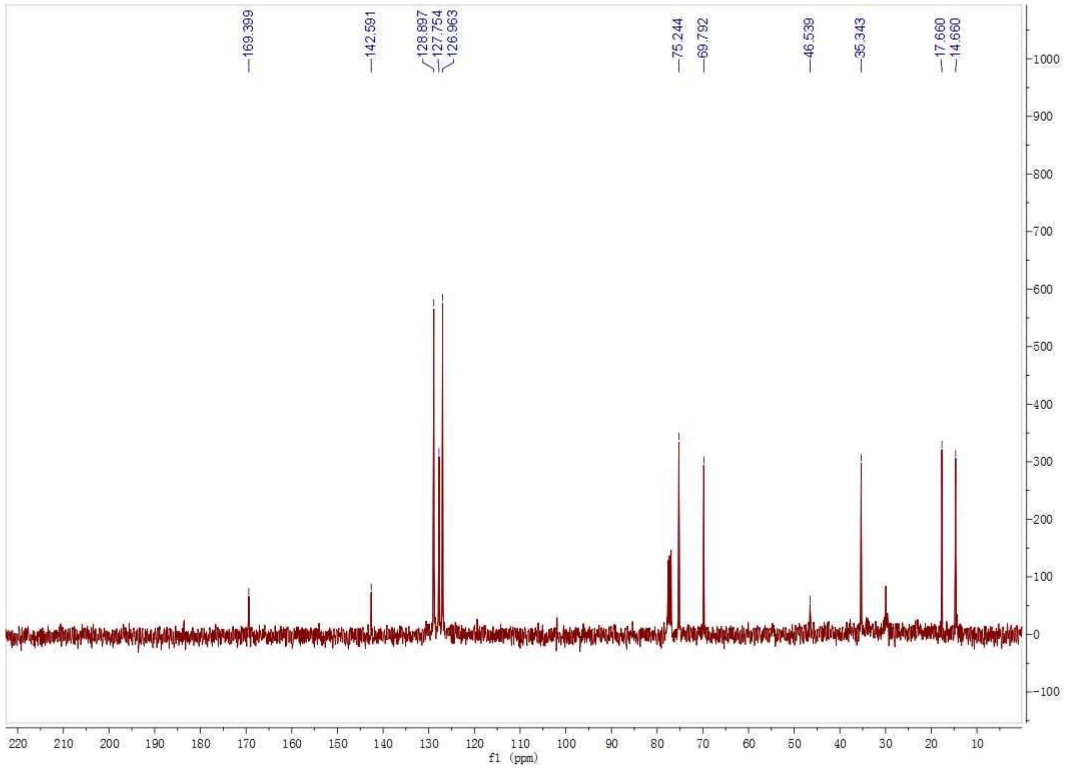

Supplementary Figure 35: ^{13}C NMR spectrum of compound **L1c** in $CDCl_3$

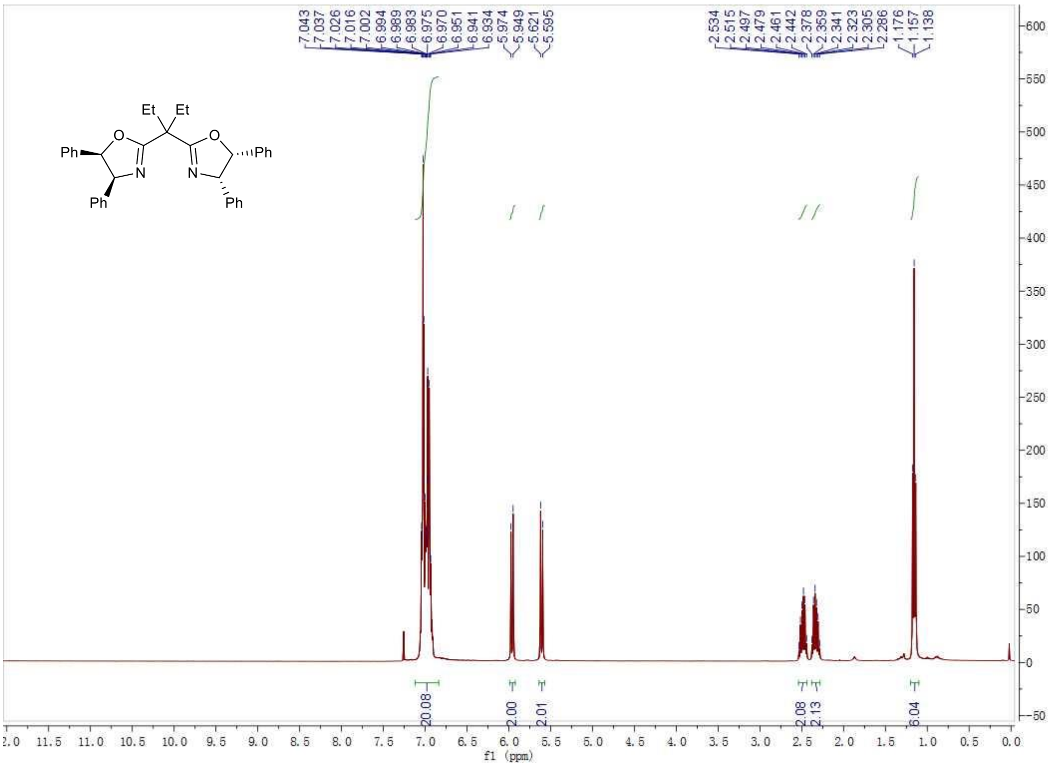

Supplementary Figure 36: ^{1}H NMR spectrum of compound **L13** in $CDCl_3$

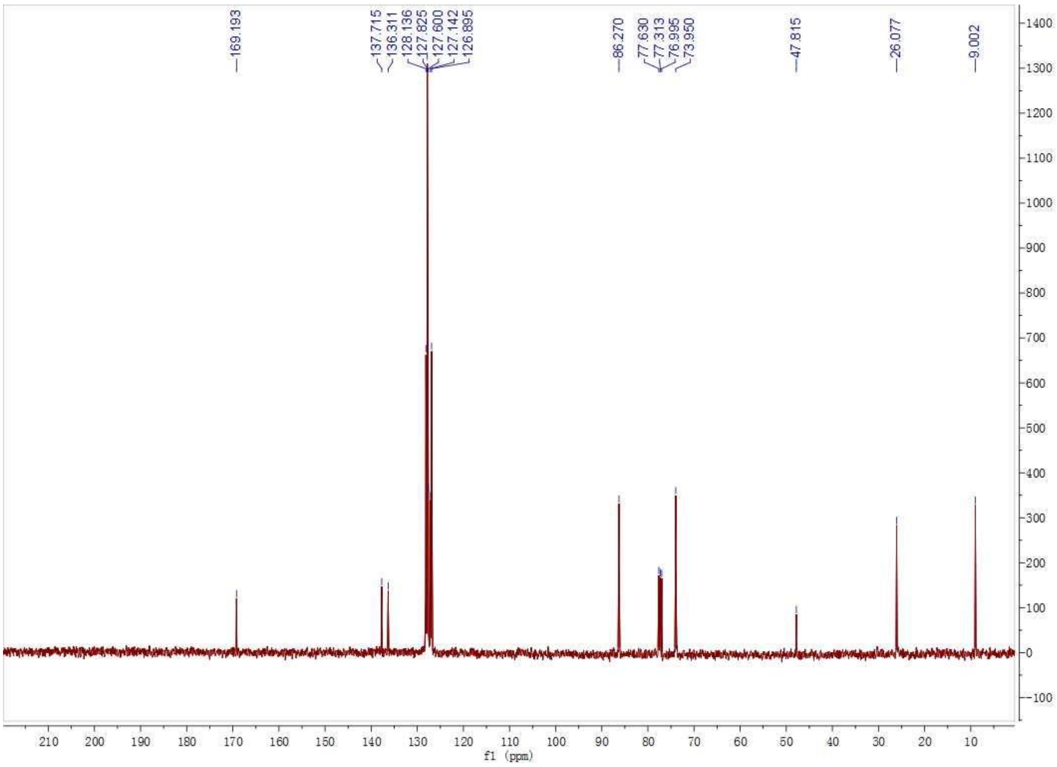

Supplementary Figure 37: ^{13}C NMR spectrum of compound **L13** in $CDCl_3$

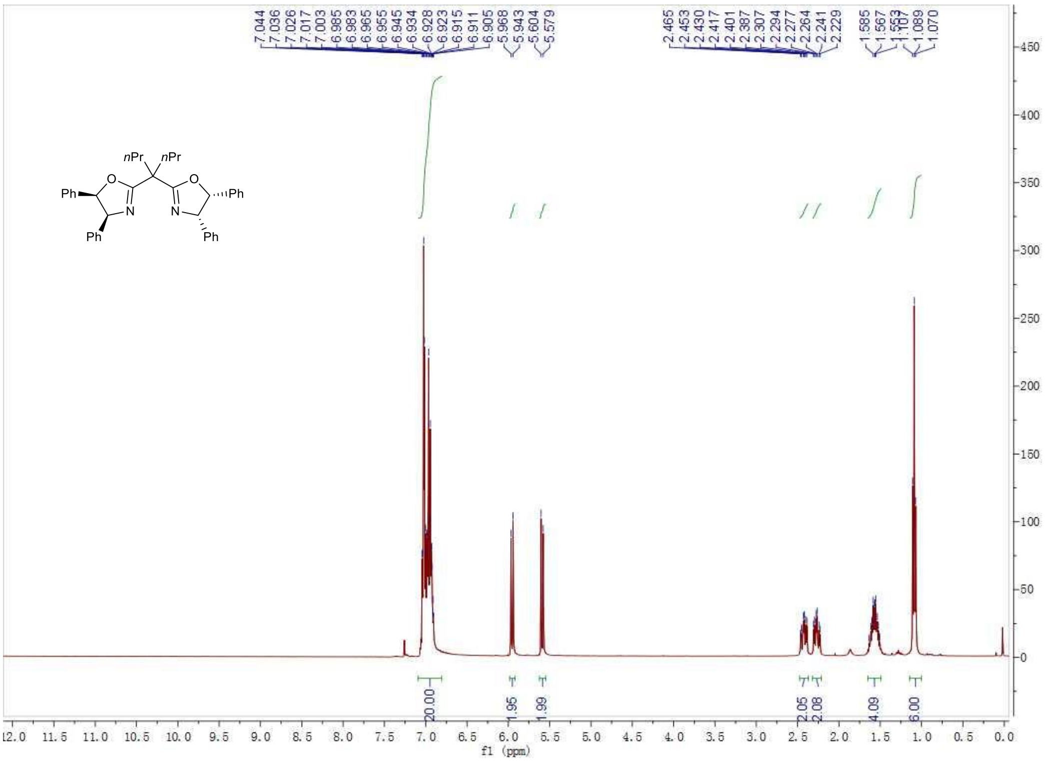

Supplementary Figure 38: ^{1}H NMR spectrum of compound **L2** in $CDCl_3$

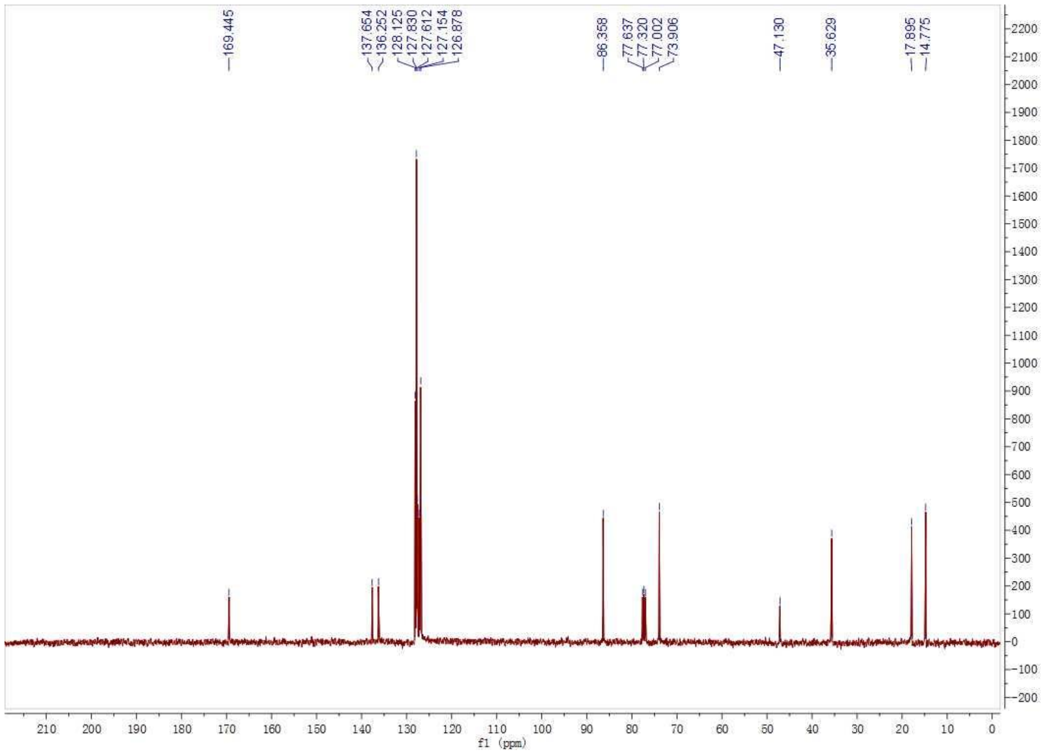

Supplementary Figure 39: ^{13}C NMR spectrum of compound **L2** in $CDCl_3$

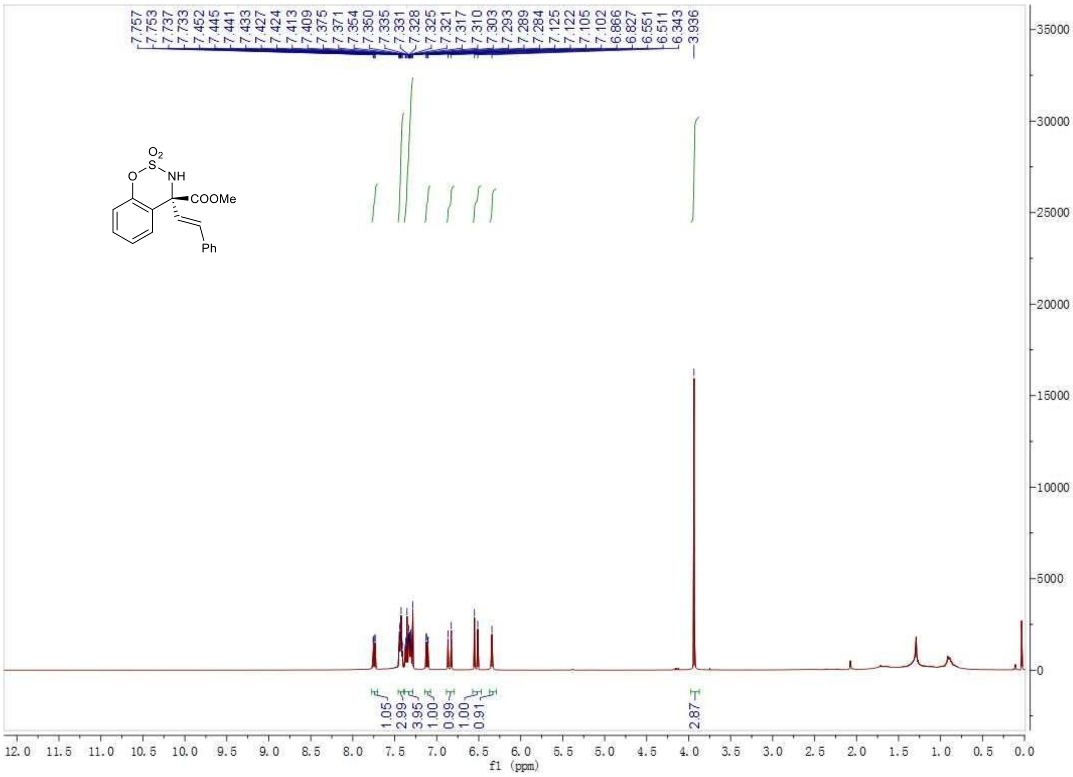

Supplementary Figure 40: ^{1}H NMR spectrum of compound **3aa** in $CDCl_3$

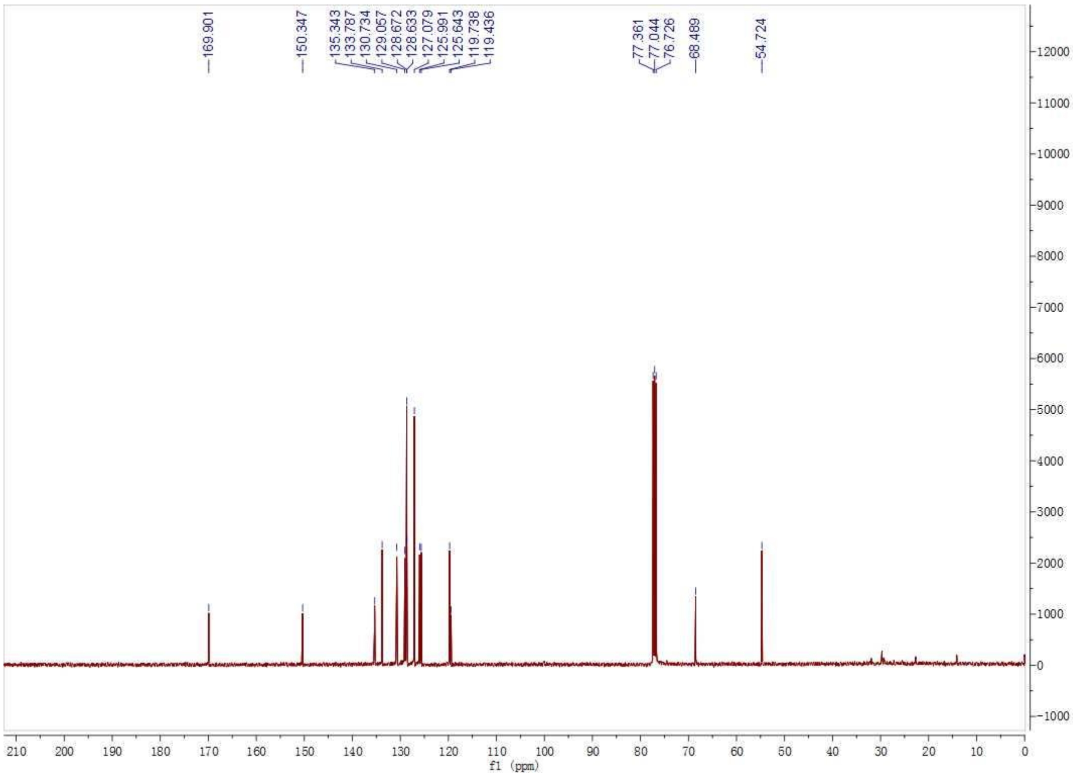

Supplementary Figure 41: ^{13}C NMR spectrum of compound **3aa** in $CDCl_3$

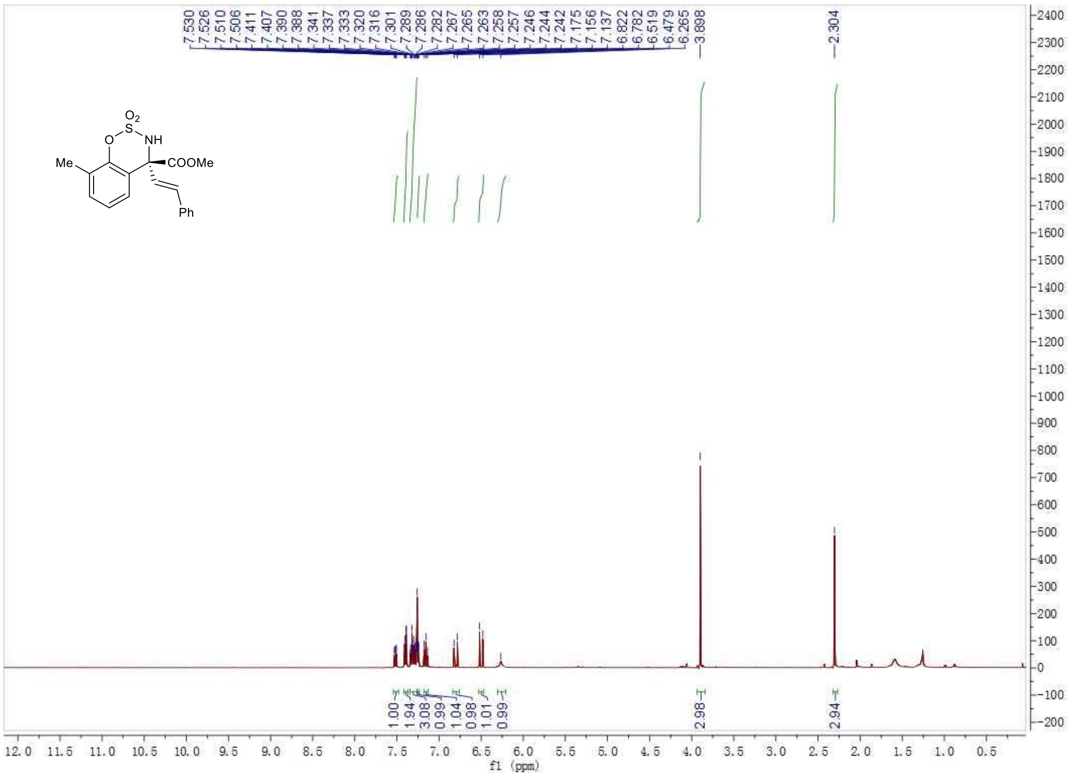

Supplementary Figure 42: ^{1}H NMR spectrum of compound **3ba** in $CDCl_3$

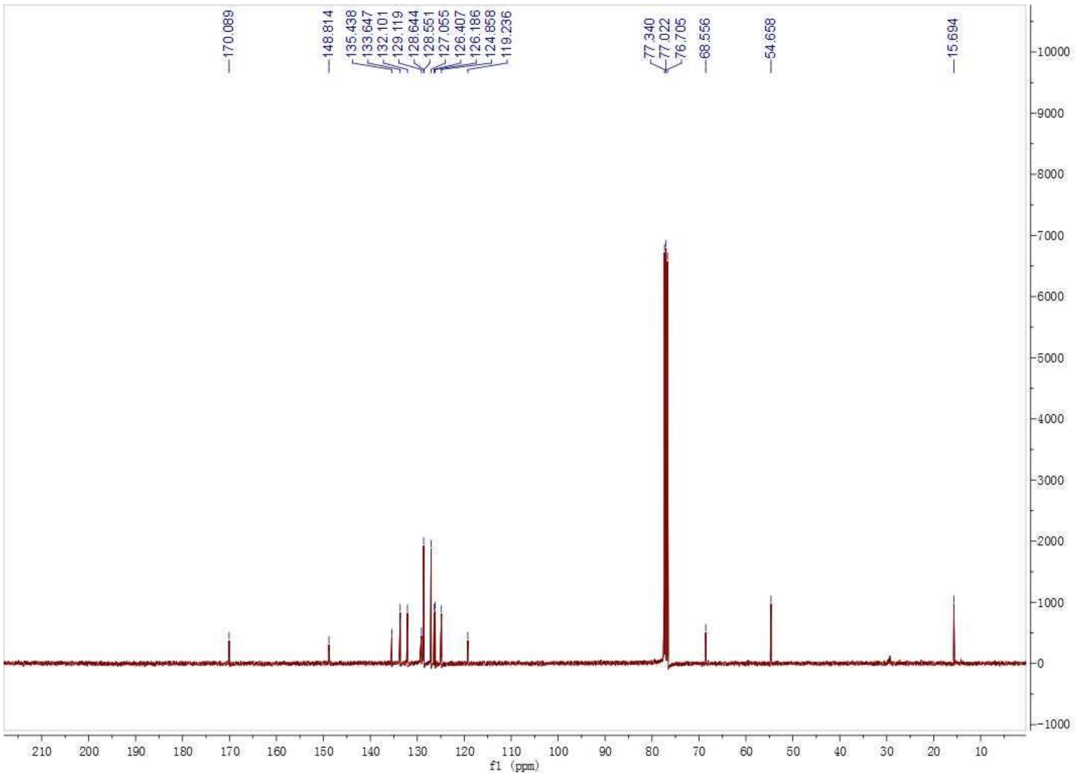

Supplementary Figure 43: ^{13}C NMR spectrum of compound **3ba** in $CDCl_3$

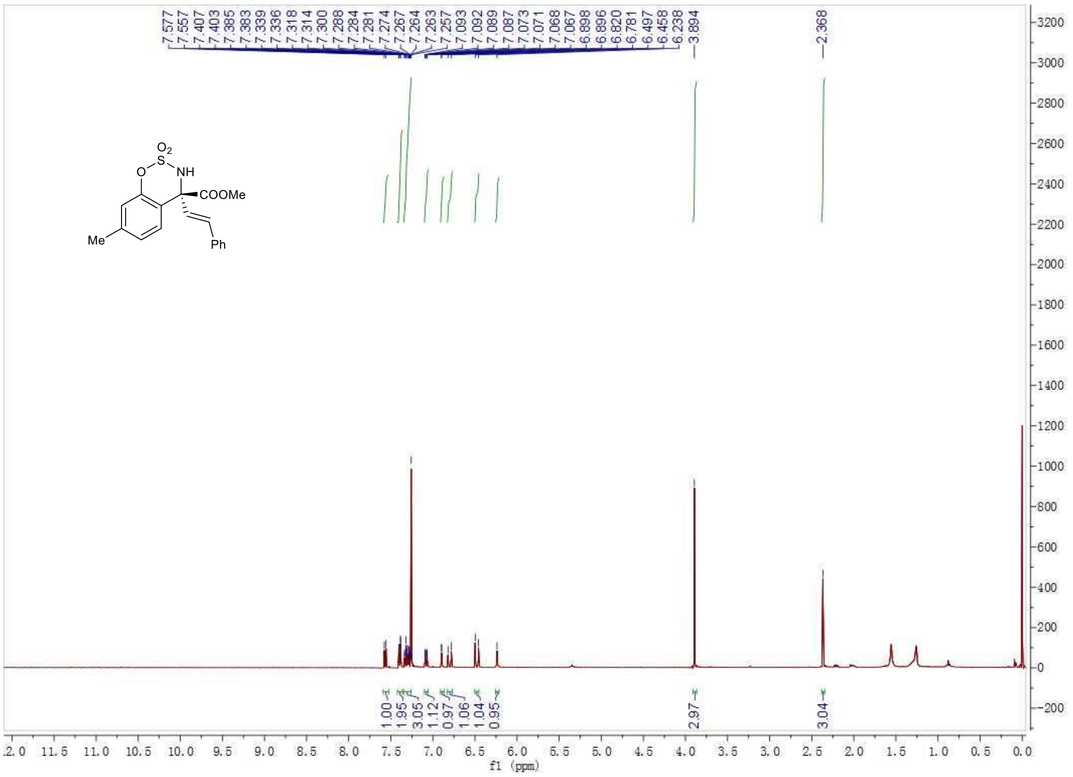

Supplementary Figure 44: ^{1}H NMR spectrum of compound **3ca** in $CDCl_3$

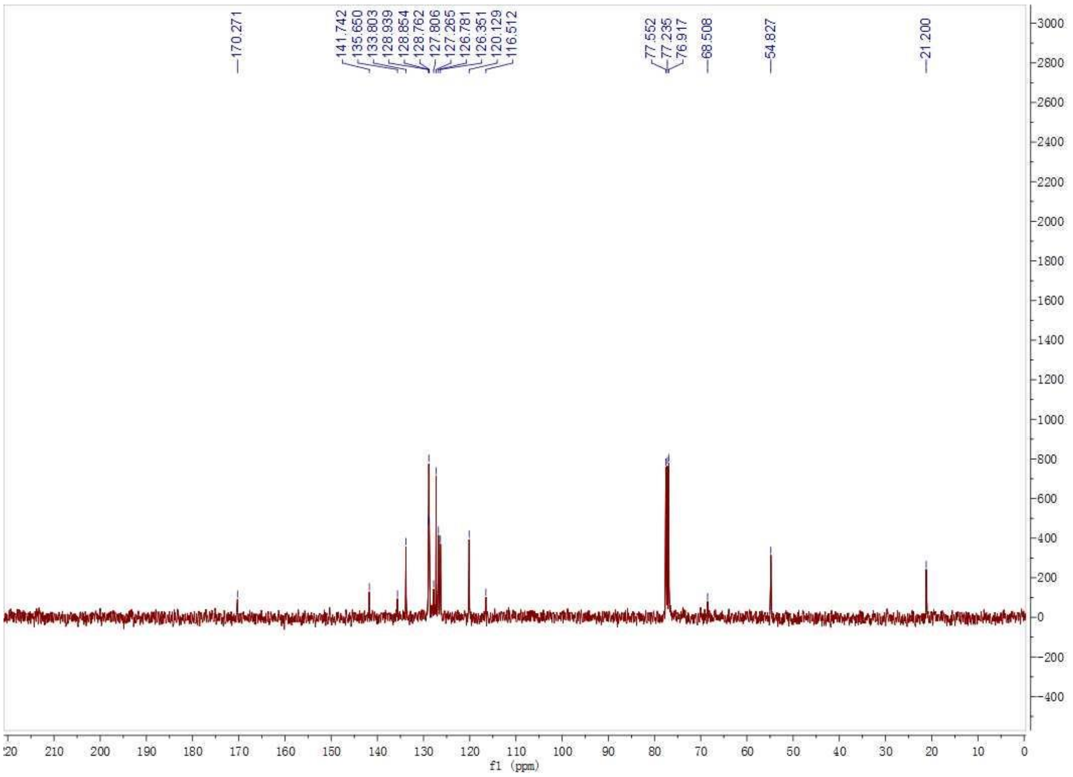

Supplementary Figure 45: ^{13}C NMR spectrum of compound **3ca** in $CDCl_3$

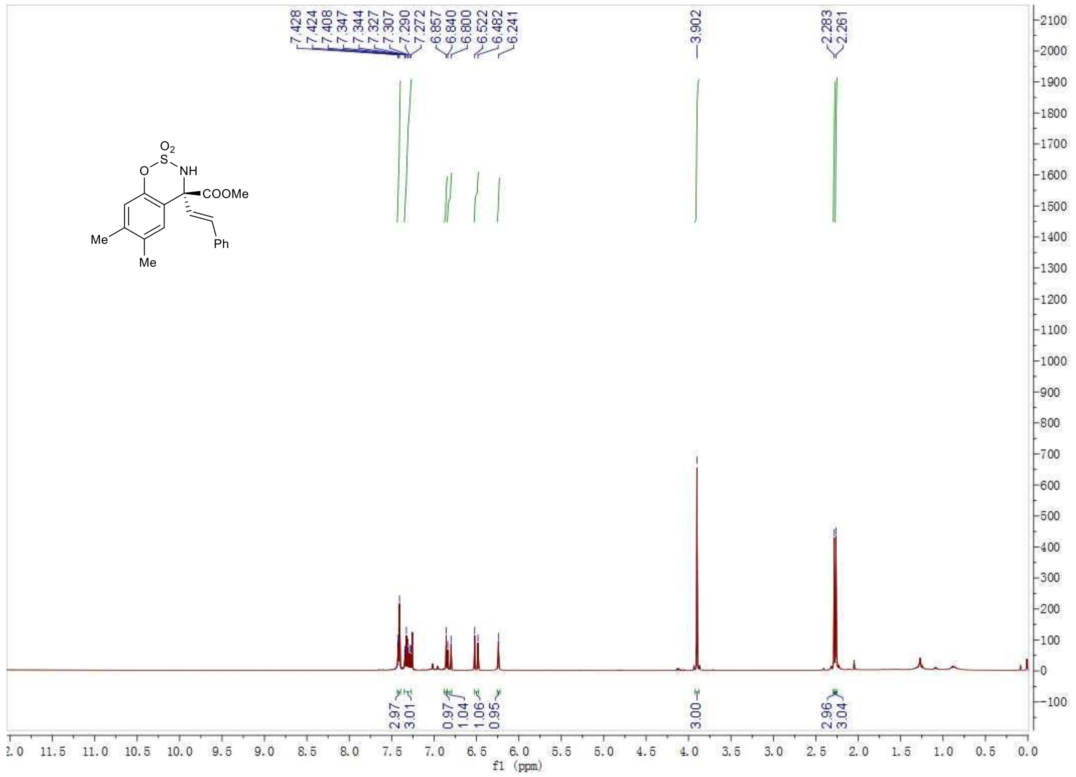

Supplementary Figure 46: ^{1}H NMR spectrum of compound **3da** in $CDCl_3$

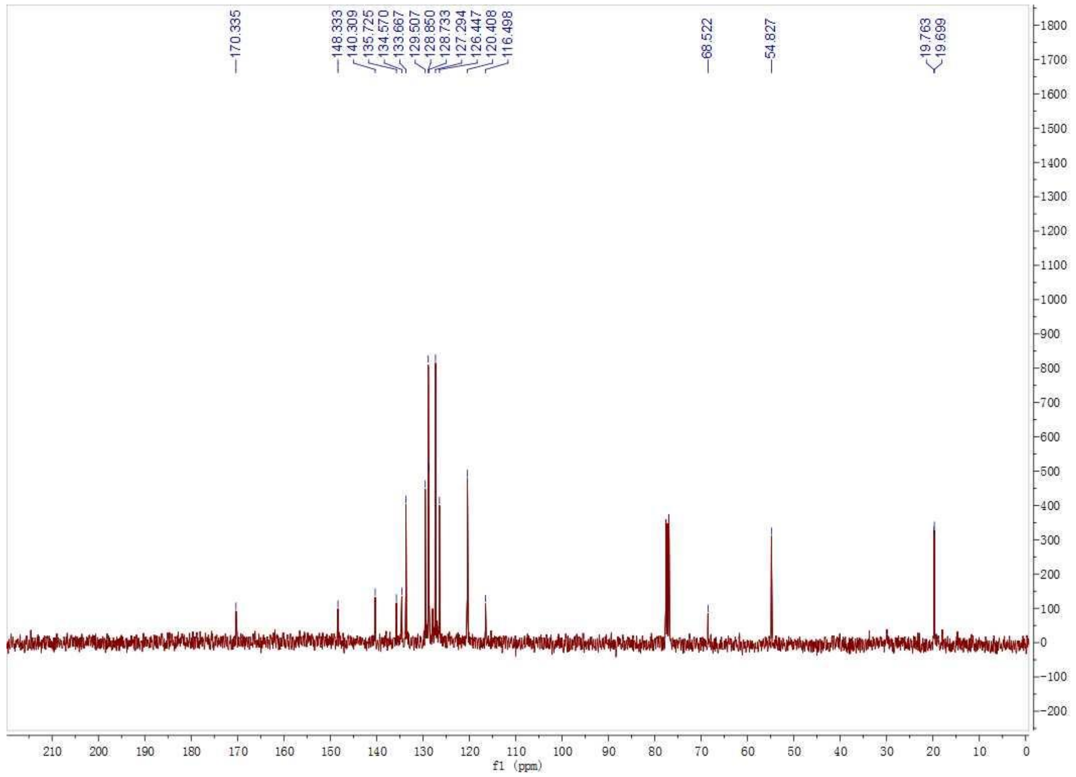

Supplementary Figure 47: ^{13}C NMR spectrum of compound **3da** in $CDCl_3$

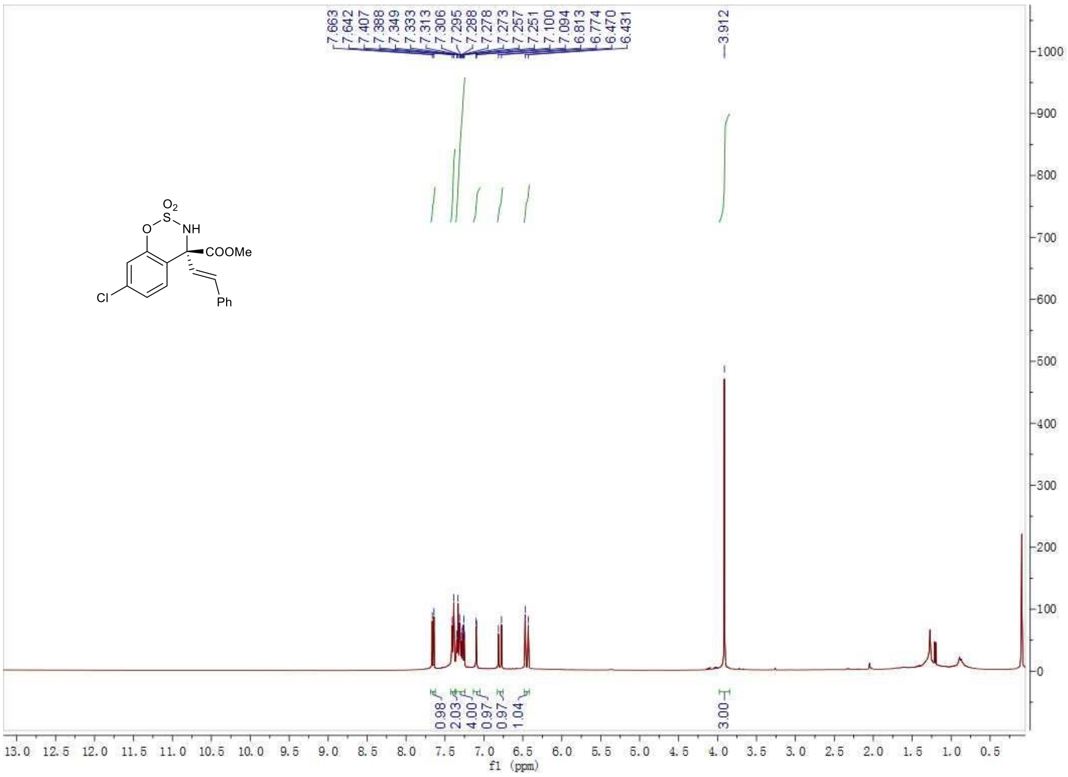

Supplementary Figure 48: ^{1}H NMR spectrum of compound **3ea** in $CDCl_3$

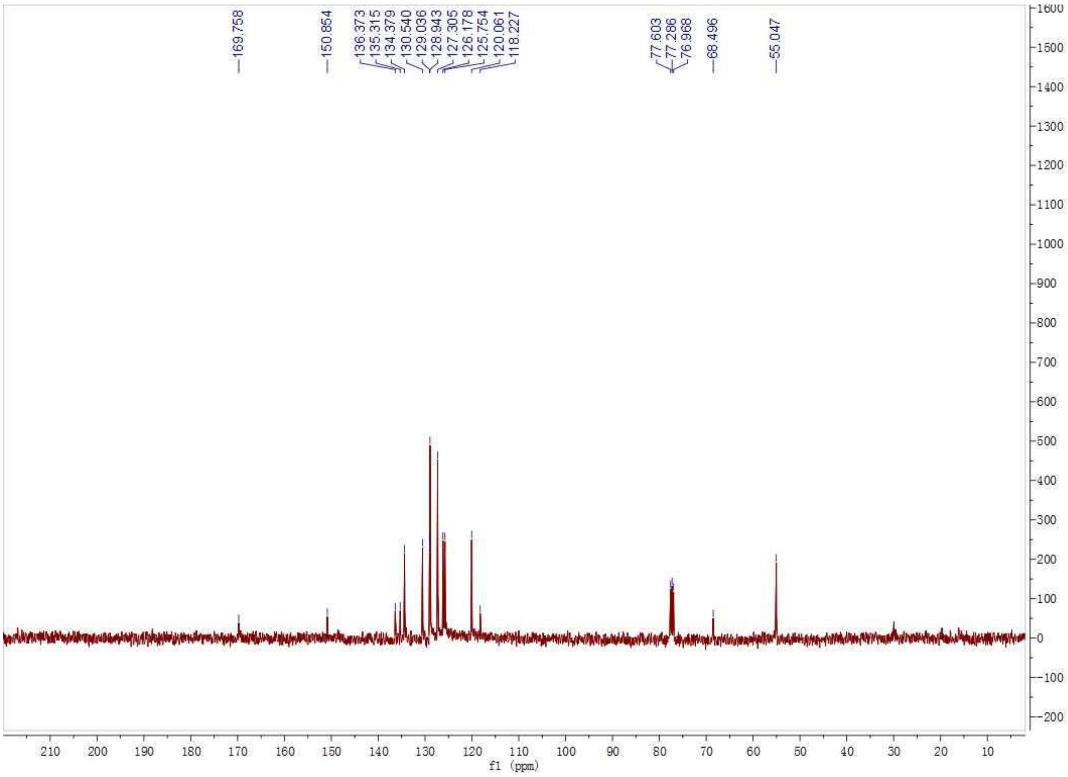

Supplementary Figure 49: ^{13}C NMR spectrum of compound **3ea** in $CDCl_3$

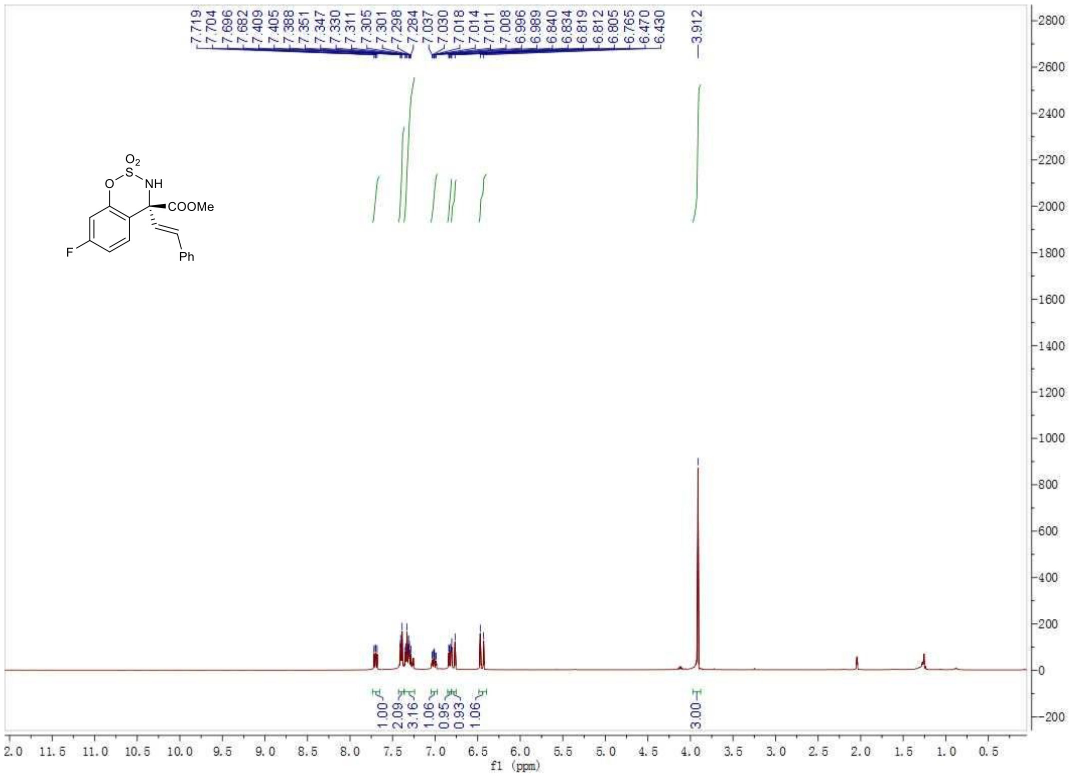

Supplementary Figure 50: ^{1}H NMR spectrum of compound **3fa** in $CDCl_3$

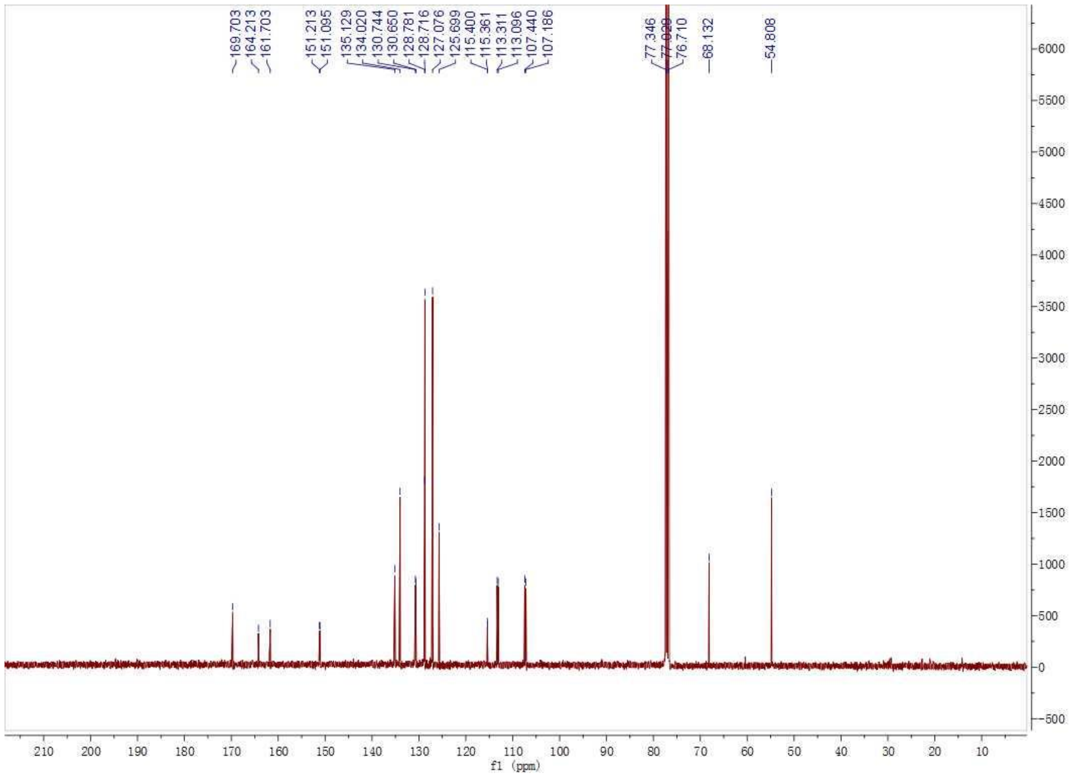

Supplementary Figure 51: ^{13}C NMR spectrum of compound **3fa** in $CDCl_3$

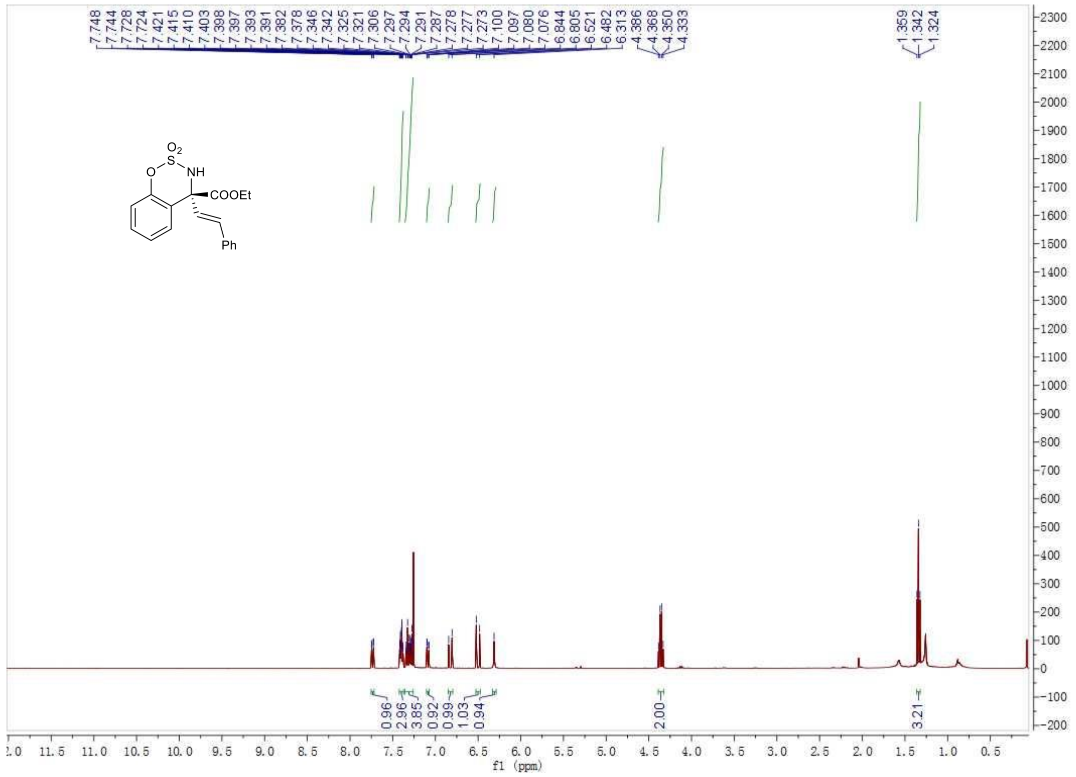

Supplementary Figure 52: ^{1}H NMR spectrum of compound **3ga** in $CDCl_3$

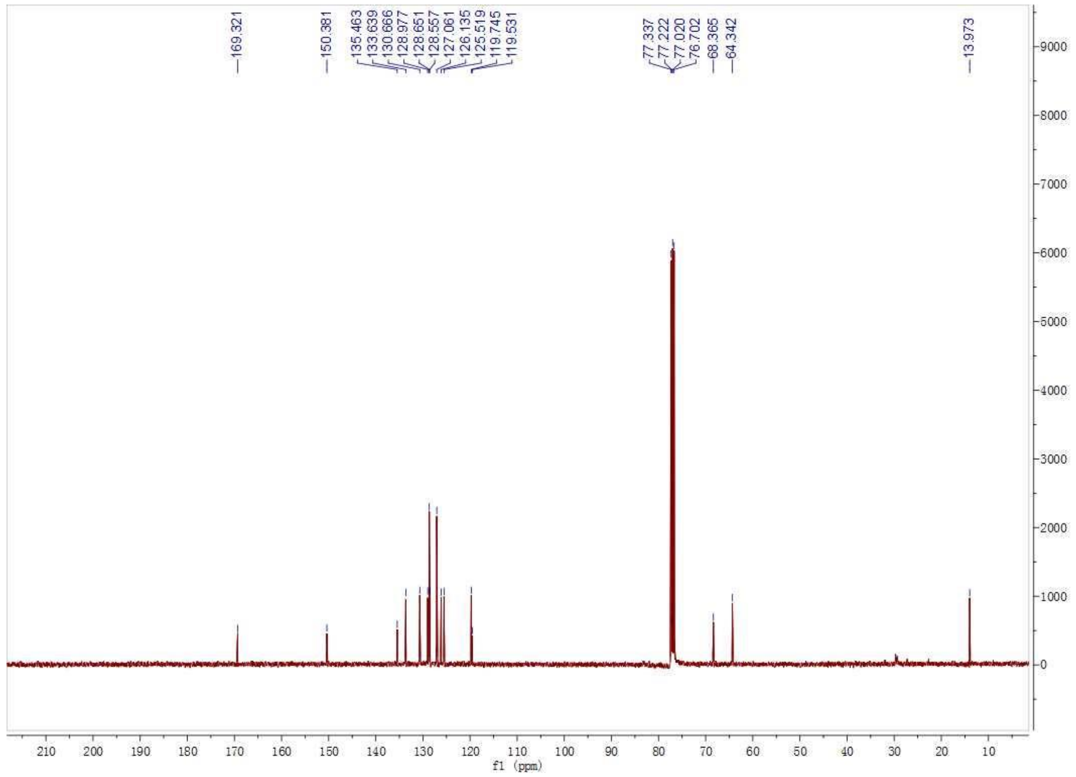

Supplementary Figure 53: ^{13}C NMR spectrum of compound **3ga** in $CDCl_3$

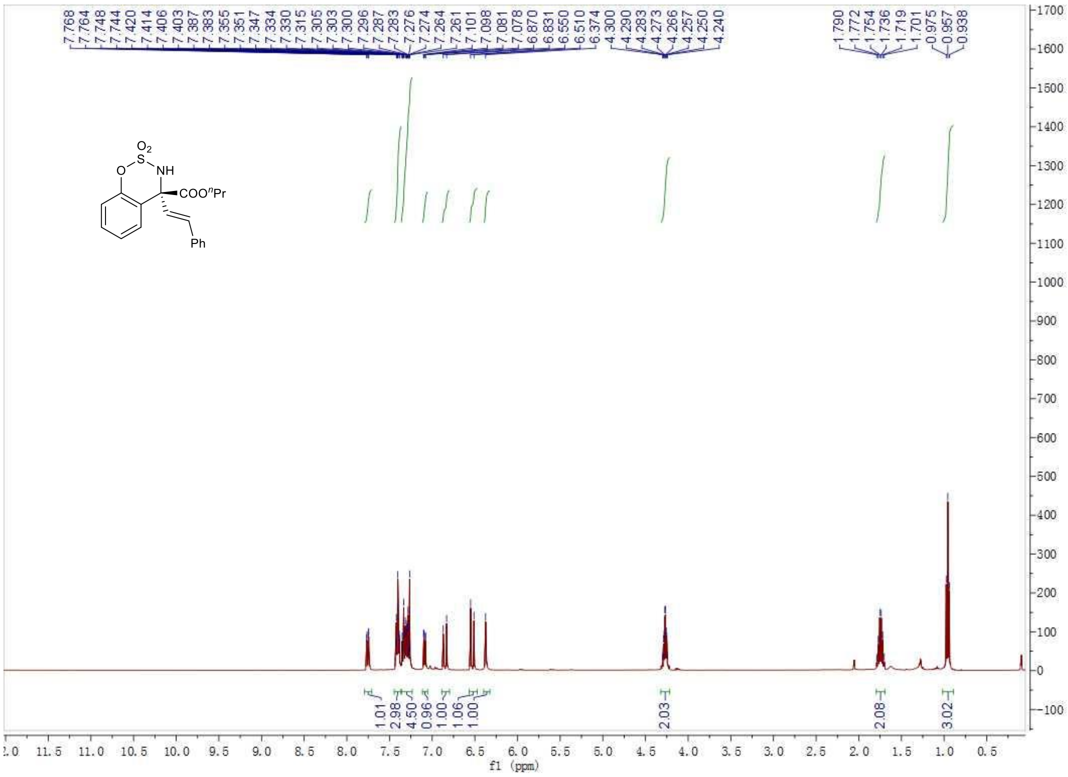

Supplementary Figure 54: ^{1}H NMR spectrum of compound **3ha** in $CDCl_3$

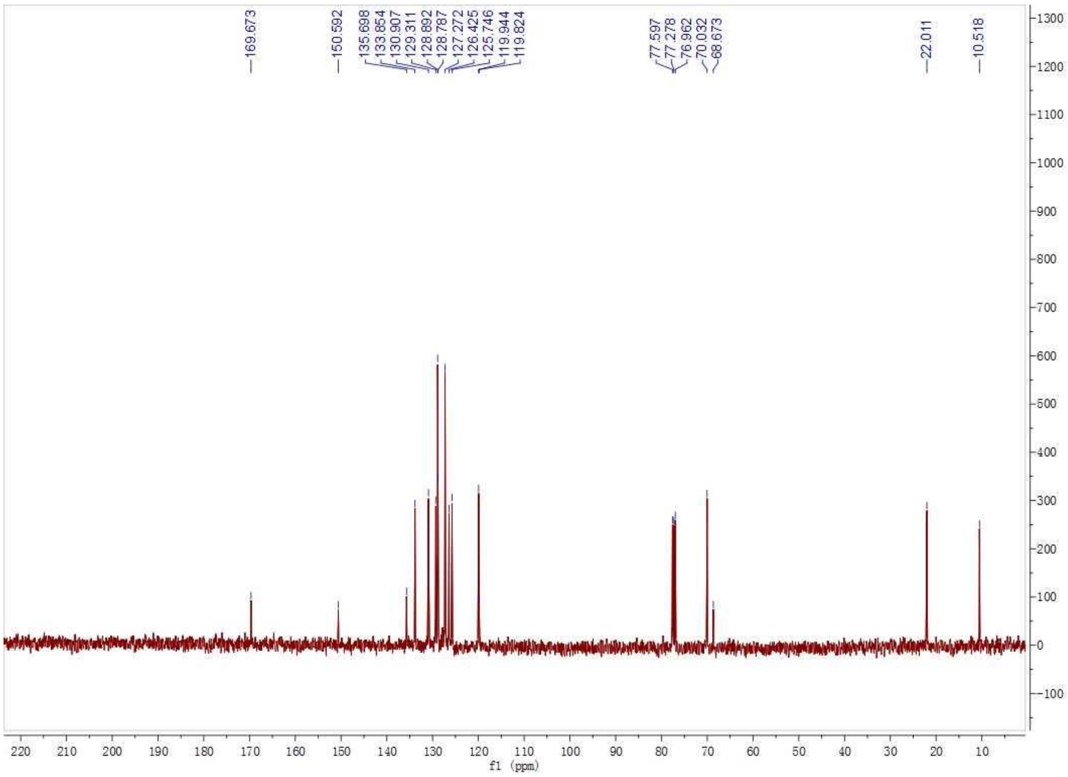

Supplementary Figure 55: ^{13}C NMR spectrum of compound **3ha** in $CDCl_3$

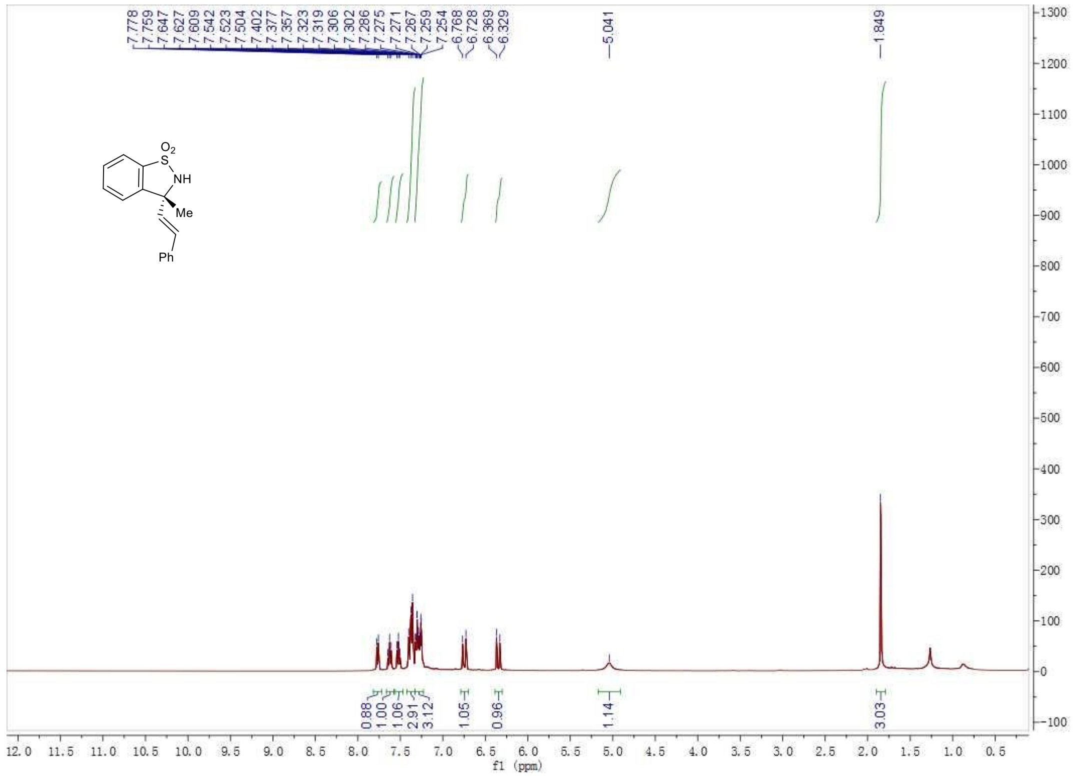

Supplementary Figure 56: ^{1}H NMR spectrum of compound **3ia** in $CDCl_3$

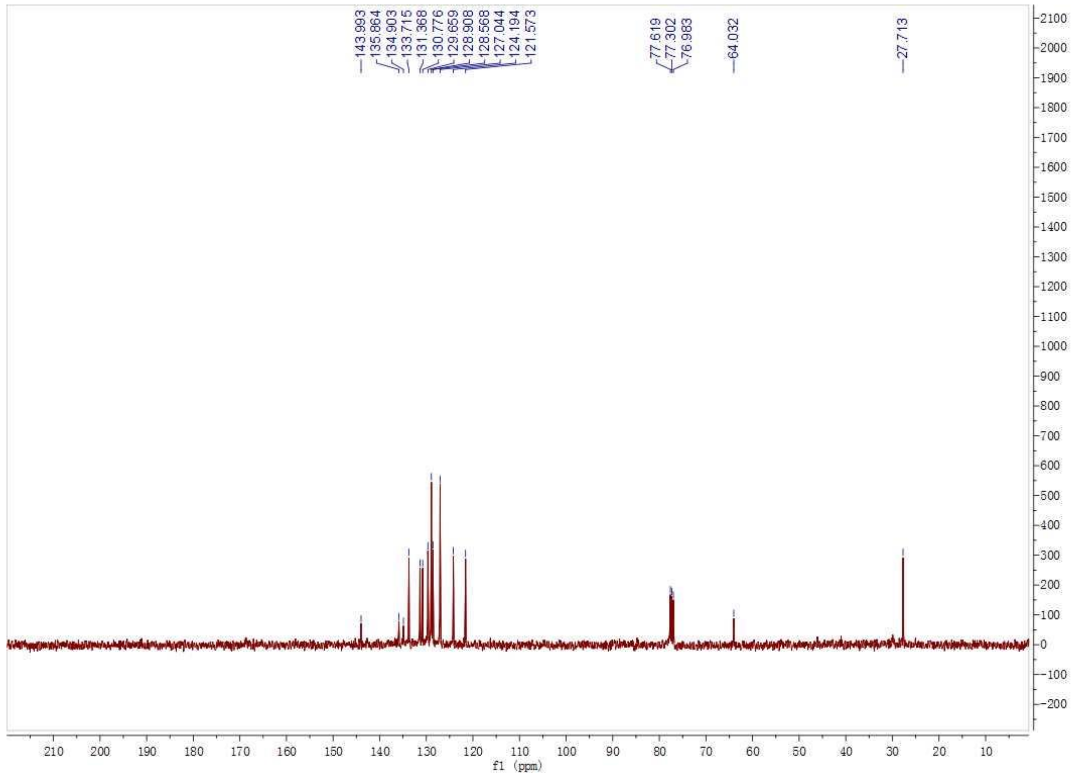

Supplementary Figure 57: ^{13}C NMR spectrum of compound **3ia** in $CDCl_3$

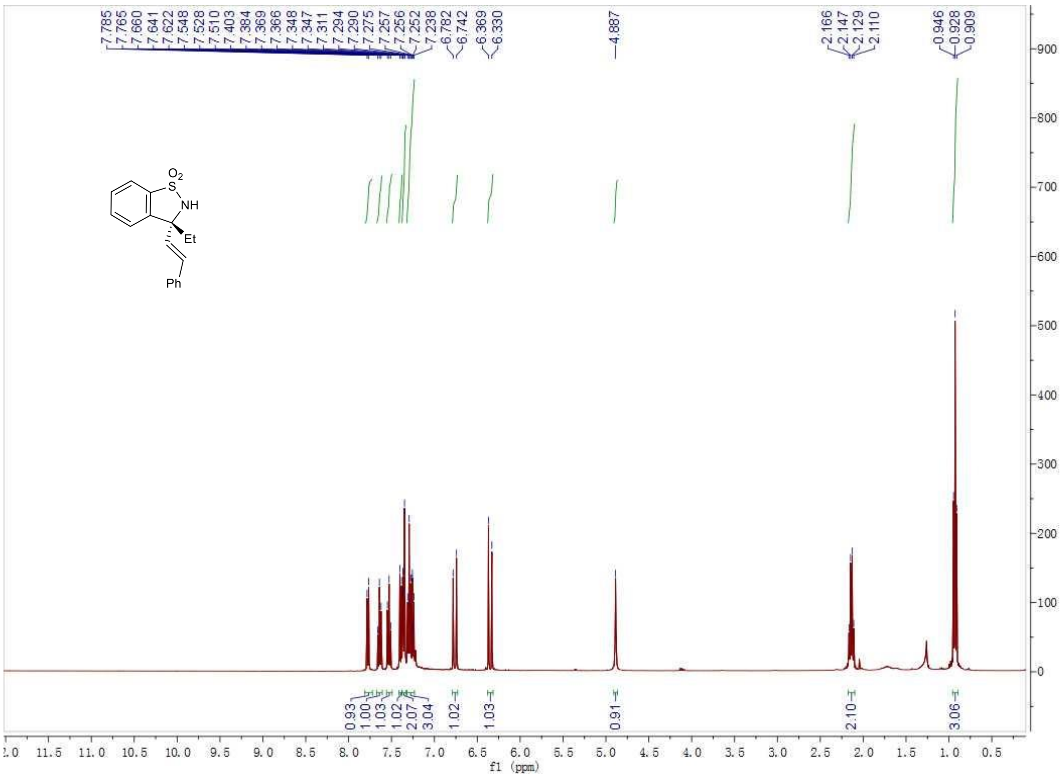

Supplementary Figure 58: ^{1}H NMR spectrum of compound **3ja** in $CDCl_3$

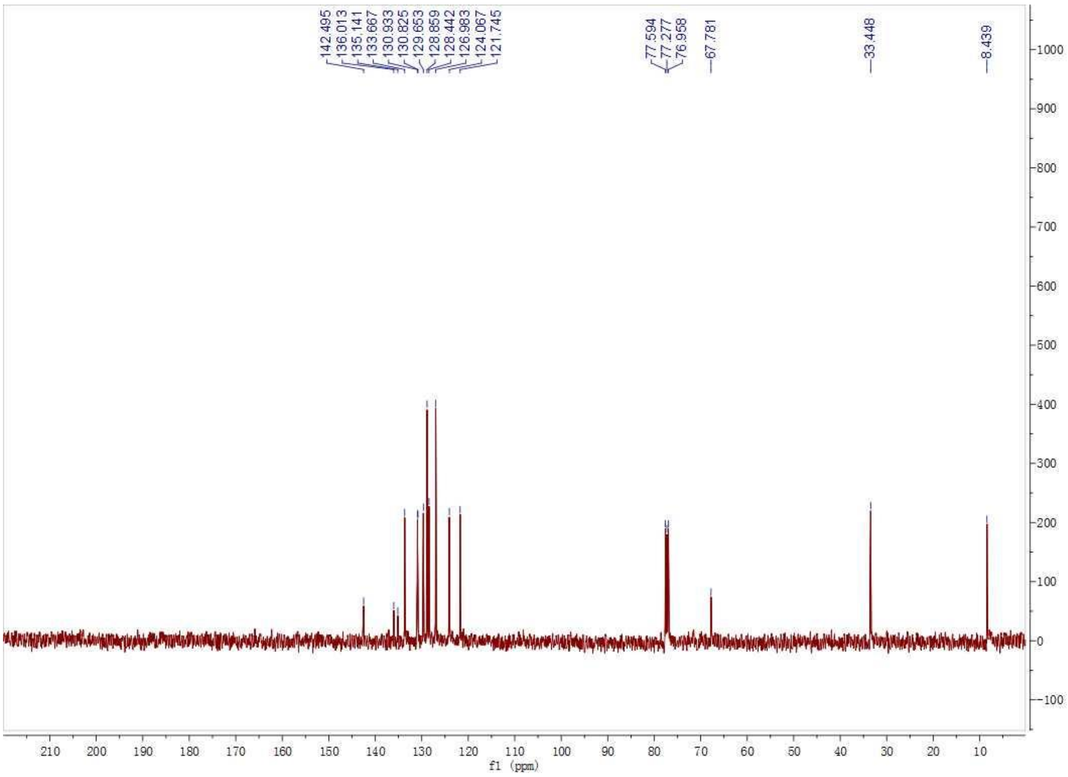

Supplementary Figure 59: ^{13}C NMR spectrum of compound **3ja** in $CDCl_3$

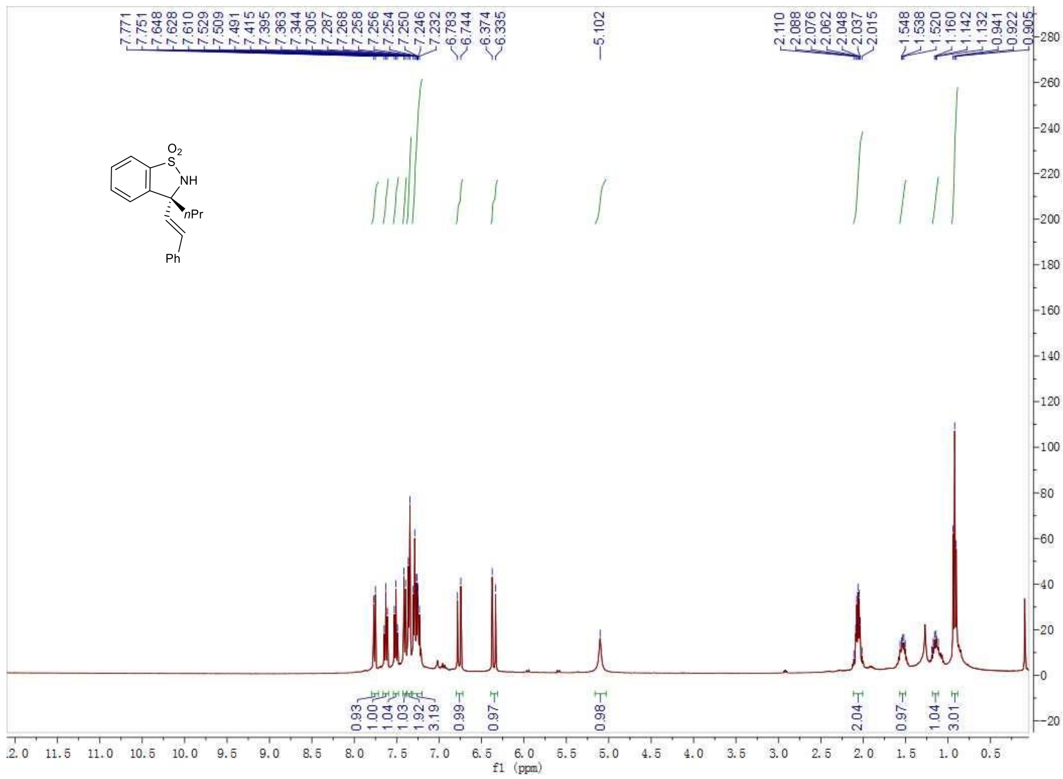

Supplementary Figure 60: ^{1}H NMR spectrum of compound **3ka** in $CDCl_3$

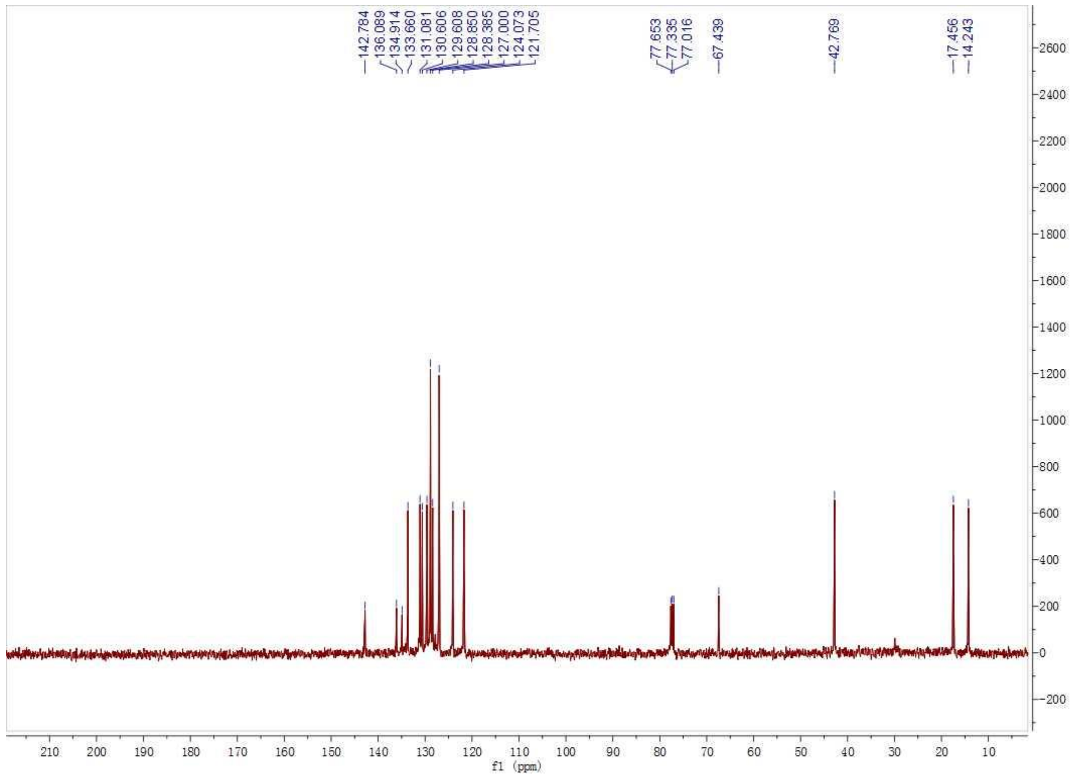

Supplementary Figure 61: ^{13}C NMR spectrum of compound **3ka** in $CDCl_3$

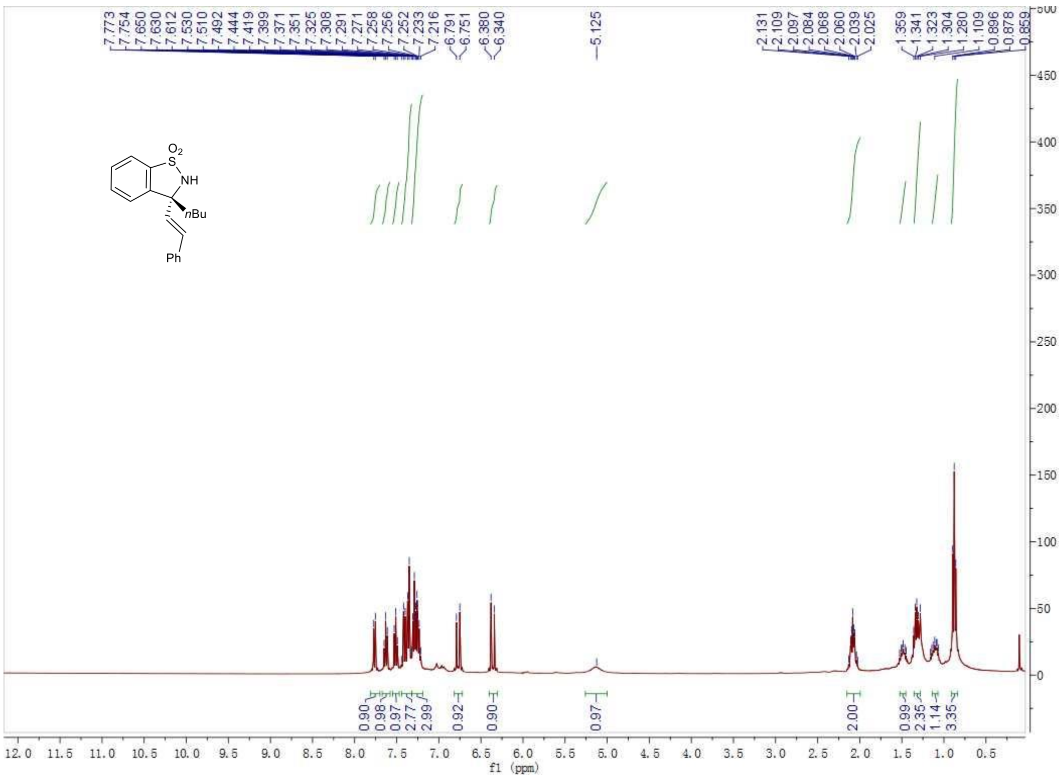

Supplementary Figure 62: ^{1}H NMR spectrum of compound **3la** in $CDCl_3$

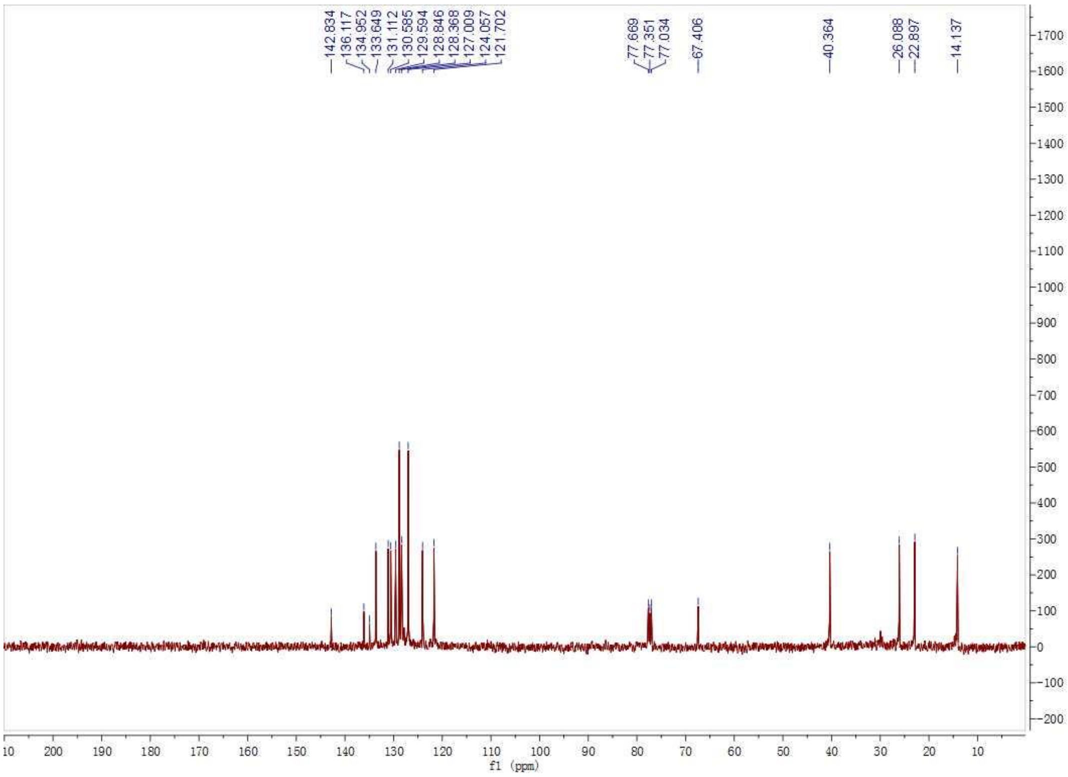

Supplementary Figure 63: ^{13}C NMR spectrum of compound **3la** in $CDCl_3$

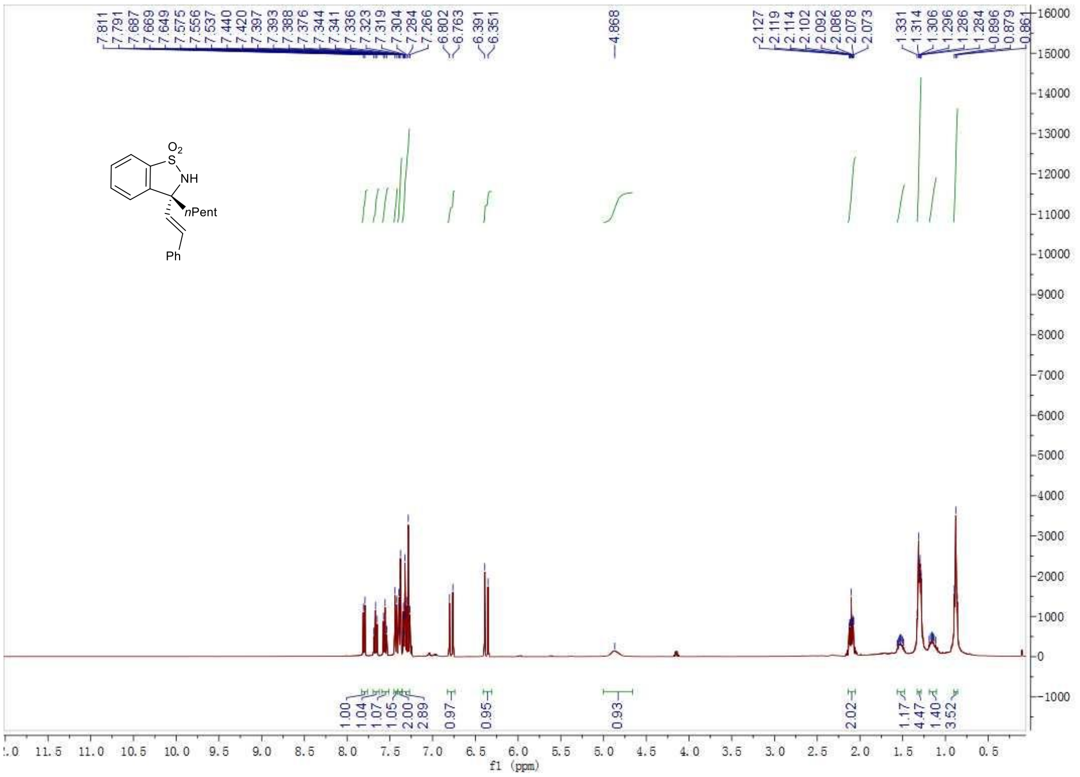

Supplementary Figure 64: ^{1}H NMR spectrum of compound **3ma** in $CDCl_3$

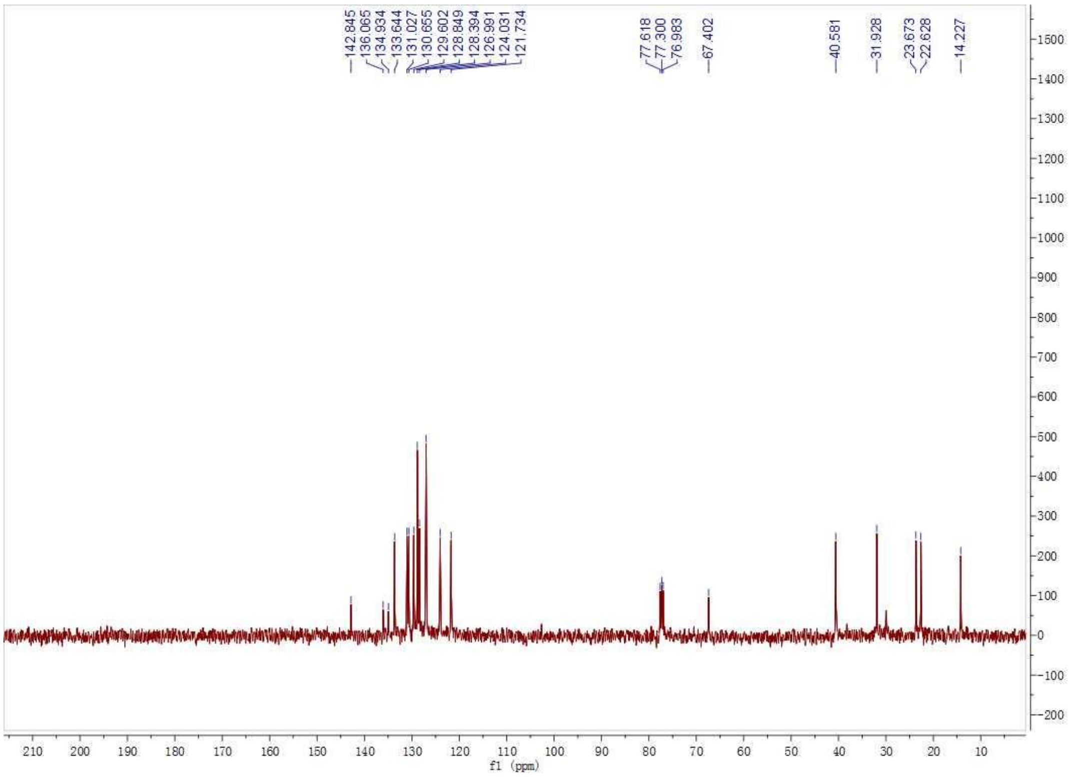

Supplementary Figure 65: ^{13}C NMR spectrum of compound **3ma** in $CDCl_3$

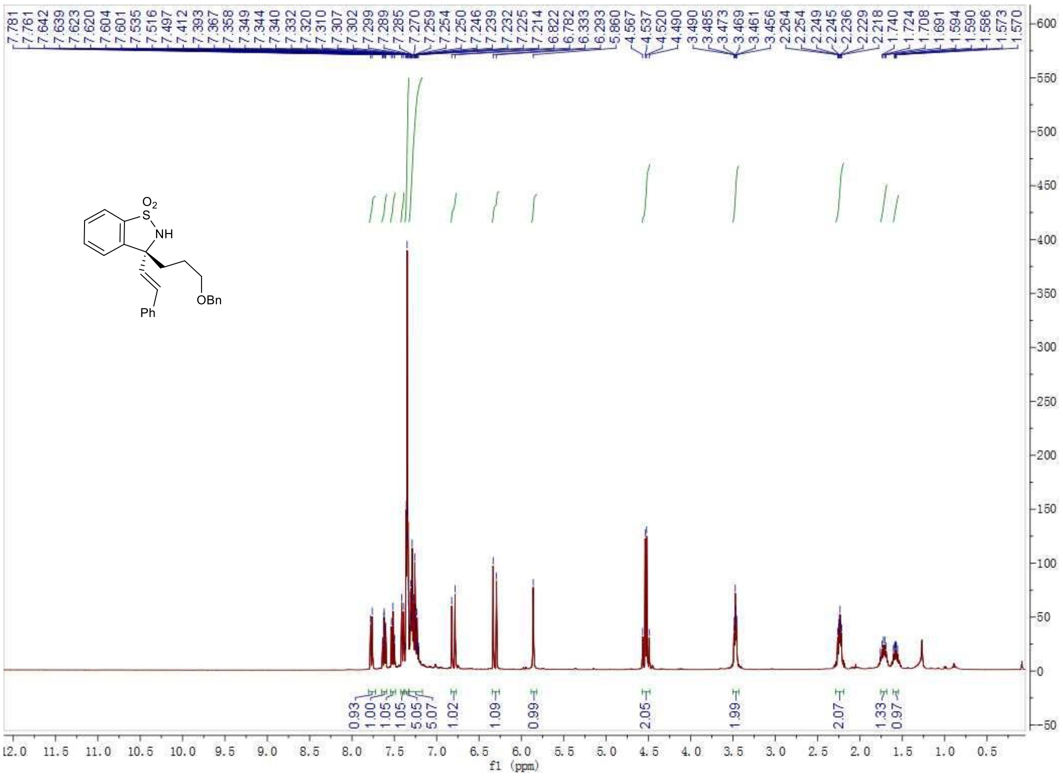

Supplementary Figure 66: ^{1}H NMR spectrum of compound **3na** in $CDCl_3$

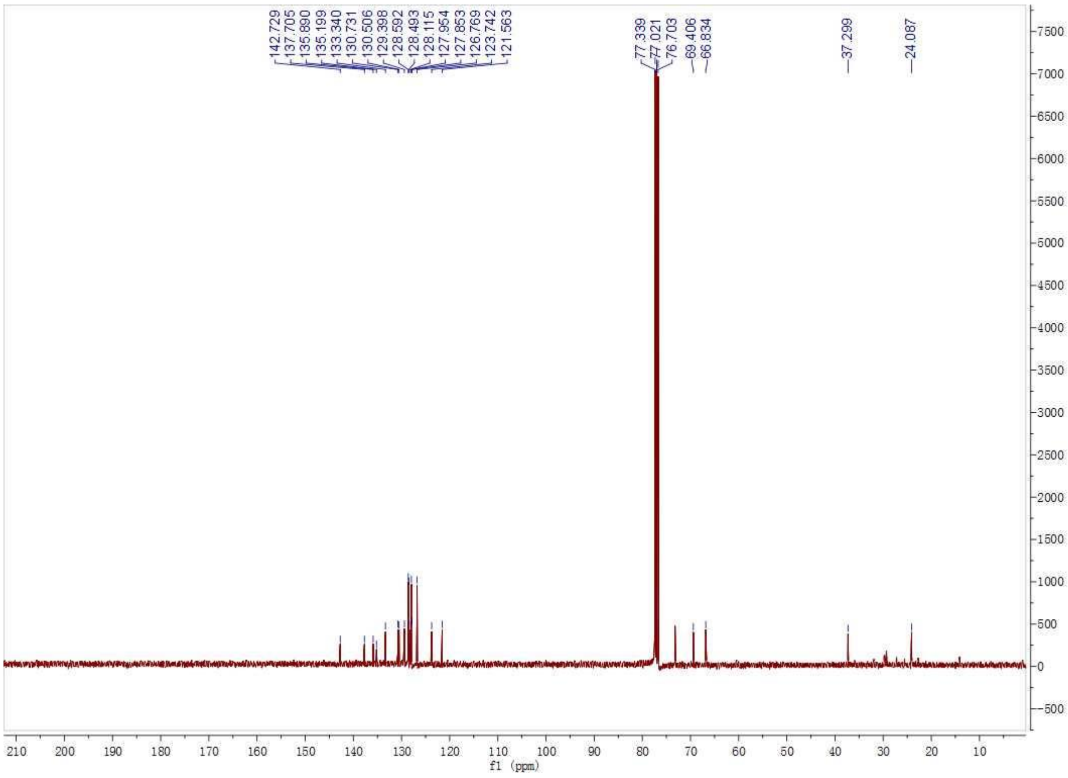

Supplementary Figure 67: ^{13}C NMR spectrum of compound **3na** in $CDCl_3$

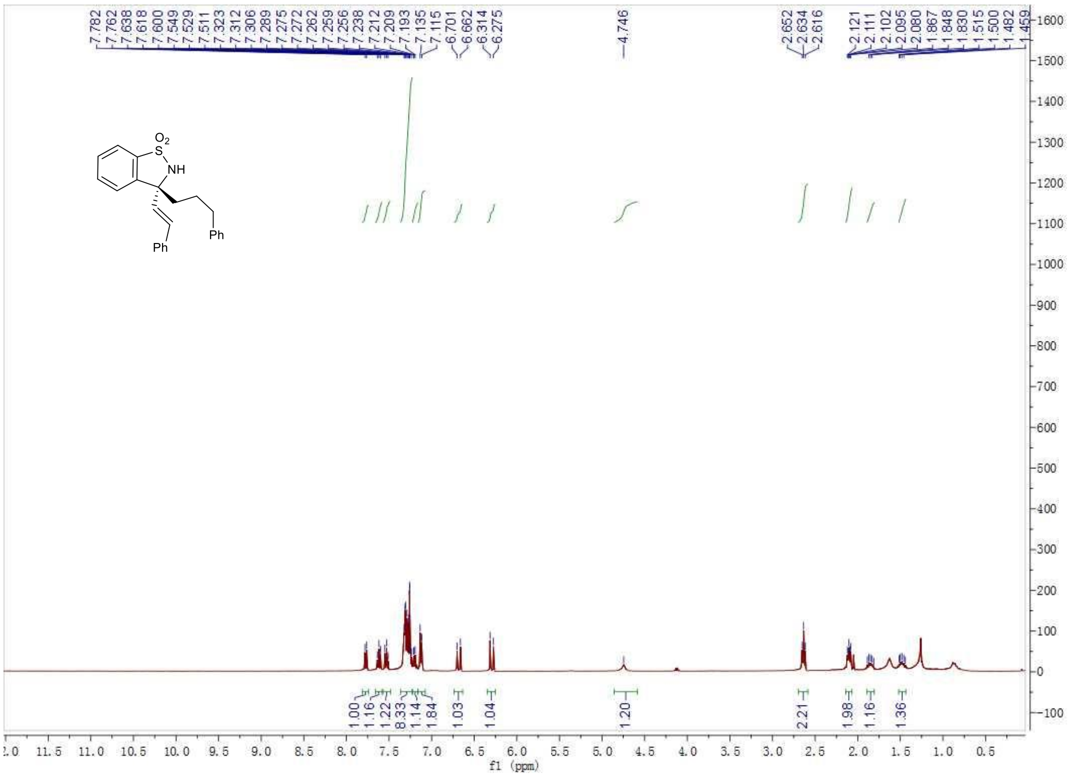

Supplementary Figure 68: ^{1}H NMR spectrum of compound **3oa** in $CDCl_3$

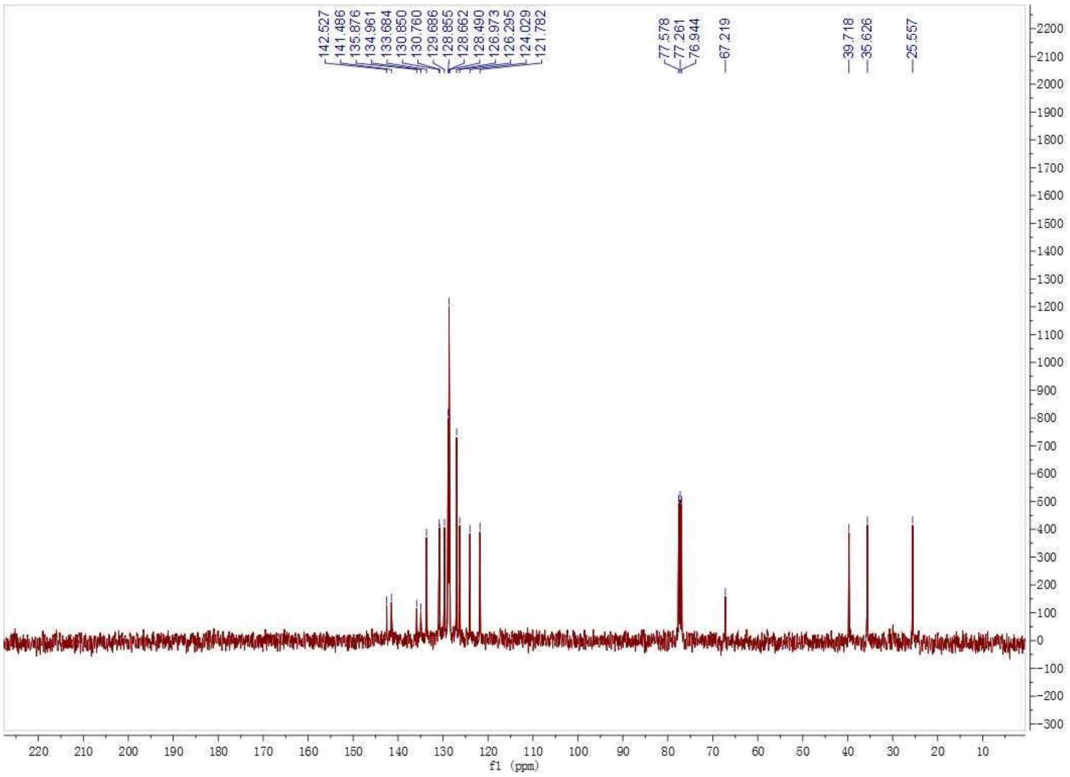

Supplementary Figure 69: ^{13}C NMR spectrum of compound **3oa** in $CDCl_3$

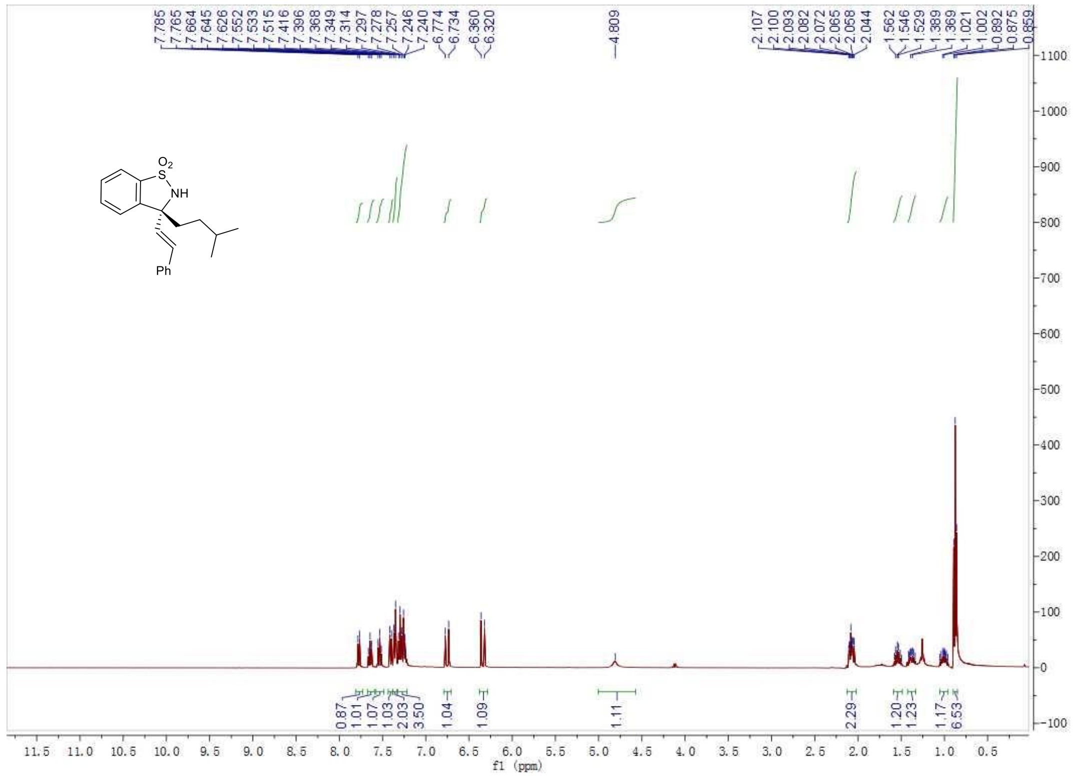

Supplementary Figure 70: ^{1}H NMR spectrum of compound **3pa** in $CDCl_3$

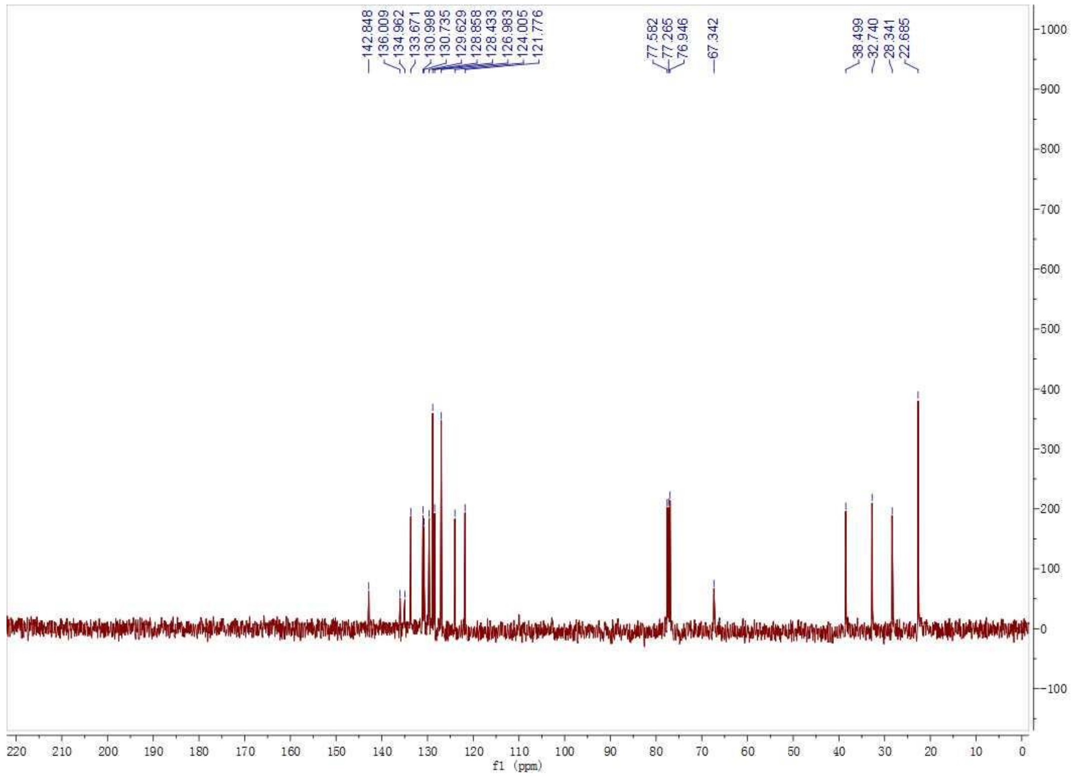

Supplementary Figure 71: ^{13}C NMR spectrum of compound **3pa** in $CDCl_3$

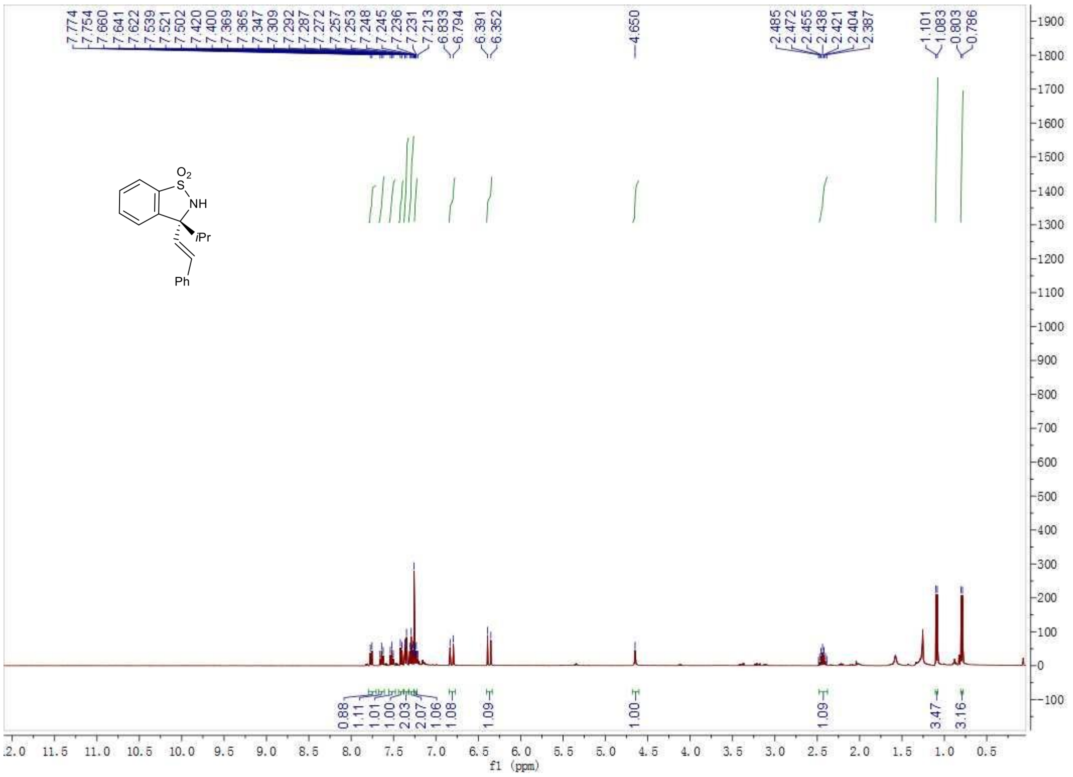

Supplementary Figure 72: ^{1}H NMR spectrum of compound **3qa** in $CDCl_3$

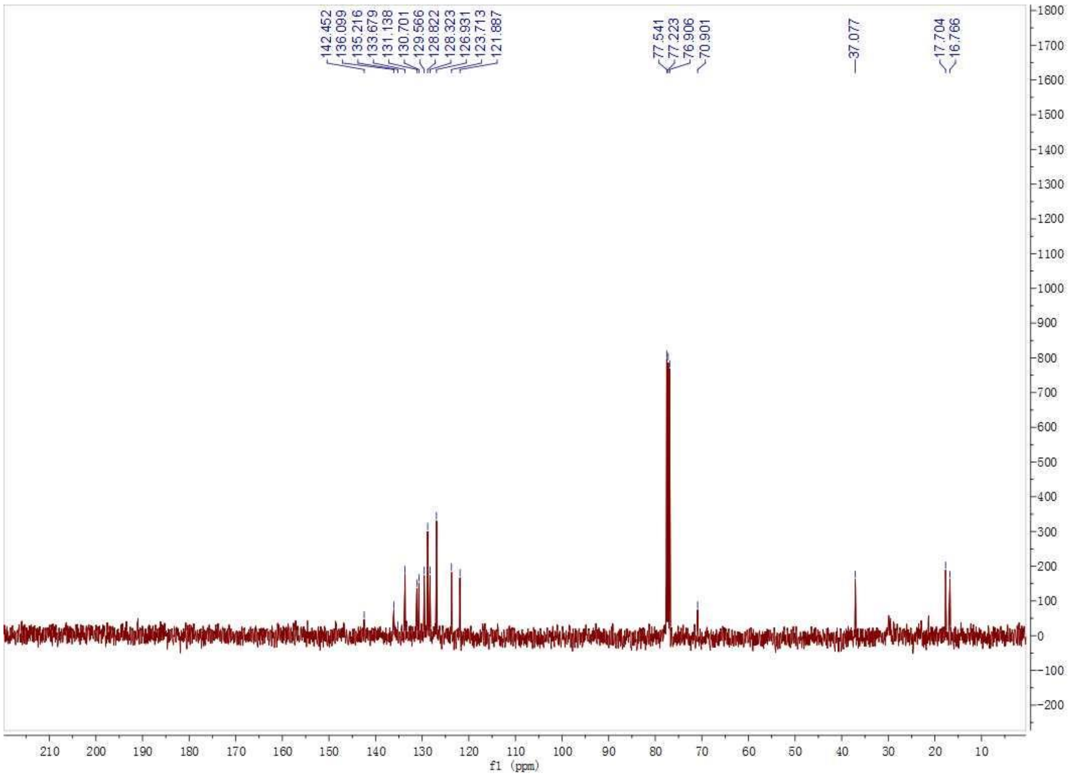

Supplementary Figure 73: ^{13}C NMR spectrum of compound **3qa** in $CDCl_3$

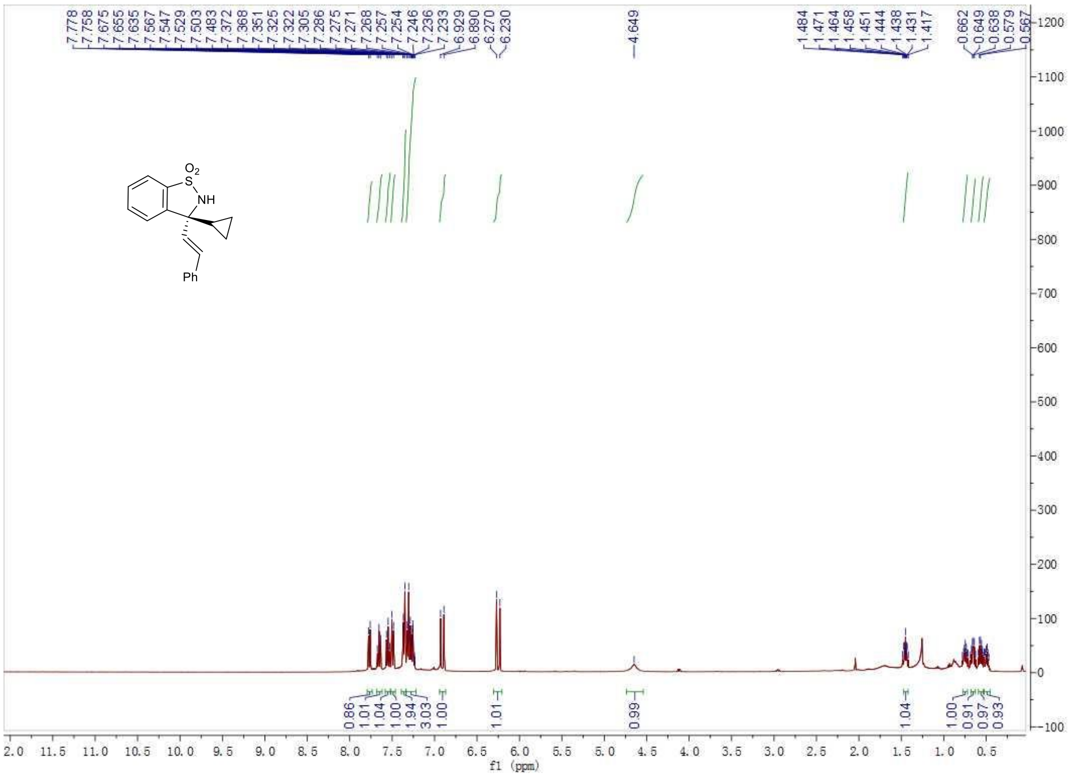

Supplementary Figure 74: ^{1}H NMR spectrum of compound **3ra** in $CDCl_3$

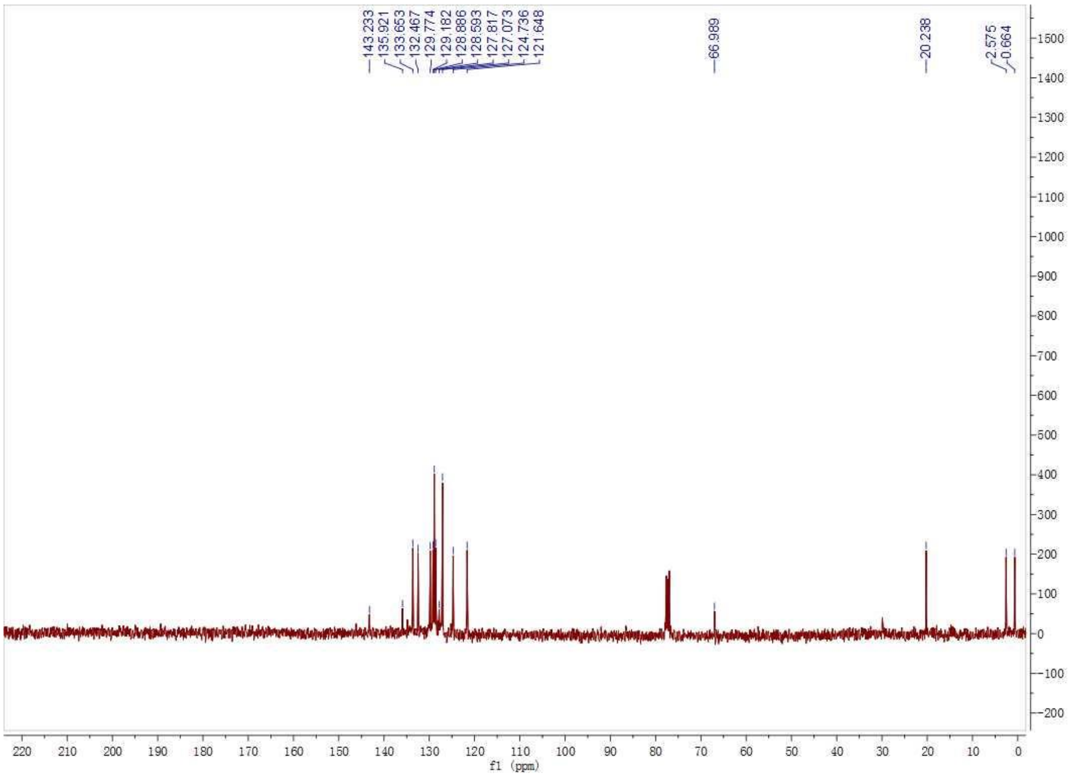

Supplementary Figure 75: ^{13}C NMR spectrum of compound **3ra** in $CDCl_3$

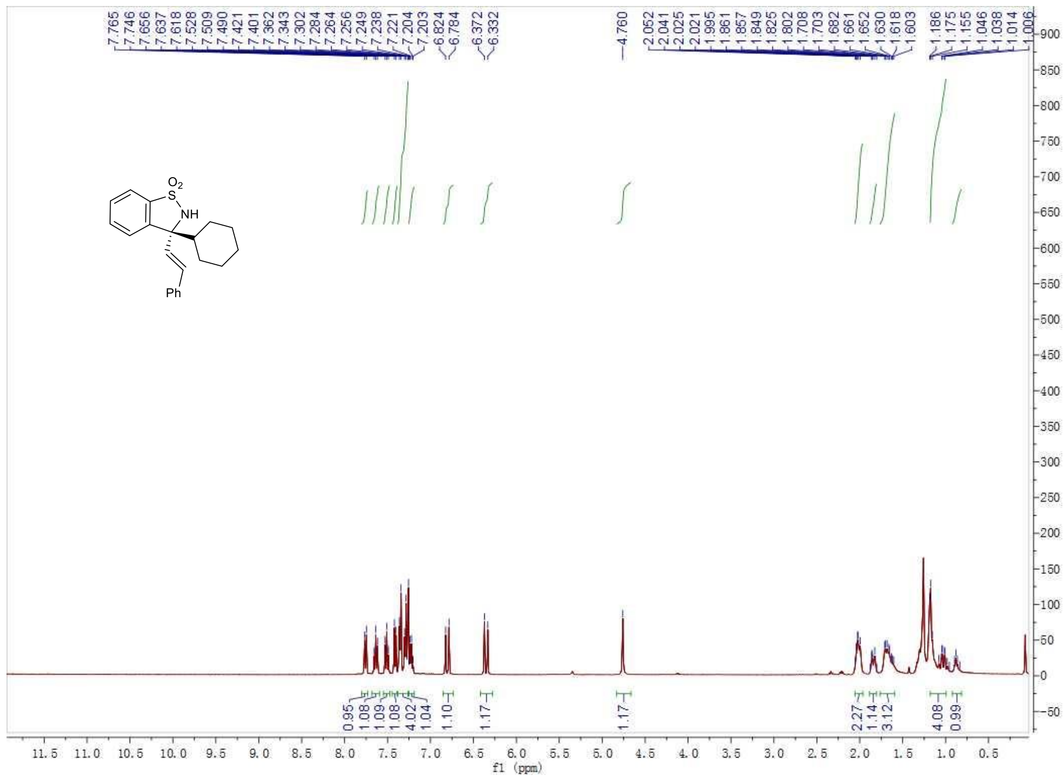

Supplementary Figure 76: ^{1}H NMR spectrum of compound **3sa** in $CDCl_3$

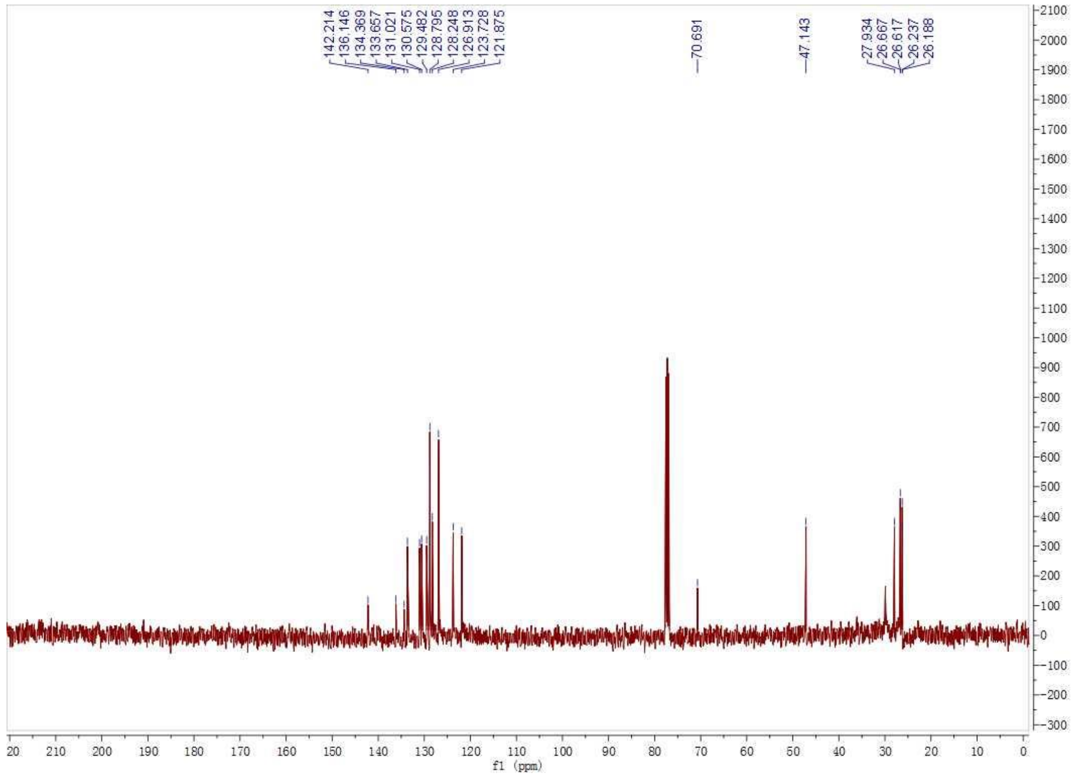

Supplementary Figure 77: ^{13}C NMR spectrum of compound **3sa** in $CDCl_3$

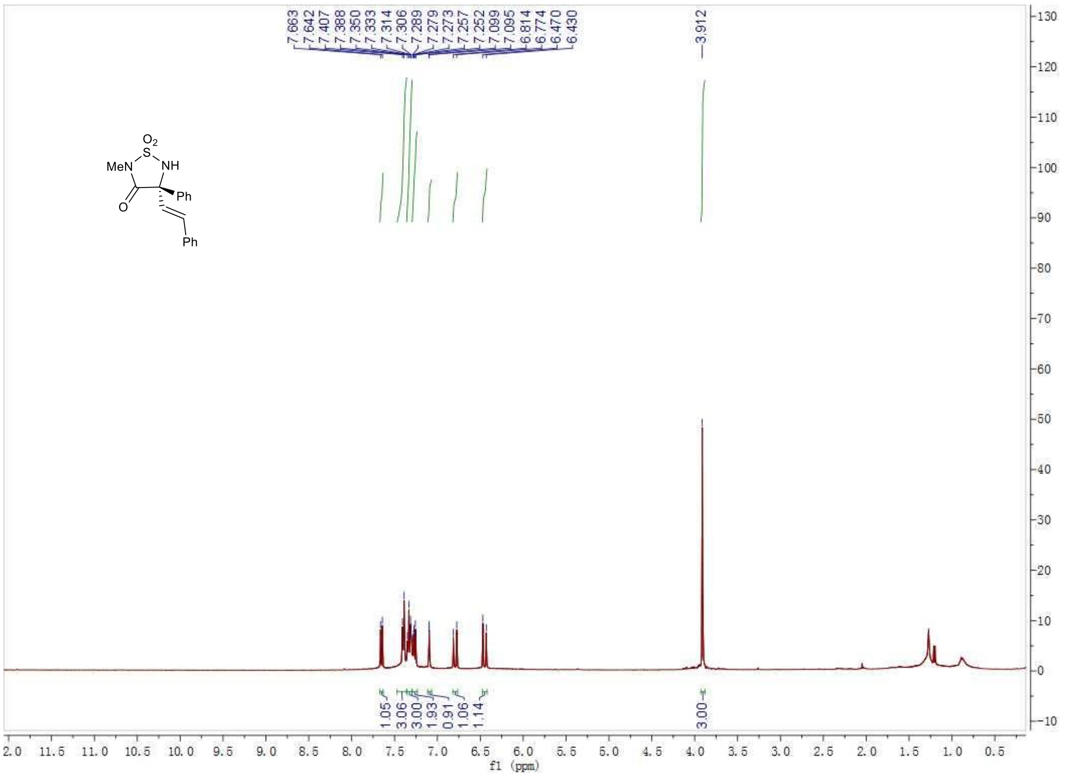

Supplementary Figure 78: ^{1}H NMR spectrum of compound **3ta** in $CDCl_3$

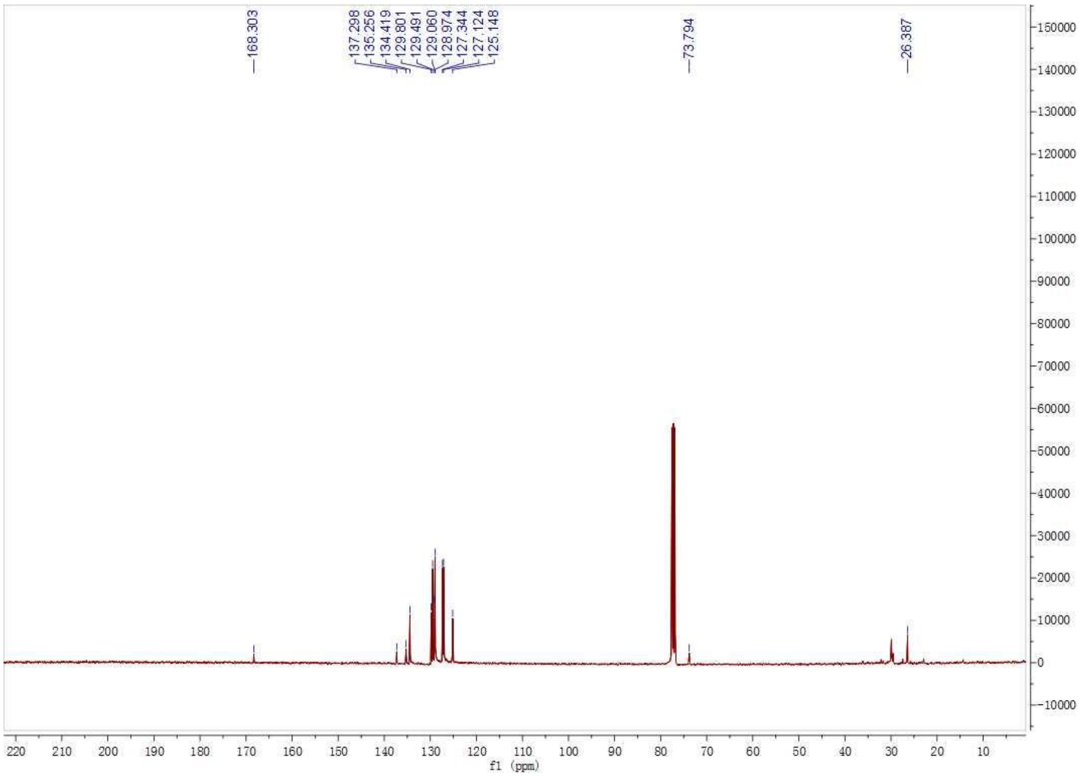

Supplementary Figure 79: ^{13}C NMR spectrum of compound **3ta** in $CDCl_3$

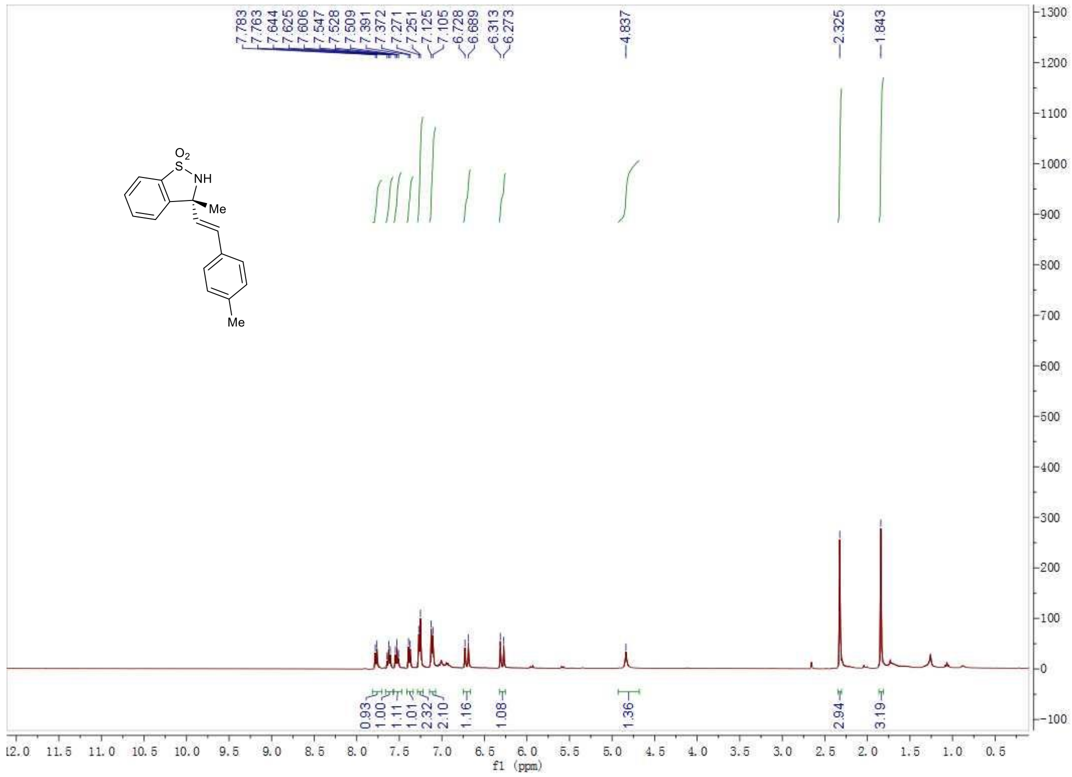

Supplementary Figure 80: ^{1}H NMR spectrum of compound **3ib** in $CDCl_3$

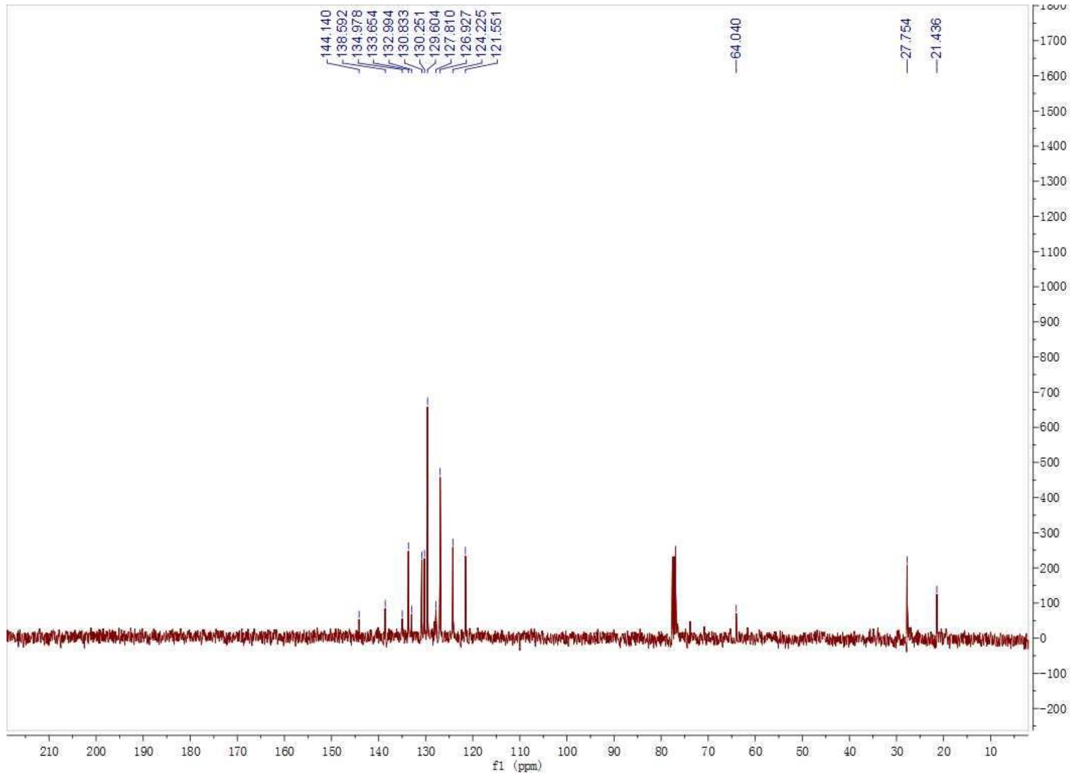

Supplementary Figure 81: ^{13}C NMR spectrum of compound **3ib** in $CDCl_3$

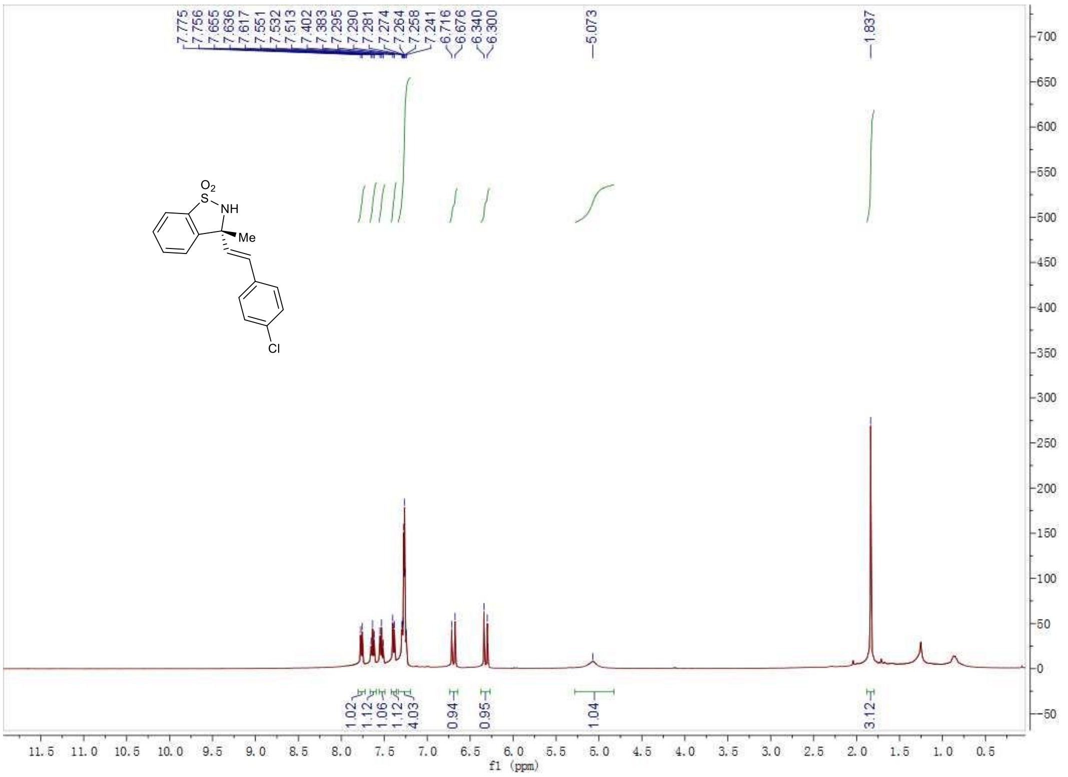

Supplementary Figure 82: ^{1}H NMR spectrum of compound **3ic** in $CDCl_3$

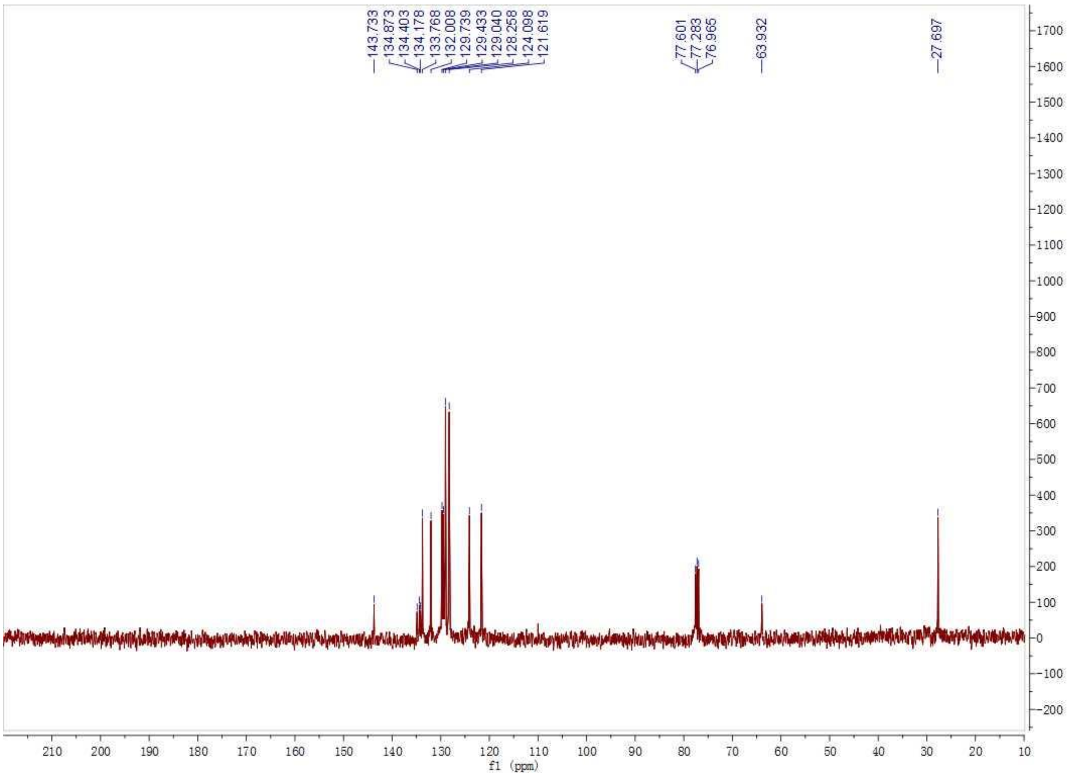

Supplementary Figure 83: ^{13}C NMR spectrum of compound **3ic** in $CDCl_3$

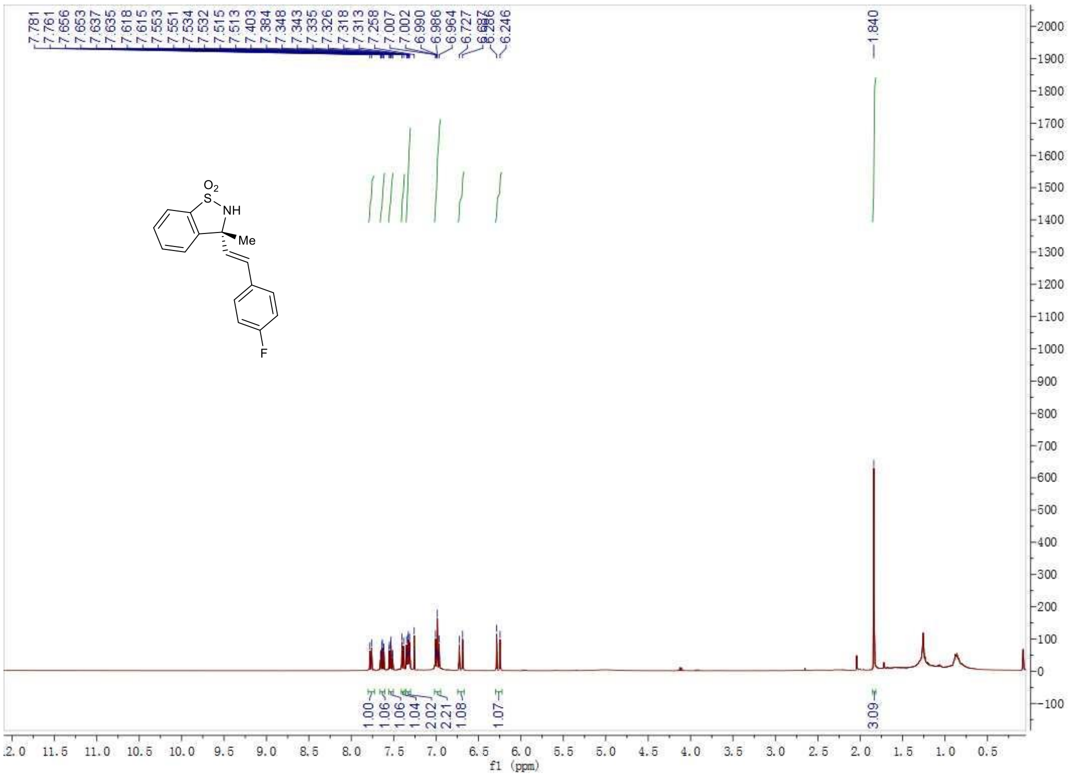

Supplementary Figure 84: ^{1}H NMR spectrum of compound **3id** in $CDCl_3$

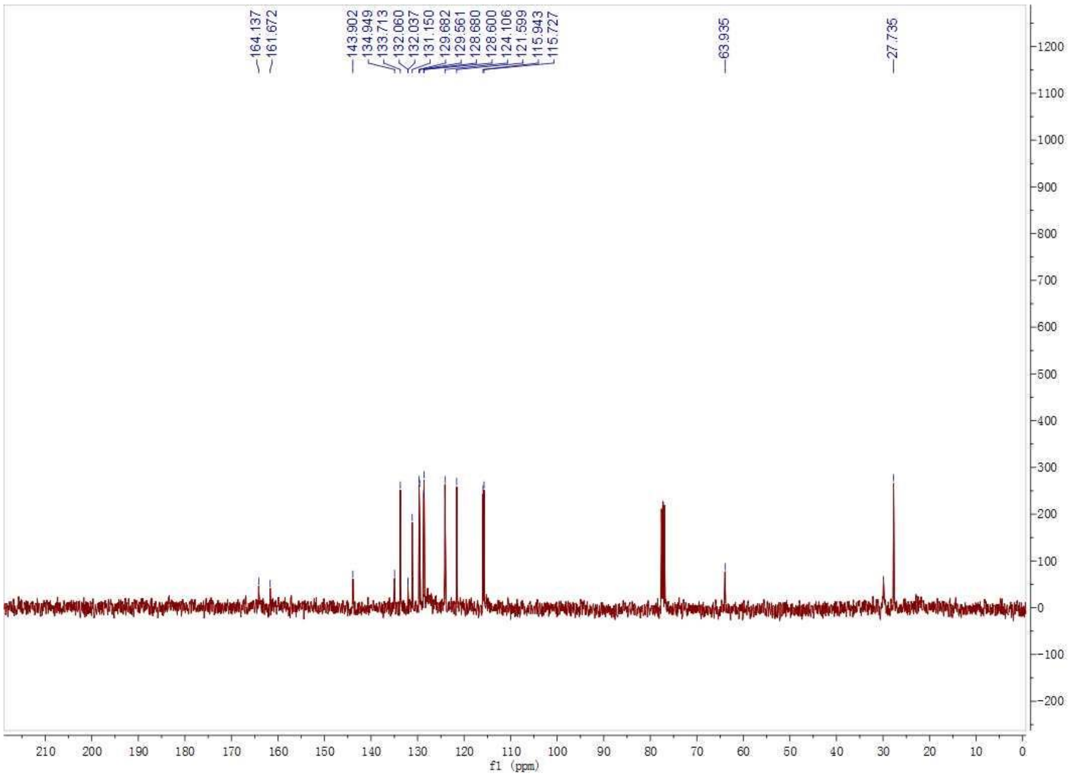

Supplementary Figure 85: ^{13}C NMR spectrum of compound **3id** in $CDCl_3$

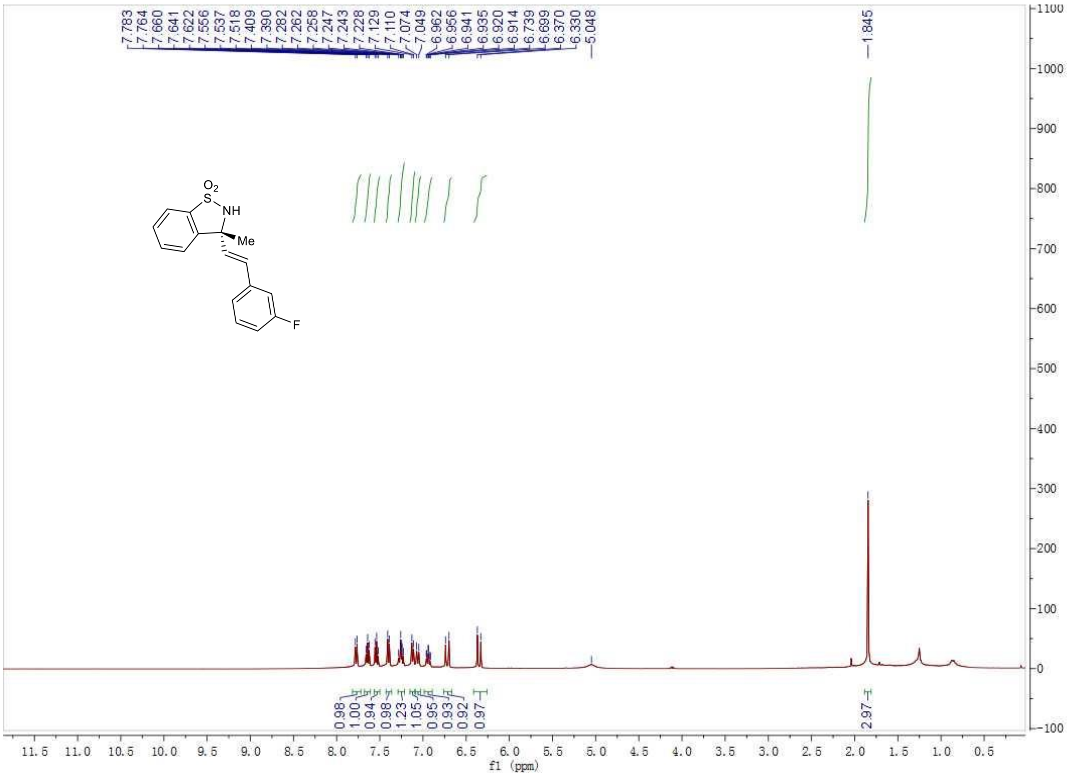

Supplementary Figure 86: ^{1}H NMR spectrum of compound **3ie** in $CDCl_3$

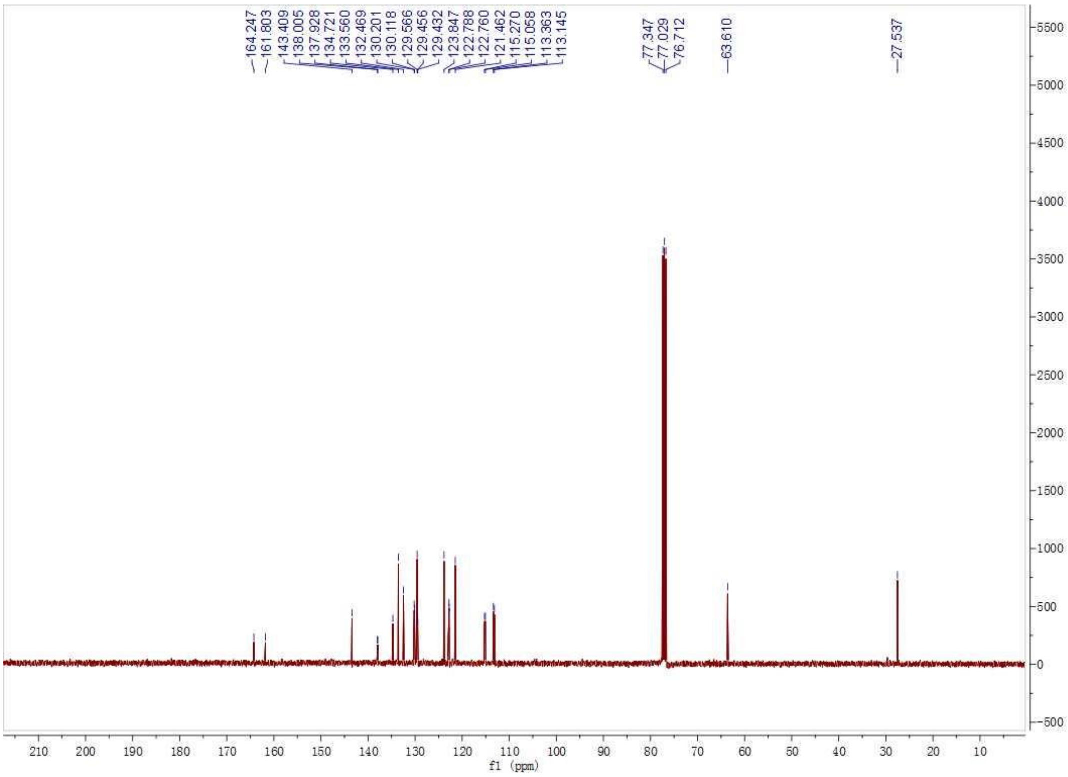

Supplementary Figure 87: ^{13}C NMR spectrum of compound **3ie** in $CDCl_3$

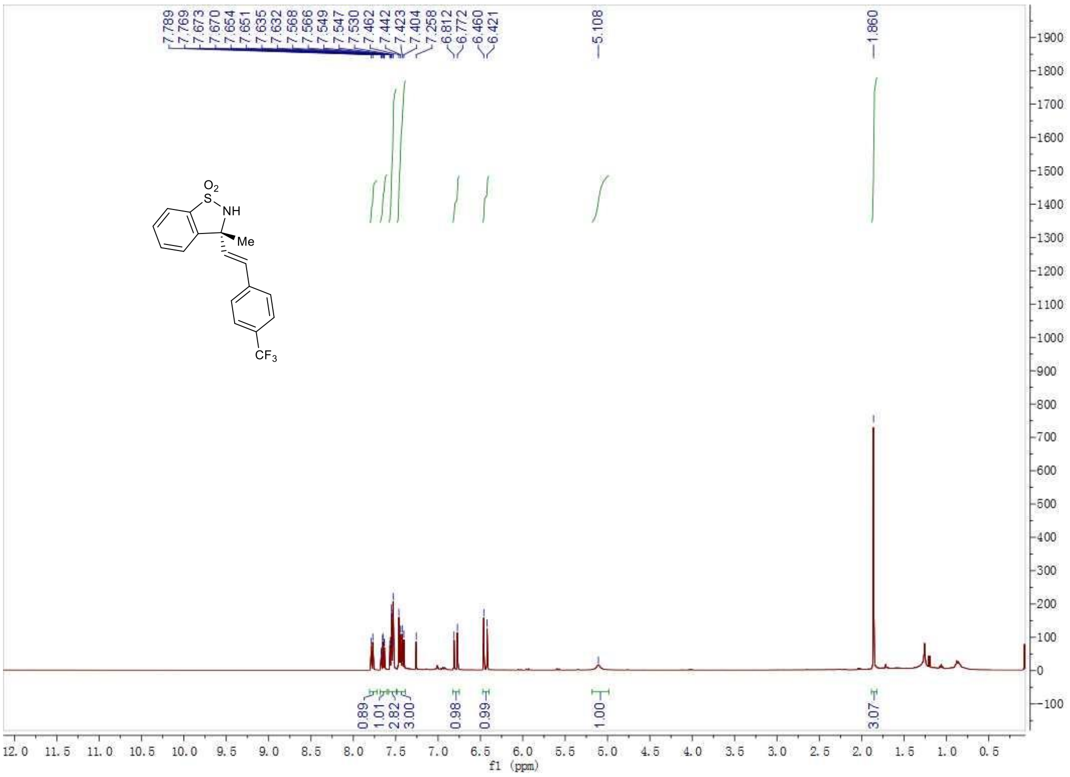

Supplementary Figure 88: ^{1}H NMR spectrum of compound **3if** in $CDCl_3$

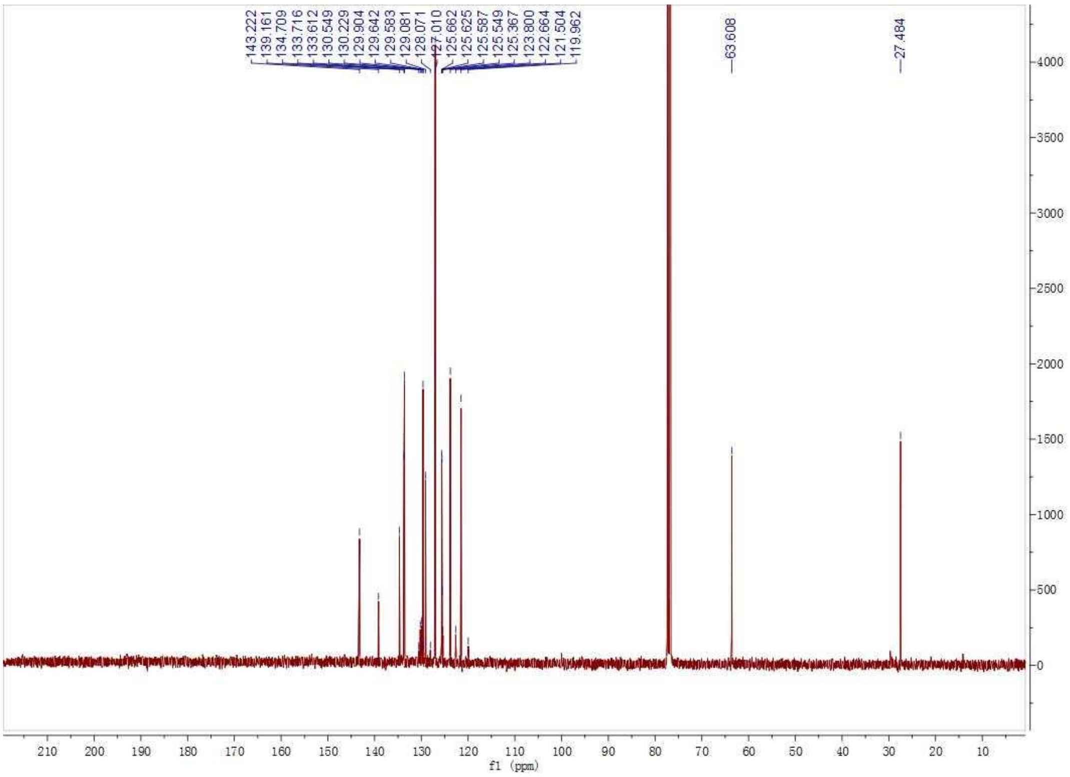

Supplementary Figure 89: ^{13}C NMR spectrum of compound **3if** in $CDCl_3$

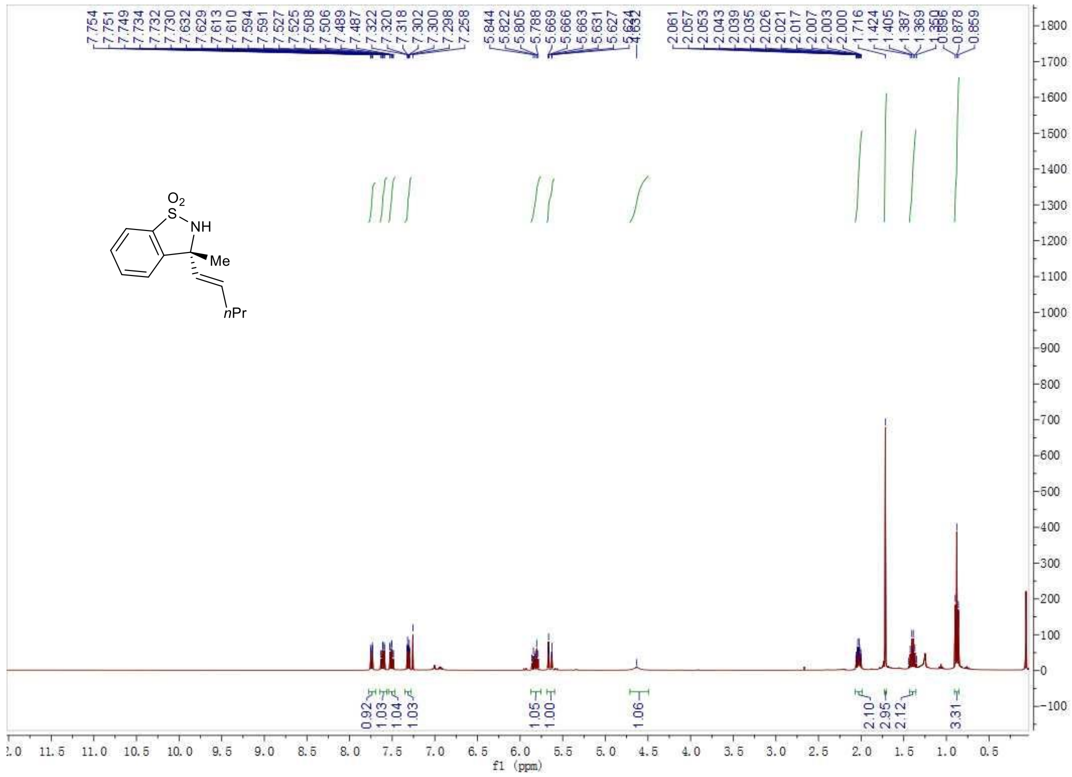

Supplementary Figure 90: ^{1}H NMR spectrum of compound **3ig** in $CDCl_3$

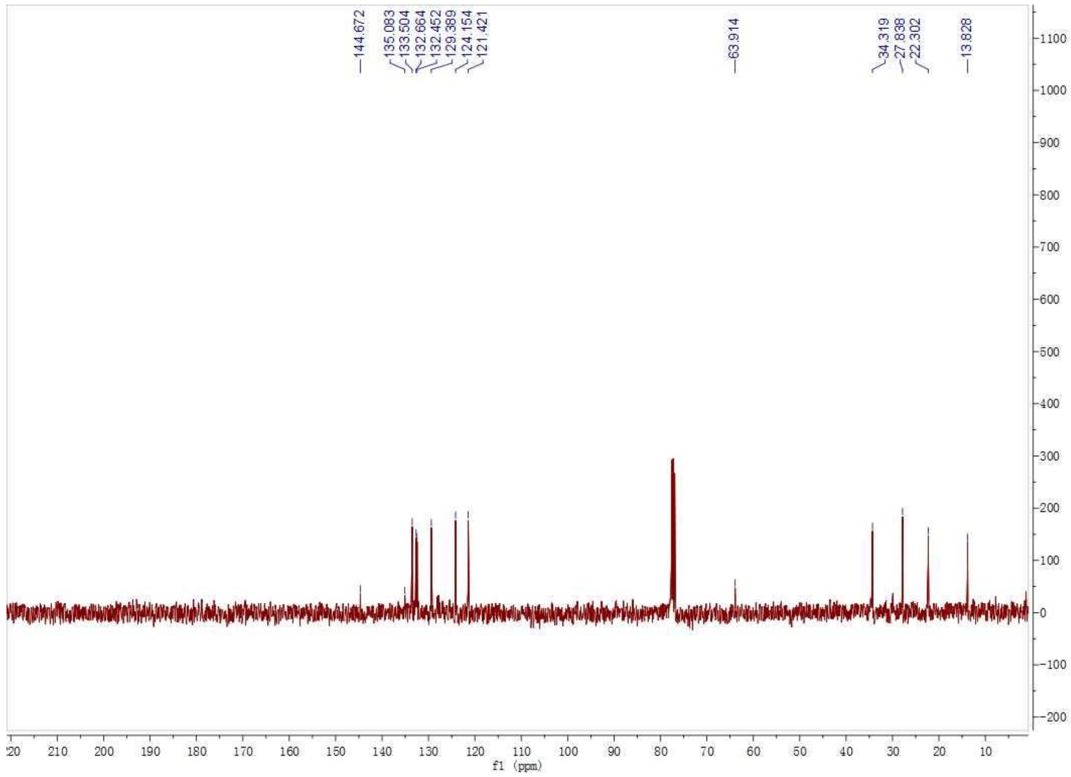

Supplementary Figure 91: ^{13}C NMR spectrum of compound **3ig** in $CDCl_3$

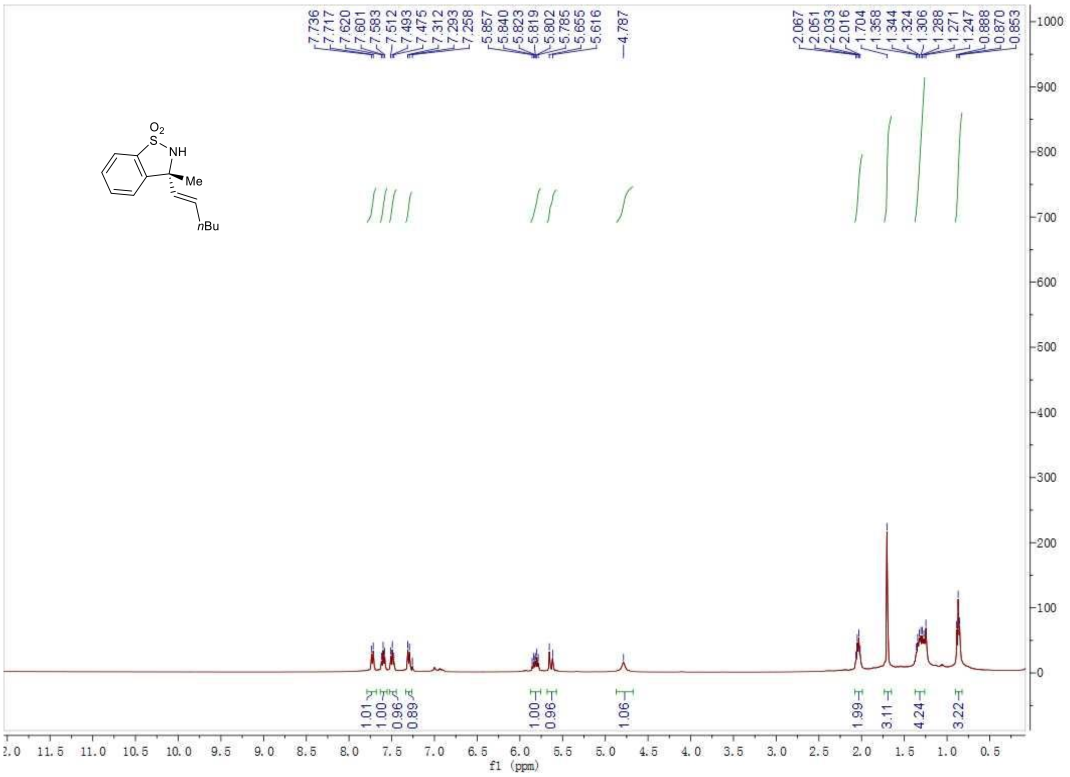

Supplementary Figure 92: ^{1}H NMR spectrum of compound **3ih** in $CDCl_3$

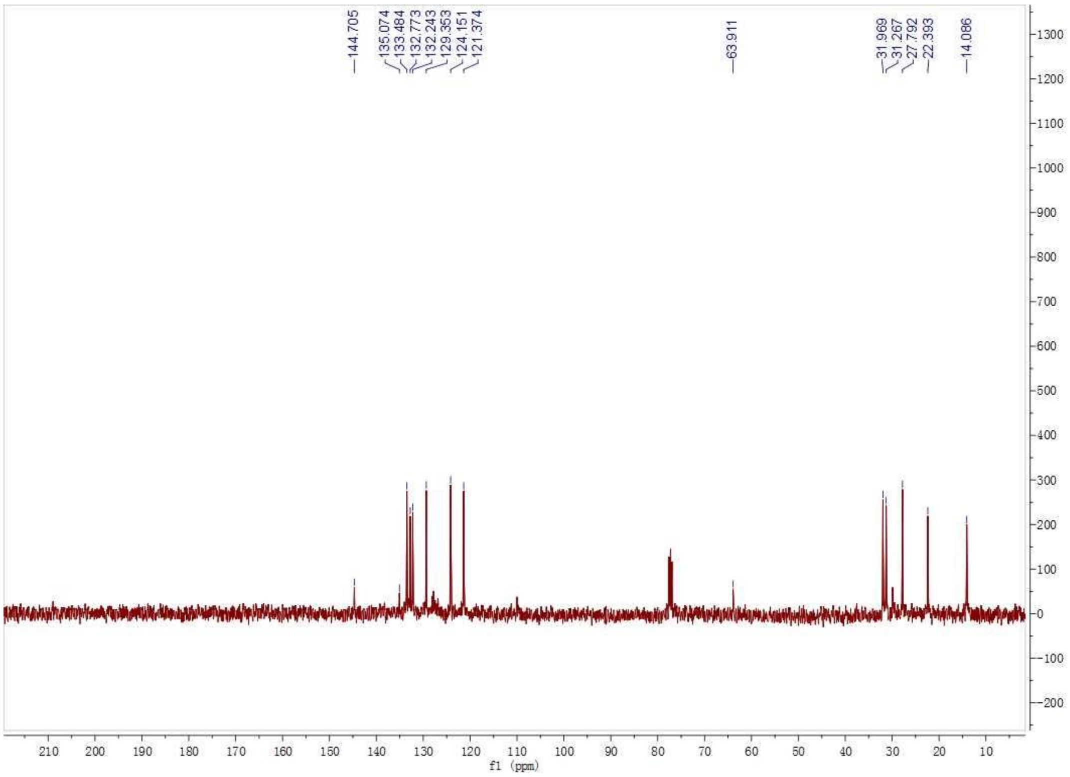

Supplementary Figure 93: ^{13}C NMR spectrum of compound **3ih** in $CDCl_3$

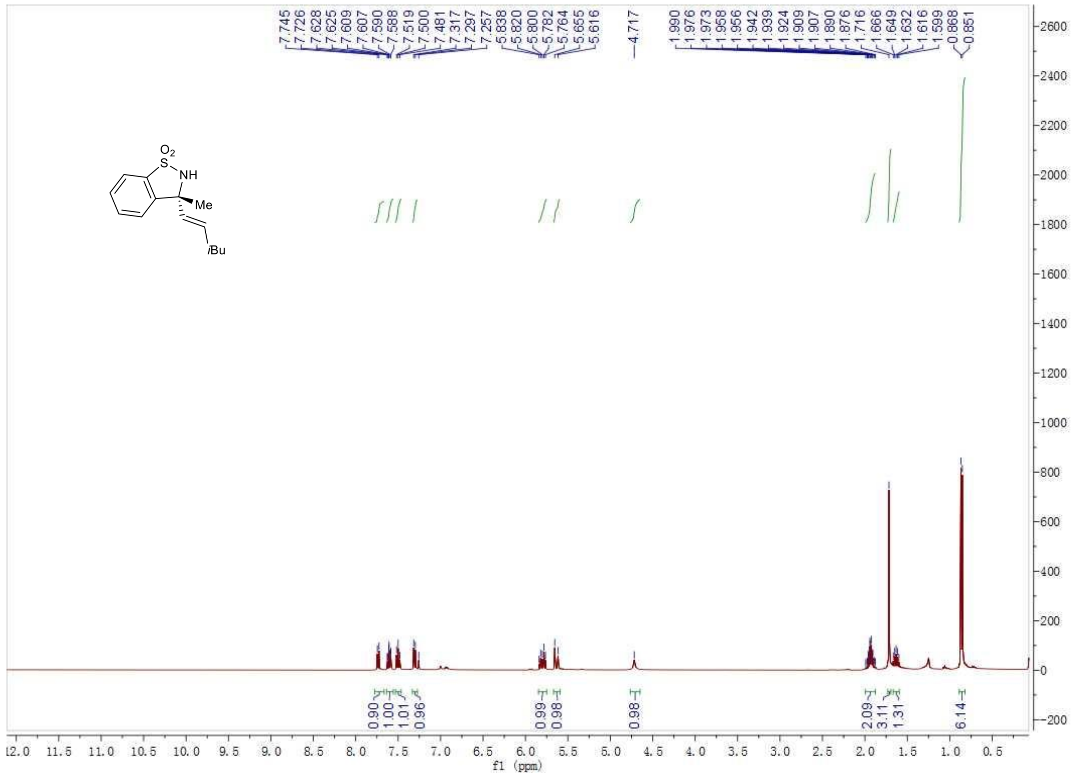

Supplementary Figure 94: ^{1}H NMR spectrum of compound **3ii** in $CDCl_3$

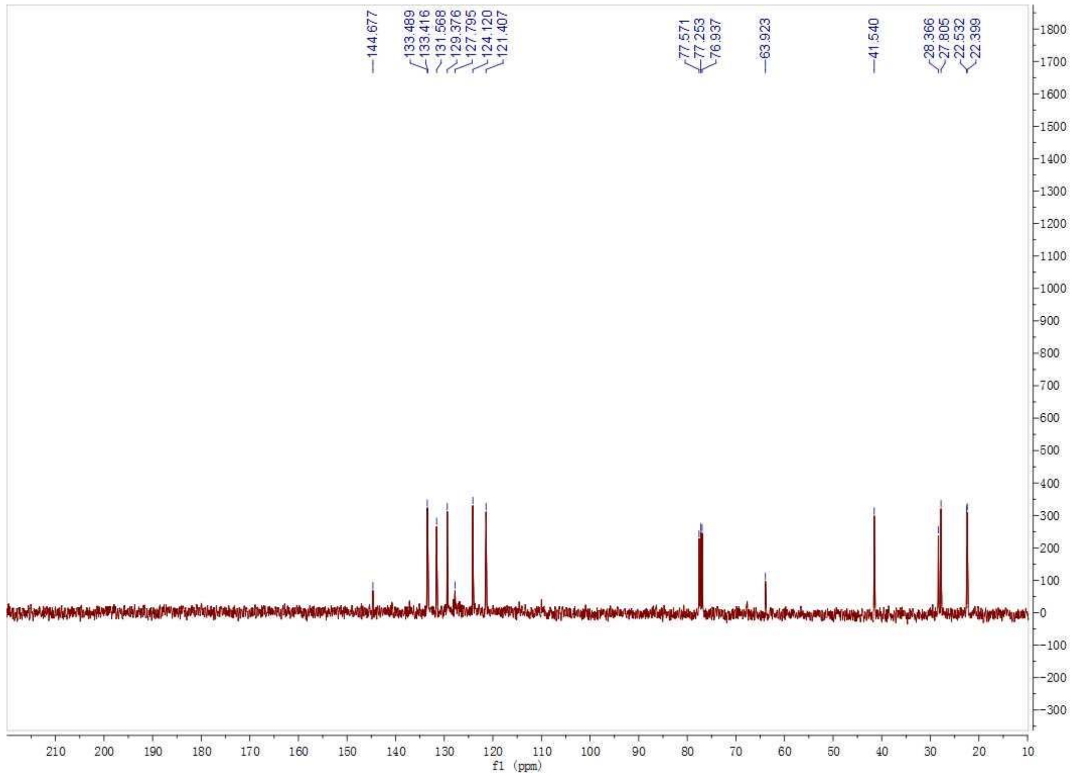

Supplementary Figure 95: ^{13}C NMR spectrum of compound **3ii** in $CDCl_3$

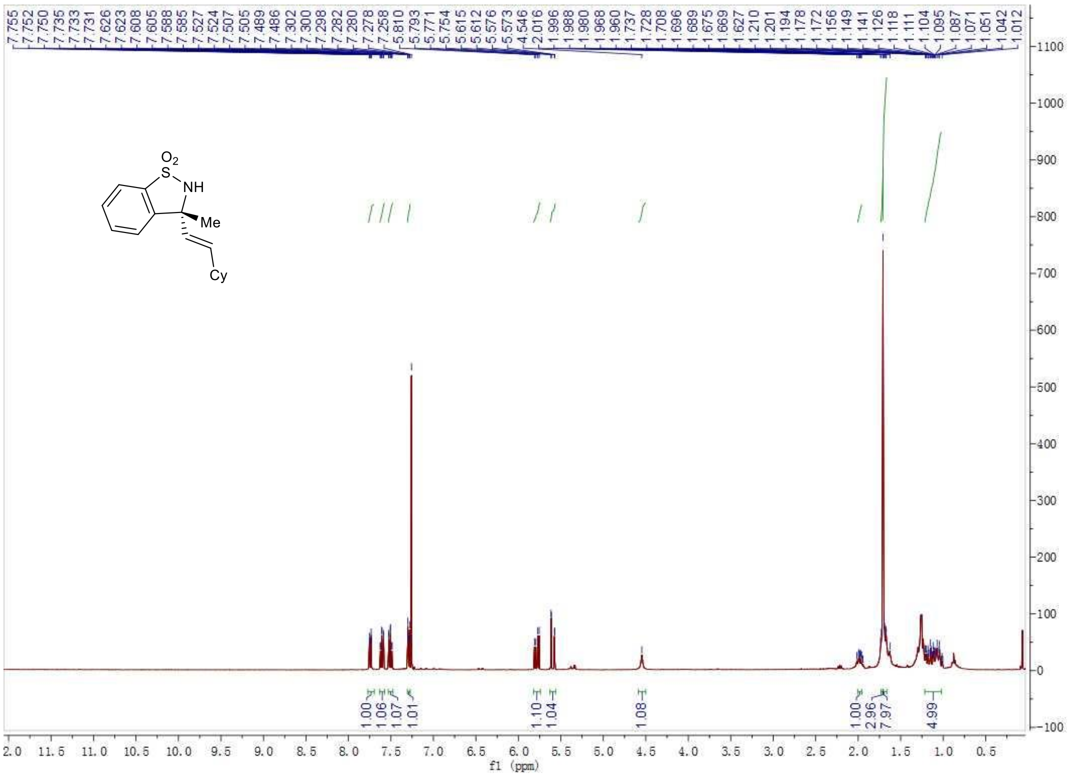

Supplementary Figure 96: ^{1}H NMR spectrum of compound **3ij** in $CDCl_3$

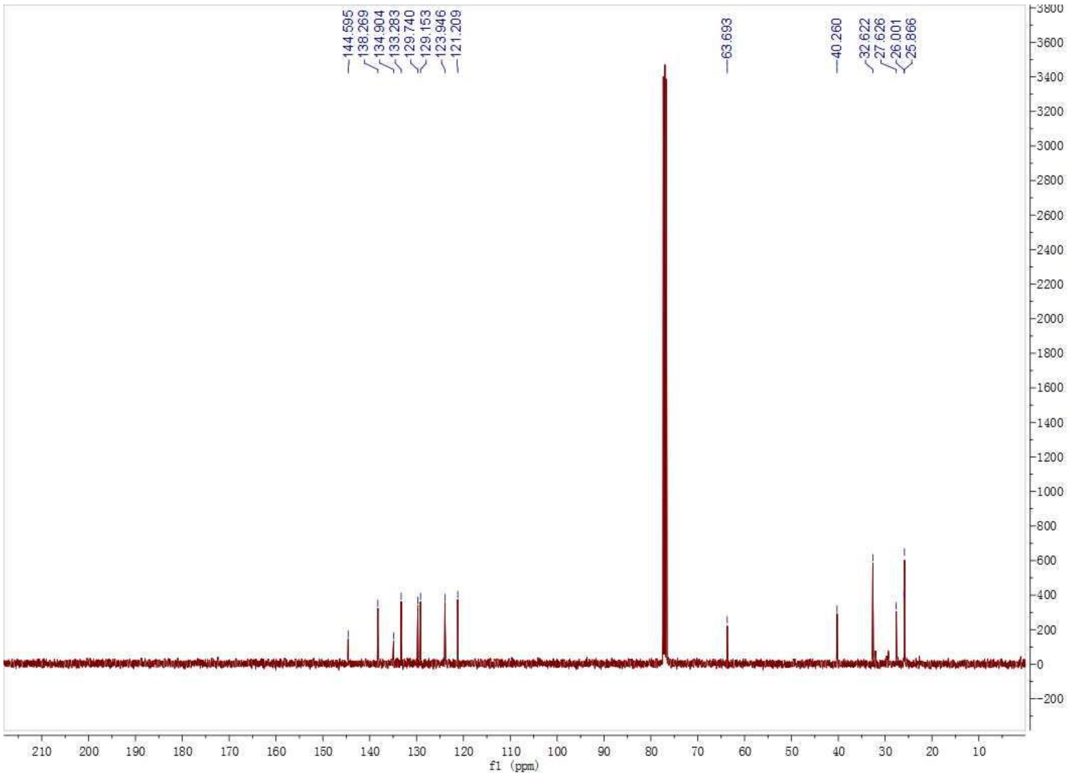

Supplementary Figure 97: ^{13}C NMR spectrum of compound **3ij** in $CDCl_3$

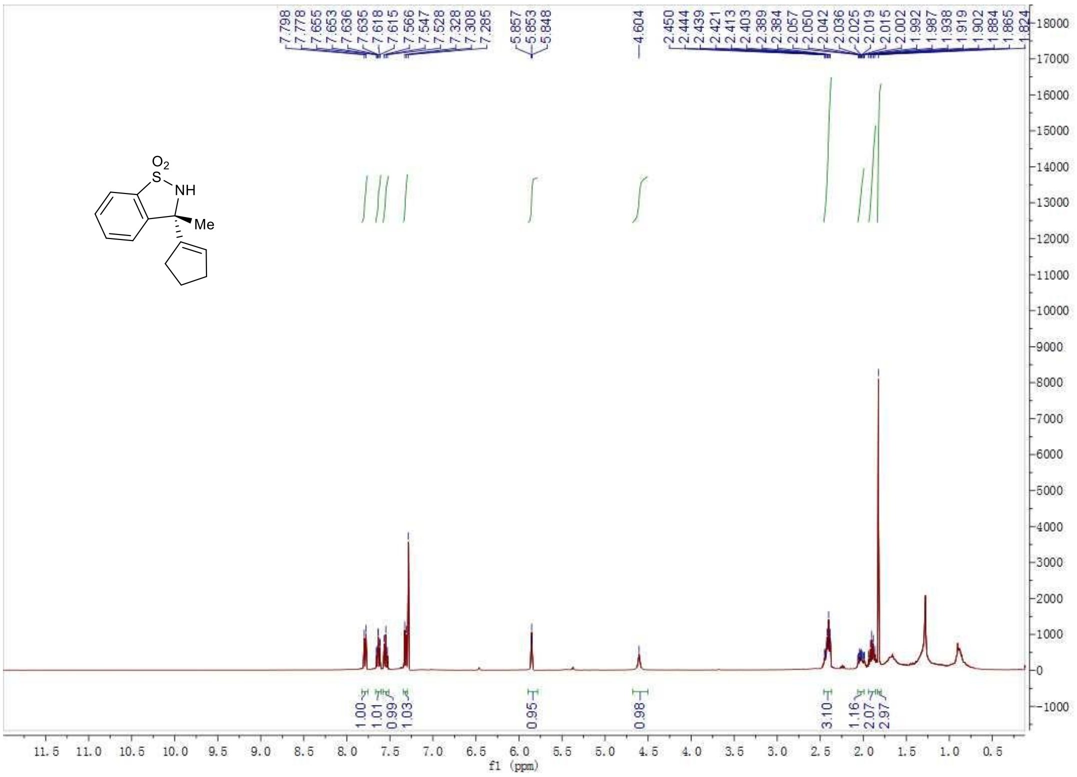

Supplementary Figure 98: ^{1}H NMR spectrum of compound **3ik** in $CDCl_3$

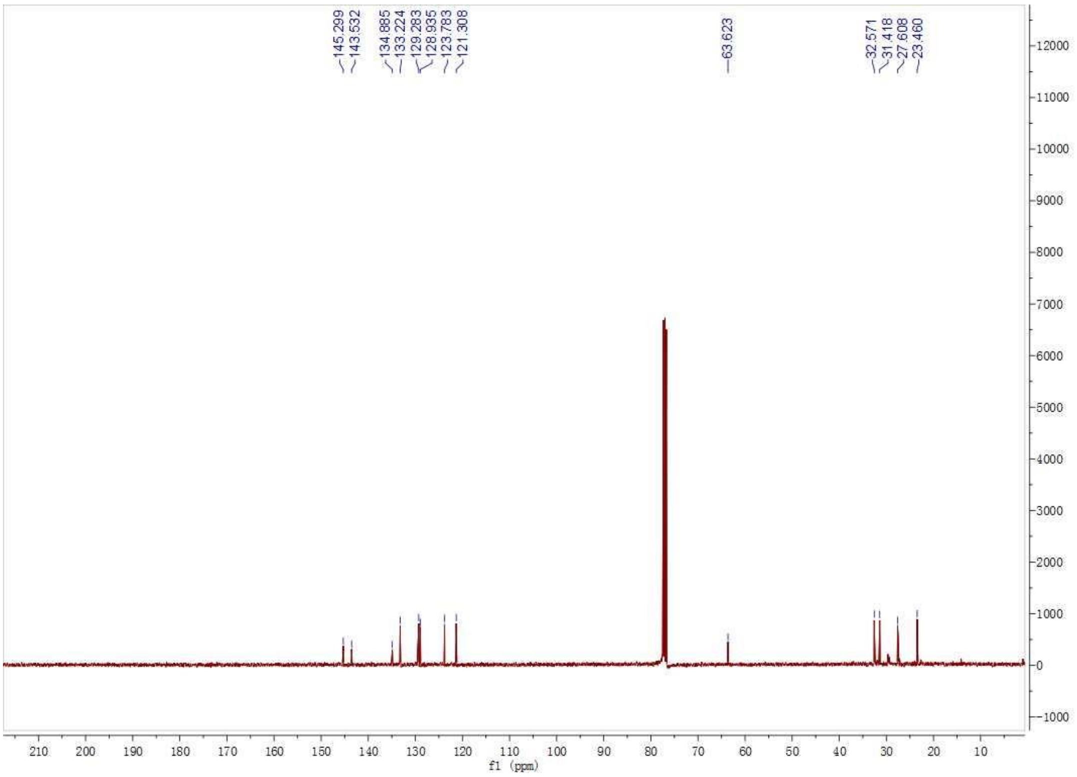

Supplementary Figure 99: ^{13}C NMR spectrum of compound **3ik** in $CDCl_3$

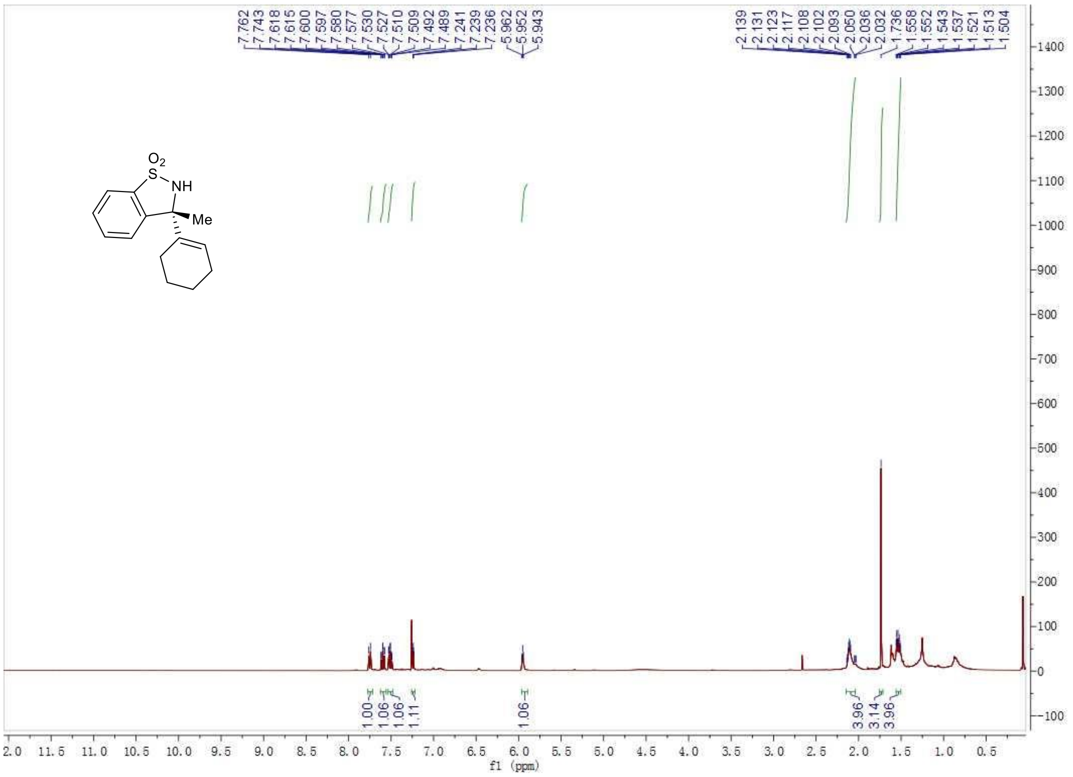

Supplementary Figure 100: ^{1}H NMR spectrum of compound **3il** in $CDCl_3$

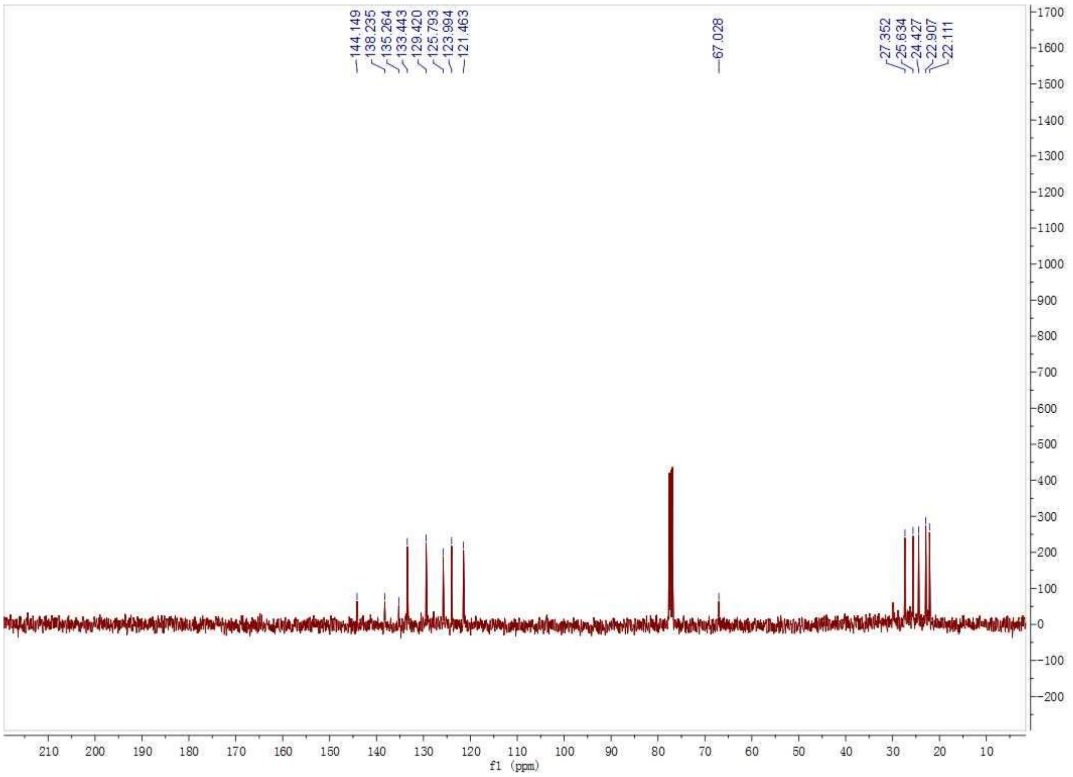

Supplementary Figure 101: ^{13}C NMR spectrum of compound **3il** in $CDCl_3$

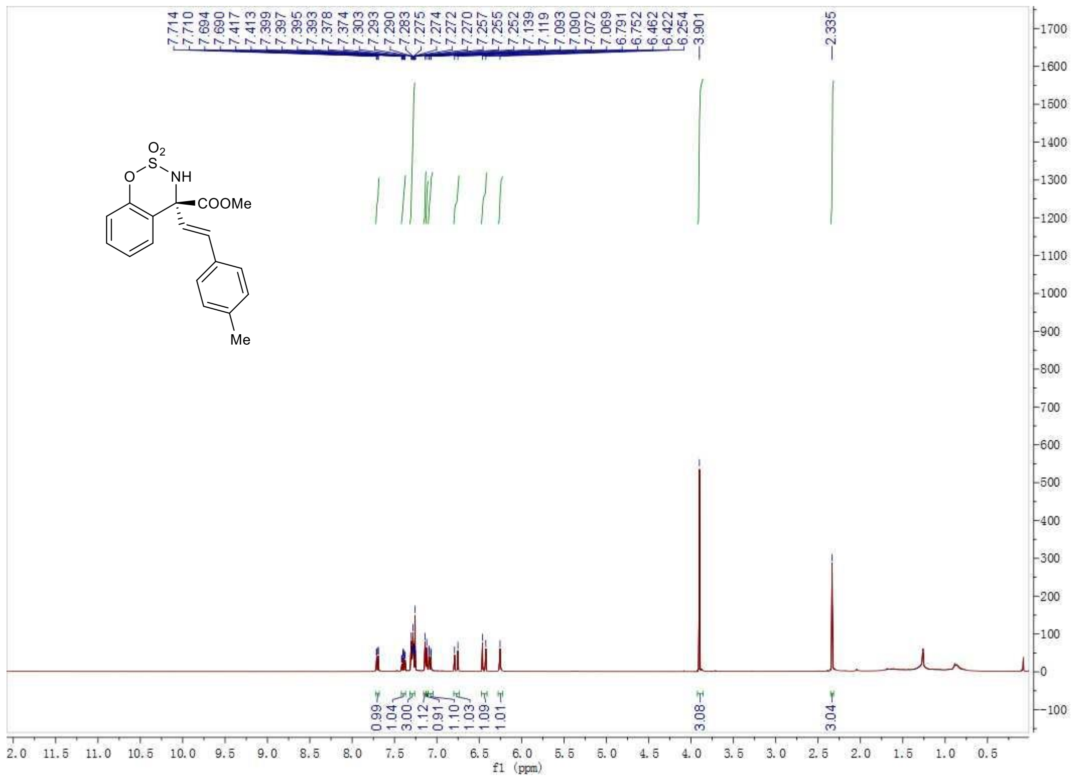

Supplementary Figure 102: ^{1}H NMR spectrum of compound **3ab** in $CDCl_3$

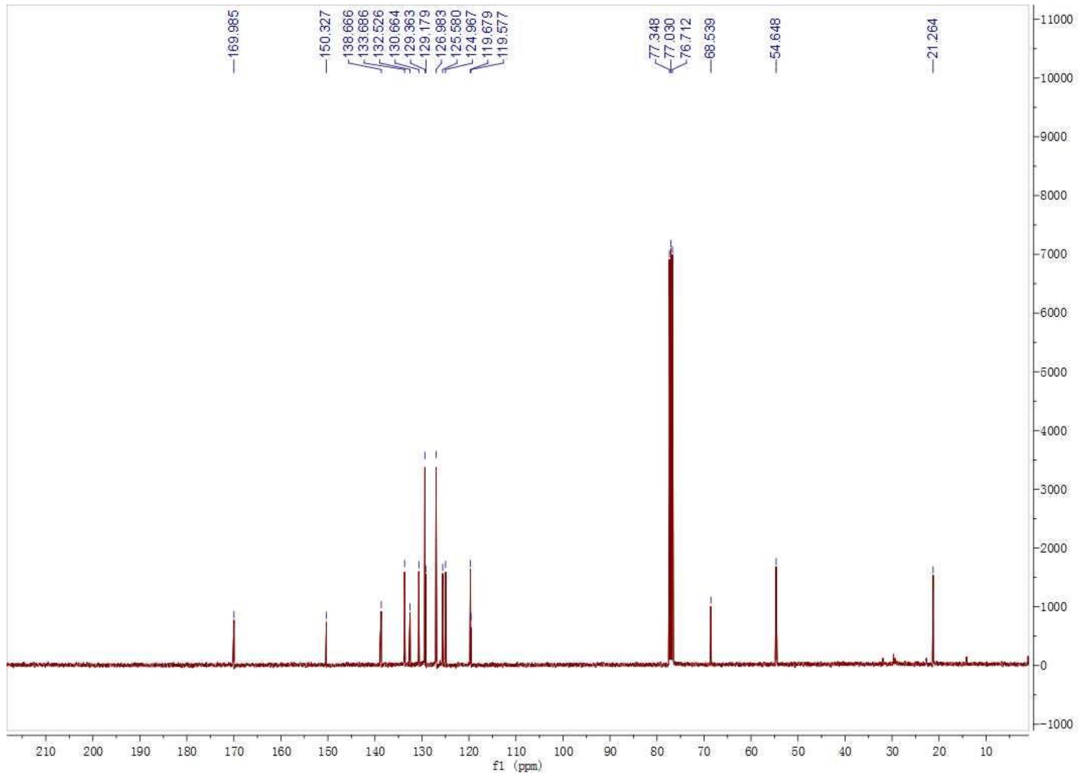

Supplementary Figure 103: ^{13}C NMR spectrum of compound **3ab** in $CDCl_3$

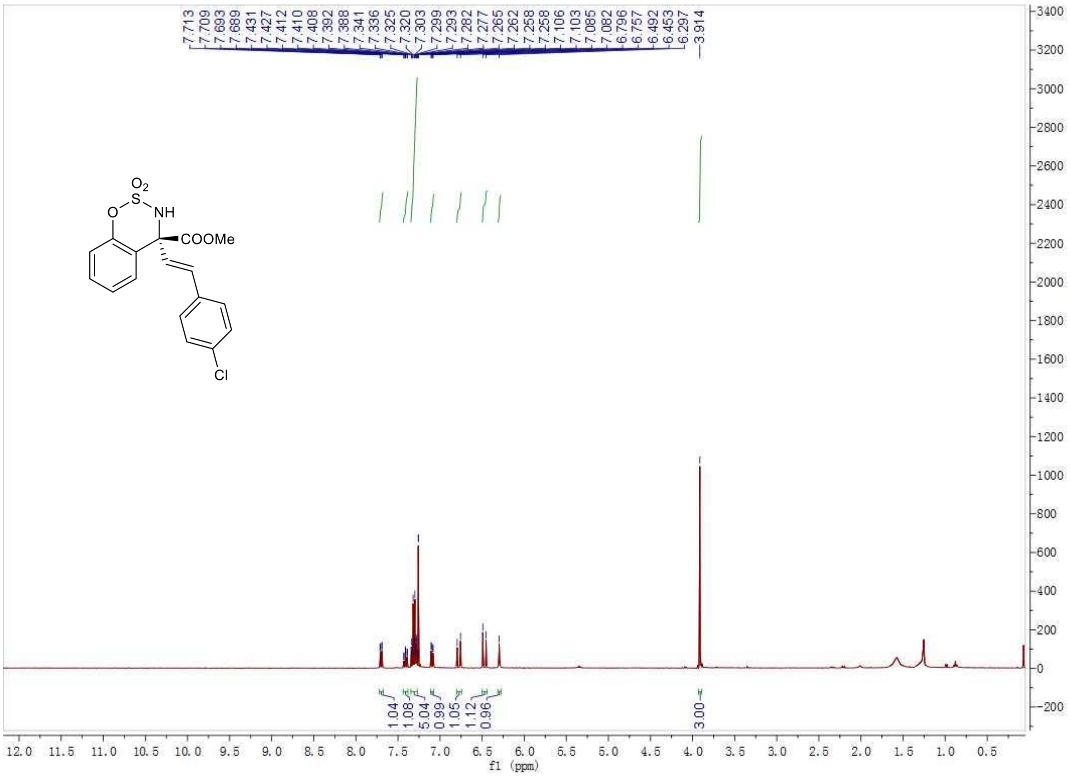

Supplementary Figure 104: ^{1}H NMR spectrum of compound **3ac** in $CDCl_3$

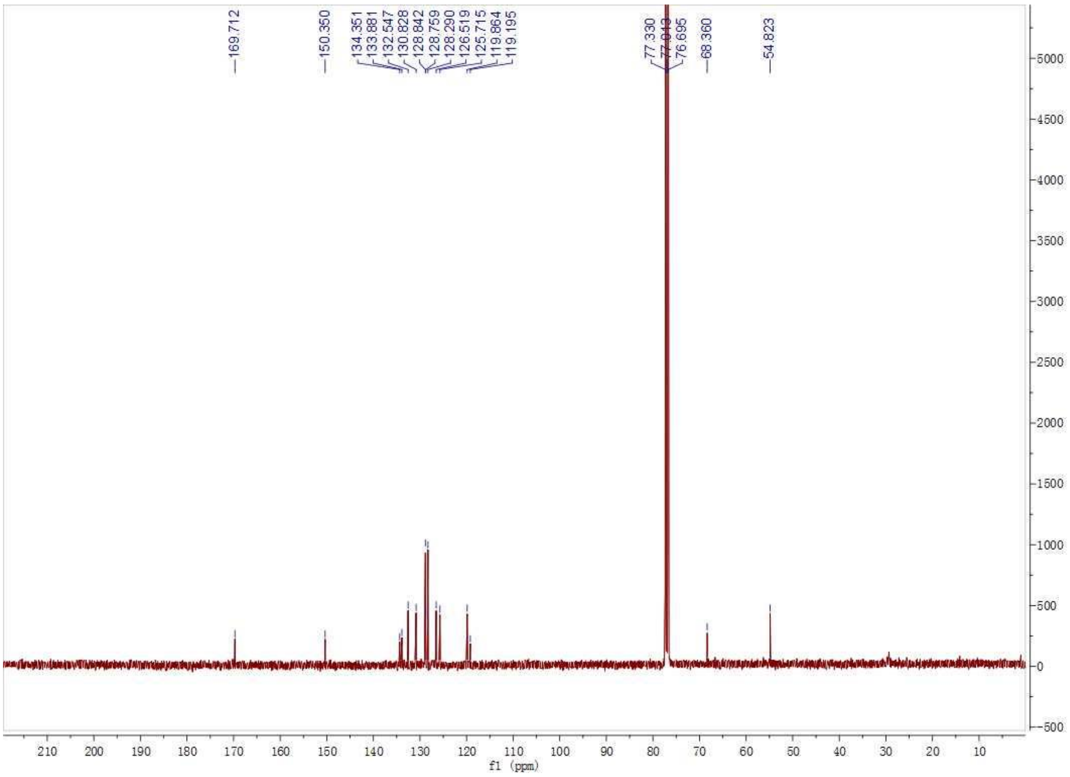

Supplementary Figure 105: ^{13}C NMR spectrum of compound **3ac** in $CDCl_3$

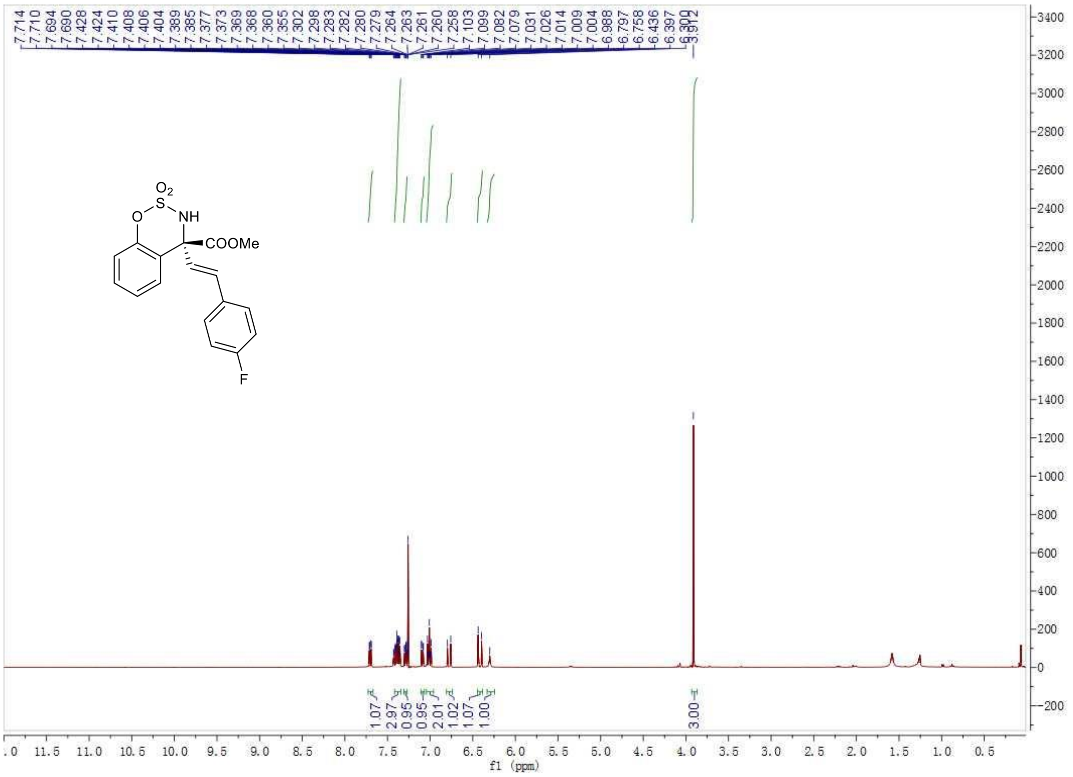

Supplementary Figure 106: ^{1}H NMR spectrum of compound **3ad** in $CDCl_3$

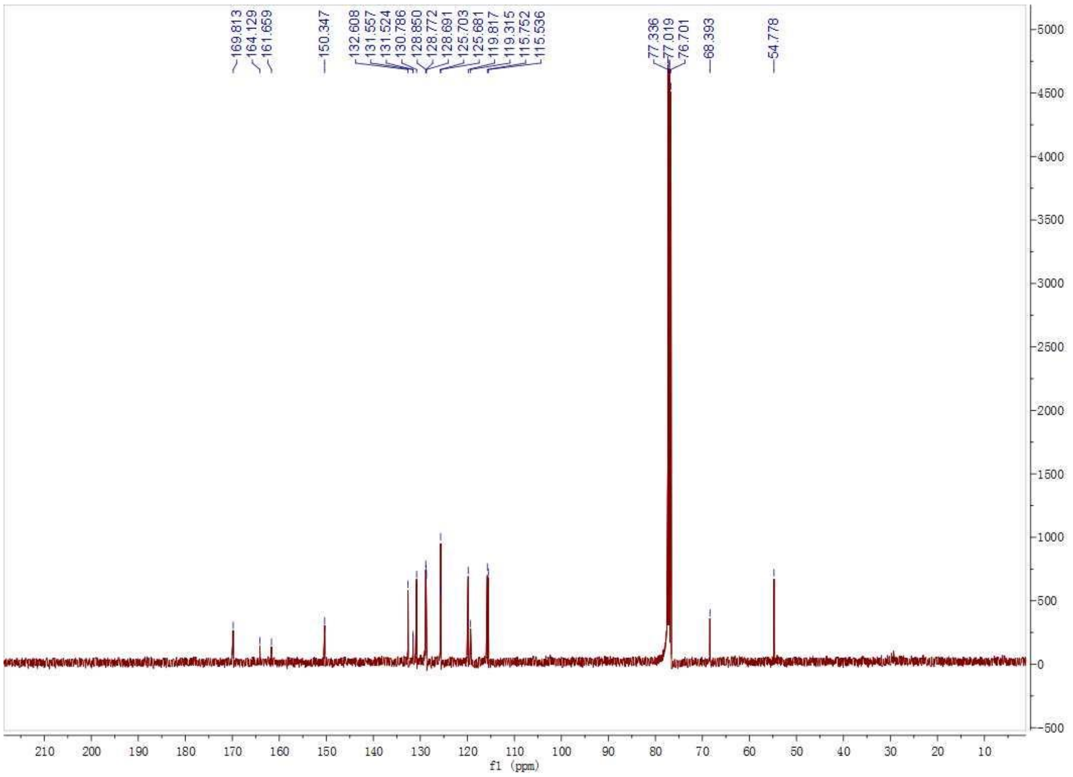

Supplementary Figure 107: ^{13}C NMR spectrum of compound **3ad** in $CDCl_3$

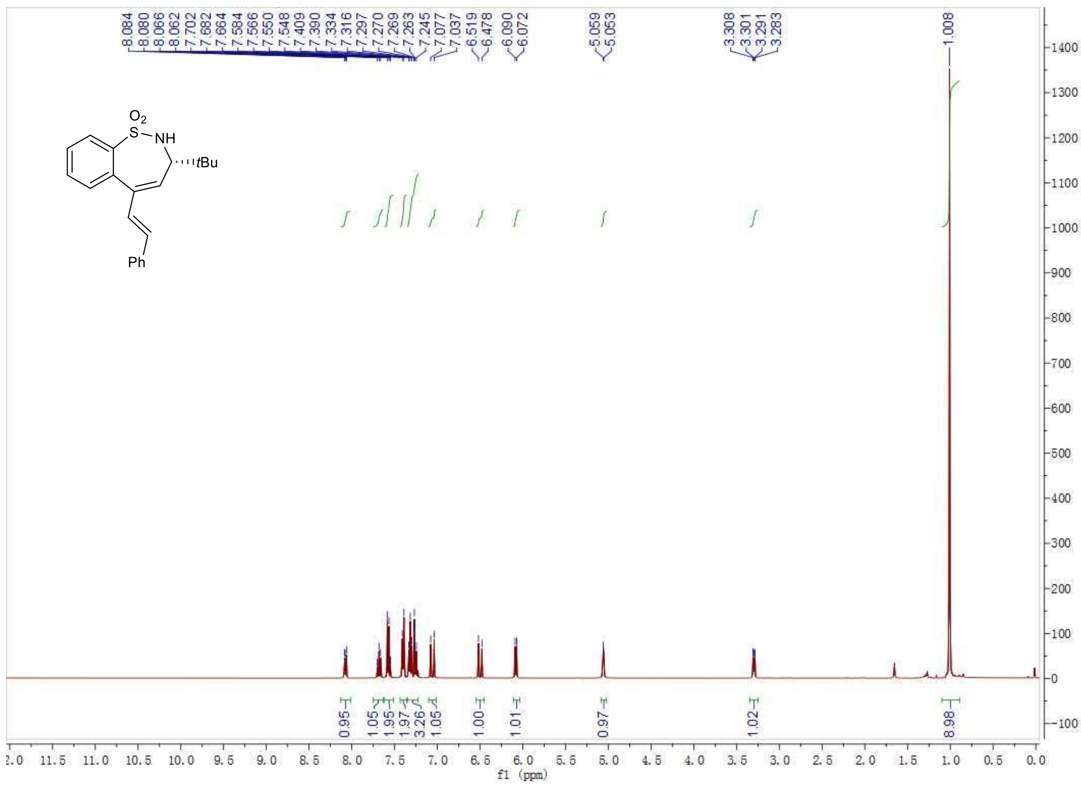

Supplementary Figure 108: ^{1}H NMR spectrum of compound **5a** in $CDCl_3$

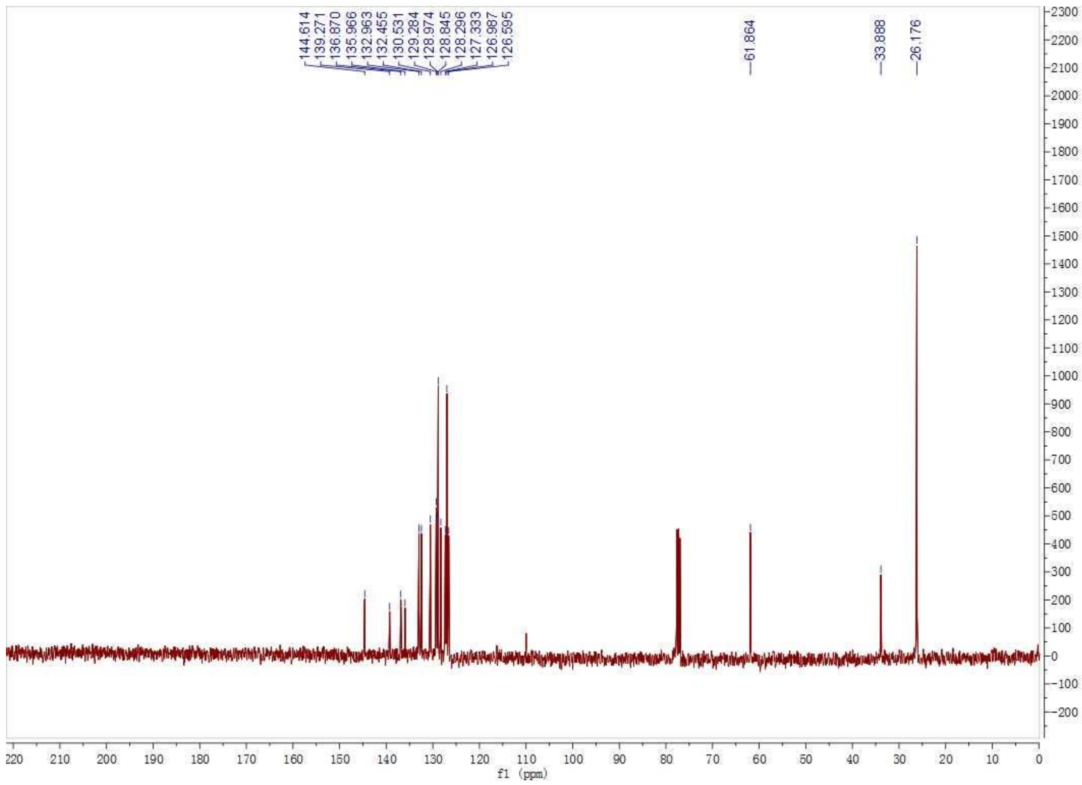

Supplementary Figure 109: ^{13}C NMR spectrum of compound **5a** in $CDCl_3$

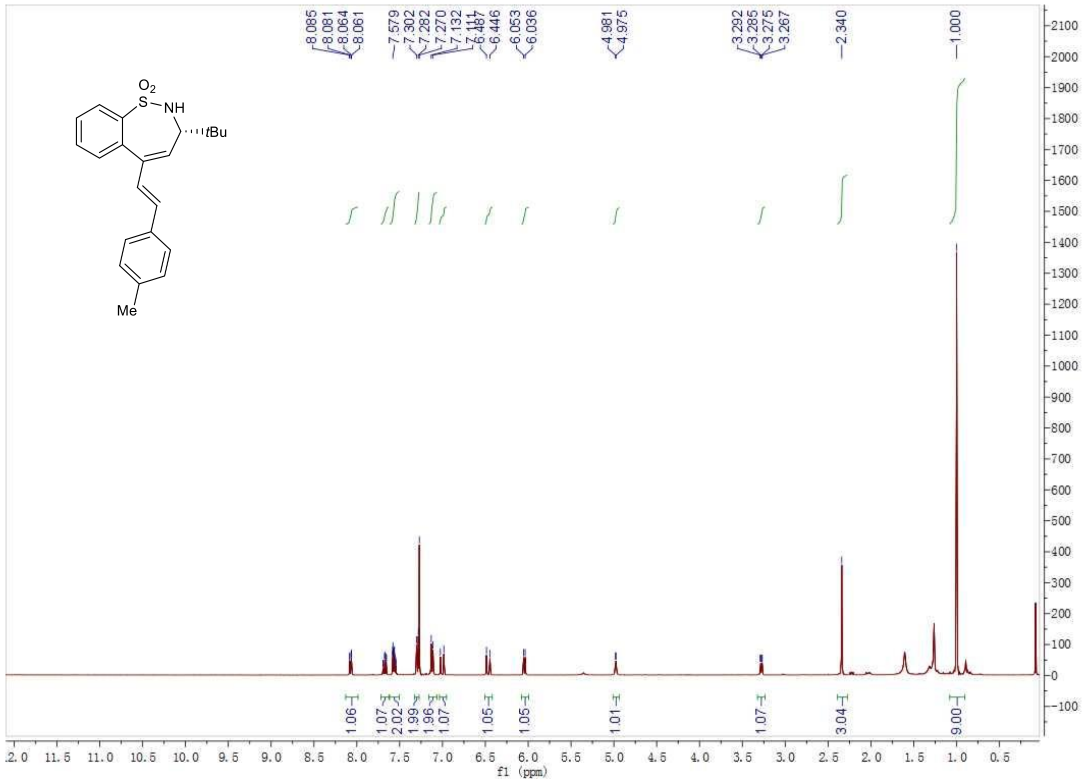

Supplementary Figure 110: ^{1}H NMR spectrum of compound **5b** in $CDCl_3$

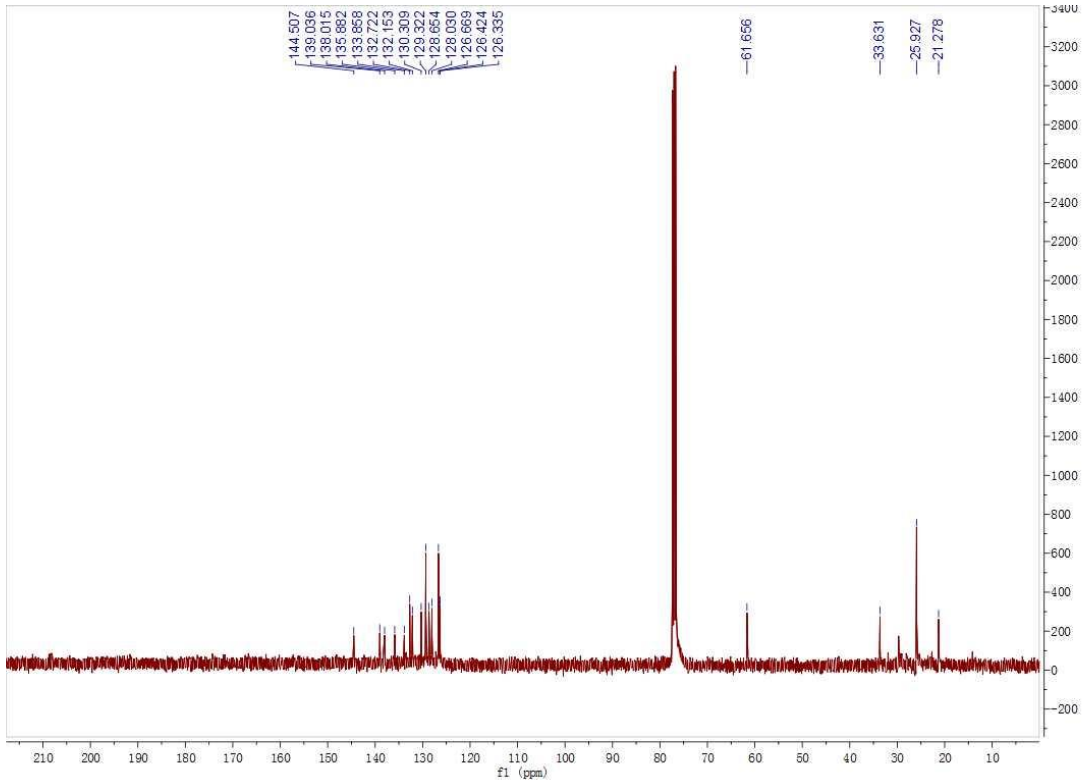

Supplementary Figure 111: ^{13}C NMR spectrum of compound **5b** in $CDCl_3$

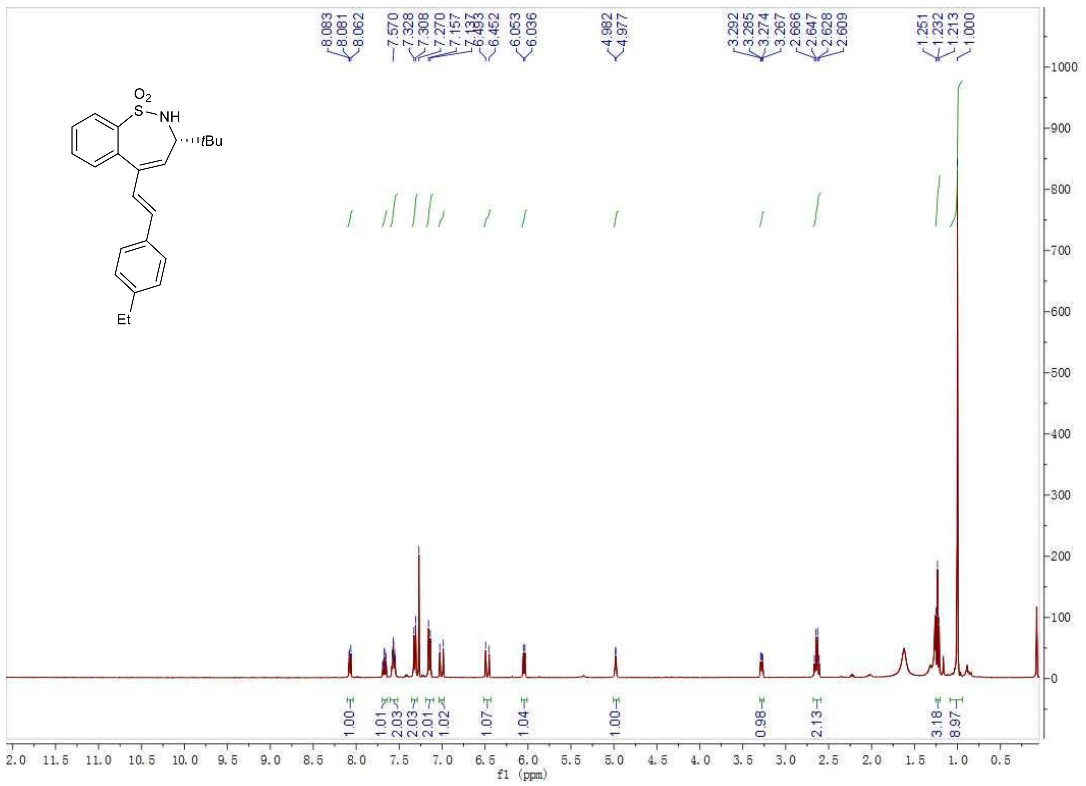

Supplementary Figure 112: ^{1}H NMR spectrum of compound **5c** in $CDCl_3$

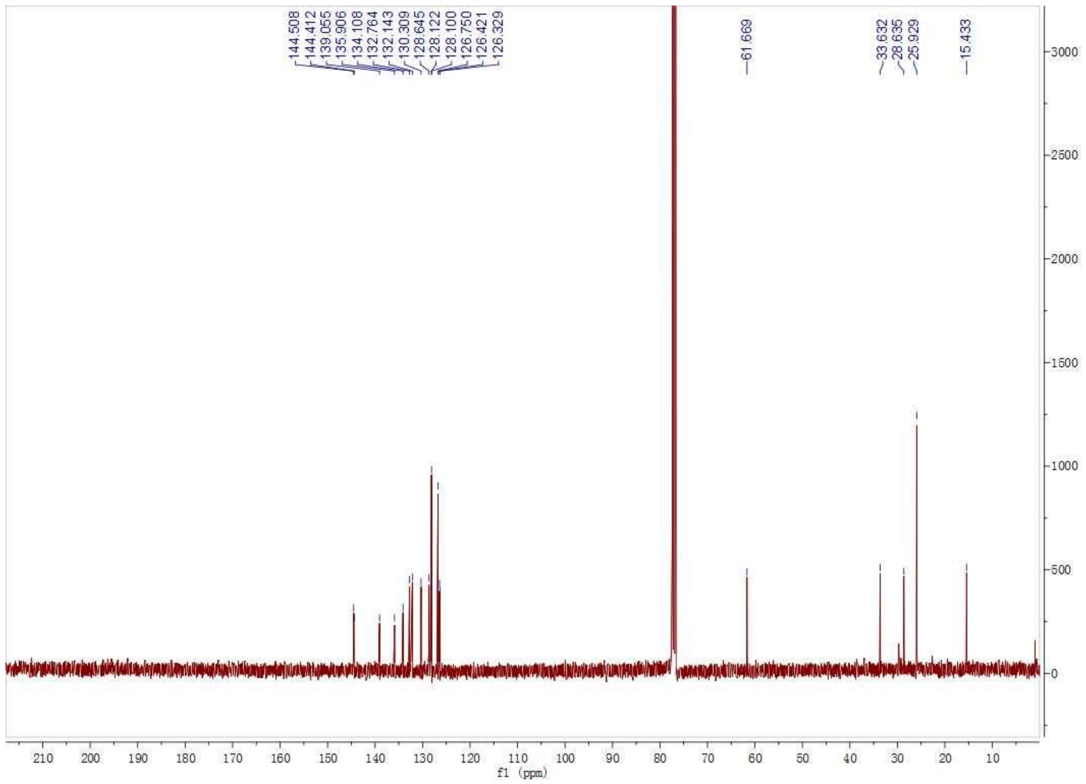

Supplementary Figure 113: ^{13}C NMR spectrum of compound **5c** in $CDCl_3$

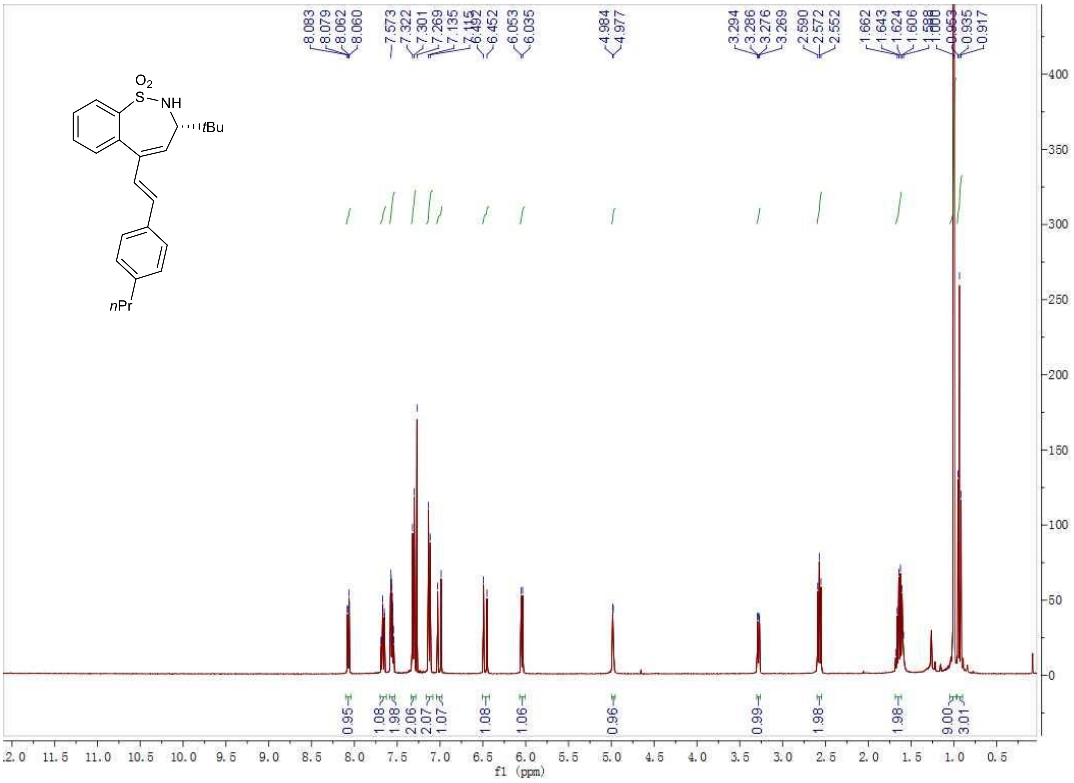

Supplementary Figure 114: ^{1}H NMR spectrum of compound **5d** in $CDCl_3$

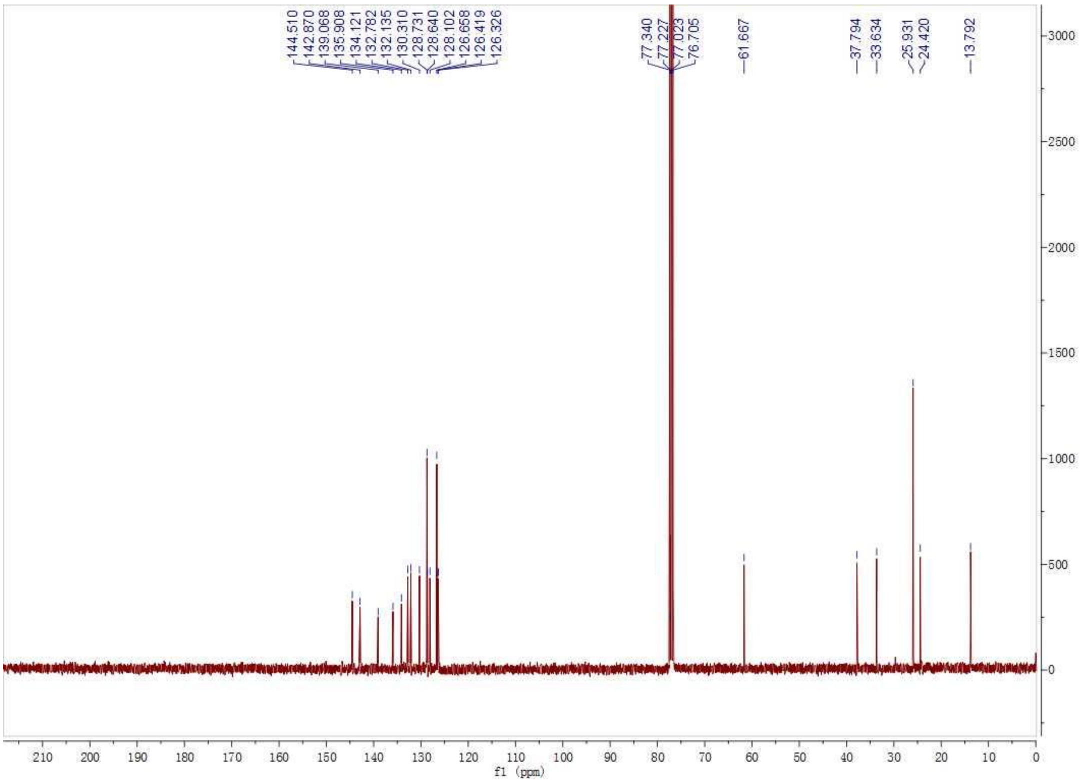

Supplementary Figure 115: ^{13}C NMR spectrum of compound **5d** in $CDCl_3$

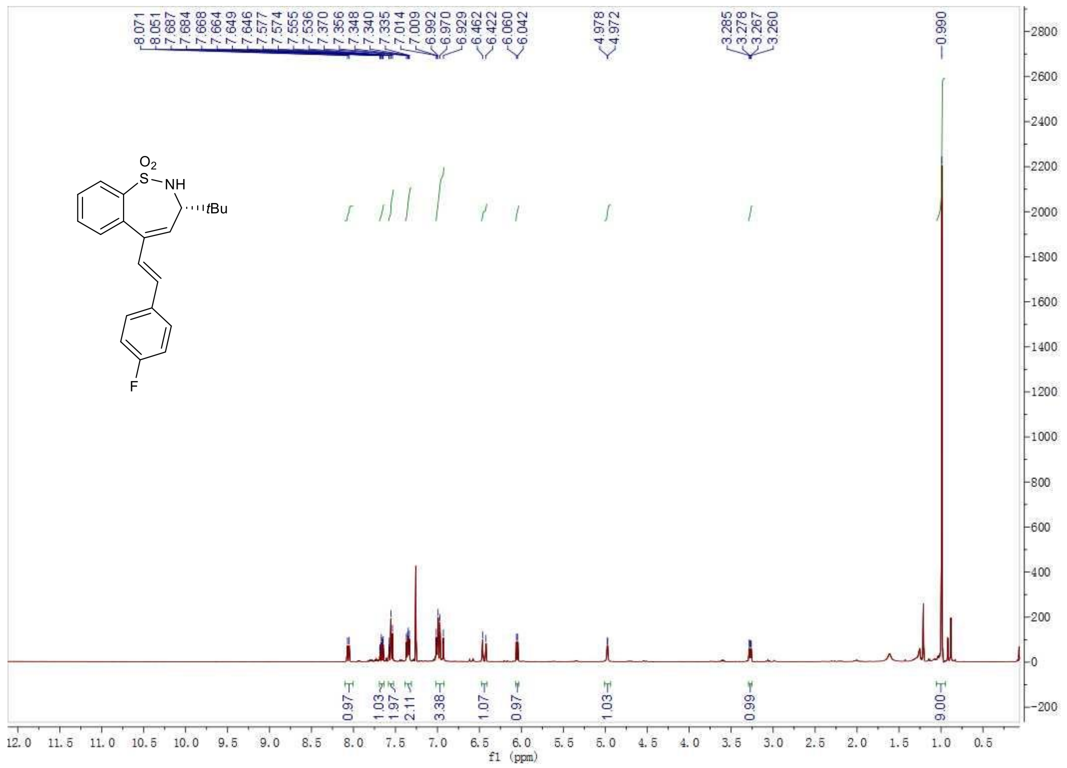

Supplementary Figure 116: ^{1}H NMR spectrum of compound **5e** in $CDCl_3$

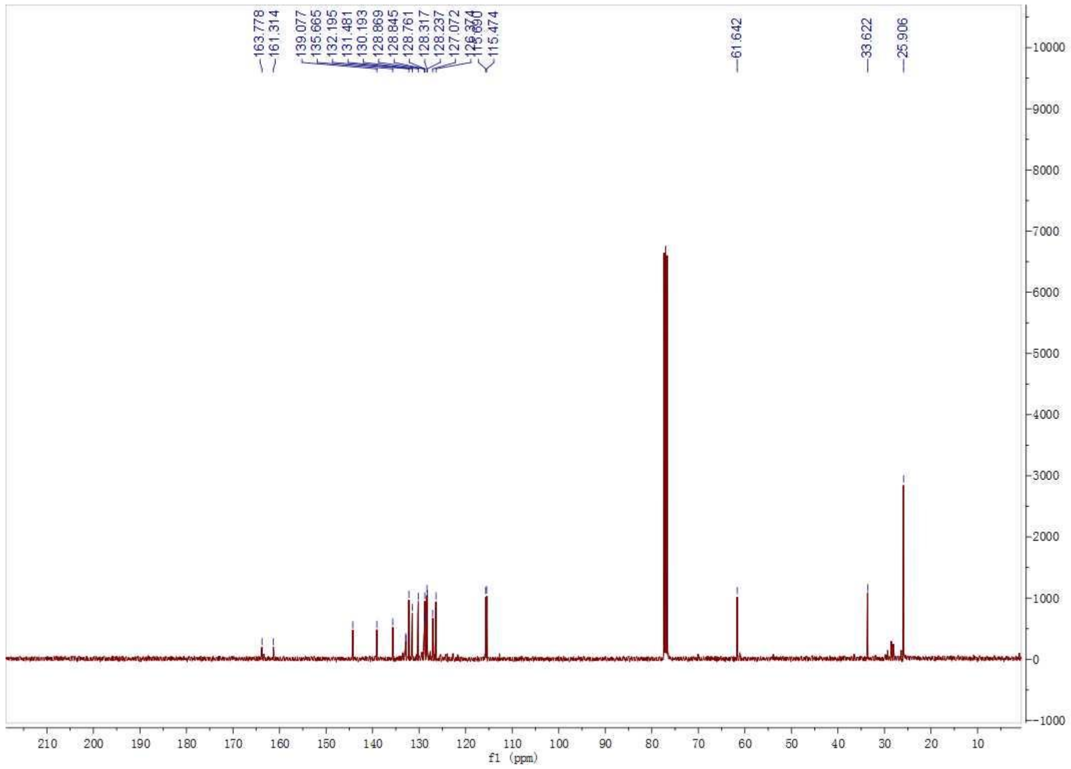

Supplementary Figure 117: ^{13}C NMR spectrum of compound **5e** in $CDCl_3$

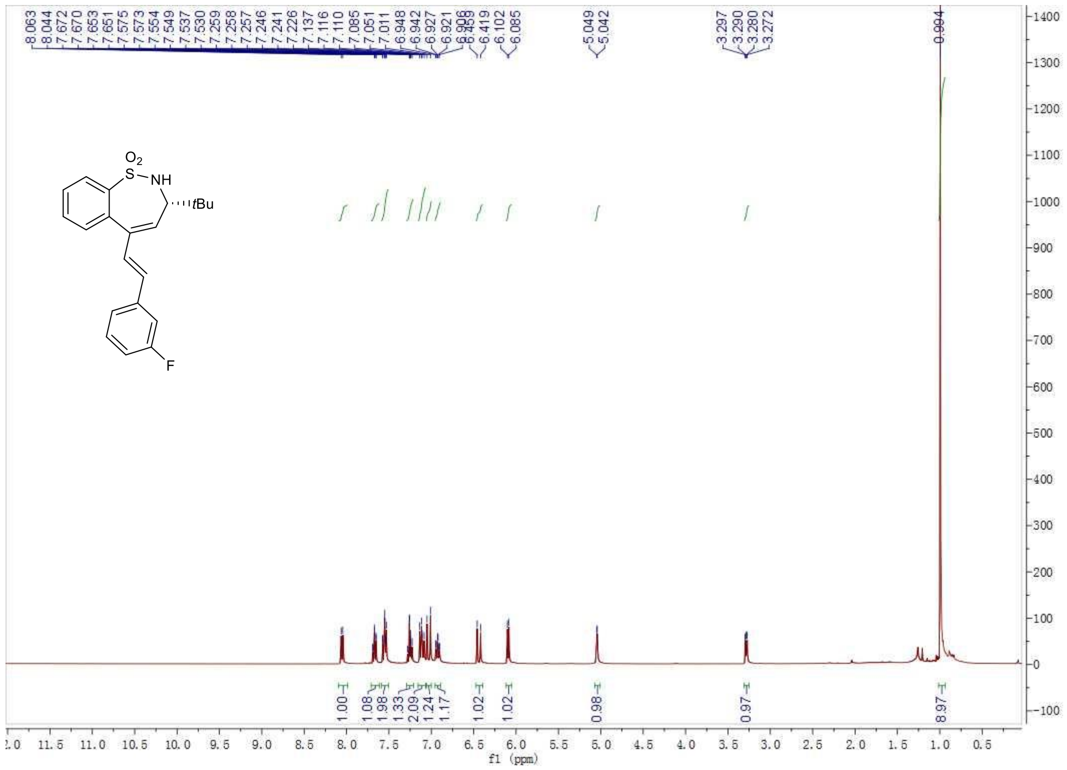

Supplementary Figure 118: ^{1}H NMR spectrum of compound **5f** in $CDCl_3$

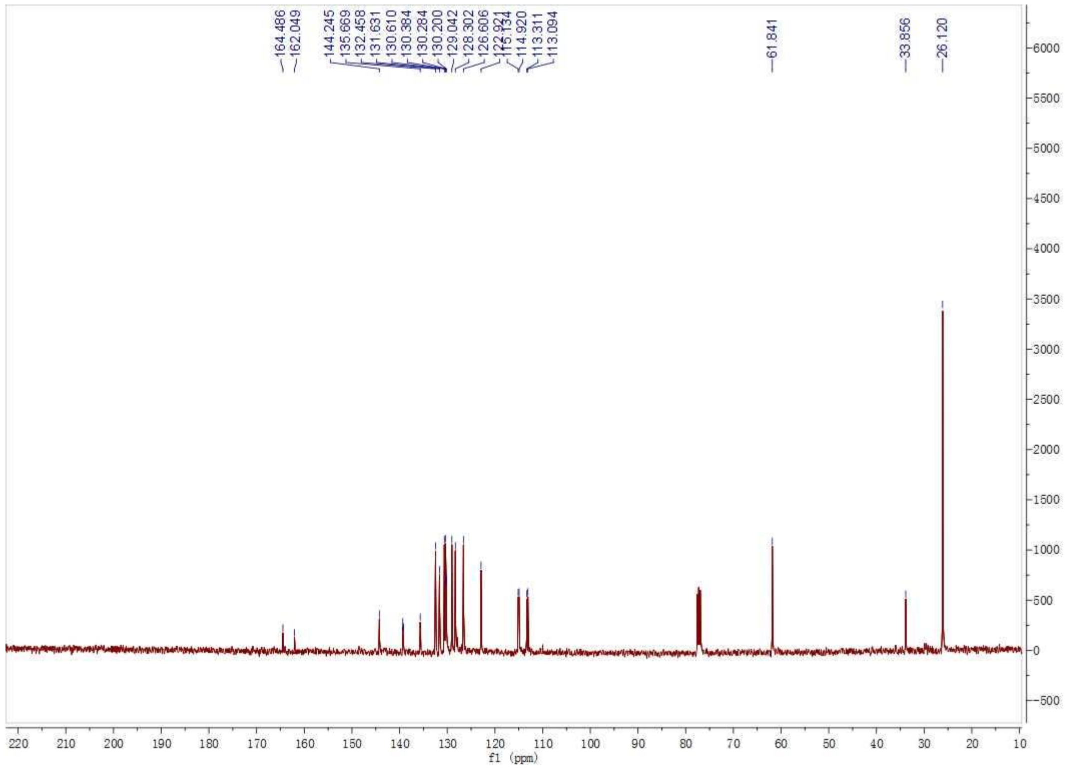

Supplementary Figure 119: ^{13}C NMR spectrum of compound **5f** in $CDCl_3$

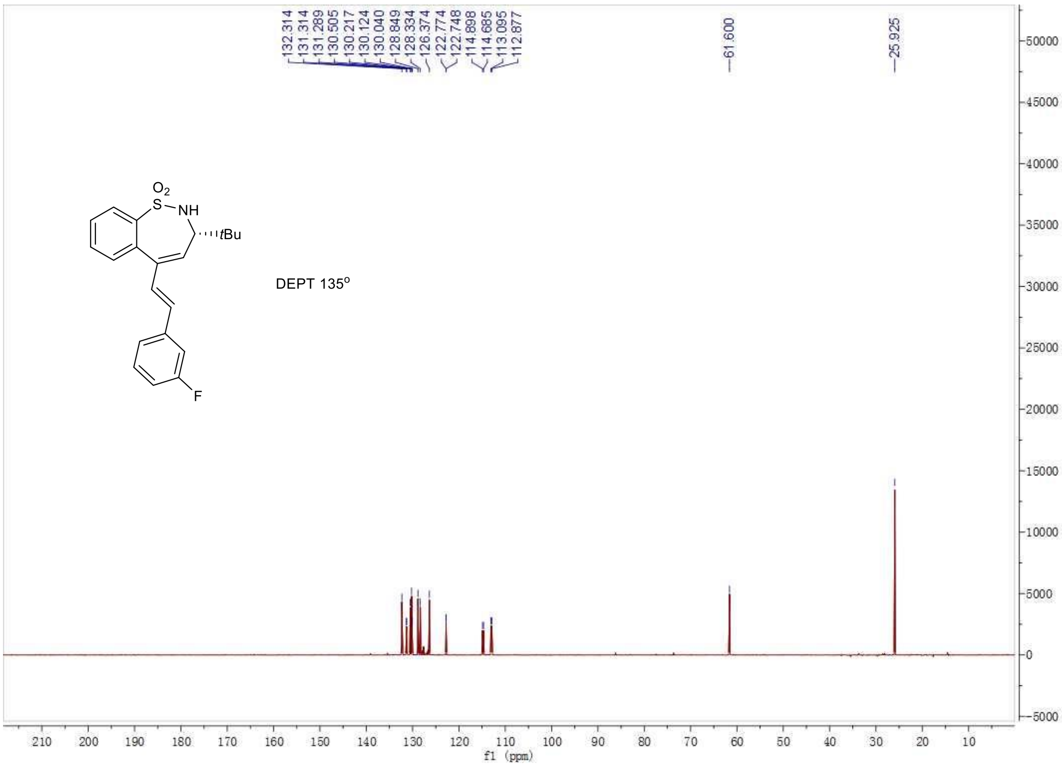

Supplementary Figure 120: ^{13}C NMR spectrum of compound **5f** in $CDCl_3$

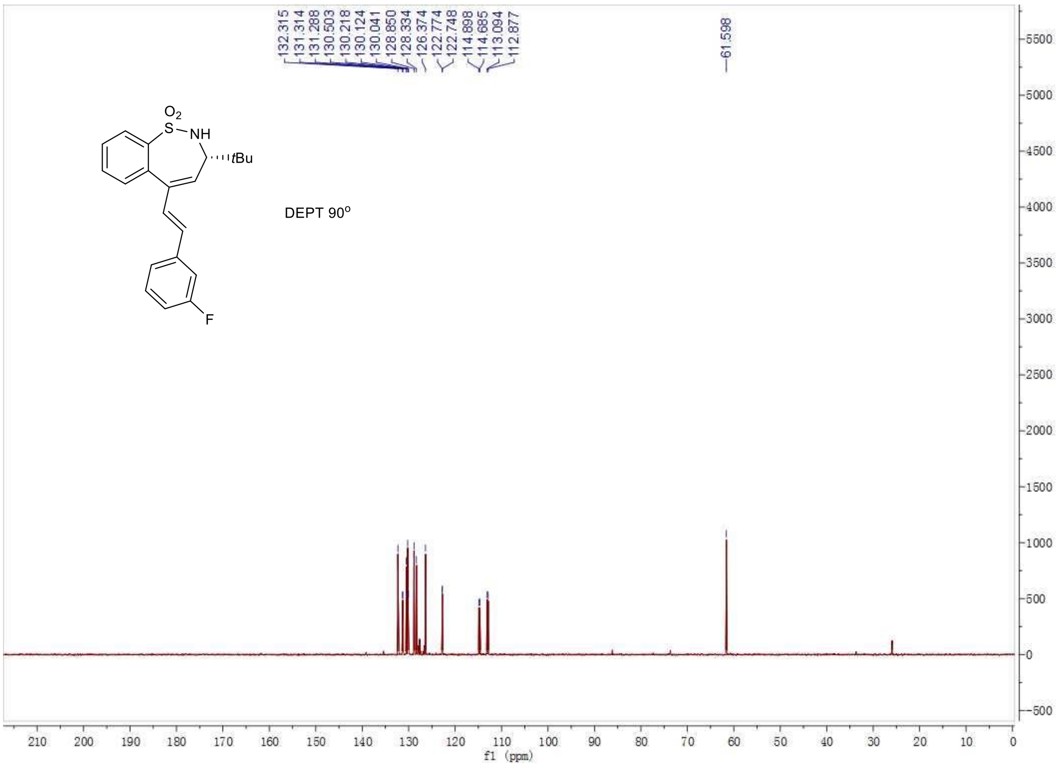

Supplementary Figure 121: ^{13}C NMR spectrum of compound **5f** in $CDCl_3$

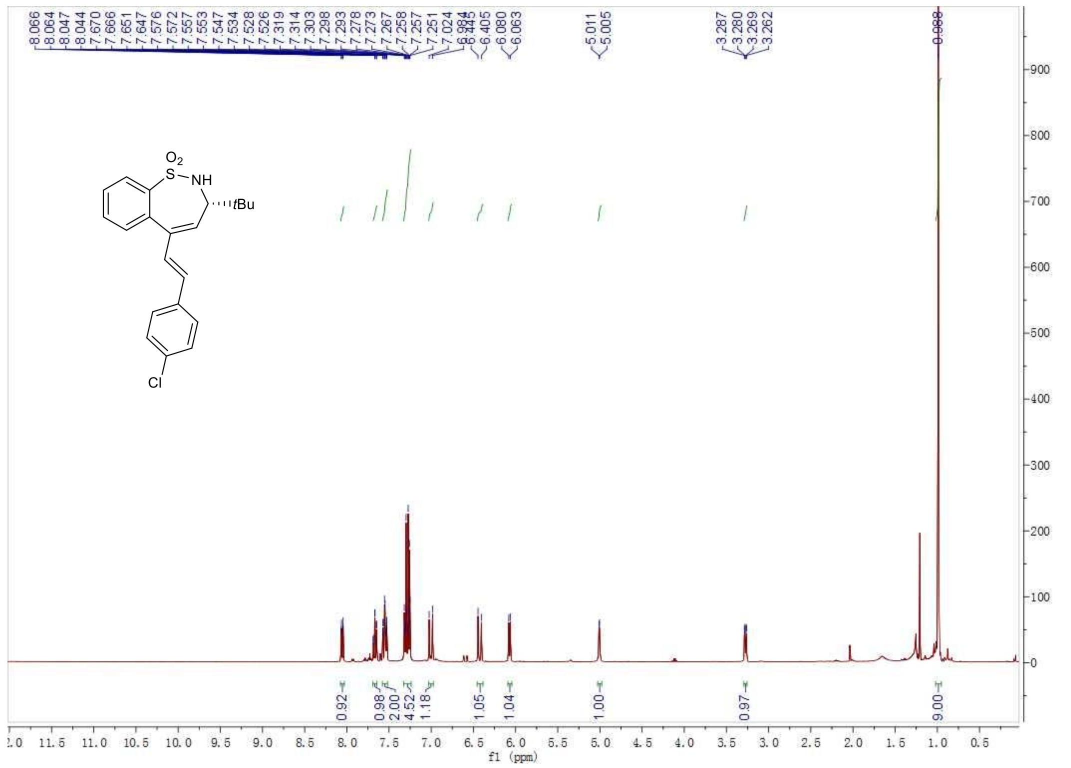

Supplementary Figure 122: ^{1}H NMR spectrum of compound **5g** in $CDCl_3$

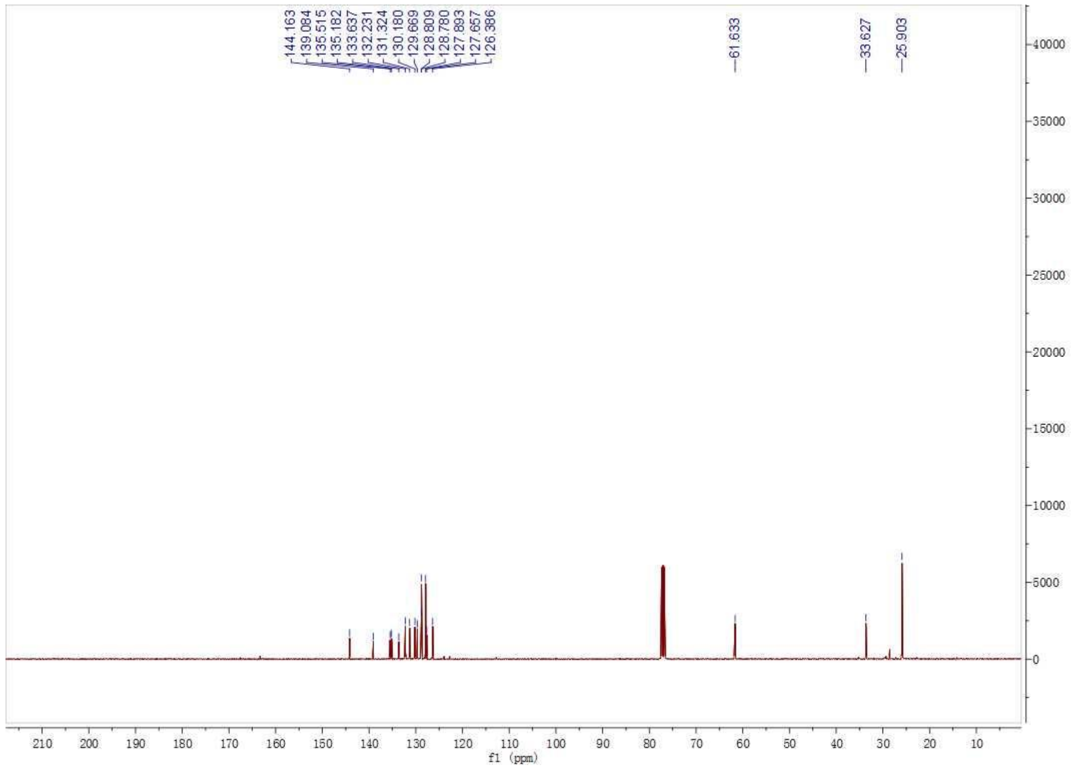

Supplementary Figure 123: ^{13}C NMR spectrum of compound **5g** in $CDCl_3$

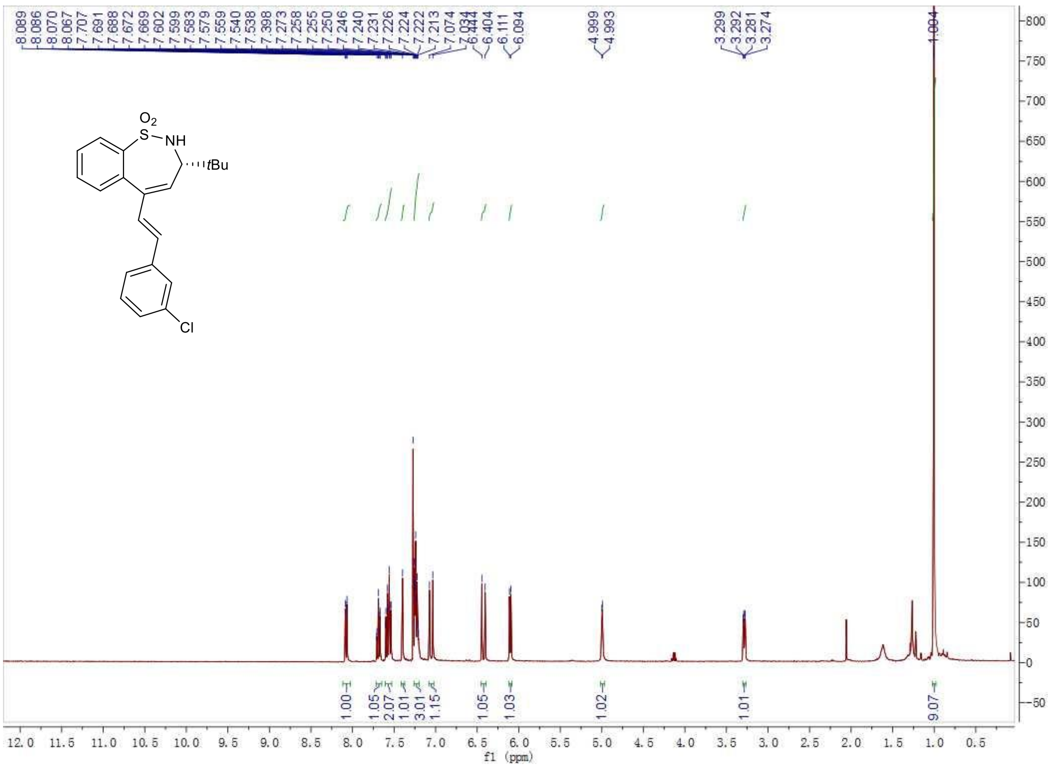

Supplementary Figure 124: ^{1}H NMR spectrum of compound **5h** in $CDCl_3$

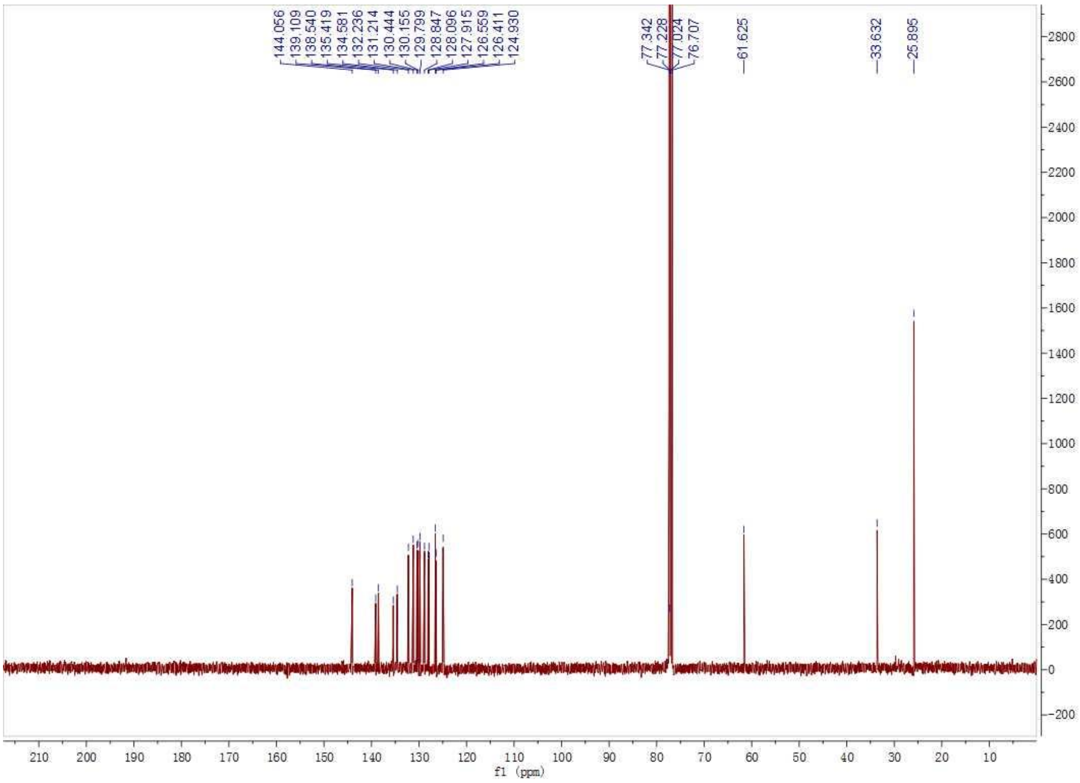

Supplementary Figure 125: ^{13}C NMR spectrum of compound **5h** in $CDCl_3$

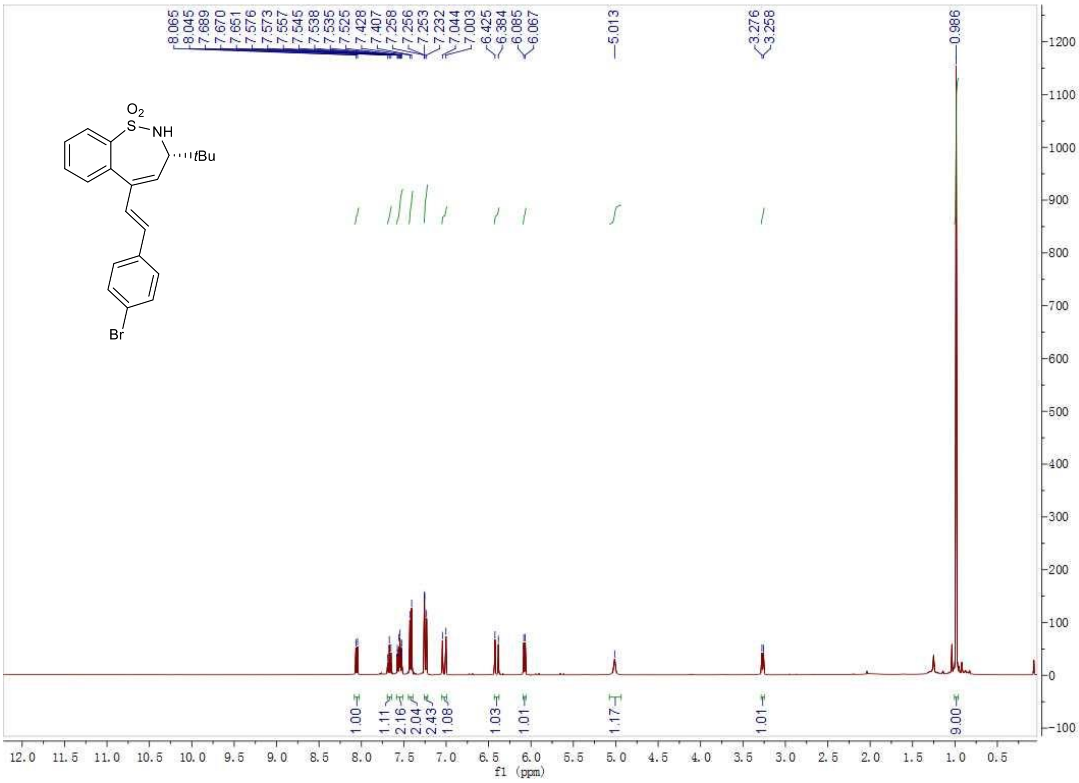

Supplementary Figure 126: ^{1}H NMR spectrum of compound **5i** in $CDCl_3$

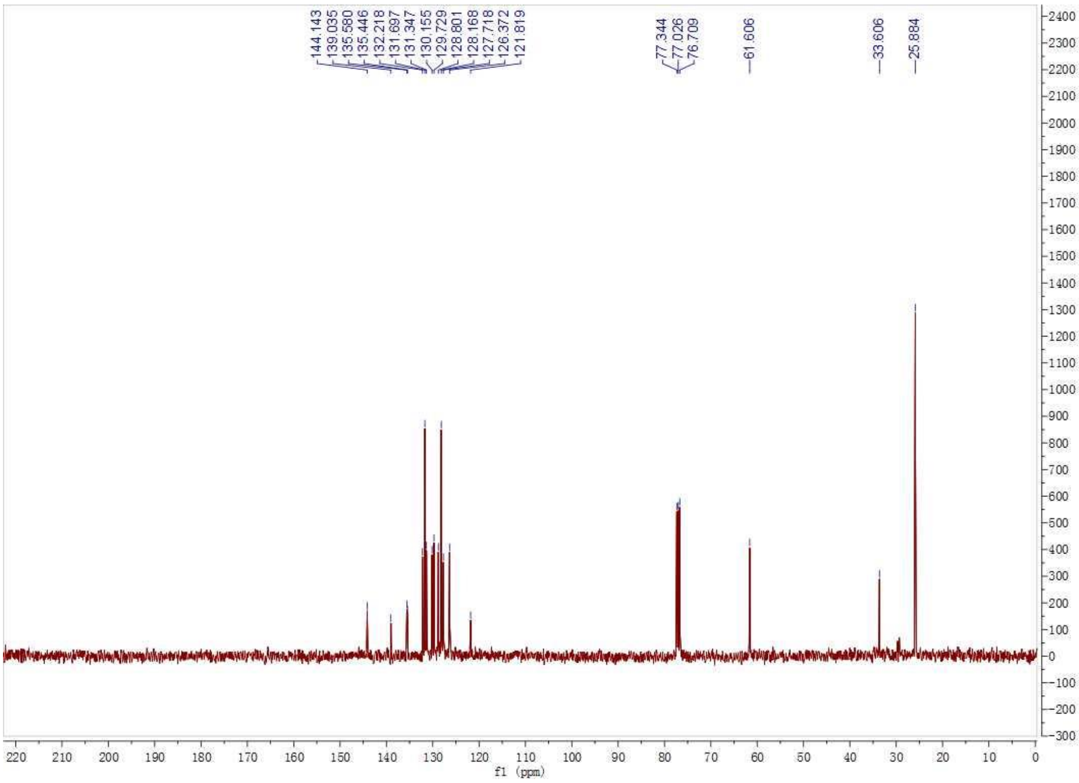

Supplementary Figure 127: ^{13}C NMR spectrum of compound **5i** in $CDCl_3$

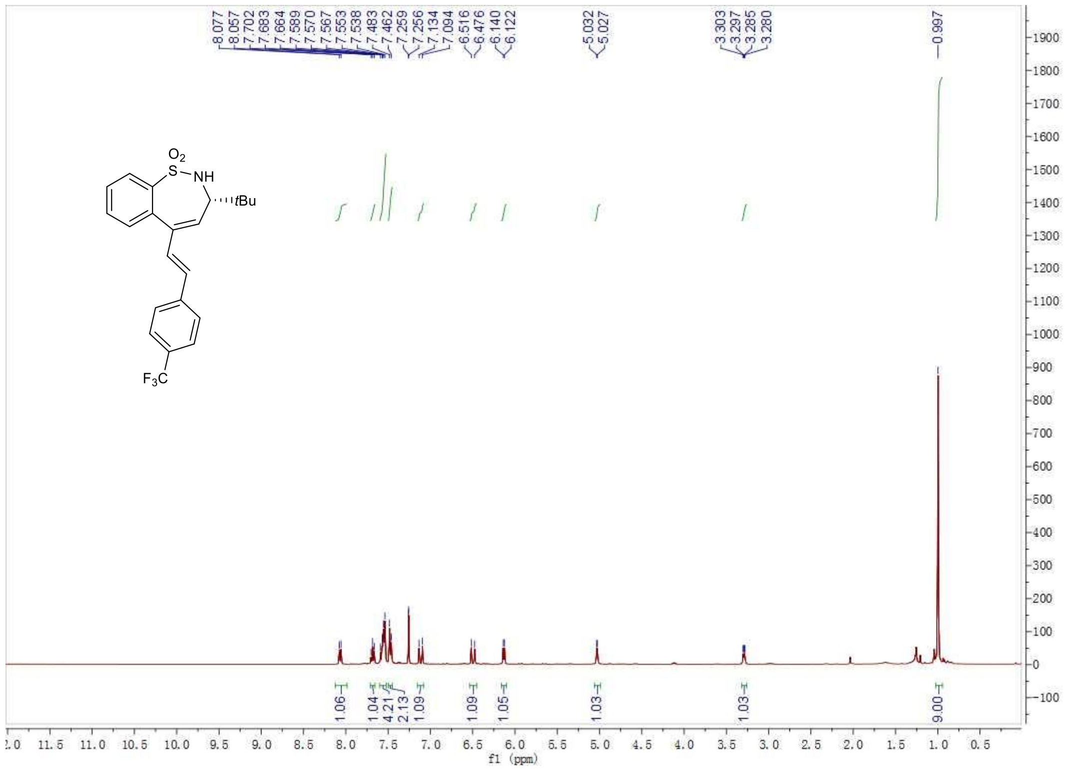

Supplementary Figure 128: ^{1}H NMR spectrum of compound **5j** in $CDCl_3$

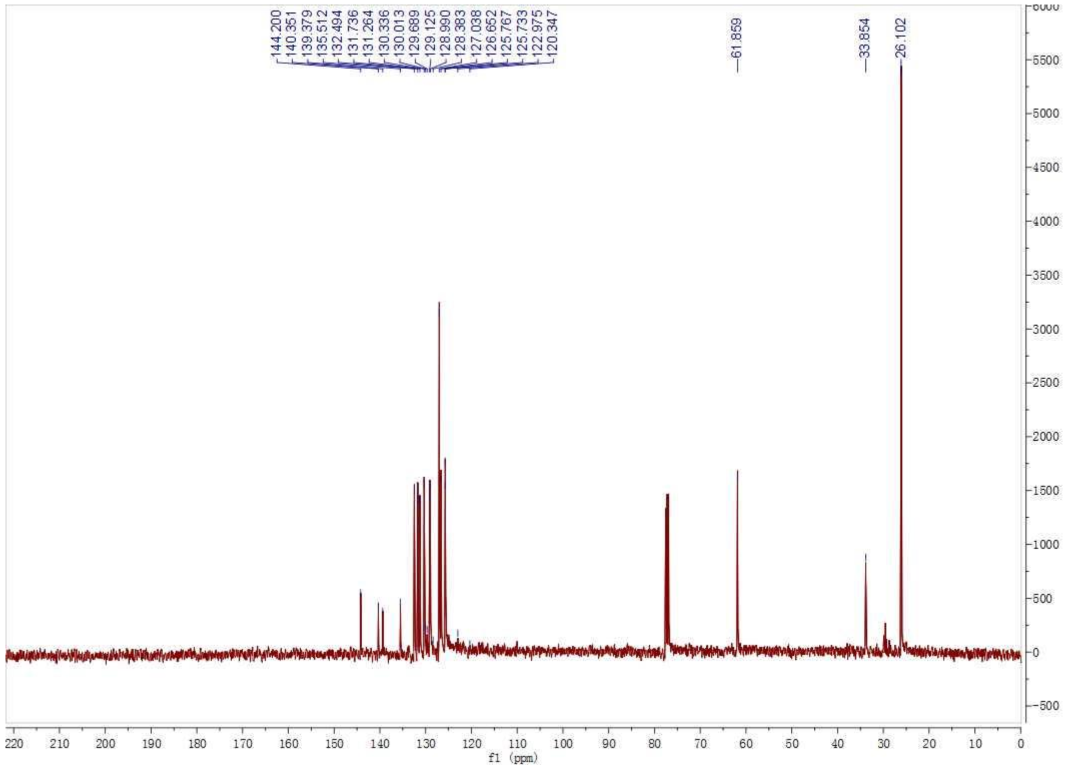

Supplementary Figure 129: ^{13}C NMR spectrum of compound **5j** in $CDCl_3$

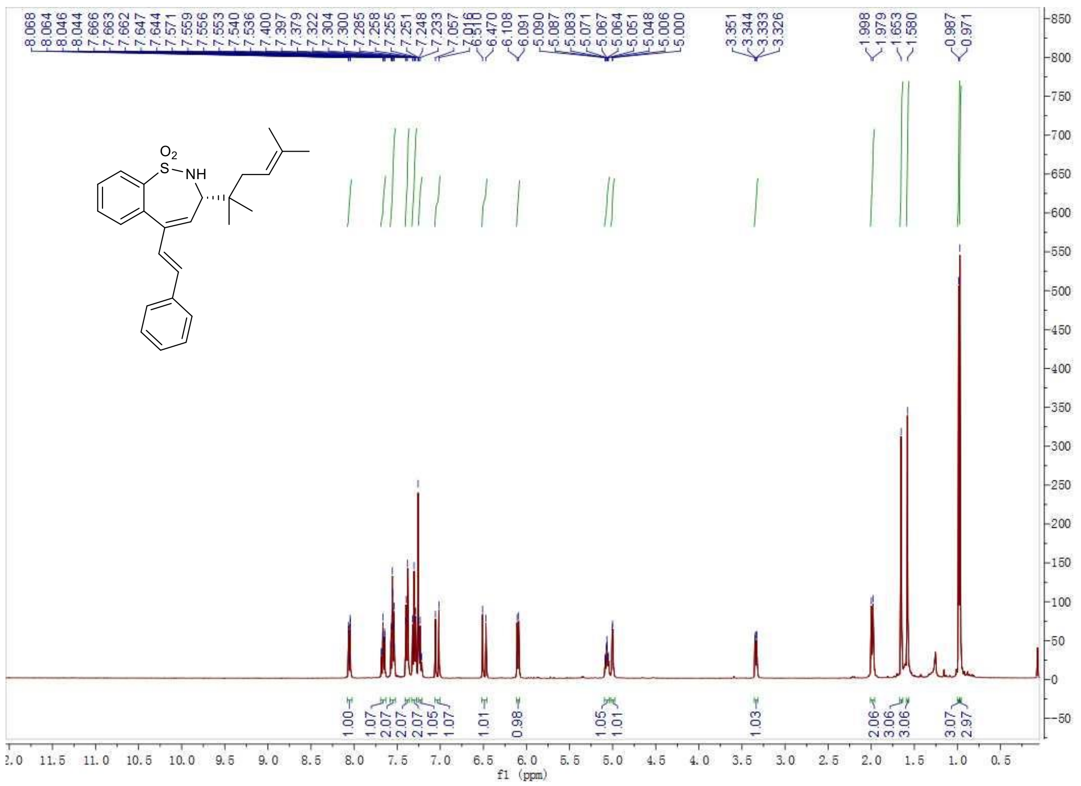

Supplementary Figure 130: ^{1}H NMR spectrum of compound **5k** in $CDCl_3$

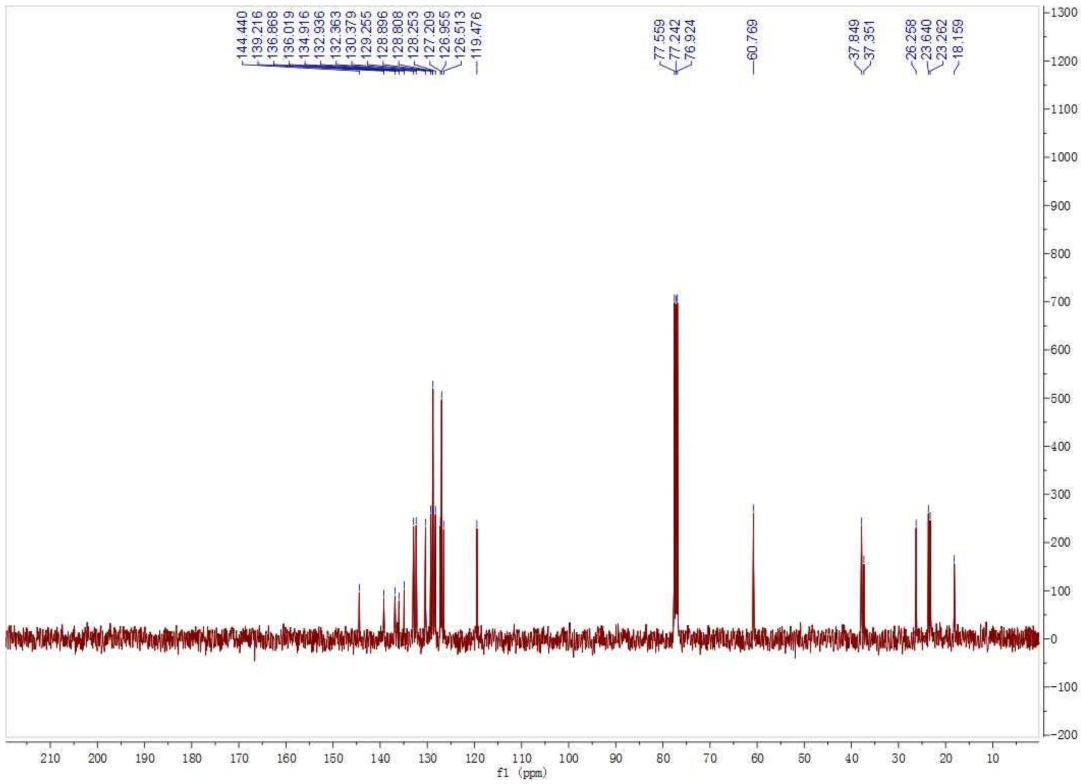

Supplementary Figure 131: ^{13}C NMR spectrum of compound **5k** in $CDCl_3$

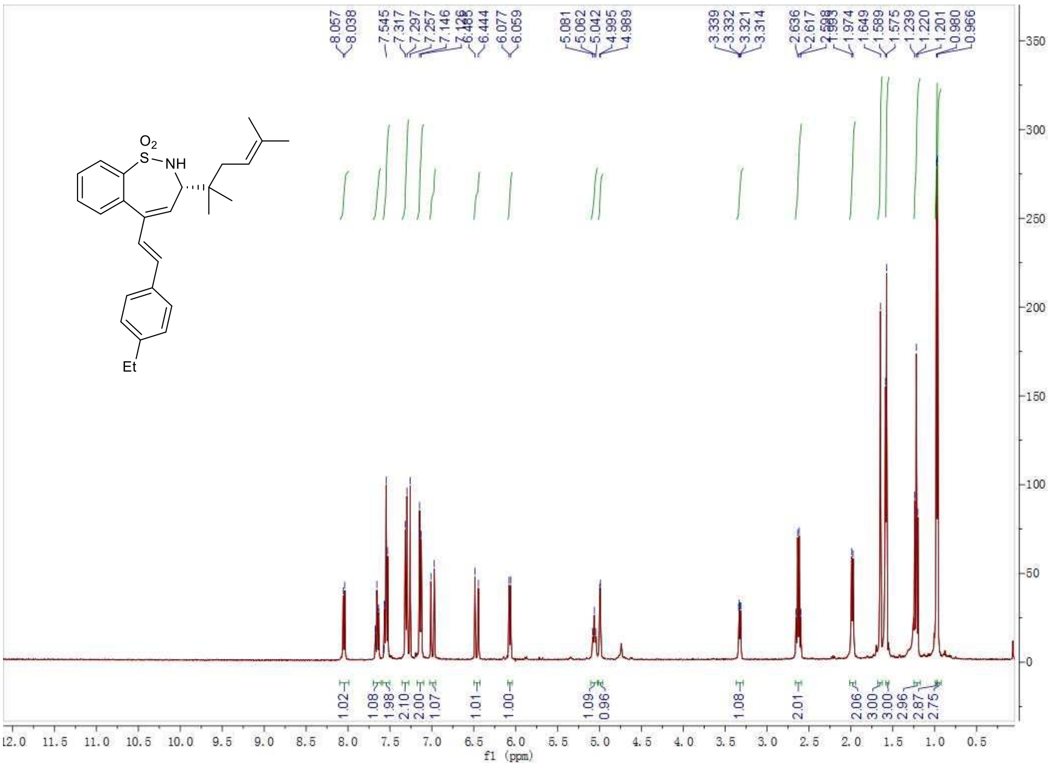

Supplementary Figure 132: ^{1}H NMR spectrum of compound **5l** in $CDCl_3$

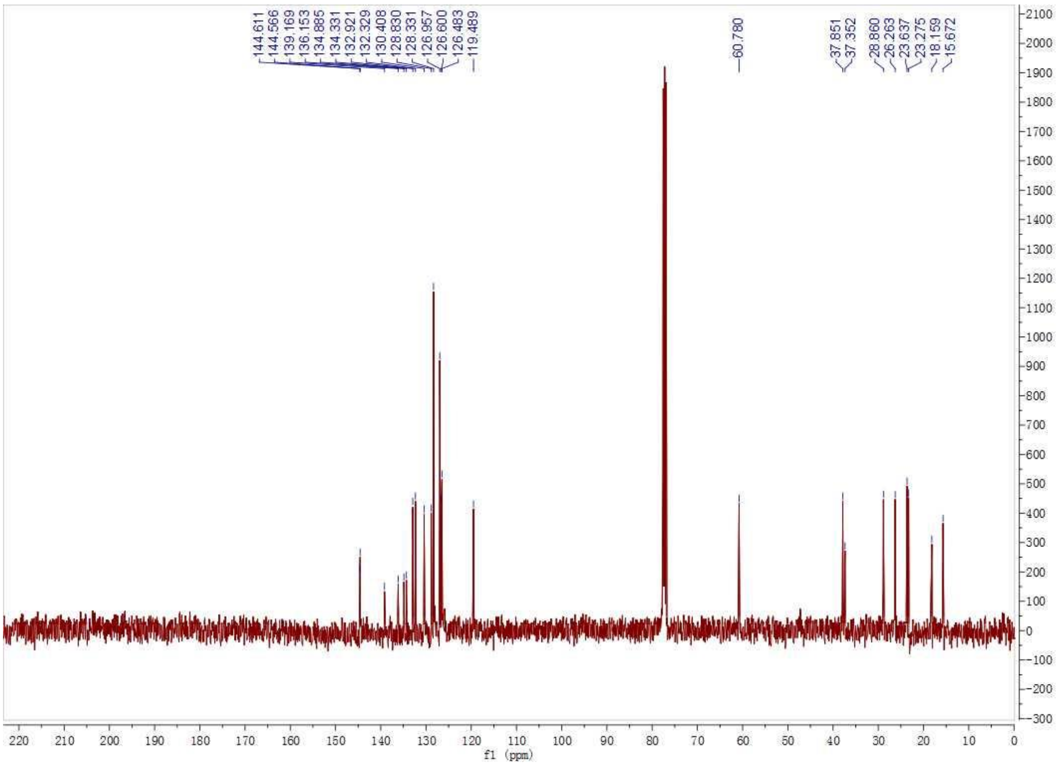

Supplementary Figure 133: ^{13}C NMR spectrum of compound **5l** in $CDCl_3$

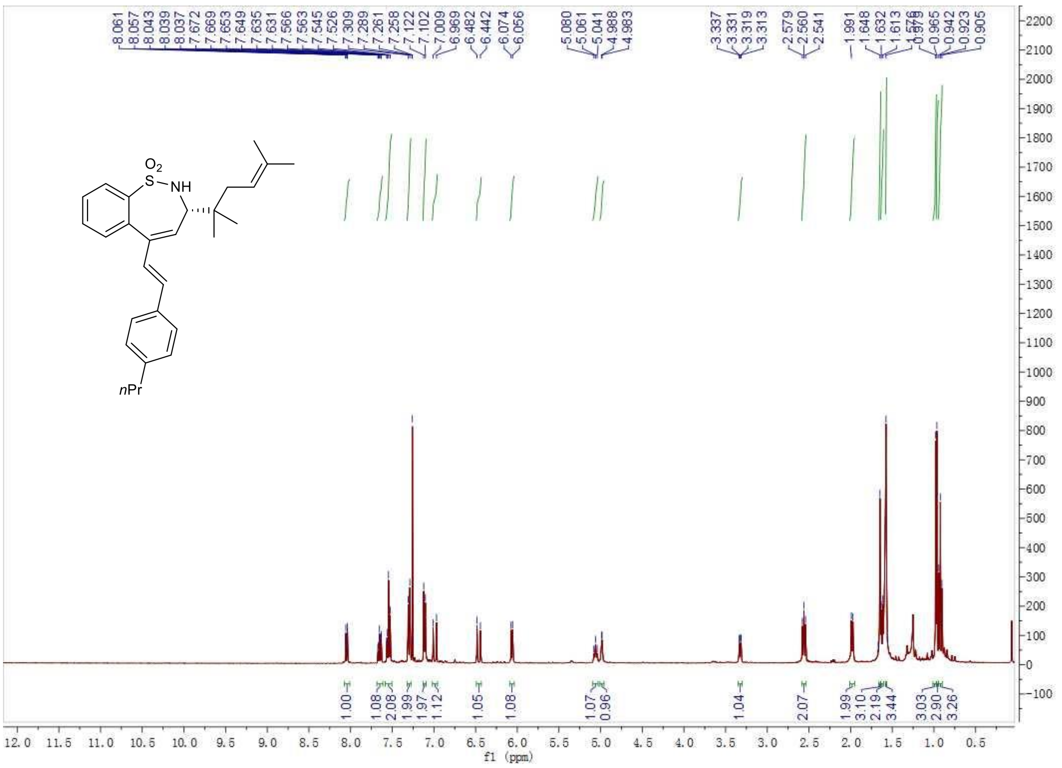

Supplementary Figure 134: ^{1}H NMR spectrum of compound **5m** in $CDCl_3$

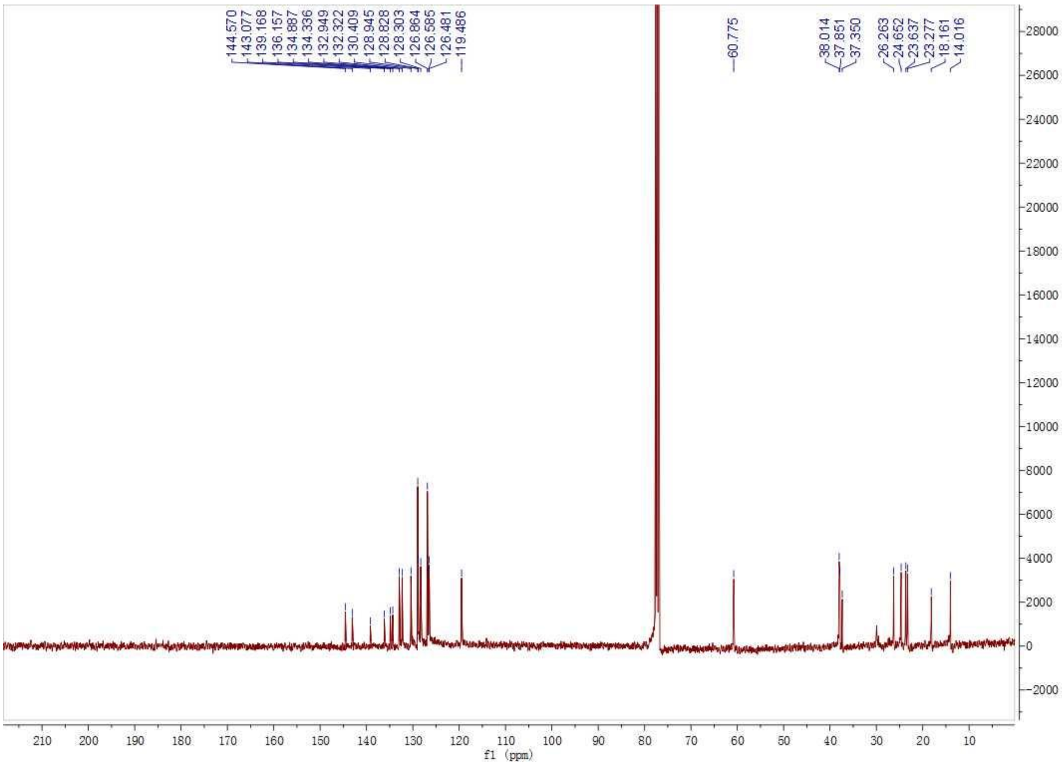

Supplementary Figure 135: ^{13}C NMR spectrum of compound **5m** in $CDCl_3$

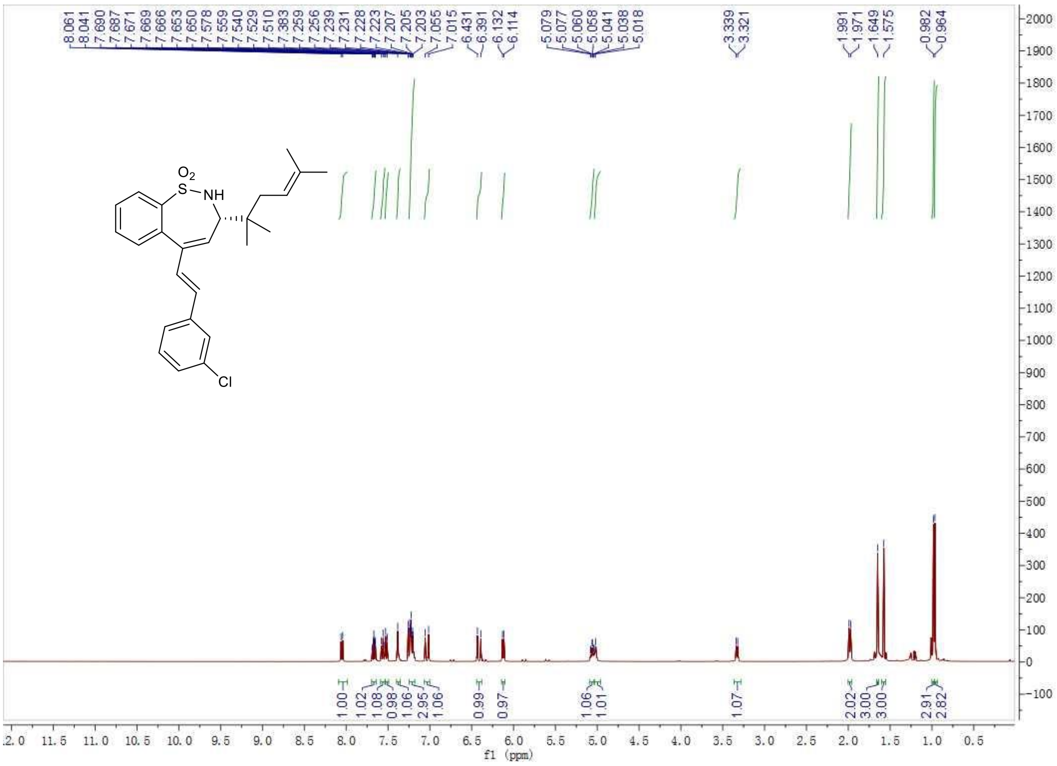

Supplementary Figure 136: ^{1}H NMR spectrum of compound **5n** in $CDCl_3$

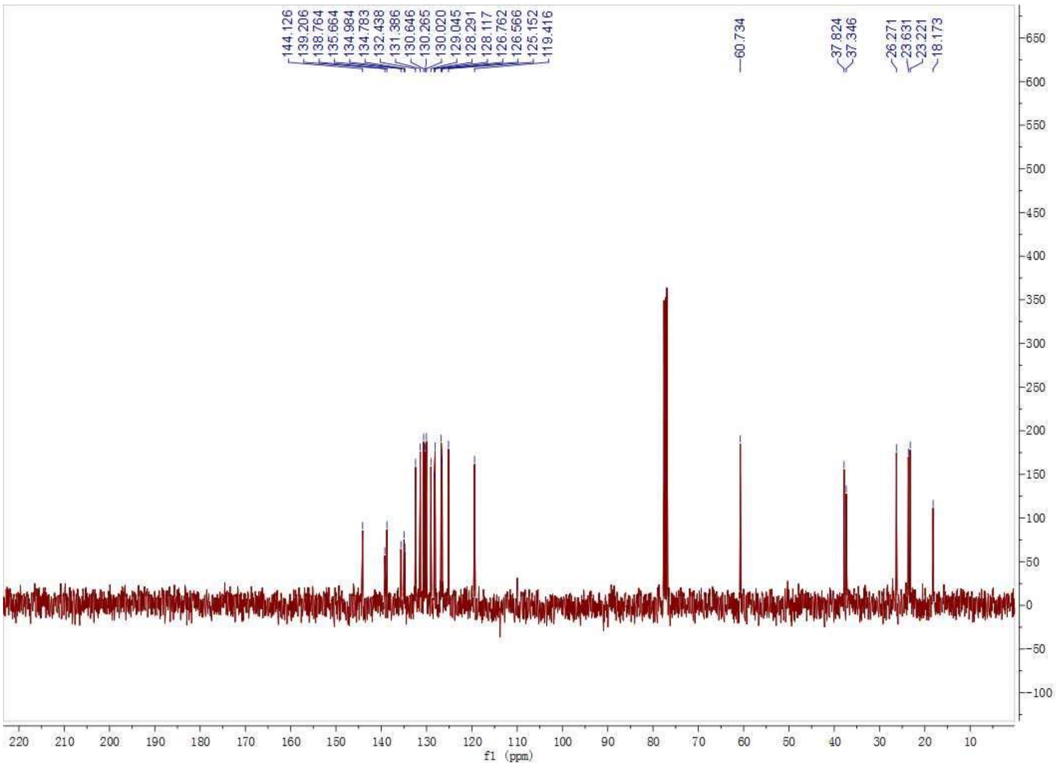

Supplementary Figure 137: ^{13}C NMR spectrum of compound **5n** in $CDCl_3$

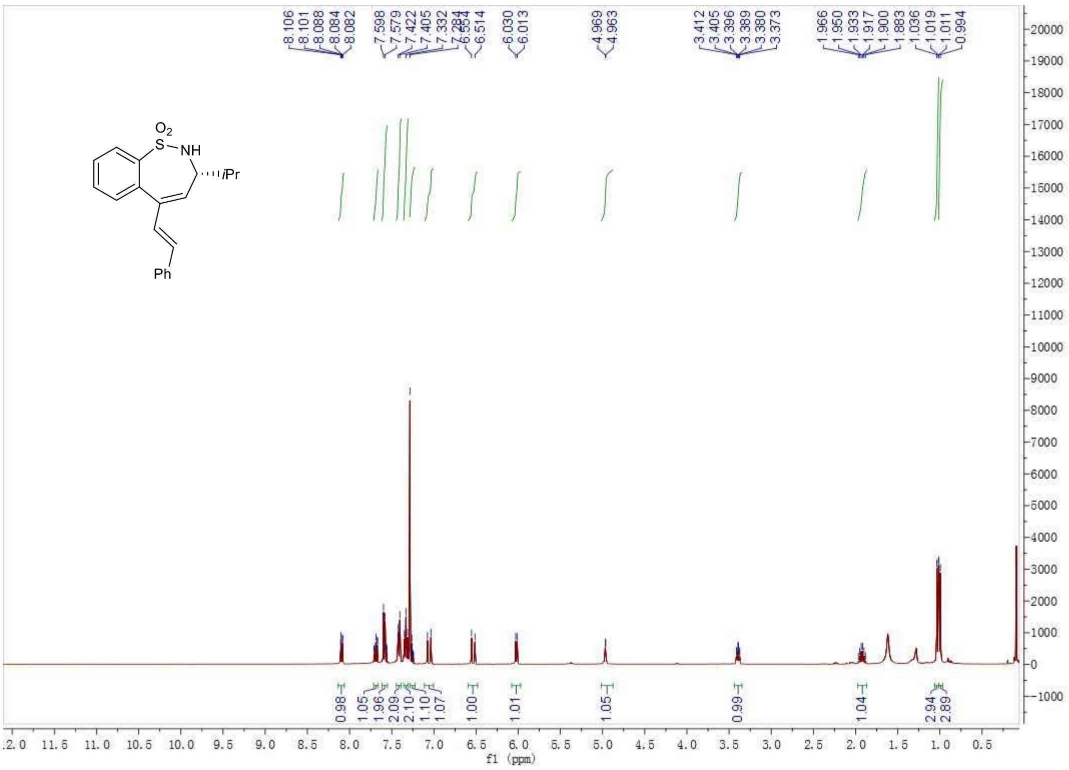

Supplementary Figure 138: ^{1}H NMR spectrum of compound **5o** in $CDCl_3$

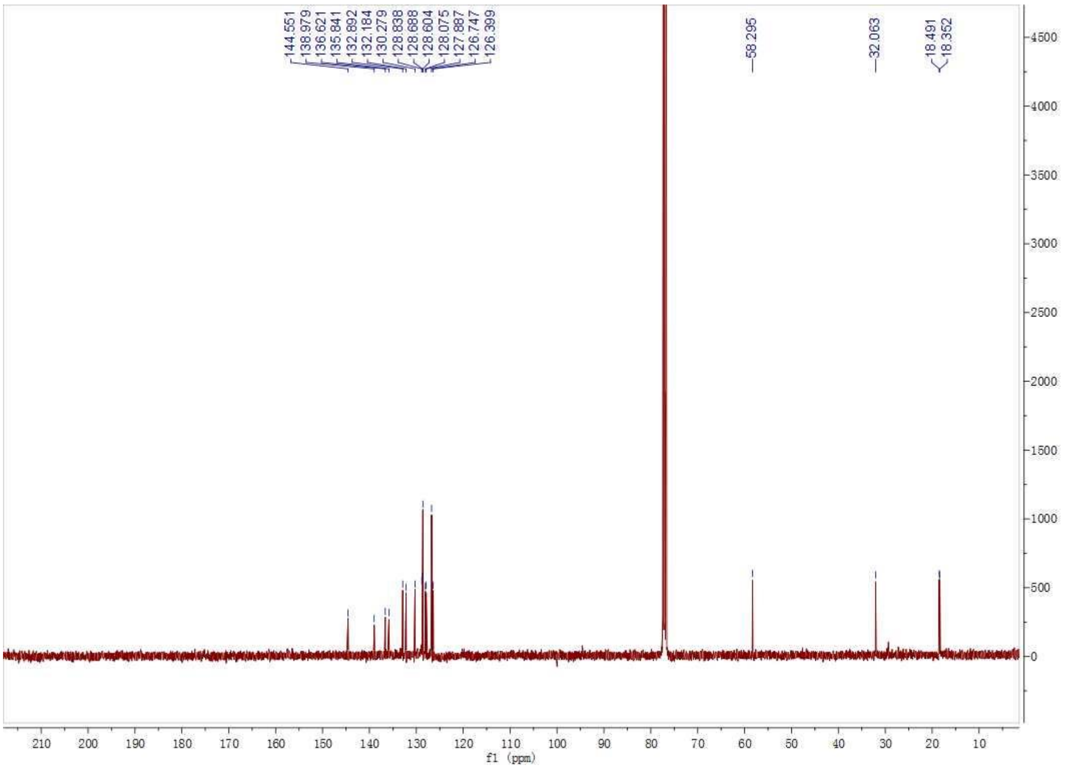

Supplementary Figure 139: ^{13}C NMR spectrum of compound **5o** in $CDCl_3$

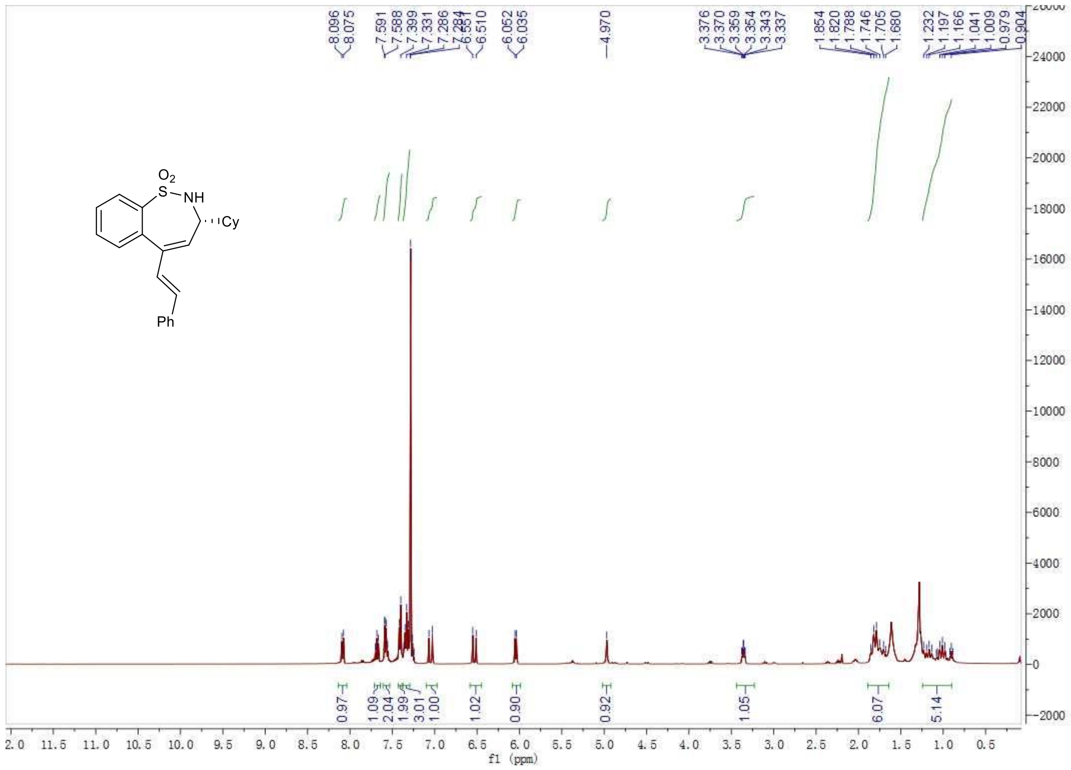

Supplementary Figure 140: ^{1}H NMR spectrum of compound **5p** in $CDCl_3$

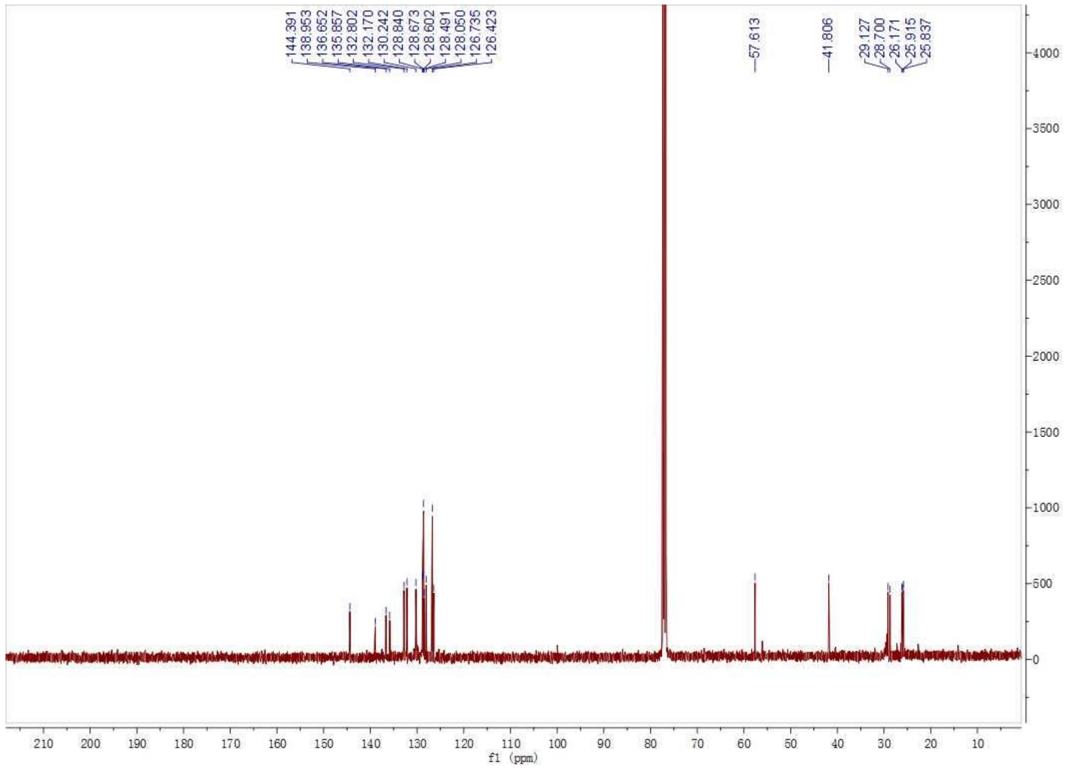

Supplementary Figure 141: ^{13}C NMR spectrum of compound **5p** in $CDCl_3$

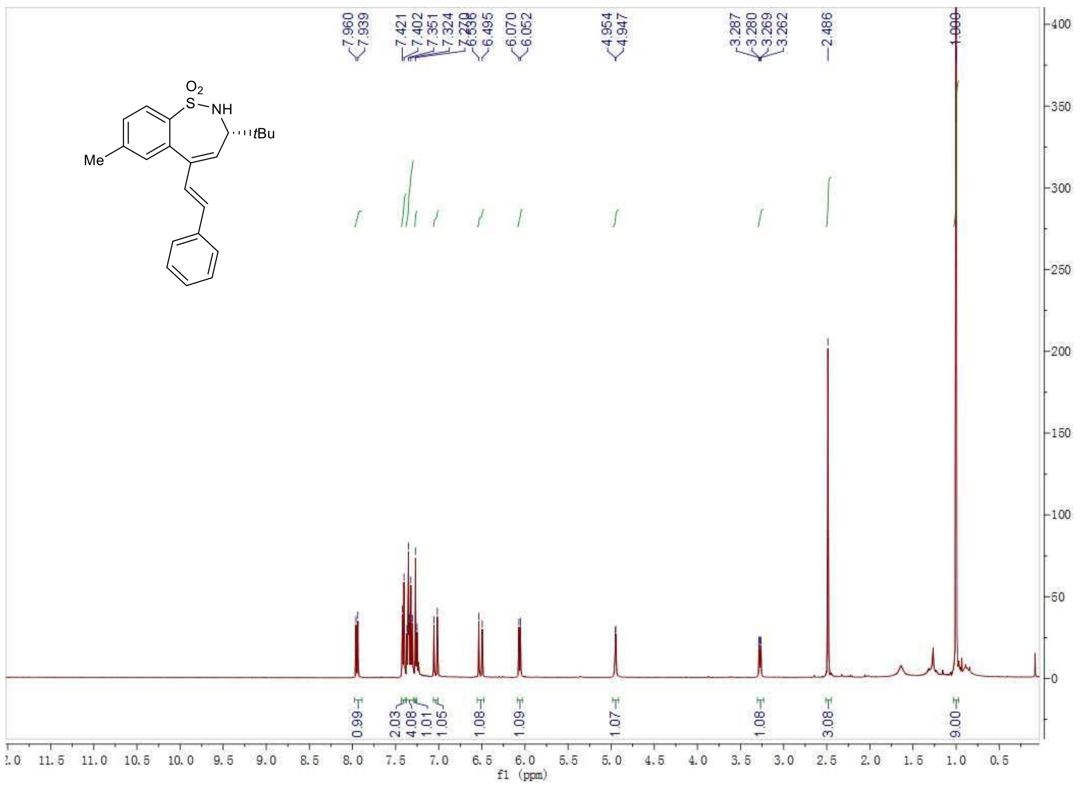

Supplementary Figure 142: ^{1}H NMR spectrum of compound **5q** in $CDCl_3$

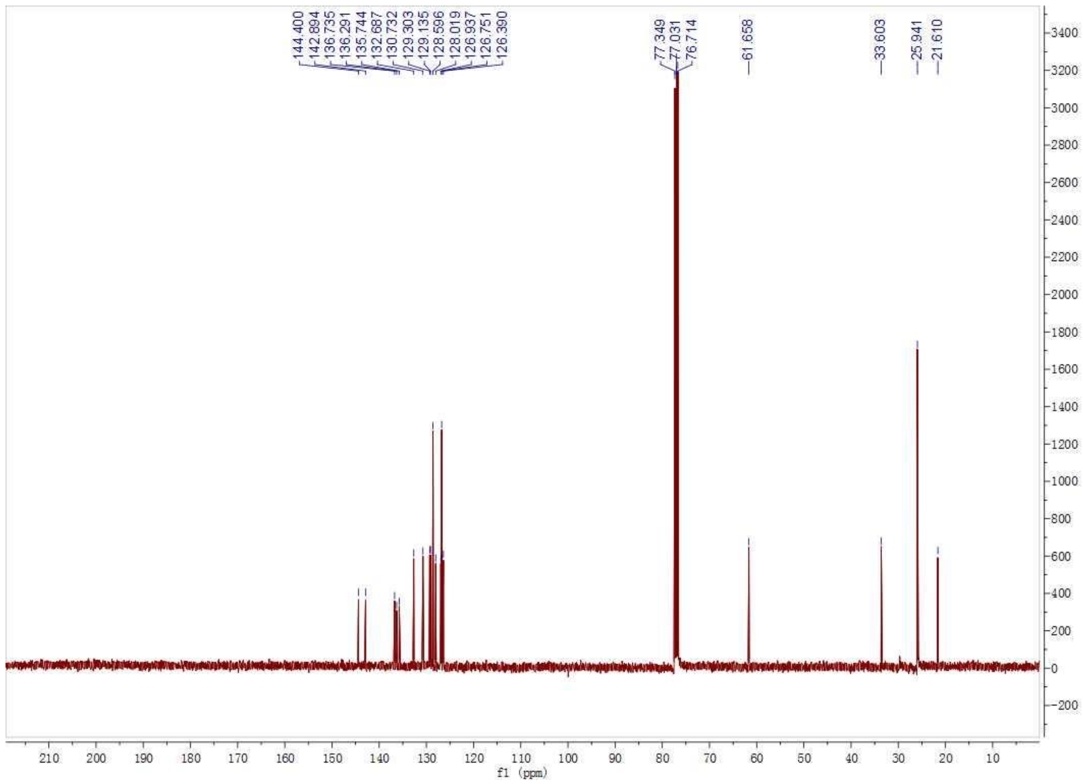

Supplementary Figure 143: ^{13}C NMR spectrum of compound **5q** in $CDCl_3$

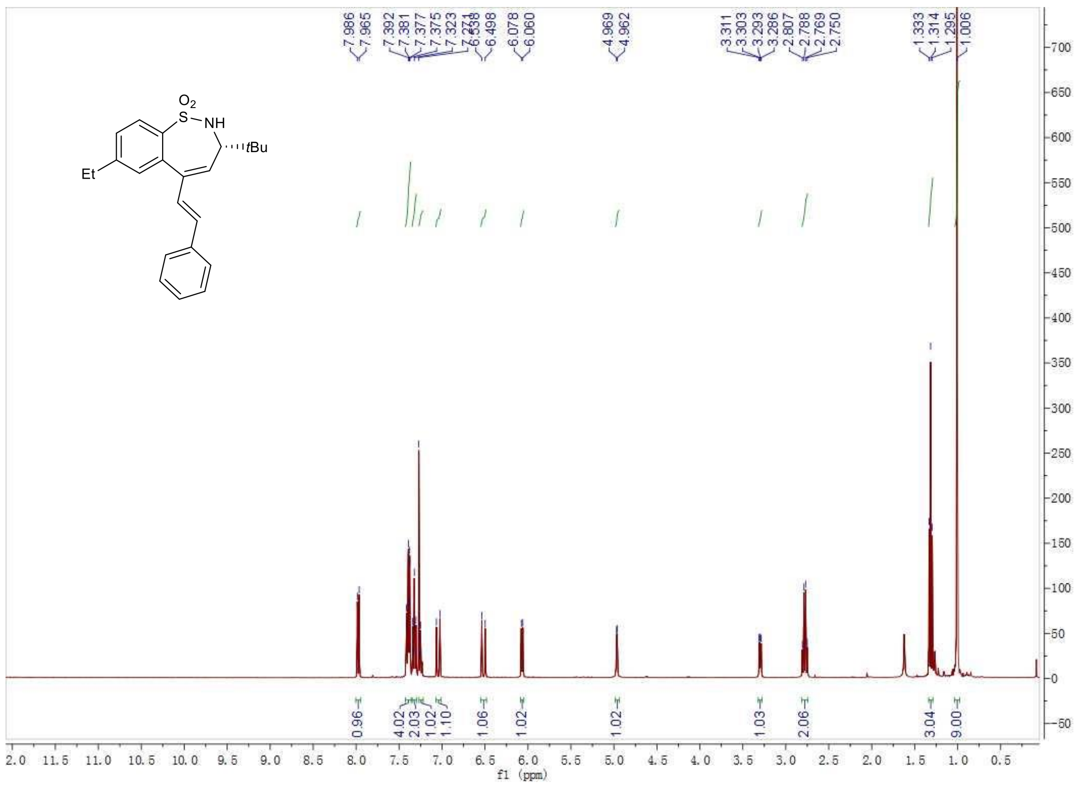

Supplementary Figure 144: ^{1}H NMR spectrum of compound **5r** in $CDCl_3$

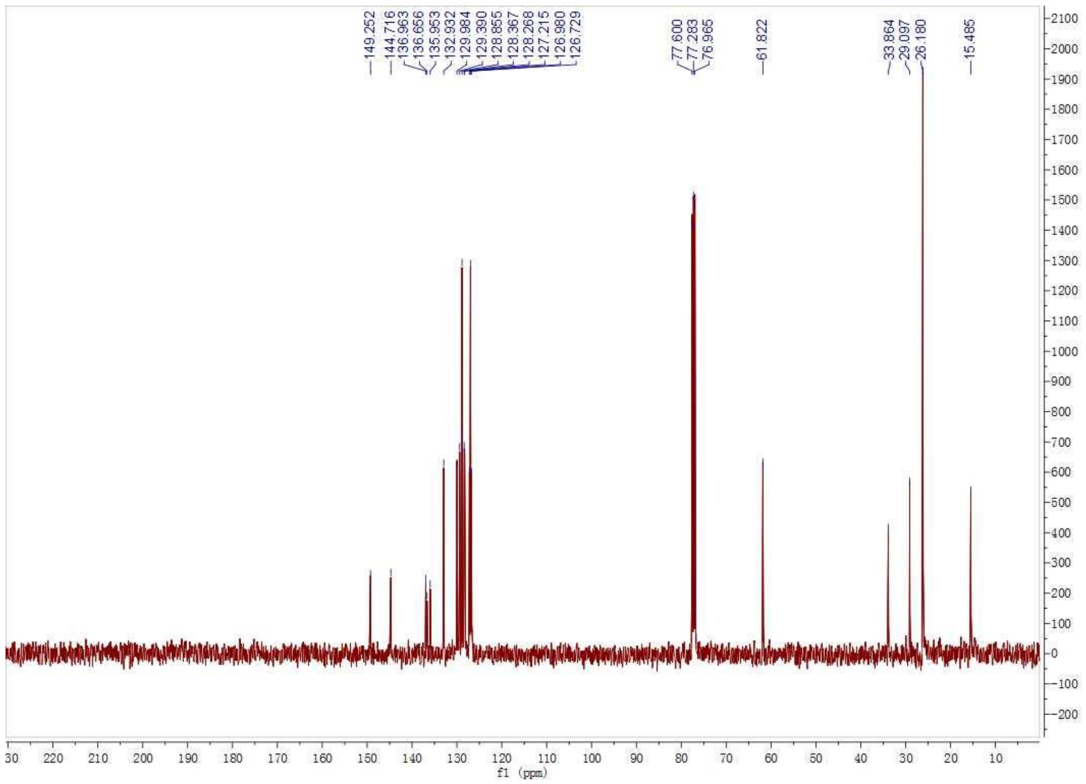

Supplementary Figure 145: ^{13}C NMR spectrum of compound **5r** in $CDCl_3$

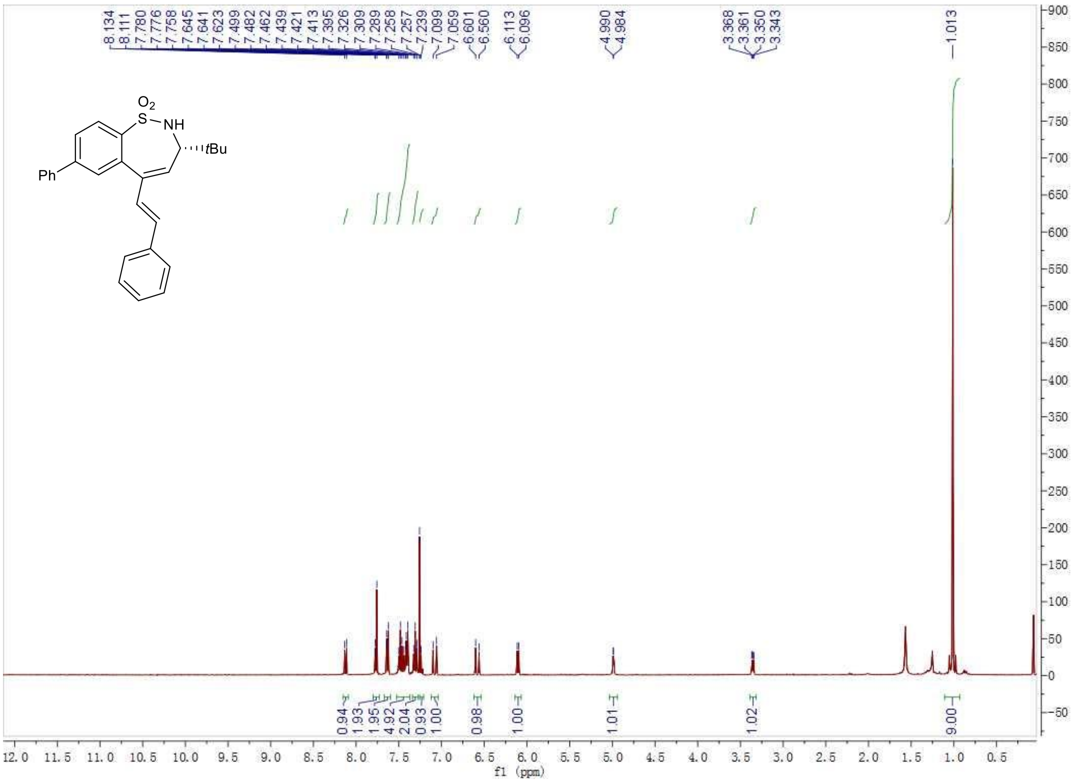

Supplementary Figure 146: ^{1}H NMR spectrum of compound **5s** in $CDCl_3$

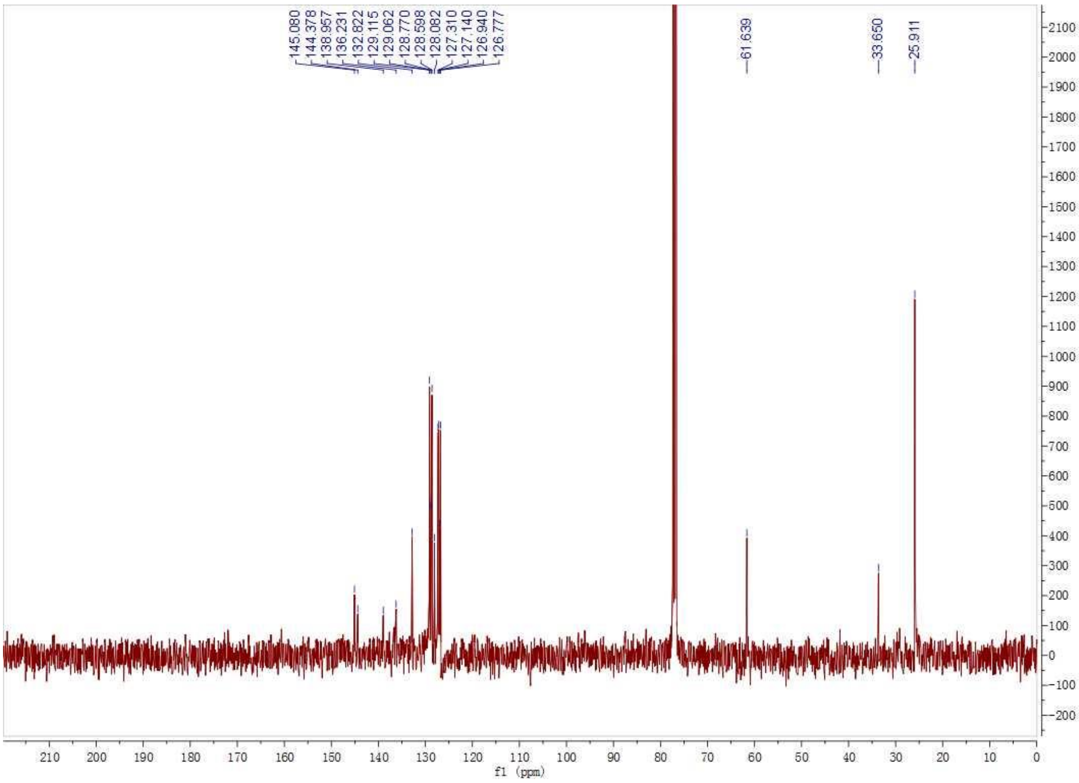

Supplementary Figure 147: ^{13}C NMR spectrum of compound **5s** in $CDCl_3$

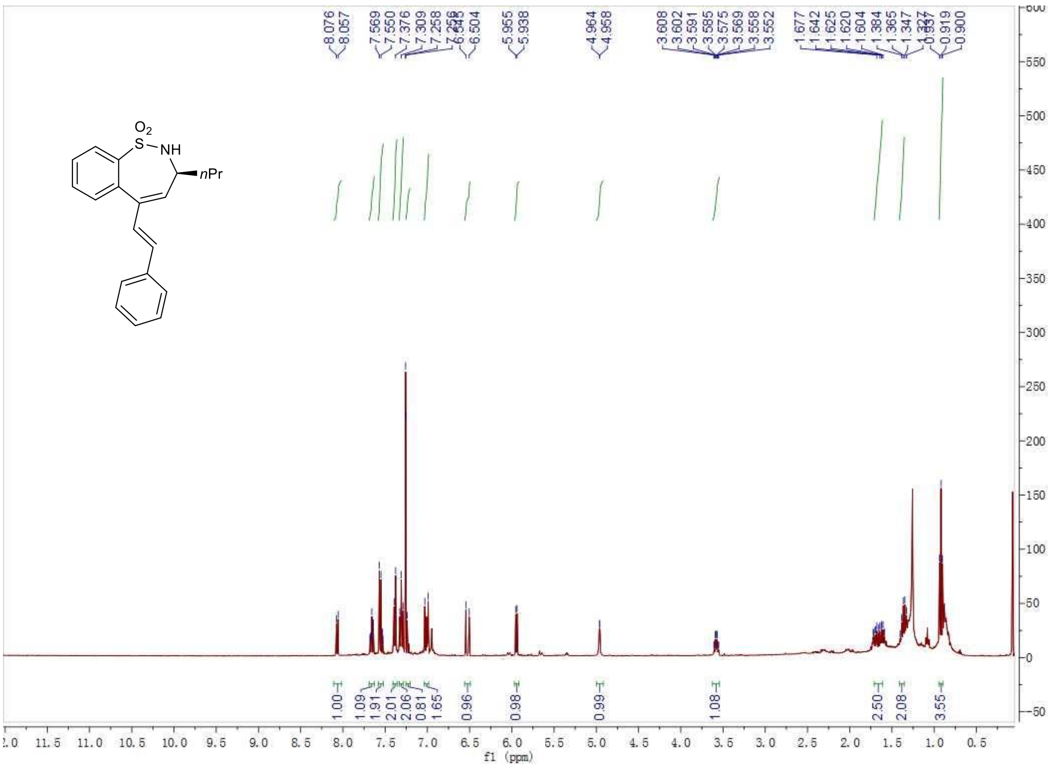

Supplementary Figure 148: ^{1}H NMR spectrum of compound **6a** in $CDCl_3$

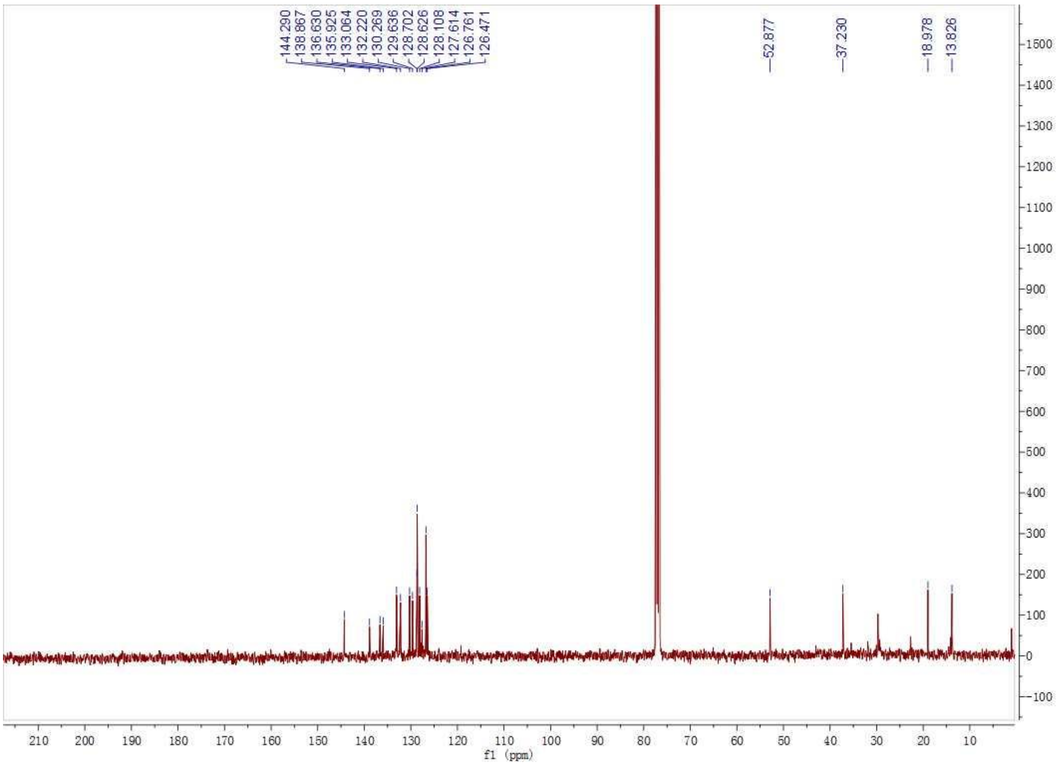

Supplementary Figure 149: ^{13}C NMR spectrum of compound **6a** in $CDCl_3$

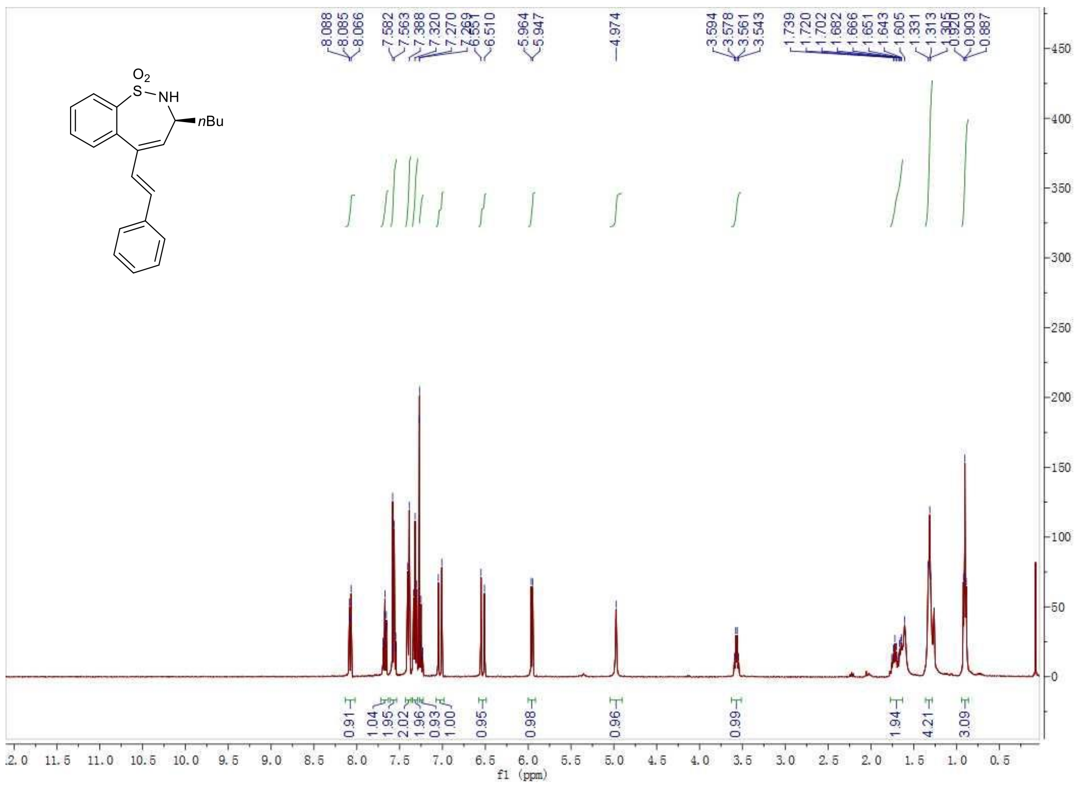

Supplementary Figure 150: ^{1}H NMR spectrum of compound **6b** in $CDCl_3$

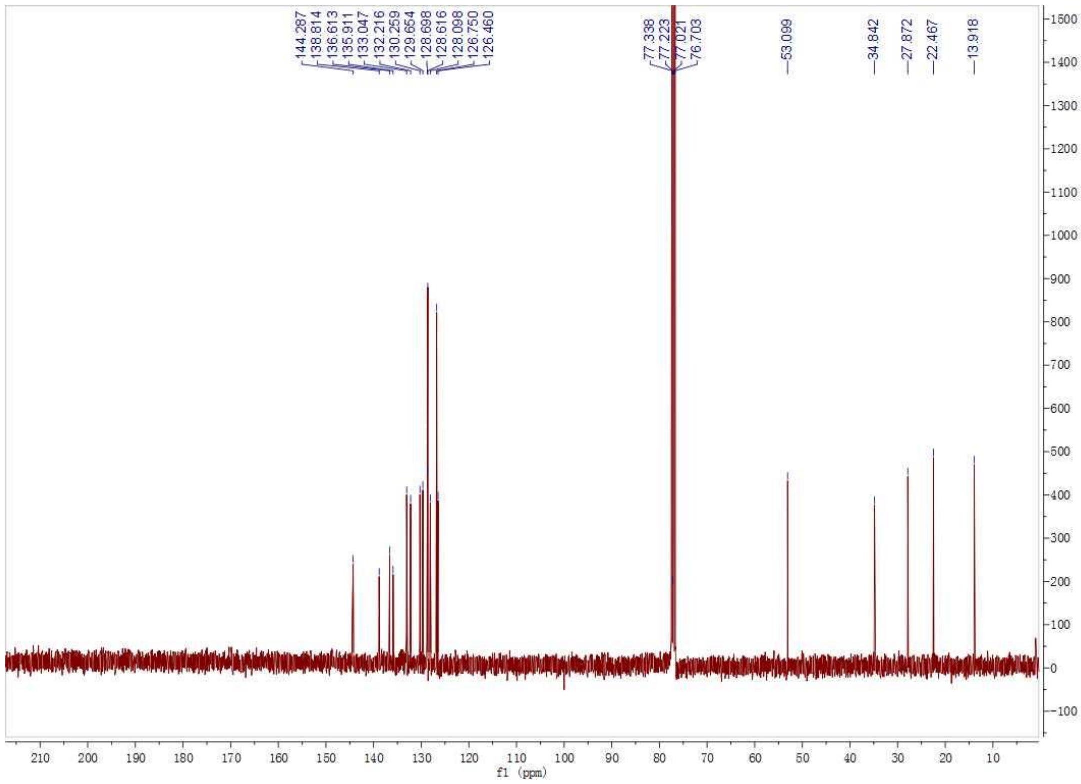

Supplementary Figure 151: ^{13}C NMR spectrum of compound **6b** in $CDCl_3$

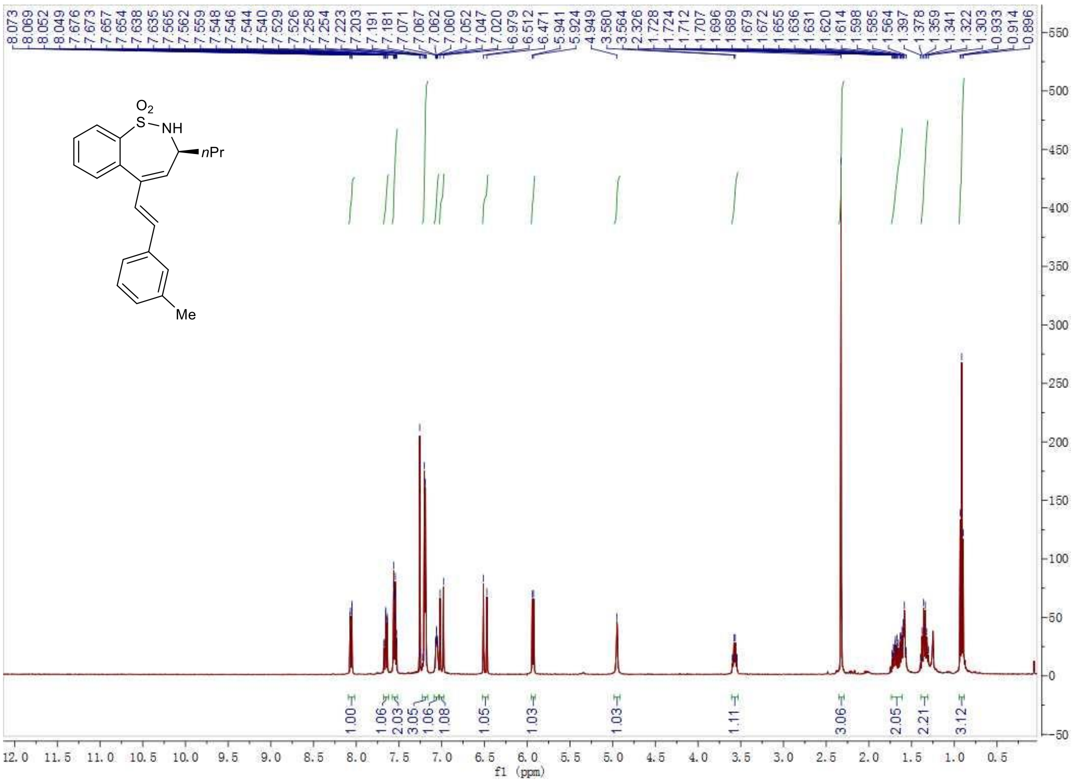

Supplementary Figure 152: ^{1}H NMR spectrum of compound **6d** in $CDCl_3$

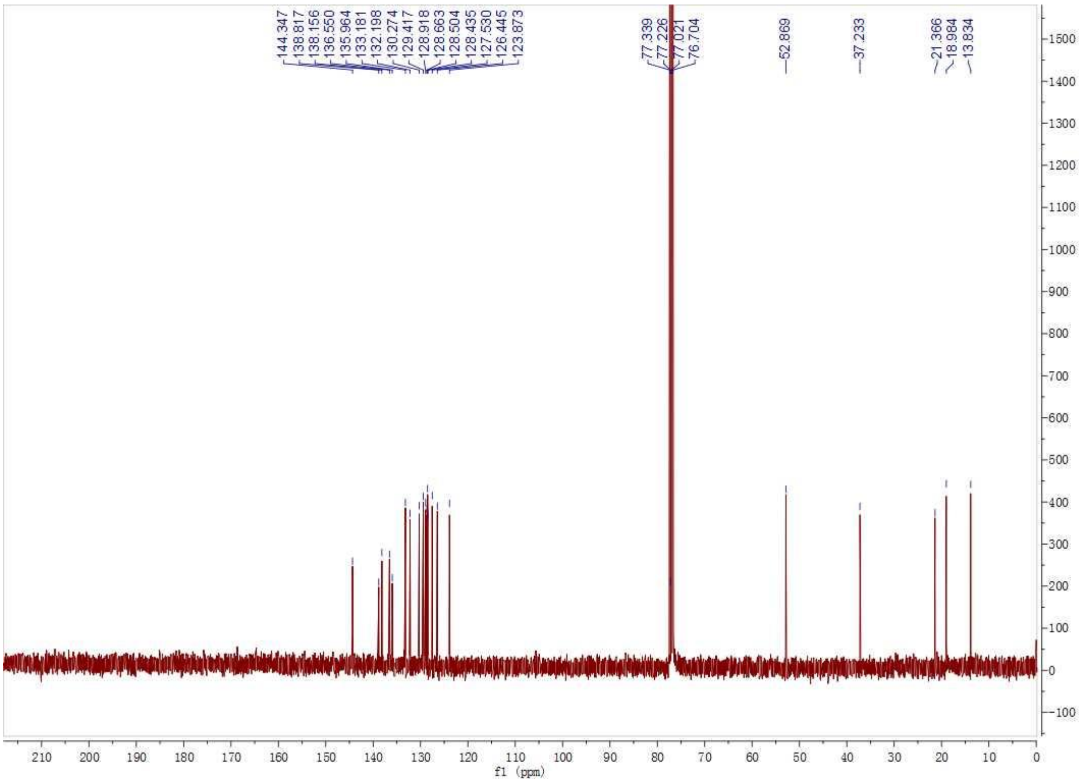

Supplementary Figure 153: ^{13}C NMR spectrum of compound **6d** in $CDCl_3$

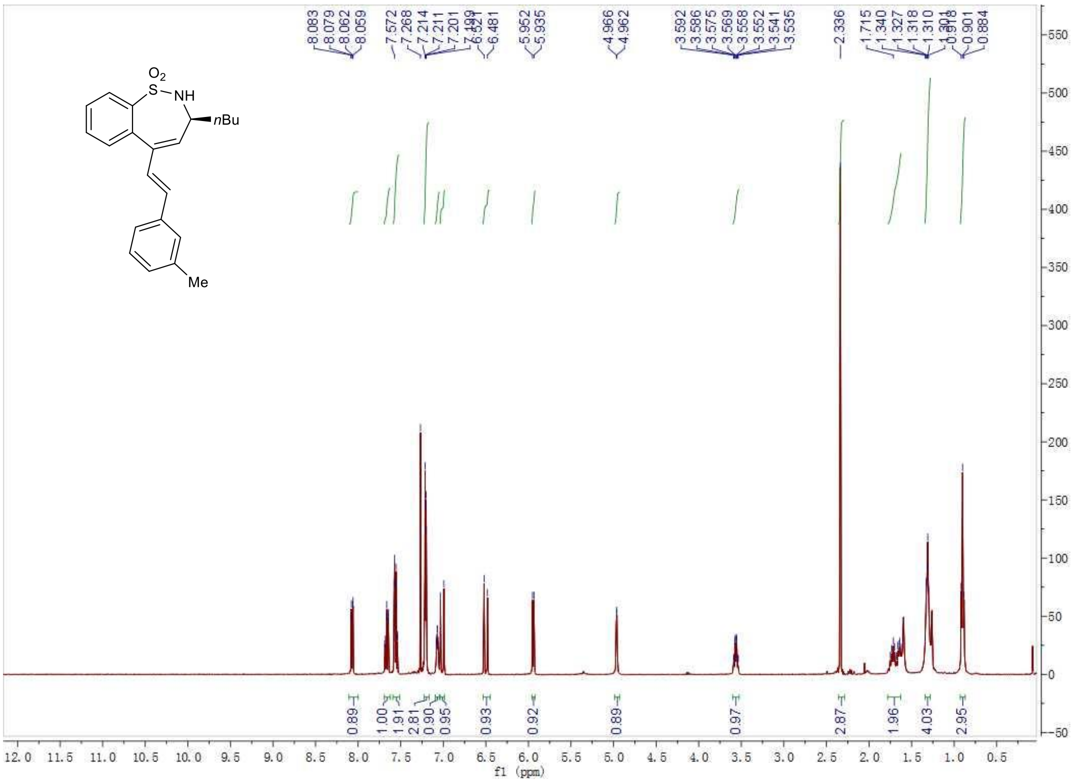

Supplementary Figure 154: ^{1}H NMR spectrum of compound **6e** in $CDCl_3$

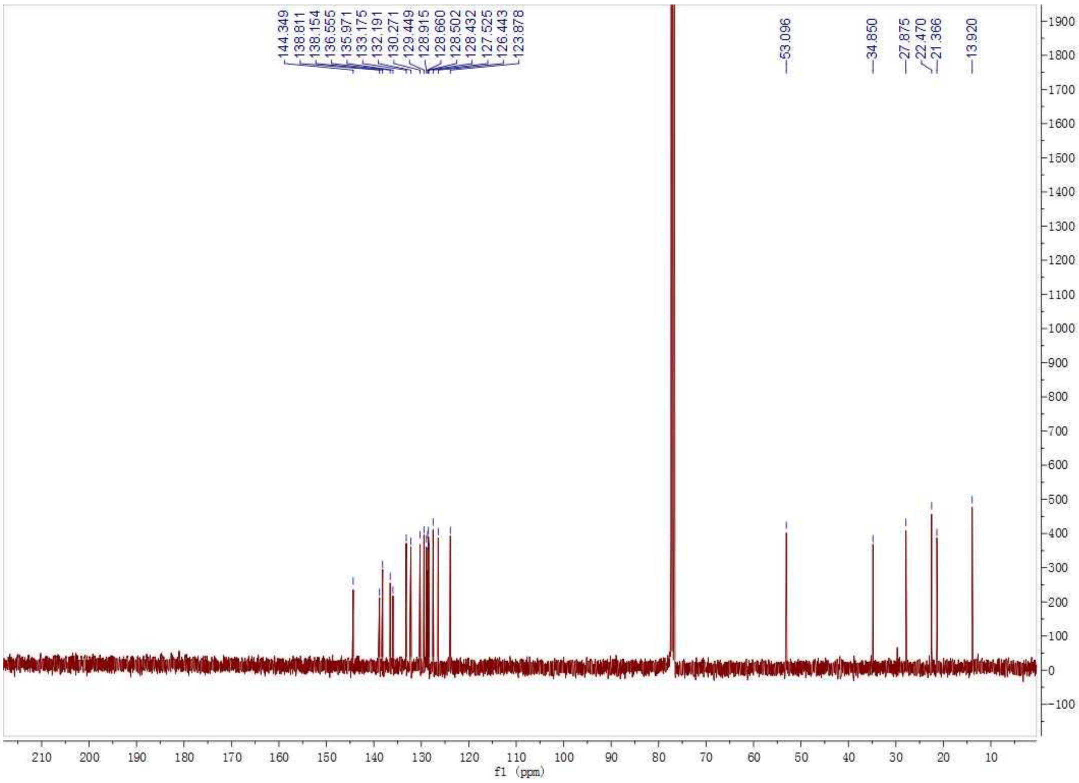

Supplementary Figure 155: ^{13}C NMR spectrum of compound **6e** in $CDCl_3$

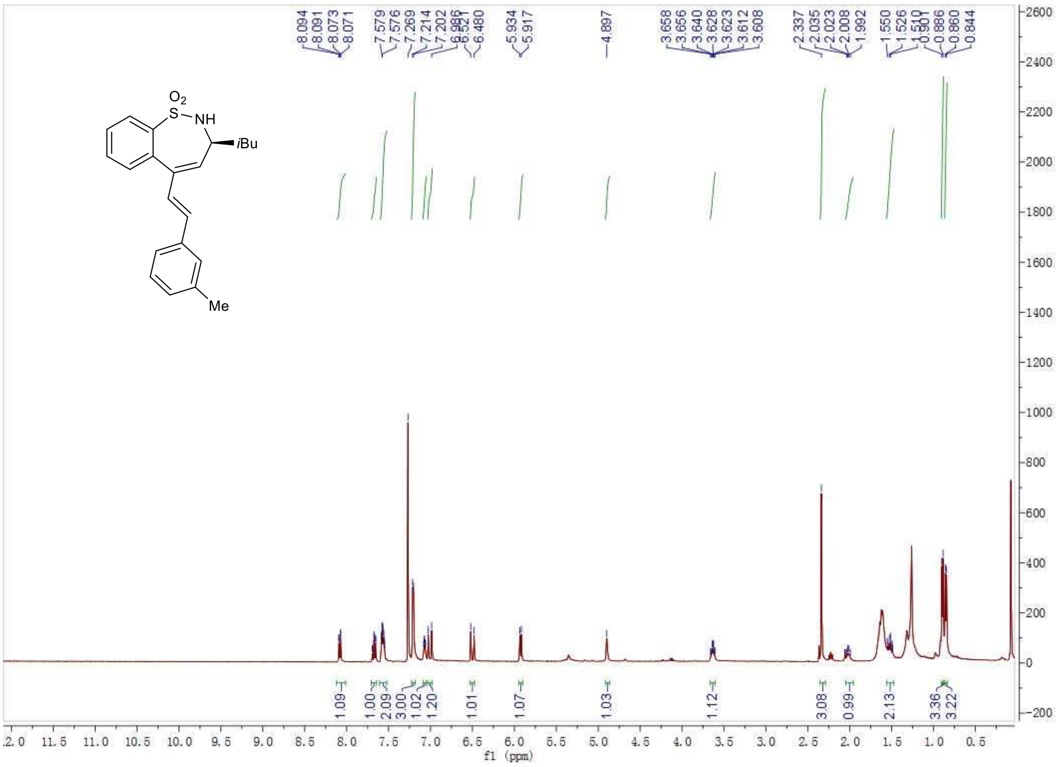

Supplementary Figure 156: ^{1}H NMR spectrum of compound **6f** in $CDCl_3$

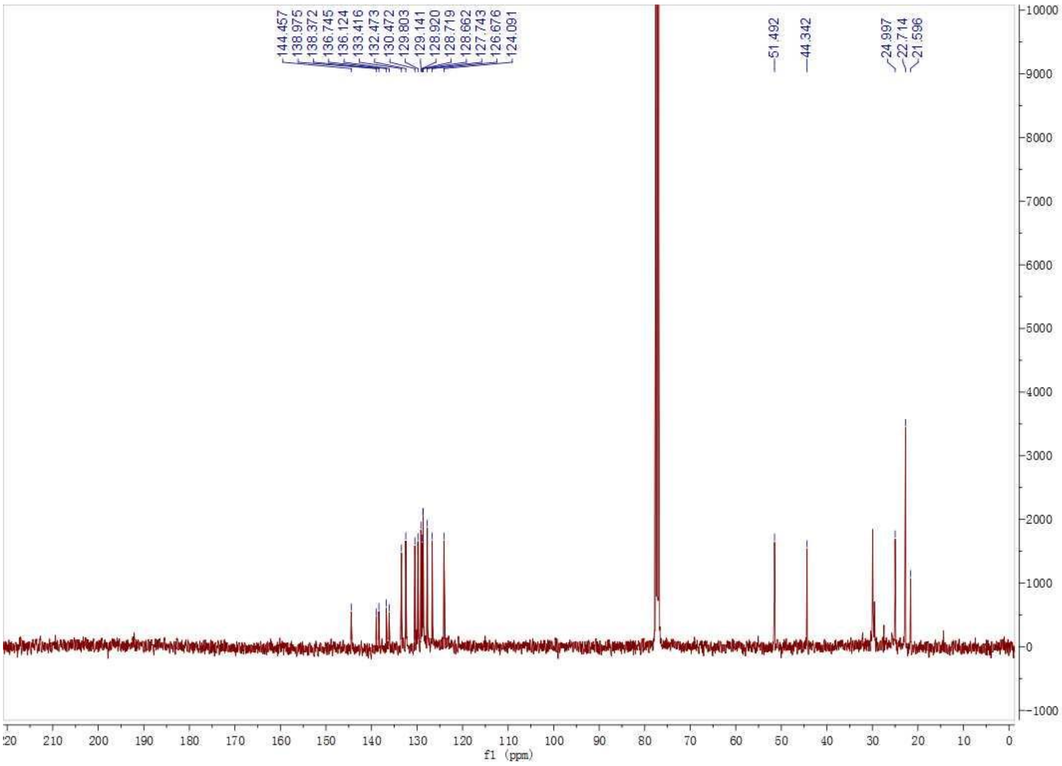

Supplementary Figure 157: ^{13}C NMR spectrum of compound **6f** in $CDCl_3$

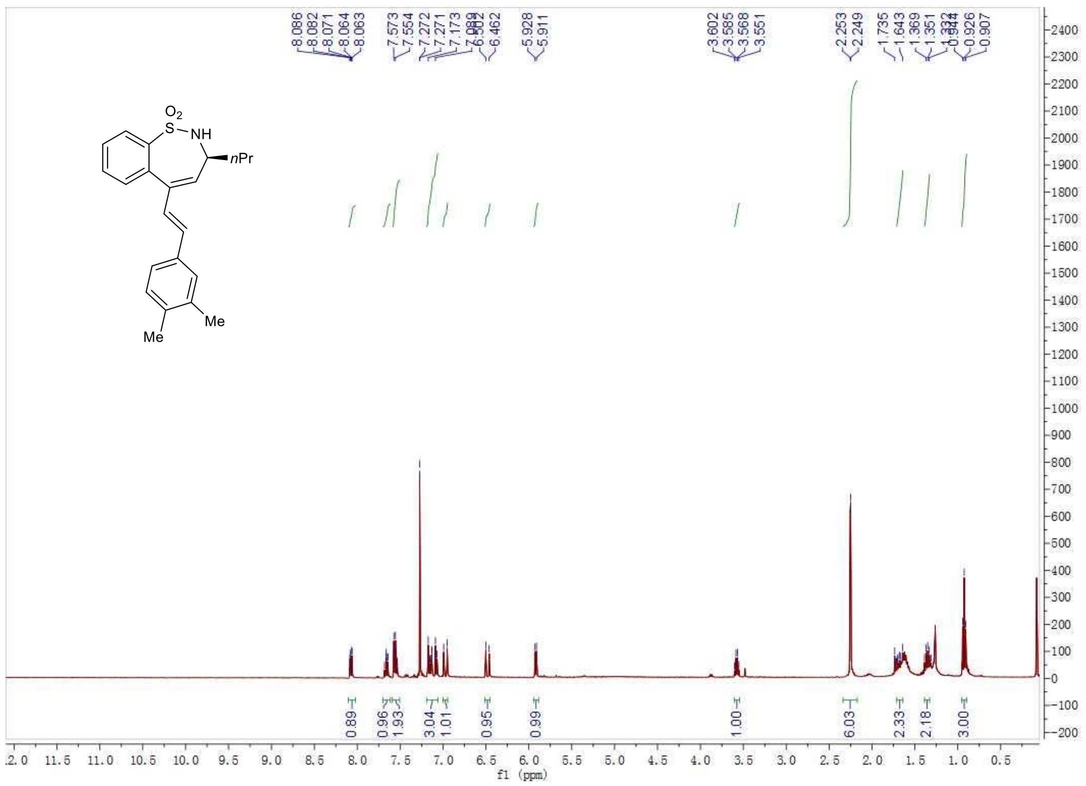

Supplementary Figure 158: ^{1}H NMR spectrum of compound **6g** in $CDCl_3$

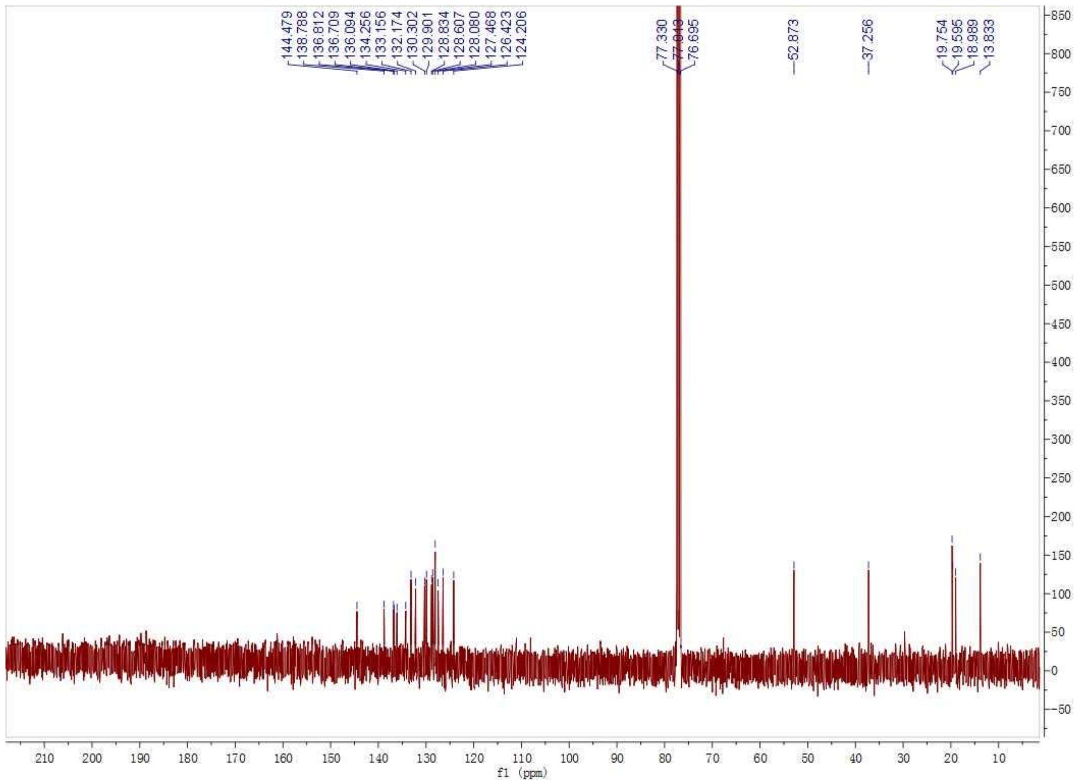

Supplementary Figure 159: ^{13}C NMR spectrum of compound **6g** in $CDCl_3$

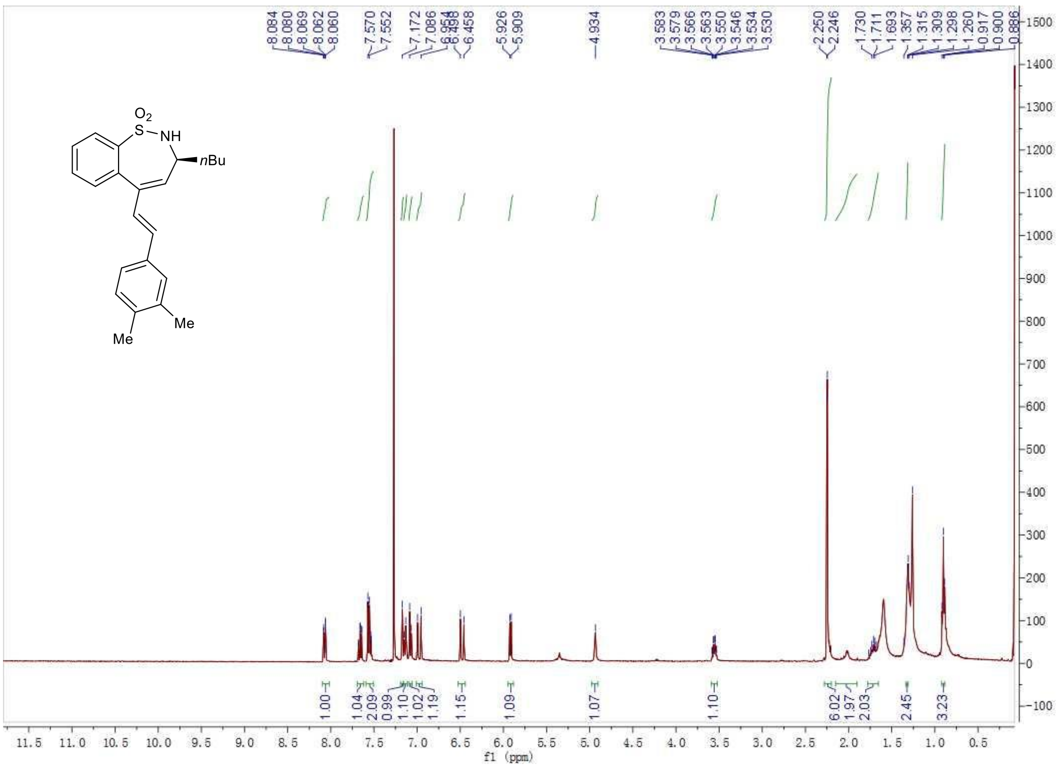

Supplementary Figure 160: ^{1}H NMR spectrum of compound **6h** in $CDCl_3$

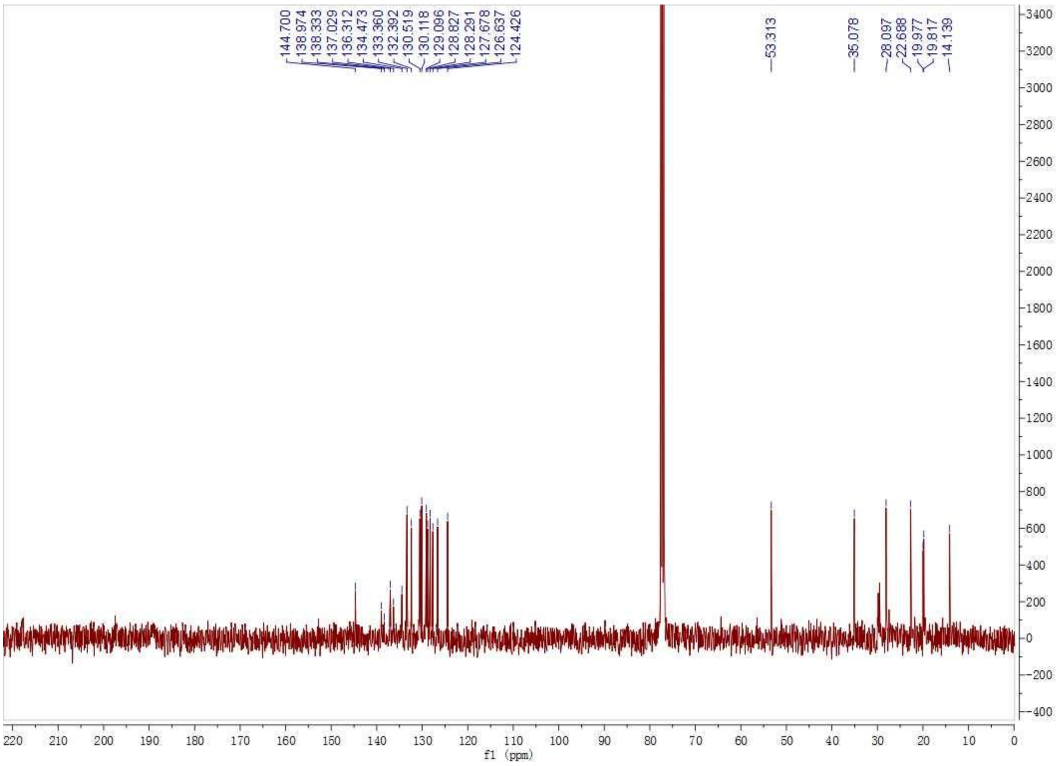

Supplementary Figure 161: ^{13}C NMR spectrum of compound **6h** in $CDCl_3$

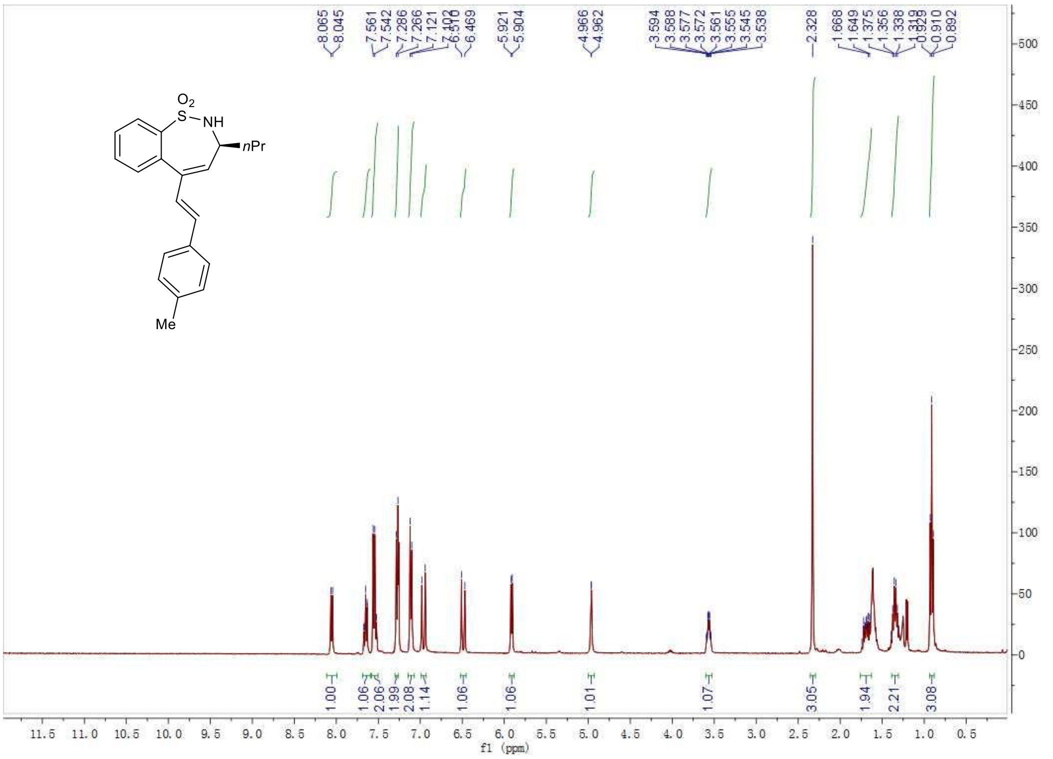

Supplementary Figure 162: ^{1}H NMR spectrum of compound **6i** in $CDCl_3$

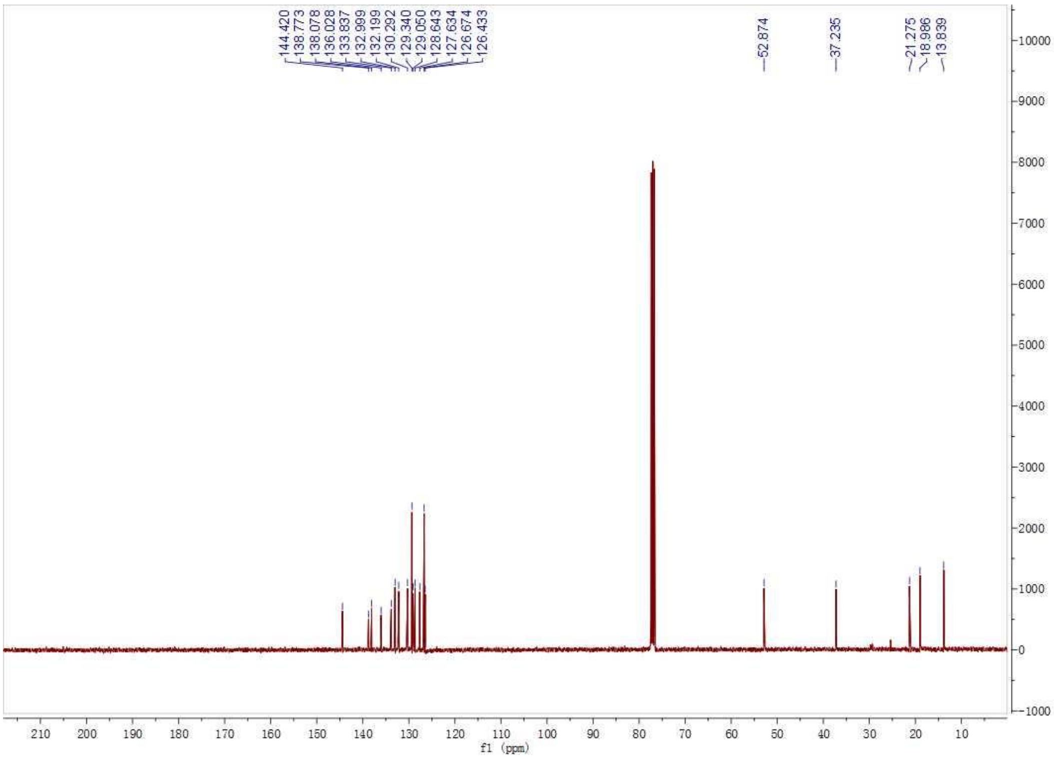

Supplementary Figure 163: ^{13}C NMR spectrum of compound **6i** in $CDCl_3$

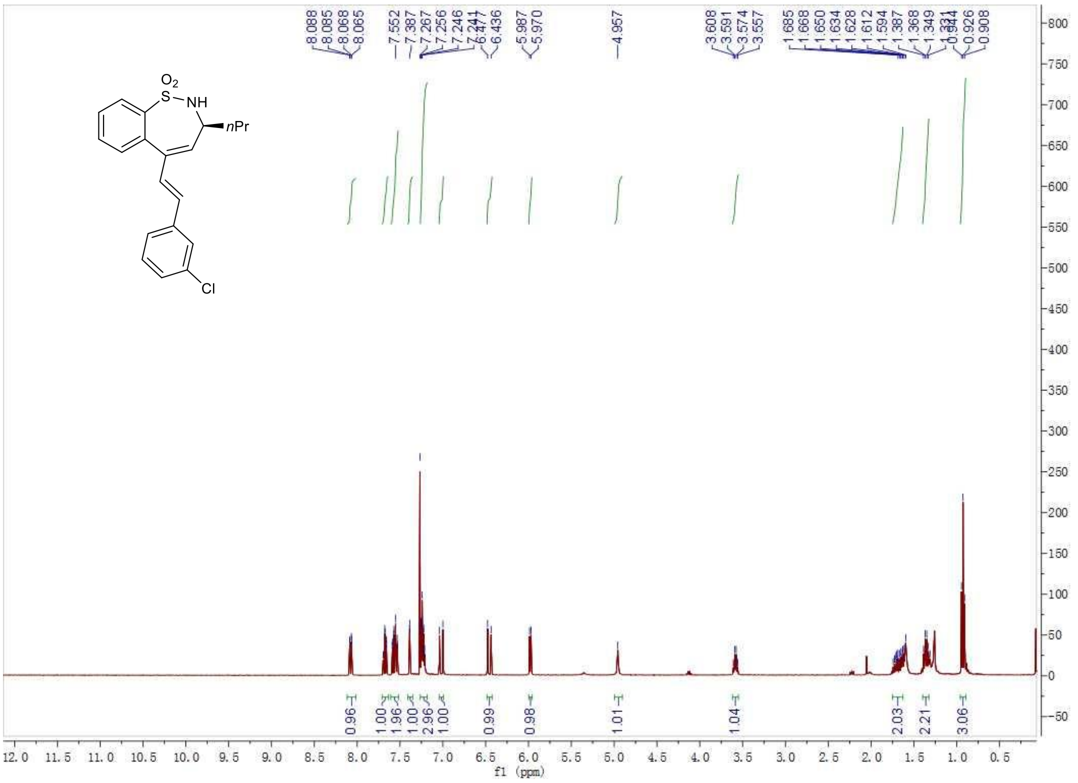

Supplementary Figure 164: ^{1}H NMR spectrum of compound **6j** in $CDCl_3$

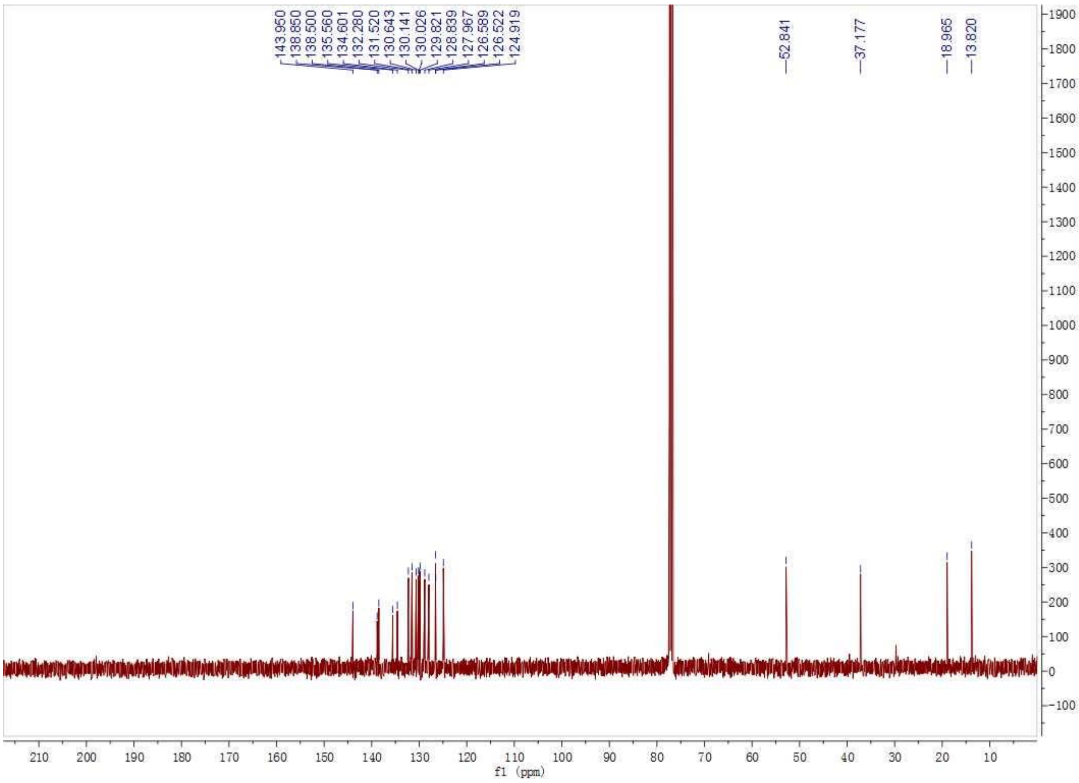

Supplementary Figure 165: ^{13}C NMR spectrum of compound **6j** in $CDCl_3$

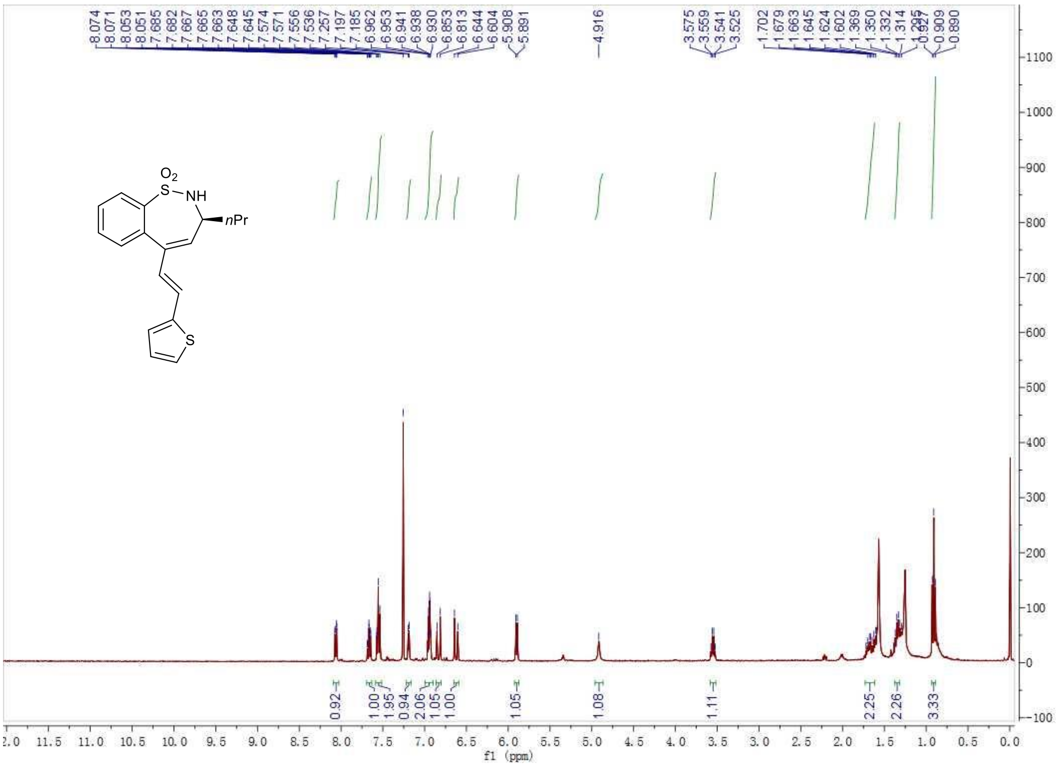

Supplementary Figure 166: ^{1}H NMR spectrum of compound **6k** in $CDCl_3$

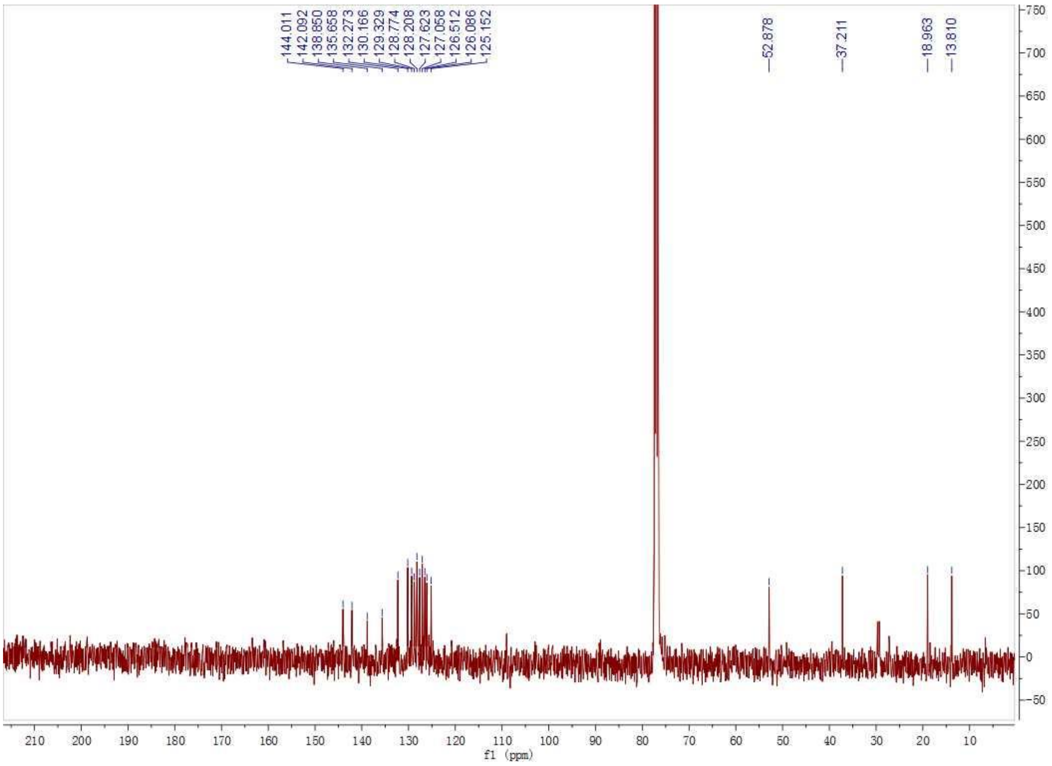

Supplementary Figure 167: ^{13}C NMR spectrum of compound **6k** in $CDCl_3$

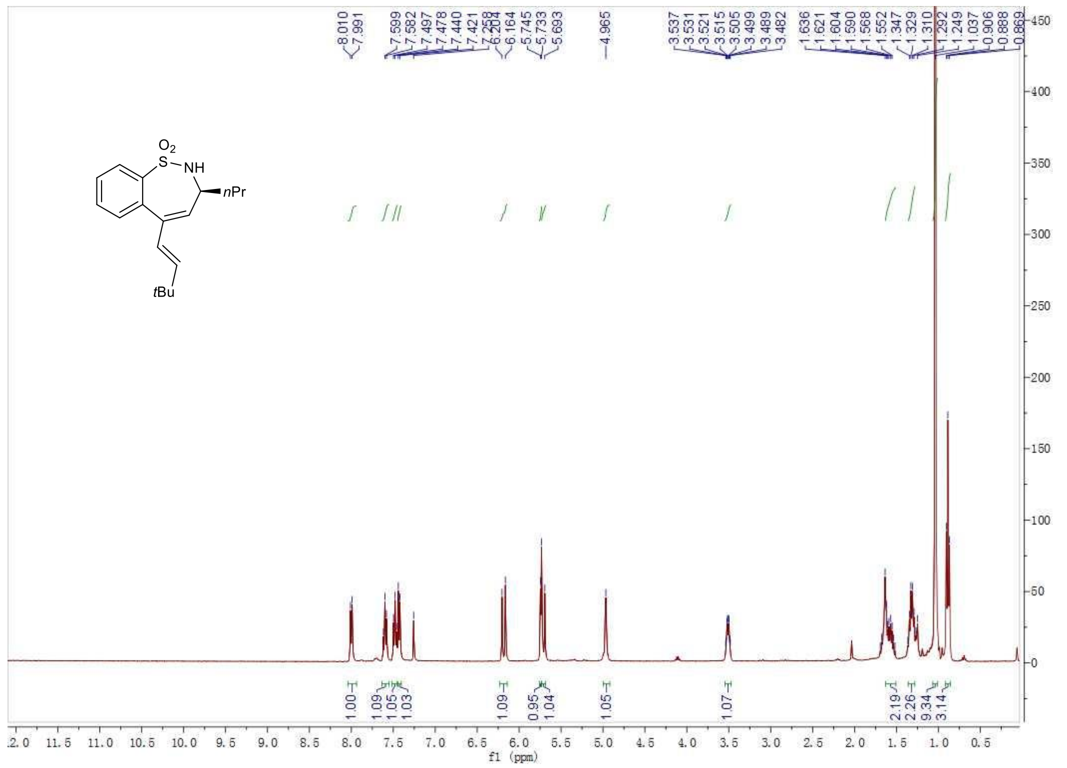

Supplementary Figure 168: ^{1}H NMR spectrum of compound **6l** in $CDCl_3$

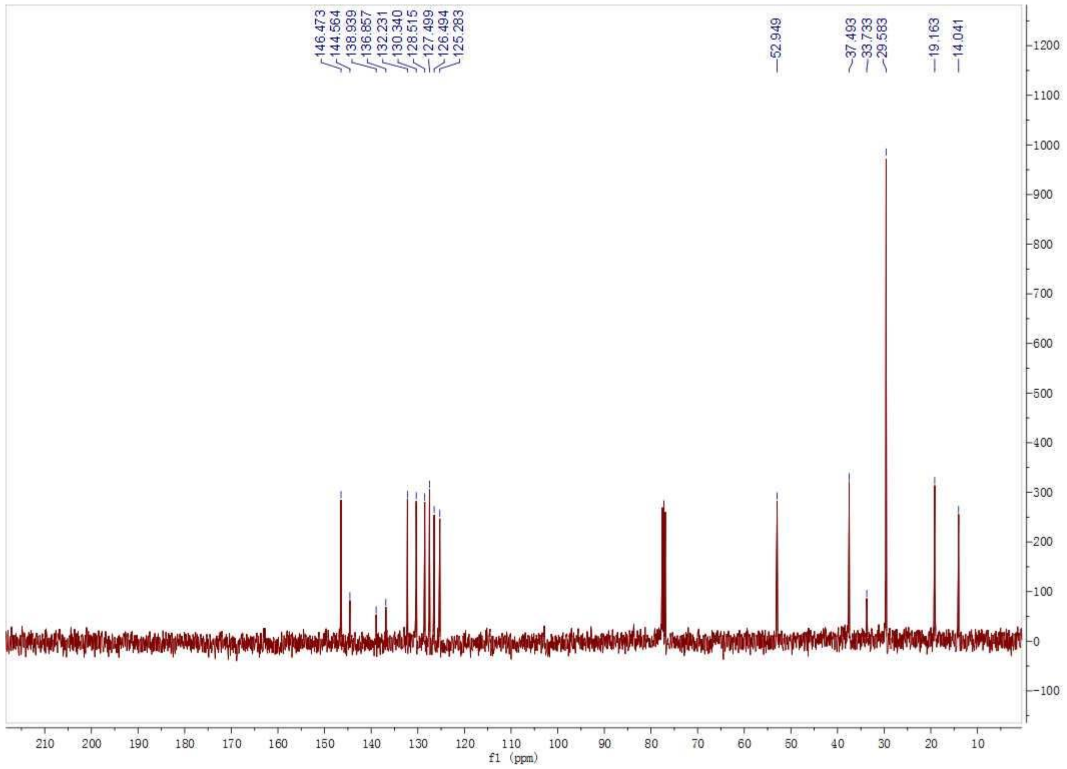

Supplementary Figure 169: ^{13}C NMR spectrum of compound **6l** in $CDCl_3$

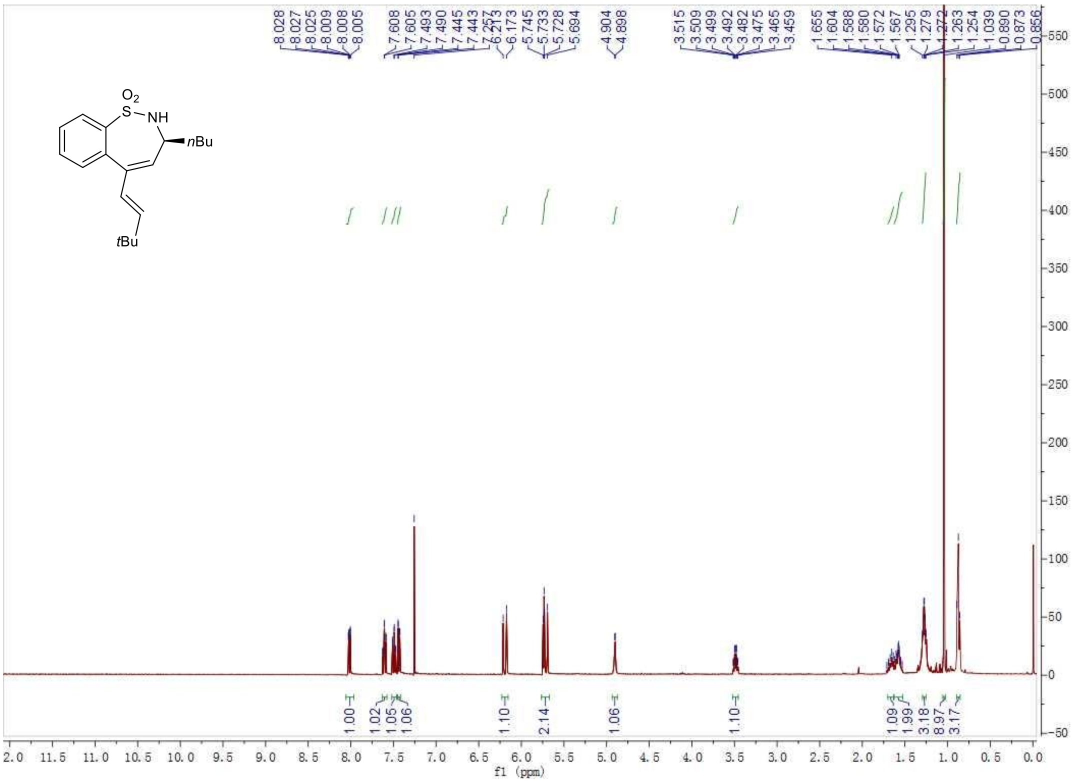

Supplementary Figure 170: ^{1}H NMR spectrum of compound **6m** in $CDCl_3$

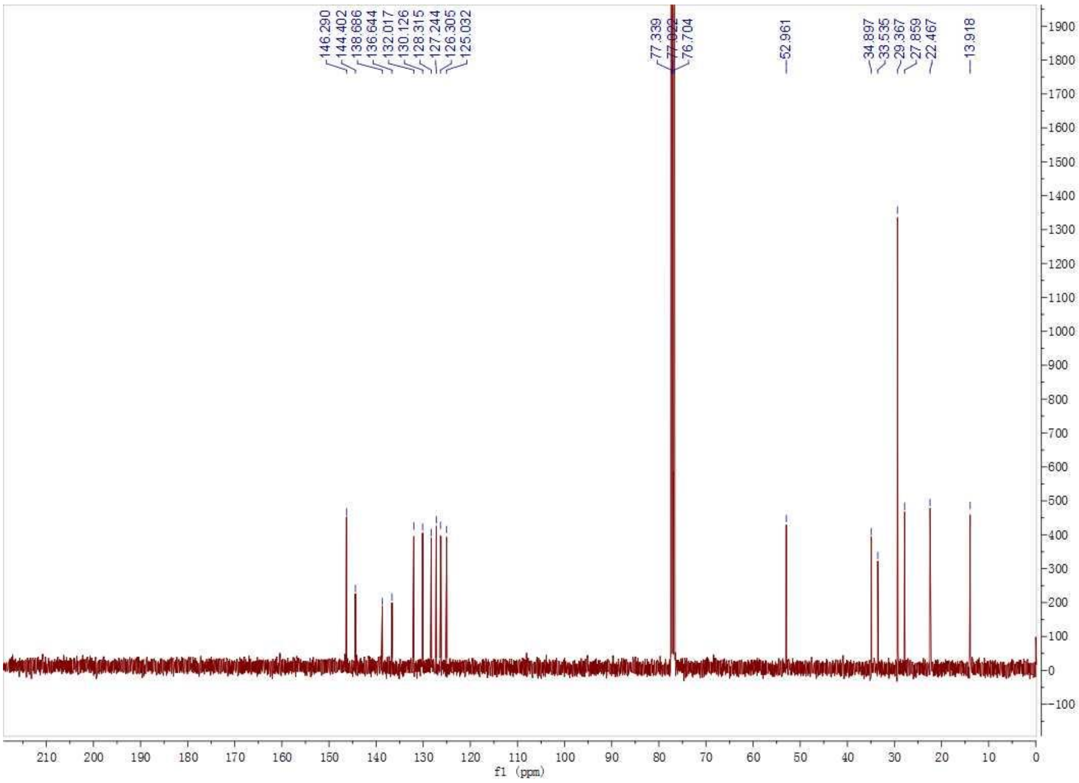

Supplementary Figure 171: ^{13}C NMR spectrum of compound **6m** in $CDCl_3$

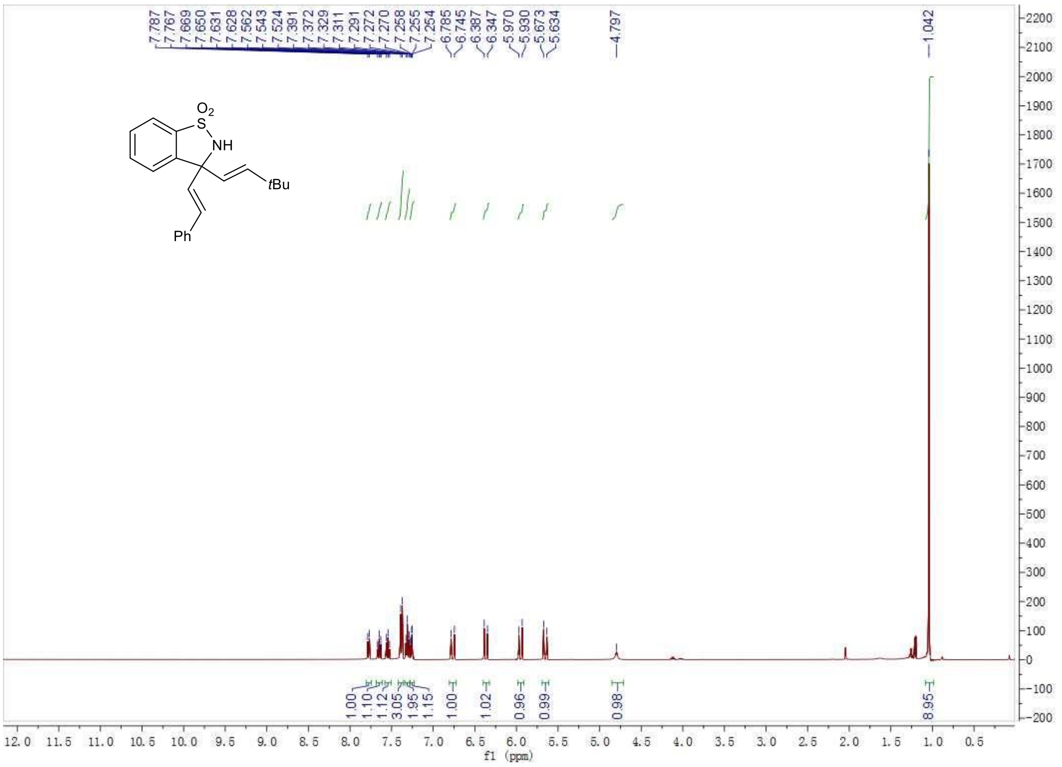

Supplementary Figure 172: ^{1}H NMR spectrum of compound **7** in $CDCl_3$

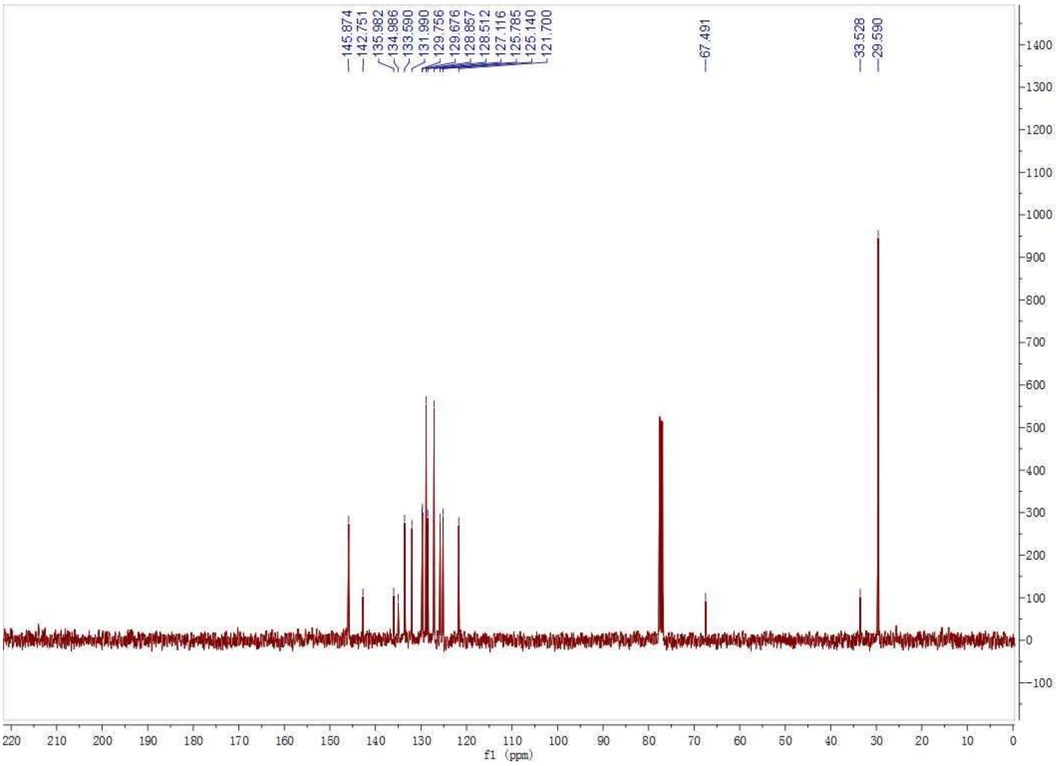

Supplementary Figure 173: ^{13}C NMR spectrum of compound **7** in $CDCl_3$

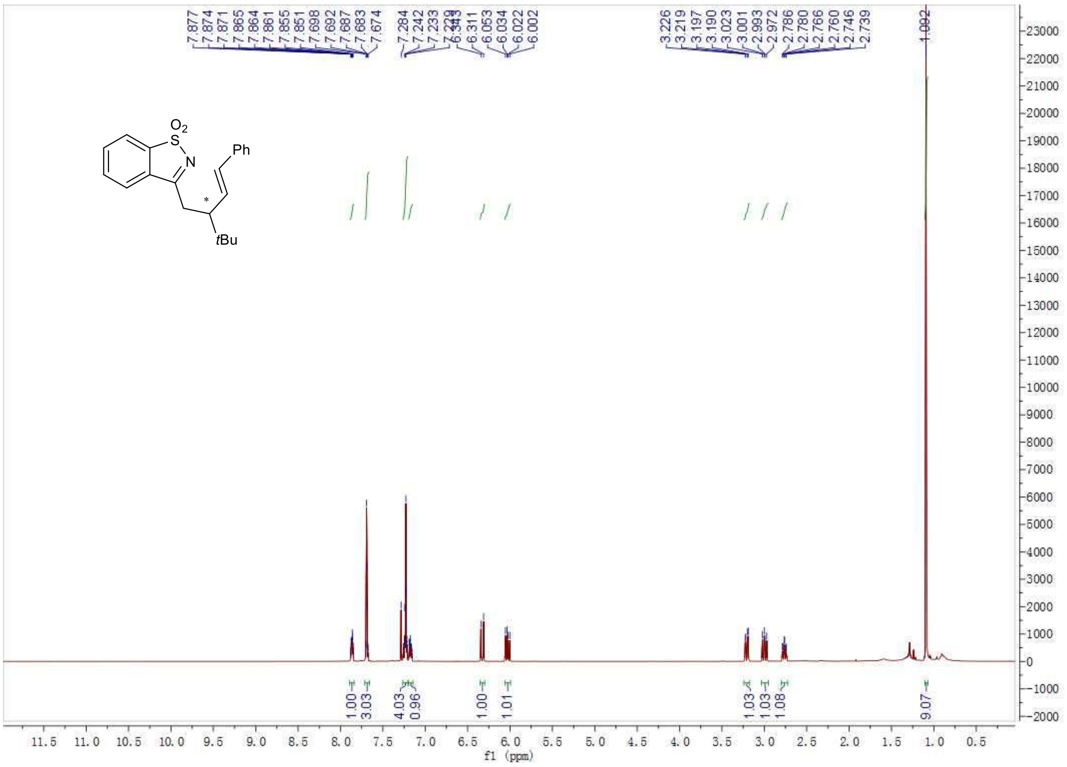

Supplementary Figure 174: ^{1}H NMR spectrum of compound **9** in $CDCl_3$

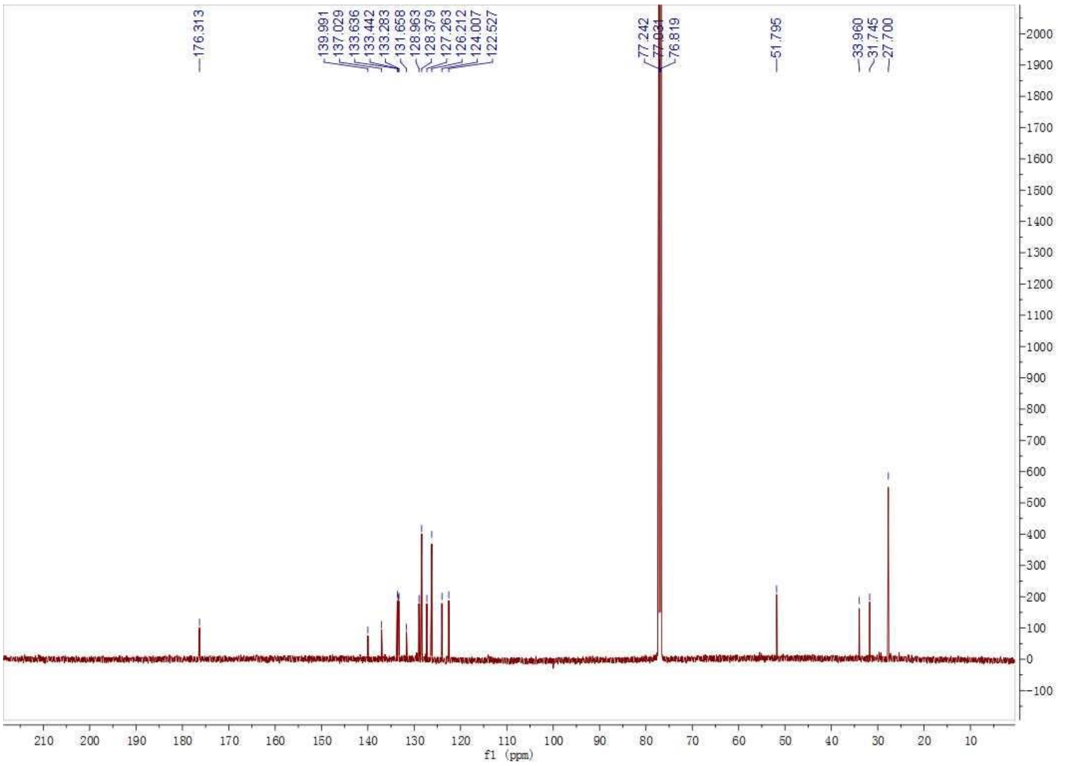

Supplementary Figure 175: ^{13}C NMR spectrum of compound **9** in $CDCl_3$

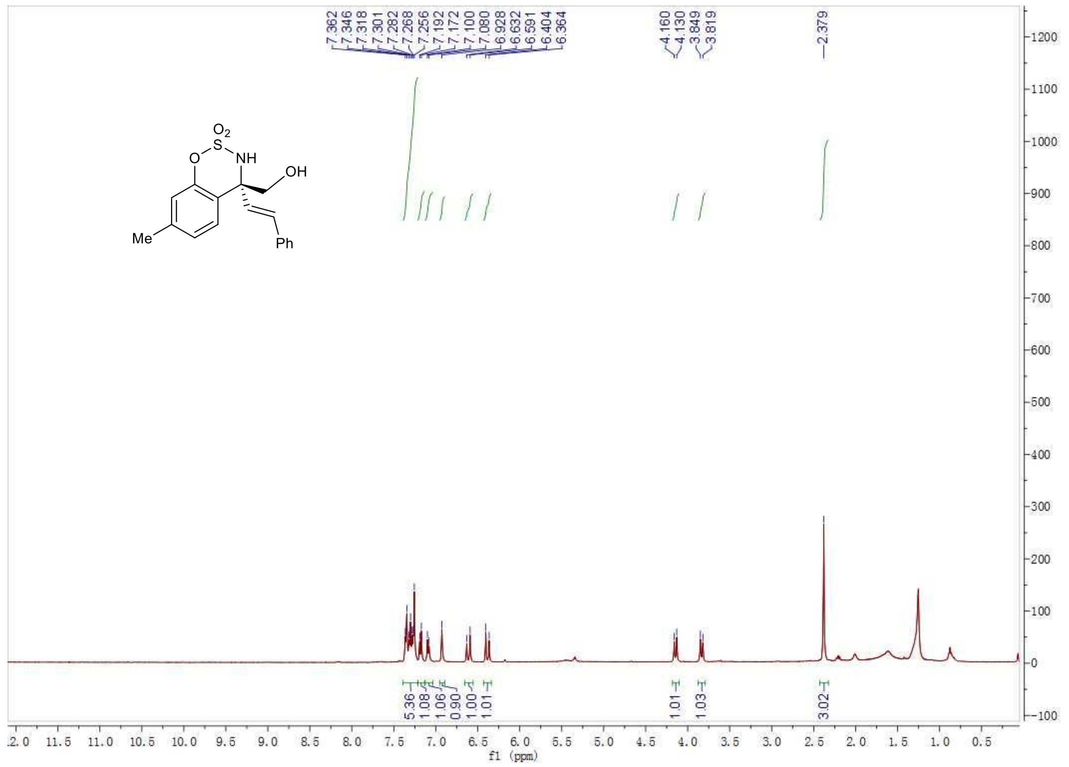

Supplementary Figure 176: ^{1}H NMR spectrum of compound **10** in $CDCl_3$

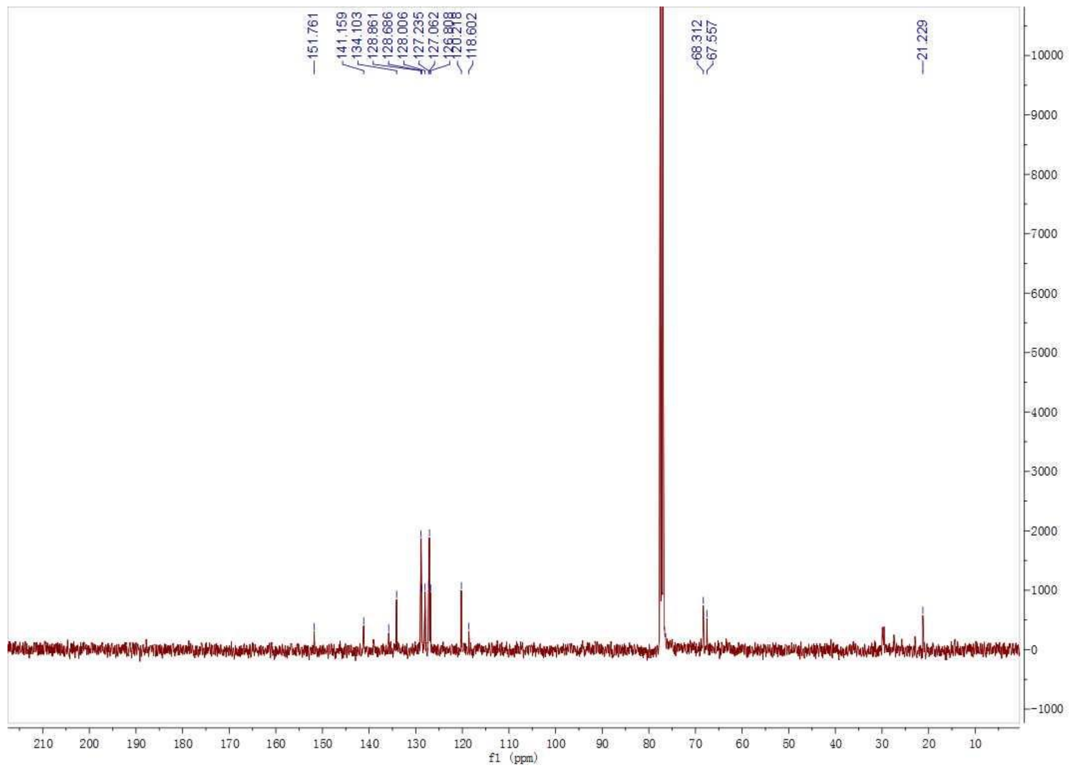

Supplementary Figure 177: ^{13}C NMR spectrum of compound **10** in $CDCl_3$

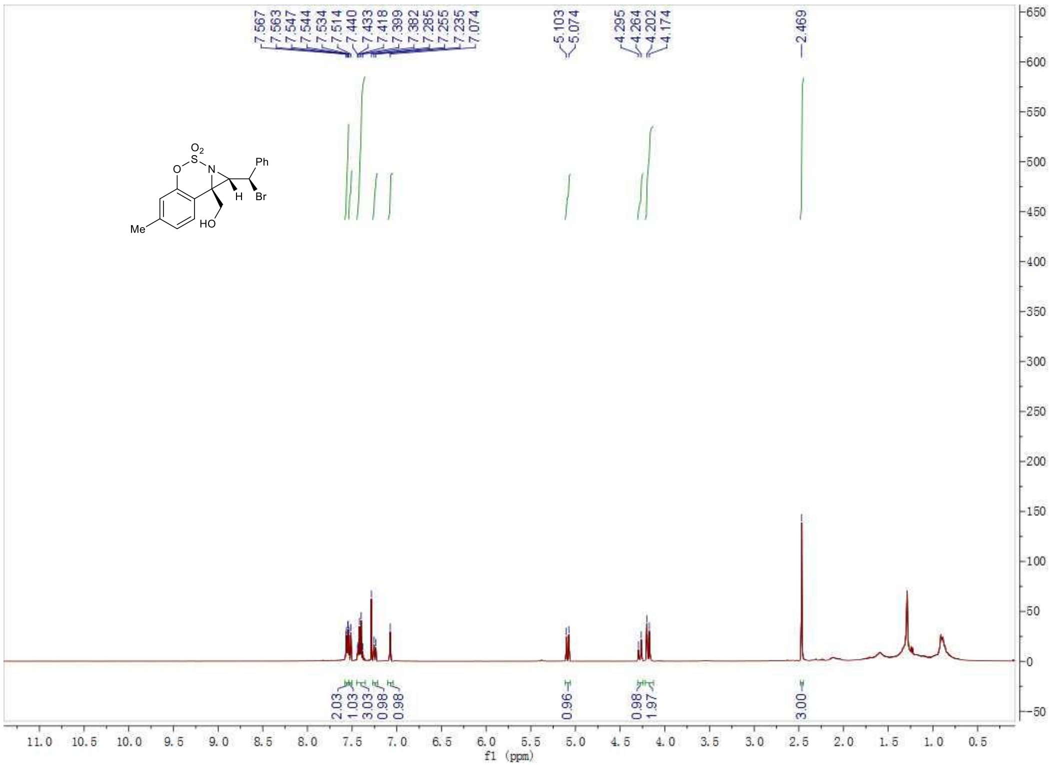

Supplementary Figure 178: ^{1}H NMR spectrum of compound **11** in $CDCl_3$

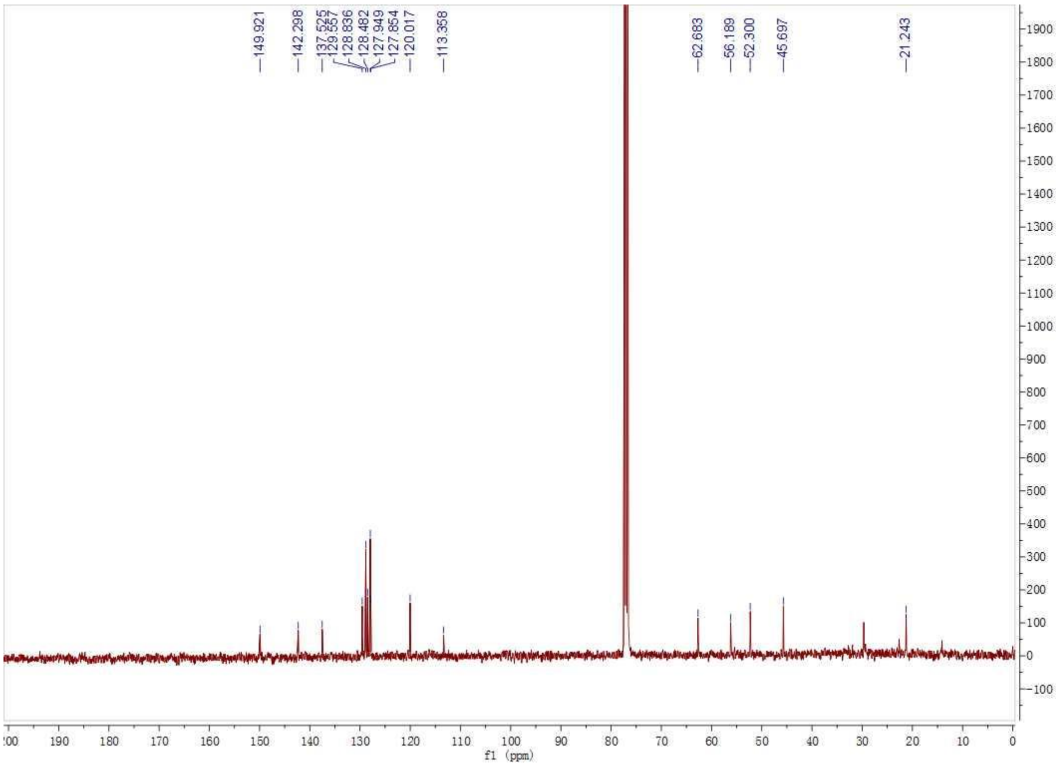

Supplementary Figure 179: ^{13}C NMR spectrum of compound **11** in $CDCl_3$

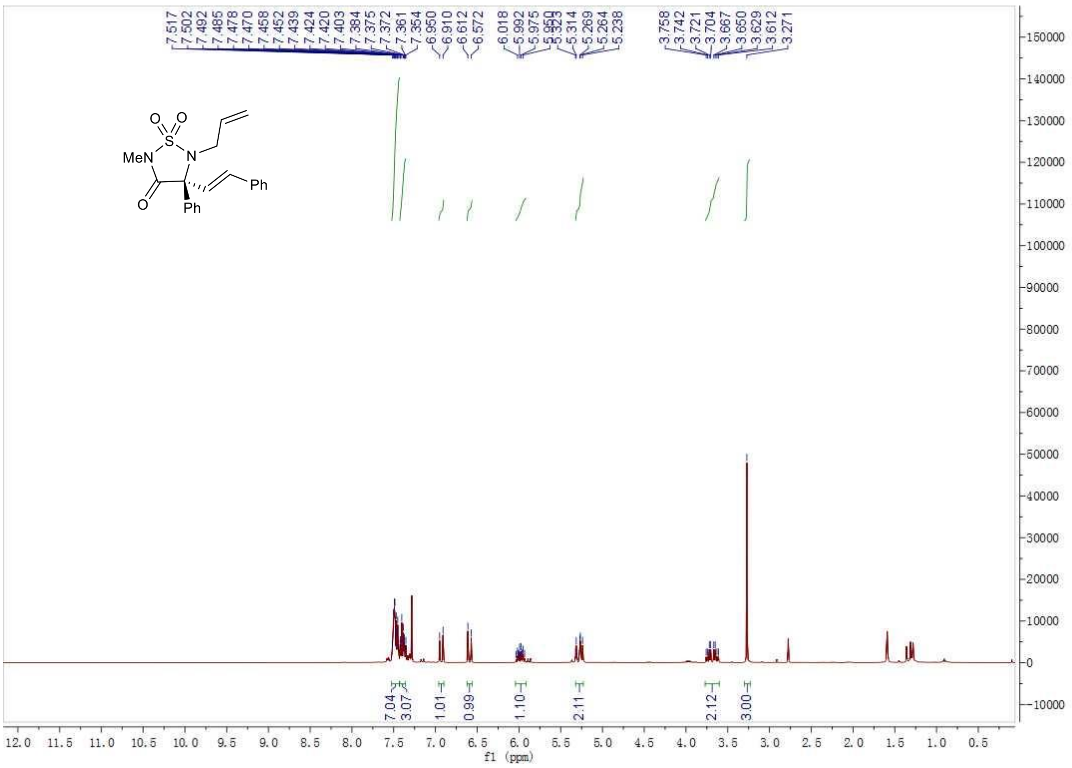

Supplementary Figure 180: ^{1}H NMR spectrum of compound **12** in $CDCl_3$

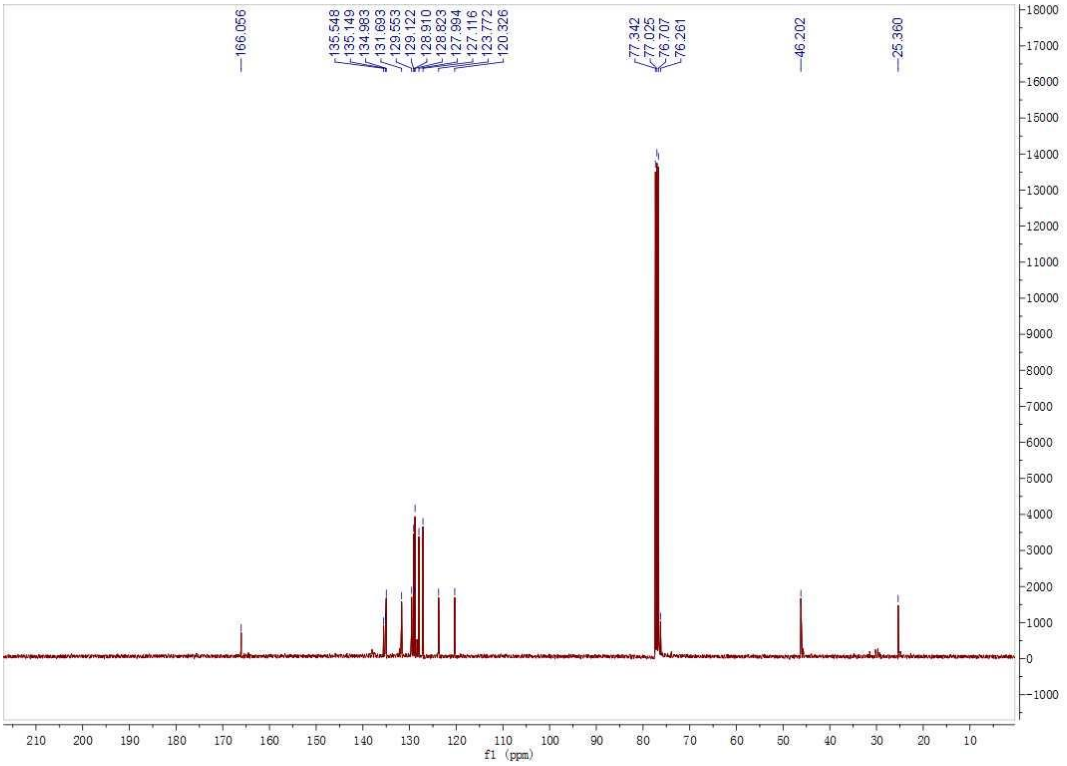

Supplementary Figure 181: ^{13}C NMR spectrum of compound **12** in $CDCl_3$

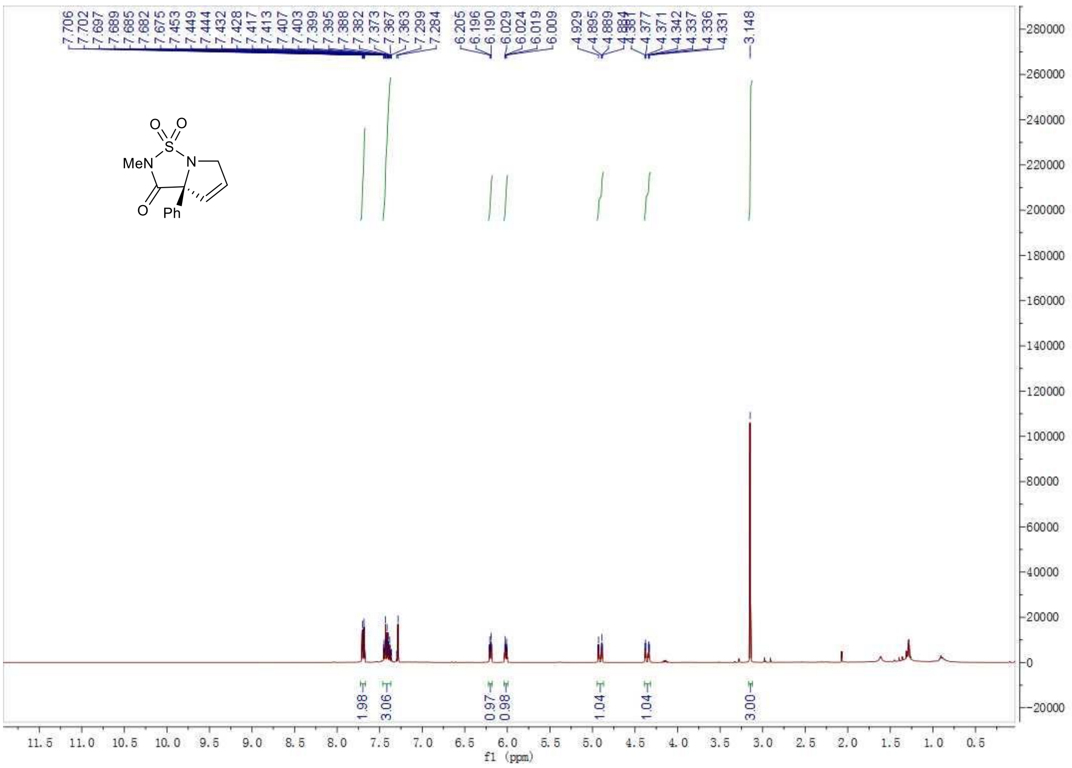

Supplementary Figure 182: ^{1}H NMR spectrum of compound **13** in $CDCl_3$

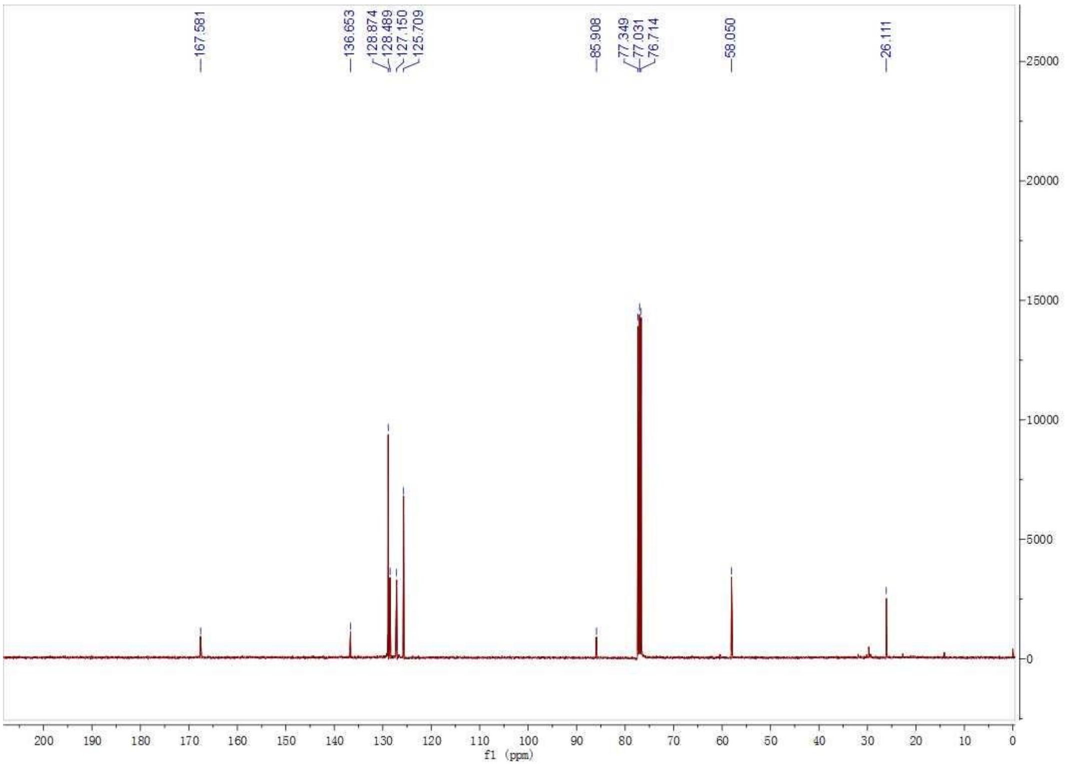

Supplementary Figure 183: ^{13}C NMR spectrum of compound **13** in $CDCl_3$

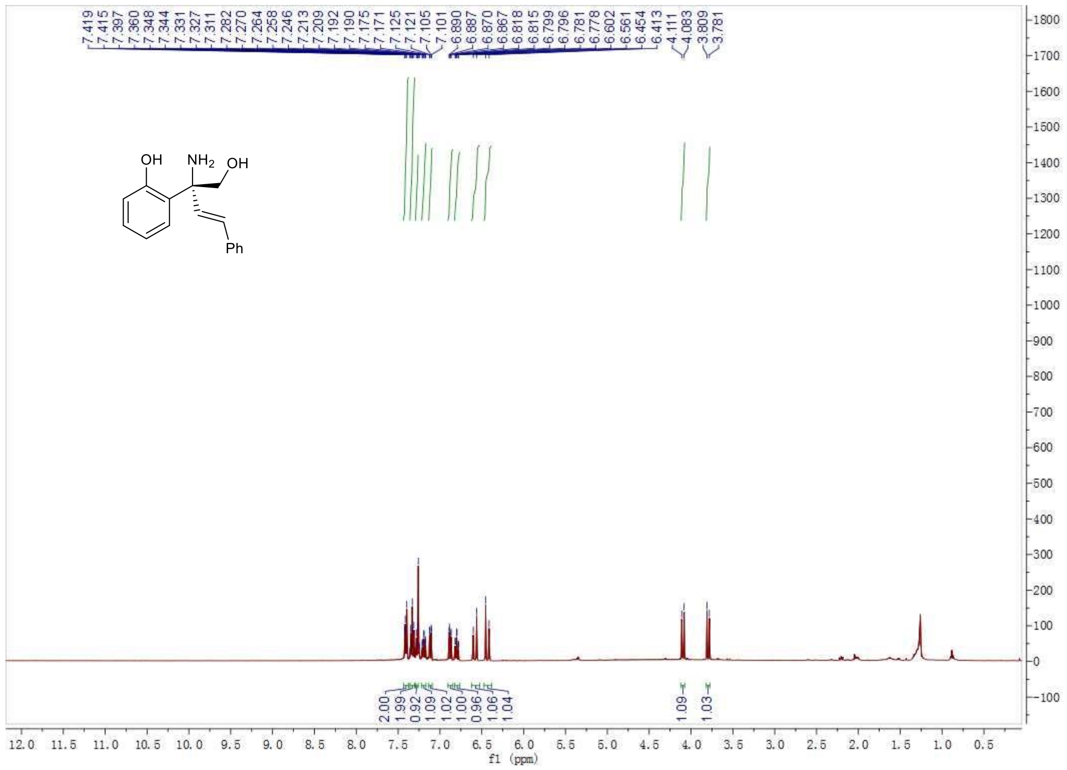

Supplementary Figure 184: ^{1}H NMR spectrum of compound **SI-6** in $CDCl_3$

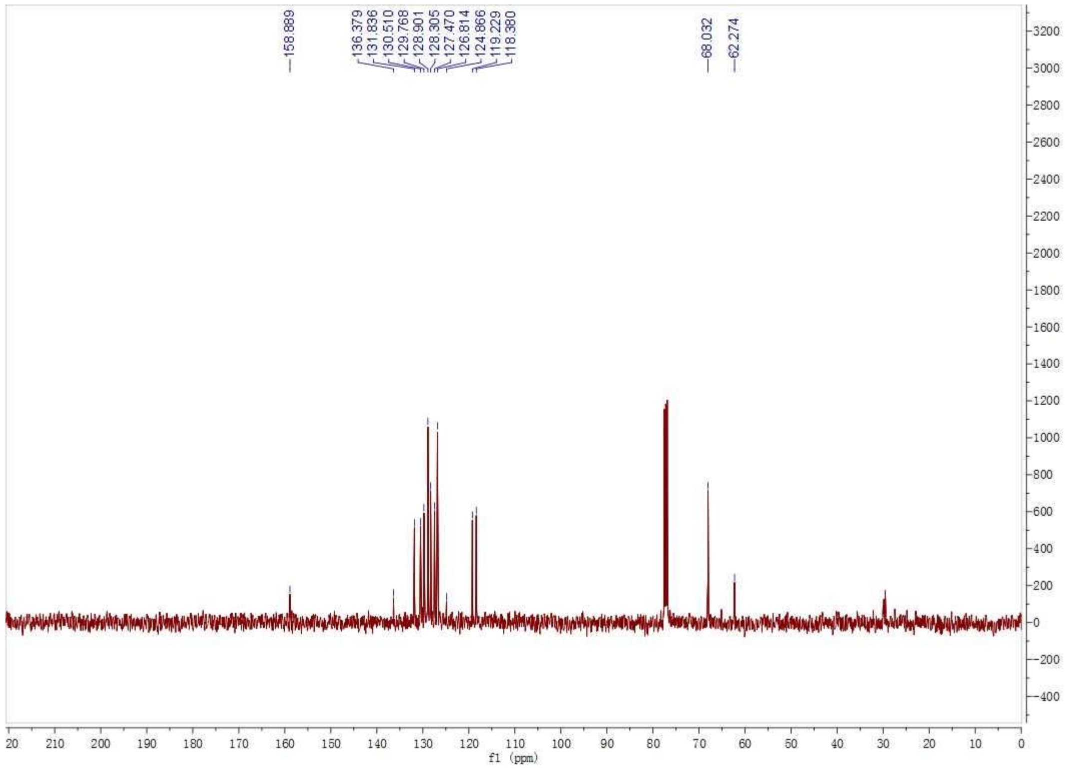

Supplementary Figure 185: ^{13}C NMR spectrum of compound **SI-6** in $CDCl_3$

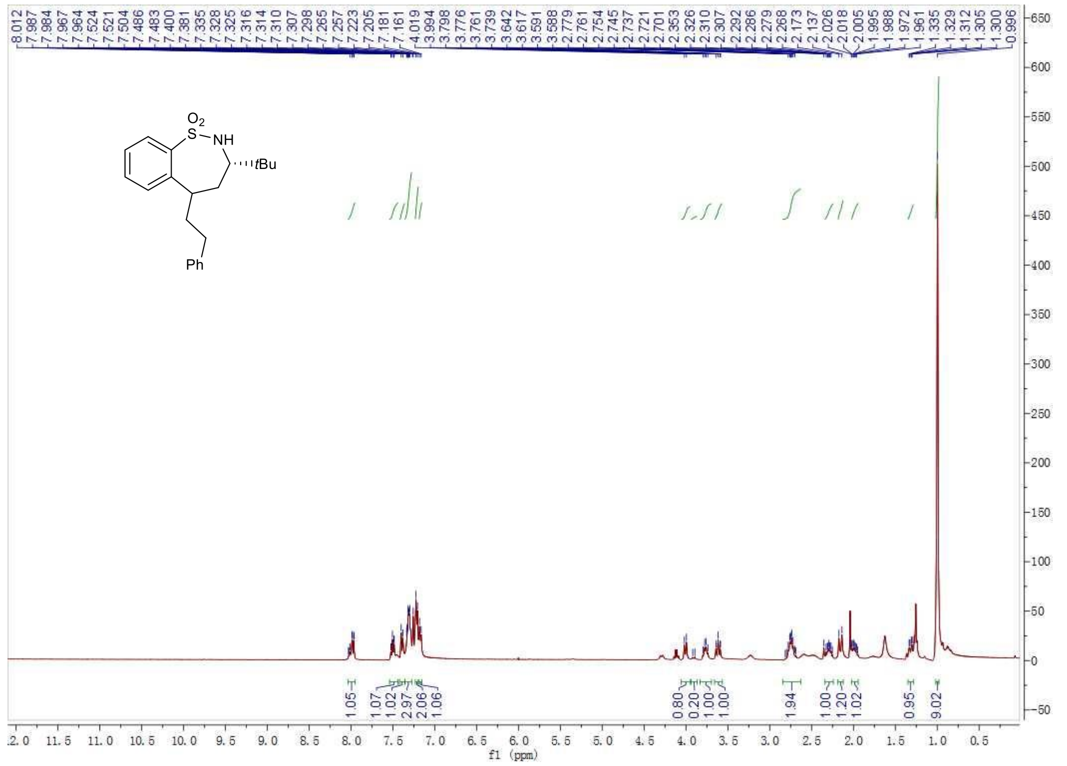

Supplementary Figure 186: ^{1}H NMR spectrum of compound **14** in $CDCl_3$

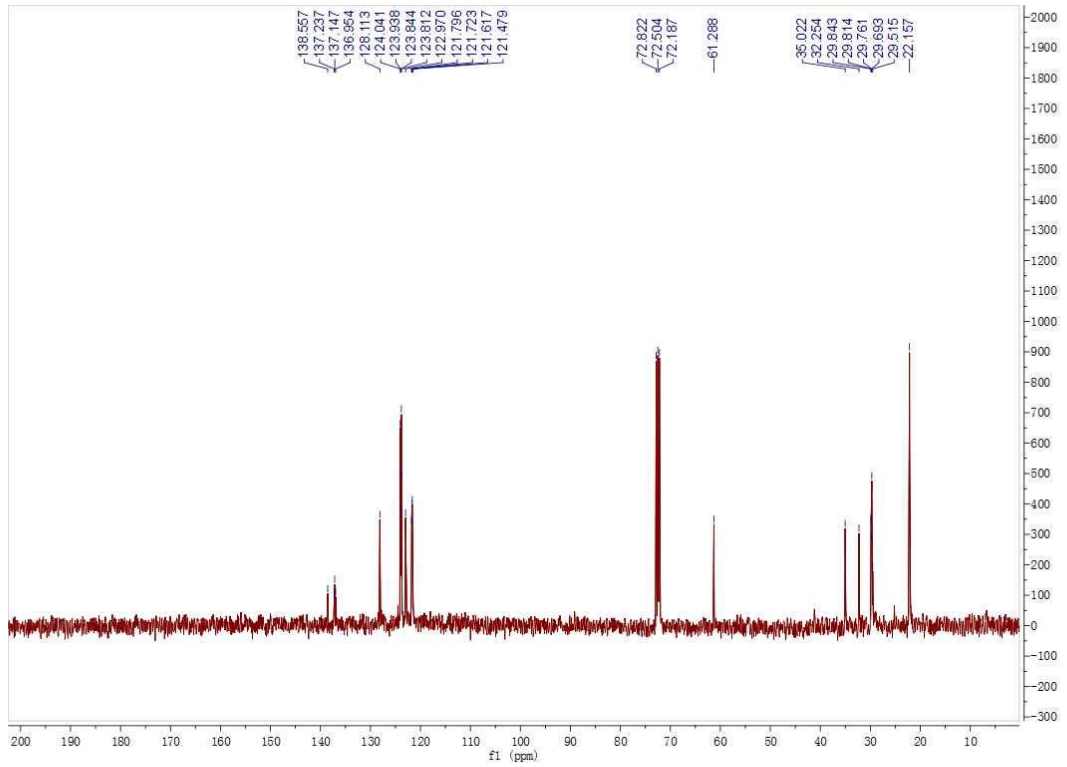

Supplementary Figure 187: ^{13}C NMR spectrum of compound **14** in $CDCl_3$

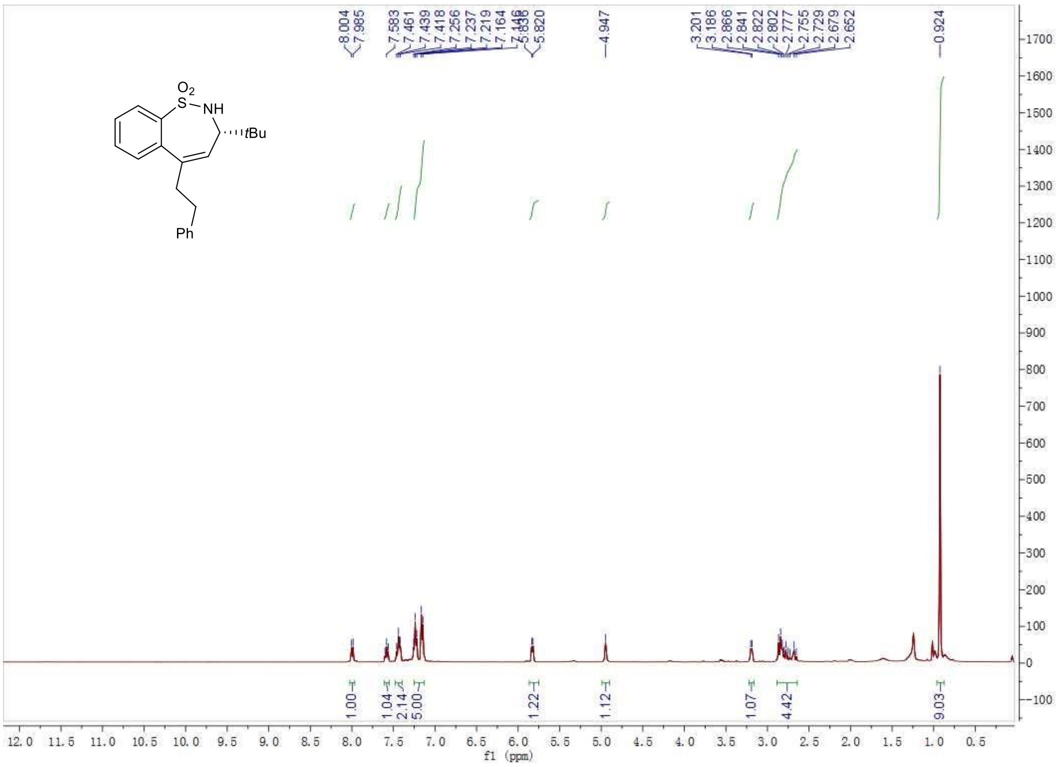

Supplementary Figure 188: ^{1}H NMR spectrum of compound **15** in $CDCl_3$

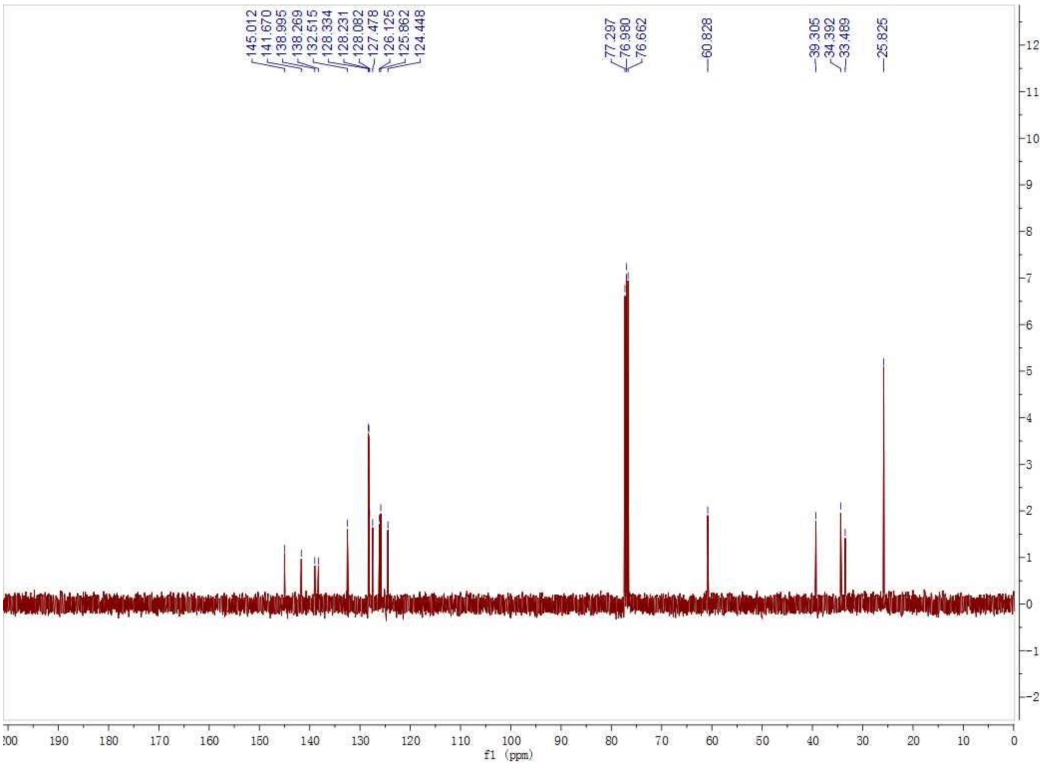

Supplementary Figure 189: ^{13}C NMR spectrum of compound **15** in $CDCl_3$

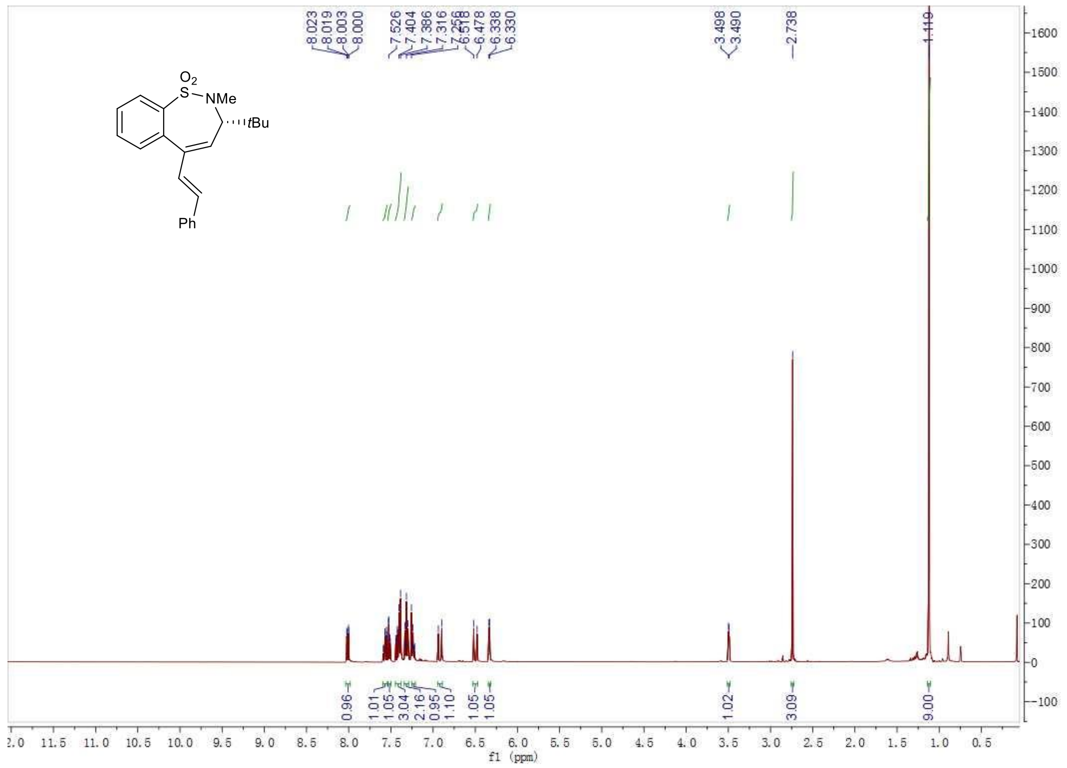

Supplementary Figure 190: ^{1}H NMR spectrum of compound **16** in $CDCl_3$

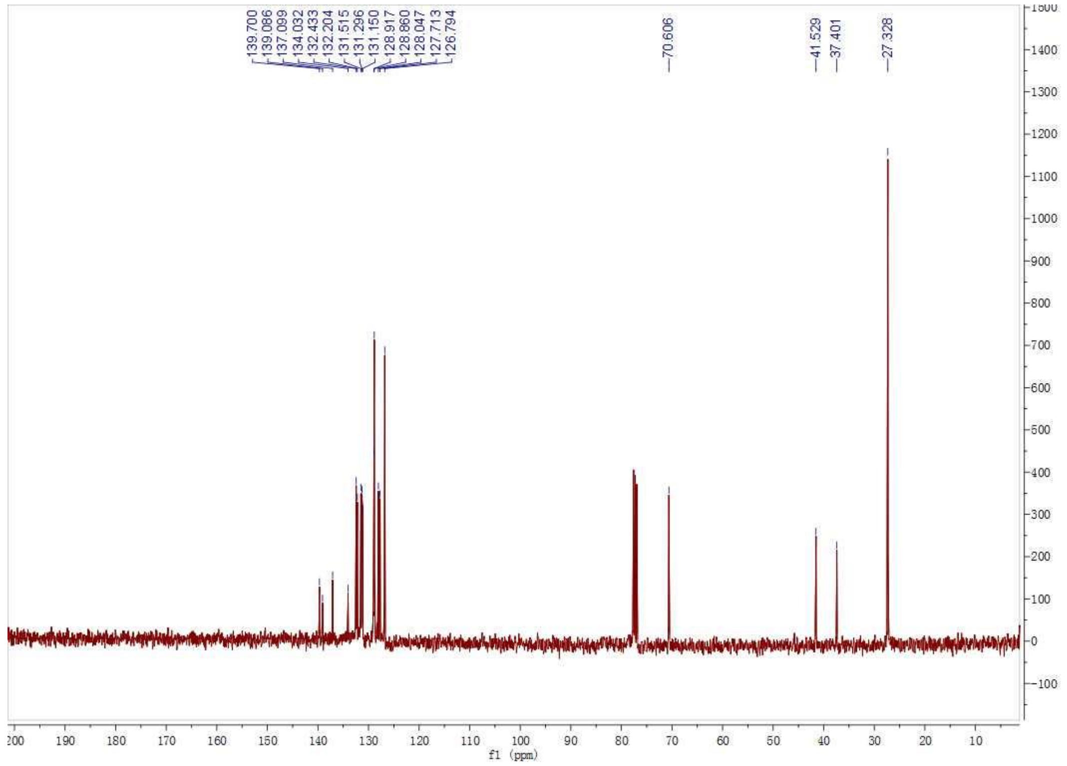

Supplementary Figure 191: ^{13}C NMR spectrum of compound **16** in $CDCl_3$

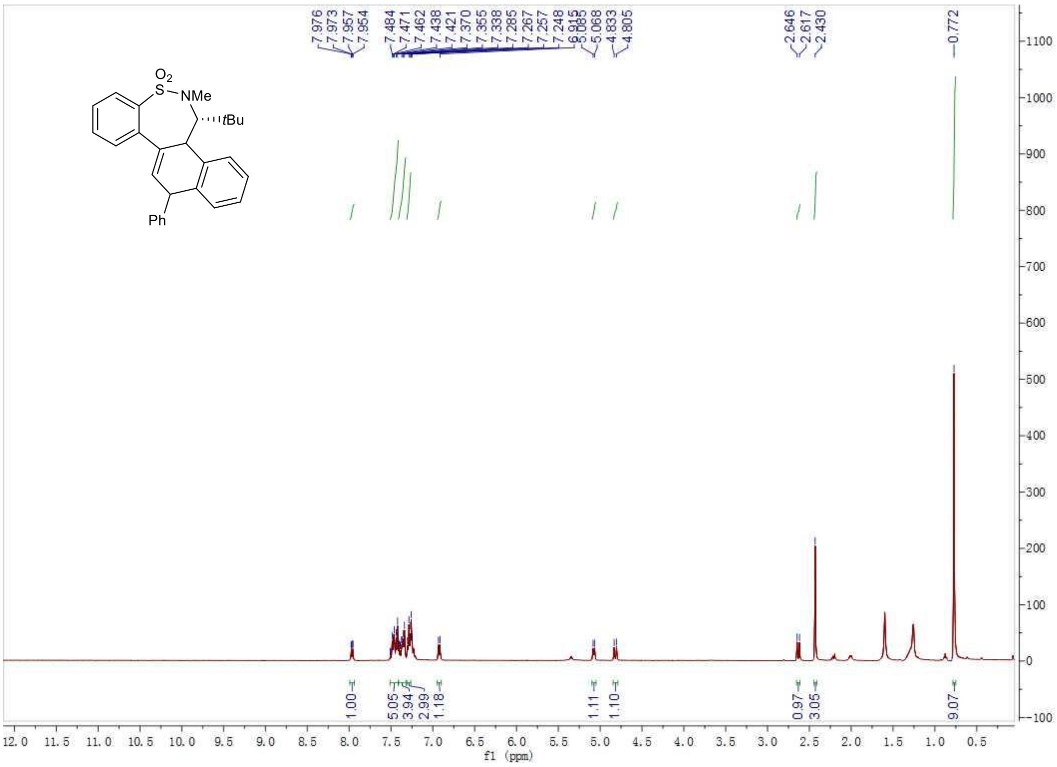

Supplementary Figure 192: ^{1}H NMR spectrum of compound **17** in $CDCl_3$

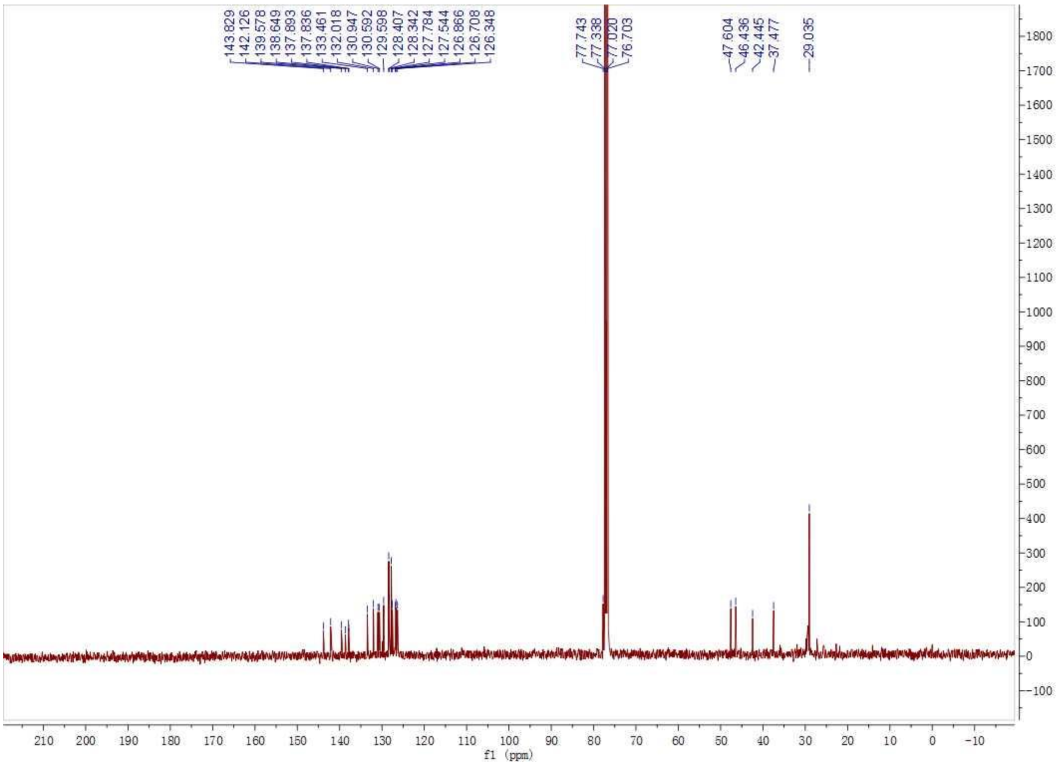

Supplementary Figure 193: ^{13}C NMR spectrum of compound **17** in $CDCl_3$

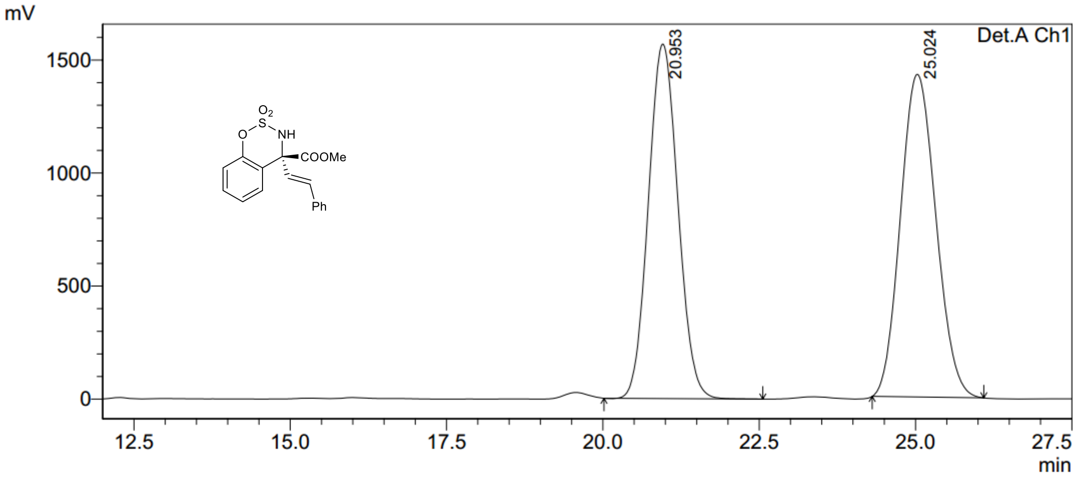

1 Det.A Ch1/210nm

PeakTable

Detector A Ch1 210nm

Peak#	Ret. Time	Area	Height	Area %	Height %
1	20.953	51542569	1568871	47.829	52.364
2	25.024	56221779	1427230	52.171	47.636
Total		107764348	2996101	100.000	100.000

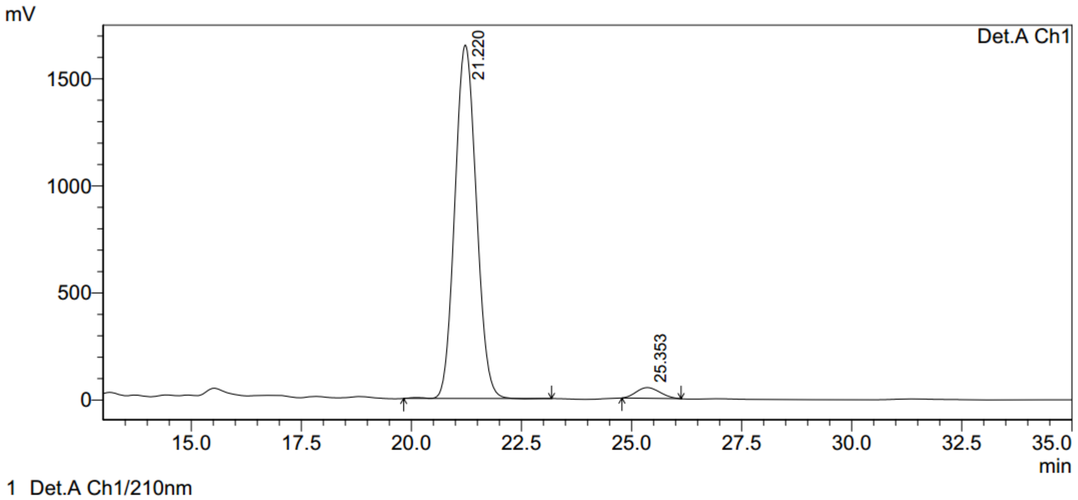

1 Det.A Ch1/210nm

PeakTable

Detector A Ch1 210nm

Peak#	Ret. Time	Area	Height	Area %	Height %
1	21.220	55940688	1650930	96.768	97.065
2	25.353	1868134	49925	3.232	2.935
Total		57808822	1700855	100.000	100.000

Supplementary Figure 194: HPLC traces for product **3aa**

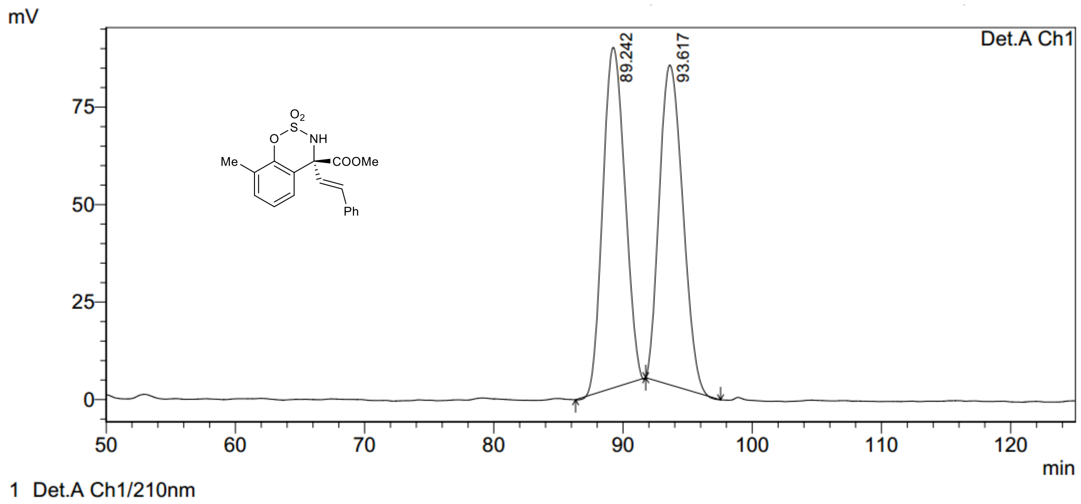

PeakTable

Detector A Ch1 210nm

Peak#	Ret. Time	Area	Height	Area %	Height %
1	89.242	10332848	87324	49.972	51.570
2	93.617	10344588	82008	50.028	48.430
Total		20677436	169331	100.000	100.000

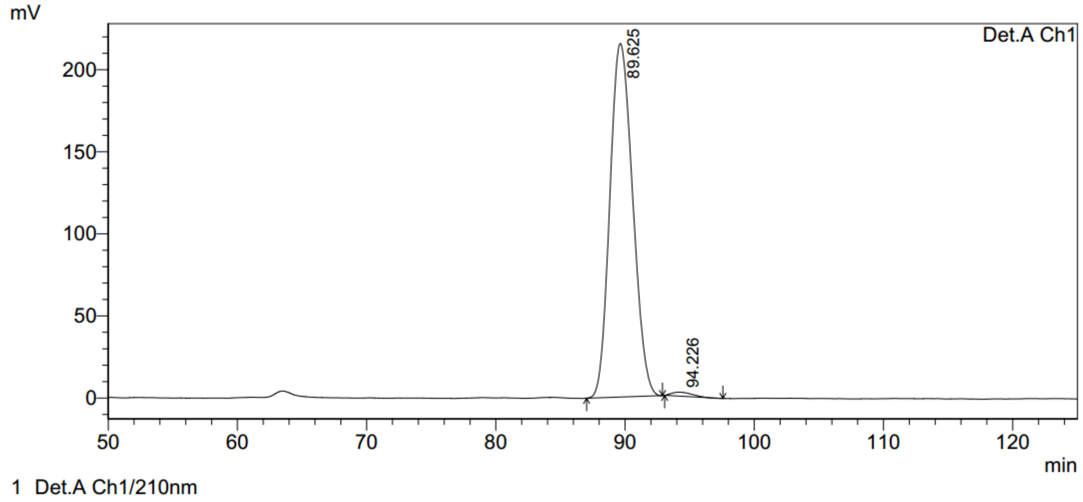

PeakTable

Detector A Ch1 210nm

Peak#	Ret. Time	Area	Height	Area %	Height %
1	89.625	26439766	215348	99.017	98.898
2	94.226	262476	2401	0.983	1.102
Total		26702242	217749	100.000	100.000

Supplementary Figure 195: HPLC traces for product **3ba**

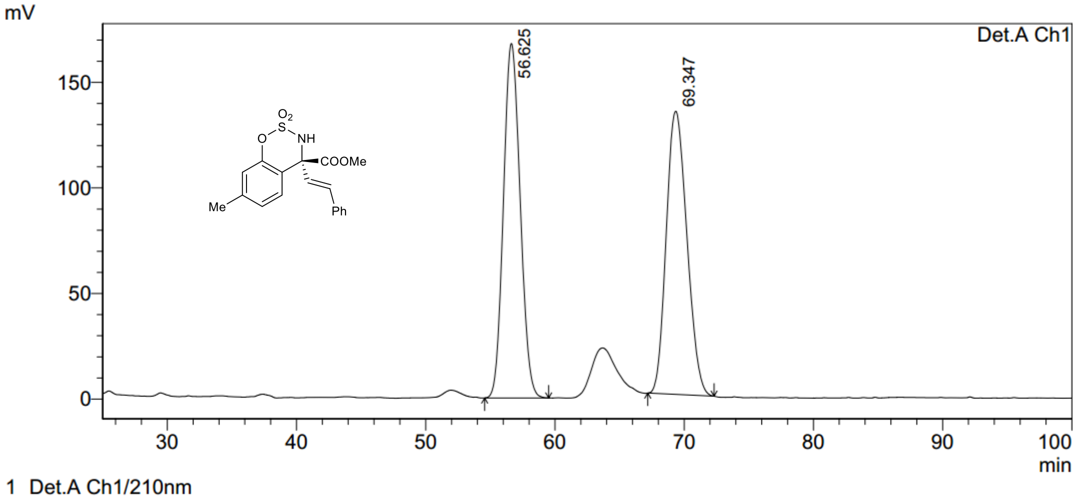

1 Det.A Ch1/210nm

PeakTable

Detector A Ch1 210nm

Peak#	Ret. Time	Area	Height	Area %	Height %
1	56.625	15083950	167897	50.283	55.596
2	69.347	14914420	134099	49.717	44.404
Total		29998370	301996	100.000	100.000

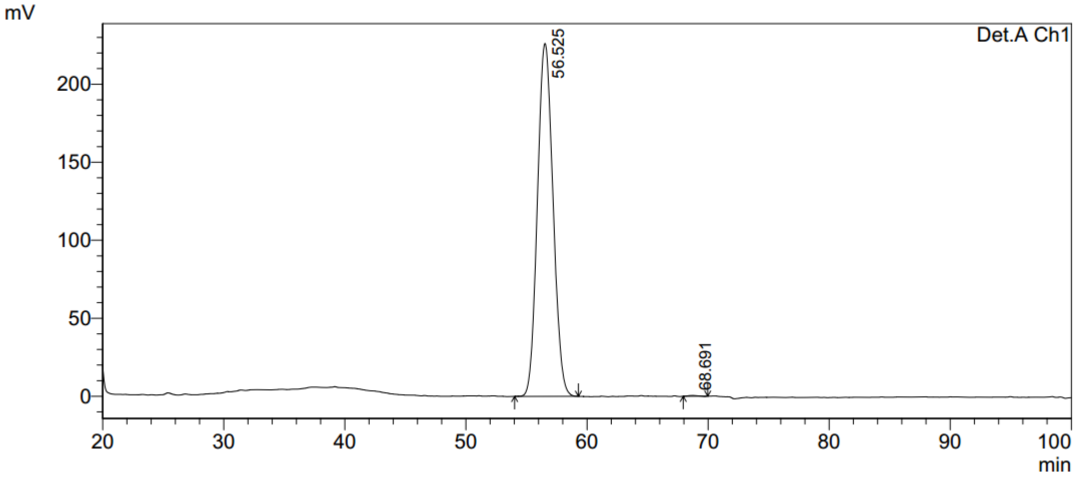

1 Det.A Ch1/210nm

PeakTable

Detector A Ch1 210nm

Peak#	Ret. Time	Area	Height	Area %	Height %
1	56.525	20189618	226195	99.902	99.748
2	68.691	19800	571	0.098	0.252
Total		20209418	226766	100.000	100.000

Supplementary Figure 196: HPLC traces for product **3ca**

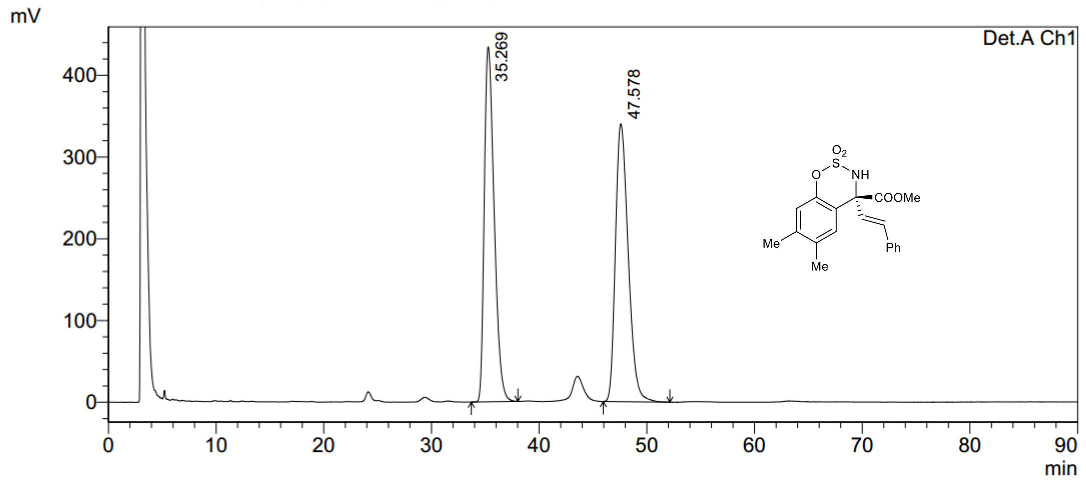

1 Det.A Ch1/210nm

PeakTable

Detector A Ch1 210nm

Peak#	Ret. Time	Area	Height	Area %	Height %
1	35.269	27639779	434405	49.642	56.102
2	47.578	28038353	339902	50.358	43.898
Total		55678131	774308	100.000	100.000

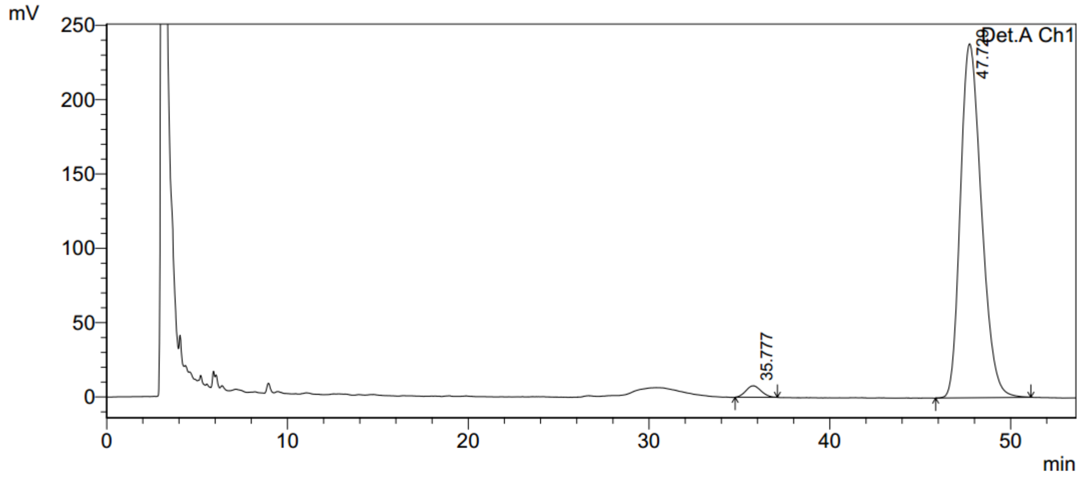

1 Det.A Ch1/210nm

PeakTable

Detector A Ch1 210nm

Peak#	Ret. Time	Area	Height	Area %	Height %
1	35.777	452082	7690	2.291	3.129
2	47.729	19284626	238114	97.709	96.871
Total		19736708	245804	100.000	100.000

Supplementary Figure 197: HPLC traces for product **3da**

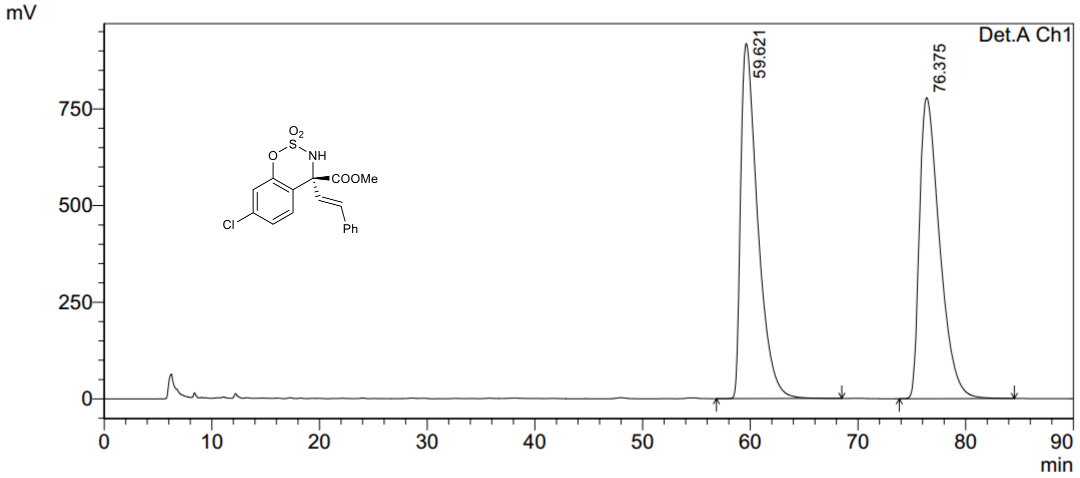

1 Det.A Ch1/210nm

PeakTable

Detector A Ch1 210nm

Peak#	Ret. Time	Area	Height	Area %	Height %
1	59.621	100930584	918698	49.887	54.125
2	76.375	101388799	778668	50.113	45.875
Total		202319383	1697366	100.000	100.000

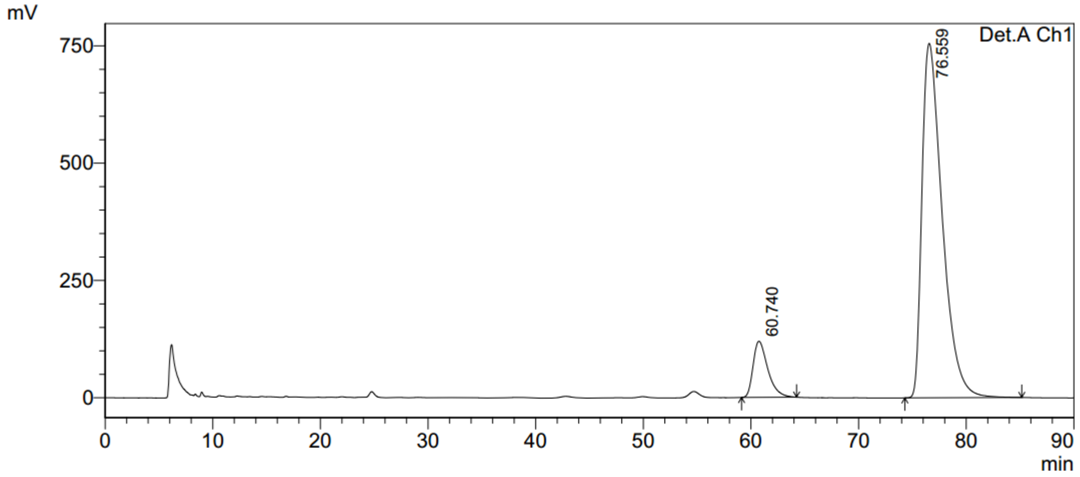

1 Det.A Ch1/210nm

PeakTable

Detector A Ch1 210nm

Peak#	Ret. Time	Area	Height	Area %	Height %
1	60.740	11378840	119666	10.317	13.674
2	76.559	98917273	755474	89.683	86.326
Total		110296113	875140	100.000	100.000

Supplementary Figure 198: HPLC traces for product **3ea**

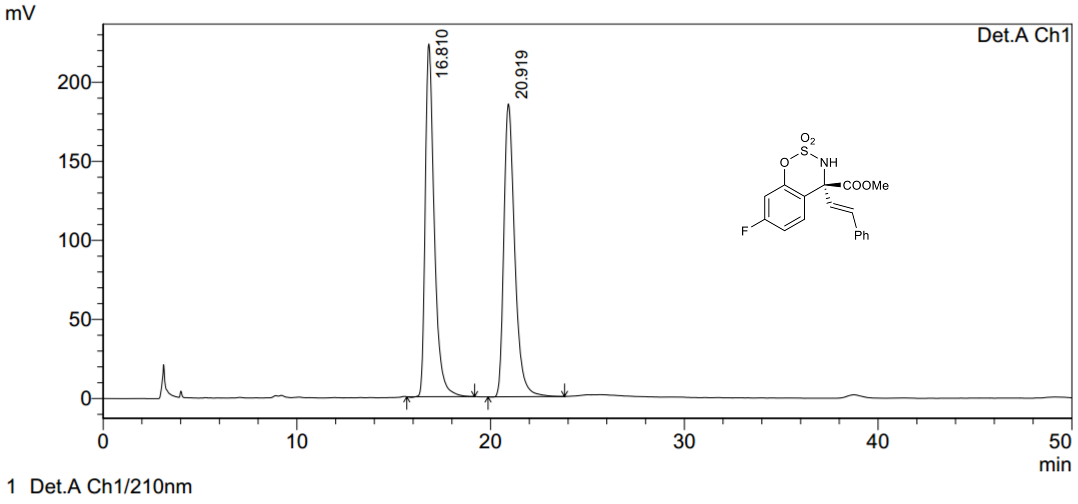

PeakTable

Detector A Ch1 210nm

Peak#	Ret. Time	Area	Height	Area %	Height %
1	16.810	7232824	223068	49.990	54.642
2	20.919	7235737	185164	50.010	45.358
Total		14468560	408232	100.000	100.000

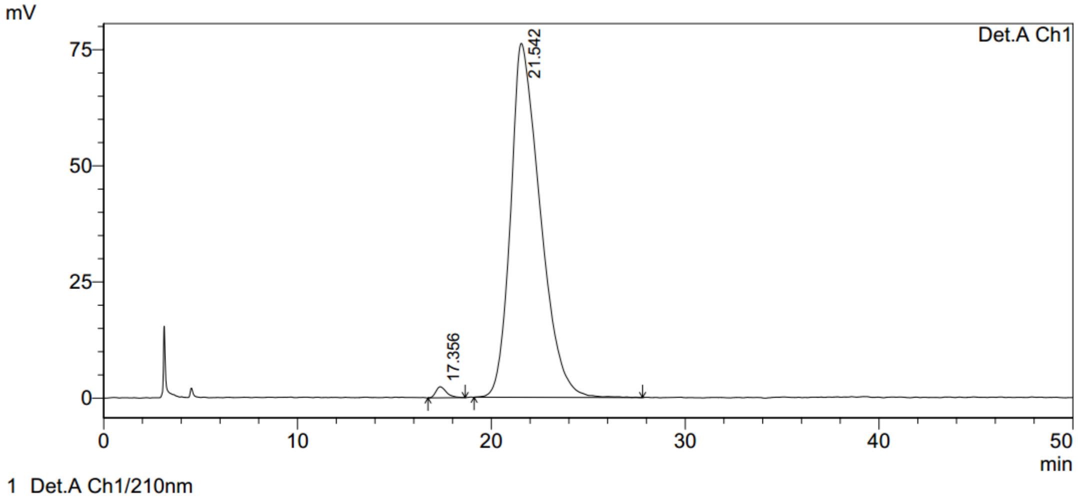

PeakTable

Detector A Ch1 210nm

Peak#	Ret. Time	Area	Height	Area %	Height %
1	17.356	90648	2374	1.106	3.021
2	21.542	8102515	76195	98.894	96.979
Total		8193163	78568	100.000	100.000

Supplementary Figure 199: HPLC traces for product **3fa**

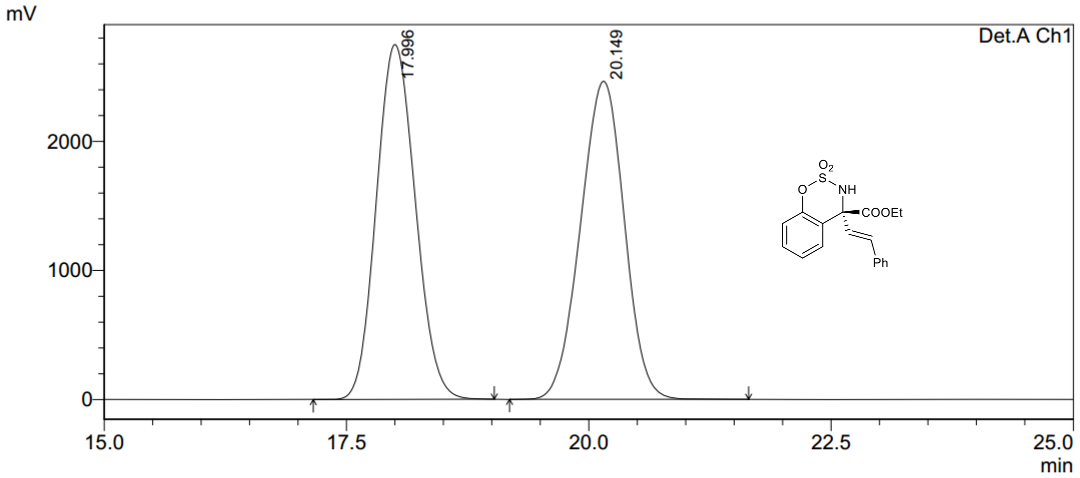

1 Det.A Ch1/210nm

PeakTable

Detector A Ch1 210nm

Peak#	Ret. Time	Area	Height	Area %	Height %
1	17.996	76917029	2749405	49.871	52.743
2	20.149	77315612	2463463	50.129	47.257
Total		154232640	5212868	100.000	100.000

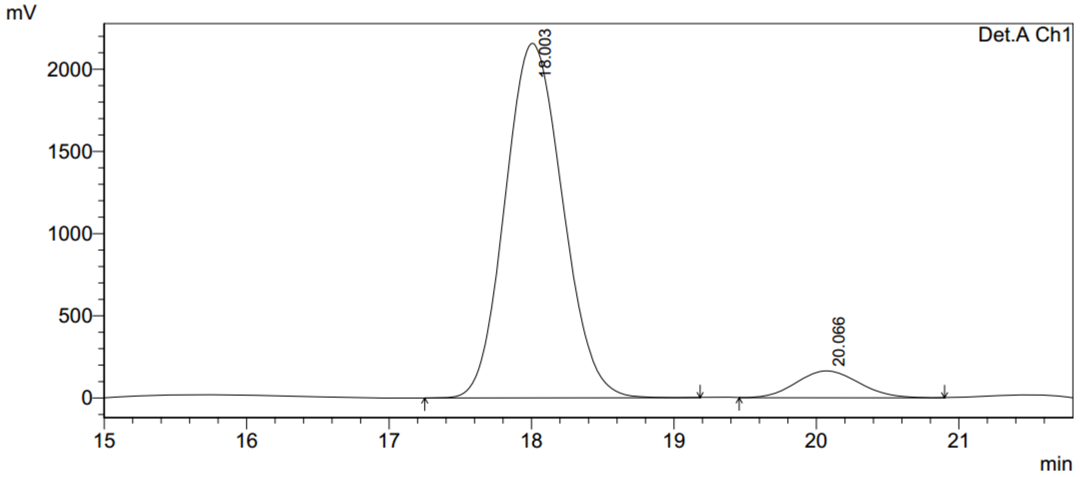

1 Det.A Ch1/210nm

PeakTable

Detector A Ch1 210nm

Peak#	Ret. Time	Area	Height	Area %	Height %
1	18.003	60468375	2156370	92.367	92.958
2	20.066	4996776	163349	7.633	7.042
Total		65465151	2319719	100.000	100.000

Supplementary Figure 200: HPLC traces for product **3ga**

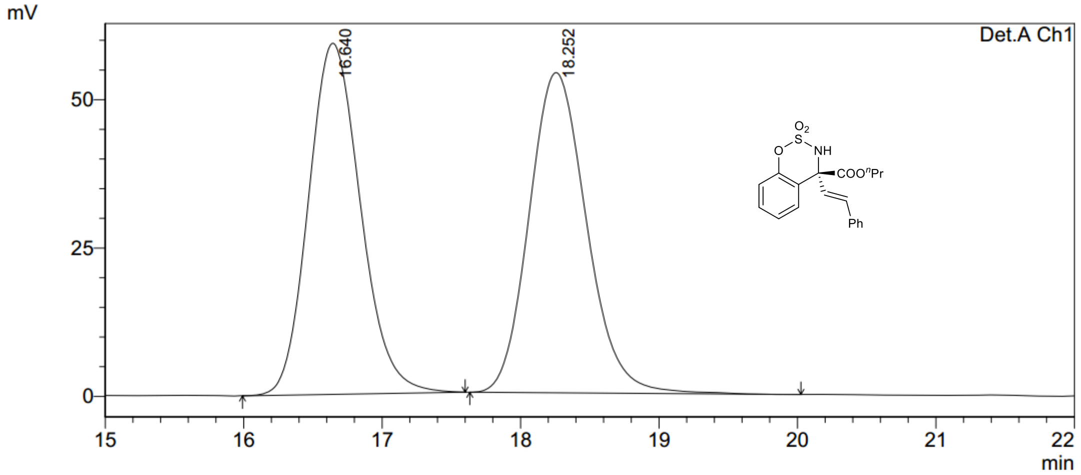

1 Det.A Ch1/210nm

PeakTable

Detector A Ch1 210nm

Peak#	Ret. Time	Area	Height	Area %	Height %
1	16.640	1546129	59161	49.811	52.296
2	18.252	1557870	53967	50.189	47.704
Total		3103999	113128	100.000	100.000

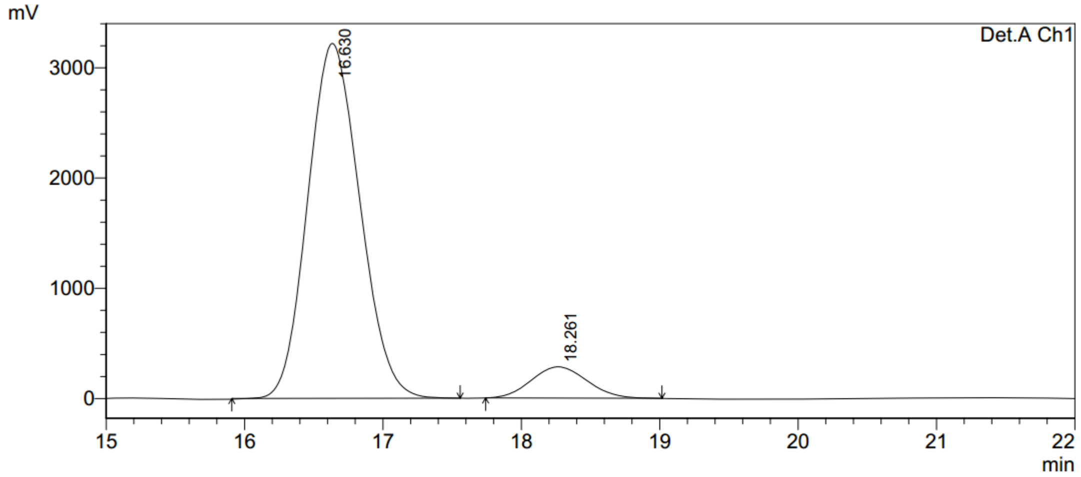

1 Det.A Ch1/210nm

PeakTable

Detector A Ch1 210nm

Peak#	Ret. Time	Area	Height	Area %	Height %
1	16.630	84281865	3218931	91.355	91.896
2	18.261	7975675	283874	8.645	8.104
Total		92257540	3502806	100.000	100.000

Supplementary Figure 201: HPLC traces for product **3ha**

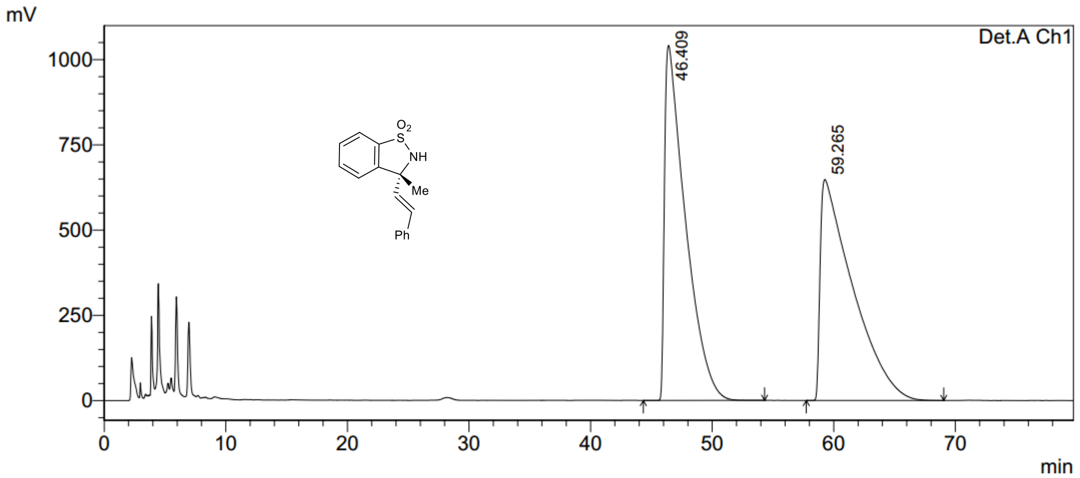

1 Det.A Ch1/210nm

PeakTable

Detector A Ch1 210nm

Peak#	Ret. Time	Area	Height	Area %	Height %
1	46.409	124179239	1041239	50.774	61.649
2	59.265	120392640	647744	49.226	38.351
Total		244571879	1688983	100.000	100.000

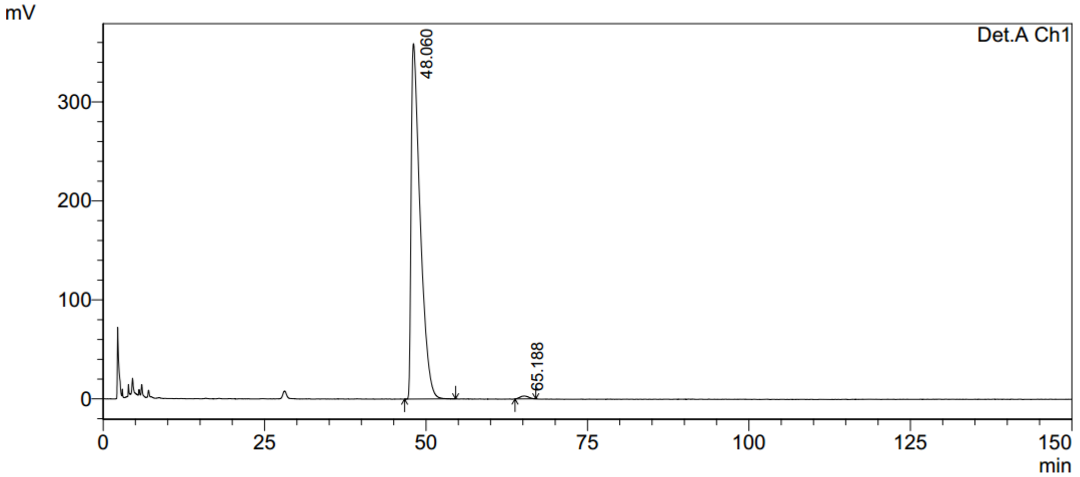

1 Det.A Ch1/210nm

PeakTable

Detector A Ch1 210nm

Peak#	Ret. Time	Area	Height	Area %	Height %
1	48.060	34940426	359208	99.228	99.195
2	65.188	272014	2916	0.772	0.805
Total		35212440	362124	100.000	100.000

Supplementary Figure 202: HPLC traces for product **3ia**

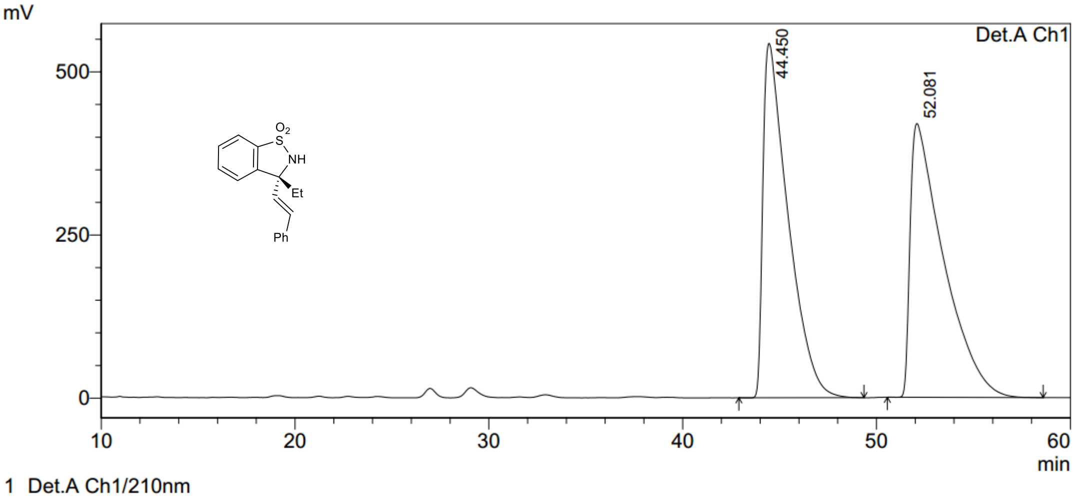

PeakTable

Detector A Ch1 210nm

Peak#	Ret. Time	Area	Height	Area %	Height %
1	44.450	48920104	543217	49.367	56.423
2	52.081	50175004	419536	50.633	43.577
Total		99095108	962752	100.000	100.000

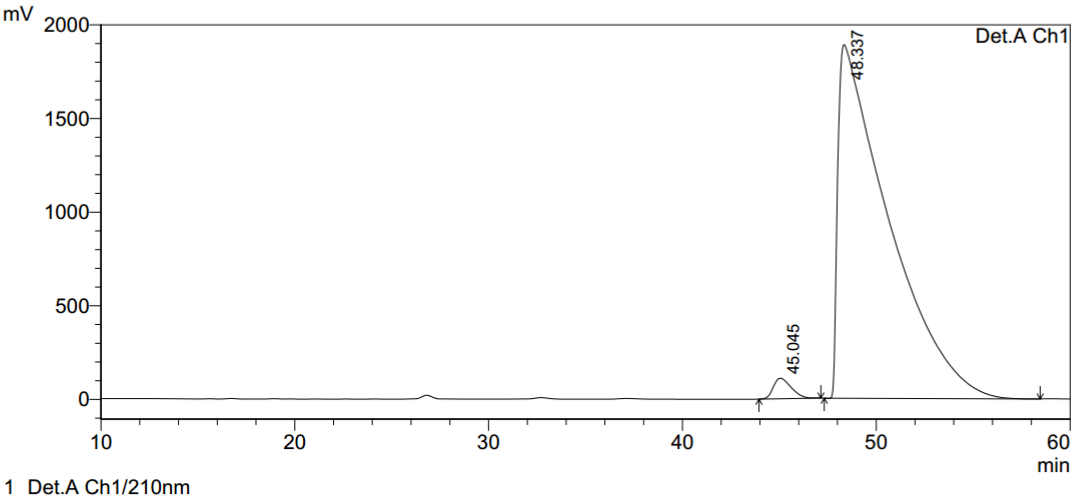

PeakTable

Detector A Ch1 210nm

Peak#	Ret. Time	Area	Height	Area %	Height %
1	45.045	6979041	109572	1.981	5.485
2	48.337	345262284	1888210	98.019	94.515
Total		352241325	1997782	100.000	100.000

Supplementary Figure 203: HPLC traces for product **3ja**

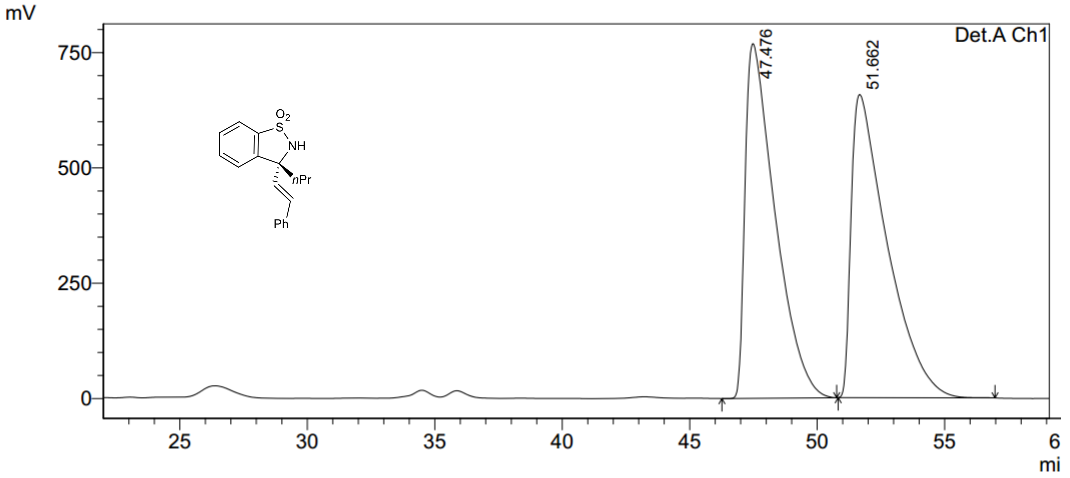

1 Det.A Ch1/210nm

PeakTable

Detector A Ch1 210nm

Peak#	Ret. Time	Area	Height	Area %	Height %
1	47.476	62866316	768897	49.340	53.915
2	51.662	64548232	657235	50.660	46.085
Total		127414548	1426132	100.000	100.000

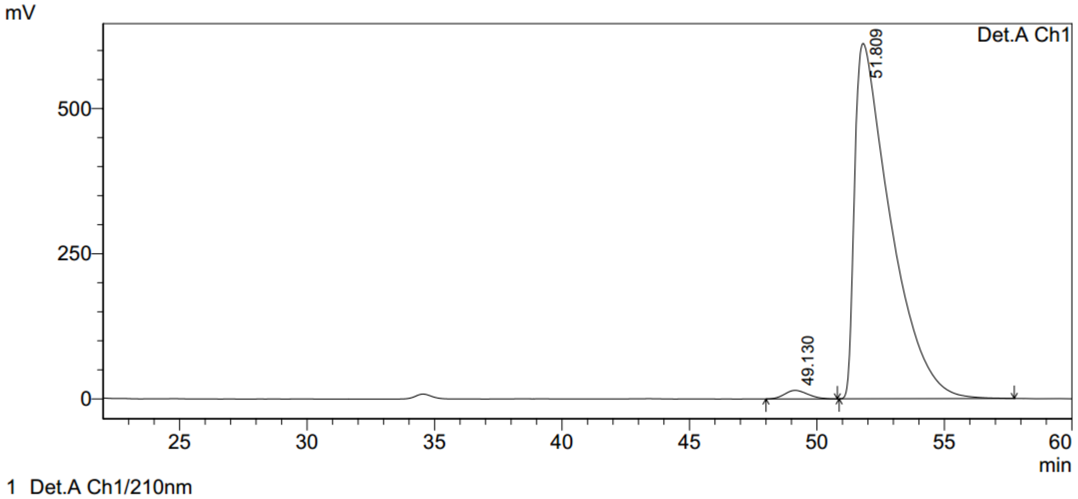

1 Det.A Ch1/210nm

PeakTable

Detector A Ch1 210nm

Peak#	Ret. Time	Area	Height	Area %	Height %
1	49.130	928209	14666	1.522	2.340
2	51.809	60071220	612041	98.478	97.660
Total		60999429	626707	100.000	100.000

Supplementary Figure 204: HPLC traces for product **3ka**

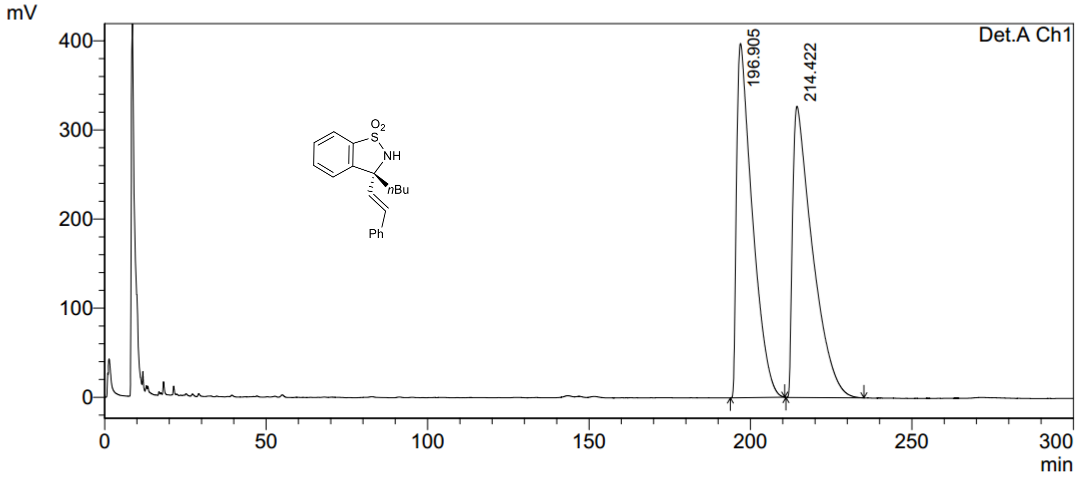

1 Det.A Ch1/210nm

PeakTable

Detector A Ch1 210nm

Peak#	Ret. Time	Area	Height	Area %	Height %
1	196.905	135324879	397602	49.107	54.890
2	214.422	140247980	326753	50.893	45.110
Total		275572858	724355	100.000	100.000

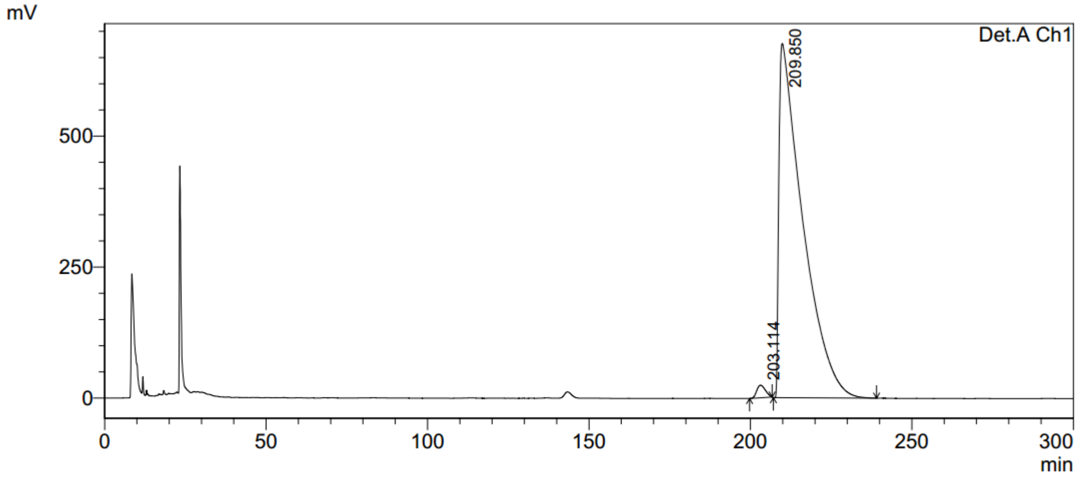

1 Det.A Ch1/210nm

PeakTable

Detector A Ch1 210nm

Peak#	Ret. Time	Area	Height	Area %	Height %
1	203.114	4473450	24085	1.279	3.439
2	209.850	345170276	676181	98.721	96.561
Total		349643726	700266	100.000	100.000

Supplementary Figure 205: HPLC traces for product **3la**

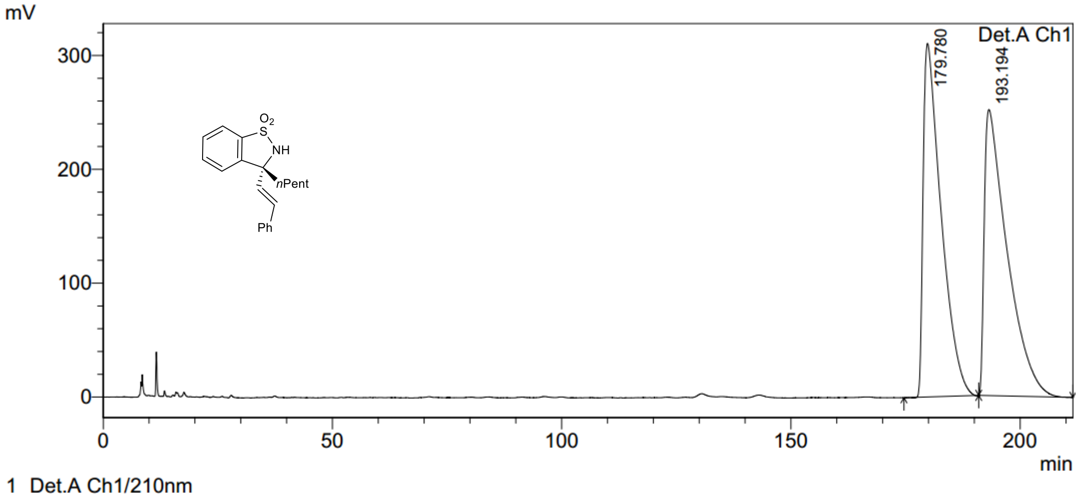

1 Det.A Ch1/210nm

PeakTable

Detector A Ch1 210nm

Peak#	Ret. Time	Area	Height	Area %	Height %
1	179.780	84566964	310551	49.226	55.281
2	193.194	87226967	251220	50.774	44.719
Total		171793931	561770	100.000	100.000

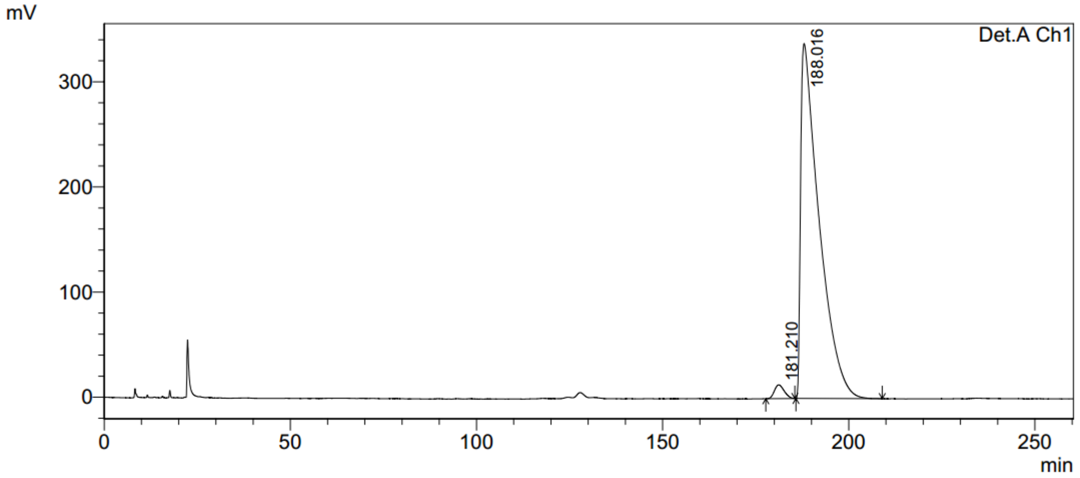

1 Det.A Ch1/210nm

PeakTable

Detector A Ch1 210nm

Peak#	Ret. Time	Area	Height	Area %	Height %
1	181.210	2395084	13087	2.016	3.732
2	188.016	116414662	337597	97.984	96.268
Total		118809746	350684	100.000	100.000

Supplementary Figure 206: HPLC traces for product **3ma**

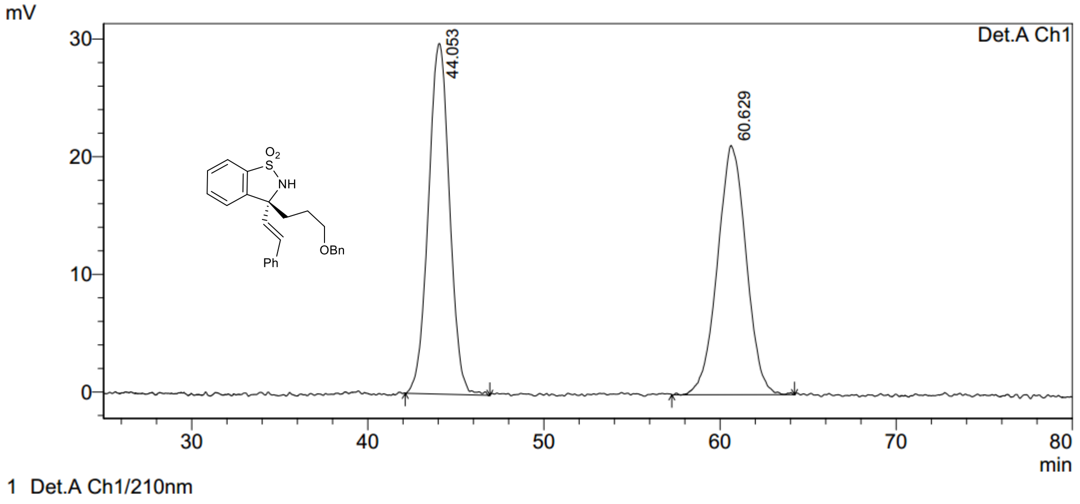

PeakTable

Detector A Ch1 210nm

Peak#	Ret. Time	Area	Height	Area %	Height %
1	44.053	2485856	29812	50.836	58.461
2	60.629	2404108	21183	49.164	41.539
Total		4889964	50994	100.000	100.000

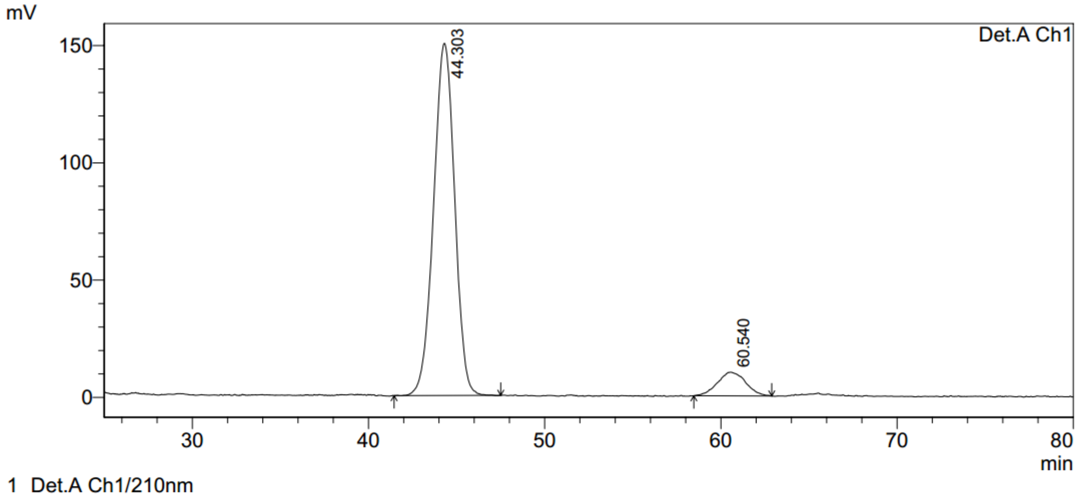

PeakTable

Detector A Ch1 210nm

Peak#	Ret. Time	Area	Height	Area %	Height %
1	44.303	12460623	150136	91.713	93.712
2	60.540	1125939	10073	8.287	6.288
Total		13586562	160210	100.000	100.000

Supplementary Figure 207: HPLC traces for product **3na**

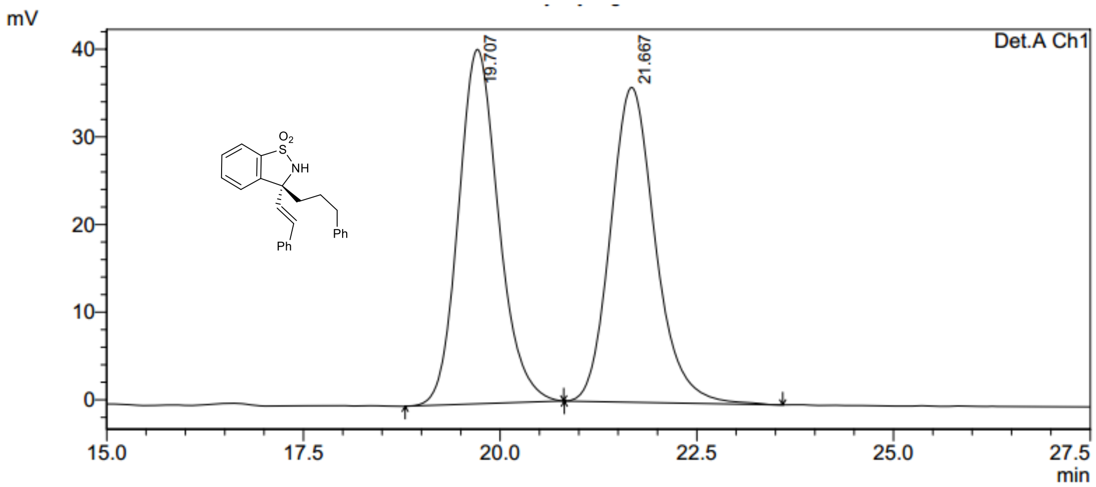

1 Det.A Ch1/210nm

PeakTable

检测器 A Ch1 210nm

Peak#	Ret. Time	Area	Height	Area %	Height %
1	19.707	1433507	40437	50.181	52.953
2	21.667	1423171	35926	49.819	47.047
Total		2856678	76363	100.000	100.000

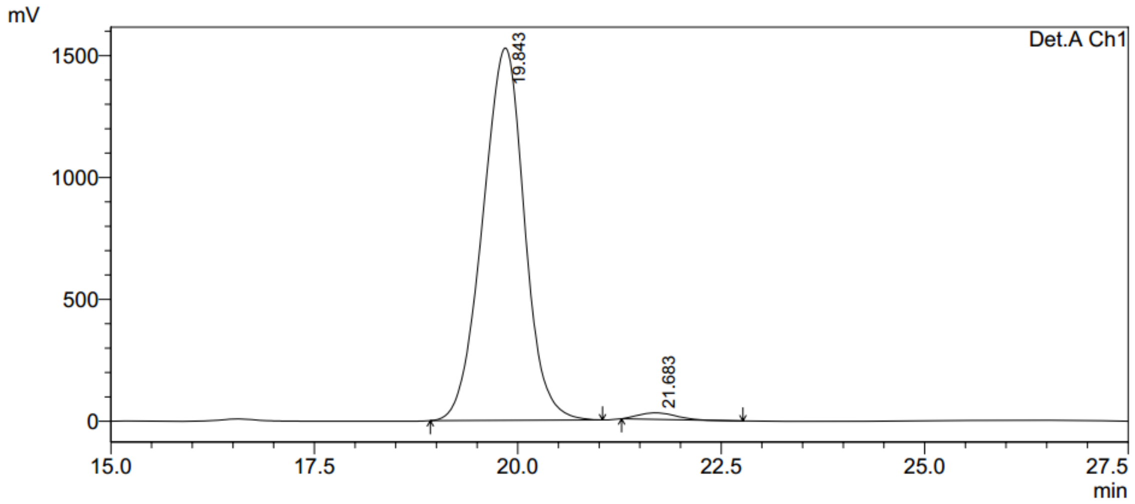

1 Det.A Ch1/210nm

PeakTable

检测器 A Ch1 210nm

Peak#	Ret. Time	Area	Height	Area %	Height %
1	19.843	54485053	1527635	98.462	98.266
2	21.683	851030	26963	1.538	1.734
Total		55336083	1554598	100.000	100.000

Supplementary Figure 208: HPLC traces for product **3oa**

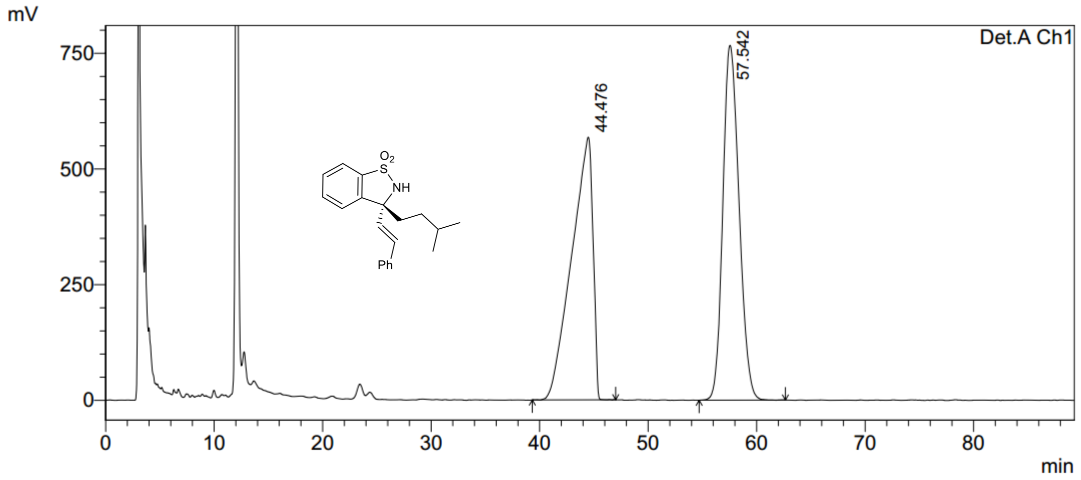

1 Det.A Ch1/210nm

PeakTable

Detector A Ch1 210nm

Peak#	Ret. Time	Area	Height	Area %	Height %
1	44.476	77340504	567765	49.243	42.534
2	57.542	79718215	767078	50.757	57.466
Total		157058719	1334842	100.000	100.000

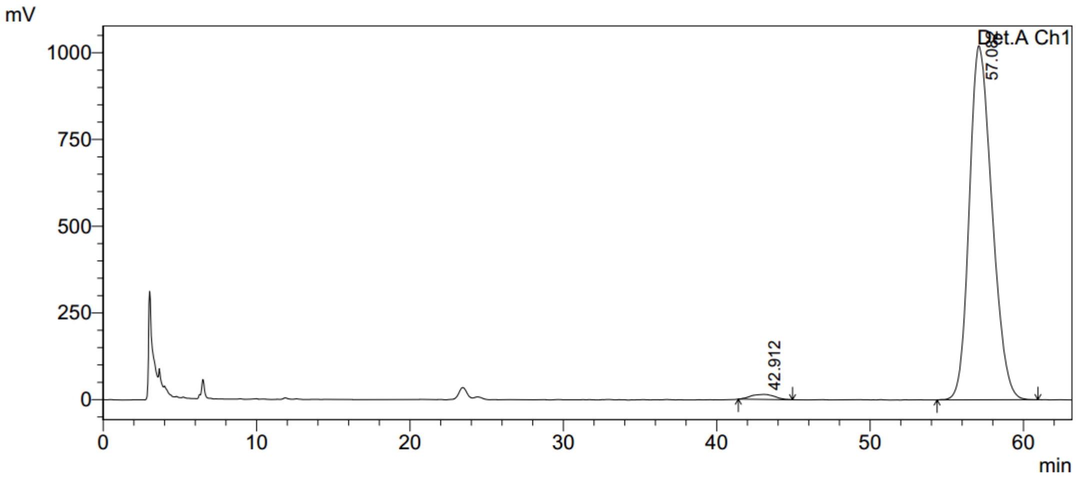

1 Det.A Ch1/210nm

PeakTable

Detector A Ch1 210nm

Peak#	Ret. Time	Area	Height	Area %	Height %
1	42.912	1544718	14127	1.449	1.365
2	57.082	105079009	1020842	98.551	98.635
Total		106623726	1034969	100.000	100.000

Supplementary Figure 209: HPLC traces for product **3pa**

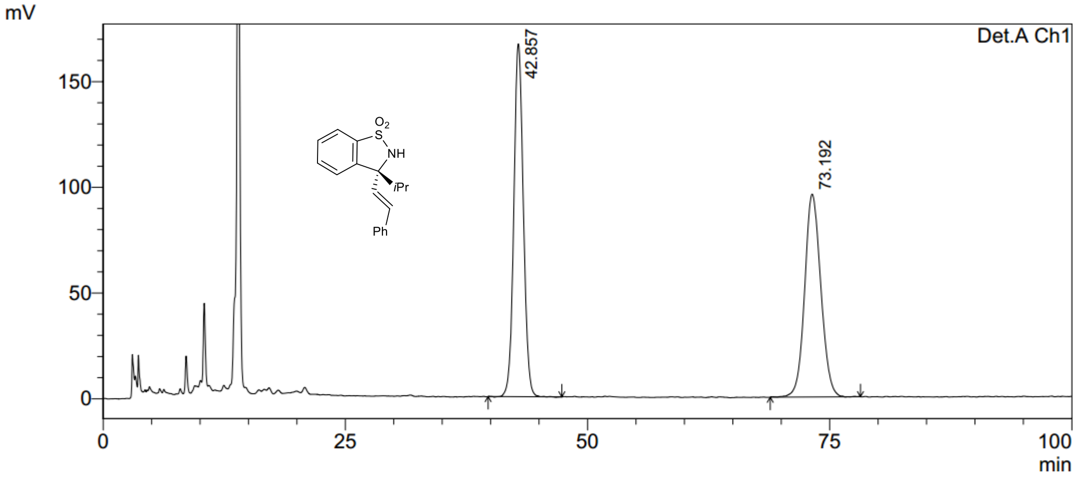

1 Det.A Ch1/210nm

PeakTable

Detector A Ch1 210nm

Peak#	Ret. Time	Area	Height	Area %	Height %
1	42.857	11375782	166893	50.534	63.497
2	73.192	11135270	95944	49.466	36.503
Total		22511052	262837	100.000	100.000

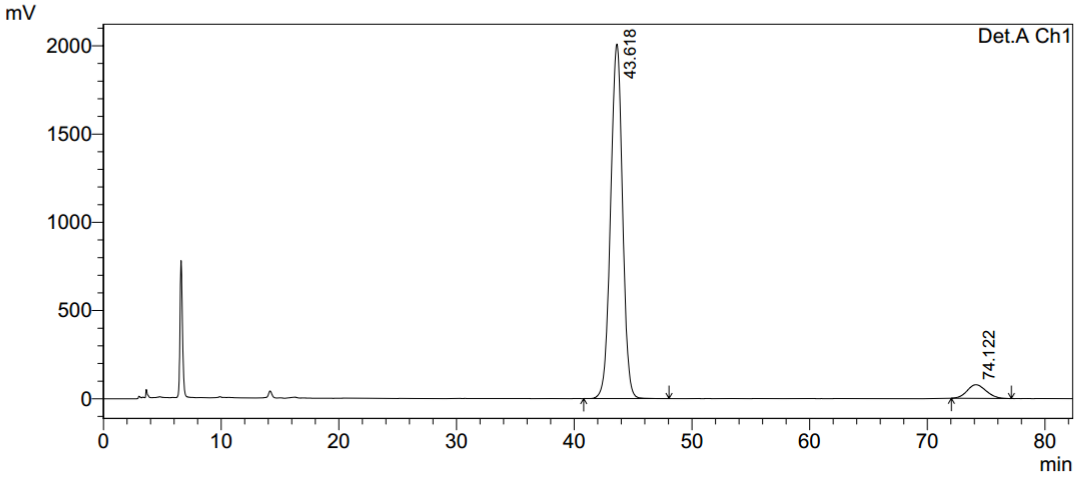

1 Det.A Ch1/210nm

PeakTable

Detector A Ch1 210nm

Peak#	Ret. Time	Area	Height	Area %	Height %
1	43.618	140947243	2009229	94.176	96.301
2	74.122	8717025	77174	5.824	3.699
Total		149664269	2086403	100.000	100.000

Supplementary Figure 210: HPLC traces for product **3qa**

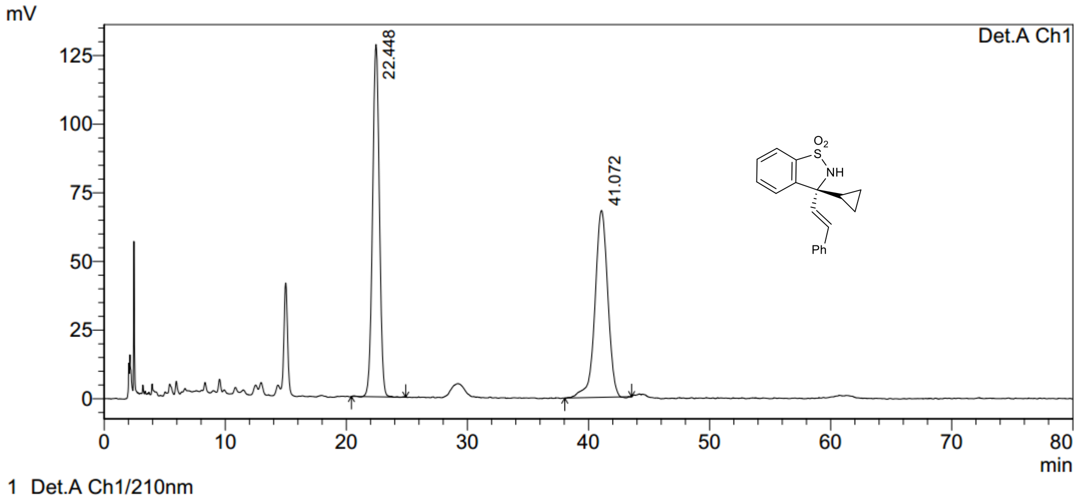

PeakTable

Detector A Ch1 210nm

Peak#	Ret. Time	Area	Height	Area %	Height %
1	22.448	5003547	128343	50.091	65.350
2	41.072	4985383	68049	49.909	34.650
Total		9988930	196392	100.000	100.000

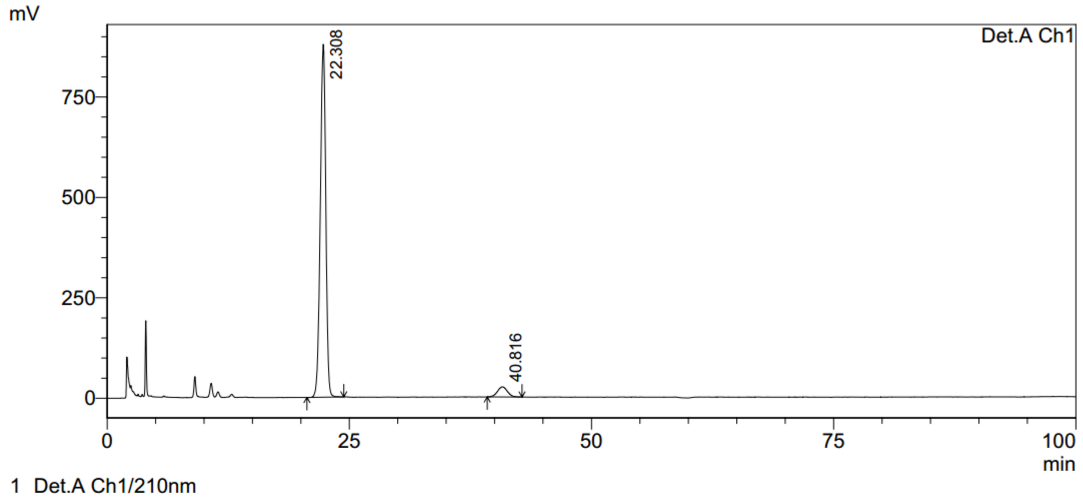

PeakTable

Detector A Ch1 210nm

Peak#	Ret. Time	Area	Height	Area %	Height %
1	22.308	34634644	878563	95.030	97.222
2	40.816	1811213	25107	4.970	2.778
Total		36445857	903670	100.000	100.000

Supplementary Figure 211: HPLC traces for product **3ra**

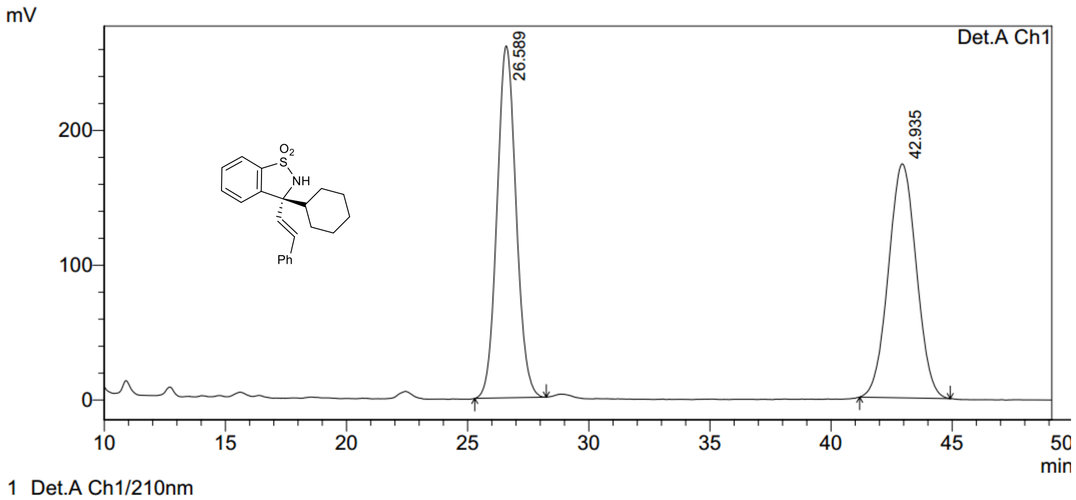

1 Det.A Ch1/210nm

PeakTable

Detector A Ch1 210nm

Peak#	Ret. Time	Area	Height	Area %	Height %
1	26.589	14283881	261142	50.638	60.065
2	42.935	13923722	173624	49.362	39.935
Total		28207603	434767	100.000	100.000

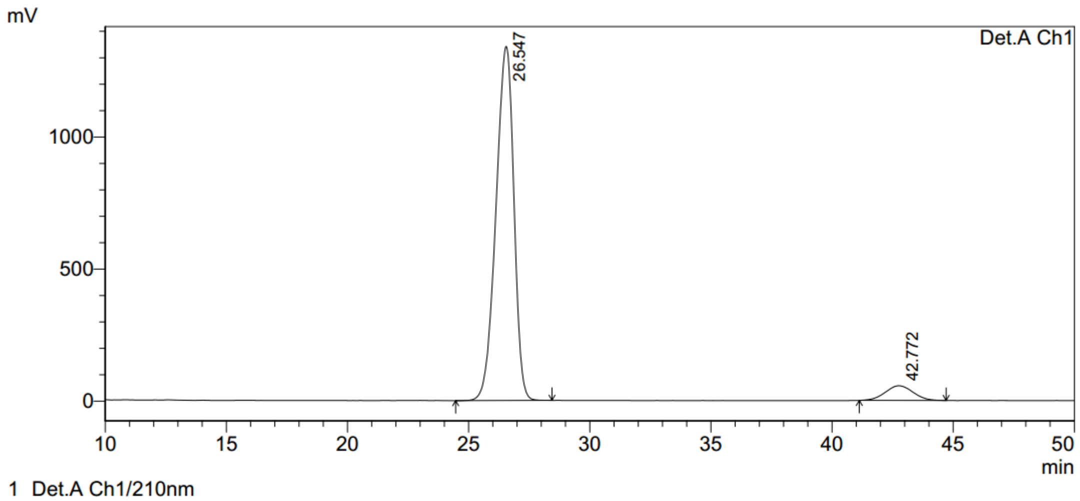

1 Det.A Ch1/210nm

PeakTable

Detector A Ch1 210nm

Peak#	Ret. Time	Area	Height	Area %	Height %
1	26.547	68927526	1340550	93.966	96.023
2	42.772	4426258	55525	6.034	3.977
Total		73353784	1396075	100.000	100.000

Supplementary Figure 212: HPLC traces for product **3sa**

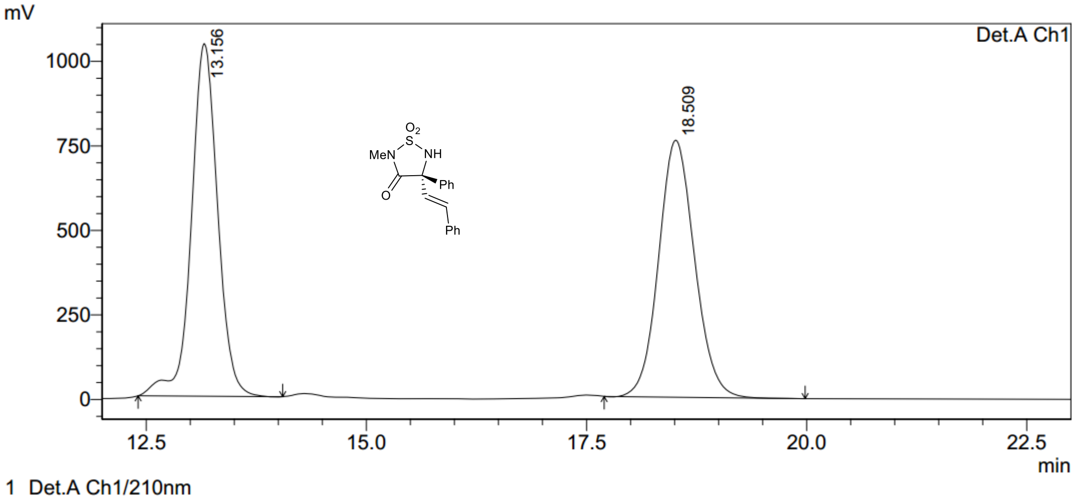

PeakTable

Detector A Ch1 210nm

Peak#	Ret. Time	Area	Height	Area %	Height %
1	13.156	21707951	1043429	50.088	57.853
2	18.509	21632069	760148	49.912	42.147
Total		43340020	1803577	100.000	100.000

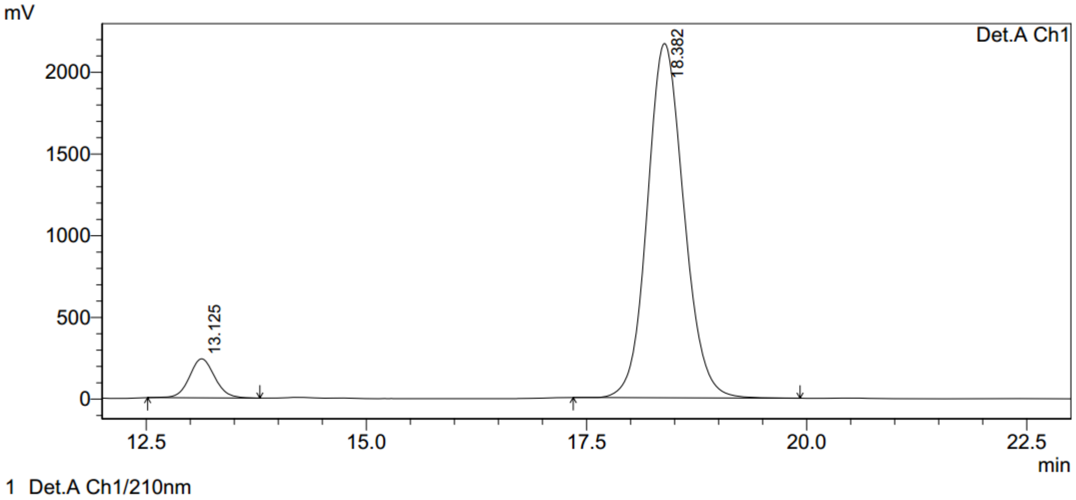

PeakTable

Detector A Ch1 210nm

Peak#	Ret. Time	Area	Height	Area %	Height %
1	13.125	4790447	239038	7.104	9.930
2	18.382	62639716	2168179	92.896	90.070
Total		67430163	2407217	100.000	100.000

Supplementary Figure 213: HPLC traces for product **3ta**

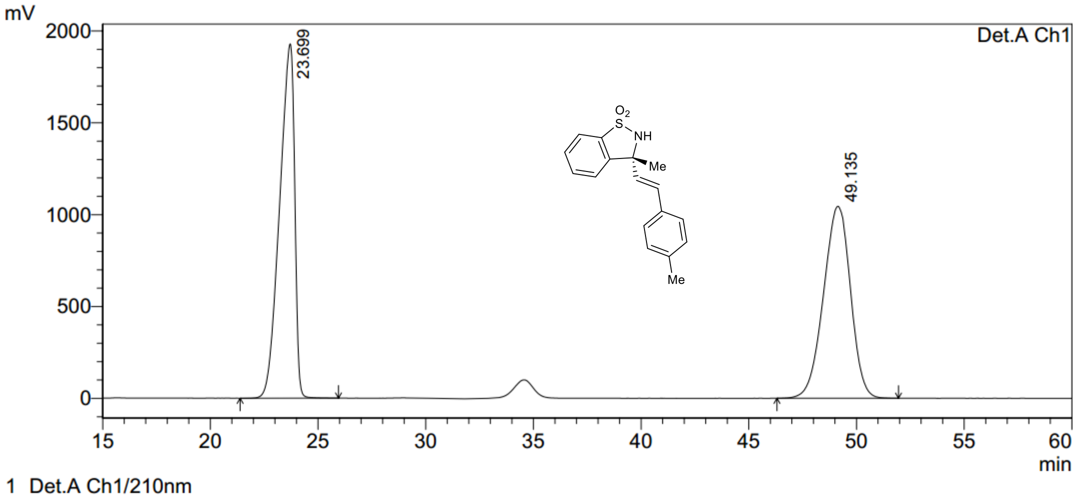

1 Det.A Ch1/210nm

PeakTable

Detector A Ch1 210nm

Peak#	Ret. Time	Area	Height	Area %	Height %
1	23.699	93512633	1928544	50.626	64.865
2	49.135	91198267	1044615	49.374	35.135
Total		184710901	2973159	100.000	100.000

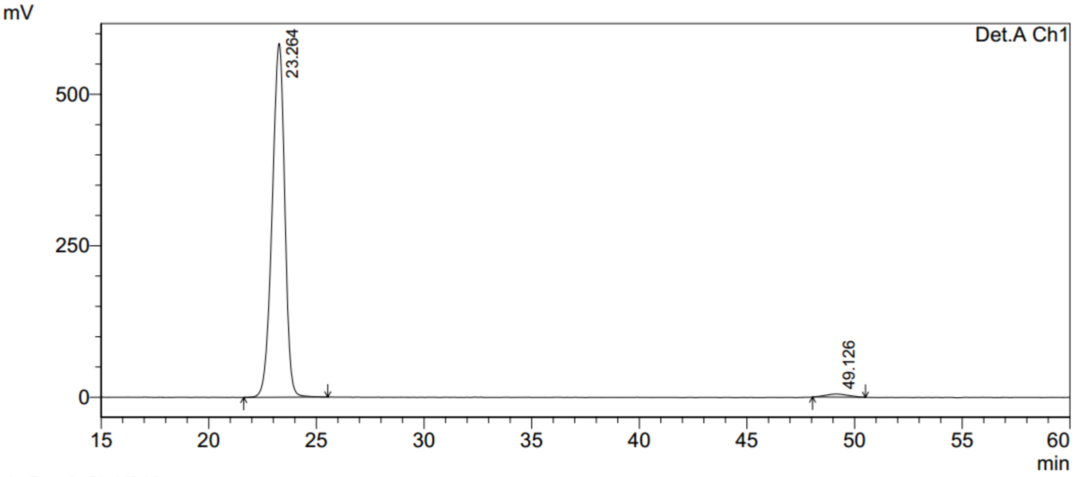

1 Det.A Ch1/210nm

PeakTable

Detector A Ch1 210nm

Peak#	Ret. Time	Area	Height	Area %	Height %
1	23.264	23744441	584271	98.428	99.141
2	49.126	379175	5065	1.572	0.859
Total		24123615	589335	100.000	100.000

Supplementary Figure 214: HPLC traces for product **3ib**

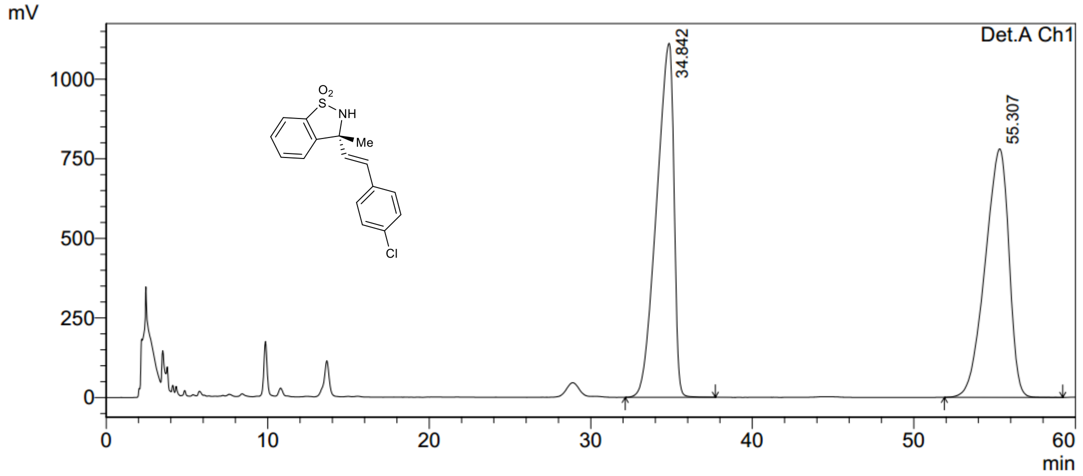

1 Det.A Ch1/210nm

PeakTable

Detector A Ch1 210nm

Peak#	Ret. Time	Area	Height	Area %	Height %
1	34.842	82761594	1111748	50.867	58.750
2	55.307	79940002	780604	49.133	41.250
Total		162701596	1892352	100.000	100.000

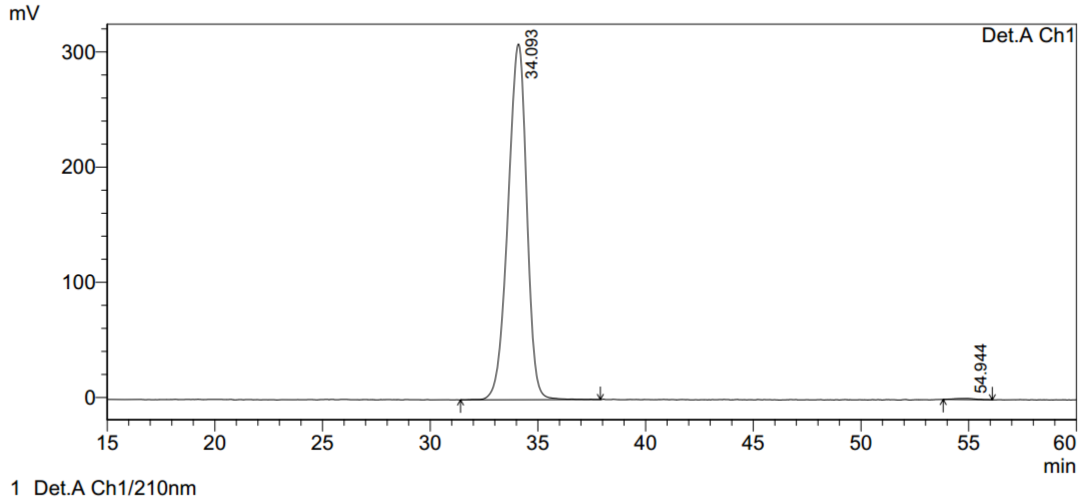

1 Det.A Ch1/210nm

PeakTable

Detector A Ch1 210nm

Peak#	Ret. Time	Area	Height	Area %	Height %
1	34.093	18693790	308848	99.615	99.652
2	54.944	72183	1079	0.385	0.348
Total		18765973	309927	100.000	100.000

Supplementary Figure 215: HPLC traces for product **3ic**

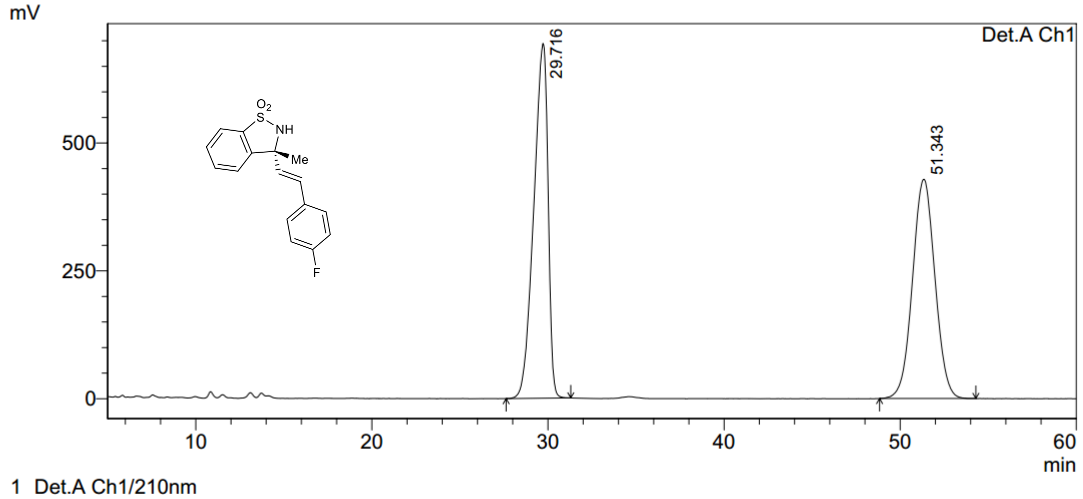

1 Det.A Ch1/210nm

PeakTable

Detector A Ch1 210nm

Peak#	Ret. Time	Area	Height	Area %	Height %
1	29.716	38985569	693794	50.681	61.804
2	51.343	37937426	428779	49.319	38.196
Total		76922995	1122573	100.000	100.000

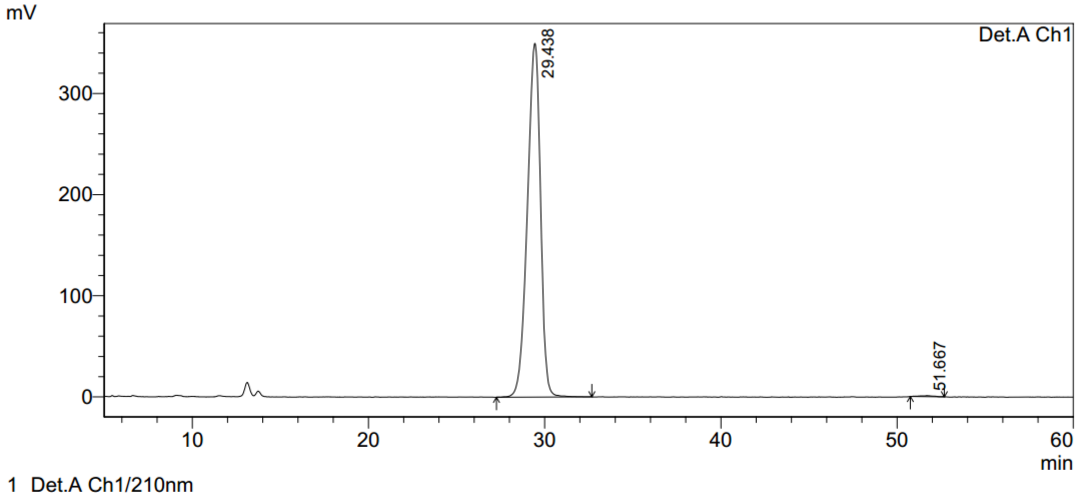

1 Det.A Ch1/210nm

PeakTable

Detector A Ch1 210nm

Peak#	Ret. Time	Area	Height	Area %	Height %
1	29.438	18103795	349794	99.698	99.699
2	51.667	54786	1056	0.302	0.301
Total		18158580	350849	100.000	100.000

Supplementary Figure 216: HPLC traces for product **3id**

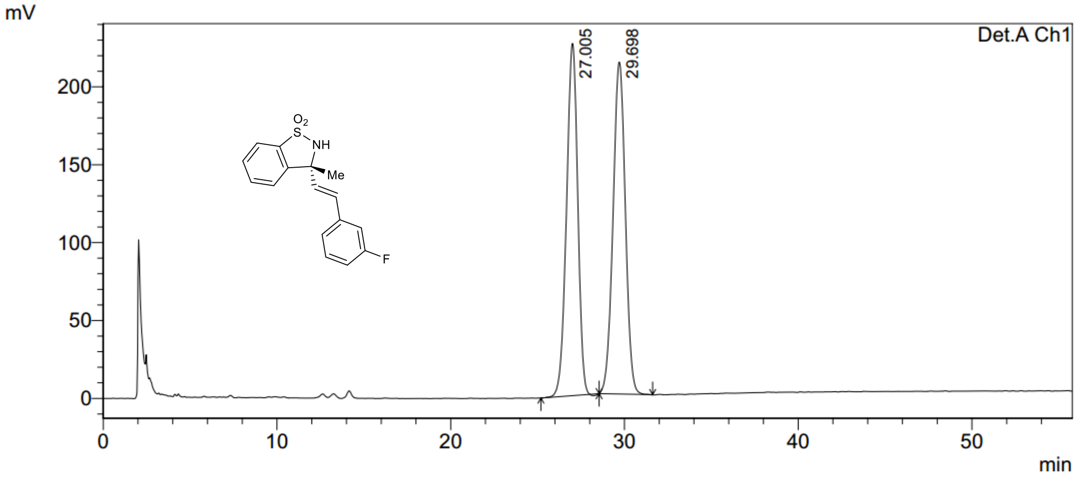

1 Det.A Ch1/210nm

PeakTable

Detector A Ch1 210nm

Peak#	Ret. Time	Area	Height	Area %	Height %
1	27.005	10109392	226065	49.114	51.479
2	29.698	10474319	213074	50.886	48.521
Total		20583711	439139	100.000	100.000

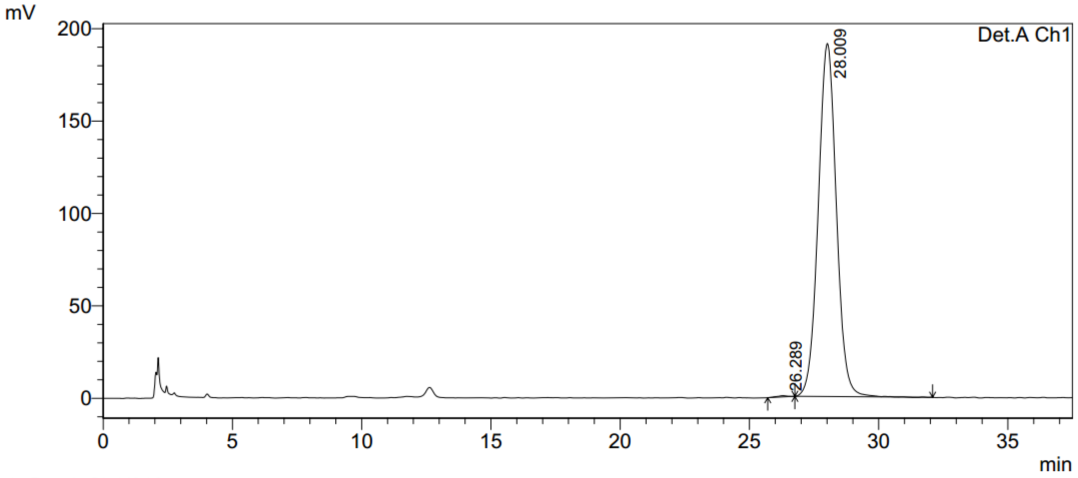

1 Det.A Ch1/210nm

PeakTable

Detector A Ch1 210nm

Peak#	Ret. Time	Area	Height	Area %	Height %
1	26.289	17212	645	0.185	0.336
2	28.009	9295836	191103	99.815	99.664
Total		9313048	191747	100.000	100.000

Supplementary Figure 217: HPLC traces for product **3ie**

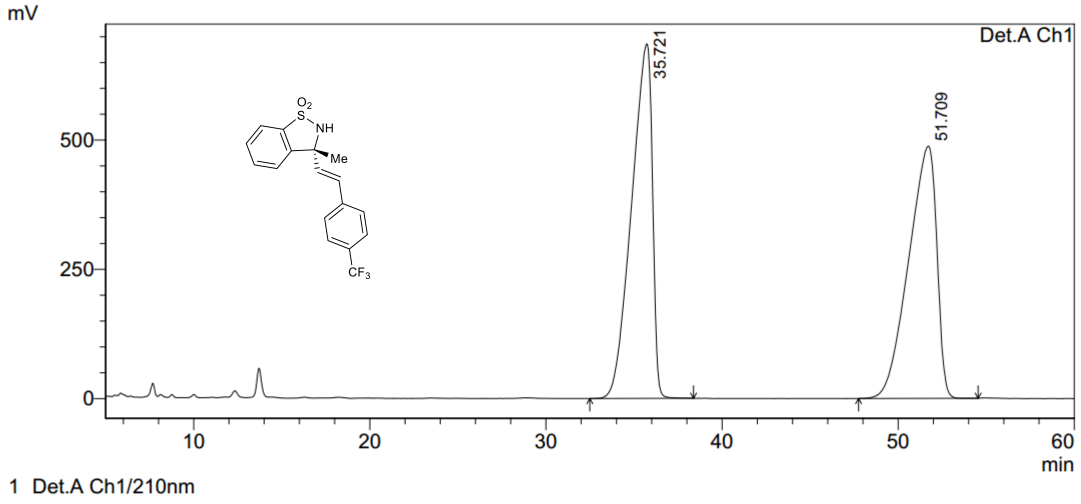

PeakTable

Detector A Ch1 210nm

Peak#	Ret. Time	Area	Height	Area %	Height %
1	35.721	56261816	685563	50.987	58.429
2	51.709	54082755	487758	49.013	41.571
Total		110344570	1173322	100.000	100.000

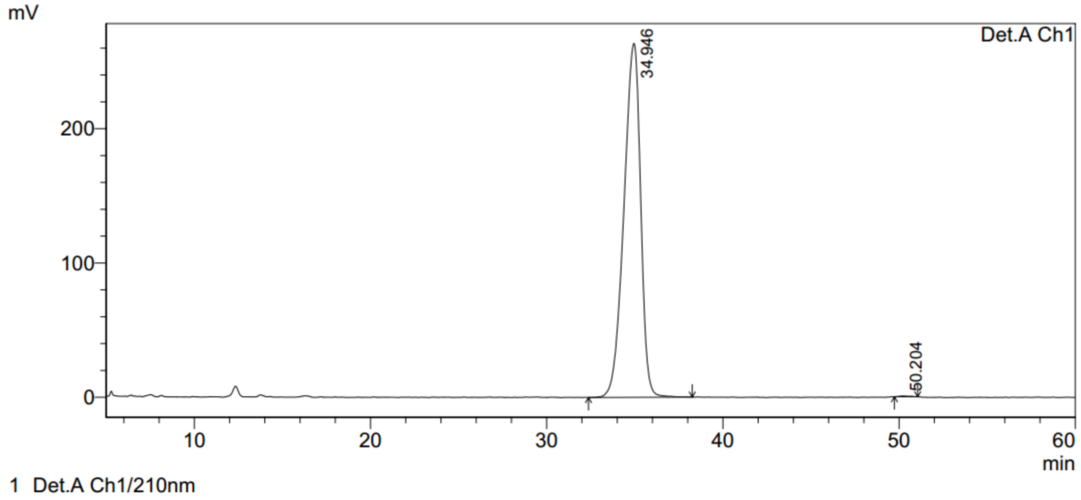

PeakTable

Detector A Ch1 210nm

Peak#	Ret. Time	Area	Height	Area %	Height %
1	34.946	17373548	263604	99.864	99.774
2	50.204	23706	598	0.136	0.226
Total		17397253	264202	100.000	100.000

Supplementary Figure 218: HPLC traces for product **3if**

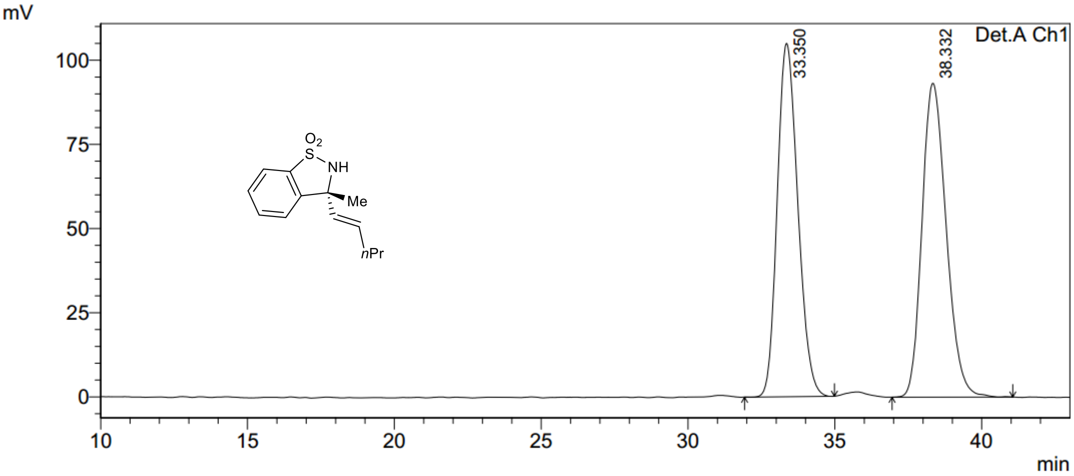

1 Det.A Ch1/210nm

PeakTable

Detector A Ch1 210nm

Peak#	Ret. Time	Area	Height	Area %	Height %
1	33.350	5111249	104943	48.855	52.956
2	38.332	5350893	93228	51.145	47.044
Total		10462142	198172	100.000	100.000

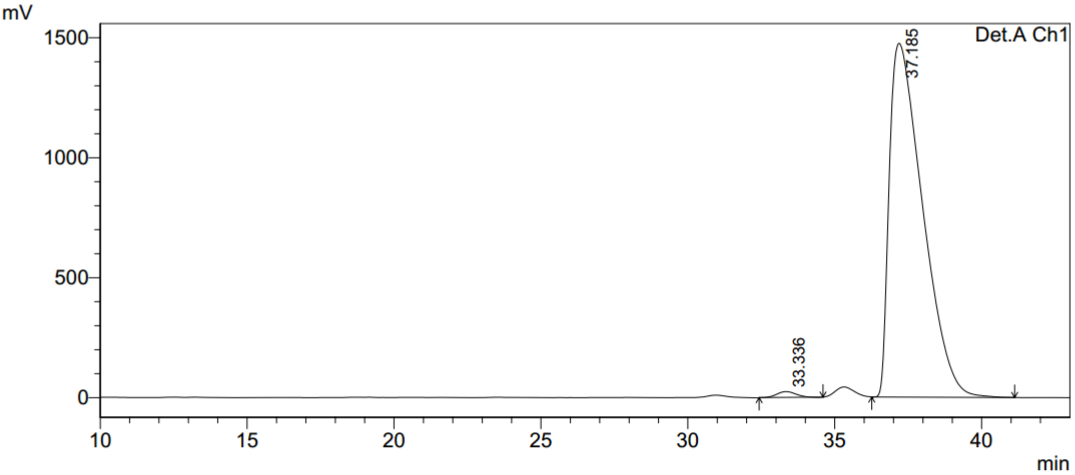

1 Det.A Ch1/210nm

PeakTable

Detector A Ch1 210nm

Peak#	Ret. Time	Area	Height	Area %	Height %
1	33.336	1074432	24106	0.916	1.609
2	37.185	116181489	1474311	99.084	98.391
Total		117255921	1498417	100.000	100.000

Supplementary Figure 219: HPLC traces for product **3ig**

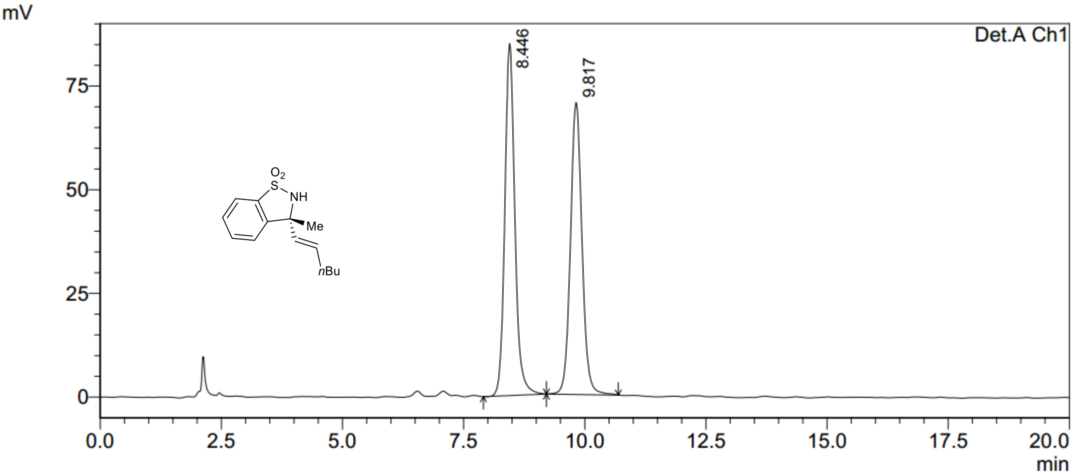

1 Det.A Ch1/210nm

PeakTable

Detector A Ch1 210nm

Peak#	Ret. Time	Area	Height	Area %	Height %
1	8.446	1224677	84934	51.215	54.685
2	9.817	1166555	70381	48.785	45.315
Total		2391232	155315	100.000	100.000

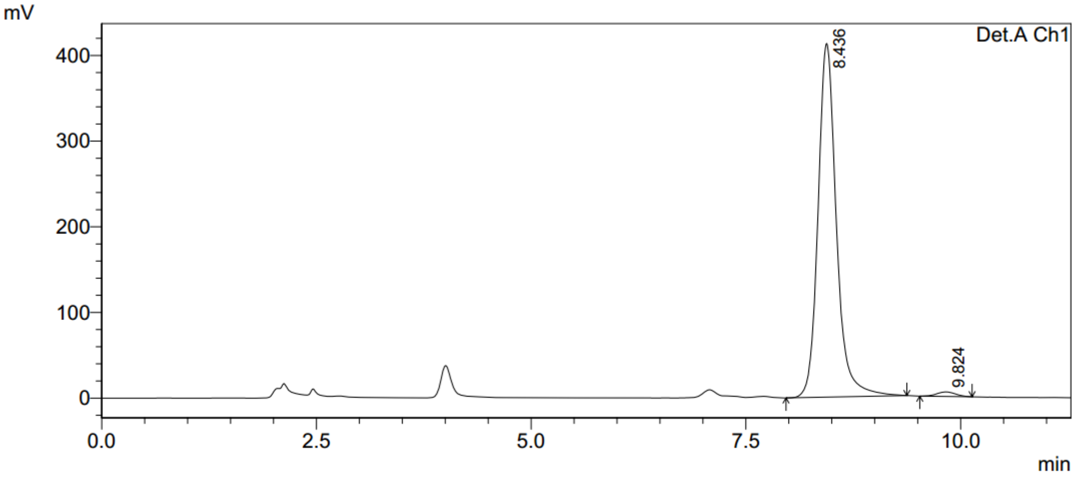

1 Det.A Ch1/210nm

PeakTable

Detector A Ch1 210nm

Peak#	Ret. Time	Area	Height	Area %	Height %
1	8.436	6122999	412977	98.704	98.726
2	9.824	80411	5328	1.296	1.274
Total		6203409	418306	100.000	100.000

Supplementary Figure 220: HPLC traces for product **3ih**

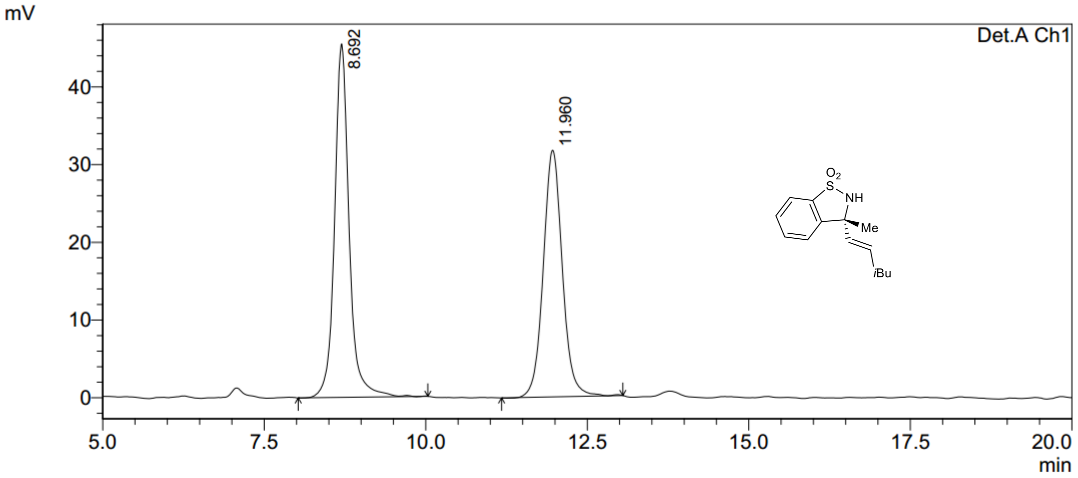

1 Det.A Ch1/210nm

PeakTable

Detector A Ch1 210nm

Peak#	Ret. Time	Area	Height	Area %	Height %
1	8.692	711113	45480	52.291	58.894
2	11.960	648799	31744	47.709	41.106
Total		1359912	77224	100.000	100.000

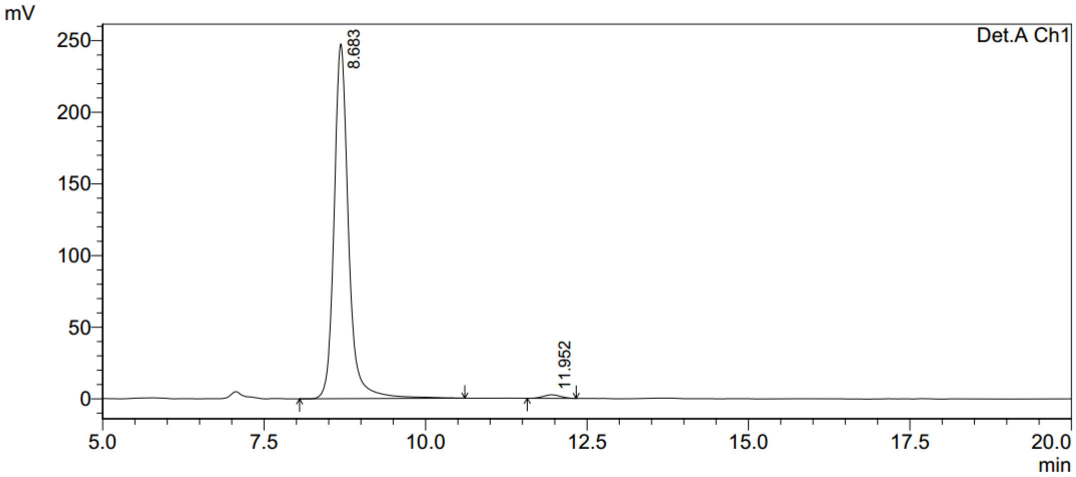

1 Det.A Ch1/210nm

PeakTable

Detector A Ch1 210nm

Peak#	Ret. Time	Area	Height	Area %	Height %
1	8.683	3841537	247551	98.808	98.991
2	11.952	46352	2522	1.192	1.009
Total		3887889	250073	100.000	100.000

Supplementary Figure 221: HPLC traces for product **3ii**

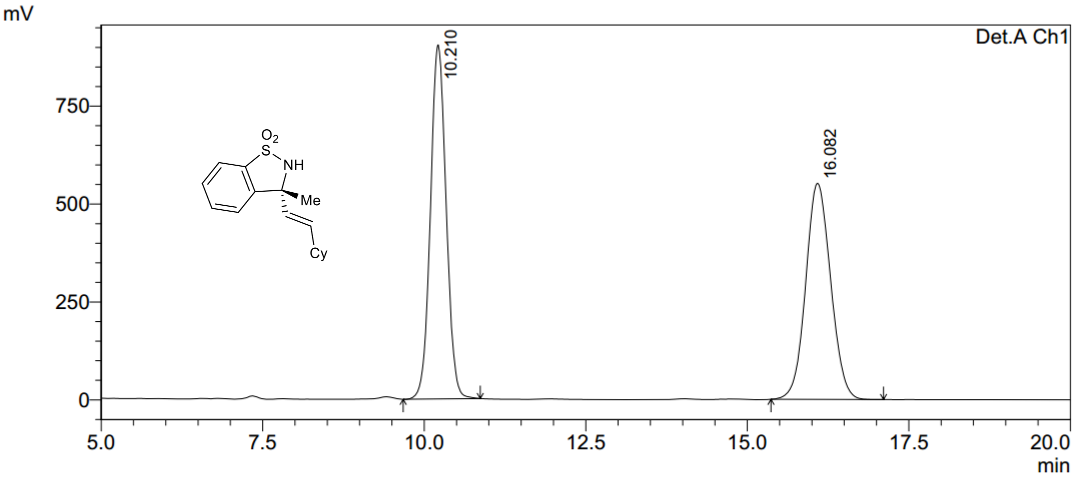

1 Det.A Ch1/210nm

PeakTable

Detector A Ch1 210nm

Peak#	Ret. Time	Area	Height	Area %	Height %
1	10.210	15311634	904213	50.807	62.103
2	16.082	14824939	551769	49.193	37.897
Total		30136572	1455982	100.000	100.000

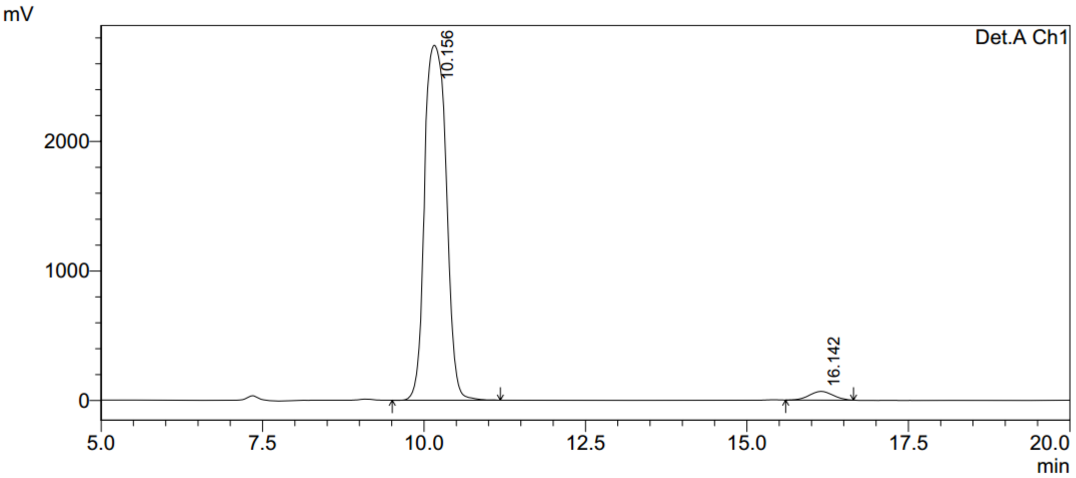

1 Det.A Ch1/210nm

PeakTable

Detector A Ch1 210nm

Peak#	Ret. Time	Area	Height	Area %	Height %
1	10.156	64147272	2740789	97.414	97.611
2	16.142	1703082	67073	2.586	2.389
Total		65850354	2807862	100.000	100.000

Supplementary Figure 222: HPLC traces for product **3ij**

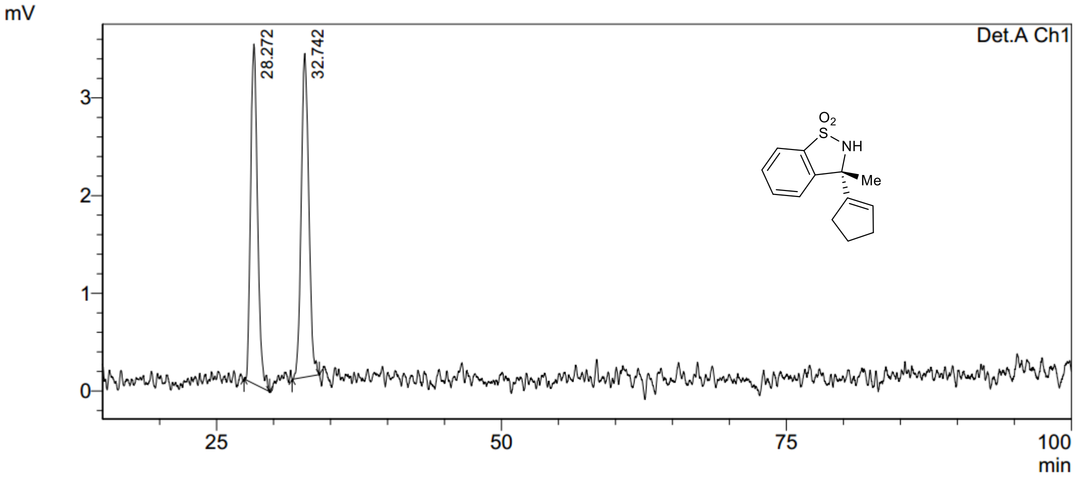

1 Det.A Ch1/210nm

PeakTable

Detector A Ch1 210nm

Peak#	Ret. Time	Area	Height	Area %	Height %
1	28.272	147122	3479	49.077	51.212
2	32.742	152655	3314	50.923	48.788
Total		299777	6793	100.000	100.000

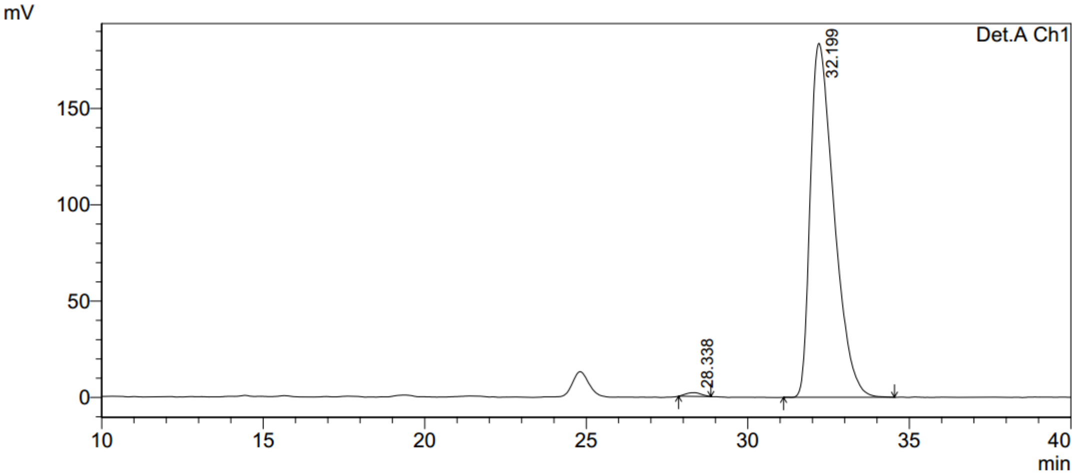

1 Det.A Ch1/210nm

PeakTable

Detector A Ch1 210nm

Peak#	Ret. Time	Area	Height	Area %	Height %
1	28.338	63439	1900	0.672	1.024
2	32.199	9378736	183714	99.328	98.976
Total		9442176	185614	100.000	100.000

Supplementary Figure 223: HPLC traces for product **3ik**

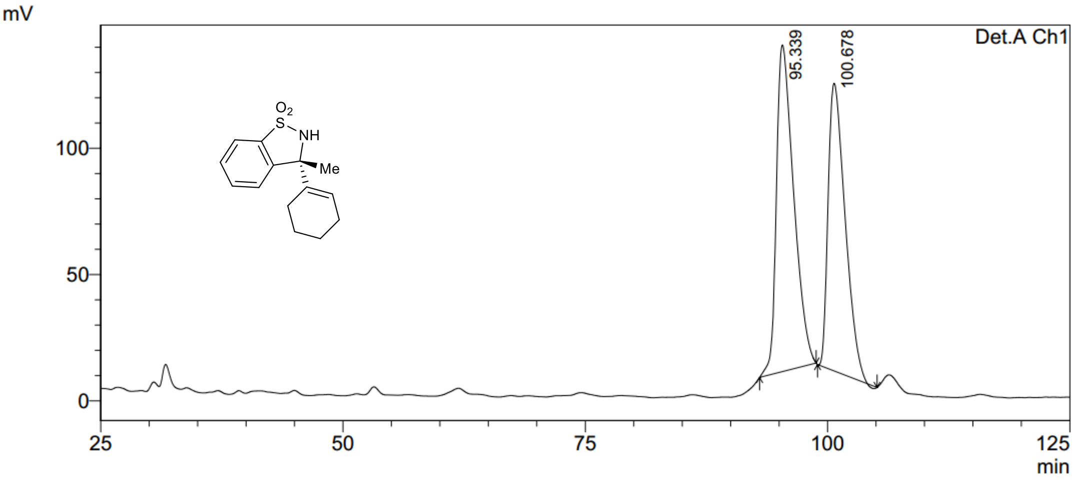

1 Det.A Ch1/210nm

PeakTable

Detector A Ch1 210nm

Peak#	Ret. Time	Area	Height	Area %	Height %
1	95.339	16046418	129366	53.575	53.181
2	100.678	13905094	113889	46.425	46.819
Total		29951512	243256	100.000	100.000

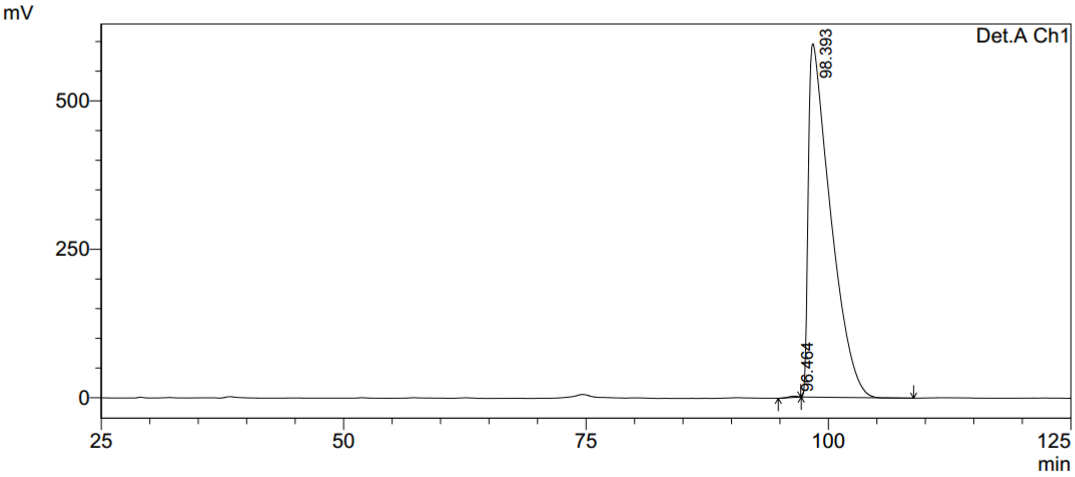

1 Det.A Ch1/210nm

PeakTable

Detector A Ch1 210nm

Peak#	Ret. Time	Area	Height	Area %	Height %
1	96.464	121819	2083	0.128	0.349
2	98.393	95032723	595275	99.872	99.651
Total		95154541	597357	100.000	100.000

Supplementary Figure 224: HPLC traces for product **3il**

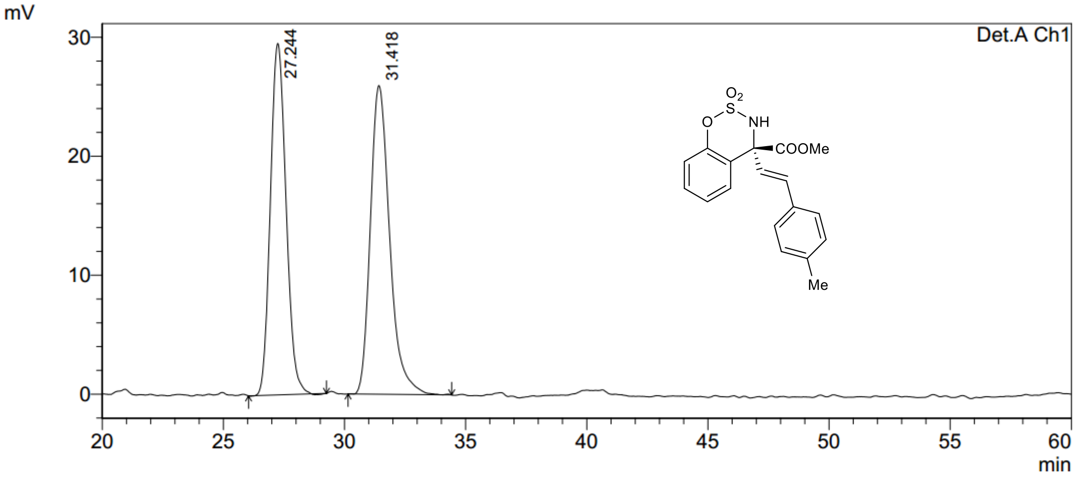

1 Det.A Ch1/210nm

PeakTable

Detector A Ch1 210nm

Peak#	Ret. Time	Area	Height	Area %	Height %
1	27.244	1324103	29540	47.980	53.240
2	31.418	1435593	25945	52.020	46.760
Total		2759695	55485	100.000	100.000

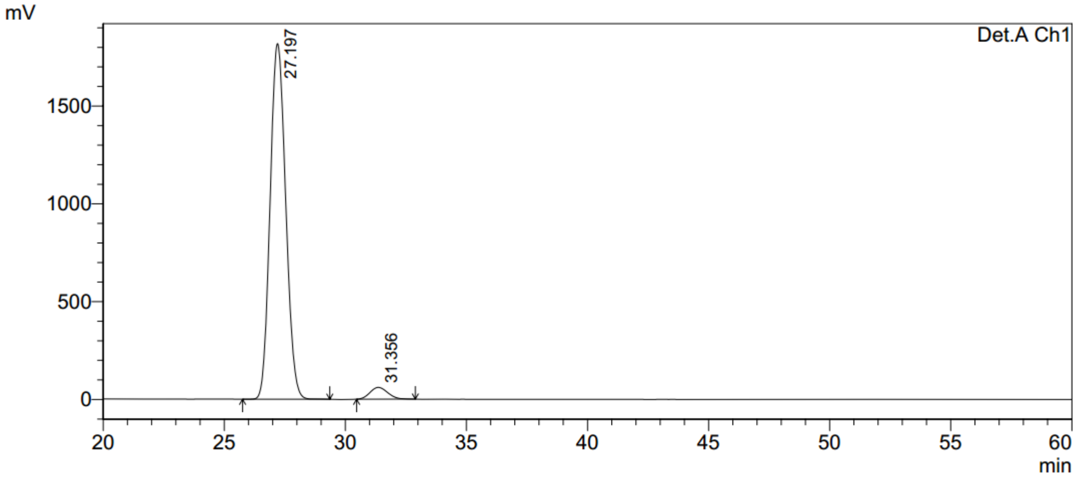

1 Det.A Ch1/210nm

PeakTable

Detector A Ch1 210nm

Peak#	Ret. Time	Area	Height	Area %	Height %
1	27.197	81265209	1818131	96.381	96.804
2	31.356	3050985	60035	3.619	3.196
Total		84316194	1878166	100.000	100.000

Supplementary Figure 225: HPLC traces for product **3ab**

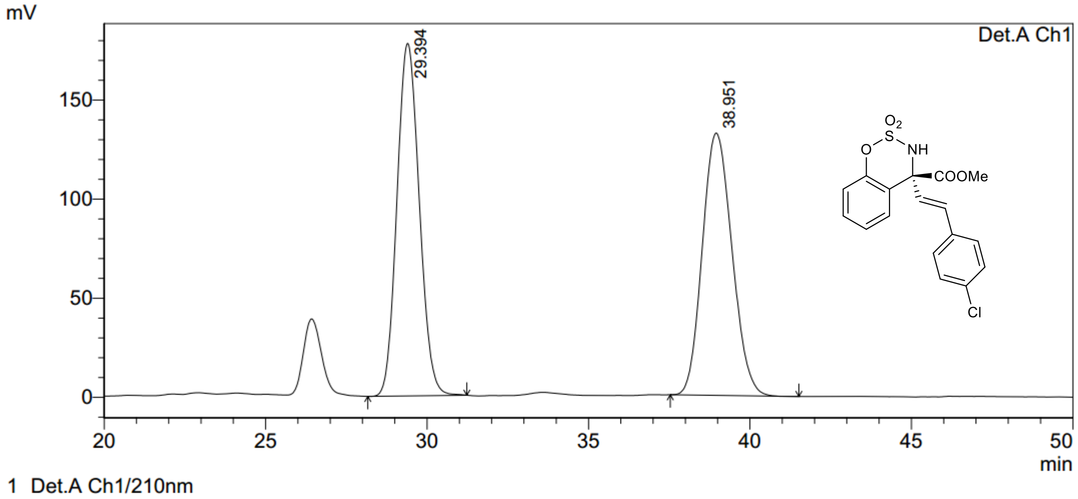

PeakTable

Detector A Ch1 210nm

Peak#	Ret. Time	Area	Height	Area %	Height %
1	29.394	8553831	177908	50.271	57.336
2	38.951	8461542	132382	49.729	42.664
Total		17015373	310289	100.000	100.000

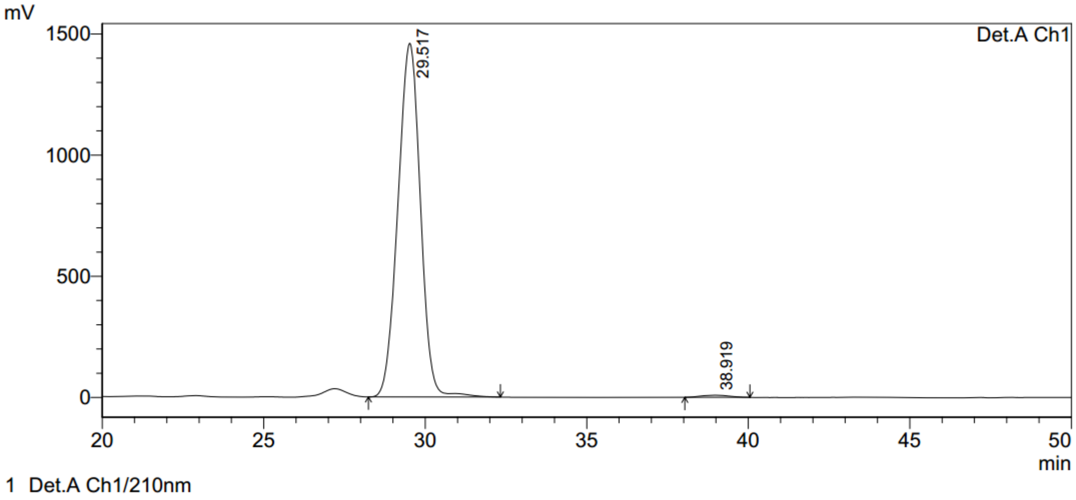

PeakTable

Detector A Ch1 210nm

Peak#	Ret. Time	Area	Height	Area %	Height %
1	29.517	70567693	1459400	99.261	99.389
2	38.919	525231	8964	0.739	0.611
Total		71092924	1468364	100.000	100.000

Supplementary Figure 226: HPLC traces for product **3ac**

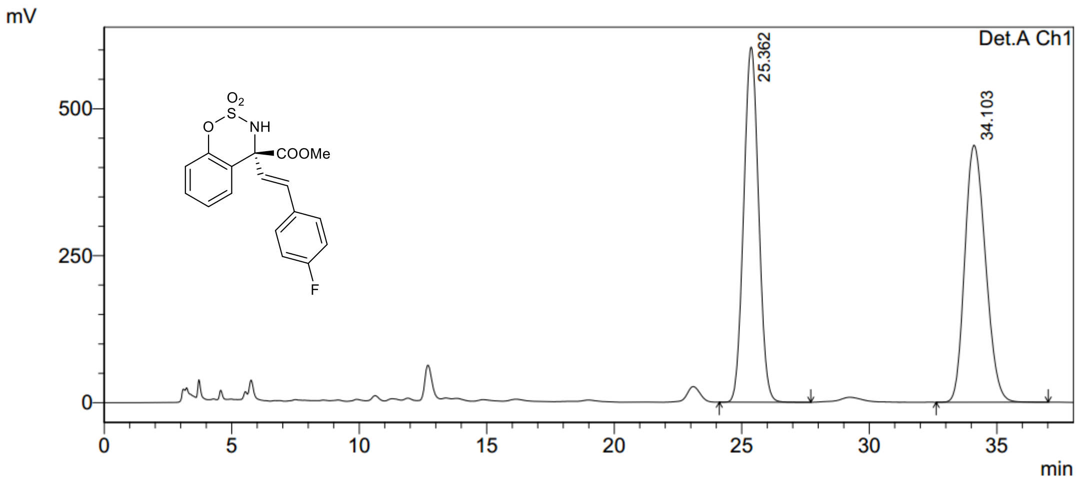

1 Det.A Ch1/210nm

PeakTable

Detector A Ch1 210nm

Peak#	Ret. Time	Area	Height	Area %	Height %
1	25.362	24542788	604285	49.804	58.017
2	34.103	24735810	437282	50.196	41.983
Total		49278598	1041568	100.000	100.000

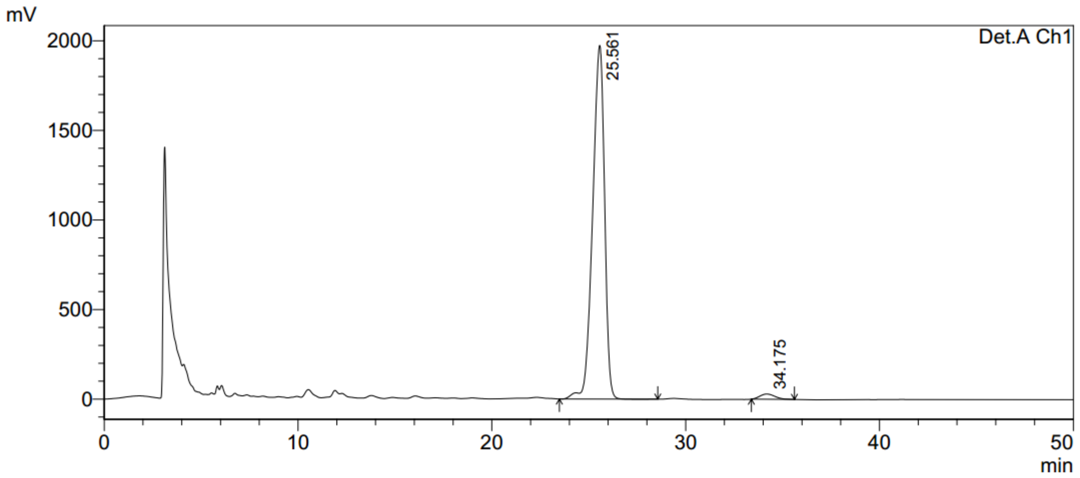

1 Det.A Ch1/210nm

PeakTable

Detector A Ch1 210nm

Peak#	Ret. Time	Area	Height	Area %	Height %
1	25.561	84141414	1974487	98.155	98.543
2	34.175	1581528	29191	1.845	1.457
Total		85722942	2003678	100.000	100.000

Supplementary Figure 227: HPLC traces for product **3ad**

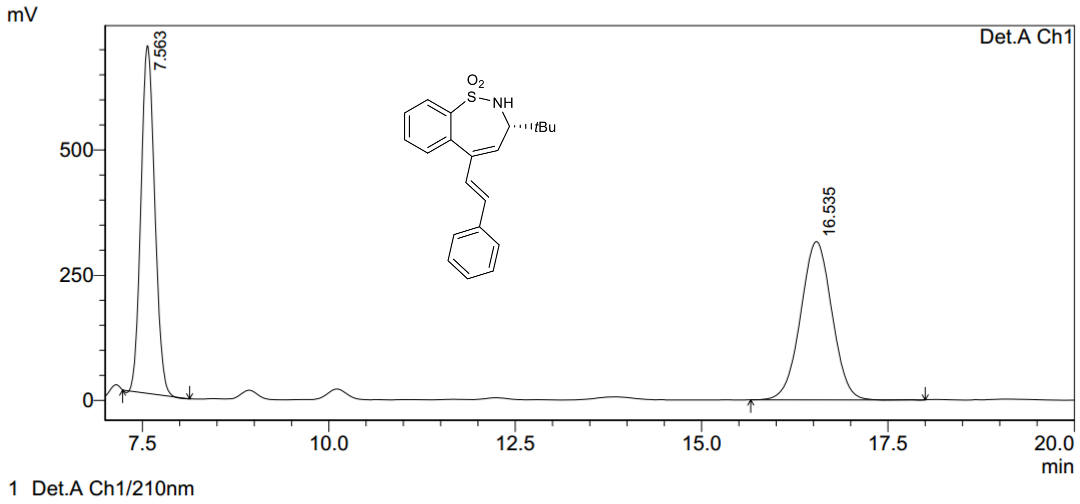

PeakTable

Detector A Ch1 210nm

Peak#	Ret. Time	Area	Height	Area %	Height %
1	7.563	8905608	694354	49.054	68.711
2	16.535	9249004	316182	50.946	31.289
Total		18154612	1010536	100.000	100.000

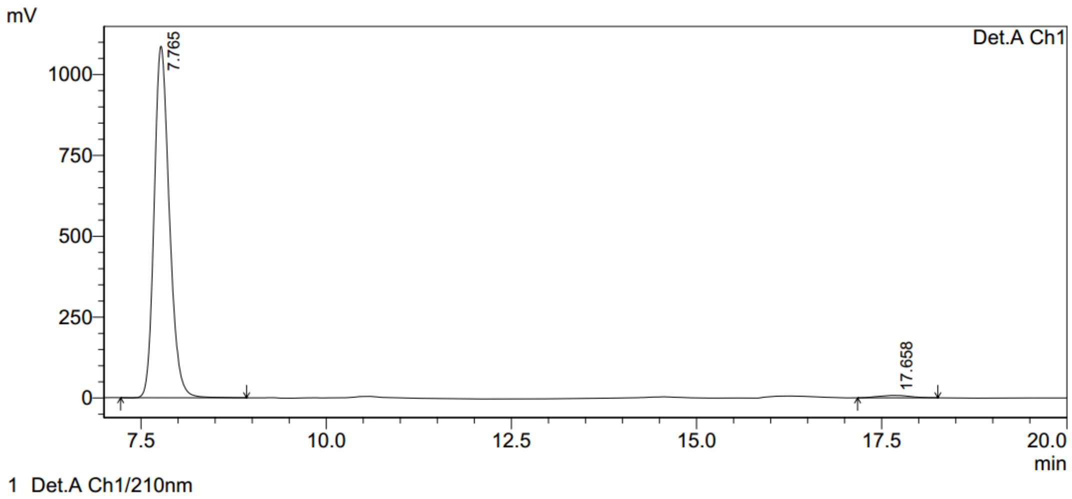

PeakTable

Detector A Ch1 210nm

Peak#	Ret. Time	Area	Height	Area %	Height %
1	7.765	15499826	1087282	98.550	99.292
2	17.658	227975	7747	1.450	0.708
Total		15727801	1095029	100.000	100.000

Supplementary Figure 228: HPLC traces for product **5a**

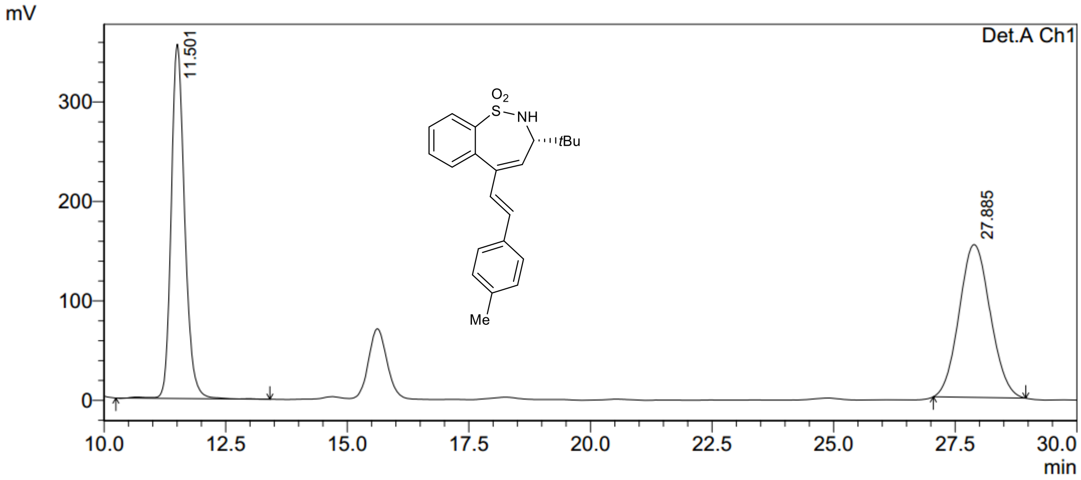

1 Det.A Ch1/210nm

PeakTable

Detector A Ch1 210nm

Peak#	Ret. Time	Area	Height	Area %	Height %
1	11.501	6858795	356369	50.037	69.851
2	27.885	6848722	153813	49.963	30.149
Total		13707517	510182	100.000	100.000

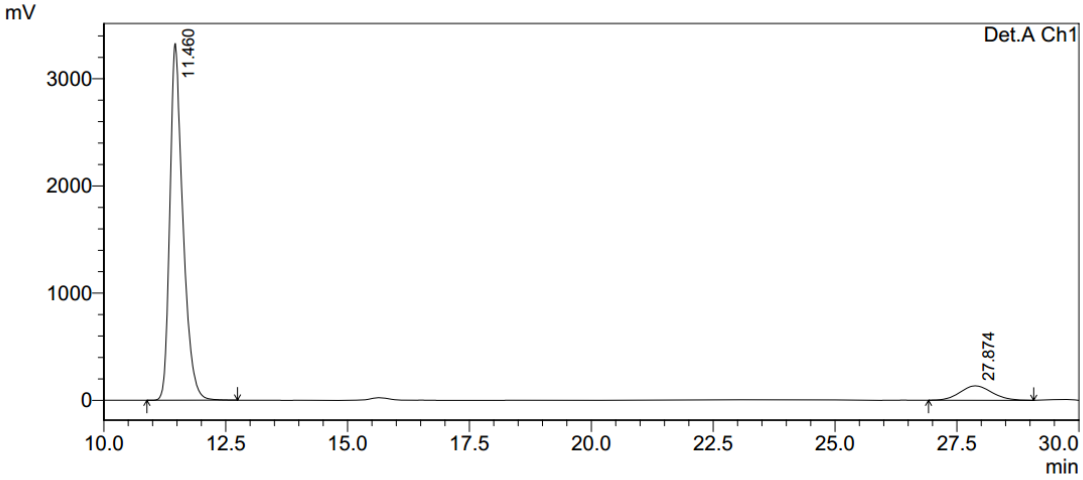

1 Det.A Ch1/210nm

PeakTable

Detector A Ch1 210nm

Peak#	Ret. Time	Area	Height	Area %	Height %
1	11.460	61635331	3329123	90.967	96.118
2	27.874	6120220	134471	9.033	3.882
Total		67755551	3463593	100.000	100.000

Supplementary Figure 229: HPLC traces for product **5b**

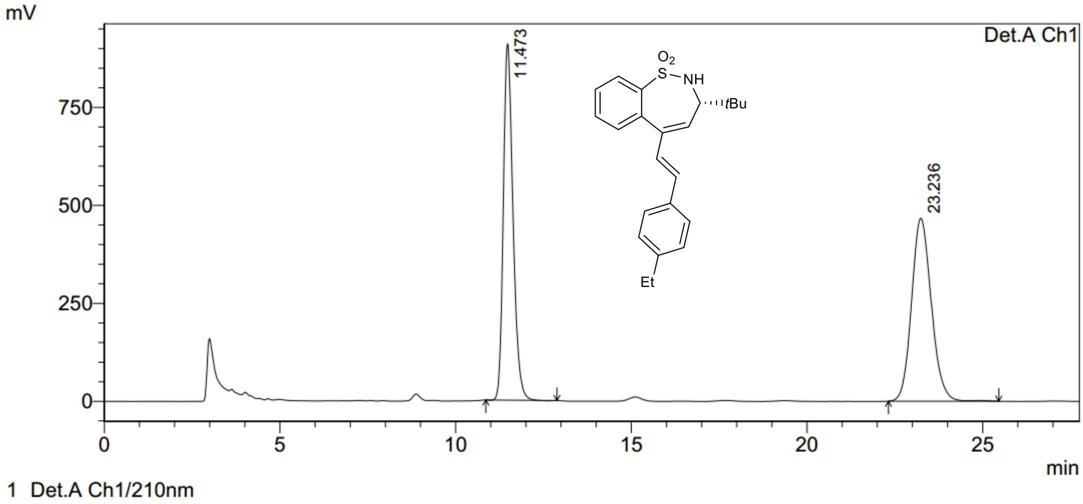

PeakTable

Detector A Ch1 210nm

Peak#	Ret. Time	Area	Height	Area %	Height %
1	11.473	17318745	909176	48.725	66.076
2	23.236	18225040	466771	51.275	33.924
Total		35543785	1375947	100.000	100.000

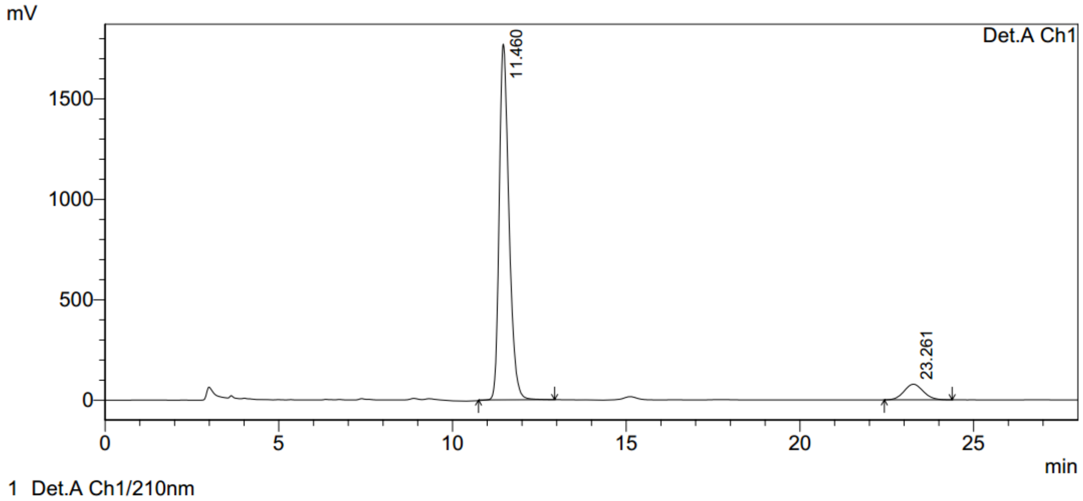

PeakTable

Detector A Ch1 210nm

Peak#	Ret. Time	Area	Height	Area %	Height %
1	11.460	34566761	1771355	92.014	95.792
2	23.261	3000135	77813	7.986	4.208
Total		37566897	1849167	100.000	100.000

Supplementary Figure 230: HPLC traces for product **5c**

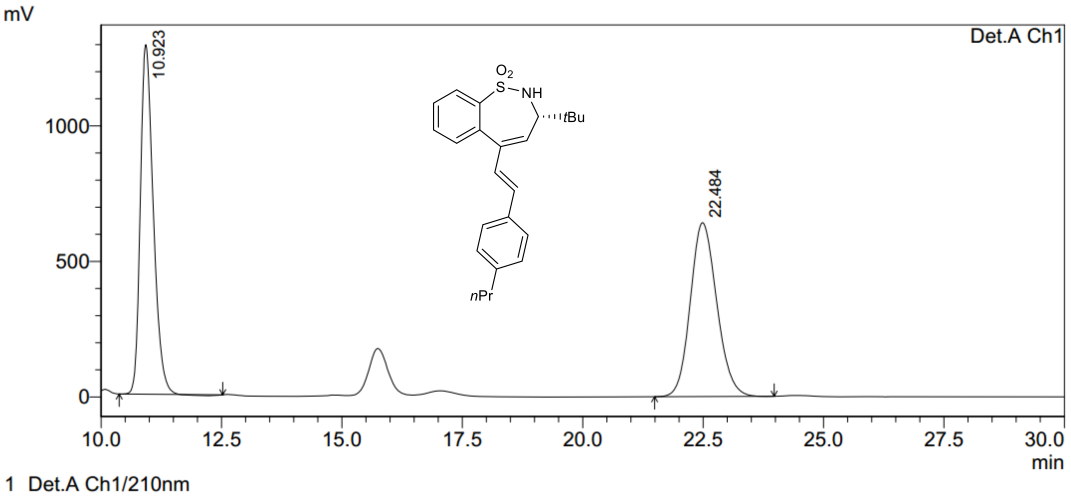

PeakTable

Detector A Ch1 210nm

Peak#	Ret. Time	Area	Height	Area %	Height %
1	10.923	23971061	1289541	49.460	66.782
2	22.484	24494176	641427	50.540	33.218
Total		48465237	1930968	100.000	100.000

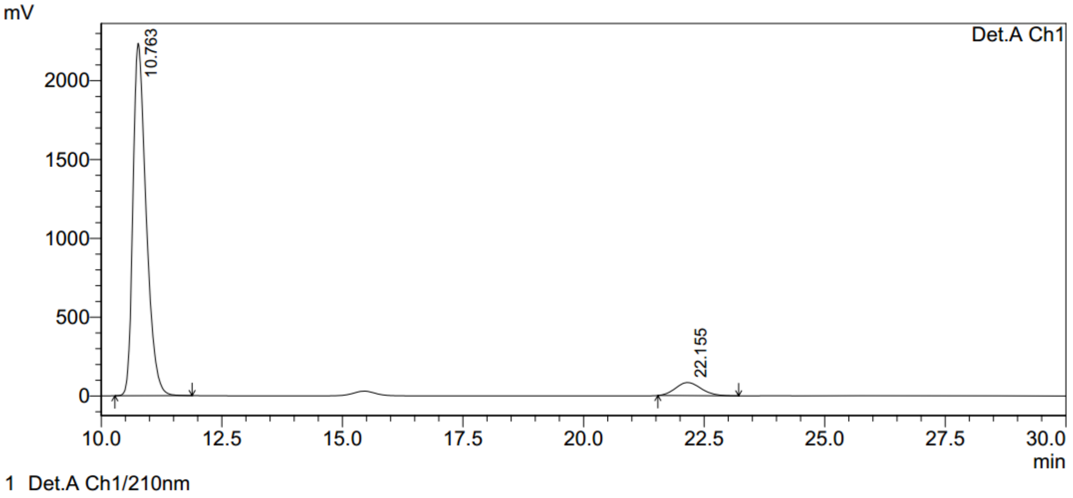

PeakTable

Detector A Ch1 210nm

Peak#	Ret. Time	Area	Height	Area %	Height %
1	10.763	42507976	2236292	93.337	96.428
2	22.155	3034683	82836	6.663	3.572
Total		45542659	2319127	100.000	100.000

Supplementary Figure 231: HPLC traces for product **5d**

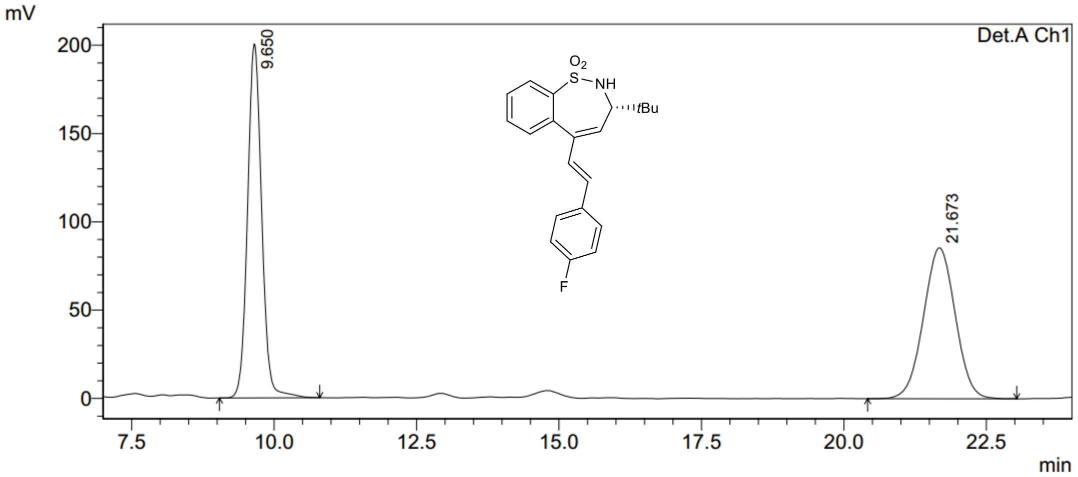

1 Det.A Ch1/210nm

PeakTable

Detector A Ch1 210nm

Peak#	Ret. Time	Area	Height	Area %	Height %
1	9.650	3456760	200446	51.060	70.113
2	21.673	3313214	85444	48.940	29.887
Total		6769974	285890	100.000	100.000

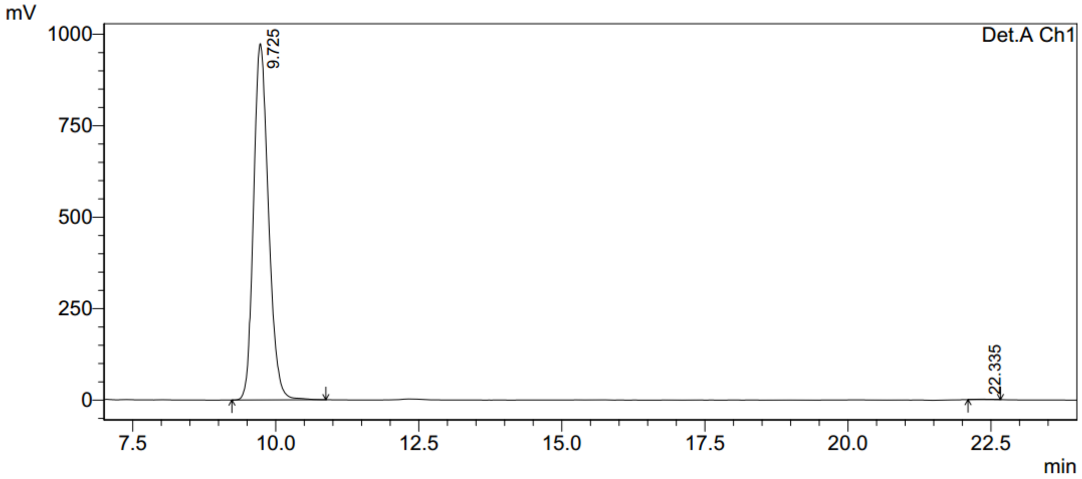

1 Det.A Ch1/210nm

PeakTable

Detector A Ch1 210nm

Peak#	Ret. Time	Area	Height	Area %	Height %
1	9.725	17633048	974038	99.886	99.907
2	22.335	20202	911	0.114	0.093
Total		17653250	974949	100.000	100.000

Supplementary Figure 232: HPLC traces for product **5e**

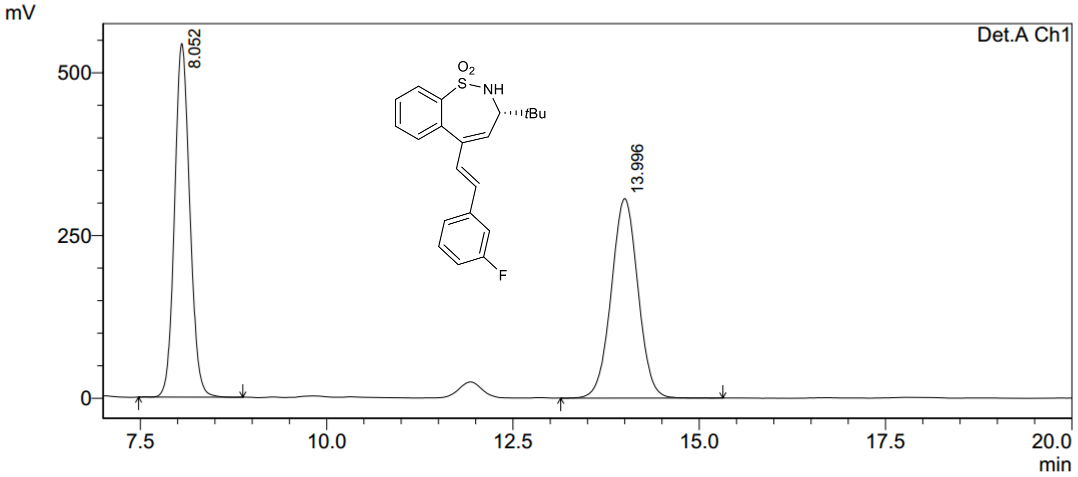

PeakTable

Detector A Ch1 210nm

Peak#	Ret. Time	Area	Height	Area %	Height %
1	8.052	7709698	543269	50.262	63.935
2	13.996	7629287	306450	49.738	36.065
Total		15338985	849719	100.000	100.000

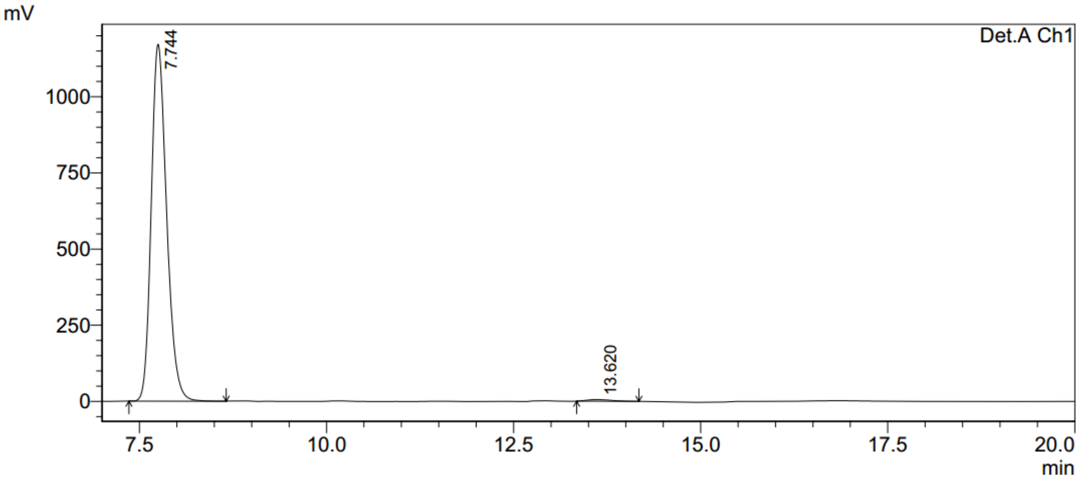

PeakTable

Detector A Ch1 210nm

Peak#	Ret. Time	Area	Height	Area %	Height %
1	7.744	16773064	1171782	99.296	99.545
2	13.620	118878	5356	0.704	0.455
Total		16891942	1177138	100.000	100.000

Supplementary Figure 233: HPLC traces for product **5f**

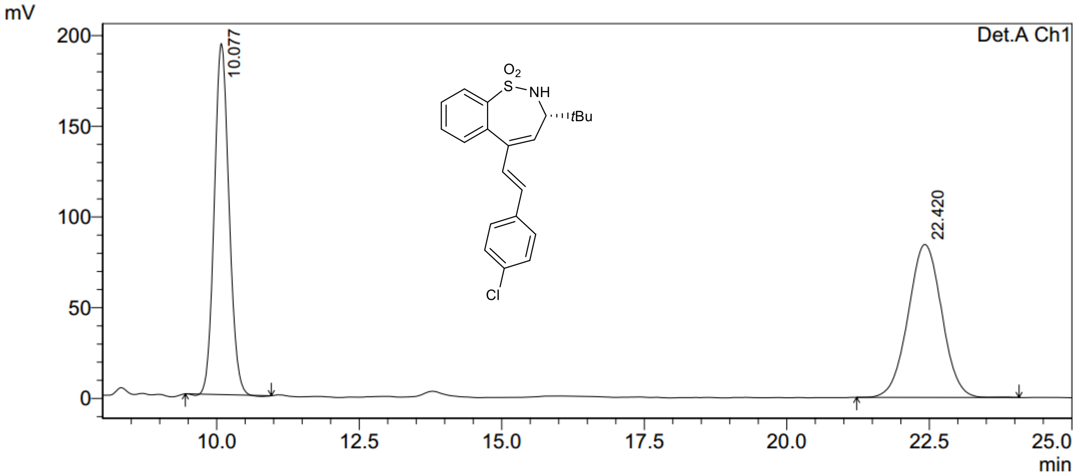

1 Det.A Ch1/210nm

PeakTable

Detector A Ch1 210nm

Peak#	Ret. Time	Area	Height	Area %	Height %
1	10.077	3455306	193562	50.006	69.649
2	22.420	3454508	84348	49.994	30.351
Total		6909813	277910	100.000	100.000

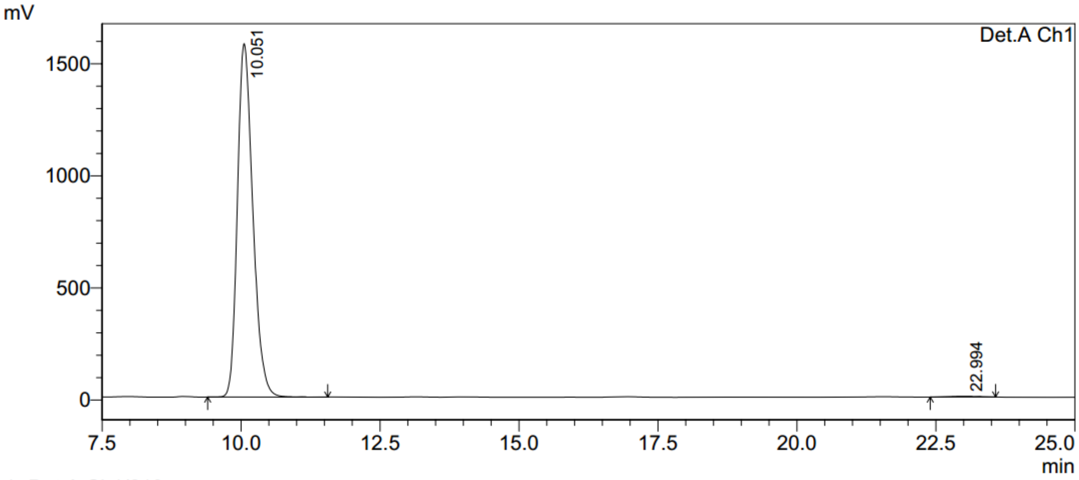

1 Det.A Ch1/210nm

PeakTable

Detector A Ch1 210nm

Peak#	Ret. Time	Area	Height	Area %	Height %
1	10.051	30407041	1576572	99.573	99.777
2	22.994	130360	3524	0.427	0.223
Total		30537401	1580096	100.000	100.000

Supplementary Figure 234: HPLC traces for product **5g**

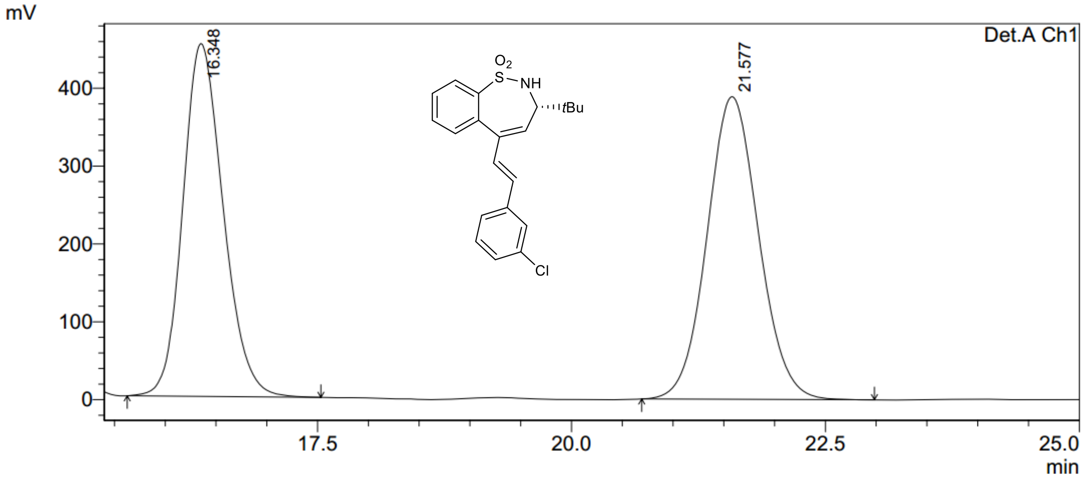

1 Det.A Ch1/210nm

PeakTable

Detector A Ch1 210nm

Peak#	Ret. Time	Area	Height	Area %	Height %
1	16.348	12521206	453055	47.688	53.827
2	21.577	13735416	388630	52.312	46.173
Total		26256622	841685	100.000	100.000

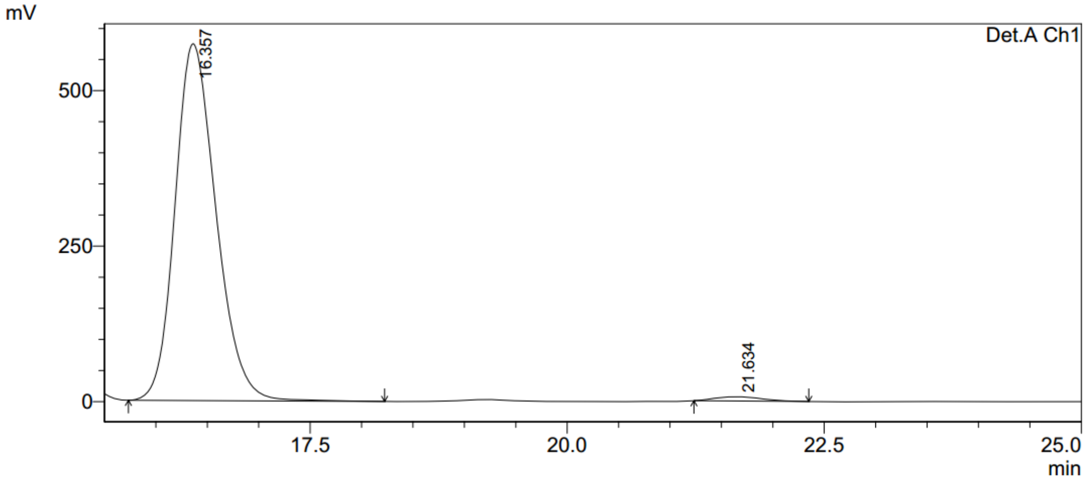

1 Det.A Ch1/210nm

PeakTable

Detector A Ch1 210nm

Peak#	Ret. Time	Area	Height	Area %	Height %
1	16.357	15929781	573730	98.644	98.796
2	21.634	219034	6991	1.356	1.204
Total		16148815	580722	100.000	100.000

Supplementary Figure 235: HPLC traces for product **5h**

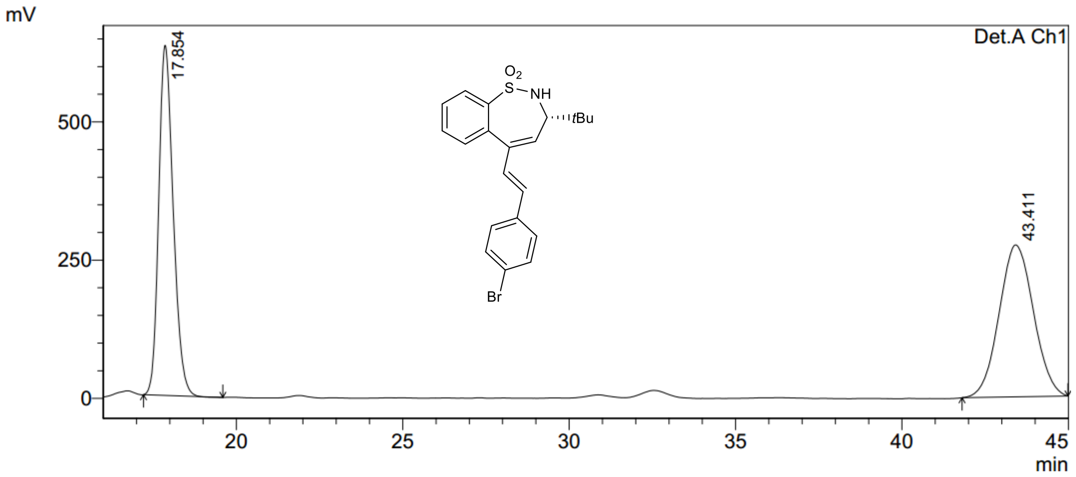

1 Det.A Ch1/210nm

PeakTable

Detector A Ch1 210nm

Peak#	Ret. Time	Area	Height	Area %	Height %
1	17.854	19140727	633255	49.226	69.739
2	43.411	19743015	274775	50.774	30.261
Total		38883743	908030	100.000	100.000

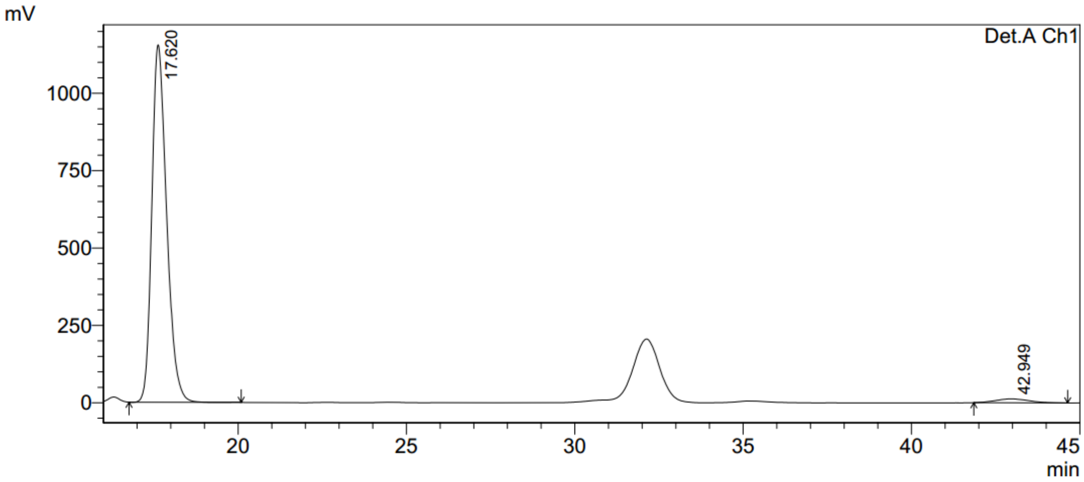

1 Det.A Ch1/210nm

PeakTable

Detector A Ch1 210nm

Peak#	Ret. Time	Area	Height	Area %	Height %
1	17.620	35239766	1155095	97.628	98.921
2	42.949	856143	12597	2.372	1.079
Total		36095909	1167692	100.000	100.000

Supplementary Figure 236: HPLC traces for product **5i**

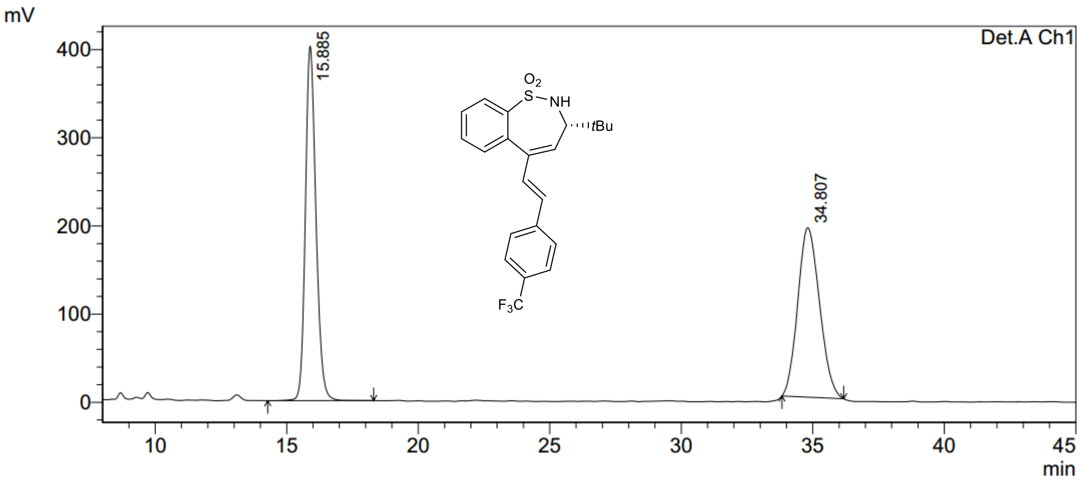

1 Det.A Ch1/210nm

PeakTable

Detector A Ch1 210nm

Peak#	Ret. Time	Area	Height	Area %	Height %
1	15.885	11211846	402077	50.324	67.660
2	34.807	11067618	192187	49.676	32.340
Total		22279464	594263	100.000	100.000

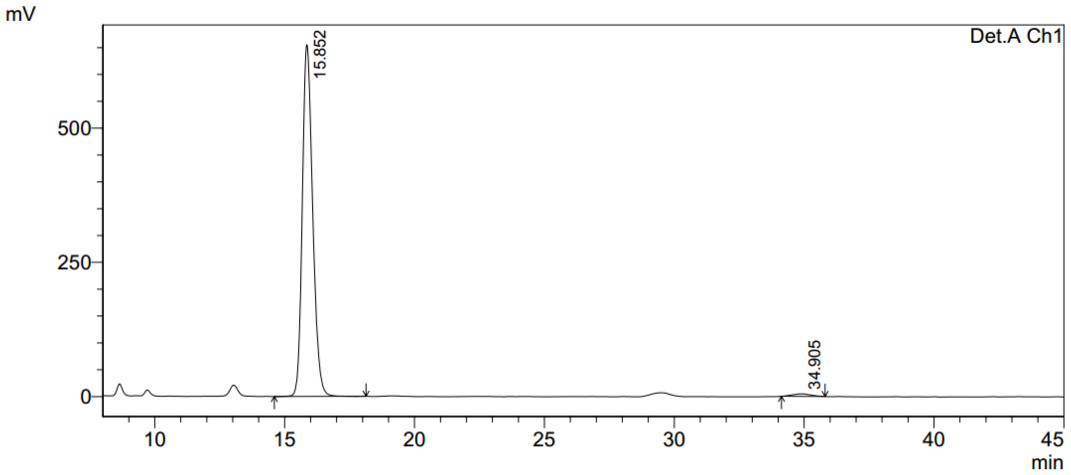

1 Det.A Ch1/210nm

PeakTable

Detector A Ch1 210nm

Peak#	Ret. Time	Area	Height	Area %	Height %
1	15.852	18125727	655305	98.743	99.290
2	34.905	230650	4688	1.257	0.710
Total		18356377	659993	100.000	100.000

Supplementary Figure 237: HPLC traces for product **5j**

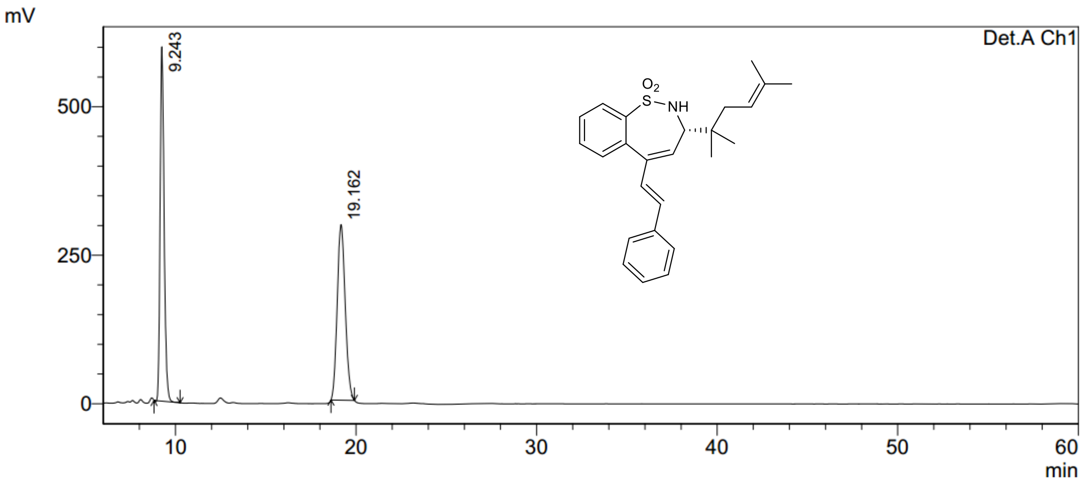

1 Det.A Ch1/210nm

PeakTable

检测器 A Ch1 210nm

Peak#	Ret. Time	Area	Height	Area %	Height %
1	9.243	9176353	596785	50.194	66.865
2	19.162	9105428	295740	49.806	33.135
Total		18281782	892525	100.000	100.000

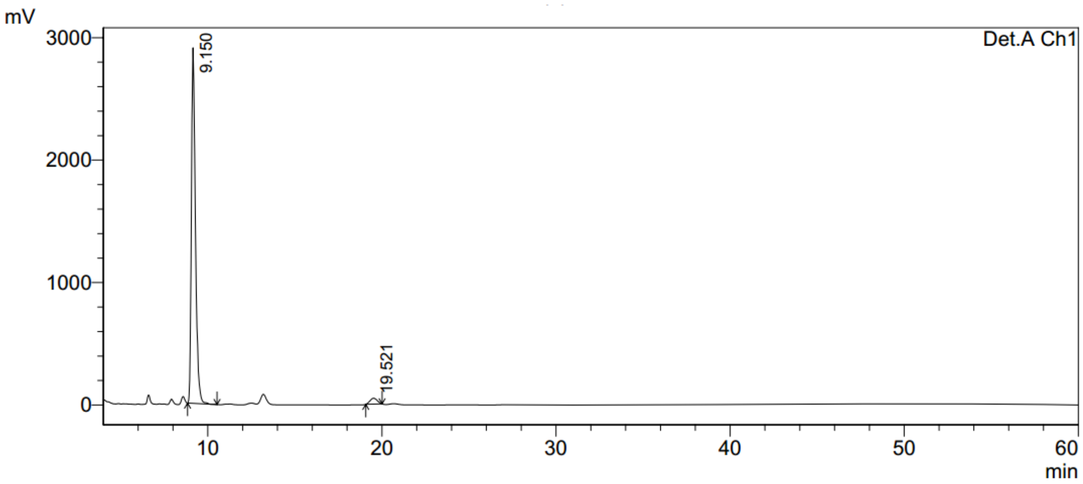

1 Det.A Ch1/210nm

PeakTable

检测器 A Ch1 210nm

Peak#	Ret. Time	Area	Height	Area %	Height %
1	9.150	46714347	2904606	97.126	98.374
2	19.521	1382089	48008	2.874	1.626
Total		48096437	2952614	100.000	100.000

Supplementary Figure 238: HPLC traces for product **5k**

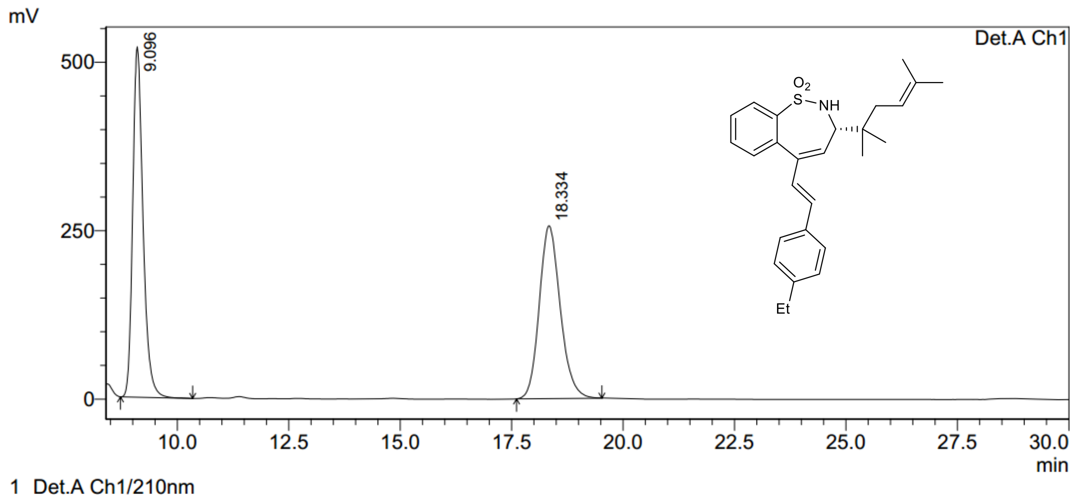

PeakTable

检测器 A Ch1 210nm

Peak#	Ret. Time	Area	Height	Area %	Height %
1	9.096	8363964	520406	49.887	66.991
2	18.334	8401998	256425	50.113	33.009
Total		16765962	776831	100.000	100.000

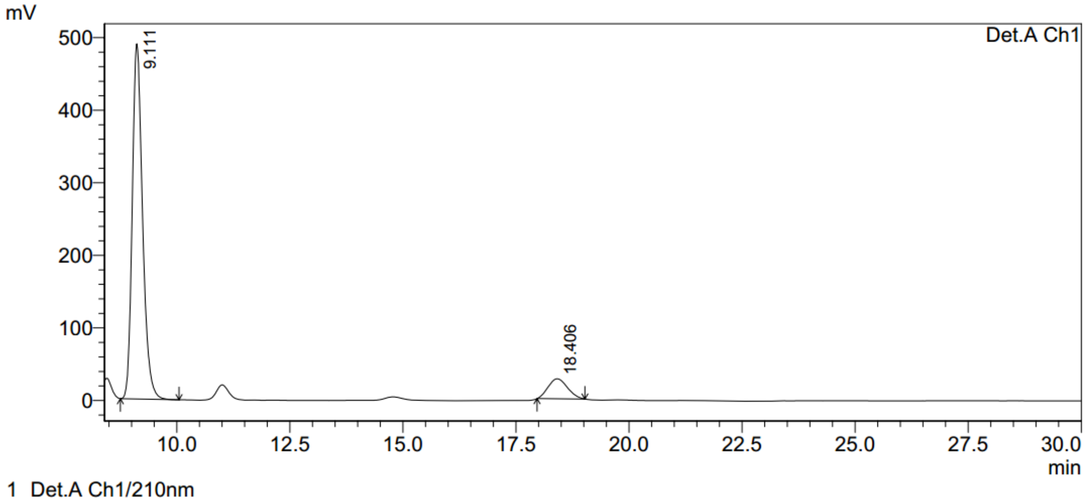

PeakTable

检测器 A Ch1 210nm

Peak#	Ret. Time	Area	Height	Area %	Height %
1	9.111	7753624	489676	90.440	94.668
2	18.406	819609	27582	9.560	5.332
Total		8573233	517258	100.000	100.000

Supplementary Figure 239: HPLC traces for product **5l**

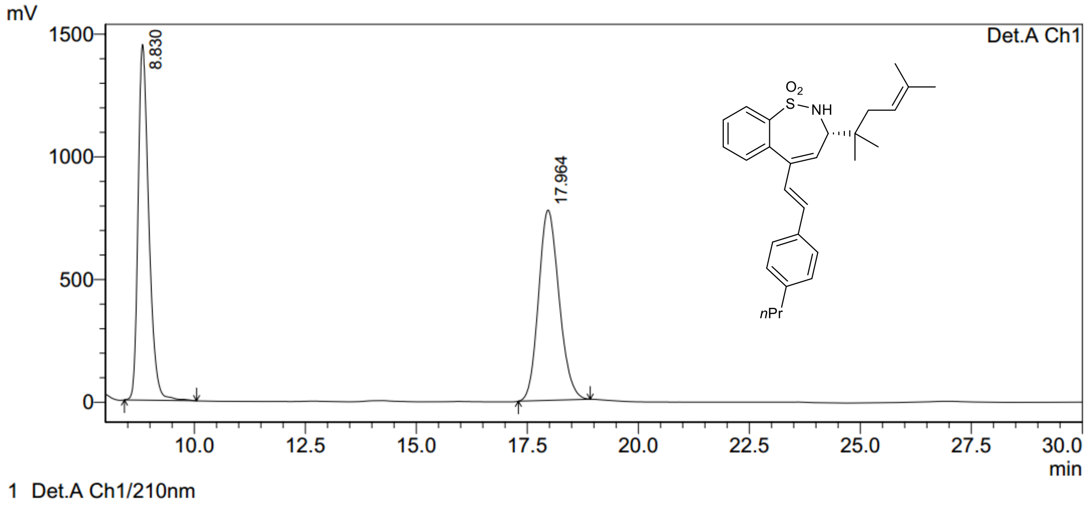

PeakTable

检测器 A Ch1 210nm

Peak#	Ret. Time	Area	Height	Area %	Height %
1	8.830	24257977	1450202	49.276	65.154
2	17.964	24970583	775619	50.724	34.846
Total		49228561	2225821	100.000	100.000

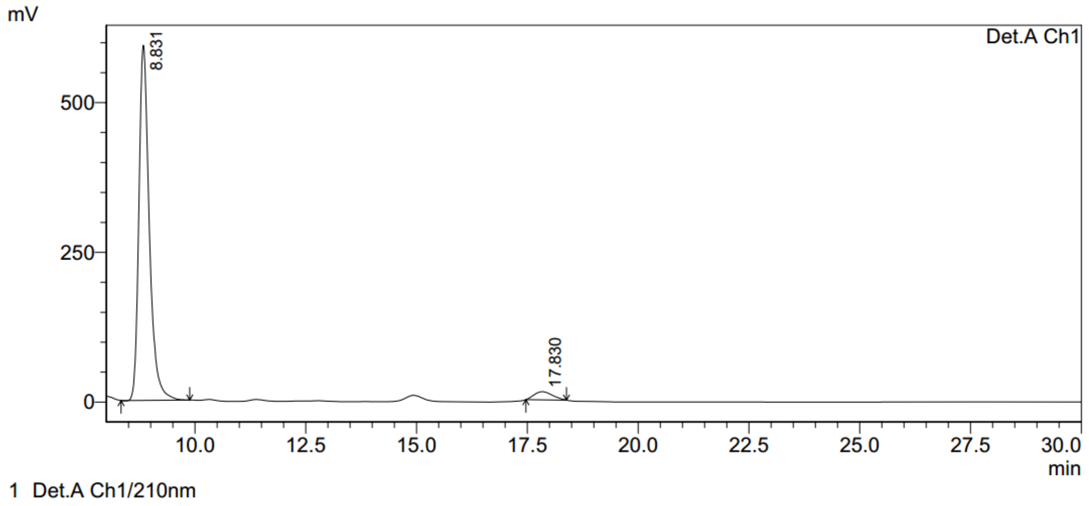

PeakTable

检测器 A Ch1 210nm

Peak#	Ret. Time	Area	Height	Area %	Height %
1	8.831	9662722	593087	96.112	97.756
2	17.830	390932	13613	3.888	2.244
Total		10053655	606700	100.000	100.000

Supplementary Figure 240: HPLC traces for product **5m**

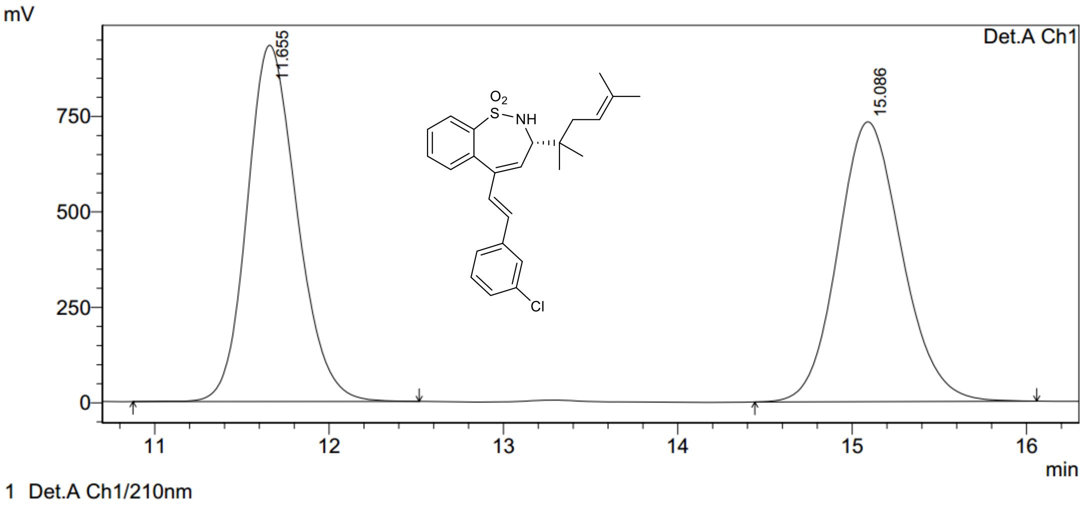

PeakTable

Detector A Ch1 210nm

Peak#	Ret. Time	Area	Height	Area %	Height %
1	11.655	18600771	932948	49.890	56.011
2	15.086	18682473	732715	50.110	43.989
Total		37283244	1665662	100.000	100.000

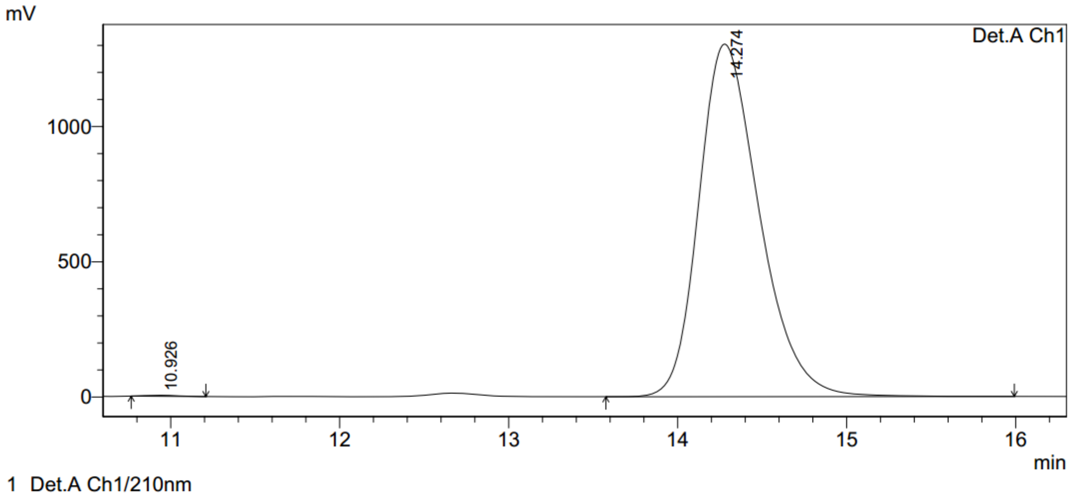

PeakTable

Detector A Ch1 210nm

Peak#	Ret. Time	Area	Height	Area %	Height %
1	10.926	48749	3702	0.150	0.283
2	14.274	32467095	1303146	99.850	99.717
Total		32515844	1306848	100.000	100.000

Supplementary Figure 241: HPLC traces for product **5n**

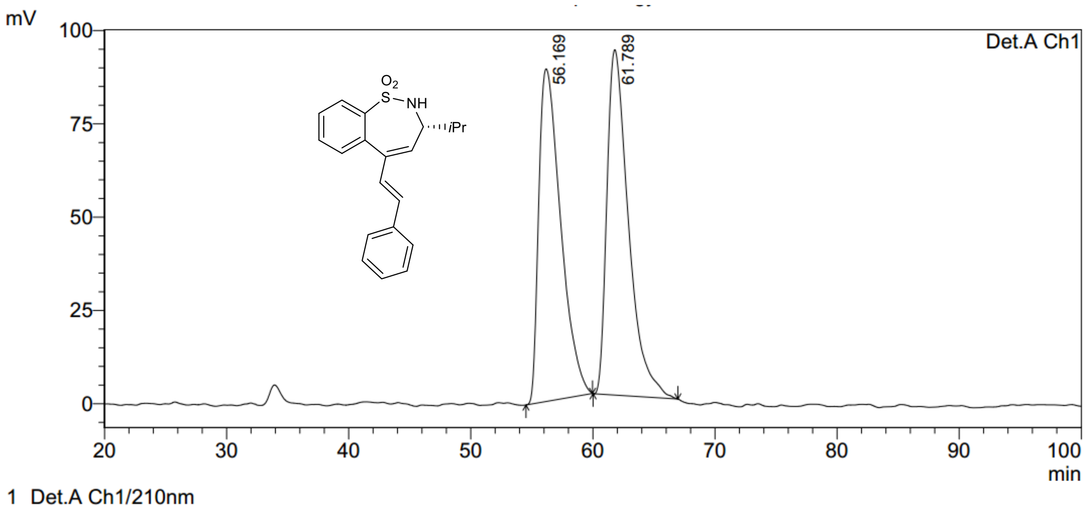

PeakTable

Detector A Ch1 210nm

Peak#	Ret. Time	Area	Height	Area %	Height %
1	56.169	11134968	89134	49.501	49.053
2	61.789	11359321	92574	50.499	50.947
Total		22494288	181708	100.000	100.000

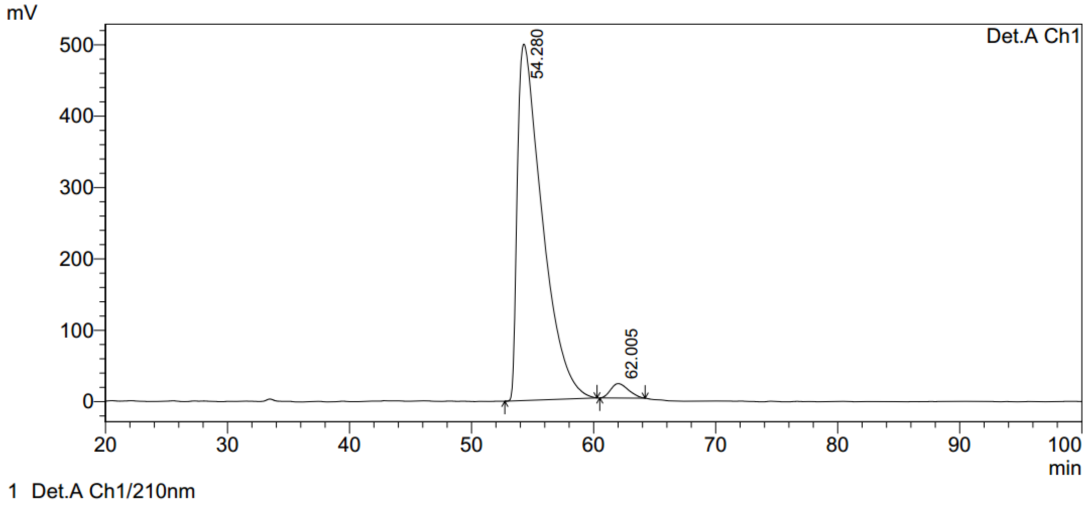

PeakTable

Detector A Ch1 210nm

Peak#	Ret. Time	Area	Height	Area %	Height %
1	54.280	70809110	499379	97.185	96.103
2	62.005	2051268	20251	2.815	3.897
Total		72860378	519630	100.000	100.000

Supplementary Figure 242: HPLC traces for product **5o**

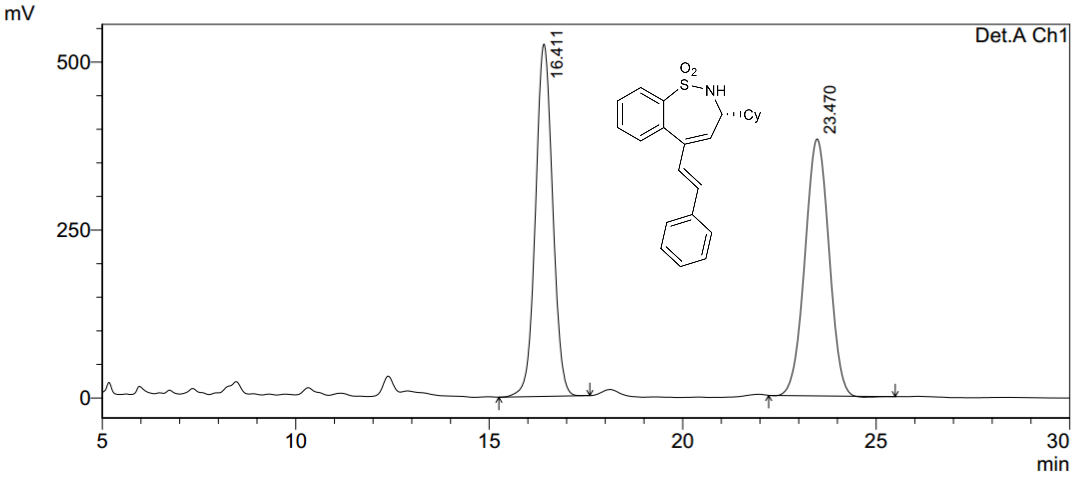

1 Det.A Ch1/210nm

PeakTable

Detector A Ch1 210nm

Peak#	Ret. Time	Area	Height	Area %	Height %
1	16.411	16184350	524396	49.510	57.834
2	23.470	16504383	382336	50.490	42.166
Total		32688732	906731	100.000	100.000

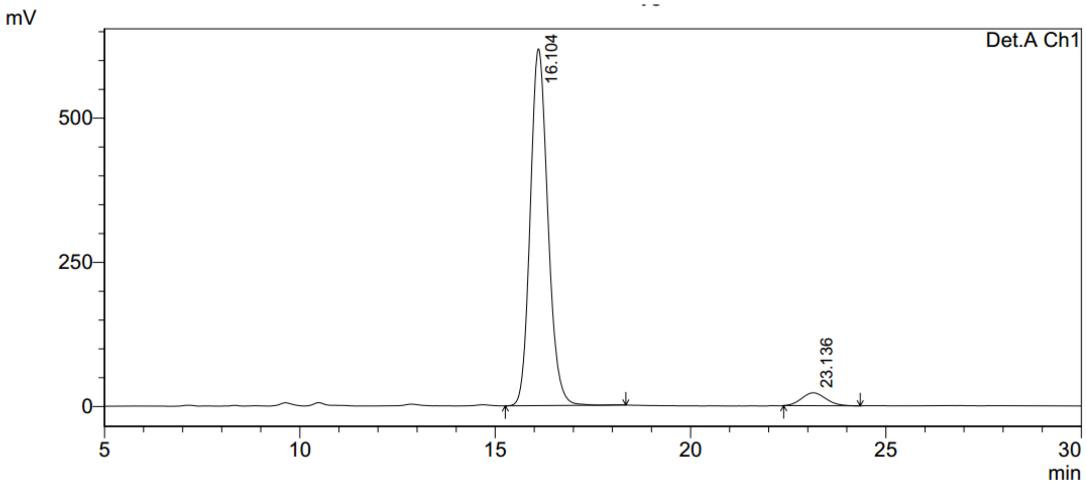

1 Det.A Ch1/210nm

PeakTable

检测器 A Ch1 210nm

Peak#	Ret. Time	Area	Height	Area %	Height %
1	16.104	19442457	618837	95.302	96.512
2	23.136	958327	22363	4.698	3.488
Total		20400784	641200	100.000	100.000

Supplementary Figure 243: HPLC traces for product **5p**

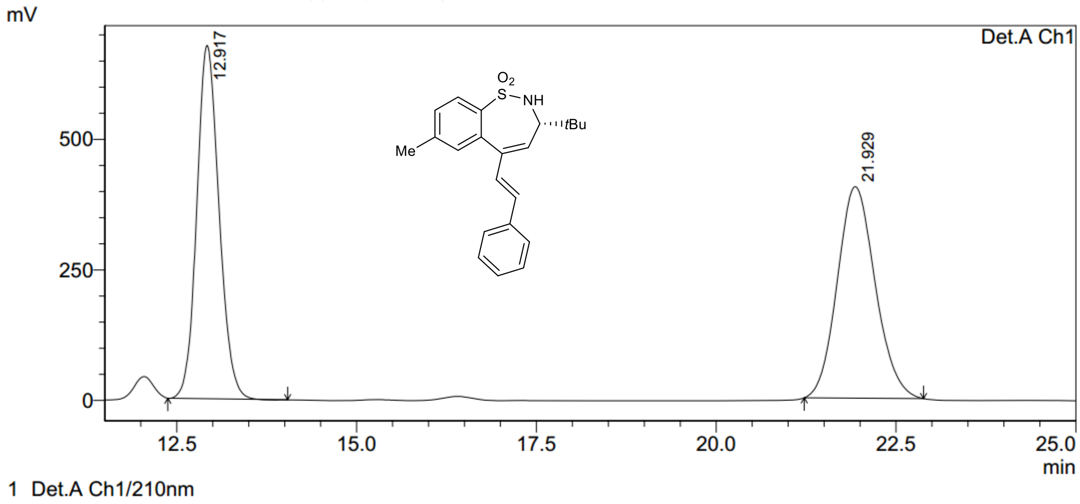

PeakTable

Detector A Ch1 210nm

Peak#	Ret. Time	Area	Height	Area %	Height %
1	12.917	14535988	676524	49.416	62.539
2	21.929	14879657	405238	50.584	37.461
Total		29415645	1081762	100.000	100.000

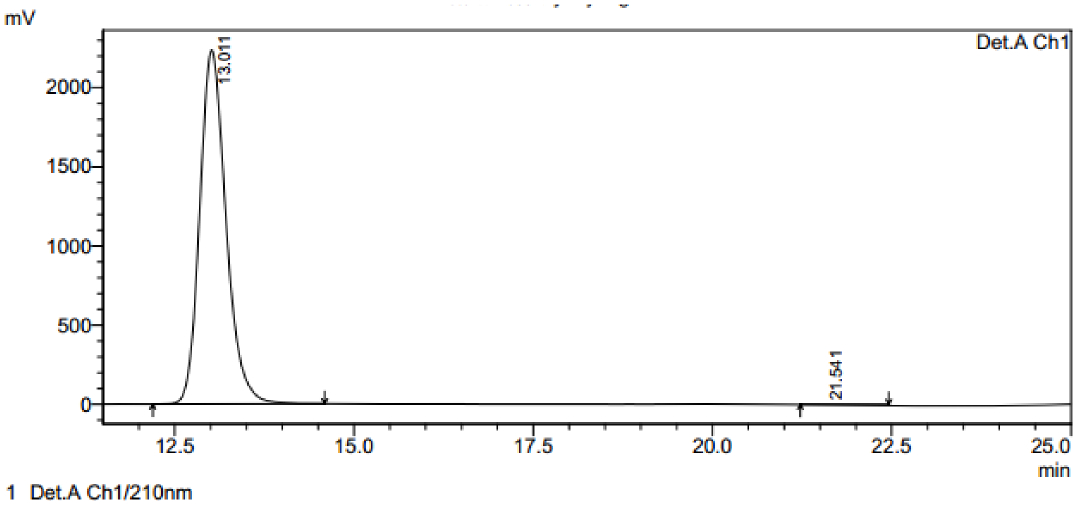

PeakTable

检测器 A Ch1 210nm

Peak#	Ret. Time	Area	Height	Area %	Height %
1	13.011	56911563	2234991	99.992	99.993
2	21.541	4837	167	0.008	0.007
Total		56916401	2235158	100.000	100.000

Supplementary Figure 244: HPLC traces for product **5q**

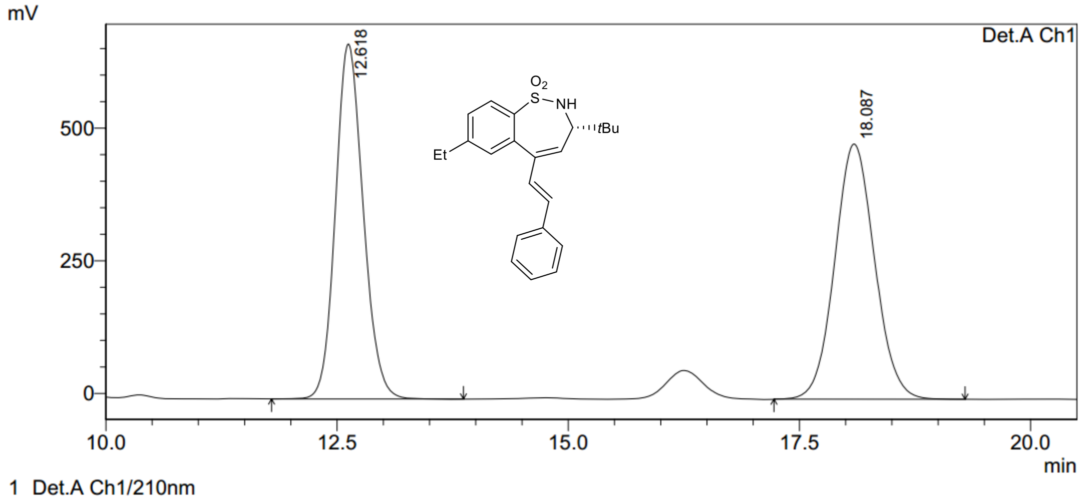

PeakTable

Detector A Ch1 210nm

Peak#	Ret. Time	Area	Height	Area %	Height %
1	12.618	13950824	669002	49.176	58.181
2	18.087	14418082	480870	50.824	41.819
Total		28368906	1149872	100.000	100.000

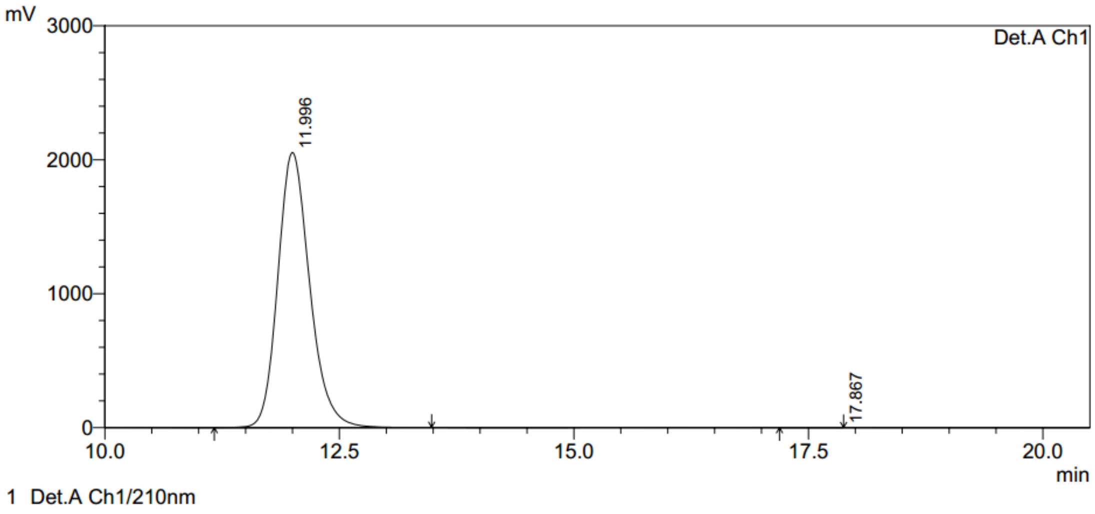

PeakTable

检测器 A Ch1 210nm

Peak#	Ret. Time	Area	Height	Area %	Height %
1	11.996	48394496	2053942	99.979	99.948
2	17.867	10167	1069	0.021	0.052
Total		48404663	2055011	100.000	100.000

Supplementary Figure 245: HPLC traces for product **5r**

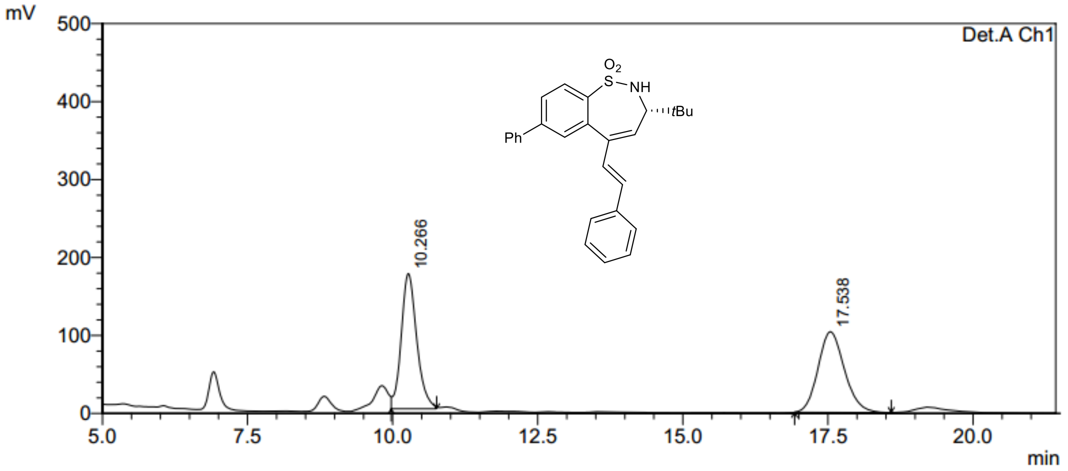

1 Det.A Ch1/210nm

PeakTable

Detector A Ch1 210nm

Peak#	Ret. Time	Area	Height	Area %	Height %
1	10.266	3213597	173269	49.619	62.637
2	17.538	3262956	103355	50.381	37.363
Total		6476554	276625	100.000	100.000

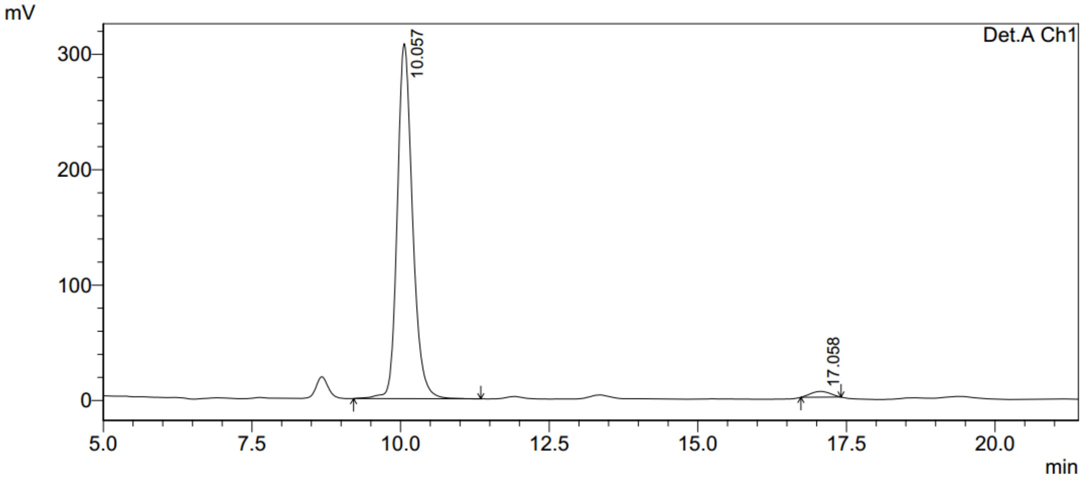

1 Det.A Ch1/210nm

PeakTable

Detector A Ch1 210nm

Peak#	Ret. Time	Area	Height	Area %	Height %
1	10.057	5495551	307691	97.942	98.389
2	17.058	115470	5038	2.058	1.611
Total		5611021	312729	100.000	100.000

Supplementary Figure 246: HPLC traces for product **5s**

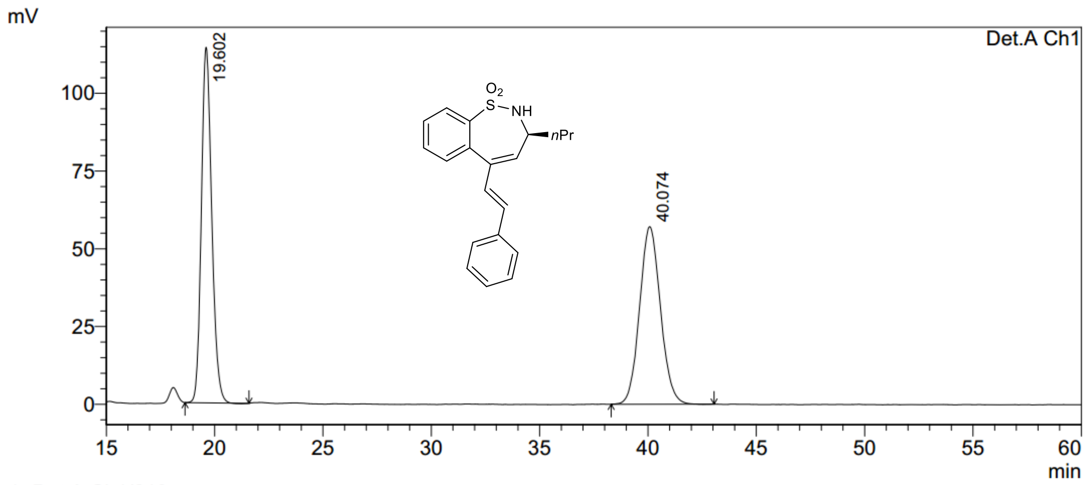

1 Det.A Ch1/210nm

PeakTable

检测器 A Ch1 210nm

Peak#	Ret. Time	Area	Height	Area %	Height %
1	19.602	3793793	114356	49.874	66.699
2	40.074	3813012	57095	50.126	33.301
Total		7606805	171451	100.000	100.000

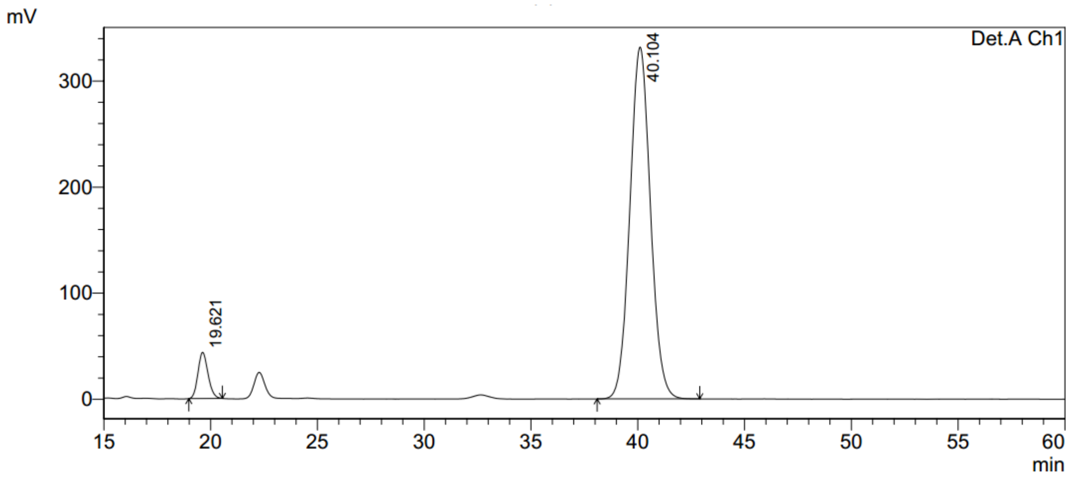

1 Det.A Ch1/210nm

PeakTable

检测器 A Ch1 210nm

Peak#	Ret. Time	Area	Height	Area %	Height %
1	19.621	1425663	43555	6.033	11.604
2	40.104	22205145	331773	93.967	88.396
Total		23630808	375328	100.000	100.000

Supplementary Figure 247: HPLC traces for product **6a**

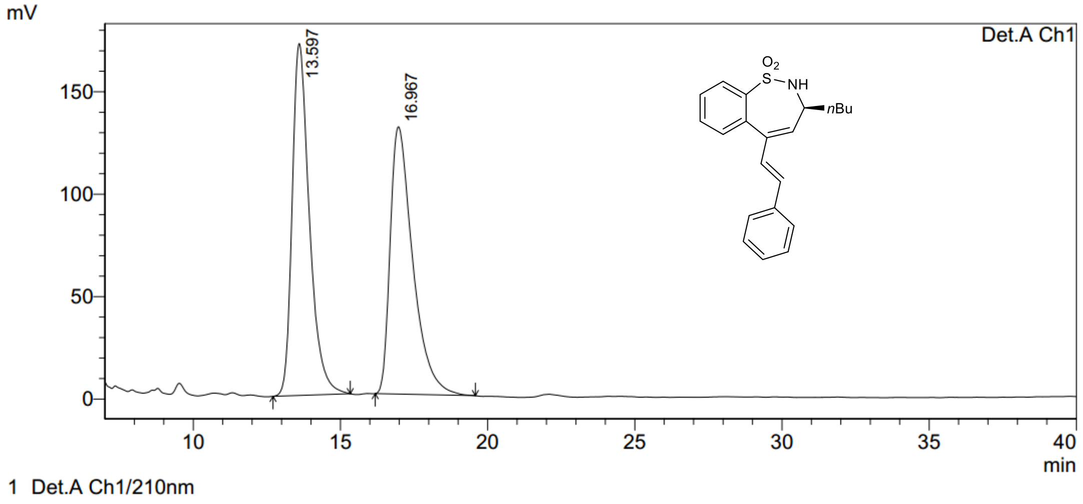

PeakTable

Detector A Ch1 210nm

Peak#	Ret. Time	Area	Height	Area %	Height %
1	13.597	6998362	171802	50.168	56.836
2	16.967	6951456	130473	49.832	43.164
Total		13949818	302275	100.000	100.000

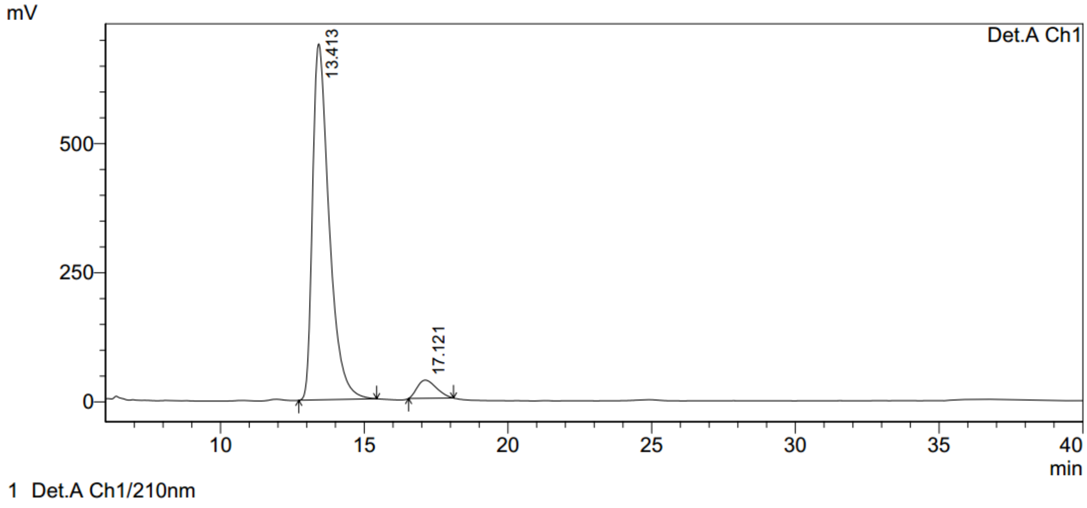

PeakTable

Detector A Ch1 210nm

Peak#	Ret. Time	Area	Height	Area %	Height %
1	13.413	27666502	689552	94.411	95.146
2	17.121	1637929	35175	5.589	4.854
Total		29304432	724728	100.000	100.000

Supplementary Figure 248: HPLC traces for product **6b**

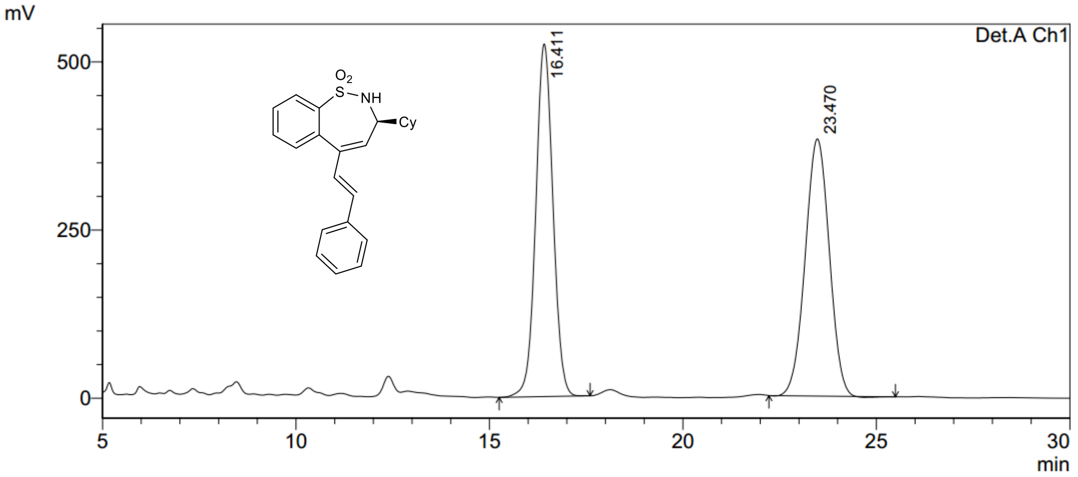

1 Det.A Ch1/210nm

PeakTable

Detector A Ch1 210nm

Peak#	Ret. Time	Area	Height	Area %	Height %
1	16.411	16184350	524396	49.510	57.834
2	23.470	16504383	382336	50.490	42.166
Total		32688732	906731	100.000	100.000

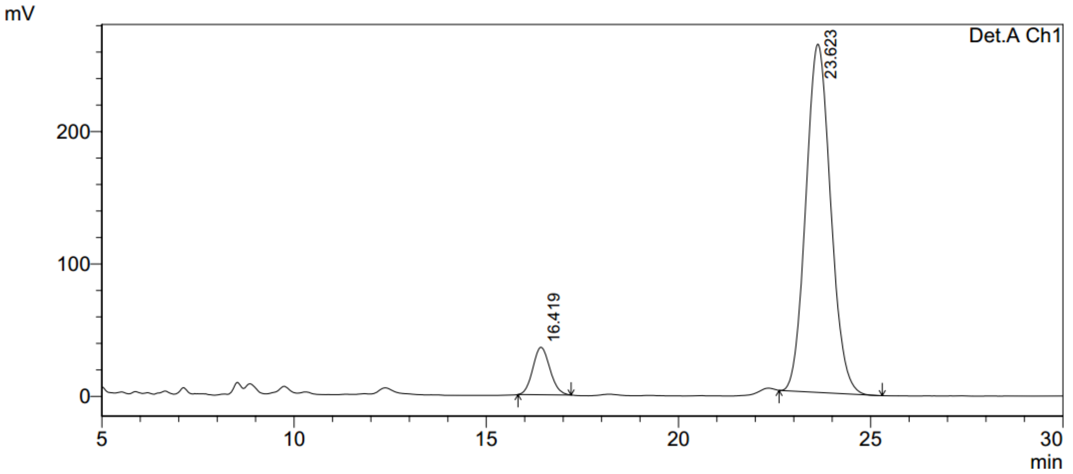

1 Det.A Ch1/210nm

PeakTable

检测器 A Ch1 210nm

Peak#	Ret. Time	Area	Height	Area %	Height %
1	16.419	1110319	35840	8.610	11.994
2	23.623	11785053	262987	91.390	88.006
Total		12895372	298827	100.000	100.000

Supplementary Figure 249: HPLC traces for product **6c**

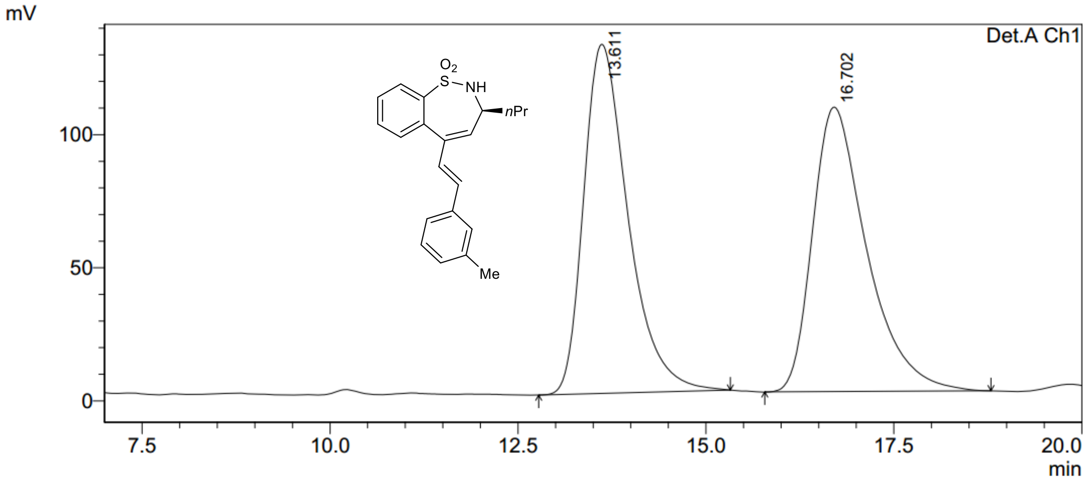

1 Det.A Ch1/210nm

PeakTable

Detector A Ch1 210nm

Peak#	Ret. Time	Area	Height	Area %	Height %
1	13.611	5396448	131171	50.150	55.104
2	16.702	5364135	106870	49.850	44.896
Total		10760584	238041	100.000	100.000

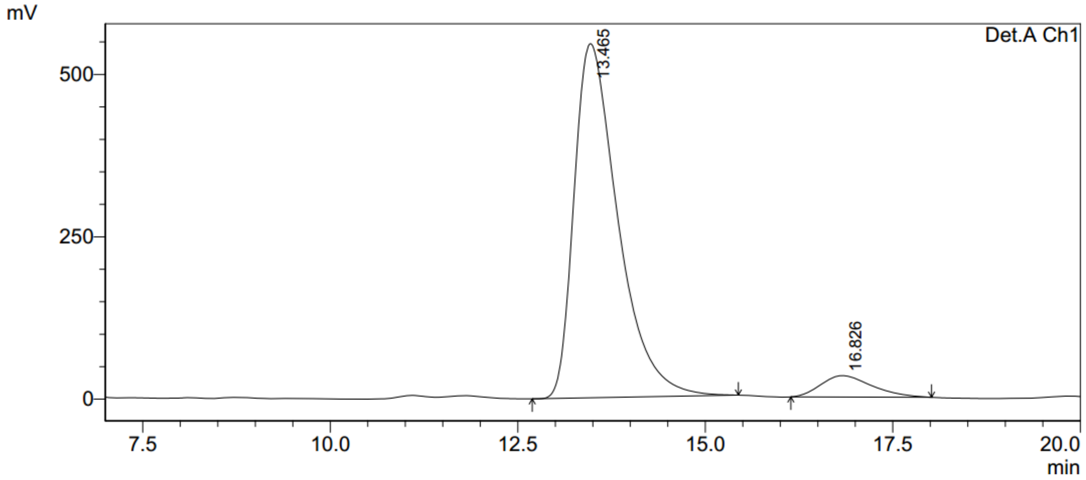

1 Det.A Ch1/210nm

PeakTable

Detector A Ch1 210nm

Peak#	Ret. Time	Area	Height	Area %	Height %
1	13.465	22174693	545309	93.421	94.293
2	16.826	1561600	33007	6.579	5.707
Total		23736294	578316	100.000	100.000

Supplementary Figure 250: HPLC traces for product **6d**

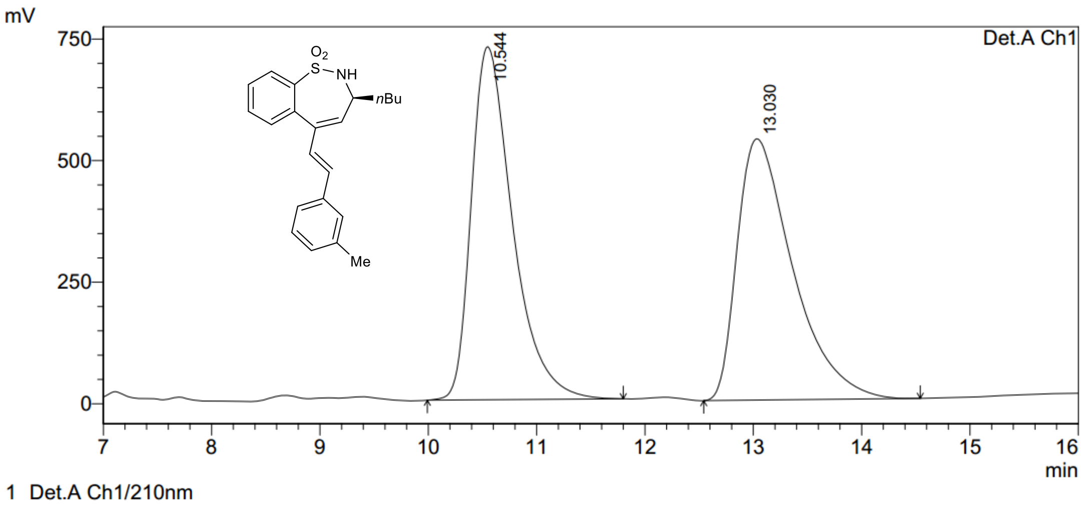

PeakTable

Detector A Ch1 210nm

Peak#	Ret. Time	Area	Height	Area %	Height %
1	10.544	19231124	726169	50.094	57.475
2	13.030	19158776	537291	49.906	42.525
Total		38389900	1263460	100.000	100.000

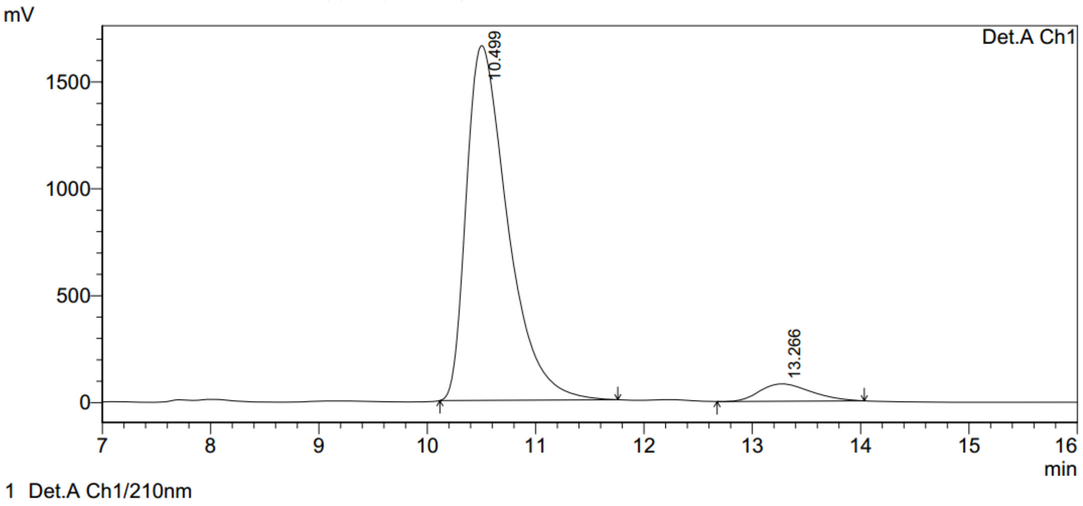

PeakTable

Detector A Ch1 210nm

Peak#	Ret. Time	Area	Height	Area %	Height %
1	10.499	44041623	1659616	94.247	95.314
2	13.266	2688566	81591	5.753	4.686
Total		46730189	1741206	100.000	100.000

Supplementary Figure 251: HPLC traces for product **6e**

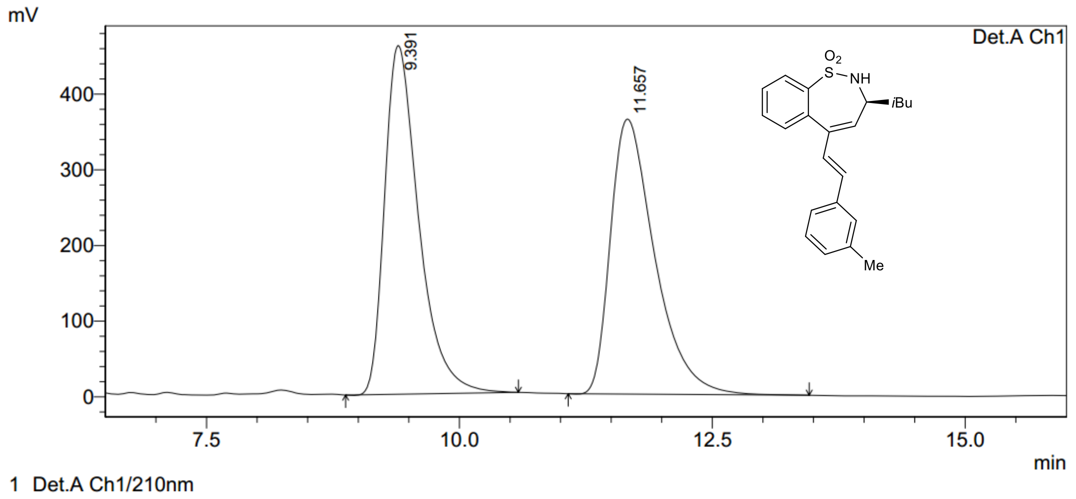

PeakTable

Detector A Ch1 210nm

Peak#	Ret. Time	Area	Height	Area %	Height %
1	9.391	10807506	460829	49.454	55.910
2	11.657	11046360	363410	50.546	44.090
Total		21853866	824239	100.000	100.000

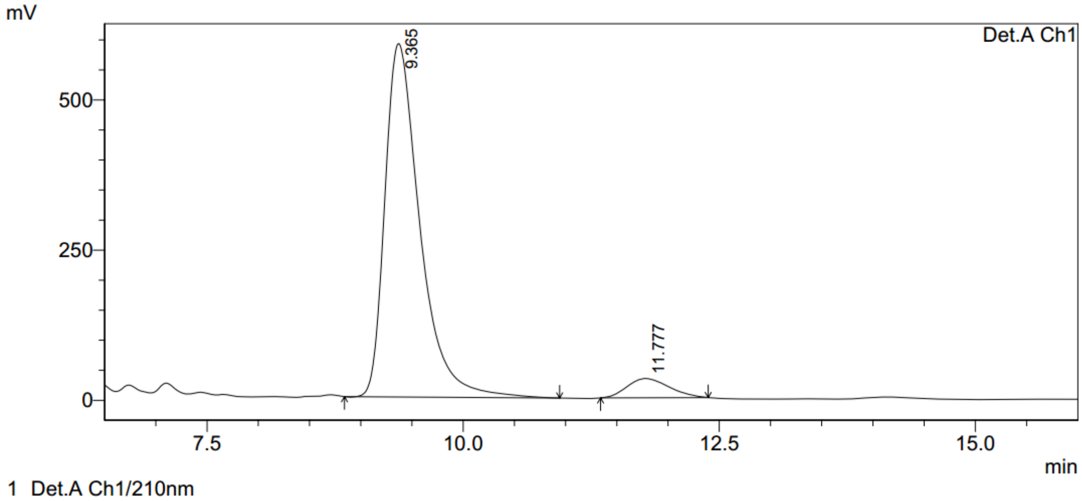

PeakTable

Detector A Ch1 210nm

Peak#	Ret. Time	Area	Height	Area %	Height %
1	9.365	13987137	588297	93.845	94.825
2	11.777	917351	32106	6.155	5.175
Total		14904488	620403	100.000	100.000

Supplementary Figure 252: HPLC traces for product **6f**

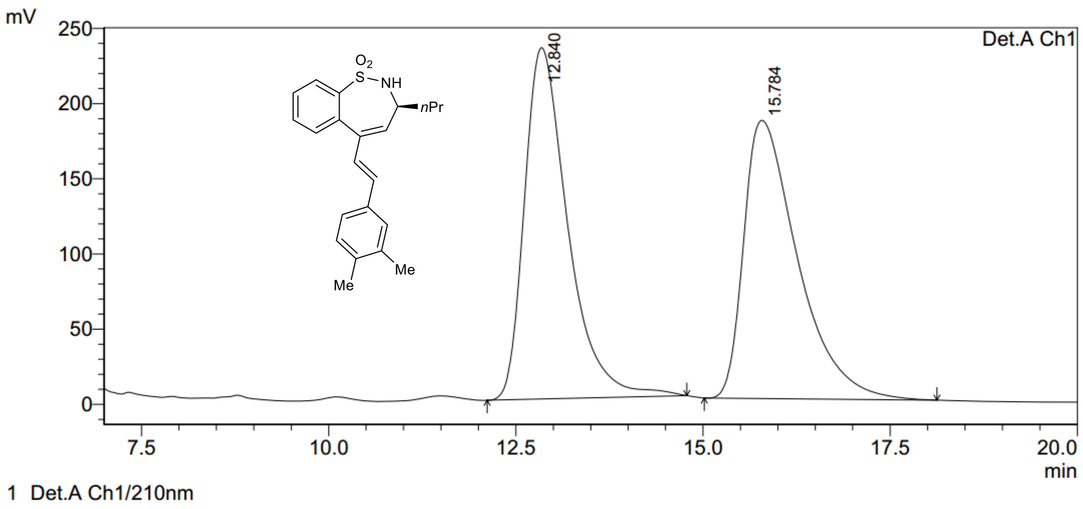

PeakTable

Detector A Ch1 210nm

Peak#	Ret. Time	Area	Height	Area %	Height %
1	12.840	9421717	233629	50.418	55.811
2	15.784	9265424	184977	49.582	44.189
Total		18687142	418607	100.000	100.000

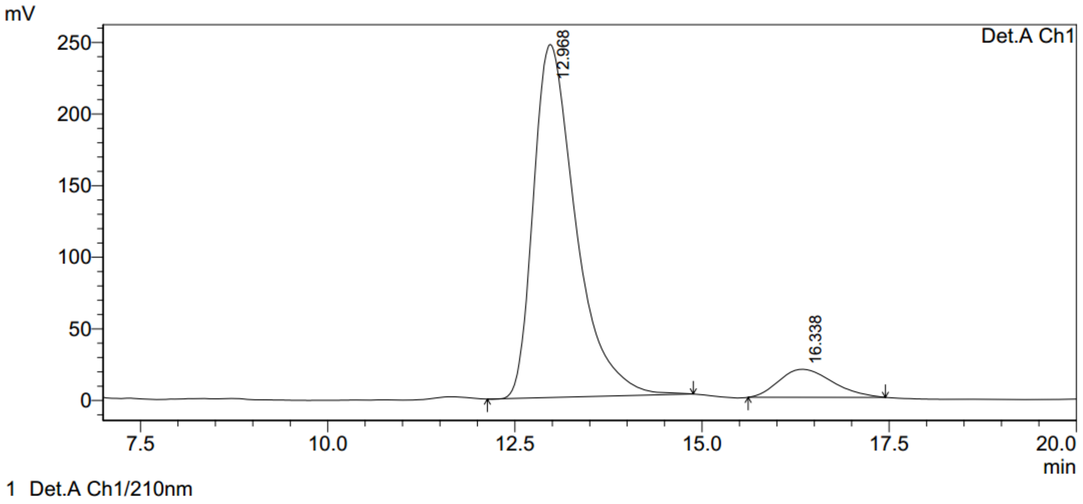

PeakTable

Detector A Ch1 210nm

Peak#	Ret. Time	Area	Height	Area %	Height %
1	12.968	9707468	246468	90.829	92.599
2	16.338	980162	19699	9.171	7.401
Total		10687630	266167	100.000	100.000

Supplementary Figure 253: HPLC traces for product **6g**

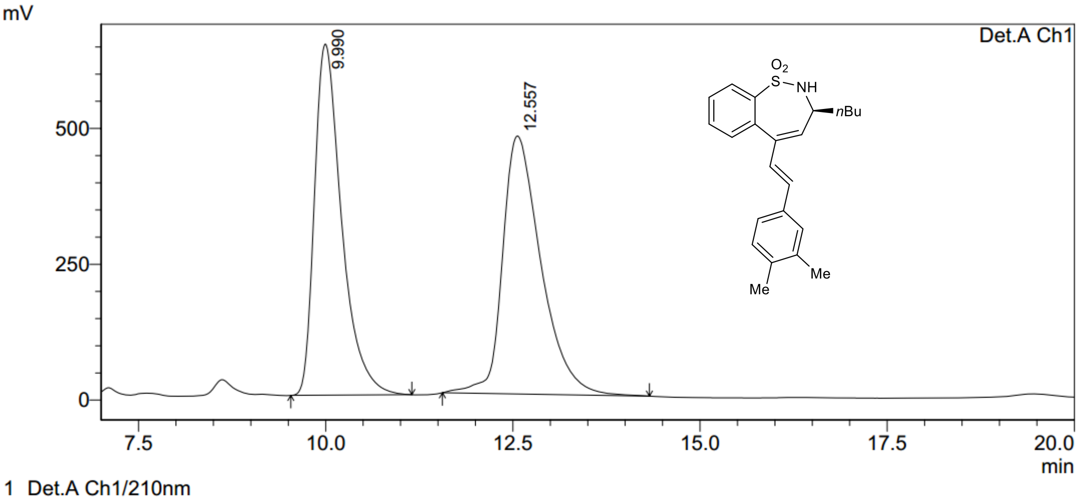

PeakTable

Detector A Ch1 210nm

Peak#	Ret. Time	Area	Height	Area %	Height %
1	9.990	16283414	646333	48.938	57.645
2	12.557	16990363	474902	51.062	42.355
Total		33273778	1121235	100.000	100.000

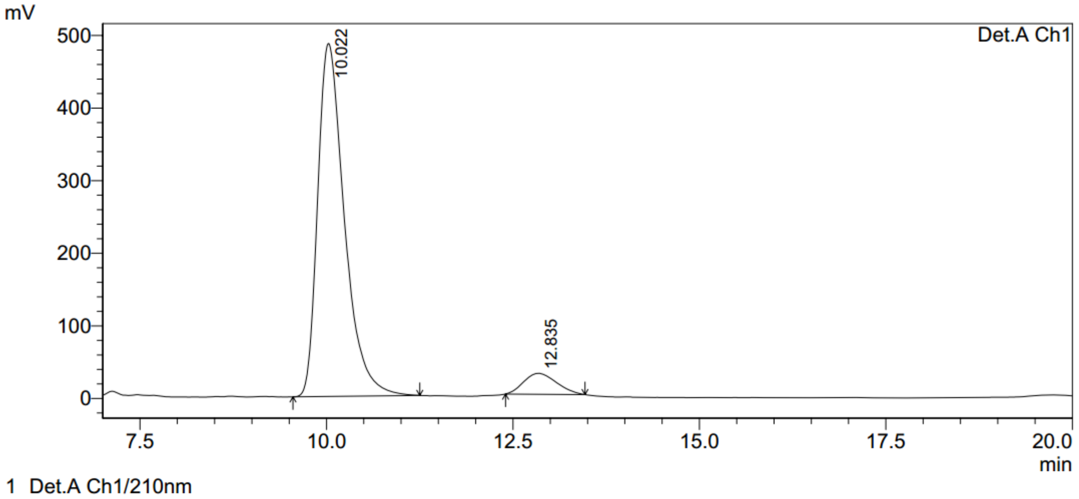

PeakTable

Detector A Ch1 210nm

Peak#	Ret. Time	Area	Height	Area %	Height %
1	10.022	12103795	486354	93.204	94.414
2	12.835	882609	28777	6.796	5.586
Total		12986404	515131	100.000	100.000

Supplementary Figure 254: HPLC traces for product **6h**

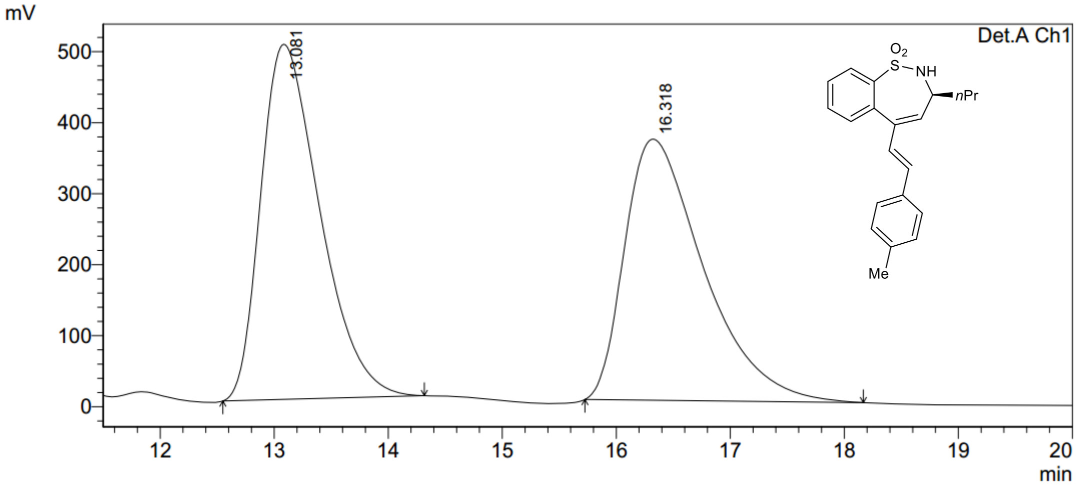

1 Det.A Ch1/210nm

PeakTable

检测器 A Ch1 210nm

Peak#	Ret. Time	Area	Height	Area %	Height %
1	13.081	18007316	500109	50.477	57.625
2	16.318	17667038	367765	49.523	42.375
Total		35674354	867874	100.000	100.000

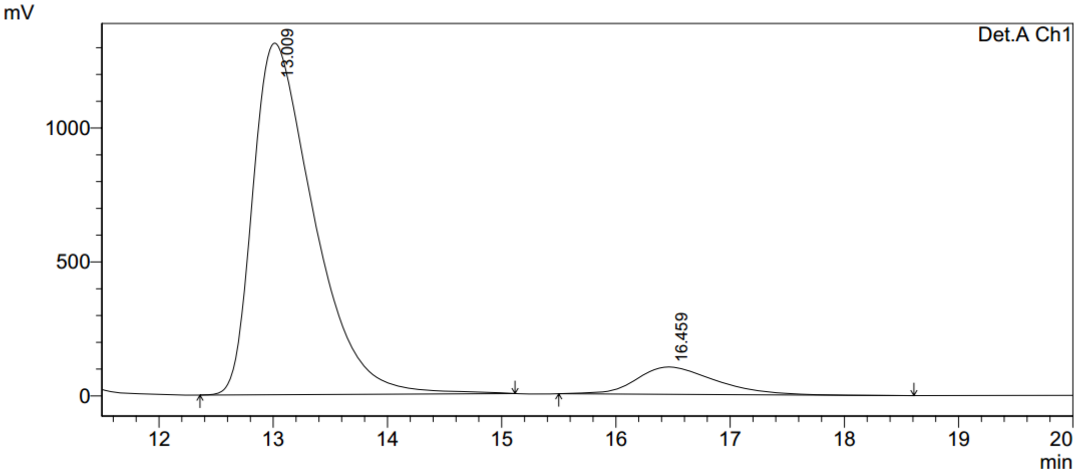

1 Det.A Ch1/210nm

PeakTable

检测器 A Ch1 210nm

Peak#	Ret. Time	Area	Height	Area %	Height %
1	13.009	48629446	1312258	90.823	92.799
2	16.459	4913518	101826	9.177	7.201
Total		53542964	1414084	100.000	100.000

Supplementary Figure 255: HPLC traces for product **6i**

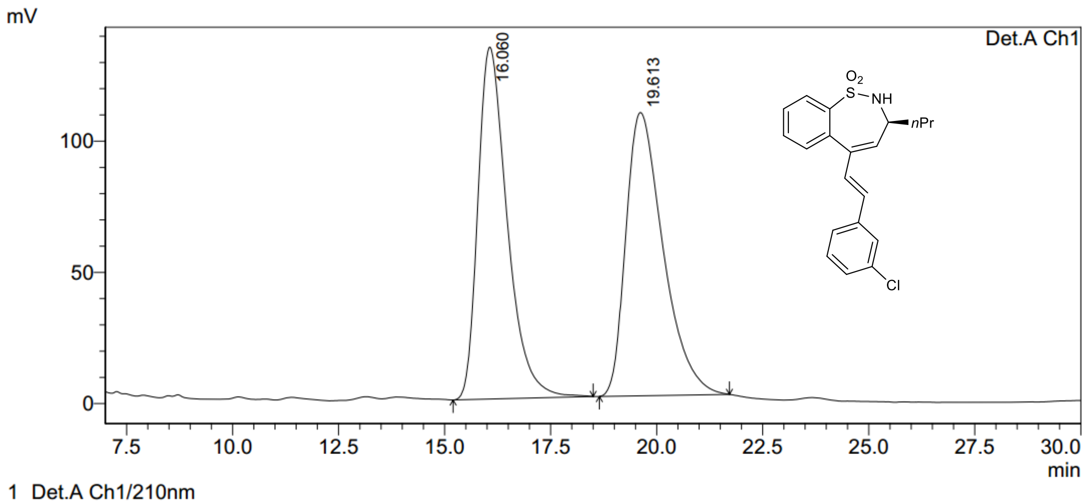

1 Det.A Ch1/210nm

PeakTable

Detector A Ch1 210nm

Peak#	Ret. Time	Area	Height	Area %	Height %
1	16.060	6446708	134115	49.396	55.400
2	19.613	6604250	107971	50.604	44.600
Total		13050958	242086	100.000	100.000

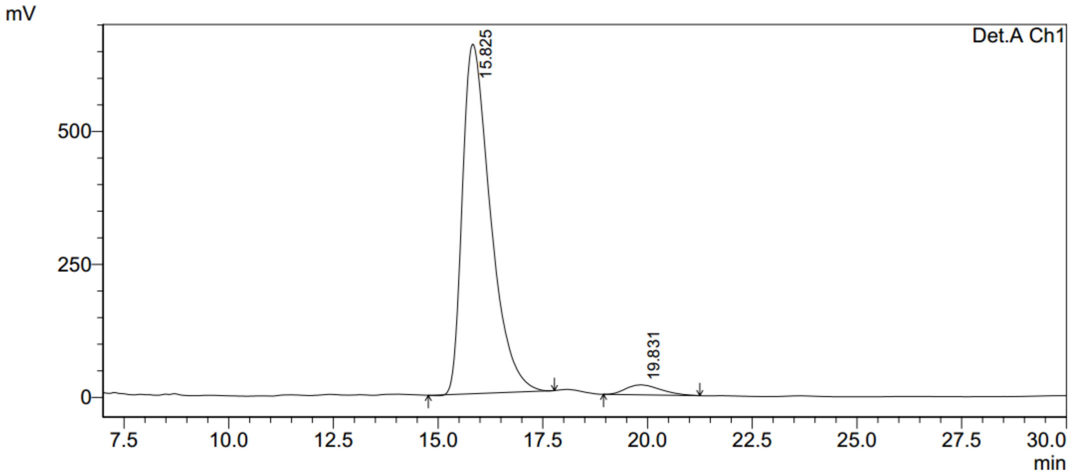

1 Det.A Ch1/210nm

PeakTable

Detector A Ch1 210nm

Peak#	Ret. Time	Area	Height	Area %	Height %
1	15.825	30554909	656984	96.516	97.218
2	19.831	1102880	18800	3.484	2.782
Total		31657789	675785	100.000	100.000

Supplementary Figure 256: HPLC traces for product **6j**

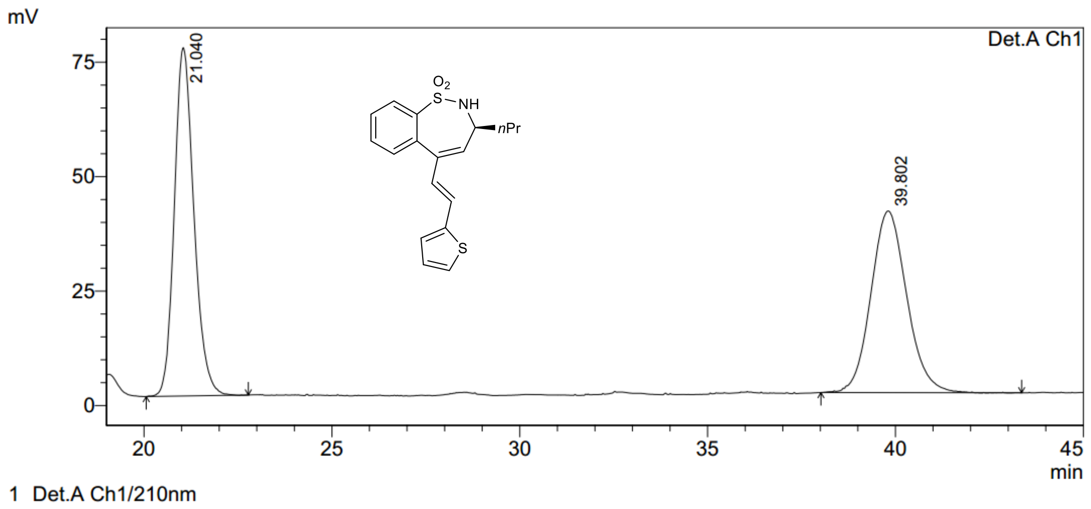

PeakTable

检测器 A Ch1 210nm

Peak#	Ret. Time	Area	Height	Area %	Height %
1	21.040	2756068	76087	51.158	65.722
2	39.802	2631344	39684	48.842	34.278
Total		5387412	115771	100.000	100.000

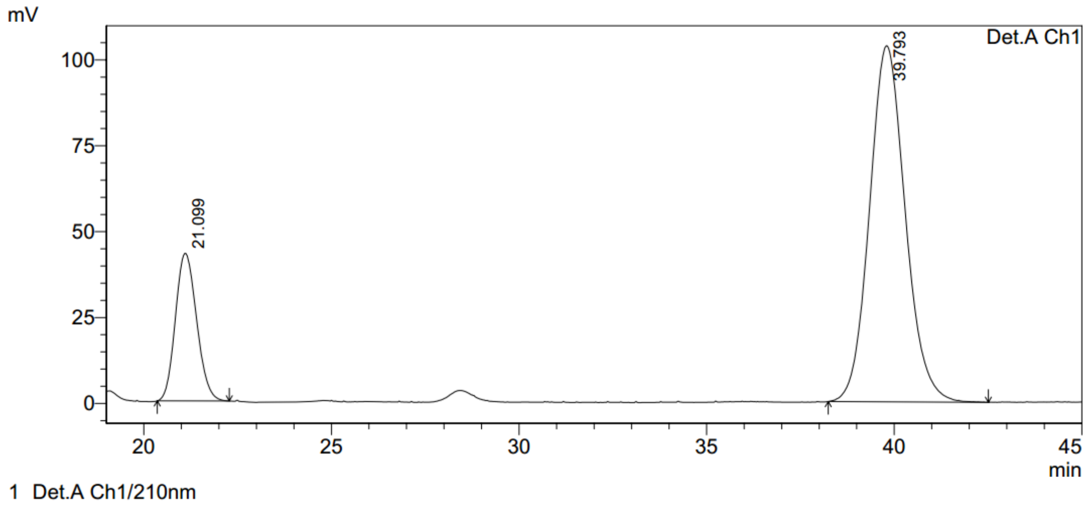

PeakTable

检测器 A Ch1 210nm

Peak#	Ret. Time	Area	Height	Area %	Height %
1	21.099	1690462	42991	19.751	29.314
2	39.793	6868463	103665	80.249	70.686
Total		8558925	146656	100.000	100.000

Supplementary Figure 257: HPLC traces for product **6k**

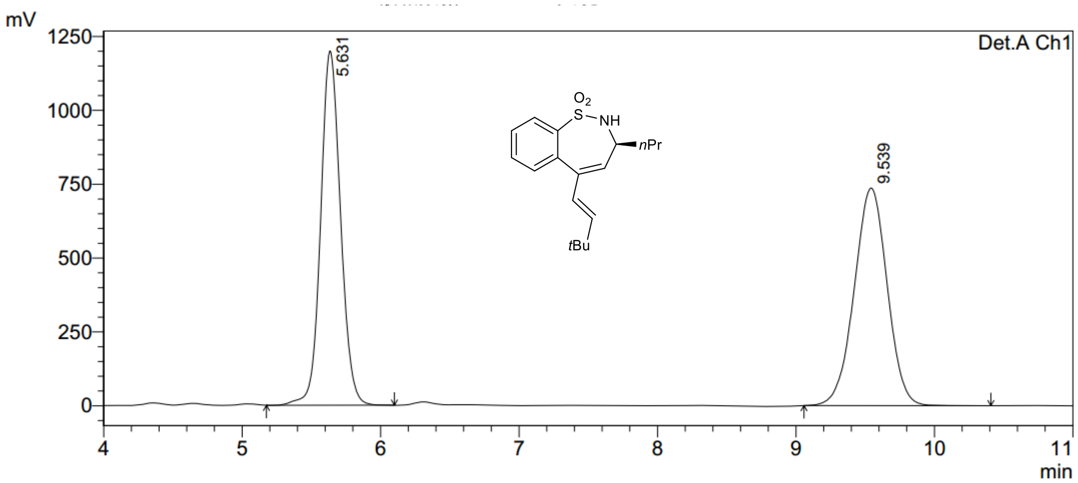

1 Det.A Ch1/210nm

PeakTable

Detector A Ch1 210nm

Peak#	Ret. Time	Area	Height	Area %	Height %
1	5.631	11983517	1199672	49.579	61.955
2	9.539	12186865	736685	50.421	38.045
Total		24170382	1936357	100.000	100.000

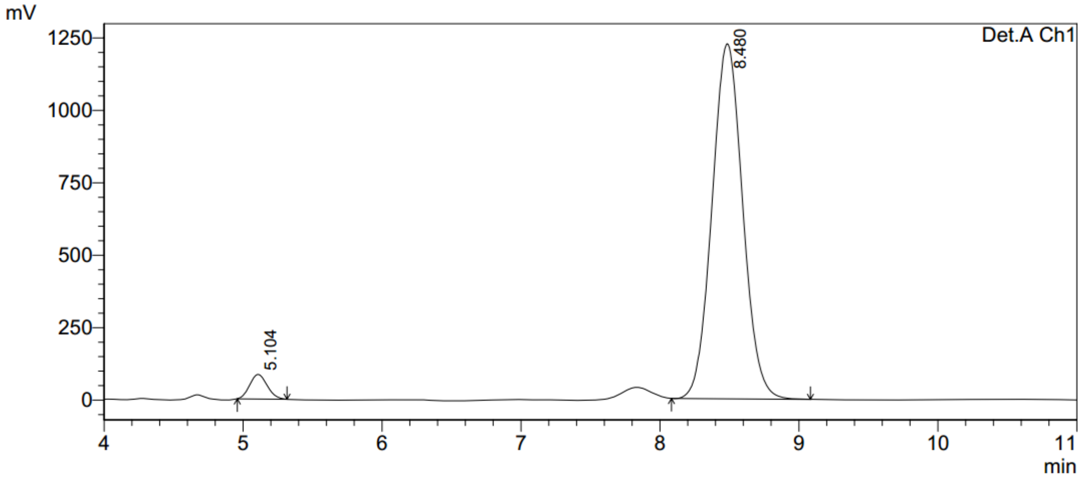

1 Det.A Ch1/210nm

PeakTable

Detector A Ch1 210nm

Peak#	Ret. Time	Area	Height	Area %	Height %
1	5.104	744347	84903	3.804	6.476
2	8.480	18823329	1226079	96.196	93.524
Total		19567676	1310983	100.000	100.000

Supplementary Figure 258: HPLC traces for product **6l**

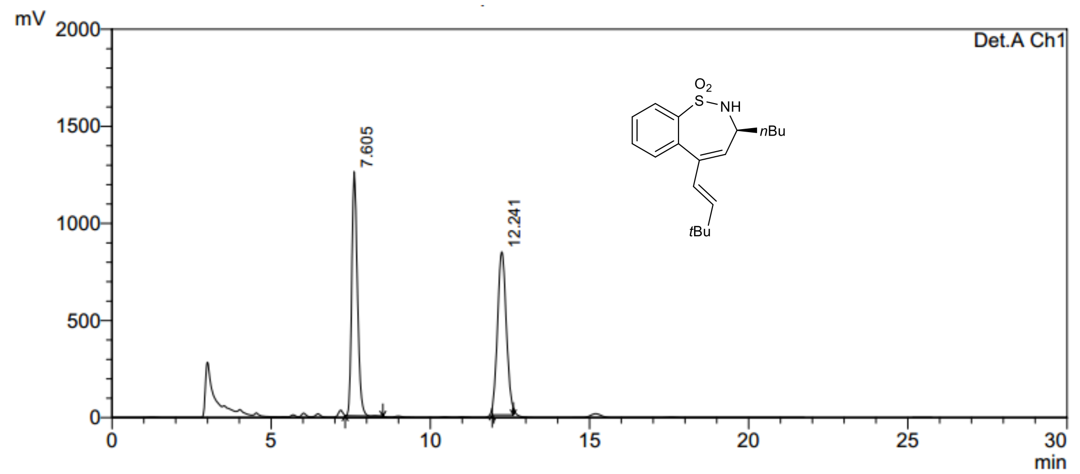

1 Det.A Ch1/210nm

PeakTable

Detector A Ch1 210nm

Peak#	Ret. Time	Area	Height	Area %	Height %
1	7.605	15535631	1257969	49.068	59.985
2	12.241	16125828	839187	50.932	40.015
Total		31661459	2097156	100.000	100.000

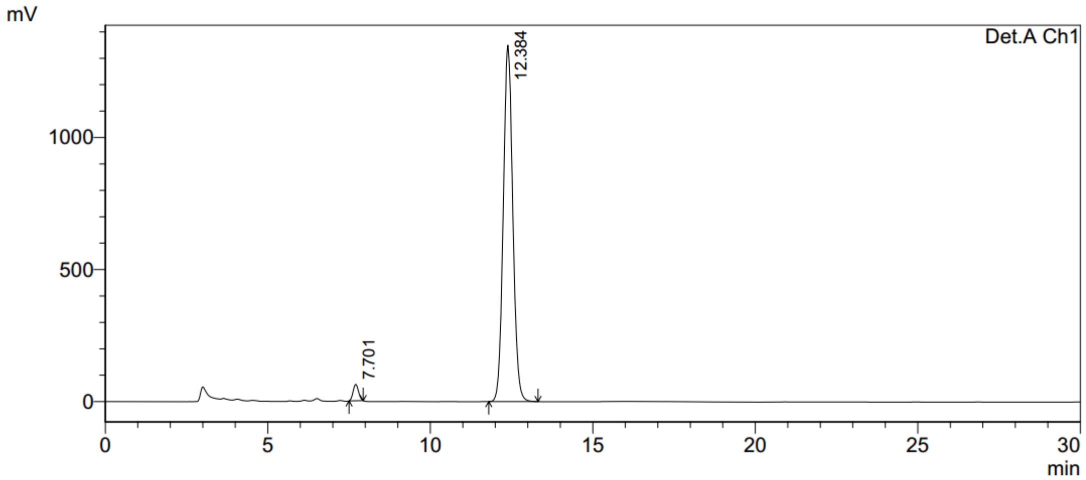

1 Det.A Ch1/210nm

PeakTable

Detector A Ch1 210nm

Peak#	Ret. Time	Area	Height	Area %	Height %
1	7.701	714773	61548	2.538	4.362
2	12.384	27447245	1349556	97.462	95.638
Total		28162018	1411104	100.000	100.000

Supplementary Figure 259: HPLC traces for product **6m**

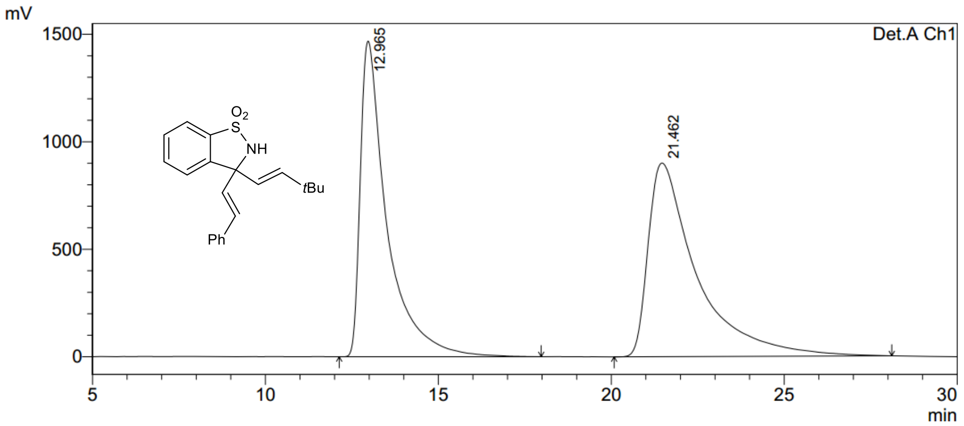

1 Det.A Ch1/210nm

PeakTable

Detector A Ch1 210nm

Peak#	Ret. Time	Area	Height	Area %	Height %
1	12.965	78388399	1468211	47.097	61.963
2	21.462	88053042	901287	52.903	38.037
Total		166441441	2369499	100.000	100.000

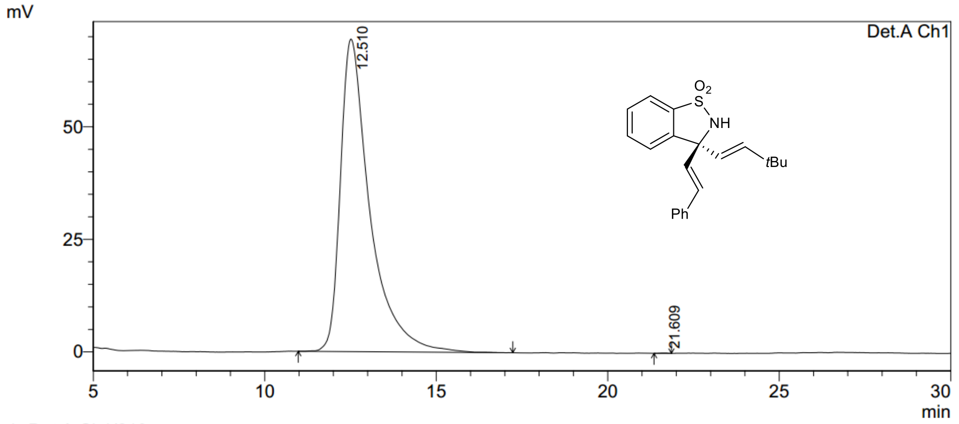

1 Det.A Ch1/210nm

PeakTable

检测器 A Ch1 210nm

Peak#	Ret. Time	Area	Height	Area %	Height %
1	12.510	4150153	69410	99.977	99.912
2	21.609	953	61	0.023	0.088
Total		4151106	69472	100.000	100.000

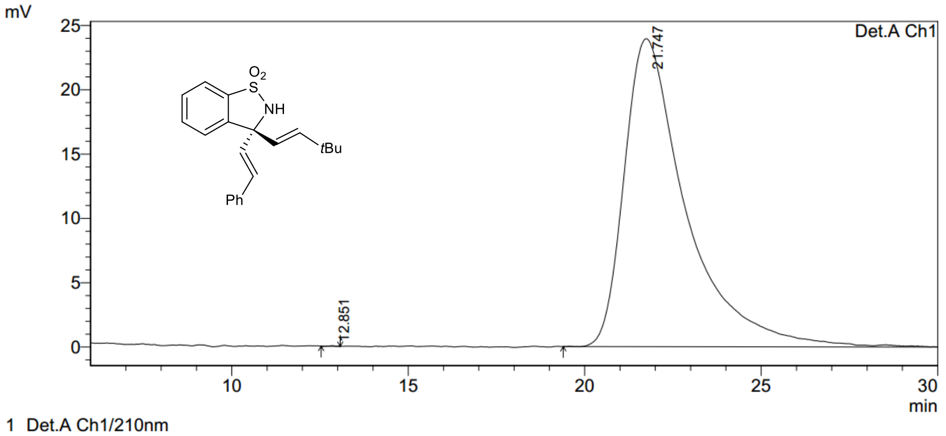

1 Det.A Ch1/210nm

PeakTable

检测器 A Ch1 210nm

Peak#	Ret. Time	Area	Height	Area %	Height %
1	12.851	460	52	0.016	0.218
2	21.747	2915015	23935	99.984	99.782
Total		2915474	23988	100.000	100.000

Supplementary Figure 260: HPLC traces for product **7**

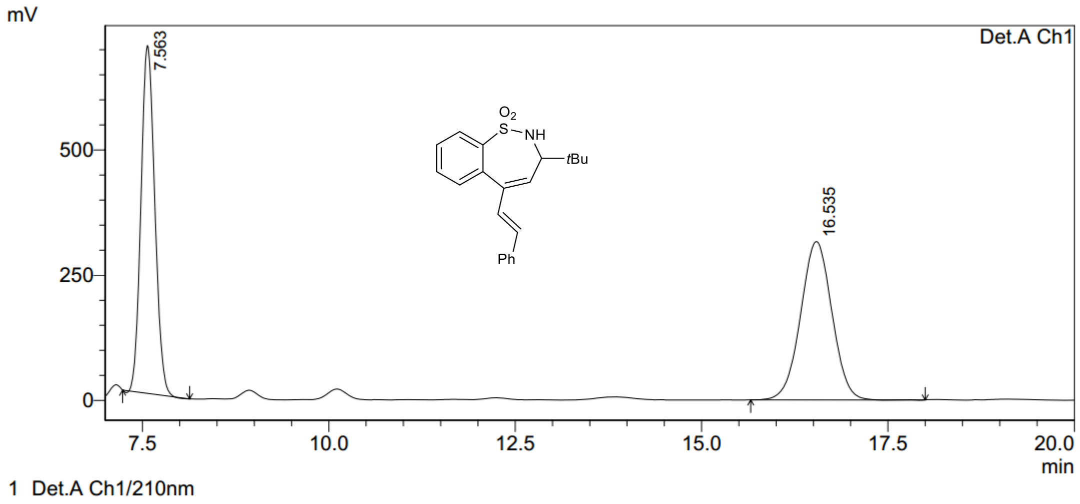

PeakTable

Detector A Ch1 210nm

Peak#	Ret. Time	Area	Height	Area %	Height %
1	7.563	8905608	694354	49.054	68.711
2	16.535	9249004	316182	50.946	31.289
Total		18154612	1010536	100.000	100.000

Supplementary Figure 261: HPLC traces for product ***rac*-5a**

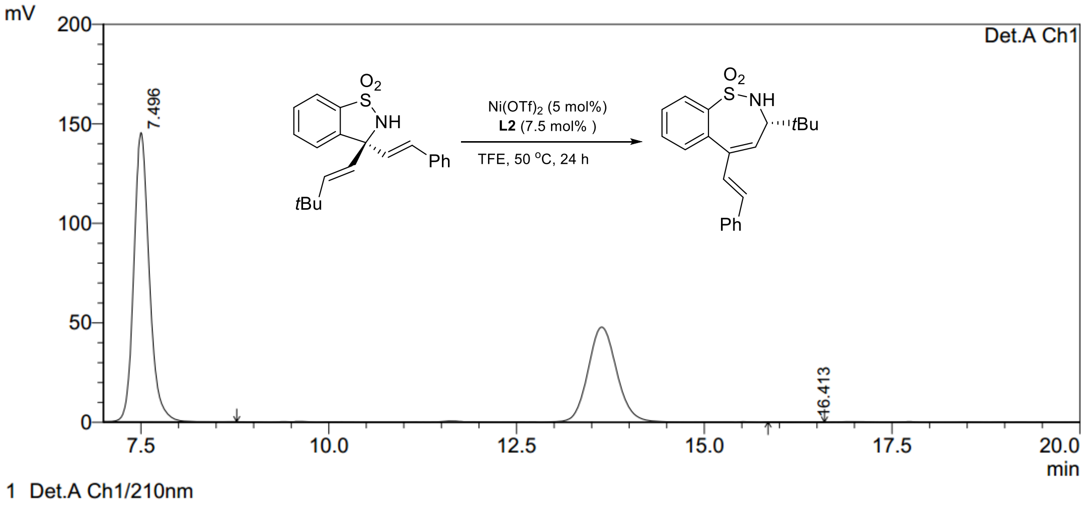

PeakTable

检测器 A Ch1 210nm

Peak#	Ret. Time	Area	Height	Area %	Height %
1	7.496	2071498	145266	99.961	99.869
2	16.413	804	190	0.039	0.131
Total		2072302	145456	100.000	100.000

Supplementary Figure 262: HPLC traces for control experiment with Ni-catalyst

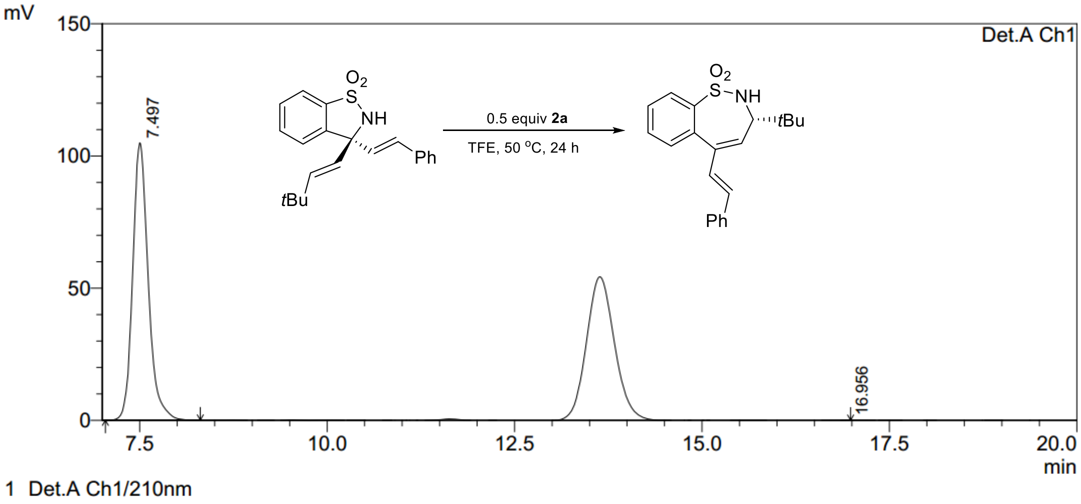

PeakTable

检测器 A Ch1 210nm

Peak#	Ret. Time	Area	Height	Area %	Height %
1	7.497	1506955	104888	99.929	99.973
2	16.956	1074	28	0.071	0.027
Total		1508030	104916	100.000	100.000

Supplementary Figure 263: HPLC traces for control experiment with alkenylboronic acid **2a**

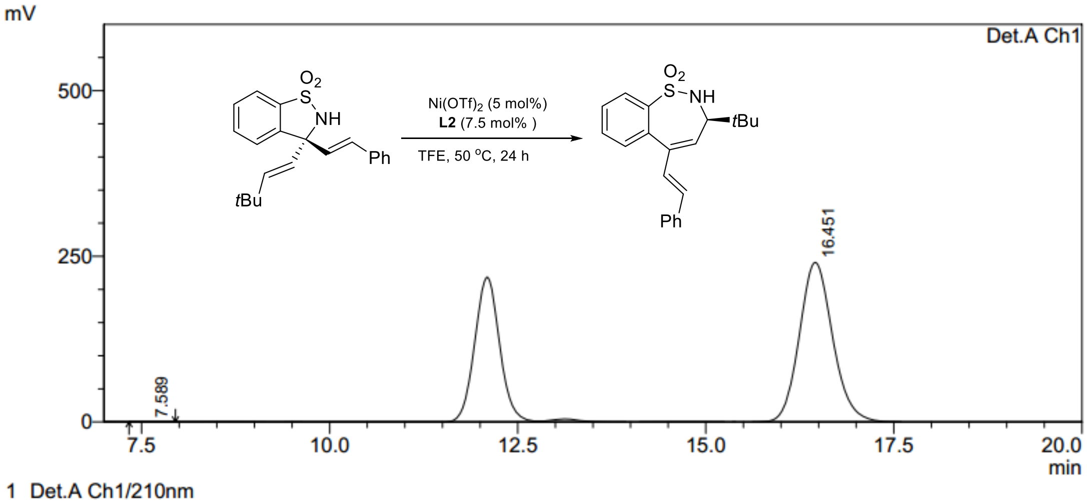

PeakTable

Detector A Ch1 210nm

Peak#	Ret. Time	Area	Height	Area %	Height %
1	7.589	2561	146	0.034	0.061
2	16.451	7471565	241166	99.966	99.939
Total		7474125	241312	100.000	100.000

Supplementary Figure 264: HPLC traces for match/mismatch experiment with Ni-catalyst

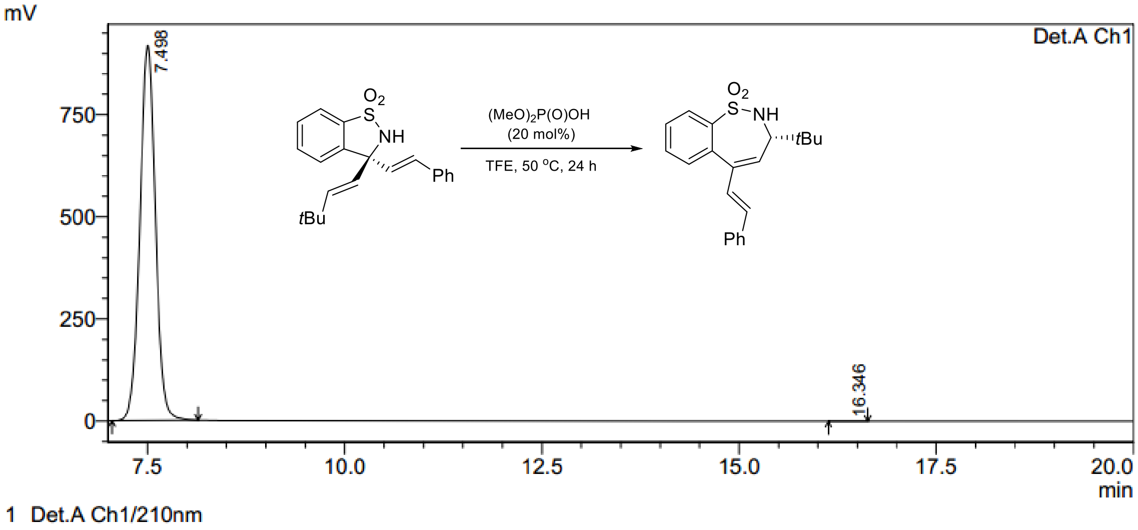

PeakTable

检测器 A Ch1 210nm

Peak#	Ret. Time	Area	Height	Area %	Height %
1	7.498	12571732	918139	99.992	99.993
2	16.346	1027	64	0.008	0.007
Total		12572759	918203	100.000	100.000

Supplementary Figure 265: HPLC traces for control experiment with $(MeO)_2P(O)OH$

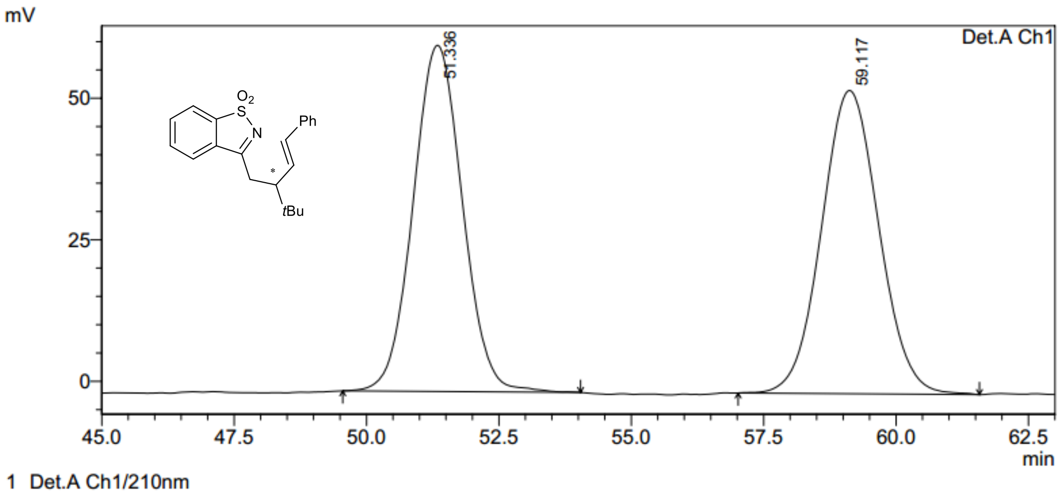

PeakTable

检测器 A Ch1 210nm

Peak#	Ret. Time	Area	Height	Area %	Height %
1	51.336	3962956	61145	49.499	53.303
2	59.117	4043208	53567	50.501	46.697
Total		8006164	114712	100.000	100.000

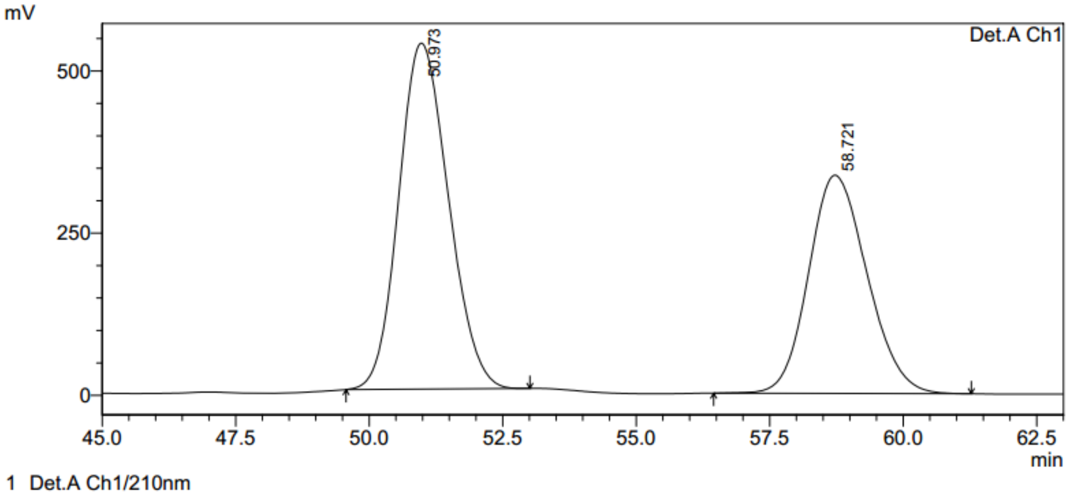

PeakTable

检测器 A Ch1 210nm

Peak#	Ret. Time	Area	Height	Area %	Height %
1	50.973	34518167	533253	57.347	61.314
2	58.721	25673487	336459	42.653	38.686
Total		60191654	869712	100.000	100.000

Supplementary Figure 266: HPLC traces for product **9**

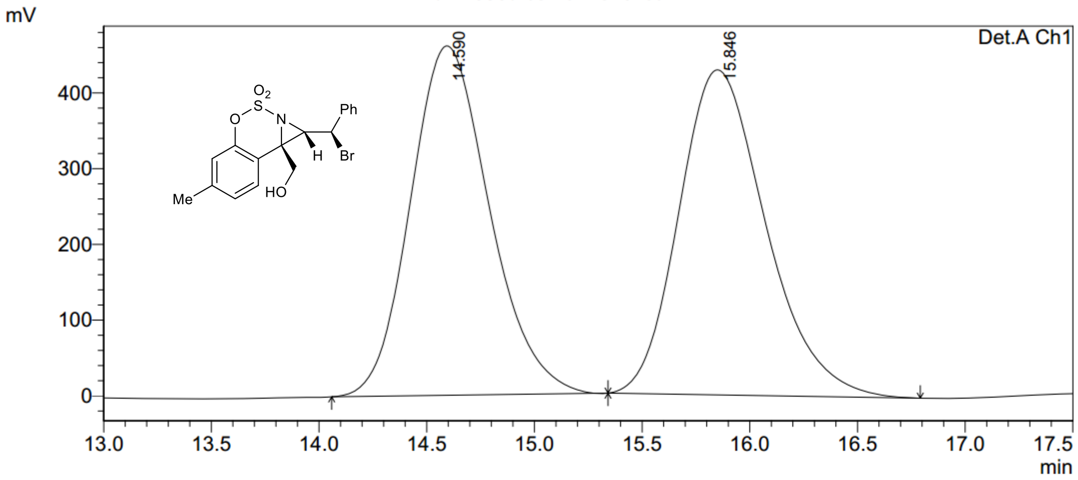

1 Det.A Ch1/210nm

PeakTable

检测器 A Ch1 210nm

Peak#	Ret. Time	Area	Height	Area %	Height %
1	14.590	11751865	461416	49.396	51.805
2	15.846	12039318	429271	50.604	48.195
Total		23791183	890687	100.000	100.000

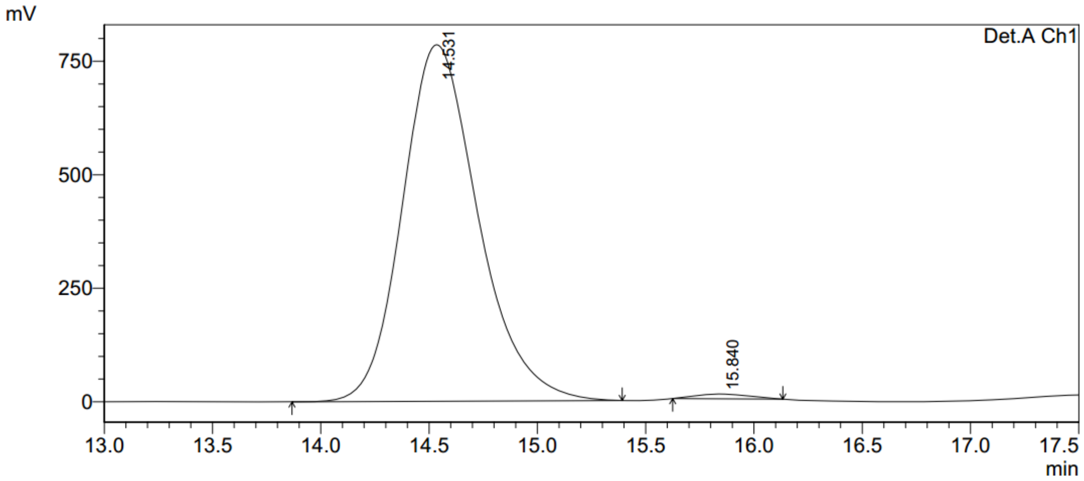

1 Det.A Ch1/210nm

PeakTable

检测器 A Ch1 210nm

Peak#	Ret. Time	Area	Height	Area %	Height %
1	14.531	19108278	785227	99.050	98.692
2	15.840	183315	10409	0.950	1.308
Total		19291592	795636	100.000	100.000

Supplementary Figure 267: HPLC traces for product **11**

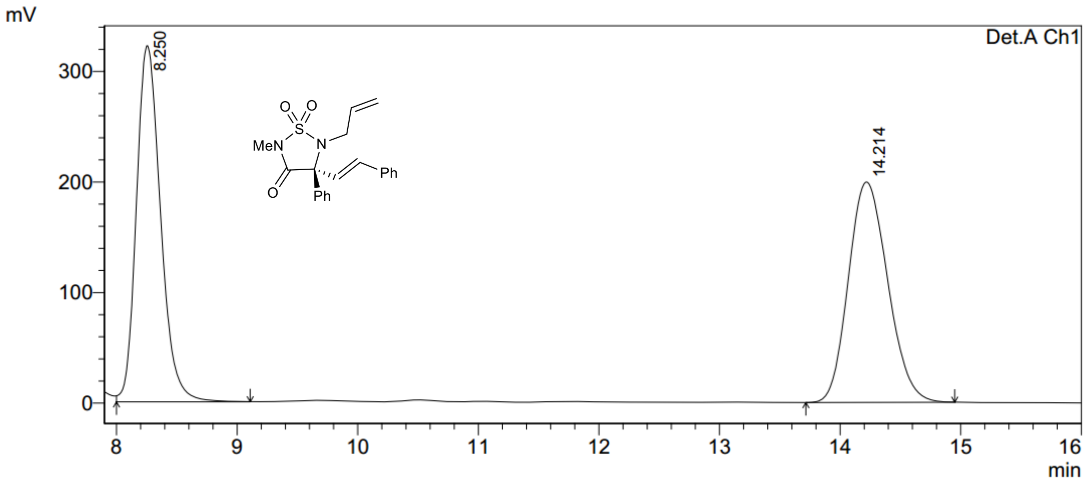

1 Det.A Ch1/210nm

PeakTable

检测器 A Ch1 210nm

Peak#	Ret. Time	Area	Height	Area %	Height %
1	8.250	4488789	321988	49.825	61.759
2	14.214	4520271	199370	50.175	38.241
Total		9009061	521359	100.000	100.000

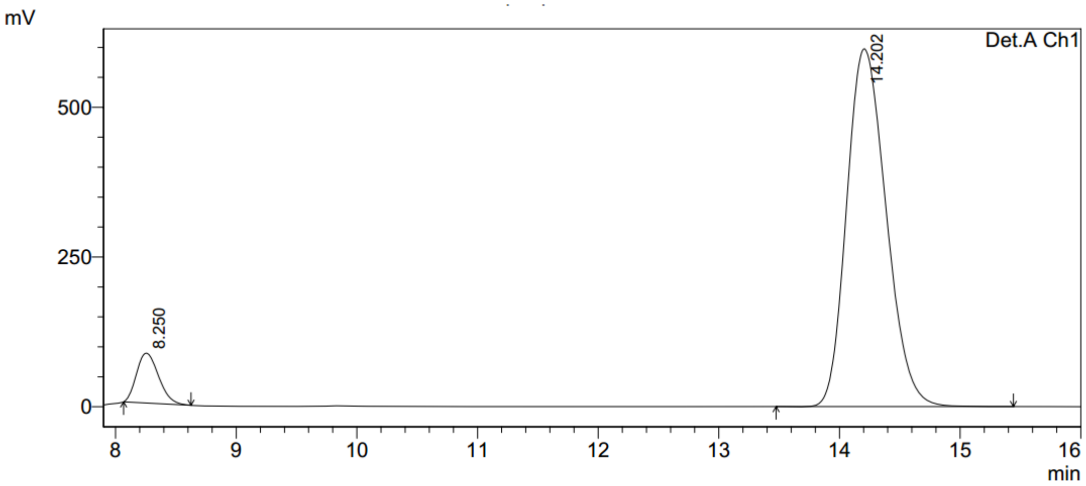

1 Det.A Ch1/210nm

PeakTable

检测器 A Ch1 210nm

Peak#	Ret. Time	Area	Height	Area %	Height %
1	8.250	1057025	83115	7.227	12.214
2	14.202	13568413	597371	92.773	87.786
Total		14625438	680486	100.000	100.000

Supplementary Figure 268: HPLC traces for product **12**

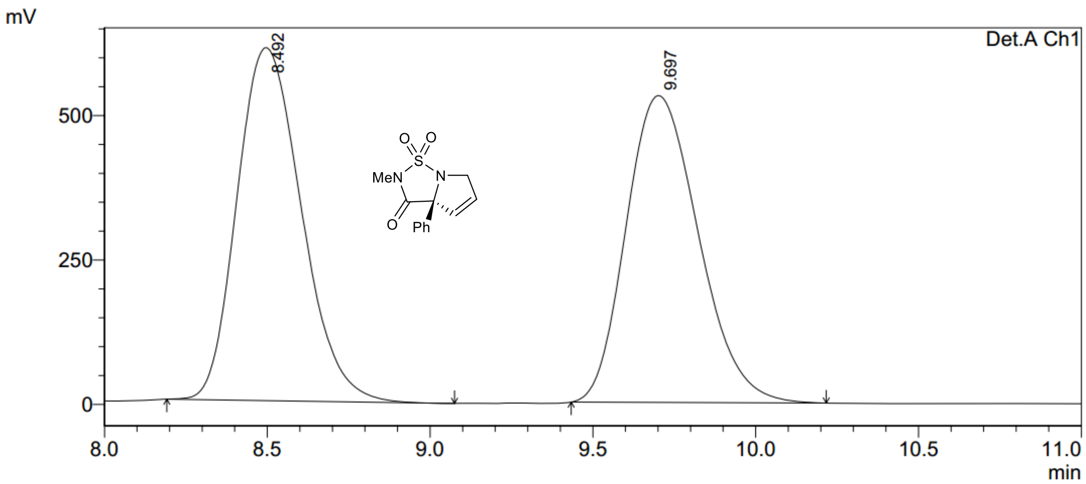

1 Det.A Ch1/210nm

PeakTable

检测器 A Ch1 210nm

Peak#	Ret. Time	Area	Height	Area %	Height %
1	8.492	8406530	610784	50.589	53.476
2	9.697	8210665	531390	49.411	46.524
Total		16617194	1142174	100.000	100.000

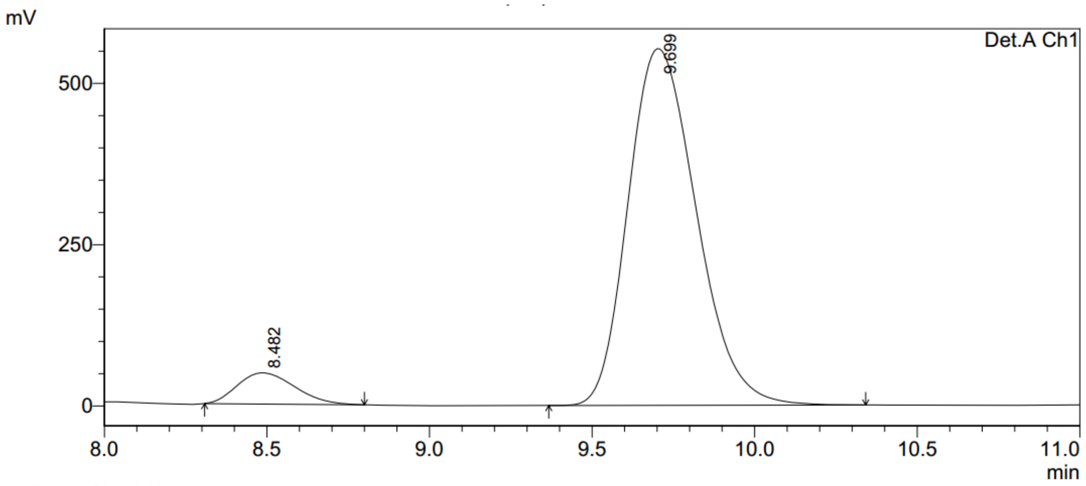

1 Det.A Ch1/210nm

PeakTable

检测器 A Ch1 210nm

Peak#	Ret. Time	Area	Height	Area %	Height %
1	8.482	596132	48347	6.874	8.041
2	9.699	8075640	552896	93.126	91.959
Total		8671772	601242	100.000	100.000

Supplementary Figure 269: HPLC traces for product **13**

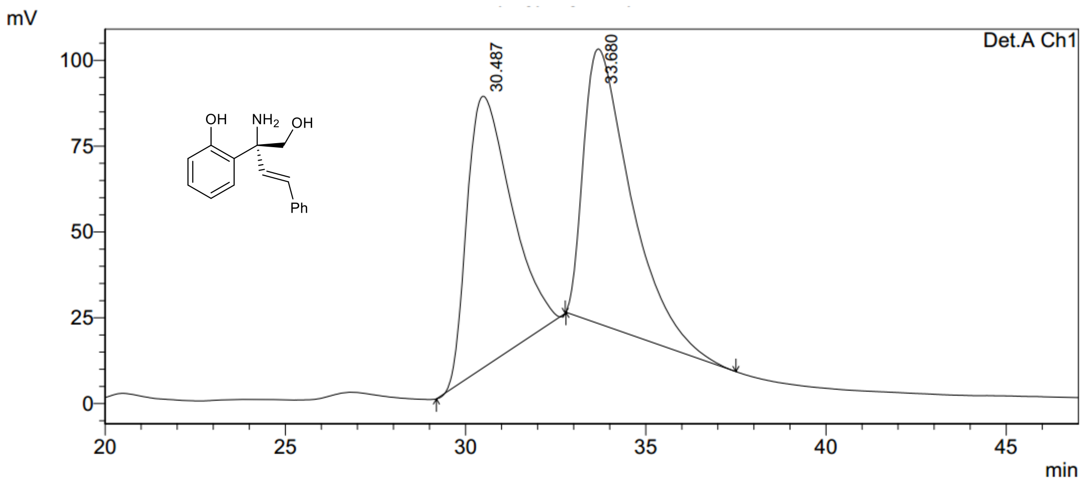

1 Det.A Ch1/210nm

PeakTable

Detector A Ch1 210nm

Peak#	Ret. Time	Area	Height	Area %	Height %
1	30.487	6771089	79133	47.926	49.726
2	33.680	7357245	80006	52.074	50.274
Total		14128334	159139	100.000	100.000

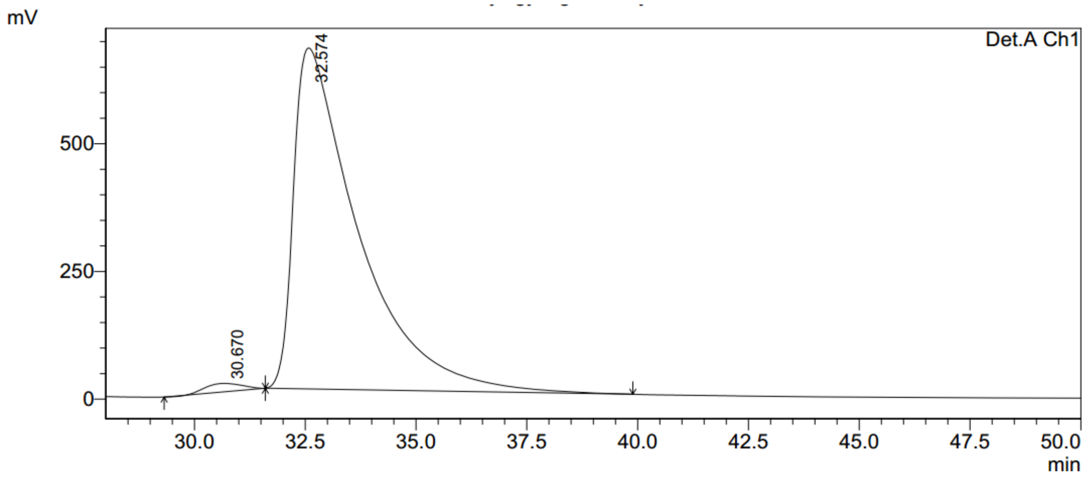

1 Det.A Ch1/210nm

PeakTable

Detector A Ch1 210nm

Peak#	Ret. Time	Area	Height	Area %	Height %
1	30.670	960265	16567	1.394	2.421
2	32.574	67933483	667614	98.606	97.579
Total		68893747	684181	100.000	100.000

Supplementary Figure 270: HPLC traces for product **SI-6**

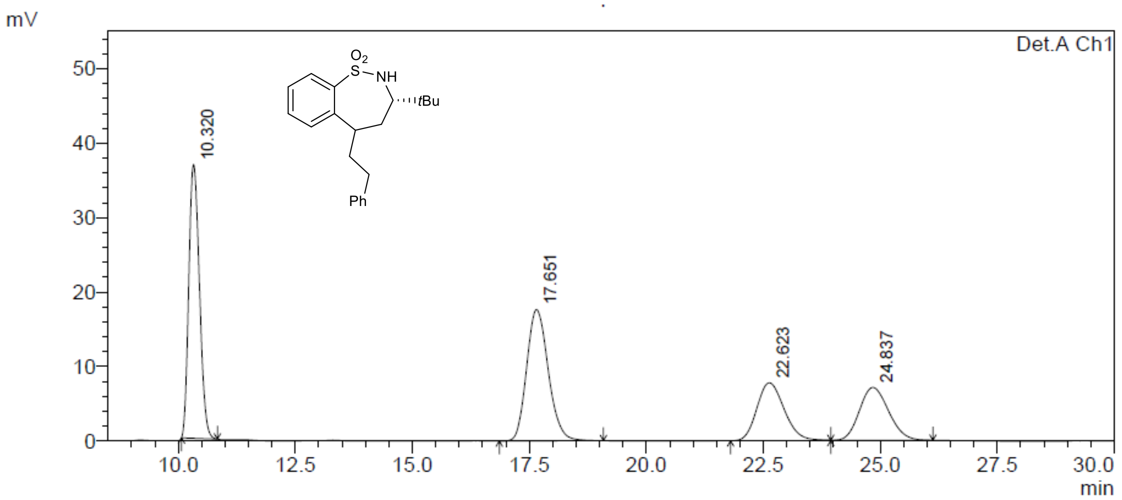

1 Det.A Ch1/254nm

PeakTable

检测器 A Ch1 254nm

Peak#	Ret. Time	Area	Height	Area %	Height %
1	10.320	581759	36775	32.790	53.076
2	17.651	567036	17634	31.960	25.450
3	22.623	315664	7766	17.792	11.208
4	24.837	309719	7114	17.457	10.267
Total		1774177	69288	100.000	100.000

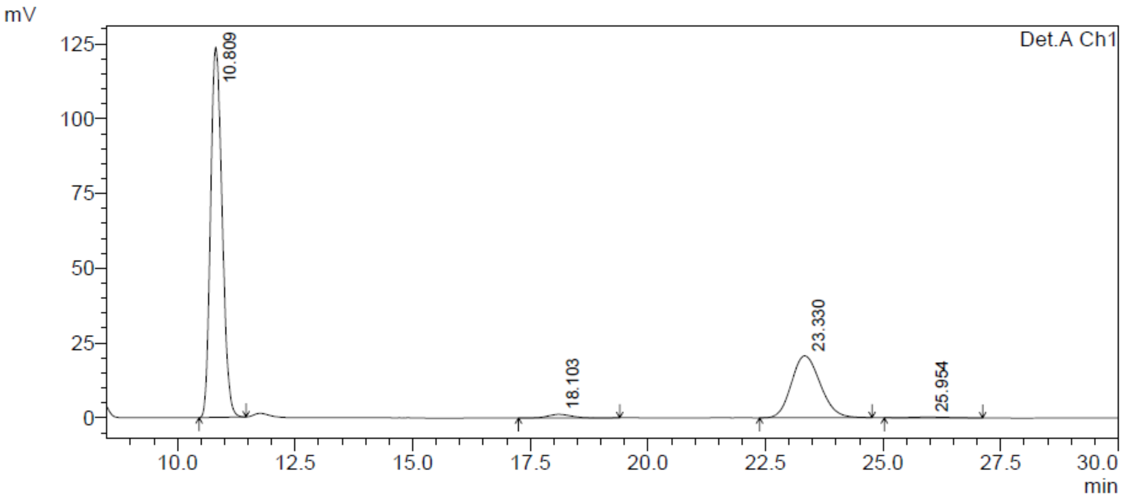

1 Det.A Ch1/254nm

PeakTable

检测器 A Ch1 254nm

Peak#	Ret. Time	Area	Height	Area %	Height %
1	10.809	2116190	123806	69.340	84.789
2	18.103	37107	1163	1.216	0.797
3	23.330	883763	20750	28.958	14.210
4	25.954	14848	298	0.487	0.204
Total		3051909	146017	100.000	100.000

Supplementary Figure 271: HPLC traces for product **14**

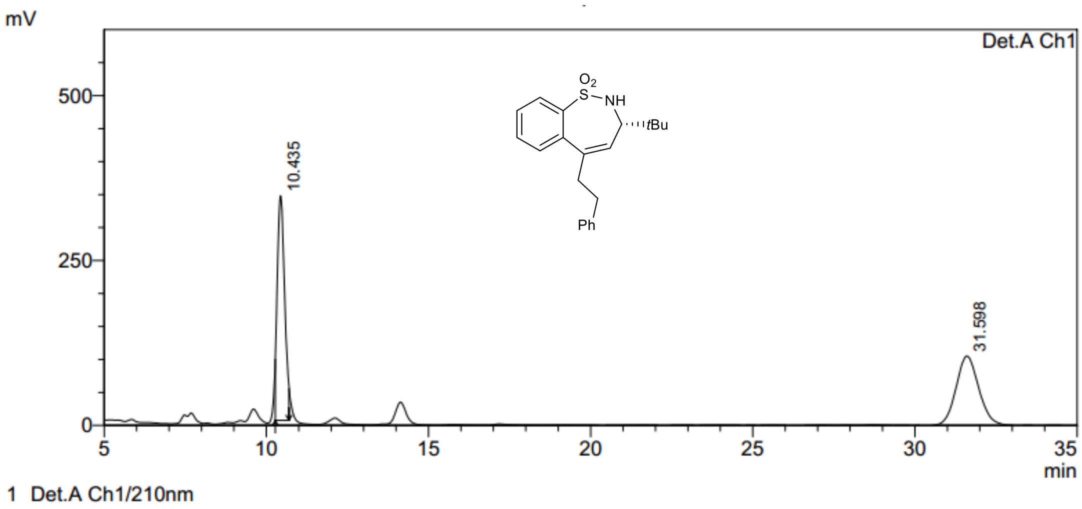

PeakTable

Detector A Ch1 210nm

Peak#	Ret. Time	Area	Height	Area %	Height %
1	10.435	5327547	340694	50.586	76.307
2	31.598	5204089	105785	49.414	23.693
Total		10531636	446478	100.000	100.000

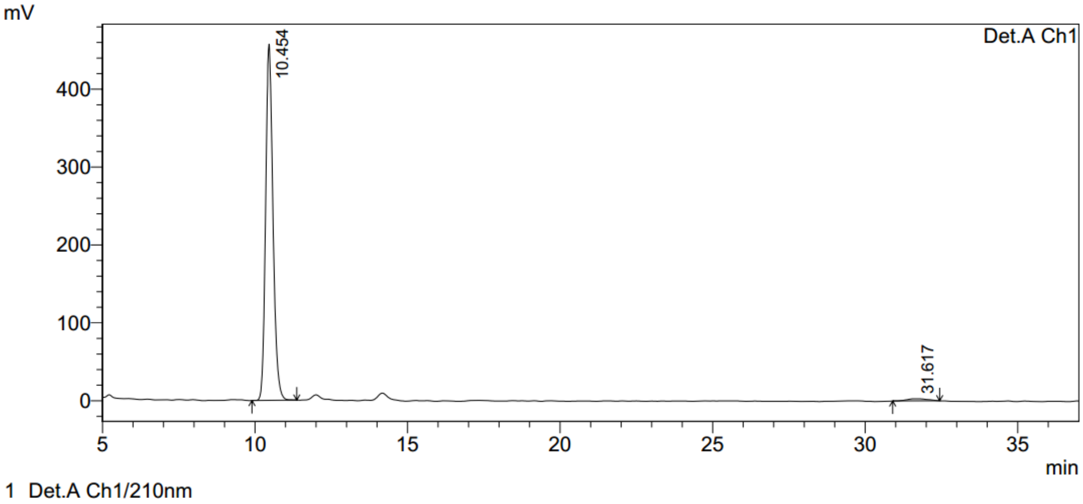

PeakTable

Detector A Ch1 210nm

Peak#	Ret. Time	Area	Height	Area %	Height %
1	10.454	7625149	457416	98.358	99.413
2	31.617	127323	2699	1.642	0.587
Total		7752472	460115	100.000	100.000

Supplementary Figure 272: HPLC traces for product **15**

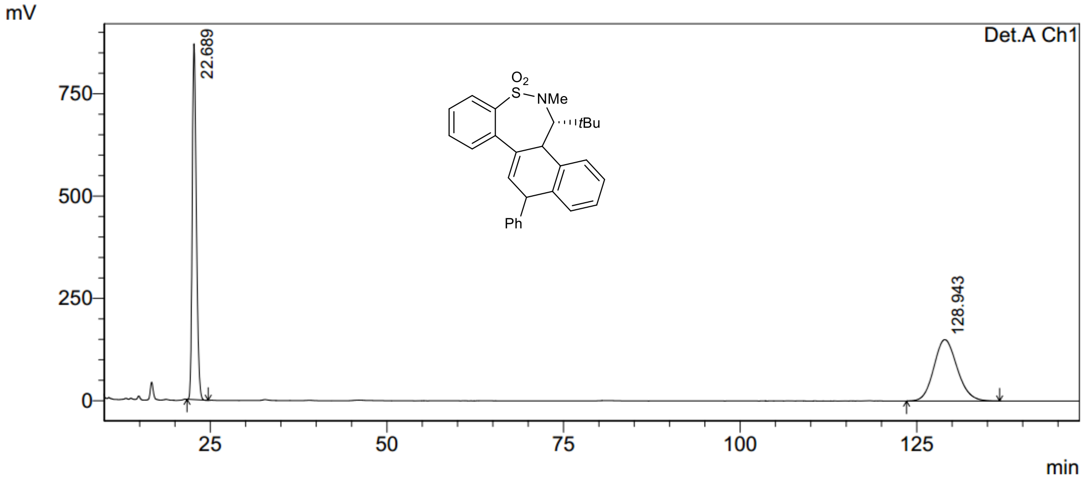

1 Det.A Ch1/210nm

PeakTable

检测器 A Ch1 210nm

Peak#	Ret. Time	Area	Height	Area %	Height %
1	22.689	35120602	869518	50.523	85.307
2	128.943	34393791	149761	49.477	14.693
Total		69514393	1019279	100.000	100.000

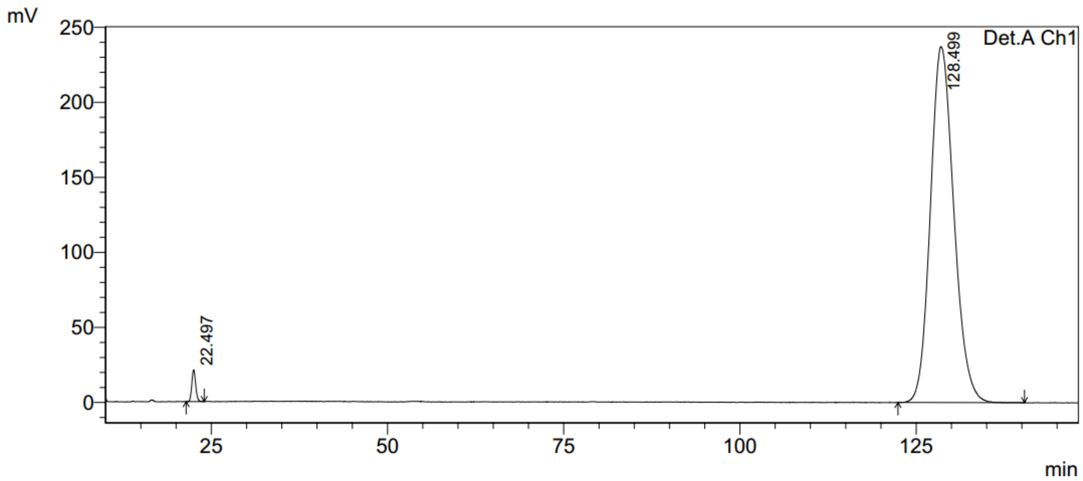

1 Det.A Ch1/210nm

PeakTable

检测器 A Ch1 210nm

Peak#	Ret. Time	Area	Height	Area %	Height %
1	22.497	809666	21171	1.450	8.194
2	128.499	55018037	237203	98.550	91.806
Total		55827704	258374	100.000	100.000

Supplementary Figure 273: HPLC traces for product **17**

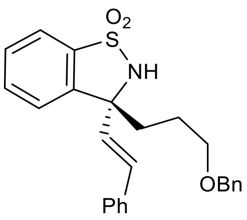

3na

The crystal data of compound **(3na)** have been deposited in CCDC with number 1486078. Empirical Formula: $C_{25}H_{25}NO_3S$; Formula Weight: 419.16; Crystal Color: Colorless. The crystal was obtained from a *i*PrOH solution at room temperature under N_2.

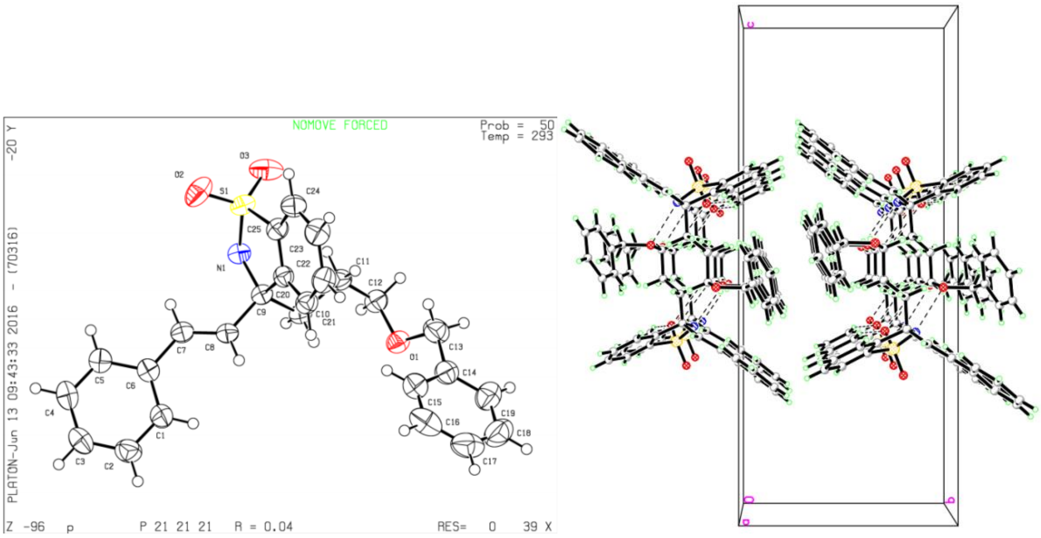

Supplementary Figure 274: X-ray analysis of crystal structure of **3na**

Supplementary Table 4.

Bond precision:	C-C = 0.0047 Å		Wavelength=1.54178
Cell:	a=7.6515(1)	b=11.0047(2)	c=26.0381(4)
	alpha=90	beta=90	gamma=90
Temperature:	293 K		

	Calculated	Reported
Volume	2192.47(6)	2192.47(6)
Space group	P 21 21 21	P 21 21 21
Hall group	P 2ac 2ab	P 2ac 2ab
Moiety formula	C25 H24 N O3 S	C25 H24 N O3 S
Sum formula	C25 H24 N O3 S	C25 H24 N O3 S
Mr	418.51	418.51
Dx,g cm-3	1.268	1.268
Z	4	4
Mu (mm-1)	1.518	1.518
F000	884.0	796.0
F000'	887.75	
h,k,lmax	9,13,31	9,13,31
Nref	4011[2318]	3832
Tmin,Tmax	0.930,0.955	
Tmin'	0.927	

Correction method= Not given

Data completeness= 1.65/0.96   Theta(max)= 68.219

R(reflections)= 0.0353(3616)   wR2(reflections)= 0.0986(3832)

S = 1.074   Npar= 272

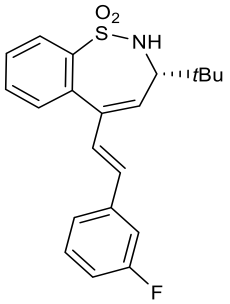

The crystal data of compound (**5f**) have been deposited in CCDC with number 1556426. Empirical Formula: $C_{21}H_{22}FNO_2S$; Formula Weight: 371.45; Crystal Color: Colorless. The crystal was obtained from a *i*PrOH solution at room temperature under N_2

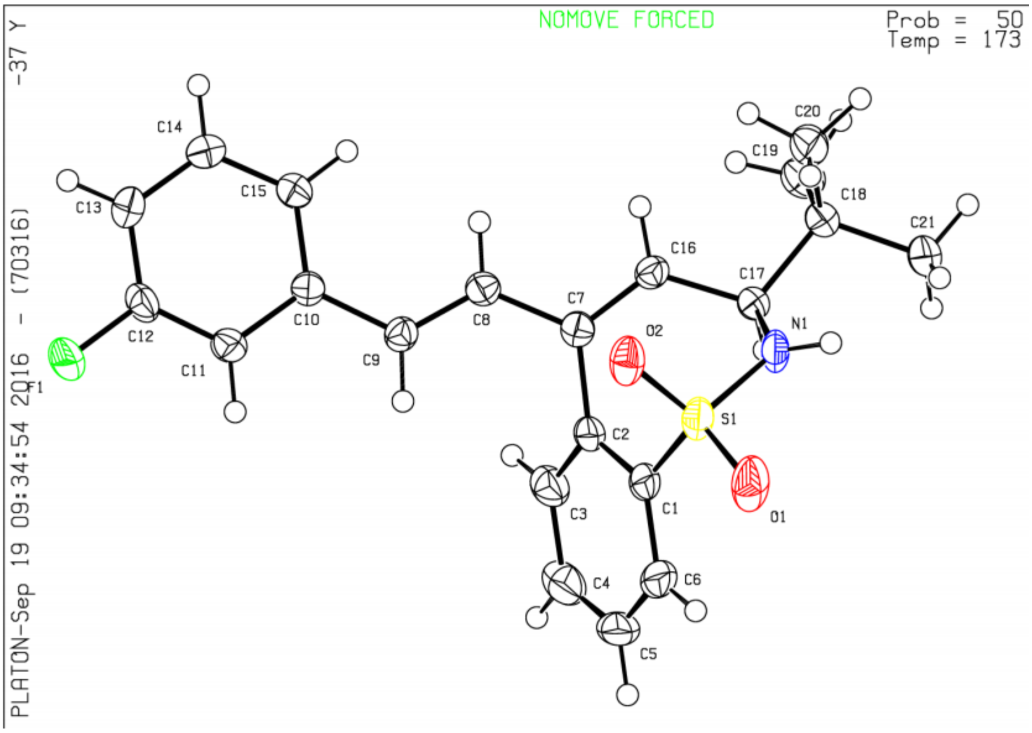

Supplementary Figure 275: X-ray analysis of crystal structure of **5f**

Supplementary Table 5.

Bond precision: C-C = 0.0074 A Wavelength=1.54178

Cell:	a=19.2452(3)	b=19.2452(3)	c=9.2133(2)
	alpha=90	beta=90	gamma=120
Temperature:	173 K		

	Calculated	Reported
Volume	2955.23(13)	2955.23(11)
Space group	P 65	P 65
Hall group	P 65	P 65
Moiety formula	C21 H22 F N O2 S	C21 H22 F N O2 S
Sum formula	C21 H22 F N O2 S	C21 H22 F N O2 S
Mr	371.46	371.45
Dx,g cm-3	1.252	1.252
Z	6	6
Mu (mm-1)	1.655	1.655
F000	1176.0	1176.0
F000’	1181.35	
h,k,lmax	23,23,11	23,23,11
Nref	3613[1930]	3603
Tmin,Tmax	0.683,0.767	
Tmin’	0.619	

Correction method= Not given

Data completeness= 1.87/1.00 Theta(max)= 68.254

R(reflections)= 0.0495(3471) wR2(reflections)= 0.1575(3603)

S = 1.021 Npar= 238

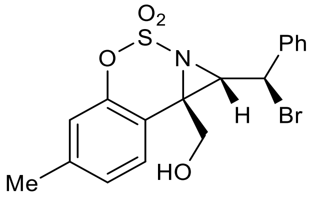

The crystal data of compound (**11**) have been deposited in CCDC with number 1816920. Empirical Formula: $C_{17}H_{16}BrNO_4S$; Formula Weight: 410.27; Crystal Color: Colorless. The crystal was obtained from a Et_2O solution at room temperature.

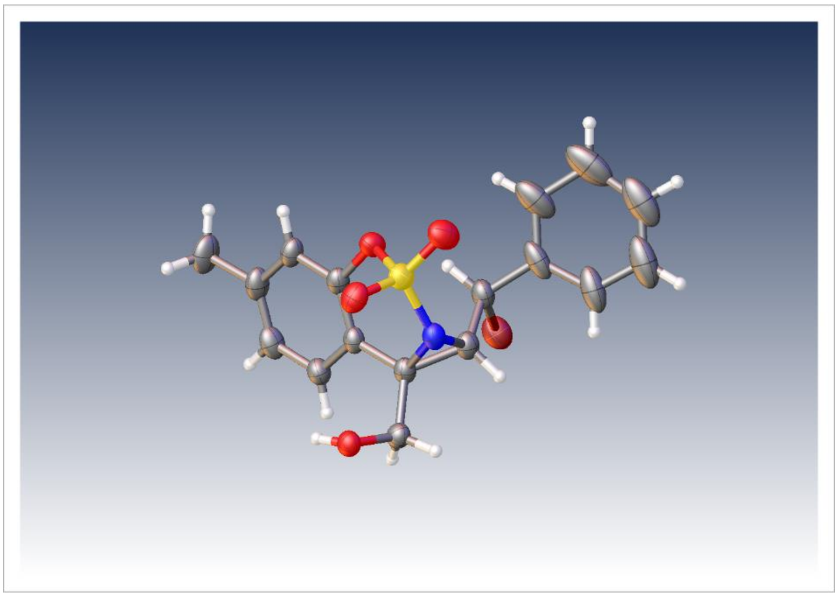

Supplementary Figure 276: X-ray analysis of crystal structure of **11**

Supplementary Table 6.

Bond precision:	C-C = 0.0137 A		Wavelength=1.34139
Cell:	a=16.1588(4)	b=16.1588(4)	c=6.7139(2)
	alpha=90	beta=90	gamma=120
Temperature:	170 K		

	Calculated	Reported
Volume	1518.18(10)	1518.18(9)
Space group	P 31	P 31
Hall group	P 31	P 31
Moiety formula	C17 H16 Br N O4 S [+ solvent]	C17 H16 Br N O4 S
Sum formula	C17 H16 Br N O4 S [+ solvent]	C17 H16 Br N O4 S
Mr	410.27	410.28
Dx,g cm-3	1.346	1.346
Z	3	3
Mu (mm-1)	2.569	2.630
F000	624.0	624.0
F000'	623.25	
h,k,lmax	19,19,8	19,19,8
Nref	3872[1936]	3851
Tmin,Tmax	0.969,0.974	0.522,0.751
Tmin'	0.854	

Correction method= # Reported T Limits: Tmin=0.522 Tmax=0.751
AbsCorr = MULTI-SCAN

Data completeness= 1.99/0.99 Theta(max)= 54.966

R(reflections)= 0.0418(3196) wR2(reflections)= 0.1137(3851)

S = 1.005 Npar= 219

SI-1

Supplementary Table 7.

Center Number	Atomic Number	Atomic Type	Coordinates (Angstroms) X	Y	Z
1	6	0	-2.382067	-2.440291	2.225311
2	6	0	-2.424056	-1.154214	1.695663
3	6	0	-1.993592	-0.951489	0.388158
4	6	0	-1.537679	-2.040055	-0.337425
5	6	0	-1.485054	-3.334156	0.164689
6	6	0	-1.919584	-3.522724	1.471155
7	1	0	-2.713749	-2.605204	3.245132
8	1	0	-2.786437	-0.321766	2.289422
9	1	0	-1.123919	-4.161572	-0.437465
10	1	0	-1.898576	-4.515201	1.907506
11	16	0	-0.925159	-1.508317	-1.895719
12	8	0	0.553559	-1.494625	-1.865244
13	8	0	-1.502676	-2.206458	-3.041522
14	6	0	-1.868587	0.383548	-0.333422
15	7	0	-1.592765	-0.009467	-1.736387
16	6	0	-0.737392	1.160455	0.319880
17	6	0	0.377222	1.585394	-0.275149
18	1	0	-0.918933	1.384407	1.370004
19	1	0	0.542799	1.395561	-1.332778
20	6	0	-3.126697	1.236004	-0.319445
21	6	0	-4.354689	0.791020	-0.065229
22	1	0	-2.947225	2.279281	-0.567724
23	1	0	-4.498628	-0.263777	0.172269
24	6	0	1.435661	2.357737	0.403583
25	6	0	2.109182	3.363376	-0.300340
26	6	0	1.794214	2.110373	1.733851
27	6	0	3.092563	4.126986	0.318936
28	1	0	1.855848	3.548032	-1.340799
29	6	0	2.785202	2.867754	2.349707
30	1	0	1.314774	1.303141	2.279390
31	6	0	3.432951	3.882147	1.647581
32	1	0	3.599551	4.908975	-0.238572
33	1	0	3.057323	2.659875	3.380190
34	1	0	4.207551	4.470867	2.129720
35	6	0	-5.630739	1.602422	-0.063607
36	6	0	-6.269942	1.466514	1.330105
37	1	0	-7.223604	2.004538	1.367922
38	1	0	-6.464754	0.416211	1.573876
39	1	0	-5.614466	1.879120	2.104665
40	6	0	-5.394643	3.081551	-0.378240
41	1	0	-4.730174	3.547603	0.357195
42	1	0	-4.952529	3.216985	-1.371142
43	1	0	-6.346297	3.622547	-0.359649
44	6	0	-6.576858	0.995375	-1.114997
45	1	0	-6.150833	1.078515	-2.120527
46	1	0	-6.765602	-0.064881	-0.913747
47	1	0	-7.540026	1.517334	-1.108021
48	5	0	2.548361	-1.844013	1.141211
49	6	0	1.275768	-1.463895	2.017081
50	6	0	1.072249	-1.805256	3.291051
51	1	0	0.497229	-0.876553	1.526764
52	1	0	0.174127	-1.519031	3.837431
53	1	0	1.803520	-2.387309	3.851296
54	8	0	1.962060	-2.990408	-0.046552
55	1	0	1.396504	-2.529004	-0.703596
56	8	0	3.541195	-2.575929	1.830551
57	1	0	4.269492	-2.852325	1.266571
58	1	0	1.426414	-3.686773	0.356067
59	8	0	2.927741	-0.763139	0.276762
60	6	0	3.986170	-0.948627	-0.614624
61	1	0	4.935319	-1.177040	-0.112427
62	1	0	3.785235	-1.738984	-1.348637
63	6	0	4.193586	0.336035	-1.379556
64	9	0	5.166488	0.186100	-2.299164
65	9	0	4.557184	1.348949	-0.576898
66	9	0	3.083737	0.722085	-2.034914
67	1	0	-1.220670	0.700189	-2.361652

SI-1Ts

Supplementary Table 8.

Imaginary Frequency = -29.0 cm^{-1}

Center Number	Atomic Number	Atomic Type	Coordinates (Angstroms) X	Y	Z
1	6	0	2.400368	-2.950619	-2.234185
2	6	0	2.596105	-1.611128	-1.912215
3	6	0	1.990203	-1.053634	-0.781287
4	6	0	1.211141	-1.879605	0.042544
5	6	0	1.042942	-3.222551	-0.265212
6	6	0	1.626034	-3.757742	-1.410827
7	1	0	2.867436	-3.361156	-3.123110

8	1	0	3.209820	-0.982858	-2.549032
9	1	0	0.460708	-3.847995	0.401137
10	1	0	1.485037	-4.807597	-1.644859
11	16	0	0.440395	-1.254846	1.532927
12	8	0	-0.788429	-0.488492	1.087233
13	8	0	0.003444	-2.453841	2.298826
14	6	0	2.193931	0.398511	-0.539579
15	7	0	1.576702	-0.384919	2.123067
16	6	0	1.068002	1.243908	-0.658757
17	6	0	1.112405	2.587594	-0.399918
18	1	0	0.127857	0.768937	-0.911136
19	1	0	2.062086	3.042169	-0.129877
20	6	0	3.529734	0.876768	-0.353232
21	6	0	4.531681	0.045264	0.015862
22	1	0	3.725048	1.936534	-0.465787
23	1	0	4.303875	-1.008168	0.175472
24	6	0	-0.003038	3.500861	-0.418374
25	6	0	0.268031	4.863928	-0.194025
26	6	0	-1.334617	3.089518	-0.625078
27	6	0	-0.757631	5.796823	-0.192710
28	1	0	1.293470	5.179107	-0.023451
29	6	0	-2.355478	4.025966	-0.615397
30	1	0	-1.570198	2.041849	-0.772037
31	6	0	-2.069351	5.376539	-0.404626
32	1	0	-0.540182	6.845727	-0.021825
33	1	0	-3.380054	3.707380	-0.767541
34	1	0	-2.875855	6.103280	-0.399631
35	6	0	5.956382	0.420053	0.280704
36	6	0	6.831338	-0.472343	-0.622531
37	1	0	7.888436	-0.281001	-0.411989
38	1	0	6.634004	-1.534381	-0.445182
39	1	0	6.648553	-0.260036	-1.680793
40	6	0	6.256419	1.895030	0.007611
41	1	0	6.042156	2.160843	-1.032834
42	1	0	5.672619	2.551544	0.661044
43	1	0	7.316123	2.094742	0.193145
44	6	0	6.241927	0.084928	1.758946
45	1	0	5.625482	0.693737	2.427710
46	1	0	6.045680	-0.970583	1.973081
47	1	0	7.294620	0.287996	1.981187
48	5	0	-3.485586	-2.478531	-0.105358
49	6	0	-2.298183	-3.054020	-1.008976
50	6	0	-2.174984	-4.319786	-1.412920
51	1	0	-1.523662	-2.351677	-1.331068
52	1	0	-1.349361	-4.662522	-2.035908
53	1	0	-2.907109	-5.078043	-1.136280
54	8	0	-2.847439	-2.028121	1.352769
55	1	0	-2.094326	-1.363403	1.251730
56	8	0	-4.470565	-3.457039	0.219652
57	1	0	-5.143870	-3.117104	0.816313
58	1	0	-2.453787	-2.785105	1.810687
59	8	0	-3.933053	-1.214436	-0.657651
60	6	0	-4.962611	-0.505865	-0.036172
61	1	0	-5.892676	-0.548904	-0.616296
62	1	0	-5.182866	-0.840592	0.984604
63	6	0	-4.555653	0.947313	0.056508
64	9	0	-5.543002	1.700806	0.572411
65	9	0	-4.255778	1.456305	-1.156270
66	9	0	-3.470441	1.124297	0.831532
67	1	0	1.196389	0.200416	2.868405

SI-2

Supplementary Table 9.

Center Number	Atomic Number	Atomic Type	Coordinates (Angstroms) X	Y	Z
1	6	0	-2.458970	-2.939284	2.254942
2	6	0	-2.649484	-1.605139	1.909774
3	6	0	-2.018331	-1.059018	0.786238
4	6	0	-1.218066	-1.894375	-0.008898
5	6	0	-1.056850	-3.232971	0.321242
6	6	0	-1.664033	-3.754804	1.460420
7	1	0	-2.944893	-3.338588	3.138840
8	1	0	-3.277236	-0.969909	2.525550
9	1	0	-0.458406	-3.864812	-0.323924
10	1	0	-1.525869	-4.801305	1.710770
11	16	0	-0.410148	-1.302130	-1.494445
12	8	0	0.801094	-0.514218	-1.037041
13	8	0	0.062171	-2.517084	-2.213083
14	6	0	-2.217543	0.390633	0.531621
15	7	0	-1.535510	-0.460302	-2.141115
16	6	0	-1.089422	1.233791	0.640402
17	6	0	-1.130972	2.577141	0.375675
18	1	0	-0.150677	0.757227	0.894483
19	1	0	-2.078465	3.030017	0.094664
20	6	0	-3.550579	0.872000	0.336230
21	6	0	-4.555217	0.044993	-0.037073
22	1	0	-3.742081	1.932848	0.443707
23	1	0	-4.335559	-1.010747	-0.190538

24	6	0	-0.017638	3.492454	0.402508
25	6	0	-0.286003	4.851489	0.150507
26	6	0	1.309719	3.088609	0.648943
27	6	0	0.737693	5.786356	0.156737
28	1	0	-1.307938	5.161583	-0.047338
29	6	0	2.328655	4.026996	0.648241
30	1	0	1.543890	2.044783	0.822813
31	6	0	2.045031	5.372901	0.406510
32	1	0	0.522350	6.832072	-0.035225
33	1	0	3.349692	3.713578	0.831597
34	1	0	2.849948	6.101335	0.408323
35	6	0	-5.976668	0.428402	-0.307884
36	6	0	-6.856558	-0.448684	0.605941
37	1	0	-7.912514	-0.243311	0.402751
38	1	0	-6.675622	-1.513764	0.428952
39	1	0	-6.662739	-0.237140	1.662325
40	6	0	-6.266768	1.908197	-0.050616
41	1	0	-6.068421	2.179679	0.991510
42	1	0	-5.665537	2.555466	-0.697547
43	1	0	-7.321139	2.116267	-0.256227
44	6	0	-6.268360	0.078837	-1.781598
45	1	0	-5.653377	0.681533	-2.457096
46	1	0	-6.071835	-0.978372	-1.986353
47	1	0	-7.321994	0.279707	-2.001022
48	5	0	3.531785	-2.455435	0.082158
49	6	0	2.334177	-3.041775	0.963893
50	6	0	2.197319	-4.314465	1.340376
51	1	0	1.559506	-2.340894	1.288076
52	1	0	1.360142	-4.663446	1.944379
53	1	0	2.928090	-5.072243	1.059784
54	8	0	2.898981	-1.981165	-1.375028
55	1	0	2.119615	-1.350149	-1.252878
56	8	0	4.517087	-3.431023	-0.247235
57	1	0	5.230540	-3.070527	-0.782193
58	1	0	2.538015	-2.735822	-1.862518
59	8	0	3.973794	-1.197443	0.648703
60	6	0	4.987907	-0.473149	0.019217
61	1	0	5.930342	-0.519799	0.578563
62	1	0	5.184445	-0.789301	-1.012354
63	6	0	4.564466	0.976925	-0.040773
64	9	0	5.537019	1.752123	-0.552443
65	9	0	4.270678	1.459754	1.183802
66	9	0	3.468687	1.153866	-0.801402
67	1	0	-1.143791	0.086818	-2.909337

SI-2Ts1

Supplementary Table 10.

Imaginary Frequency = -46.4 cm^{-1}

Center Number	Atomic Number	Atomic Type	Coordinates (Angstroms) X	Y	Z
1	6	0	3.379133	-3.310965	0.166089
2	6	0	3.197628	-1.938515	0.239203
3	6	0	2.055386	-1.327903	0.804753
4	6	0	1.062783	-2.226832	1.319905
5	6	0	1.229548	-3.595134	1.185370
6	6	0	2.366954	-4.147977	0.598914
7	1	0	4.285462	-3.710716	-0.274664
8	1	0	3.954309	-1.312251	-0.209107
9	1	0	0.463874	-4.250892	1.580357
10	1	0	2.454831	-5.224999	0.504097
11	16	0	-0.380223	-1.812565	2.344802
12	8	0	-1.399130	-1.187564	1.409770
13	8	0	-0.930074	-3.101888	2.830458
14	6	0	1.899988	0.104236	0.505187
15	7	0	0.249802	-0.864405	3.387464
16	6	0	0.762371	0.853391	0.833241
17	6	0	0.707875	2.214275	0.618729
18	1	0	-0.096874	0.371361	1.266209
19	1	0	1.614543	2.737977	0.321337
20	6	0	2.816972	0.762890	-0.450997
21	6	0	4.092439	1.070130	-0.190728
22	1	0	2.339484	1.088974	-1.370309
23	1	0	4.519777	0.765298	0.765766
24	6	0	-0.459030	3.030619	0.802613
25	6	0	-0.310241	4.431073	0.760018
26	6	0	-1.742635	2.477062	0.991806
27	6	0	-1.409623	5.257542	0.930265
28	1	0	0.676420	4.856067	0.600879
29	6	0	-2.837421	3.309204	1.148984
30	1	0	-1.882871	1.401658	0.987306
31	6	0	-2.671295	4.695655	1.126176
32	1	0	-1.289441	6.335241	0.904810
33	1	0	-3.824113	2.880079	1.280215
34	1	0	-3.533892	5.342362	1.253356
35	6	0	5.029827	1.841118	-1.085710
36	6	0	6.226057	0.922519	-1.397782
37	1	0	6.970839	1.464051	-1.990895
38	1	0	6.711589	0.575639	-0.479360

39	1	0	5.909353	0.043671	-1.969368
40	6	0	4.367625	2.287988	-2.390438
41	1	0	4.012251	1.433125	-2.975472
42	1	0	3.517862	2.953313	-2.202627
43	1	0	5.089616	2.835465	-3.004538
44	6	0	5.522832	3.070833	-0.302780
45	1	0	4.692129	3.739821	-0.053815
46	1	0	6.013761	2.776372	0.630894
47	1	0	6.245401	3.634479	-0.902487
48	5	0	-1.780943	-1.910822	-1.960728
49	6	0	-0.204398	-1.646139	-1.936265
50	6	0	0.725940	-2.446567	-2.460404
51	1	0	0.142091	-0.734206	-1.443641
52	1	0	1.791249	-2.223213	-2.411265
53	1	0	0.458648	-3.372163	-2.970355
54	8	0	-2.208840	-2.676075	-0.540678
55	1	0	-1.932942	-2.128276	0.259013
56	8	0	-2.185087	-2.819555	-2.981633
57	1	0	-3.119861	-3.039696	-2.922618
58	1	0	-1.786625	-3.542048	-0.461445
59	8	0	-2.486587	-0.654433	-1.838604
60	6	0	-3.881623	-0.662429	-1.902843
61	1	0	-4.253056	-0.488423	-2.920775
62	1	0	-4.330786	-1.590300	-1.527163
63	6	0	-4.401856	0.457111	-1.031923
64	9	0	-5.741973	0.547204	-1.113019
65	9	0	-3.896525	1.650659	-1.388154
66	9	0	-4.093999	0.272472	0.267730
67	1	0	-0.454558	-0.627801	4.088408

SI-3

Supplementary Table 11.

Center Number	Atomic Number	Atomic Type	Coordinates (Angstroms) X	Y	Z
1	6	0	3.820364	-1.531609	2.675365
2	6	0	2.578322	-0.932009	2.511370
3	6	0	1.730816	-1.287774	1.452232
4	6	0	2.188718	-2.240317	0.520177
5	6	0	3.442354	-2.818002	0.676720
6	6	0	4.252290	-2.480236	1.757696
7	1	0	4.448906	-1.249009	3.513106
8	1	0	2.240257	-0.190473	3.227915
9	1	0	3.793330	-3.522709	-0.068344
10	1	0	5.224764	-2.948612	1.866986
11	16	0	1.302211	-2.676610	-0.985625
12	8	0	0.682907	-1.386495	-1.472393
13	8	0	2.344809	-3.109030	-1.953970
14	6	0	0.426116	-0.595370	1.401847
15	7	0	0.304339	-3.749148	-0.484033
16	6	0	-0.767779	-1.307680	1.213776
17	6	0	-1.911734	-0.683758	0.773063
18	1	0	-0.725998	-2.388190	1.290107
19	1	0	-1.838554	0.356662	0.472646
20	6	0	0.439873	0.818653	1.623113
21	6	0	-0.534277	1.472727	2.300306
22	1	0	1.322025	1.354909	1.285525
23	1	0	-1.315433	0.889252	2.787533
24	6	0	-3.196055	-1.299856	0.562596
25	6	0	-4.197639	-0.530329	-0.058861
26	6	0	-3.480864	-2.626925	0.939126
27	6	0	-5.451804	-1.072092	-0.297690
28	1	0	-3.975592	0.491402	-0.351183
29	6	0	-4.734448	-3.162556	0.695484
30	1	0	-2.724042	-3.235022	1.423251
31	6	0	-5.719130	-2.387435	0.078087
32	1	0	-6.219919	-0.474732	-0.777248
33	1	0	-4.950824	-4.185744	0.983767
34	1	0	-6.699698	-2.814215	-0.108285
35	6	0	-0.590461	2.951689	2.543602
36	6	0	-0.217705	3.186657	4.022046
37	1	0	-0.312332	4.252075	4.256600
38	1	0	-0.877305	2.628108	4.694031
39	1	0	0.814684	2.880728	4.218517
40	6	0	0.351685	3.737746	1.627348
41	1	0	1.403943	3.512679	1.830877
42	1	0	0.155881	3.514786	0.574002
43	1	0	0.207617	4.810947	1.786015
44	6	0	-2.045602	3.402064	2.312301
45	1	0	-2.344204	3.252451	1.271428
46	1	0	-2.738650	2.846960	2.952950
47	1	0	-2.144335	4.467155	2.544626
48	5	0	2.159669	1.681716	-1.896522
49	6	0	3.056753	1.352013	-0.611751
50	6	0	3.892414	2.208114	-0.019018
51	1	0	2.978702	0.357841	-0.166109
52	1	0	4.470972	1.945081	0.866207
53	1	0	4.036525	3.219404	-0.398612

54	8	0	2.076472	0.306089	-2.807828
55	1	0	1.564819	-0.416961	-2.315142
56	8	0	2.748090	2.672293	-2.735282
57	1	0	2.202363	2.881163	-3.499383
58	1	0	2.945205	-0.049575	-3.037048
59	8	0	0.763674	1.860326	-1.527752
60	6	0	-0.121902	2.263011	-2.528586
61	1	0	-0.152762	3.353710	-2.647151
62	1	0	0.097664	1.815158	-3.506556
63	6	0	-1.516121	1.813061	-2.162283
64	9	0	-2.412132	2.253838	-3.062109
65	9	0	-1.903101	2.283521	-0.955743
66	9	0	-1.626108	0.475534	-2.107771
67	1	0	-0.272768	-4.054253	-1.270162

SI-4

Supplementary Table 12.

Center Number	Atomic Number	Atomic Type	Coordinates (Angstroms) X	Y	Z
1	6	0	-1.120779	-1.932478	3.350432
2	6	0	-0.751392	-0.802640	2.627074
3	6	0	-1.310731	-0.532736	1.375993
4	6	0	-2.238812	-1.459594	0.871362
5	6	0	-2.621162	-2.588969	1.586619
6	6	0	-2.059836	-2.821998	2.837552
7	1	0	-0.670999	-2.116918	4.320528
8	1	0	-0.020179	-0.111971	3.035492
9	1	0	-3.333628	-3.286411	1.160329
10	1	0	-2.346068	-3.704412	3.399576
11	16	0	-2.843068	-1.298436	-0.798241
12	8	0	-1.669684	-1.215383	-1.671655
13	8	0	-3.813565	-2.358367	-1.066864
14	6	0	-0.900151	0.684770	0.625191
15	7	0	-3.683506	0.127859	-0.884921
16	6	0	0.540100	0.993477	0.629786
17	6	0	1.028071	2.230202	0.803586
18	1	0	1.210258	0.144554	0.517733
19	1	0	0.326225	3.042852	0.988201
20	6	0	-1.789466	1.448517	-0.029955
21	6	0	-3.282146	1.235152	0.020848
22	1	0	-1.414683	2.270532	-0.631257
23	1	0	-3.552744	0.957285	1.050319
24	6	0	2.450632	2.604612	0.823713
25	6	0	2.812496	3.858821	1.333452
26	6	0	3.465437	1.750948	0.365205
27	6	0	4.148099	4.243045	1.406089
28	1	0	2.036631	4.535206	1.683026
29	6	0	4.798863	2.135776	0.438918
30	1	0	3.215263	0.787278	-0.064957
31	6	0	5.147151	3.380331	0.962803
32	1	0	4.407461	5.216700	1.810763
33	1	0	5.570144	1.461240	0.078904
34	1	0	6.190189	3.676628	1.019016
35	6	0	-4.096815	2.510936	-0.324278
36	6	0	-3.686832	3.606845	0.669984
37	1	0	-4.280726	4.509840	0.496408
38	1	0	-3.854500	3.286765	1.704490
39	1	0	-2.632033	3.879684	0.566504
40	6	0	-3.842425	2.982876	-1.760457
41	1	0	-2.795449	3.253219	-1.929533
42	1	0	-4.114010	2.211203	-2.486689
43	1	0	-4.447448	3.871132	-1.970014
44	6	0	-5.595979	2.225865	-0.143342
45	1	0	-5.986829	1.539688	-0.902772
46	1	0	-5.804272	1.801414	0.845237
47	1	0	-6.163467	3.157088	-0.236852
48	5	0	2.924606	-2.375326	0.389654
49	6	0	2.764063	-1.818315	1.841735
50	6	0	2.465876	-2.595506	2.887442
51	1	0	2.857923	-0.744325	2.008512
52	1	0	2.323685	-2.193198	3.888491
53	1	0	2.346071	-3.672279	2.780788
54	8	0	0.170807	-3.156713	-0.371147
55	1	0	-0.152784	-2.365179	-0.823984
56	8	0	3.195695	-3.696894	0.203035
57	1	0	3.213576	-3.991930	-0.713483
58	1	0	0.226709	-2.894183	0.554883
59	8	0	2.785749	-1.458718	-0.628543
60	6	0	2.731828	-1.837864	-1.980433
61	1	0	3.702357	-2.185675	-2.348370
62	1	0	1.976783	-2.611673	-2.151316
63	6	0	2.334778	-0.617713	-2.778982
64	9	0	2.228604	-0.929621	-4.080458
65	9	0	3.238937	0.371458	-2.671982
66	9	0	1.153939	-0.118365	-2.380037
67	1	0	-4.673399	-0.096374	-0.800298

SI-2Ts2

Supplementary Table 13.

Imaginary Frequency = -199.1 cm^{-1}

Center Number	Atomic Number	Atomic Type	Coordinates (Angstroms) X	Y	Z
1	6	0	-2.715338	-2.491308	2.462237
2	6	0	-2.710221	-1.134728	2.156484
3	6	0	-2.155183	-0.654280	0.964988
4	6	0	-1.583882	-1.585609	0.080916
5	6	0	-1.598085	-2.943424	0.385044
6	6	0	-2.162664	-3.401211	1.570479
7	1	0	-3.152210	-2.831359	3.395491
8	1	0	-3.138646	-0.423958	2.856630
9	1	0	-1.164673	-3.643000	-0.320059
10	1	0	-2.167927	-4.463623	1.790086
11	16	0	-0.792960	-1.137003	-1.466098
12	8	0	0.587113	-0.622207	-1.098697
13	8	0	-0.625709	-2.401587	-2.232088
14	6	0	-2.237583	0.824321	0.746707
15	7	0	-1.760708	-0.071996	-2.032695
16	6	0	-0.970230	1.555993	0.631180
17	6	0	-0.808993	2.873267	0.825957
18	1	0	-0.114653	0.937081	0.383428
19	1	0	-1.656888	3.481363	1.140182
20	6	0	-3.433273	1.448968	0.806983
21	6	0	-4.781835	0.969804	0.624016
22	1	0	-3.432849	2.509982	0.528040
23	1	0	-5.356257	0.629581	1.490272
24	6	0	0.450979	3.609589	0.653510
25	6	0	0.549931	4.914529	1.156177
26	6	0	1.551708	3.068596	-0.027301
27	6	0	1.719240	5.651694	1.000136
28	1	0	-0.299106	5.349127	1.677208
29	6	0	2.716744	3.807427	-0.187761
30	1	0	1.491582	2.070816	-0.448193
31	6	0	2.806437	5.100440	0.326855
32	1	0	1.779508	6.659877	1.398954
33	1	0	3.554088	3.379373	-0.729193
34	1	0	3.718160	5.675283	0.196444
35	6	0	-5.425261	0.783442	-0.649258
36	6	0	-6.949648	0.846460	-0.579069
37	1	0	-7.374764	0.453899	-1.506461
38	1	0	-7.336960	0.259235	0.258694
39	1	0	-7.273229	1.883496	-0.460730
40	6	0	-4.838961	1.505160	-1.852691
41	1	0	-5.097917	2.566830	-1.807588
42	1	0	-3.753014	1.394808	-1.899931
43	1	0	-5.268380	1.085558	-2.766008
44	6	0	-4.944077	-0.756589	-0.652249
45	1	0	-3.944248	-0.823325	-1.077378
46	1	0	-5.006001	-1.294248	0.298533
47	1	0	-5.688327	-1.209190	-1.314422
48	5	0	2.997764	-2.794035	0.265196
49	6	0	1.770652	-3.074173	1.250852
50	6	0	1.482433	-4.252439	1.806977
51	1	0	1.116479	-2.232911	1.498671
52	1	0	0.638718	-4.397457	2.480629
53	1	0	2.092315	-5.134534	1.612549
54	8	0	2.380645	-2.461572	-1.241129
55	1	0	1.697359	-1.716269	-1.209229
56	8	0	3.832537	-3.931825	0.066417
57	1	0	4.532485	-3.766787	-0.571938
58	1	0	1.928969	-3.229620	-1.616518
59	8	0	3.622865	-1.538348	0.618065
60	6	0	4.726133	-1.084761	-0.105390
61	1	0	5.662007	-1.208228	0.453320
62	1	0	4.845994	-1.567671	-1.083861
63	6	0	4.545026	0.393406	-0.369131
64	9	0	5.635294	0.921404	-0.958027
65	9	0	4.325026	1.083011	0.761214
66	9	0	3.500384	0.633307	-1.188333
67	1	0	-1.358353	0.329443	-2.881294

SI-5

Supplementary Table 14.

Center Number	Atomic Number	Atomic Type	Coordinates (Angstroms) X	Y	Z
1	6	0	-2.867557	-1.765017	2.606155
2	6	0	-2.803062	-0.506535	2.016091
3	6	0	-2.229876	-0.311379	0.755847
4	6	0	-1.654038	-1.429296	0.120029
5	6	0	-1.725914	-2.689678	0.707866
6	6	0	-2.341654	-2.865401	1.942907
7	1	0	-3.330006	-1.879630	3.581338
8	1	0	-3.198281	0.354434	2.543681

9	1	0	-1.288644	-3.536113	0.192438
10	1	0	-2.400442	-3.855420	2.382785
11	16	0	-0.761521	-1.354294	-1.438054
12	8	0	0.592897	-0.749551	-1.117882
13	8	0	-0.550659	-2.764543	-1.868337
14	6	0	-2.255736	1.085028	0.213685
15	7	0	-1.672356	-0.467862	-2.320971
16	6	0	-0.932093	1.741323	0.150722
17	6	0	-0.698680	3.026228	0.451966
18	1	0	-0.110278	1.087897	-0.129152
19	1	0	-1.519612	3.648214	0.808626
20	6	0	-3.360231	1.776096	-0.121491
21	6	0	-4.844279	1.472866	-0.179622
22	1	0	-3.173501	2.788341	-0.472748
23	1	0	-5.315311	2.347121	-0.643827
24	6	0	0.607291	3.700571	0.362715
25	6	0	0.732825	5.003086	0.864737
26	6	0	1.735977	3.096897	-0.212333
27	6	0	1.946438	5.681338	0.803311
28	1	0	-0.133390	5.485330	1.310608
29	6	0	2.947107	3.774170	-0.274104
30	1	0	1.669420	2.093098	-0.617970
31	6	0	3.059066	5.068417	0.233139
32	1	0	2.023678	6.688464	1.202220
33	1	0	3.809454	3.290256	-0.721020
34	1	0	4.007205	5.594810	0.181249
35	6	0	-5.381553	0.303144	-0.895093
36	6	0	-6.770146	0.360278	-1.353517
37	1	0	-7.289334	-0.582414	-1.157915
38	1	0	-7.331087	1.225159	-1.002387
39	1	0	-6.682098	0.421616	-2.453188
40	6	0	-4.612633	-0.888666	-1.169003
41	1	0	-5.172893	-1.633327	-1.732426
42	1	0	-3.658885	-0.623304	-1.675326
43	1	0	-4.284517	-1.299572	-0.202474
44	6	0	-5.530555	1.380880	1.243619
45	1	0	-5.352549	0.416721	1.723652
46	1	0	-5.092917	2.166810	1.861446
47	1	0	-6.604120	1.558217	1.160239
48	5	0	2.939745	-2.895329	0.386034
49	6	0	1.648380	-3.108395	1.303848
50	6	0	1.290907	-4.250341	1.894446
51	1	0	0.999524	-2.242792	1.463189
52	1	0	0.395716	-4.338393	2.509618
53	1	0	1.884161	-5.158076	1.786886
54	8	0	2.404646	-2.583827	-1.153746
55	1	0	1.737531	-1.825276	-1.158744
56	8	0	3.745886	-4.064916	0.261679
57	1	0	4.523831	-3.920727	-0.285214
58	1	0	1.921573	-3.343627	-1.509596
59	8	0	3.583817	-1.650571	0.751448
60	6	0	4.701976	-1.202095	0.046688
61	1	0	5.618056	-1.281554	0.644974
62	1	0	4.867267	-1.722152	-0.904842
63	6	0	4.498992	0.260436	-0.279482
64	9	0	5.592080	0.787567	-0.861649
65	9	0	4.239607	0.986690	0.820792
66	9	0	3.469399	0.450600	-1.128707
67	1	0	-1.215620	-0.303898	-3.218610

Supplementary References

1. An, Q., Shen, J., Butt, N., Liu, D., Liu, Y., Zhang, W. The Construction of 3-Methyl-4-Arylpiperidines via a *trans*-Perhydroindolic Acid-Catalyzed Asymmetric Aza-Diels–Alder Reaction. *Adv. Synth. Catal.* **357**, 3627–3638 (2015).

2. Davis, F. A., Towson, J. C., Vashi, D. B., ThimmaReddy, R., McCauleyJr., J. P., Harakal, M. E., Gosciniak, D. J. Synthesis, Reactions, and Properties of 3-Substituted-1,2-benzisothiazole 1,1-Dioxide Oxides. *J. Org. Chem.* **55**, 1254–1261 (1990).

3. Christine, K. F. H., James, A. C., Thomas, D. G., Judith, A. L., James, F. W. Aromatic Heteroannulation via Metalation-Cyclization of *N*-Acyl-2-Chlorobenzenesulfonamides and *N*-Acylbenzenesulfonamides. *J. Org. Chem.* **57**, 5328–5334 (1992).

4. Ahn, K. H., Ham, C., Kim, S.-K., Cho, C.-W. Practical Synthesis of Chiral Sultam Auxiliaries: 3-Substituted-1,2-benzisothiazoline-1,1-Dioxides. *J. Org. Chem.* **62**, 7047–7048 (1997).

5. Michael, R., Takeo, F., Jeffrey, W. B. Cyclic Ketimines as Superior Electrophiles for NHC-Catalyzed Homoenolate Additions with Broad Scope and Low Catalyst Loadings. *J. Am. Chem. Soc.* **130**, 17266–17267 (2008).

6. Yang, G., Zhang, W. A Palladium-Catalyzed Enantioselective Addition of Arylboronic Acids to Cyclic Ketimines. *Angew. Chem. Int. Ed.* **52**, 7540−7544 (2013).

7. Feng, X., Zhou, Z., Ma, C., Yin, X., Li, R., Dong, L., Chen, Y.-C. Trienamines Derived from Interrupted Cyclic 2,5-Dienones: Remote δ,ε-C=C Bond Activation for Asymmetric Inverse-Electron-Demand Aza-Diels–Alder Reaction. *Angew. Chem. Int. Ed.* **52**, 14173–14176 (2013).

8. Zhang, Q.-R., Huang, J.-R., Zhang, W., Dong, L. Highly Functionalized Pyridines Synthesis from *N*-Sulfonyl Ketimines and Alkynes Using the N–S Bond as an Internal Oxidant. *Org. Lett.* **16**, 1684–1687 (2014).

9. Hepburn, H. B., Lam, H. W. The Isomerization of Allylrhodium Intermediates in the Rhodium-Catalyzed Nucleophilic Allylation of Cyclic Imines. *Angew. Chem. Int. Ed.* **53**, 11605–11610 (2014).

10 Song, B., Yu, C.-B., Huang, W.-X., Chen, M.-W., Zhou, Y.-G. Formal Palladium-Catalyzed Asymmetric Hydrogenolysis of Racemic *N*-Sulfonyloxaziridines. *Org. Lett.* **17**, 190–193 (2015).

11. Quan, M., Yang, G., Xie, F., Gridnev, I. D., Zhang, W. Pd(II)-Catalyzed Asymmetric Addition of Arylboronic Acids to Cyclic *N*-Sulfonyl Ketimine Esters and a DFT Study of Its Mechanism. *Org. Chem. Front.* **2**, 398–402 (2015).

12. Li, E., Jin, H., Jia, P., Dong, X., Huang, Y. Bifunctional-Phosphine-Catalyzed Sequential Annulations of Allenoates and Ketimines: Construction of Functionalized Poly-heterocycle Rings. *Angew. Chem. Int. Ed.* **55**, 11591–11594 (2016).

13. Deng, C., Wang, L.-J., Zhu, J., Tang, Y. A Chiral Cagelike Copper(I) Catalyst for the Highly Enantioselective Synthesis of 1,1-Cyclopropane Diesters. *Angew. Chem. Int. Ed.* **51**, 11620–11623 (2012).

14. Zhang, Y.-F., Chen, D., Chen, W.-W., Xu, M.-H. Construction of Cyclic Sulfamidates Bearing Two *gem*-Diaryl Stereocenters through a Rhodium-Catalyzed Stepwise Asymmetric Arylation Protocol. *Org. Lett.* **18**, 2726–2729 (2016).

15. Gaussian 09, Revision A.02, Frisch, M. J., Trucks, G. W., Schlegel, H. B., Scuseria, G. E., Robb, M. A., Cheeseman, J. R., Scalmani, G., Barone, V., Mennucci, B., G. Petersson, A.,

Nakatsuji, H., Caricato, M., Li, X., H. Hratchian, P., Izmaylov, A. F., Bloino, J., Zheng, G., Sonnenberg, J. L., Hada, M., Ehara, M., Toyota, K., Fukuda, R., Hasegawa, J., Ishida, M., Nakajima, T., Honda, Y., Kitao, O., Nakai, H., Vreven, T., Montgomery, J. A., Peralta, J. E., Ogliaro, F., Bearpark, M., Heyd, J. J., Brothers, E., Kudin, K. N., Staroverov, V. N., Kobayashi, R., Normand, J., Raghavachari, K., Rendell, A., Burant, J. C., Iyengar, S. S., Tomasi, J., Cossi, M., Rega, N., Millam, J. M., Klene, M., Knox, J. E., Cross, J. B., Bakken, V., Adamo, C., Jaramillo, J., Gomperts, R., Stratmann, R. E., Yazyev, O., Austin, A. J., Cammi, R., Pomelli, C., Ochterski, J. W., Martin, R. L., Morokuma, K., Zakrzewski, V. G., Voth, G. A., Salvador, P., Dannenberg, J. J., Dapprich, S., Daniels, A. D., Farkas, O., Foresman,J. B., Ortiz, J. V., Cioslowski, J., Fox, D. J. *Gaussian, Inc. Wallingford CT*, 2009.

16. Denmark, S. E., Burk, M. T. Lewis Base Catalysis of Bromo- and Iodolactonization, and Cycloetherification. *PNAS* **107**, 20655–20660 (2010).

17. Yao, Y., Li, J.-L., Zhou, Q.-Q., Dong, L., Chen, Y.-C. Enantioselective Aza-Morita–Baylis–Hillman Reaction with Ketimines and Acrolein Catalyzed by Organic Assemblies. *Chem. Eur. J.* **19**, 9447–9451 (2013).

18. Luo, Y., Carnell, A. J., Lam, H. W. Enantioselective Rhodium-Catalyzed Addition of Potassium Alkenyltrifluoroborates to Cyclic Imines. *Angew. Chem. Int. Ed.* **51**, 6762−6766 (2012).
